# Why Are 5-Thioglycopyranosyl Donors More Axially Selective than their Glycopyranosyl Counterparts? A Low and Variable Temperature NMR Spectroscopy and Computational Study

**DOI:** 10.1021/jacsau.4c01113

**Published:** 2025-01-23

**Authors:** Daniil Ahiadorme, R. Houston Givhan, Henry F. Schaefer, David Crich

**Affiliations:** † Department of Chemistry, University of Georgia, 302 East Campus Road, Athens, Georgia 30602, United States; ‡ Department of Pharmaceutical and Biomedical Sciences, University of Georgia, 250 West Green Street, Athens, Georgia 30602, United States; § Center for Computational Quantum Chemistry, University of Georgia, 1004 Cedar St, Athens, Georgia 30602, United States; ∥ Complex Carbohydrate Research Center, University of Georgia, 315 Riverbend Road, Athens, Georgia 30602, United States

**Keywords:** 5-thioglycopyranosyl donors, reactive intermediates, nuclear magnetic resonance (NMR)

## Abstract

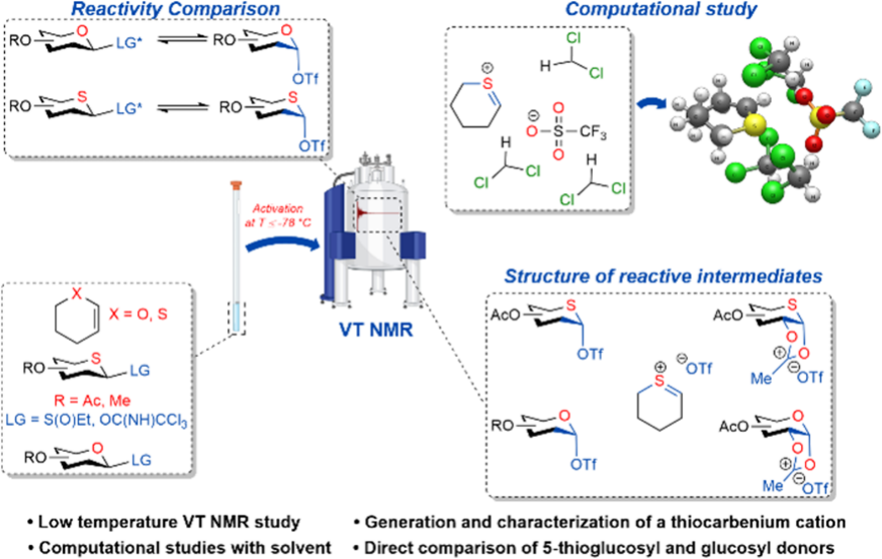

5-Thioglycopyranosyl donors differ in reactivity and selectivity from simple glycopyranosyl donors. An extensive study has been conducted on the nature and stability of the reactive intermediates generated on the activation of per-*O*-acetyl and per-*O*-methyl 5-thioglucopyranosyl donors and the corresponding glucopyranosyl donors. Variable temperature nuclear magnetic resonance (NMR) studies with per-*O*-methylated or per-*O*-acetyl glycosyl sulfoxides and trichloroacetimidates on activation with trifluoromethanesulfonic anhydride or trimethylsilyl triflate are reported. These show that following initial adduct formation with the promoter conversion of the 5-thioglucopyranosyl donors to the 5-thioglucopyranosyl triflates requires higher temperatures than that of the glucopyranosyl donors to the glucopyranosyl triflates. It is demonstrated that neighboring group participation is a less important phenomenon for the peracetylated thioglucosyl donors than for the peracetylated glucosyl donors. A simple thiocarbenium ion was generated by protonation of 2,3-dihydro-4*H*-thiopyran at low temperature and characterized by NMR spectroscopy. However, the corresponding 5-thioglucopyranosyl thenium ions were not observed in any of the NMR studies of the 5-thiopyranosyl donors: the electron-withdrawing C–O bonds around the thiopyranoside core discourage thiocarbenium ion formation, just as they discourage oxocarbenium ion formation. Density functional theory (DFT) calculations reveal the tetrahydrothiopyranyl thiocarbenium ion to be approximately 2.5 kcal/mol lower in energy than the corresponding tetrahydropyranyl oxocarbenium ion relative to the corresponding covalent triflates. However, the computations reveal a 5.8 kcal/mol activation barrier for conversion of the tetrahydrothiopyranyl triflate to the thiocarbenium ion, while formation of the oxocarbenium ion–triflate ion pair from tetrahydropyranyl triflate requires only 2.6 kcal·mol^–1^. Overall, the greater axial selectivity of 5-thioglycopyranosyl donors compared to analogous glycopyranosyl donors derives from (i) the lower kinetic reactivity necessitating higher reaction temperatures, (ii) the greater stability of the thiocarbenium ion over the oxocarbenium ion facilitating equilibration under thermodynamic conditions, (iii) the greater magnitude of the anomeric effect in the 5-thiosugars, and (iv) decreased neighboring group participation in the per-esterified 5-thiosugars.

## Introduction

Thiosugars are sugars in which hydroxy groups are replaced by thiols.^1^ When appropriately placed, such substitutions result in the replacement of the ring oxygen of the cyclic forms of sugars by divalent sulfur, as in the case of the furanose and pyranose forms of 4-thioglucose and 5-thioglucose, respectively. Such cyclic thiosugars, limited examples of which occur naturally,^2,3^ are of interest as mimics of the parent sugars owing to the potential for increased affinity with drug binding pockets and use as glycosidase and glycosyltransferase inhibitors,^4−7^ and consequently, several synthetic routes to them have been developed.^4−6,8−11^ The inclusion of the sulfur atom, with its longer bonds, in the furanose or pyranose ring results in increased ring puckering, which, in turn, causes reorientation of the C–O bonds.^12,13^ These changes often lead to reduced affinity for binding sites optimized for the natural ligand,^12−15^ and in remarkably polar compounds due to the realignment of the C–OH bonds on puckering. The anomeric effect is also enhanced in 5-thiosugars relative to simple sugars, presumably because of the longer C–S bonds and enhanced puckering, which reduce 1,3-diaxial interactions.^13^

**Scheme 1:**
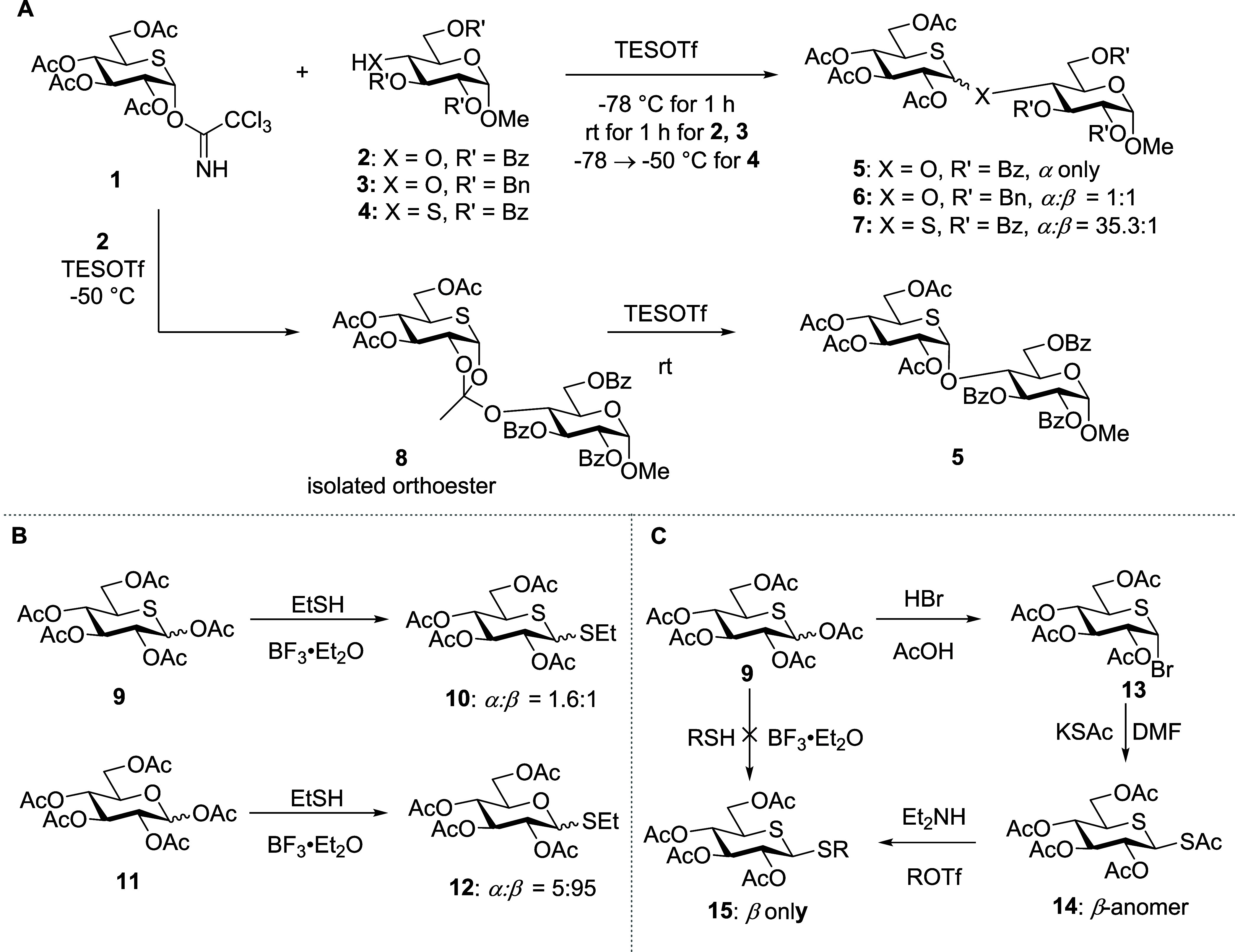
(A) Glycosylation Reactions with 5-Thioglycosyl Trichloroacetoimidate **1**, (B) Comparison of Thioglycosylation Reactions of Peracetylated 5-Thioglucopyranosyl Donor **9** and Peracetylated Glucopyranosyl Donor **11**, and (C) Alternative Synthetic Route for β-Thioglucosides

The well-understood changes in structure and properties brought about by the replacement of the ring oxygen by sulfur stand in contrast to the influence of this modification on reactivity and selectivity at the anomeric center. Thus, aqueous acidic hydrolysis of methyl 5-thioglucopyranoside and 5-thioxylopyranoside occurs more rapidly and takes place through fully solvent equilibrated thiocarbenium ions, than does that of the corresponding methyl glycosides when full equilibration of any oxocarbenium ions is not achieved, suggestive of the greater stability of thiocarbenium ions than of their oxocarbenium ion congeners.^16−21^ In contrast, UDP-*N*-acetyl 5-thioglucosamine is an excellent inhibitor of O-GlcNAc transferase by which it is turned over some 3200 times more slowly than the normal substrate, although both bind in the same manner as demonstrated crystallographically.^22^ Likewise, 5-thioglycosides inhibit glycoside hydrolases for which they are poor substrates.^23−26^ In synthetic chemistry, 5-thioglycosyl donors carrying equatorial esters at the 2-position suitable for the provision of equatorial glycosides by neighboring group participation are axially selective under typical Lewis acidic conditions (Scheme 1A,B). This is the case even though the formation of an intermediate orthoester has been demonstrated.^27−30^ Such axial selectivity is typically rationalized in terms of the enhanced stability of thiocarbenium ions and the consequent ease of equilibration to the more stable product.^7,23,25^ Alternative strategies for equatorial 5-thioglycoside synthesis have consequently been developed, which minimize the potential for equilibration (Scheme 1C).^31,32^

The 4-thiofuranosides have been less studied than the 5-thiopyranosides. However, it has been reported that the 4-thionucleosides undergo acid-catalyzed hydrolysis more slowly than their oxygen congeners,^33^ have therapeutic potential,^34−38^ and have applications as tools in biochemistry.^39,40^ So-inspired, Codée and co-workers prepared a series of perbenzylated 4-thiopentafuranosyl acetates and studied the stereoselectivity of their reactions with the weakly nucleophilic deuteriotriethylsilane on activation with trimethylsilyl triflate in dichloromethane at −30 °C. They found that all observed selectivities could be rationalized on the basis of the attack of the silane on intermediate thiocarbenium ions, for which a full conformational analysis was conducted computationally in the absence of any counterion.^10^ Of note is a recent study in which mesylation of 2-deoxy-2,2-difluoro-3,5-di-*O*-benzoyl-α,β-d-ribofuranose gave rise to a 79% isolated yield of a 1:4 α:β mixture of thiofuranosyl mesylates from which the β-isomer was isolated and fully characterized crystallographically.^41^ Thus was established the first example of a crystallographically characterized glycosyl sulfonate of any kind in spite of the intense interest in their chemistry.^42−44^

**Scheme 2:**
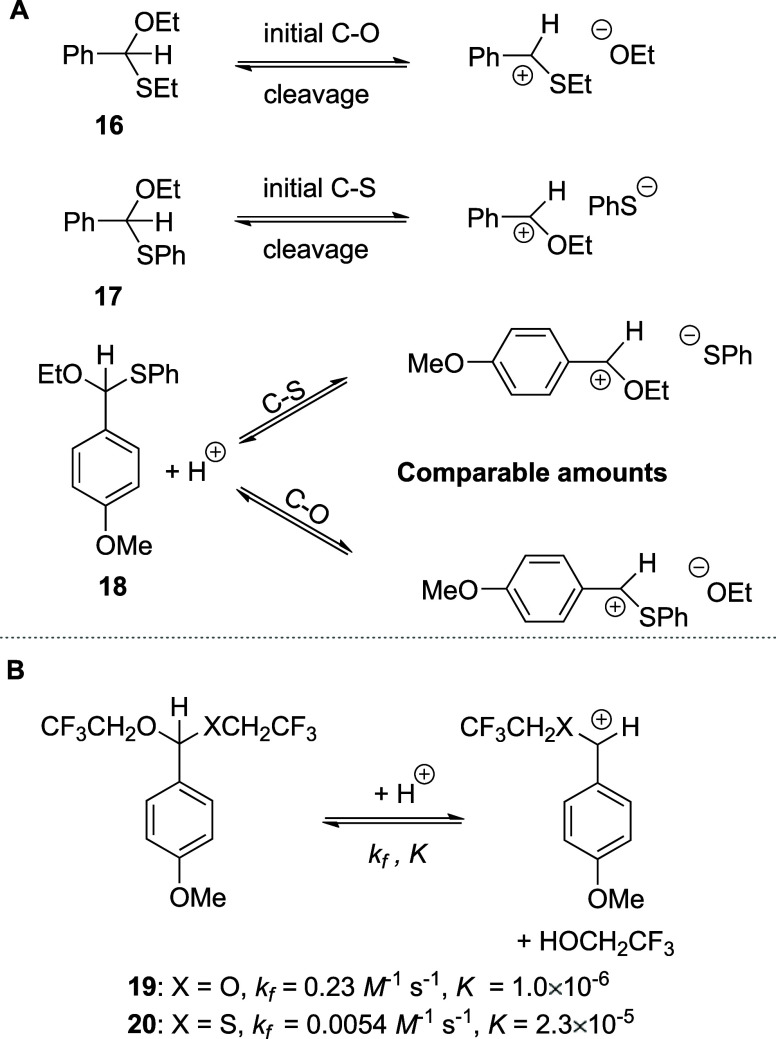
(A) Hydrolysis of the Benzaldehyde Monothioacetals **16**, **17**, and **18**. (B) Hydrolysis of Monothioacetals **19** and **20**. *k*_f_ —Rate Constant for Cation Formation; *K*—Equilibrium Constant

Beyond carbohydrates, extensive comparative studies on the hydrolysis of simple acetals and monothioacetals reveal the dependence of the mechanism on substituents and conditions, with the preferential formation of oxocarbenium ion intermediates in some instances and thiocarbenium ions in others. Thus, for example, Jensen and Jencks studied hydrolysis of benzaldehyde *O*,*S*-acetals and found that switching from the SEt group in **16** to the SPh group in **17** changes the mechanism of hydrolysis, with initial C–S bond cleavage preferred over C–O bond cleavage for **17** (Scheme 2A).^45,46^ On the other hand, for **18**, comparable amounts of C–O and C–S bond cleavage were observed, which was rationalized by the increased resonance stabilization of the intermediate cations in both cases. Richard and co-workers found that simple monothioacetals **20** were cleaved more slowly than the corresponding acetals **19** under solvolytic conditions (Scheme 2B) even though the thiocarbenium ions were judged to have longer lifetimes.^47^ This dichotomy was rationalized in terms of a lower kinetic barrier for the formation of the less stable oxocarbenium ion owing to the smaller degree of resonance stabilization at the transition state for the formation of the thiocarbenium ion.^47^

**Scheme 3:**
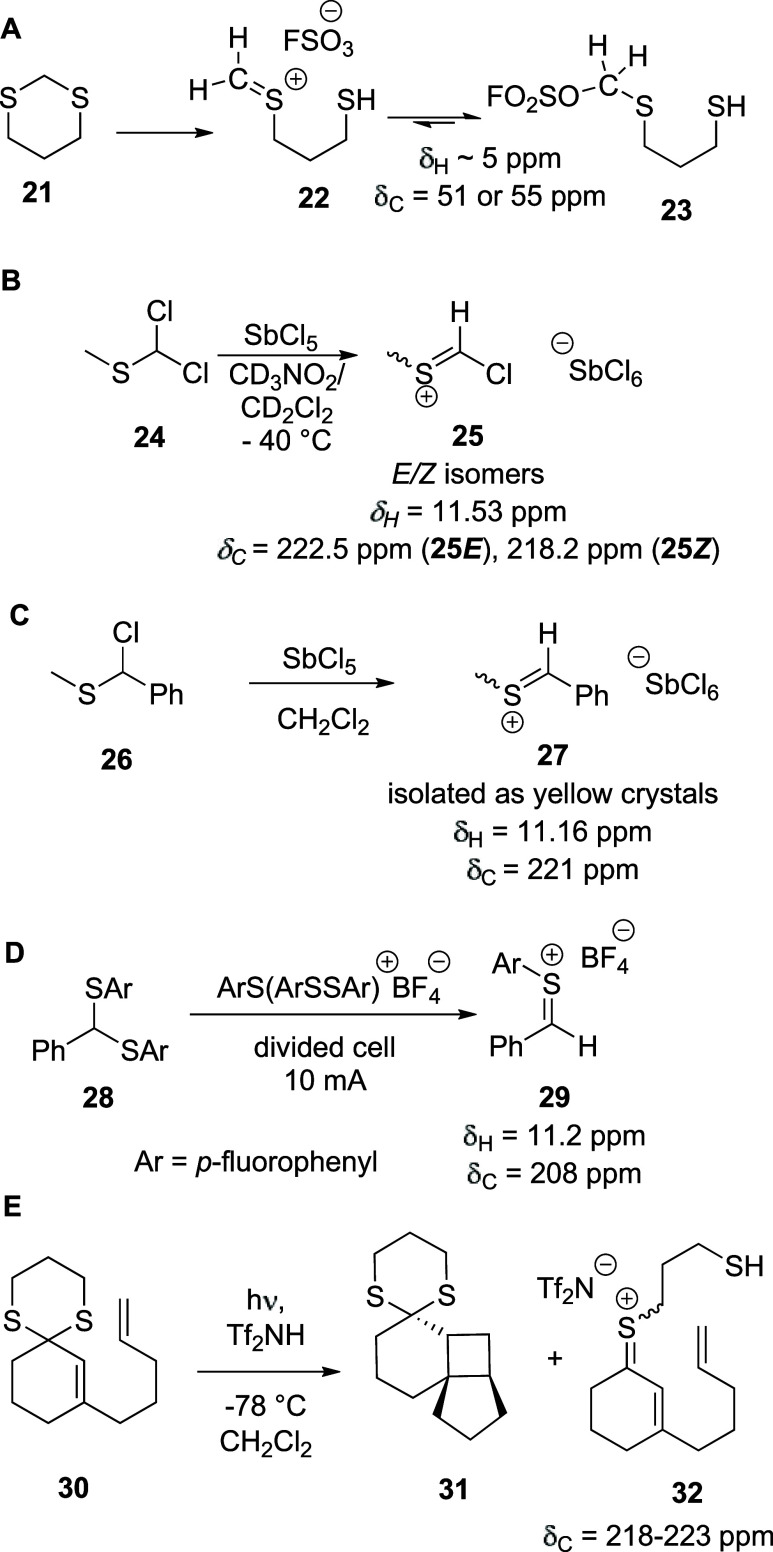
(A) Reactive Intermediates Generated from 1,3-Dithiane in Superacid, (B) α-Chlorothienium Ion Generation, (C) Benzylic Thiocarbenium Ion Generation, (D) Benzylic Thiocarbenium Ion Generated by the Cation Pool Method, and (E) α,β-Unsaturated Thiocarbenium Ion **32** Observed by NMR Spectroscopy after Protonation of 1,3-Dithiane **30**

Surprisingly, in view of the much-discussed stability^48−50^ of thiocarbenium ions, their spectroscopic characterization in the solution phase is sparse in contrast to that of simple oxocarbenium ions in both superacid and organic media.^51^ In early work, Lambert and co-workers dissolved 1,3-dithiane in fluorosulfonic acid and tentatively identified a signal at δ ∼ 5 ppm as belonging to the sp^2^C-bound hydrogen atom in ion **22** (Scheme 3A).^52^ The likely inconsistency of this chemical shift with structure **22** was recognized, however, leading to the alternative suggestion of an unidentified solvolysis product.^52^ In view of the current knowledge of oxocarbenium ions and their covalent adducts with the counterions,^21,51,53−57^ this can be tentatively assigned to the covalent fluorosulfonate **23**. Hartke and Akgün described a thiocarbenium ion **25** (Scheme 3B) generated by treating dichloromethyl methyl sulfide **24** with SbCl_5_ in CD_3_NO_2_/CD_2_Cl_2_^58^ and recorded its ^1^H and ^13^C NMR spectra, assigning signals at δ_*c*_ = 222.5 and 218.2 ppm to the cationic carbon in forms **25*****E*** and **25*****Z***, respectively.^58^ Subsequently, the phenyl (methylthio)methylium salt **27** was generated and successfully isolated as yellow crystals (Scheme 3C);^59^ its ^1^H and ^13^C NMR spectra showed signals similar to those observed for **25**, most notably a downfield carbeniun ion-type signal at δ_*C*_ = 221 ppm in the ^13^C NMR spectrum. Yoshida and co-workers used their cation pool method to generate the benzylic thiocarbenium ion **29** at −78 °C in CD_2_Cl_2_ and record its ^1^H and ^13^C NMR spectra, which showed peaks δ_H_ = 11.2 ppm and δ_C_ = 208 ppm corresponding to the thiocarbenium ion (Scheme 3D).^60^ Finally, Brenninger and Bach protonated dithiane **30** with triflimide in CD_2_Cl_2_ at −78 °C and observed by ^1^H and ^13^C NMR spectroscopy a substance ascribed to ion **32** with a characteristic downfield signal at δ_*C*_ = 218–223 ppm (Scheme 3E).^61^ Thus, to the best of our knowledge, at the outset of this study, simple thiocarbenium ions lacking stabilizing substituents had not been characterized spectroscopically. Likewise, in contrast to the multiple NMR spectra of 2-deoxyglycosyl cations described in superacidic media,^57,62^ there were no reports in the literature characterizing reactive intermediates generated from activated 5-thioglycosyl donors.

With this background in mind, seeking to shed light on the mechanisms and stereoselectivity of glycosylation by 5-thiopyranosyl donors, we have conducted and report here an extensive experimental and computational investigation into the nature of the intermediates generated on the activation of ether- and ester-protected 5-thioglucopyranosyl donors under two different sets of conditions in comparison to the corresponding *O*-glycosyl donors.

## Results and Discussion

**Scheme 4:**
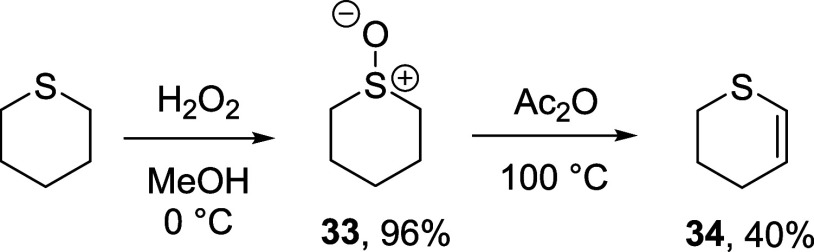
Synthesis of Dihydrothiopyran **34**

In view of the paucity of NMR data on thiocarbenium ions, we first set out to generate and characterize a simple unstabilized six-membered cyclic thiocarbenium ion in deuteriodichloromethane, which is the most widely used solvent for glycosylation reactions. To this end, 2,3-dihydro-4*H*-thiopyran **34** was accessed by a literature method^63,64^ involving oxidation of thiopyran with hydrogen peroxide to the sulfoxide **33** in 96% yield, followed by Pummerer reaction with acetic anhydride, which gave the desired product **34** in 40% isolated yield (Scheme 4).

The addition of 1.1 equiv of TfOH to a 0.25 M solution of **34** in CD_2_Cl_2_ precooled to −78 °C in the probe of the NMR spectrometer and rapid acquisition of ^1^H and ^13^C NMR spectra revealed a complex mixture that nevertheless contained what was subsequently identified as a trace amount of the desired thiocarbenium species **35**. After termination of the VT NMR experiment, a complex mixture of self-condensation products was obtained, in which dimer **36** could be identified by mass spectrometric analysis and by the presence of diagnostic signals in the ^1^H NMR spectrum that matched those reported in the literature (see Supporting Information).^65^

**Scheme 5:**
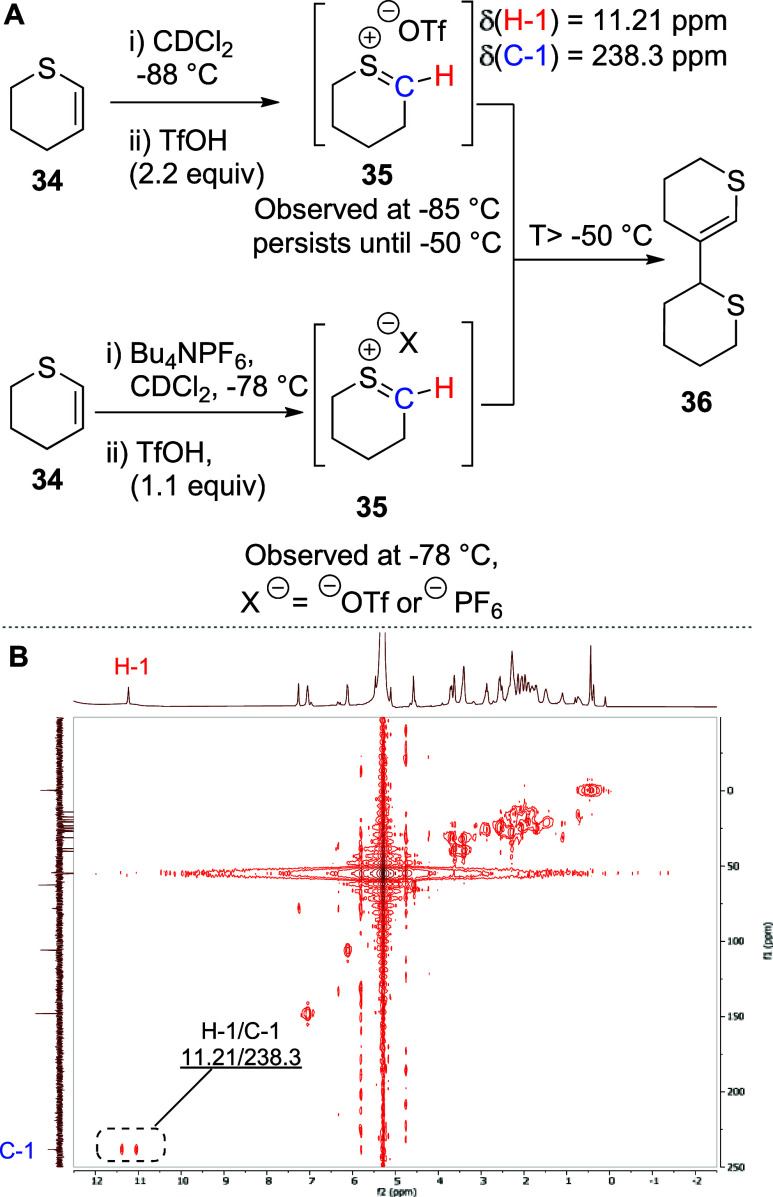
(A) Reactive Intermediates and Products Generated during VT NMR Experiments with 2,3-Dihydro-4*H*-thiopyran **34** and (B) Partial 2D HMQC Spectrum of Cation **35** at −85 °C after Protonation of **34** with 2.2 Equiv of TfOH

Seeking to increase the polarity of the reaction mixture and taking inspiration from the Yoshida cation pool method,^66^ we admixed **35** with freshly dried Bu_4_NPF_6_ (0.3 M in CD_2_Cl_2_) before adding 1.1 equiv of TfOH at −78 °C. In this manner, we were able to generate improved spectra of the thiocarbenium cation **35**, which was characterized by a peak at δ_H_ = 11.21 ppm corresponding to the proton attached directly to the thiocarbenium carbon. In the DEPT-135 spectrum, a downfield resonance at δ_c_ = 238.3 ppm was observed, which showed an HMQC correlation to the proton at δ_H_ = 11.21 ppm and was therefore assigned as the sp^2^-hybridized cationic carbon (Scheme 5A,B). On gradual warming, these signals persisted until ∼−50 °C, suggestive of only moderate stability of the thiocarbenium ion under the reaction conditions (see the Supporting Information for the VT NMR traces).

As TfOH is poorly soluble in CD_2_Cl_2_ and freezes at temperatures below −40 °C, we reasoned that incomplete protonation of 2,3-dihydrothiopyran resulted in oligomerization. Accordingly, we doubled the amount of TfOH added and decreased the initial temperature to −88 °C to compensate for the inevitable temperature spike occurring during the brief removal of the tube from the cold probe for the addition of TfOH (Supporting Information). As a result, we were able to generate higher-quality spectra of thiocarbenium cation **35** in the absence of the quaternary ammonium salt (Scheme 5A,B). Our attempted generation and characterization of the corresponding oxocarbenium ion, previously generated electrochemically by Yoshida and co-workers and demonstrated to be stable until (∼−60 °C) in CD_2_Cl_2_,^66^ from dihydropyran under the same conditions was unsuccessful. Similarly, attempts to generate simple acyclic thiocarbenium ions by analogous low-temperature protonations of acyclic vinyl sulfides failed, presumably due to rapid self-condensation (see the Supporting Information).

**Figure 1:**
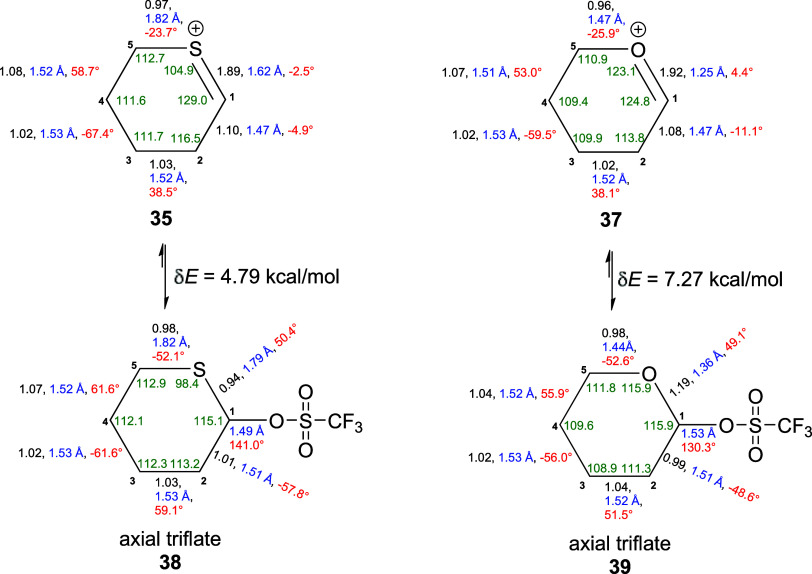
Computed natural bond orders (black), bond lengths (blue), ring torsional angles (red), and internal bond angles (green) for thiocarbenium ion **35** and oxocarbenium ion **37** and for axial covalent 2-thianyl triflate **38** and axial covalent tetrahydropyranosyl triflate **39**. An MP2/cc-pVDZ method was used to compute natural bond order, and an M06-2X/cc-pVDZ with cc-pVTZ on H, C, and S methods was used to compute bond lengths, ring torsion angles, and internal bond angles, and the difference of the electronic energies of the covalent triflates and the corresponding cations.

To provide further support for the structure of thiocarbenium ion **35**, we turned to computation. Density functional theory (DFT) calculations were carried out at the M06-2X level of theory for the gas phase with the cc-pVDZ basis set. The computed internal bond angles and ring torsional angles of the thiocarbenium ion **35** indicate considerably more puckering than in the oxocarbenium ion **37**. This is evidenced by (i) the significantly decreased C5–S–C1 bond angle (104.9°) of **35** relative to the C5–O–C1 bond angle (123.1°) in **37**, (ii) the increased S–C1–C2 bond angle in **35** (129.0°) compared to the O–C1–C2 bond angle in **37** (124.8°), and (iii) the significantly altered C5–S–C1–C2 and S–C1–C2–C3 ring torsion angles (−2.5, and −4.9°, respectively) of **35** with respect to the C5–O–C1–C2 and O–C1–C2–C3 ring torsion angles (4.4 and −11.1°, respectively) in **37**. Both cations have bond orders of *ca* 1.9 for the heteroatom-C1 bonds, suggesting a high degree of π-character in both cases. The sulfur-containing covalent triflate **38** also exhibits greater ring puckering than the oxygen-based analog **39** as evidenced by (i) a significantly decreased C5–S–C1 bond angle (98.4°) in **38** relative to the C5–O–C1 bond angle (115.9°) in **39** and (ii) the significantly increased S–C1–C2–C3, C1–C2–C3–C4, C2–C3–C4–C5 and C3–C4–C5–S ring torsion angles (−57.8, 59.1, –61.6 and 61.6°, respectively) in **38** compared to the corresponding O–C1–C2–C3, C1–C2–C3–C4, C2–C3–C4–C5 and C3–C4–C5–O torsion angles (−48.6, 51.5, –56.0 and 55.9°, respectively) in **39**. Importantly, the formation of the thiocarbenium ion **35** from the corresponding triflate **38** is approximately 2.5 kcal/mol less endothermic than that of the oxocarbenium ion **37** from the corresponding triflate **39** (Figure 1). Finally, the computed chemical shift at the BP86/6-311G+(2d,p) level with SMD using dichloromethane as solvent of δ 246 for the thienyl carbon in cation **35**, for a method estimated to have an error of ±8 ppm, is in good agreement with the experimental value of δ 238.3.

We next turned to the preparation of substrates for a comparative VT NMR study of the intermediates generated on activation of 5-thioglucopyranosyl and the corresponding glucopyranosyl donors with both arming ether and disarming ester-protecting groups.^67,68^ Among the multiple other types of donor that have been employed in VT NMR studies of glycosylation reactions,^69,70^ we initially selected the glycosyl sulfoxides as they are readily prepared and characterized, are shelf-stable, and are typically cleanly activated by simple addition of triflic anhydride at −78 °C, thereby minimizing experimental complications.^71,72^ Taking into account Hashimoto’s^73^ demonstration of the greater reactivity toward oxidation of the exocyclic sulfur compared to its endocyclic counterpart in ethyl 1,5-dithioglucopyranosides as compared to phenyl 1,5-dithioglucopyranosides, alkyl thioglucosides were selected as substrates for glycosyl sulfoxide generation. Complications in the activation of the 5-thioglucosyl sulfoxides subsequently led us to prepare and study the corresponding thioglucosyl and glucosyl trichloroacetoimidates.^74^ Here, we describe first the preparation and characterization of the full set of thioglucosyl and glucosyl donors before turning to the VT experiments and our findings.

**Scheme 6:**
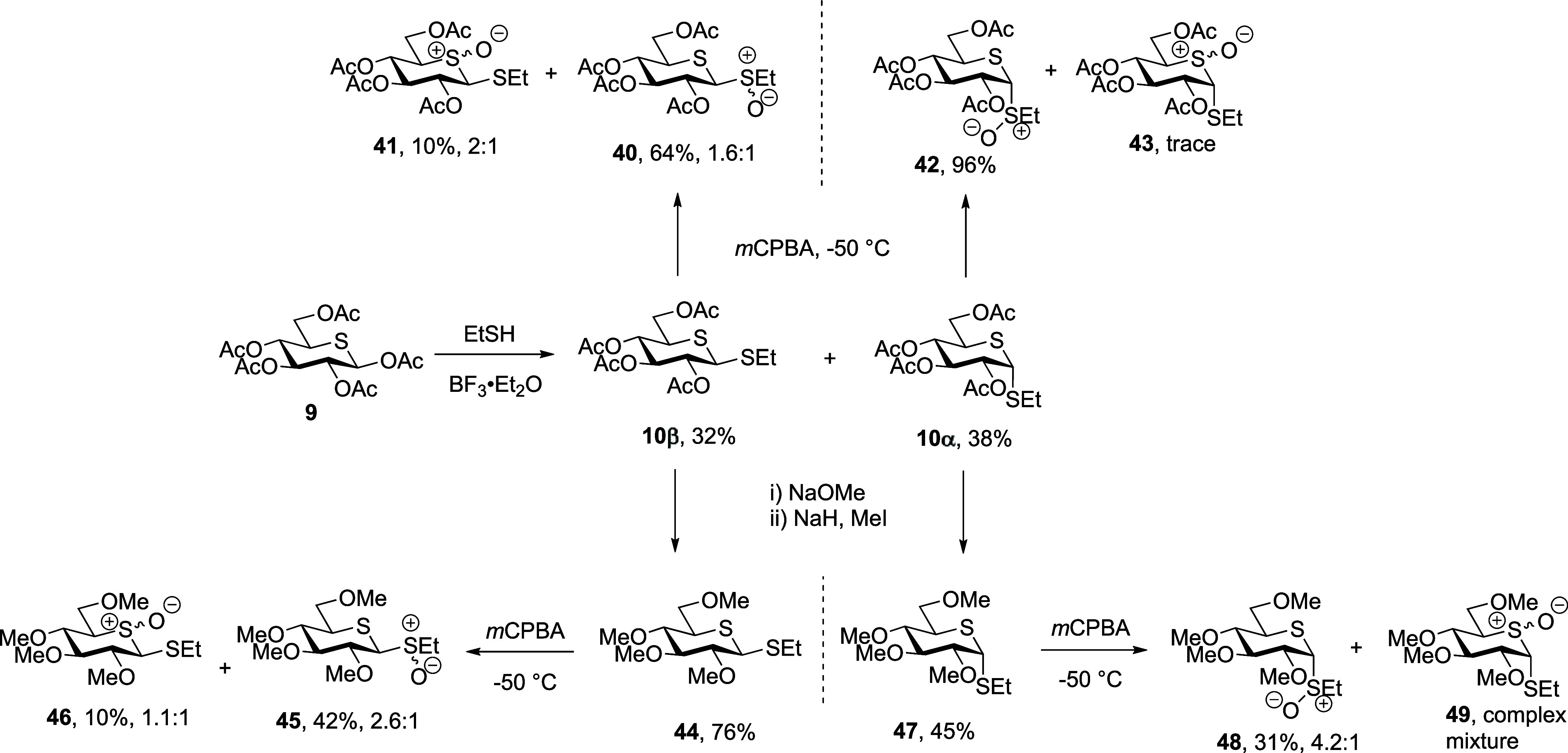
Synthesis of 5-Thioglycosyl Sulfoxides **40**, **42**, **45**, and **48**

Focusing first on the 5-thioglucosyl sulfoxides **40**, **42**, **45**, and **48** (Scheme 6), following the reported protocol and taking advantage of an unselective thioglycosylation reaction,^28^ both β-thioglycoside **10β** and α-thioglycoside **10α** were synthesized from 5-thioglucose pentaacetate **9**(31,75) in 32 and 38% yield, respectively. Oxidation of β-thioglycoside **10β** with *m*CPBA afforded a mixture of sulfoxides from which we were able to isolate diastereomeric 5-*S*-oxides **41** in 10% yield, as well as the desired 1-*S-*oxides **40** in 64% yield. The *m*CPBA oxidation of α-thioglycoside **10α** gave 1-*S-*oxide **42** as a single diastereomer in 96% yield accompanied by only a trace amount of 5-*S-*oxide **43**. The synthesis of the per-*O*-methyl sulfoxides **45** and **48** began with deacetylation of thioglycosides **10β** and **10α**, respectively, under Zemplen conditions. This was followed by methylation with MeI in the presence of NaH giving first the thioglycosides **44** and **47** in 76 and 45% yield, respectively, and finally exposure to *m*CPBA. Oxidation of **44** in this manner gave a mixture of sulfoxides, which were separated to afford the desired 1-*S-*oxides **45** in 42% yield and the corresponding 5-*S-*oxides **46** in 10% yield. Parallel processing of permethylated α-thioglycoside **47** gave desired 1-*S-*oxides **48** in 31% yield together with a complex mixture of 5-*S-*oxides **49** (Scheme 6).

**Figure 2:**
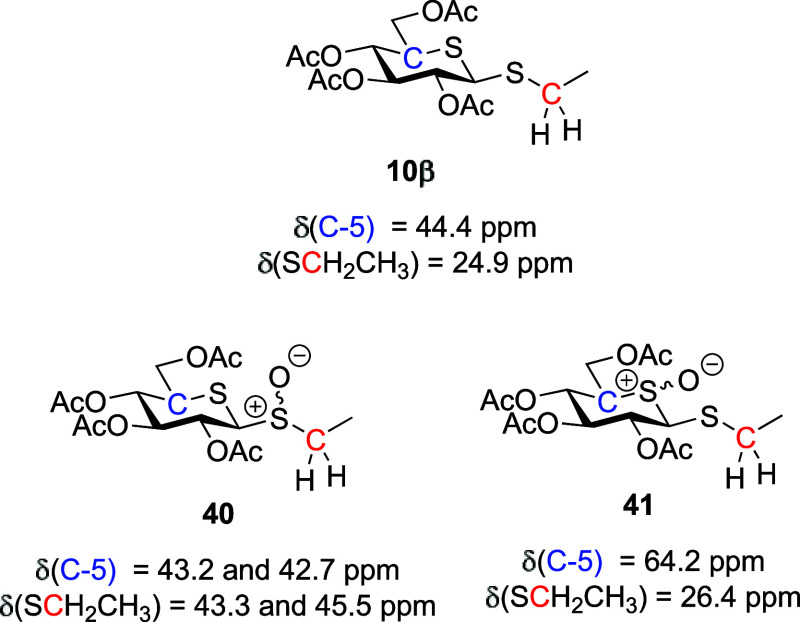
Diagnostic ^13^C NMR chemical shifts for 5-thioglucosyl ethyl thioglycosides and their *exo-* and *endo*-sulfoxides.

The regioselectivity of these *m*CPBA oxidations was determined by NMR spectroscopy and comparison with the literature spectral data.^73^ For example, in the ^1^H NMR spectrum of **40**, a characteristic geminal splitting of the methylene protons of the SCH_2_CH_3_ group was observed. Additionally, the ^13^C NMR spectrum revealed changes in the chemical shift of the SCH_2_CH_3_ methylene group carbon (δ_c_ = 43.3 and 42.7 ppm) in comparison to that of thiosugar starting material **10β** (δ_c_ = 24.9 ppm). In contrast, little to no change was observed in the chemical shift of C5 (δ_c_ = 43.2 and 45.1 ppm) in comparison to the starting material **10β** (δ_c_ = 44.4 ppm) (Figure 2). On the other hand, no geminal splitting of the SCH_2_CH_3_ methylene protons was observed in the ^1^H NMR spectrum of the endocyclic sulfoxide **41**, and the chemical shift of the SCH_2_CH_3_ methylene carbon (δ_c_ = 26.4 ppm) of **41** was little changed from **10β** (δ_c_ = 24.9 ppm). In contrast, a significant change was noted in the chemical shift of C5 in endocyclic sulfoxide **41** (δ_c_ = 64.2 ppm) with respect to starting material **10β** (δ_c_ = 44.4 ppm) (Figure 2). Parallel analyses were conducted for all *exo*-sulfoxides **42**, **45**, and **48** prepared (see the Supporting Information).

**Scheme 7:**
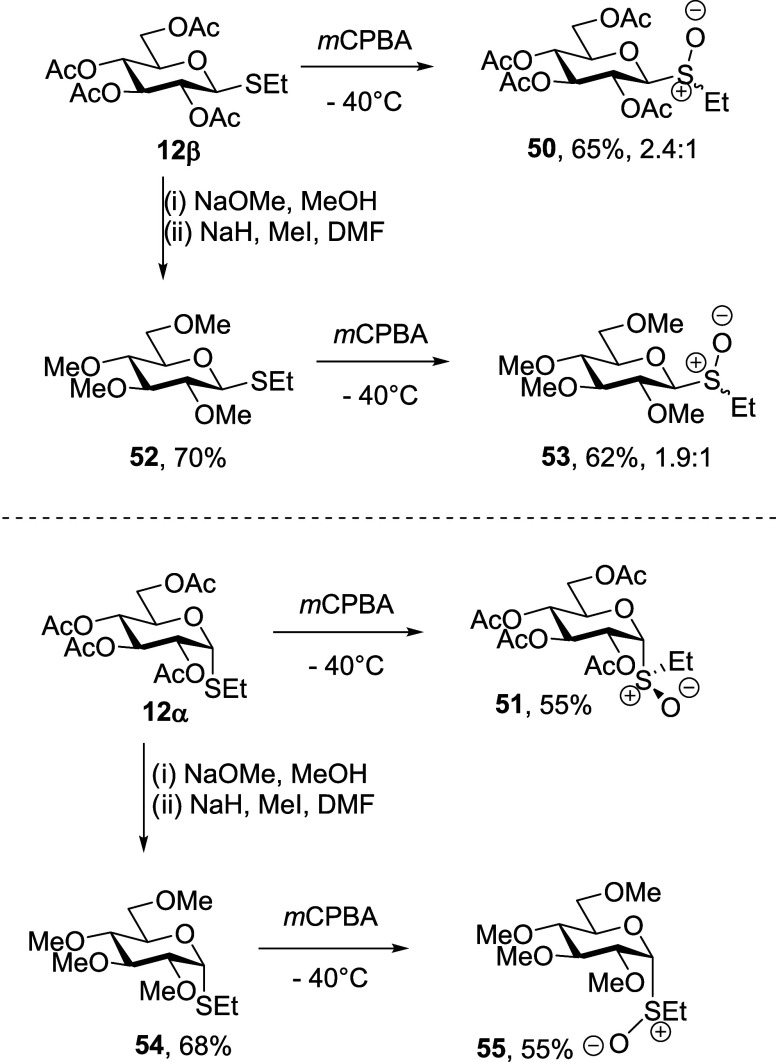
Synthesis of the Glucosyl Sulfoxides **50**, **51**, **53**, and **55**

The synthesis of the glucosyl sulfoxide comparators began with the commercially available per-*O*-acetyl α- and β-thioglucosides **12α** and **12β** (Scheme 7). *m*CPBA oxidation of the α-thioglycoside **12α** gave glucosyl sulfoxide **51** as a single diastereomer in 55% yield that was assigned the *R*_*S*_ configuration by analogy with the precedent.^76^ That β-thioglycoside **12β** afforded sulfoxides **50** as a mixture of diastereomers in 65% yield. For the synthesis of the per-*O*-methylated sulfoxides **53** and **55**, following the reported protocol,^77^ β-thioglycoside **12β** was deacetylated with NaOMe and subsequently alkylated with methyl iodide in the presence of sodium hydride to afford **52** in 70% yield. The *m*CPBA oxidation of **52** gave per-*O*-methylated β-glucosyl sulfoxide **53** as a mixture of diastereoisomers in 62% yield. The α-thioglycoside **12α** was similarly converted into the per-*O*-methylated thioglycoside **54** in 68% yield and then oxidized with *m*CPBA to give the armed α-sulfoxide **55** in 55% yield as a single diastereomer.

**Scheme 8:**
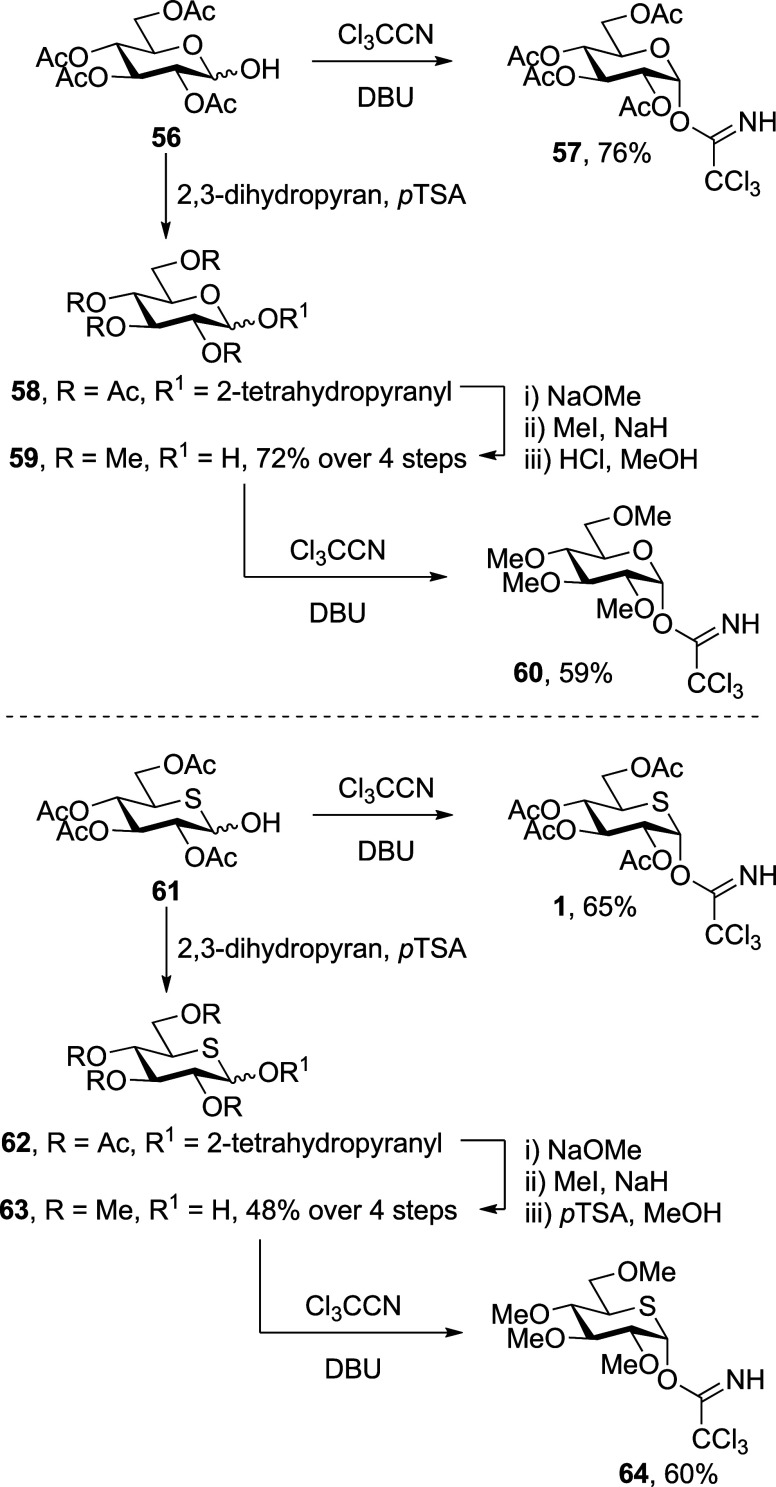
Synthesis of Glycosyl Trichloroacetoimidates **57**, **60**, **1**, and **64**

Preparation of the trichloroacetoimidate-based donors (Scheme 8), began with the commercially available hemiacetal **56** and 5-thioglucose hemiacetal **61**, itself prepared from 5-thioglucose pentaacetate **9** by a reported protocol.^27^ Hemiacetal **56** was coupled with trichloroacetonitrile in the presence of catalytic DBU in the standard manner to give the per-*O*-acetyl imidate **57** in 76% yield. To access the corresponding per-*O*-methylated imidate **60**, **56** was coupled with 2,3-dihydropyran in the presence of *p*TSA to give the tetrahydropyranyl glycoside **58**. This was followed by deacetylation with NaOMe, methylation with methyl iodide in the presence of sodium hydride, and hydrolysis with methanolic HCl to afford the per-*O*-methylated hemiacetal **59** in 72% yield over 4 steps. Finally, coupling of this hemiacetal with trichloroacetonitrile in the presence of catalytic DBU afforded per-*O*-methylated imidate **60** in 59% yield. The per-*O*-acetyl 5-thioglucosyl imidate **1** (Scheme 8) was obtained in 65% yield by coupling hemiacetal **61** with trichloroacetonitrile in the presence of DBU as previously described.^29^ For the synthesis of the per-*O*-methylated analog **64**, the hemiacetal **61** was protected as the tetrahydropyranyl glycosides **62**,^29^ which were sequentially subjected to deacetylation, methylation, and hydrolysis with *p*TSA in methanol to produce the per-*O*-methylated hemiacetal **63** in 48% yield. Coupling of **63** with trichloroacetonitrile in the presence of catalytic DBU then afforded the target per-*O*-methylated trichloroacetoimidate **64** in 60% yield.

### Variable Temperature NMR Studies

**Figure 3:**
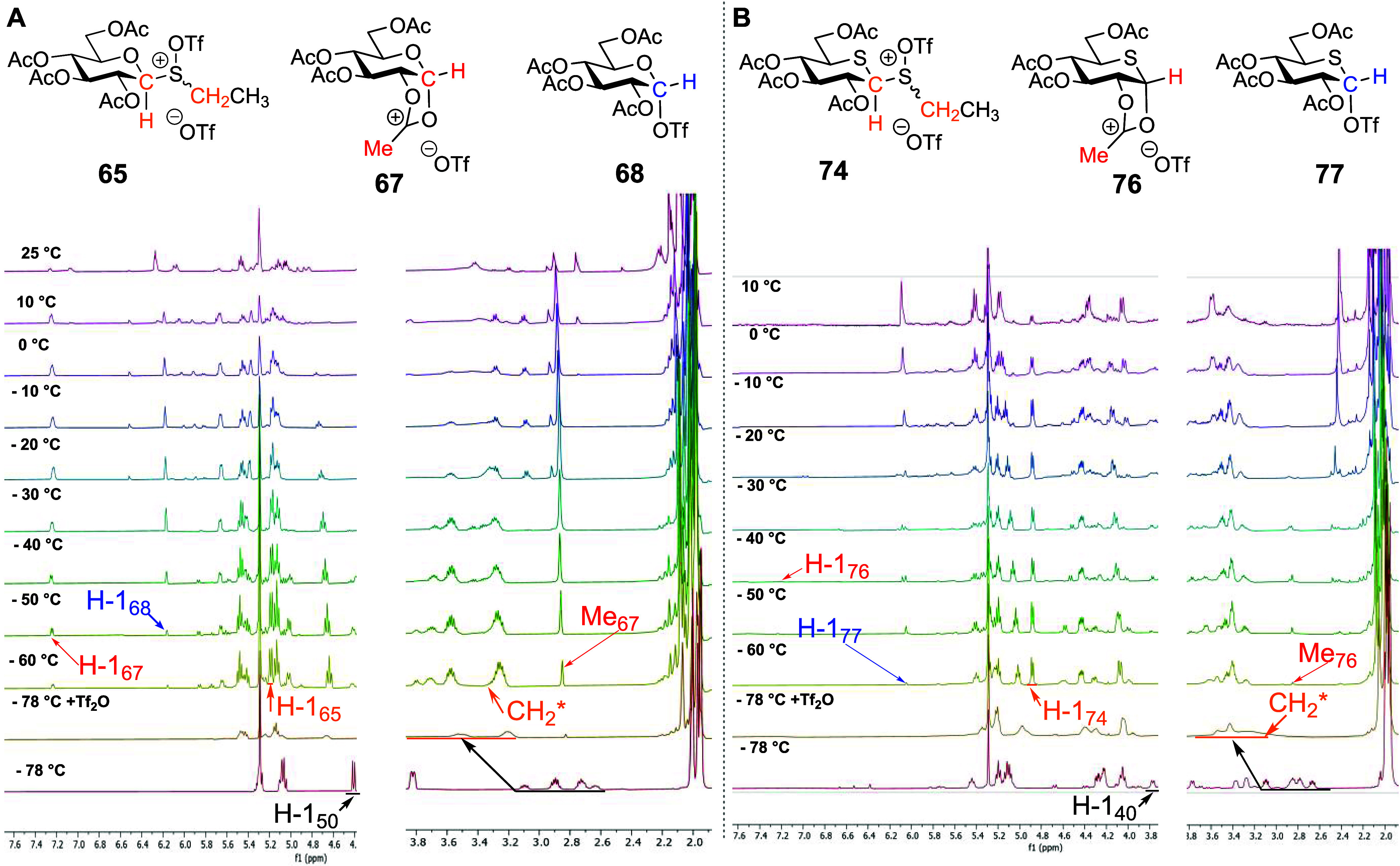
(A) Structures of reactive intermediates **65**, **67**, and **68**, and partial stacked ^1^H NMR spectra from VT experiments with glucosyl sulfoxide **50**. (B) Structures of reactive intermediates **74**, **76**, and **77**, and partial stacked ^1^H NMR spectra from VT experiments with 5-thioglycosyl sulfoxide **40**. Analogous spectra were observed when working with the anomeric donors **51** and **42**, respectively (Supporting Information).

**Figure 4:**
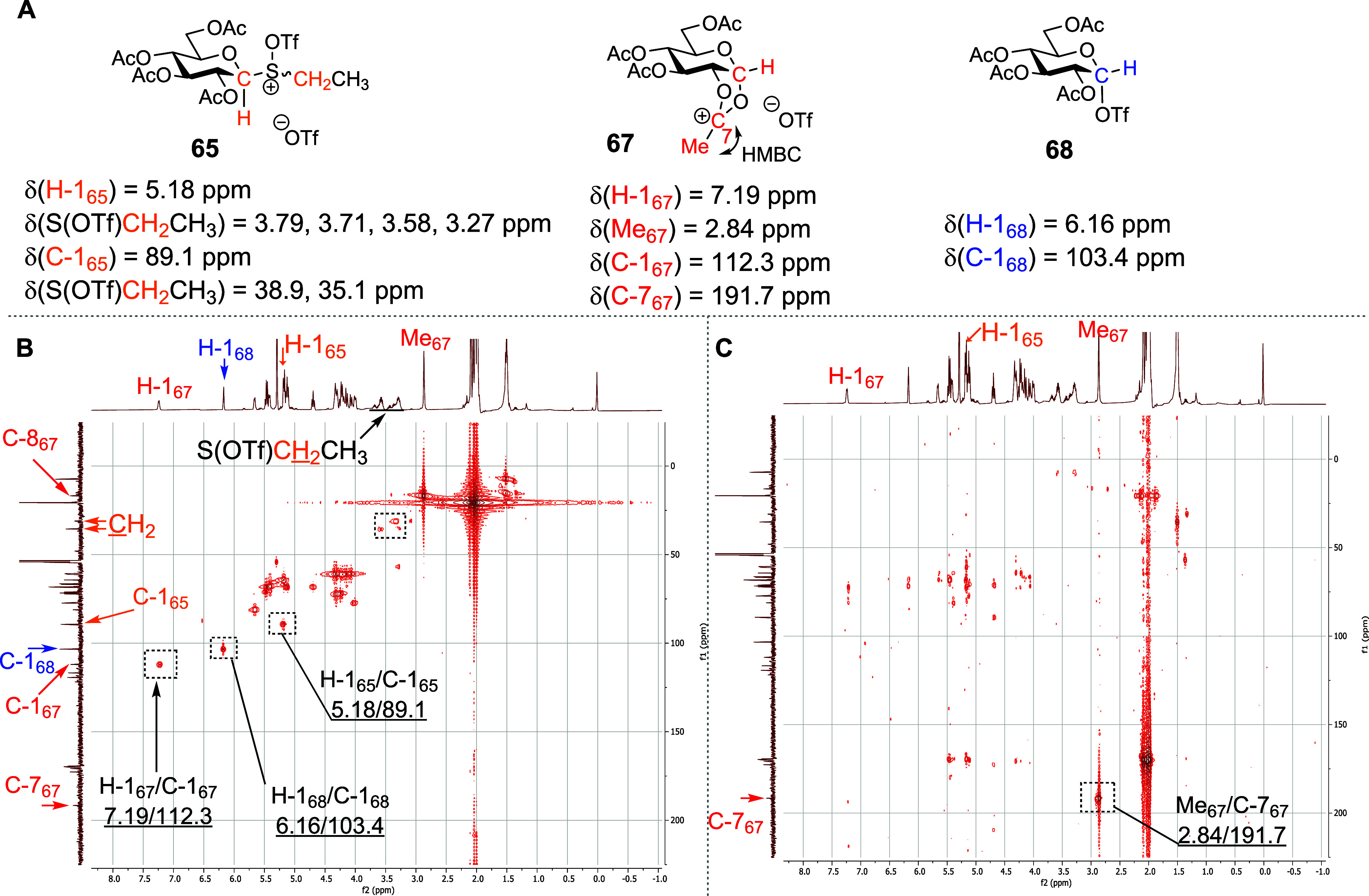
(A) Structures of reactive intermediates **65**, **67**, and **68**, identified during VT NMR experiments with sulfoxide **50**. (B) Partial HMQC spectrum at −30 °C. (C) Partial HMBC spectrum at −30 °C. An analogous picture was observed when running VT NMR experiments with the *a*-isomer **51** (see the Supporting Information).

Beginning with the two anomers **50** and **51** of the peracetylated glucosyl sulfoxides, activation with triflic anhydride at −78 °C in CD_2_Cl_2_ led to the generation of mixtures comprised mainly of the “activated sulfoxide” species **65** and **66**, respectively, and lesser amounts of the dioxalenium ion **67** and the α-glycosyl triflate **68** (Figure 3A and Supporting Information). The “activated sulfoxide” species **65** and **66** were characterized by downfield chemical shifts of the SCH_2_CH_3_ methylene and anomeric protons H-1 (e.g., δ_H1_ = 5.18 and 4.41 ppm for **65** and **50**, respectively), and the connection between the S(OTf)CH_2_CH_3_ chain and the pyranose ring was established by HMQC/HMBC correlations (Figure 4). The dioxalenium ion **67** was characterized by two diagnostic ^1^H NMR signals: (i) a downfield anomeric signal at δ_H_ = 7.19 ppm and (ii) a singlet at δ_H_ = 2.84 ppm, corresponding to the dioxalenium methyl group. Additionally, in the ^13^C NMR spectra of dioxalenium ion **67**, a downfield signal at δ_C_ = 191.7 ppm, corresponding to the positively charged C7, and a downfield-shifted anomeric signal at δ_C_ = 112.3 ppm were observed. The signal at δ_C_ = 191.7 ppm showed a weak HMBC correlation to the anomeric proton at δ_H_ = 7.19 ppm and a strong correlation to the dioxalenium methyl group at δ_H_ = 2.84 ppm, all fully consistent with the assigned structure **67** (Figure 4) and with literature data.^78^ Triflate **68** was characterized by a ^1^H NMR resonance at δ_H_ = 6.16 ppm and by a ^13^C NMR resonance at δ_C_ = 103.4 ppm corresponding to its anomeric H-1 and C1 atoms, respectively (Figure 3A), again consistent with the literature.^78^ On gradual warming, the activated sulfoxides **65** and **66** were converted to the dioxalenium ion **67** and the triflate **68**, with the transformation being essentially complete by ∼−20 °C for the β-anomer **65** and ∼−10 °C for α-the anomer **66**. The concentration of triflate **68** reached a maximum between −20 and −10 °C, above which it rapidly fell off, while the signals due to the dioxalenium ion **67** were maximal between −20 and 0 °C (Figure 3A) but persisted even at +25 °C.

**Scheme 9:**
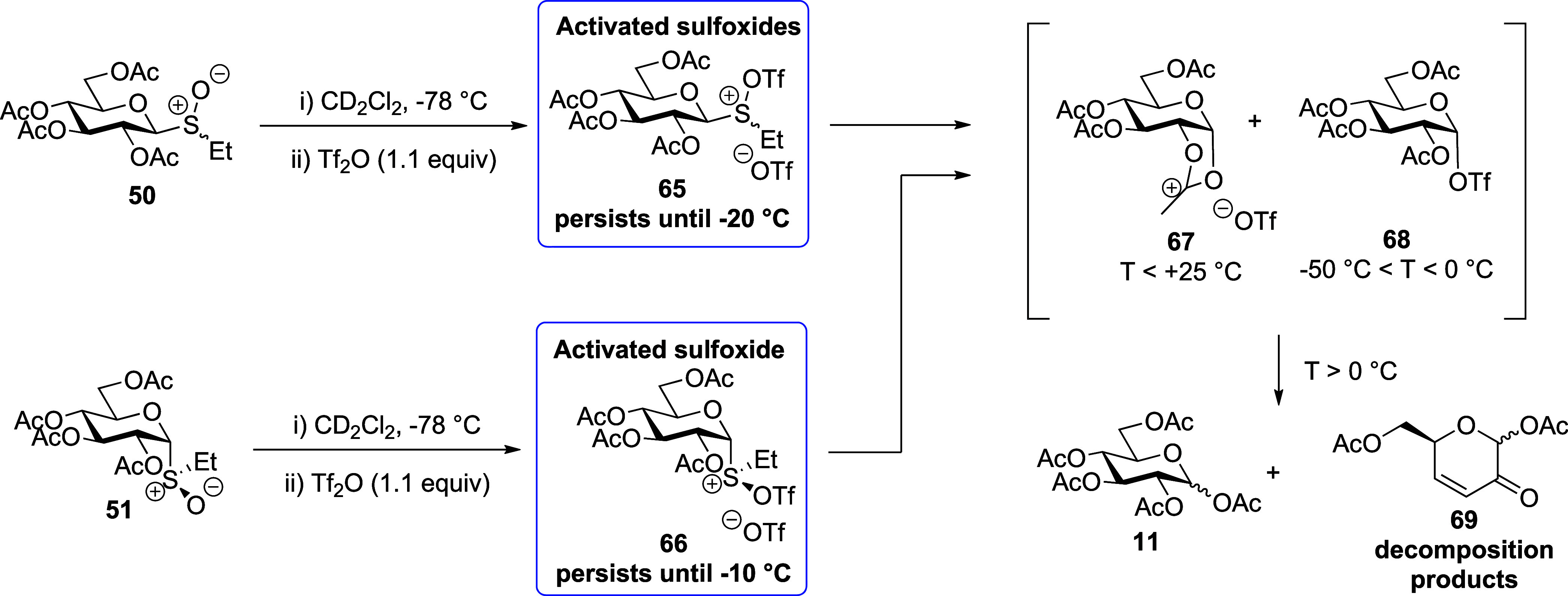
Intermediates and Decomposition Products Generated during VT NMR Experiments with Glucosyl Sulfoxides **50** and **51**

After termination of the VT NMR experiments with **50** and **51**, the byproducts glucose pentaacetate **11** and the enone **69** were isolated from the reaction mixtures, both with spectral data in agreement with the literature (Scheme 9).^79,80^

**Scheme 10:**
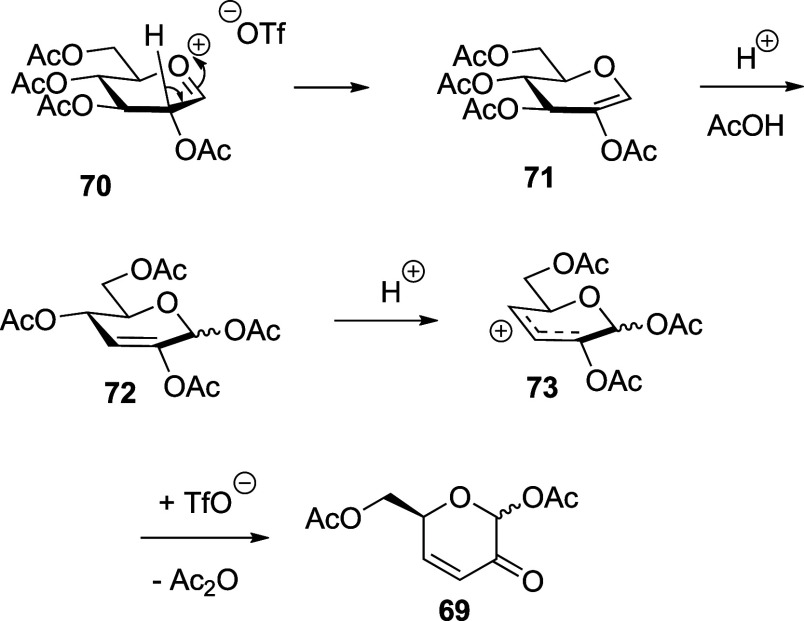
Proposed Mechanism for the Formation of **69** from Oxocarbenium Ion **70**

The enone **69** was previously reported by Metzger^80^ who obtained it by thermal decomposition of α,β-glucopyranose pentaacetate and proposed a mechanism for its formation involving a series of eliminations and 3,3-sigmatropic rearrangements. However, as during the VT NMR experiments TfOH is present in the reaction mixture, we propose a mechanism based on a series of acid-catalyzed rearrangements and eliminations beginning with the transient oxocarbenium ion accessible from any one of the activated sulfoxides, the dioxalenium ion **67** or the glycosyl triflate **68** (Scheme 10).

**Figure 5:**
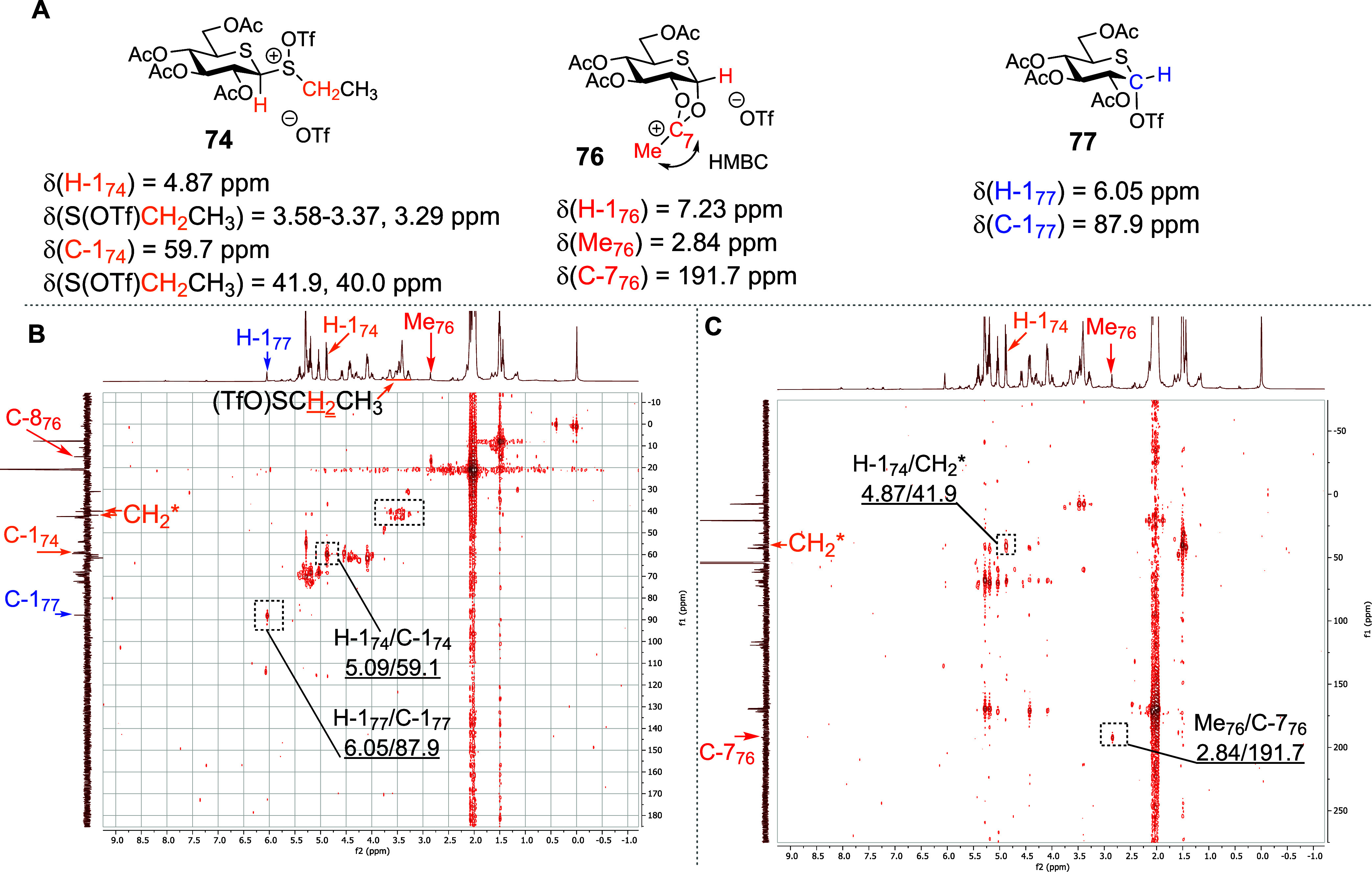
(A) Structures of reactive intermediates **74**, **76**, and **77**, identified during VT NMR experiments with sulfoxide **40**. (B) Partial HMQC spectrum at −50 °C plotted with DEPT-135 as the *Y*-axis. (C) Partial HMBC spectrum at −50 °C. The analogous pattern was observed when running VT NMR experiments with the *a*-isomer **42** (see the Supporting Information).

Turning to the per-*O*-acetyl 5-thioglycosyl sulfoxides **40** and **42**, activation with triflic anhydride in CD_2_Cl_2_ at −78 °C leads to the generation of the “activated sulfoxide” species **74** and **75**. Similarly to the parent glucosides, these were characterized by downfield shifts of the SCH_2_CH_3_ methylene protons and of the anomeric H-1 protons (e.g., δ_H_ = 4.88 and 3.77 ppm corresponding to the anomeric protons H-1 of **74** and **40**, respectively). The connection between the S(OTf)CH_2_CH_3_ moiety and the carbohydrate was confirmed by HMQC/HMBC NMR spectroscopy (Figure 5 and Supporting Information). However, in contrast to **50** and **51**, much smaller quantities of expected dioxalenium ion **76** and thioglycosyl triflate **77** were formed from either **74** or **75** at −60 °C (Figure 3B). Similarly to **67**, the dioxalenium ion **76** was characterized by HMBC-correlated signals at δ_H_ = 2.84 ppm and δ_C_ = 191.7 ppm, for the dioxalenium methyl group and the positively charged carbon atom, and by an anomeric signal at δ_H_ = 7.19 ppm (Figure 5). Triflate **77** was characterized by ^1^H and ^13^C NMR resonances at δ_H_ = 6.05 ppm and δ_C_ = 87.9 ppm corresponding to its anomeric proton and carbon atoms. Further support for covalent triflate generation was found in the ^19^F NMR spectrum, wherein a characteristic peak at δ_F_ = −75.36 ppm was observed. On warming, the amount of dioxalenium ion **76**, present in the reaction mixture increased gradually reaching a maximum at −40 °C, above which decomposition set in and was complete by −10 °C (Figure 3B). The concentration of triflate **77** present in the reaction mixture also increased with temperature, reaching a maximum around at −40 °C, but was fully dissipated by −10 °C. Pertinently, no signals were detected in either the ^1^H or ^13^C NMR spectra of the activated per-*O*-acetyl 5-thioglucosides **40** and **42**, which could be assigned to a thiocarbenium ion intermediate at any temperature studied.

**Scheme 11:**
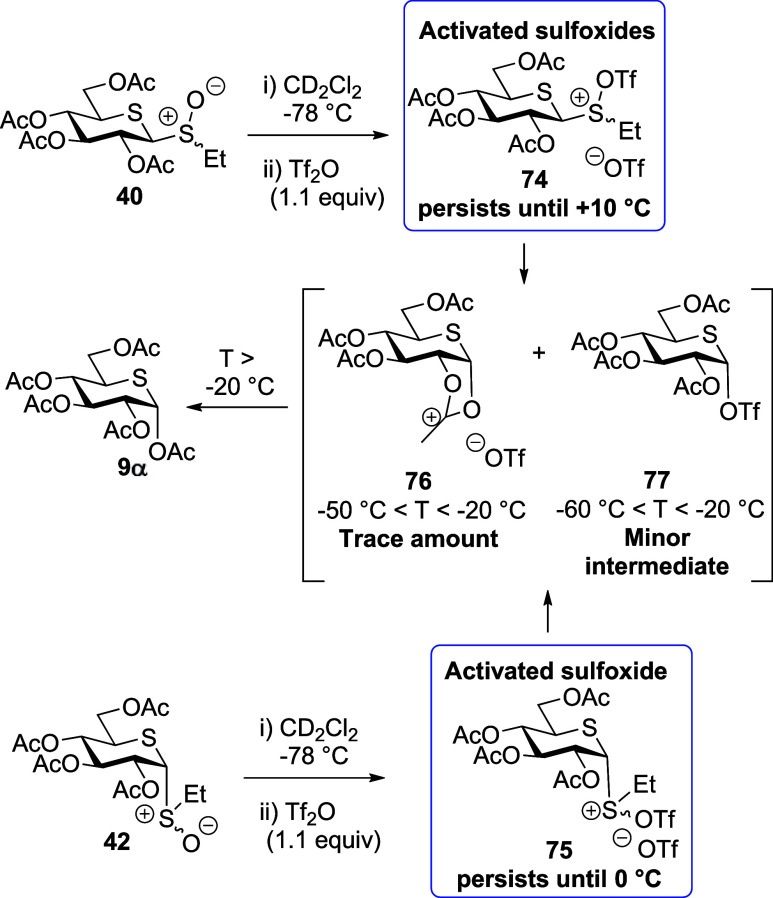
Intermediates and the Decomposition Product Generated during VT NMR Experiments with 5-Thioglycosyl Sulfoxides **40** and **42**

In contrast to the relatively clean VT NMR mixture arising from the peracetylated glucosyl sulfoxides **50** and **51**, by the time the NMR probe reached room temperature and the sample was retrieved from the probe, the reaction mixture from the activation of either peracetyl 5-thioglucosyl sulfoxide was a black tar, from which only the pentaacetate **9α** could be isolated (Scheme 11). Presumably, decomposition of the 5-thioglucose-derived dioxalenium ion **76** and the triflate **77** follows an analogous path to that presented for the formation of enone **69** (Scheme 10) from the corresponding glucosyl derivatives, but one or more of the sulfur-based intermediates is unstable and undergoes further decomposition

**Figure 6:**
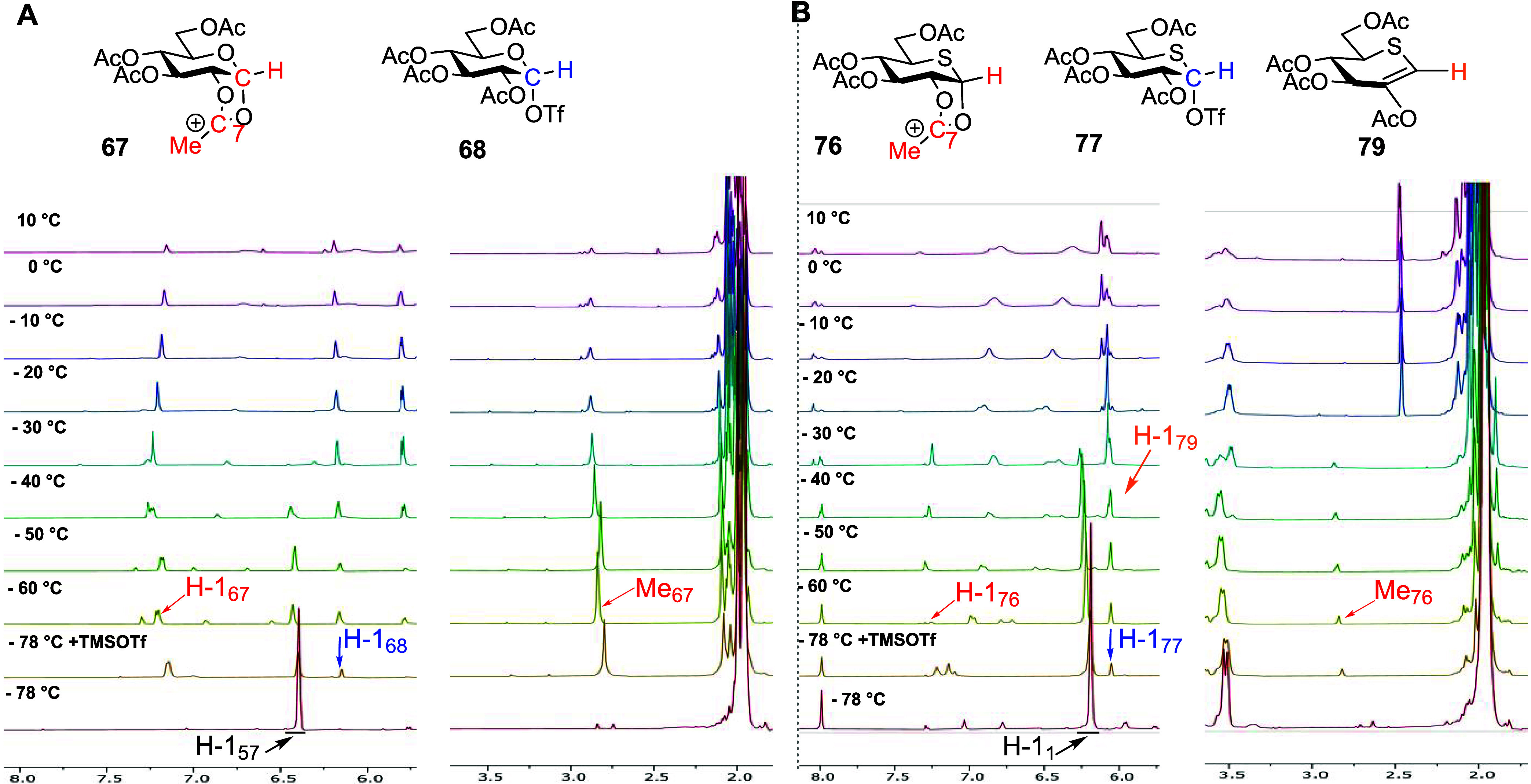
(A) Structures of reactive intermediates **67** and **68** and partial stacked ^1^H NMR spectra from VT experiments with glucosyl trichloroacetoimidate **57**. (B) Structures of reactive intermediates **76**, **77**, and **79** and partial stacked ^1^H NMR spectra from VT experiments with 5-thioglucosyl trichloroacetoimidate **1**.

**Scheme 12:**
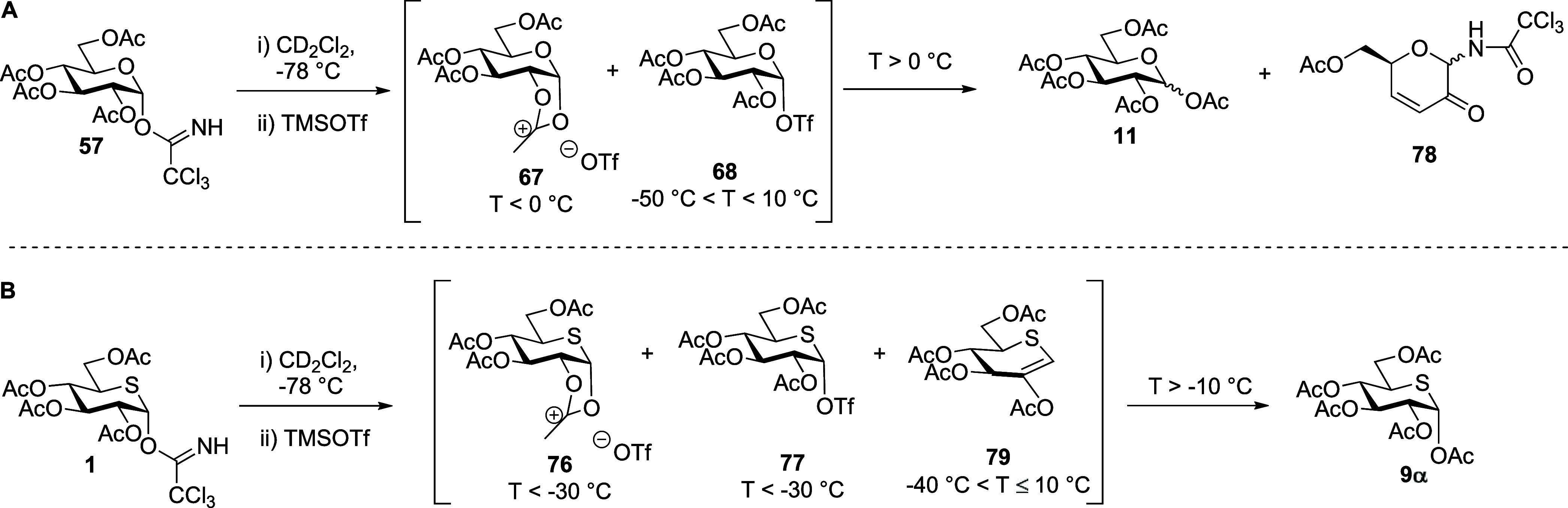
Intermediates and Decomposition Products Generated during VT NMR Experiments with (A) Glucosyl Trichloroacetoimidate **57** and (B) Thioglucosyl Trichloroacetoimidate **1**

The unexpected complexities observed in the activation of the various peracetylated sulfoxides, especially the persistence of the initially activated sulfoxides at low temperatures, resulting in their slow conversion to the anticipated (thio)glucosyl dioxalenium ions and triflates, prompted the synthesis and investigation of the corresponding (thio)glucosyl trichloroacetimidates. Activation of these donors was performed by the addition of TMSOTf at −78 °C in CD_2_Cl_2_ in an NMR tube, followed by rapid return of the tube to the precooled probe and recording of spectra, and then incremental warming with spectra recorded at each interval. Beginning with the per-*O*-acetyl glycosyl trichloroacetoimidate **57** (Figure 6A), minor amounts of both the dioxalenium ion **67** and the glycosyl triflate **68** were observed at −78 °C. The latter became increasingly more prominent from −60 °C upward. Nevertheless, conversion was slow at the lower temperatures, and the imidate was not fully consumed until approximately −30 °C, a temperature at which both the dioxalenium ion **67** and the triflate **68** had mostly been converted to glucose pentaacetate and a second product that remained stable until room temperature. This product was characterized following isolation as the enonyl amide derivative **78**, which presumably arises by a pathway analogous to that outlined for the formation of enonyl acetate **69** (Scheme 10) but with the intervention of the silylated trichloroacetamide derivative, formed during the activation process, as a nucleophile. Turning to the per-*O*-acetyl 5-thioglycosyl trichloroacetoimidate **1** (Figure 6B), immediately after the addition of TMSOTf at −78 °C, the formation of dioxalenium ion **76** and considerably more covalent triflate **77** was observed. However, as with the glucosyl imidate **57**, trichloroacetoimidate **1** was not fully consumed on the initial addition of TMSOTf, with complete consumption of the starting material only seen at temperatures >−30 °C. Both the dioxalenium ion **76** and covalent triflate **77** slowly accumulated in the reaction mixture on warming, with the dioxalenium ion reaching a maximum concentration −60 °C < *T* < −50 °C and the triflate at −50 °C, above which temperatures decomposition slowly set in. Beginning around −40 °C, a further product, identified as the thioglycal **79** by the olefinic signals at δ_H_ = 6.07 ppm and δ_C_ = 113.5 ppm (see the Supporting Information) consistent with literature data,^30^ was observed; this product was essentially absent from the reaction mixture above −30 °C. Similarly to the sulfoxides **40** and **42**, only pentaacetate **9α** was isolated from the reaction mixture after termination of the VT NMR experiment (see the Supporting Information). The overall results of the VT NMR experiments with both peracetylated trichloroacetimidates are summarized in Scheme 12. Again, notably, although cleaner and less complex than the sulfoxide-based studies, no indication of the formation of a thiocarbenium ion was found in the course of these experiments.

**Figure 7:**
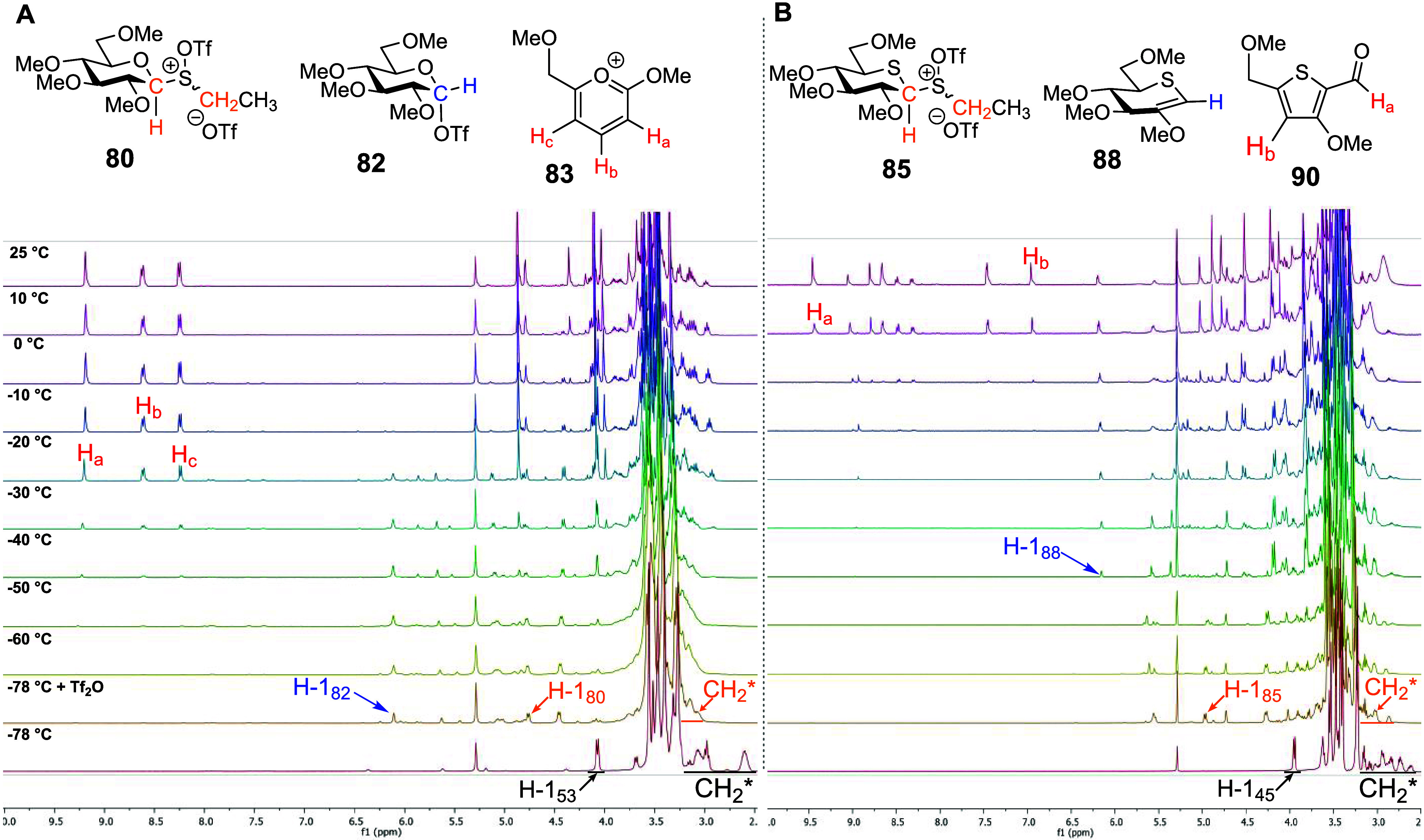
(A) Structures of reactive intermediates **80**, **82**, and byproduct **83** and partial stacked ^1^H NMR spectra from VT experiments with permethylated glucosyl sulfoxide **53**. (B) Structures of reactive intermediates **85**, and byproducts **88** and **90** and partial stacked ^1^H NMR spectra from VT experiments with permethylated 5-thioglucosyl sulfoxide **45**. Analogous spectra were observed when working with the anomeric donors **55** and **48**, respectively (Supporting Information).

**Figure 8:**
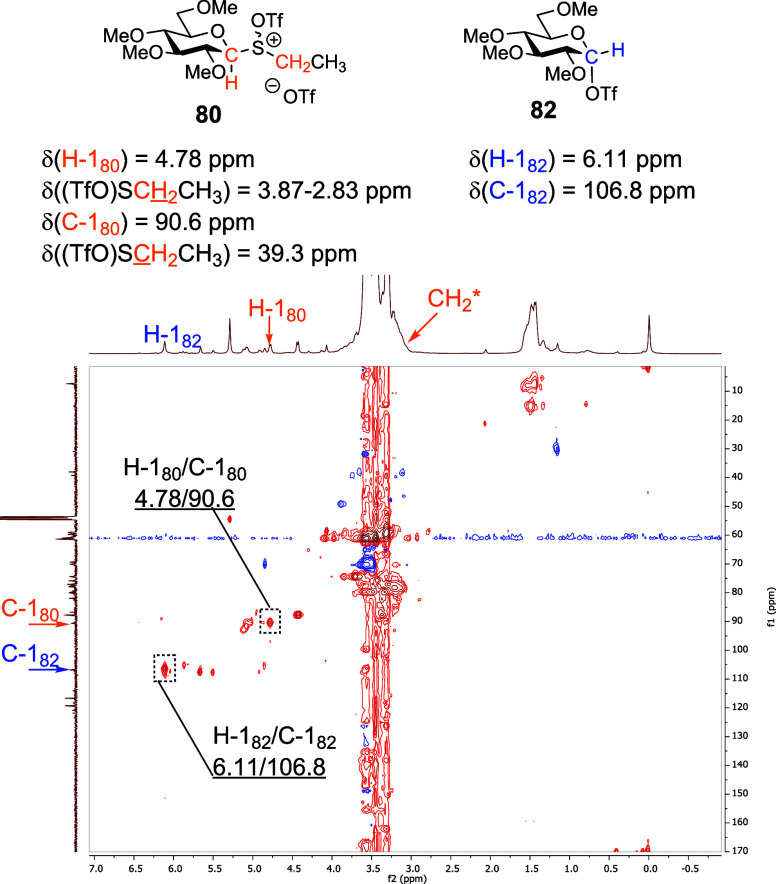
Structures of reactive intermediates **80** and **82** identified during VT NMR experiments with **53**; partial HSQC spectrum at −50 °C with key correlations.

**Scheme 13:**
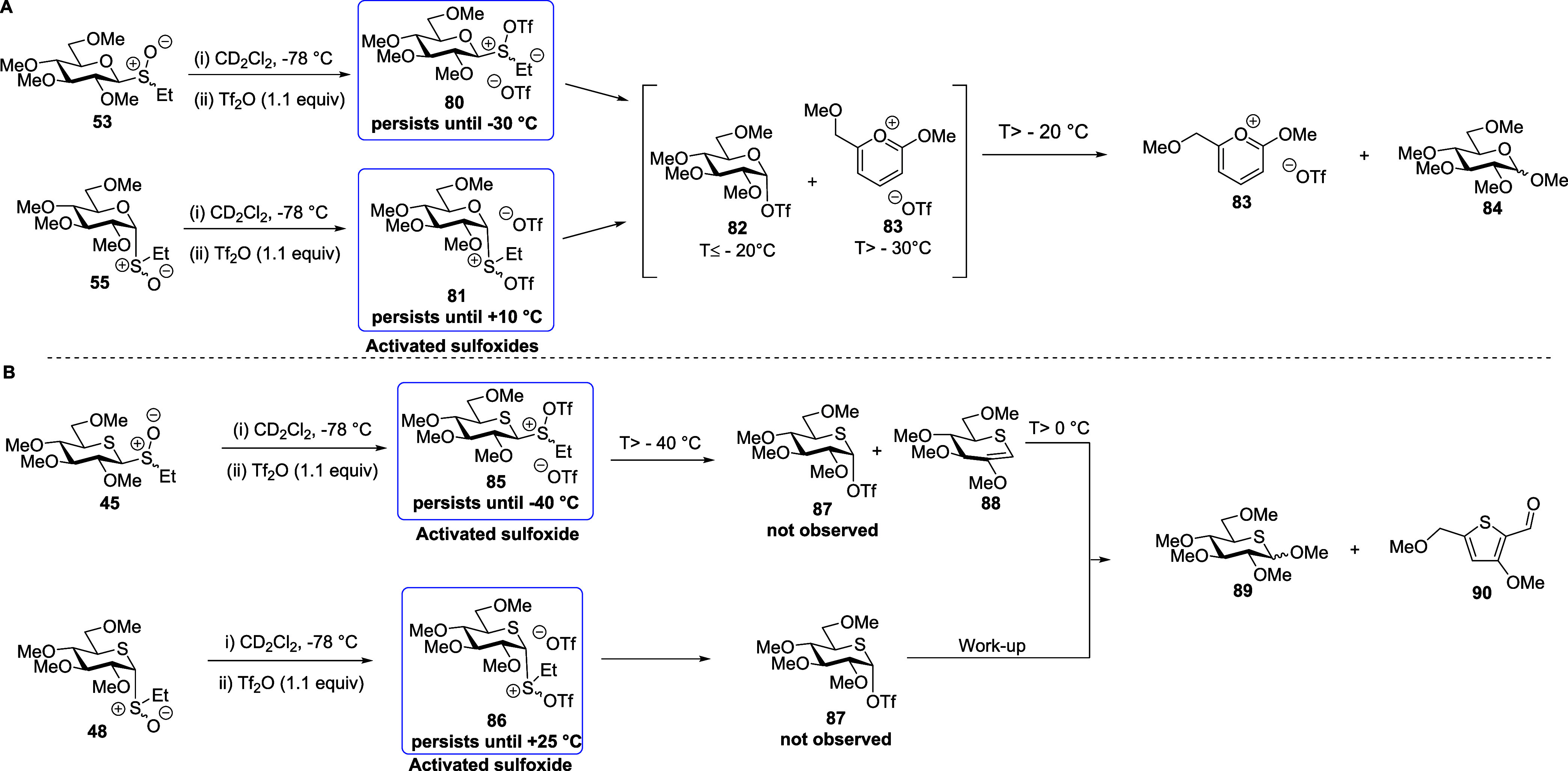
Intermediates and Products Generated during VT NMR Experiments with (A) per-*O*-Methylated Glycosyl Sulfoxide **53** and **50** and (B) Per-*O*-methylated 5-Thioglucose Sulfoxides **45** and **48**

With regard to the armed per-*O*-methylated donors, first, we performed VT NMR experiments with the glucosyl sulfoxides **53** and **55** (Figure 7A, and Supporting Information). As with the per-*O*-acetylated sulfoxides **50** and **51**, the “activated sulfoxides” **80** and **81** were formed immediately on the addition of Tf_2_O at −78 °C in CD_2_Cl_2_ and were characterized by downfield shifts of the SCH_2_CH_3_ methylene protons (δ_H_ = 3.87–2.83 and 2.60 ppm in **80** and **81**, respectively) and of the anomeric proton signals. Notably, activated sulfoxide **81**, unlike **80**, persisted on warming until +10 °C (see the Supporting Information). A signal corresponding to the formation of the covalent triflate **82**, namely a downfield anomeric signal at δ_H_ = 6.11 ppm of HSQC-correlated with an anomeric signal at δ_C_ = 106.3 ppm, was observed immediately after addition of Tf_2_O (Figure 8). The covalent triflate **82** accumulated gradually with increasing temperature reaching a maximum at −40 °C above which decomposition set in and was complete by −20 °C. At temperatures *T* > −30 °C, a new species assigned as the pyrylium ion **83** was observed and accumulated rapidly with further increases in temperature (see the Supporting Information). After termination of this experiment, both **83** and pentamethyl glucose **84** were detected in the reaction mixture by mass spectrometry; however, only pentamethyl glucose **84** could be isolated owing to the instability of **83** toward hydrolysis (Scheme 13A).

Switching to the per-*O*-methyl 5-thioglycosyl sulfoxides **45** and **48** (Figure 7B, Scheme 13B and Supporting Information), “activated sulfoxides” **85** and **86** were similarly formed immediately on addition of Tf_2_O at −78 °C. However, no signals corresponding to the anticipated covalent triflate **87** were observed at this temperature. In the case of the β-glycosyl sulfoxide **45**, on warming the “activated sulfoxide” **85** persisted until −40 °C when decomposition set in with the formation of the thioglycal **88** and penta-*O*-methyl 5-thioglucose **89**. Above *T* = 0 °C, glycal **88** and residual activated sulfoxides **85** underwent further decomposition, leading to the thiophene derivative **90**. In contrast, the α-anomer **86**, similar to the parent-activated sulfoxide **75**, was found to be stable until ∼+25 °C (see the Supporting Information). After the termination of this experiment, both **89** and **90** were detected in the reaction mixture by mass spectrometry, but only thiophene **90**, admixed with trace amounts of **89**, was isolated from the reaction mixture. No indication of the formation of a thiocarbenium ion was found at any temperature on activation of either of the per-*O*-methyl 5-thioglycosyl sulfoxides **45** and **48**.

**Figure 9:**
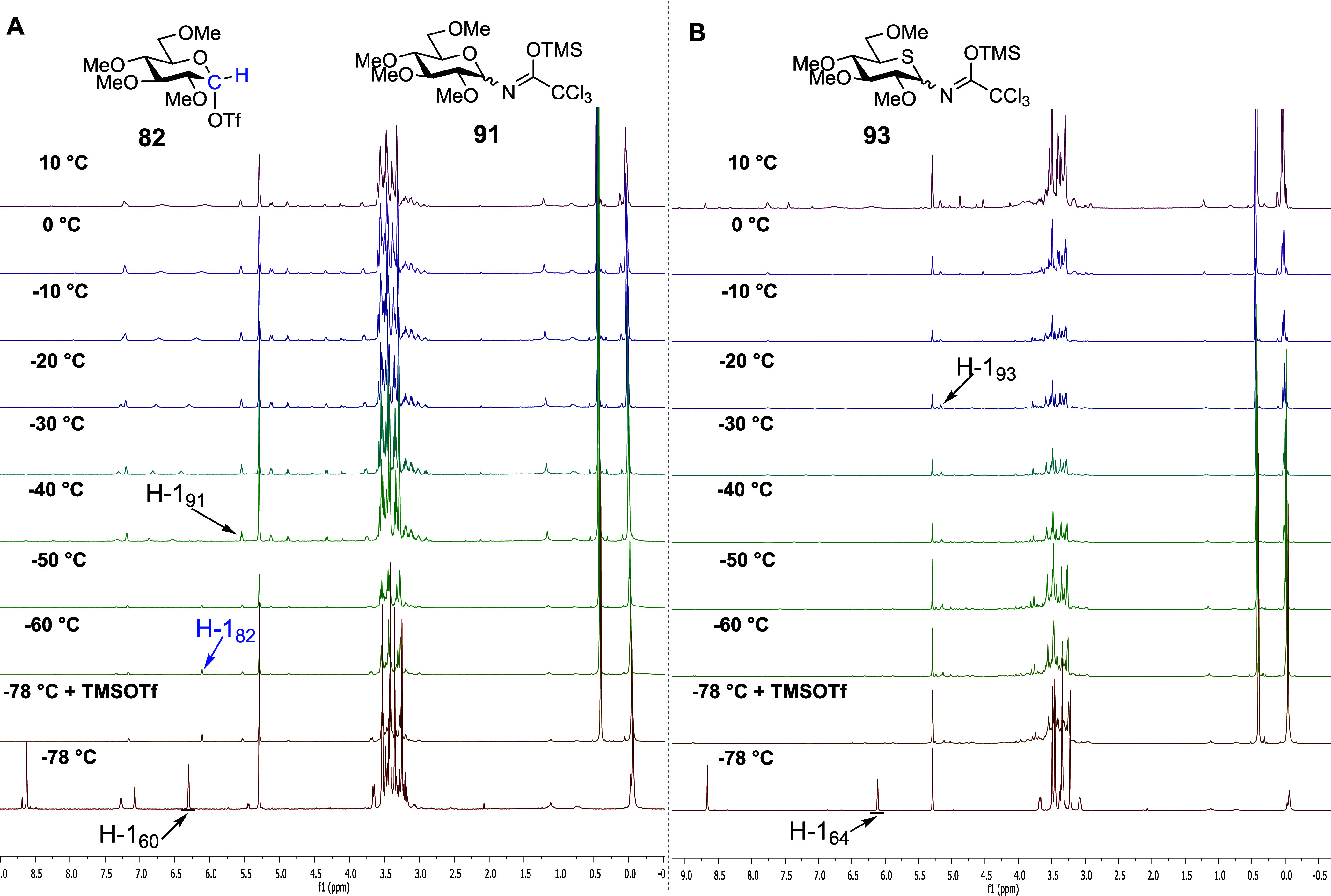
(A) Structures of reactive intermediates **82**, and byproduct **91** and partial of stacked ^1^H NMR spectra from VT experiments with permethylated glucosyl trichloroacetimidate **60**. (B) Partial stacked ^1^H NMR spectra from VT experiments with permethylated 5-thioglucosyl trichloroacetimidate **64**.

**Scheme 14:**
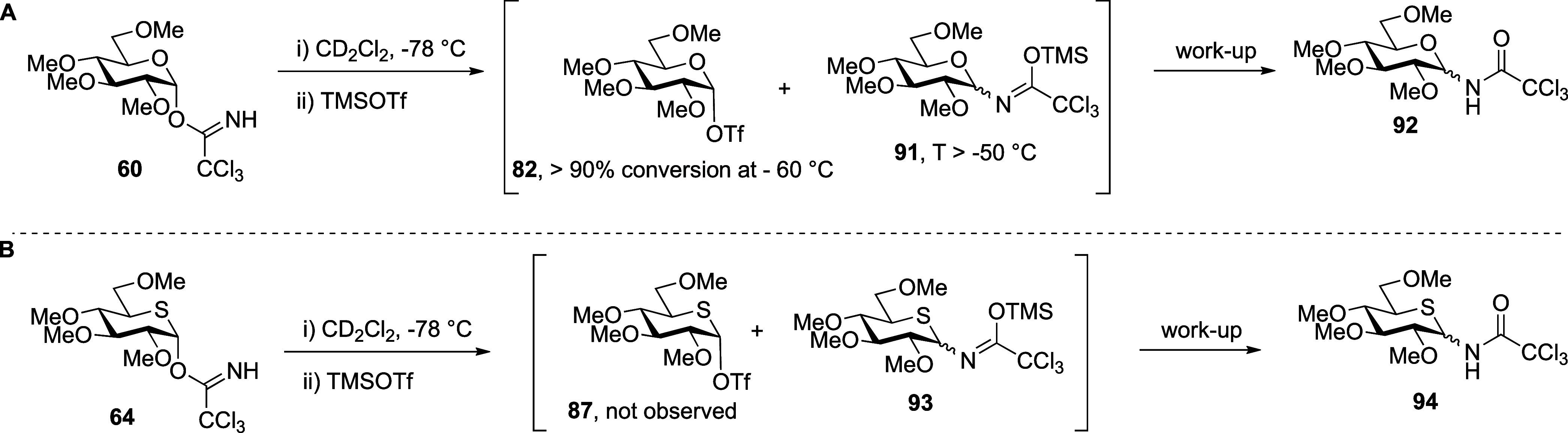
Intermediates and Products Generated during VT NMR Experiments with (A) Permethylated Trichloroacetoimidate **60** and (B) Permethylated 5-Thioglucosyl Trichloroacetoimidate **64**

Finally, we performed VT NMR experiments with per-*O*-methyl trichloroacetoimidates **60** and **64** (Figure 9 and Scheme 14A). The covalent triflate **82** was generated immediately after the addition of TMSOTf to the parent glucosyl trichloroacetoimidate **60** at −78 °C, and by −60 °C, more than 90% of the starting material **60** was converted into triflate **82**. Above −50 °C, triflate **82** was converted to the presumed imidate **91**, which, after aqueous workup, produced trichloroacetoamide **92** as the only isolated product (Figure 9A). In stark contrast, no evidence was found to support the formation of a covalent thioglucosyl triflate **89** on activation of 5-thioglycosyl trichloroacetoimidate **64** (Figure 9B and Scheme 14B) with TMSOTf at −78 °C or on gradual warming. Rather, slow conversion directly to the presumed imidate **93** was observed, resulting after workup in trichloroacetoamide **94**, which was the only product isolated. Critically, even for these armed systems, no evidence was found to support the formation of a thiocarbenium ion in this experiment.

The ensemble of VT NMR experiments described above reveals clear differences in reactivity between glucopyranosyl donors and 5-thioglucopyranosyl donors on activation at −78 °C and on subsequent warming. The first difference is apparent in the decomposition of the disarmed peracetylated “activated sulfoxides” generated by the reaction of peracetyl glucosyl and thioglucosyl ethyl sulfoxides with triflic anhydride. In the case of the thioglucosyl system, the “activated sulfoxide” persists in the reaction mixture at a higher temperature than in the case of the analogous glucosyl system. After the conversion of the “activated sulfoxide” to the dioxalenium ion and the corresponding glycosyl triflate, the relative proportions of these two intermediates vary between the glucosyl and thioglucosyl systems, with both being formed in approximately equal ratios in the glucosyl system but with the covalent triflate predominating for the thioglucosyl system (Figure 3). A directly analogous pattern was seen when attention was shifted to the peracetylated glucosyl and thioglucosyl trichloroacetimidates, with the glucosyl system undergoing complete activation at a lower temperature than that of the corresponding 5-thioglucosyl system (Figure 6). Moreover, in full agreement with the peracetylated sulfoxides, the glucopyranosyl trichloroacetimidate yielded a dioxalenium ion-glycosyl triflate mixture whose proportions changed with temperature, consistent with the observations of Huang, Whitfield and co-workers,^78^ but which nevertheless contained significant amounts of both intermediates. On the other hand, the activated peracetyl 5-thioglucosyl trichloroacetimidate was transformed into mixtures containing significantly greater proportions of the covalent triflate compared to the dioxalenium ion arising from participation by the neighboring acetoxy group. In other words, neighboring group participation is apparently less favorable in the 5-thioglucopyranosyl series than in the glucopyranosyl series, with respect to the corresponding glycosyl triflates. This observation might be interpreted in terms of the greater stability of the fused dioxalenium ion in the glucosyl series or in terms of the reduced 1,3-diaxial interactions in the case of the thioglucosyl system, as reflected in the greater anomeric effect seen in the 5-thioglycopyranosides^12,13^ and the consequent greater stability of the axial thioglucosyl triflate. For the armed permethylated donors, very distinct differences were seen between the glucosyl and the thioglucosyl series. For the sulfoxides, activation of the simple glucopyranosyl donor gave rise to the formation of the anticipated glycosyl triflate at −78 °C, whereas the corresponding 5-thioglucopyranosyl system did not provide an observable glycosyl triflate at any temperature, with the “activated sulfoxide” seemingly undergoing direct decomposition at higher temperatures. Again, a similar pattern was seen with the armed trichloroacetimidates with the glucosyl system readily yielding the glycosyl triflate on activation and gradual warming. No such triflate was seen on activation of the thioglucosyl trichloroacetimidate at any temperature, with the activated imidate seemingly converted directly to the rearranged thioglucosyl trichloroacetamide in the reaction mixture. Overall, it is apparent that, following activation with either triflic anhydride for the sulfoxides or TMSOTf for the trichloroacetimidates, the 5-thioglucopyranosyl donors are less reactive than the corresponding glucopyranosyl donors, whether armed or disarmed.

One interesting question concerns the contrast between the formation of a glycosyl triflate from the disarmed 5-thioglycosyl donors **40**, **42**, and **1** and the absence of such covalent triflate on activation of the corresponding armed 5-thioglycosyl donors **45**, **48**, and **64**. We suggest that the initial positively charged activated donors, triflated sulfoxides or silylated trichloroacetimidates, are destabilized in the disarmed peracetyl series and so more reactive with respect toward triflate formation than in the armed permethylated series.

To further probe the differential reactivity of the glucopyranosyl and 5-thioglucopyranosyl donors, we again turned to computation. Cognizant of the importance of counterions in glycosylation and related reactions^56,81^ and of previous difficulties in computing oxocarbenium ion-counterion pairs in the absence of artificial constraints preventing spontaneous collapse to covalent adducts,^82,83^ we turned to the method of Hosoya, Kosma and co-workers. In this method, four explicit molecules of dichloromethane are included to stabilize the triflate counterion by hydrogen-bonding, as shown to correlate well with the experiment.^84−87^ These gas phase computations including explicit solvent were optimized at the M06–2x/aug-cc-pVTZ level with cc-pVTZ on H,C, and S; the solvent phase calculations were optimized using SMD, which is a universal solvation model utilizing electron densities, at the M06–2*x*/6-31G* level of theory. The overall final Gibbs Free Energy (Δ*G*) is the summation of the electronic energy with the zero point energy (ZPE) correction (δ*E*) and the Gibbs Free Energy in the gas phase with four solvent molecules (δ*G*_g_), and computations with implicit solvation by dichloromethane using SMD (δ*G*_s_) (eq 1).
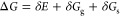
1

**Figure 10:**
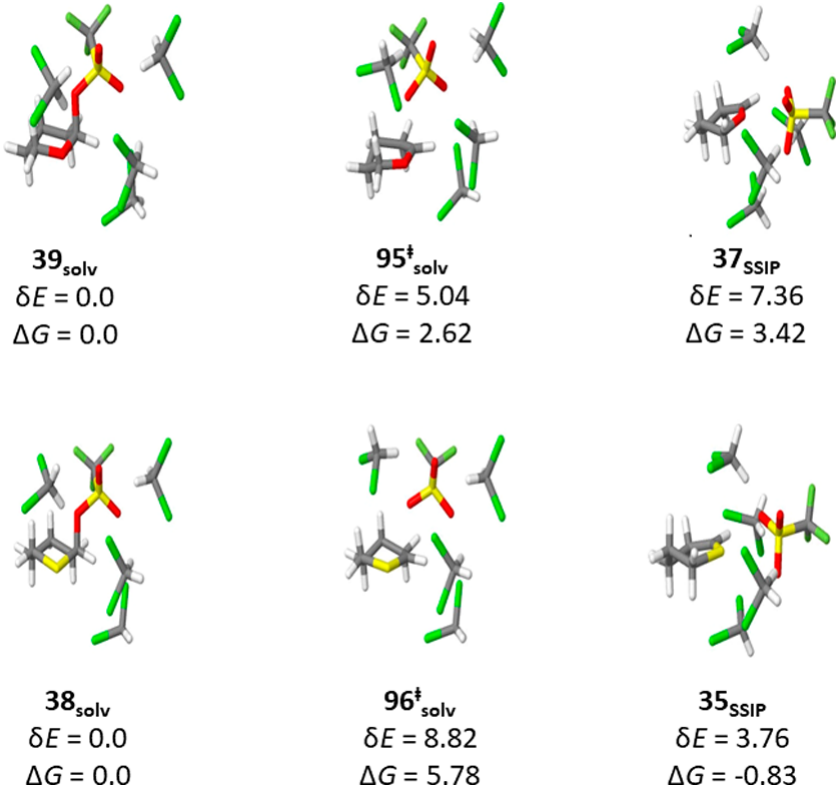
Computed Δ*G* and δ*E* for the tetrahydropyranyl triflate **39**_**solv**_ and tetrahydrothiopyranyl triflate **38**_**solv**_, SSIPs **37**_**SSIP**_ and **35**_**SSIP**_, and intervening transition states **95**_**solv**_^**‡**^ and **96**_**solv**_^**‡**^. All energies are in kcal/mol and are reported for calculations at STP.

In this manner, and using simple model systems to reduce computational costs, we obtained δ*E* and Δ*G* for the tetrahydropyranyl triflate **39**_**solv**_ and the tetrahydrothiopyranyl triflate **38**_**solv**_, and the corresponding oxocarbenium and thiocarbenium ions **37**_**SSIP**_ and **35**_**SSIP**_ in the confines of solvent-separated ion pairs (SSIPs) with the triflate anion and the transition states linking them **95**_**solv**_^**‡**^ and **96**_**solv**_^**‡**^. All structures were stabilized by four explicit molecules of dichloromethane (Figure 10). As expected, the two covalent triflates **39**_**solv**_ and **38**_**solv**_ adopt chair-like conformations with the triflate group in the axial position. Both transition states **95**_**solv**_^**‡**^ and **96**_**solv**_^**‡**^ approximate envelope conformations of the ring with the partially cleaved C1-OTf bond some 2.13/4 Å above the developing π-system, while both SSIPs adopt half-chair conformations. In both SSIPs, the triflate ion is located approximately in the plane of the π-system and is separated from it by 2.92/5 Å.

As with the gas phase calculations in the absence of the triflate counterion (Figure 1), the formation of **37**_**SSIP**_ containing the oxocarbenium from covalent triflate **39**_**solv**_ is more endothermic than that of thiocarbenium ion containing SSIP **35**_**SSIP**_ from corresponding triflate **39**_**solv**_. Thus, in the gas phase with the four explicit molecules of dichloromethane, the energy difference (δ*E*) between the covalent triflate and the SSIP in the oxygen-based system is 7.36 kcal/mol, while it is only 3.76 kcal/mol in the sulfur-based system. With correction for implicit solvation by dichloromethane, the oxocarbenium ion-triflate SSIP **37**_**SSIP**_ is 4.25 kcal/mol higher in energy than the thiocarbenium ion-triflate SSIP, both with respect to the corresponding covalent triflates (Figure 10). Overall, whether in the gas phase in the absence of a counterion (Figure 1), or in an SSIP supported by four explicit molecules of dichloromethane, or in an SSIP with four explicit molecules of dichloromethane and an implicit dichloromethane solvent shell (Figure 10), the thiocarbenium ion **35** is 2.5–4.2 kcal/mol lower in energy than the corresponding oxocarbenium ion **37**, always with respect to the corresponding covalent triflates. In contrast to the enhanced stability of the thiocarbenium ion over the oxocarbenium ion, the activation energy required to access transition state **96**_**solv**_^**‡**^ for fragmentation of covalent thianyl triflate **38**_**solv**_ to SSIP **35**_**SSIP**_ is greater than that needed to achieve transition state **95**_**solv**_^**‡**^ en route from tetrahydropyranyl triflate **39**_**solv**_ to oxocarbenium ion **37**_**SSIP**_. This is true with or without implicit solvation by dichloromethane. We tentatively attribute the low energy barrier shown by transition state **95**_**solv**_^**‡**^ to the ZPE correction given the large number of low vibrational modes in the system on a shallow energy surface. Overall, the picture that emerges from this computational study is one in which the rate of formation of the thiocarbenium ion is slower than that of the corresponding oxocarbenium ion, consistent with the various variable temperature NMR studies detailed above. However, under equilibrium conditions, the thiocarbenium ion is populated to a greater extent than the corresponding oxocarbenium ion, consistent with the enhanced axial selectivity widely reported in glycosylation reactions of 5-thioglycopyranosyl donors. This picture is directly analogous to that proposed for the fragmentation reactions of monothioacetals by Richard and co-workers.^46,47^

Finally, we note the contrast between the activated forms **65**, **66**, **80**, and **81** of the per-*O*-acetyl and per-*O*-methyl *S*-ethyl glucosyl sulfoxides **50**, **51**, **53**, and **55** (Schemes 9 and 13) that only converted slowly to the corresponding glycosyl triflates, and the rapid clean, conversion of the corresponding per-*O*-acetyl and per-*O*-methyl *S*-aryl glucosyl sulfoxides to glycosyl triflates on activation at −78 °C.^69−72,78,88^ We attribute this difference in reactivity to the electron-donating character of the ethyl group in the *S*-ethyl series employed here, which stabilizes the initially formed glycosyl oxysulfonium ions (activated sulfoxides) and retards their conversion to the triflates.

## Conclusions

Low-temperature protonation of 2,3-dihydro-4*H*-thiopyran with triflic acid in CD_2_Cl_2_ enabled the generation and NMR characterization for the first time of a thiocarbenium ion in the absence of stabilizing substituents. Activation of the 5-thioglucosyl ethyl sulfoxides, whether protected with acetyl or methyl groups, yielded thioglucosyl oxysulfonium ions that were noticeably more persistent than those in the analogous glucosyl series, indicative of their slower conversion to the thioglucosyl triflates. A similar pattern was observed with the analogous glucopyranosyl and 5-thioglucopyranosyl trichloroacetimidates on activation at a low temperature with TMSOTf in CD_2_Cl_2_. Significantly greater proportions of the fused dioxalenium ions arising from acetyl group participation with respect to the corresponding glucosyl triflates were seen on activation of the glucosyl substrates than of the analogous 5-thioglucosyl donors. Neighboring group participation is, therefore, less favored in 5-thioglycosyl donors than in simple glycosyl donors, consistent with the lower 1,2-*trans* selectivity typically observed under preparative conditions. No evidence was found for the formation of a persistent 5-thioglucopyranosyl cation under any of the conditions employed, no doubt owing to the strongly destabilizing effect of the multiple electron-withdrawing C–O–bonds in the sugar-based systems.

DFT studies support the conclusion that while the thiocarbenium ions are more stable than the oxocarbenium ions, the activation barrier to their formation is higher, consistent with the suggestions of Richard in explanation of the reactivity of benzaldehyde-derived monthioacetals.^47^

In summary, experimental observations of the greater axial selectivity in glycosylation reactions conducted with 5-thioglycopyranosyl donors compared to analogous glycopyranosyl donors are the result of multiple factors: (i) the lower kinetic reactivity of the 5-thioglycosyl systems which in turn necessitates higher reaction temperatures, (ii) the greater stability of the thiocarbenium ion over the oxocarbenium ion which facilitates equilibration under thermodynamic conditions, (iii) the increased anomeric effect in the 5-thiosugars, and (iv) less favorable stereodirecting neighboring group participation in the per-esterified 5-thiosugar donors relative to the corresponding sugars.

## Methods

### Protonation of 2,3-Dihydro-4*H*-Thiopyran **34** with TfOH at −88 °C

2,3-Dihydrothiopyran **34** (30 mg, 0.3 mmol) was dissolved in CD_2_Cl_2_ (0.75 mL), and the reaction mixture was transferred into a vacuum-dried NMR tube and sealed with a septum cap under an Ar atmosphere. The NMR tube then was placed into the NMR probe and cooled down to −88 °C, and ^1^H, ^13^C and spectra were collected. The tube was quickly removed from the probe, precooled TfOH (58 μL, 0.66 mmol, 2.2 equiv) was quickly added, and the tube was quickly shaken before the tube was returned to the cold NMR probe. ^1^H, DEPT, ^13^C, HMQC, HMBC, and ^19^F spectra were recorded as soon as possible at −88 °C. The temperature then was increased to −78 °C and ^1^H and ^19^F spectra were recorded after 5 min. After that, the temperature was increased to −60 °C, and ^1^H and ^19^F spectra were recorded after 5 min. After that, the temperature was increased by 10 °C increments, and ^1^H and ^19^F spectra were recorded after 5 min at each temperature. After termination of the VT NMR experiment, the reaction mixture was diluted with EtOAc (30 mL), washed with NaHCO_3_ (10 mL) and brine (20 mL), dried over MgSO_4_, and concentrated to dryness to give a complex mixture (18 mg) of self-condensation products containing dimer **36**.

Diagnostic signals for thienium ion **35**: ^1^H NMR (500 MHz, CD_2_Cl_2_): δ 11.23 (s, 1H, H-1), 3.72–3.60 (m, 1H, H-5), 2.44–1.57 (m, 1H, H-2). ^13^C NMR (126 MHz, CD_2_Cl_2_): δ 238.3 (C=S^+^), 40.2 (C5), 20.7 (C2). Diagnostic signals for self-condensation product **36**:^89^^1^H NMR (500 MHz, CDCl_3_): δ 5.99 (s, 1H), 3.27–3.21 (m, 1H). ^13^C NMR (126 MHz, CDCl_3_): δ 132.4, 115.4. HRMS (*m*/*z*): [M + H]^+^ calcd. for C_10_H_17_S_2_^+^ 201.0766, found 201.0760.

## Data Availability

The data underlying this study are available in the published article and its Supporting Information.

## Supporting Information Available

Full experimental details; copies of all 1 and 2D NMR spectra; details of the computational studies; and Cartesian coordinates for all computed structures (PDF)
