# Supplementary material for: Why Are 5-Thioglycopyranosyl Donors More Axially Selective than their Glycopyranosyl Counterparts? A Low and Variable Temperature NMR Spectroscopy and Computational Study
Source: JACS Au. 2025 Jan 23;5(2):871–89. doi: 10.1021/jacsau.4c01113 (PMC11863163; doi:10.1021/jacsau.4c01113)
Supplement: Supplementary file 1 — au4c01113_si_001.pdf [file au4c01113_si_001.pdf]

Supplementary Information

for

Why Are 5-Thioglycopyranosyl Donors More Axially Selective than their Glycopyranosyl Counterparts? A Low and Variable Temperature NMR Spectroscopy and Computational Study

Daniil Ahiadorme,[a,b] R. Houston Givhan,[a,c] Henry F. Schaefer III,[a,c] and David Crich[a,b,d,*]

[a] Department of Chemistry, University of Georgia, 302 East Campus Road, Athens, Georgia 30602, United States

[b]Department of Pharmaceutical and Biomedical Sciences, University of Georgia, 250 West Green Street, Athens, Georgia 30602, United States

[c]Center for Computational Quantum Chemistry, University of Georgia, 1004 Cedar St, Athens, Georgia 30602 United States

[d] Complex Carbohydrate Research Center, University of Georgia, 315 Riverbend Road, Athens, Georgia 30602, United States

[*]Correspondence to david.crich@uga.edu

Contents

1. Experimental protocols 10

1.1. General Experimental 10

1.2. Preparation of thiopyran derivatives 11

1.3. Preparation of 5-thioglucosyl sulfoxides 13

1.4. Summarized comparison of the key ^{13}C NMR peaks of 5-thioglucosyl endo- and exo-sulfoxides. 23

1.5. Preparation of glucosyl sulfoxides 24

1.6. Preparation glucosyl trichloroacetimidates 31

1.7. Preparation 5-thioglucosyl trichloroacetimidates 35

2. Computational Methods 39

2.1. NMR chemical shift calculations 39

2.2. Transition state energies calculations 39

2.3. Explicit calculations 39

2.4. Implicit calculations 39

2.5. Gibbs Free Energy calculations 40

2.6. Cartesian coordinates of computed structres 40

3. Variable temperature NMR experiments 61

3.1. Experimental protocols of Variable Temperature NMR experiments with 2,3-dihydro-4*H*-thiopyran, 2,3-dihydro-4*H*-pyran, and phenyl vinyl and ethyl vinyl sulfides . 61

3.2. Experimental protocols of Variable Temperature NMR experiments with various glucosyl and 5-thioglucosyl donors and characterization data of reactive intermediates 66

3.3. Summary of VT NMR experiments with disarmed glucosyl and 5-thioglucosyl donors 80

3.4. Summary of VT NMR experiments with armed glucosyl and 5-thioglucosyl donors 81

3.5. VT NMR spectra from experiments with 2,3-dihydro-4H-thiopyran (34) and 2,3-dihydro-4H-pyran (S1). 82

3.6. VT NMR spectra from experiments with phenyl vinyl and ethyl vinyl sulfides . 99

3.7. VT NMR spectra from experiments with peracetylated sulfoxides (50, 51)... 103

3.8. VT NMR spectra from experiments with peracetylated 5-thioglycosyl sulfoxides (40, 42) 121

3.9. VT NMR spectra from experiments with peracetylated trichloroacetimidates (57, 1) 139

3.10. VT NMR spectra from experiments with permethylated glucosyl sulfoxides (53, 55): 155

3.11. VT NMR spectra from experiments with permethylated 5-thioglucosyl sulfoxides (45, 48): 178

3.12. VT NMR spectra from experiments with permethylated glycosyl trichloroacetimidates (60 and 64): 192

4. NMR spectra of synthesized compounds and isolated decomposition products.. 206

^{1}H NMR (500 MHz, $CDCl_3$) spectrum of 2,3-dihydro-4H-thiopyran (34): 206

^{13}C NMR (125.67 MHz, $CDCl_3$) spectrum of 2,3-dihydro-4H-thiopyran (34): 207

^{1}H NMR (500 MHz, $CDCl_3$) spectrum of (R_S),(S_S)-ethyl 2,3,4,6-tetra-*O*-acetyl-1,5-dithio-*β*-D-glucopyranosyl-1-*S*-oxides (40): 208

^{13}C NMR (125.67 MHz, $CDCl_3$) spectrum of (R_S),(S_S)-ethyl 2,3,4,6-tetra-*O*-acetyl-1,5-dithio-*β*-D-glucopyranosyl-1-*S*-oxides (40): 209

COSY ($CDCl_3$) spectrum of (R_S),(S_S)-ethyl 2,3,4,6-tetra-*O*-acetyl-1,5-dithio-*β*-D-glucopyranosyl-1-*S*-oxides (40): 210

HSQC ($CDCl_3$) spectrum of (R_S),(S_S)-ethyl 2,3,4,6-tetra-*O*-acetyl-1,5-dithio-*β*-D-glucopyranosyl-1-*S*-oxides (40): 211

^{1}H NMR (500 MHz, $CDCl_3$) spectrum of (R_S),(S_S)-ethyl 2,3,4,6-tetra-*O*-acetyl-1,5-dithio-*β*-D-glucopyranosyl-5-*S*-oxides (41): 212

^{13}C NMR (125.67 MHz, $CDCl_3$) spectrum of (R_S),(S_S)-ethyl 2,3,4,6-tetra-*O*-acetyl-1,5-dithio-*β*-D-glucopyranosyl-5-*S*-oxides (41): 213

COSY ($CDCl_3$) spectrum of (R_S),(S_S)-ethyl 2,3,4,6-tetra-*O*-acetyl-1,5-dithio-*β*-D-glucopyranosyl-5-*S*-oxides (41): 214

HMQC ($CDCl_3$) spectrum of (R_S),(S_S)-ethyl 2,3,4,6-tetra-*O*-acetyl-1,5-dithio-*β*-D-glucopyranosyl-5-*S*-oxides (41): 215

^{1}H NMR (500 MHz, $CDCl_3$) spectrum of ethyl 2,3,4,6-tetra-*O*-acetyl-1,5-di-thio-*α*-D-glucopyranosyl-1-*S*-Oxide (42): 216

^{13}C NMR (125.67 MHz, $CDCl_3$) spectrum of ethyl 2,3,4,6-tetra-*O*-acetyl-1,5-di-thio-*α*-D-glucopyranosyl-1-*S*-Oxide (42): 217

COSY ($CDCl_3$) spectrum of ethyl 2,3,4,6-tetra-*O*-acetyl-1,5-di-thio-*α*-D-glucopyranosyl-1-*S*-Oxide (42): 218

HSQC ($CDCl_3$) spectrum of ethyl 2,3,4,6-tetra-*O*-acetyl-1,5-di-thio-*α*-D-glucopyranosyl-1-*S*-Oxide (42): 219

^{1}H NMR (500 MHz, $CDCl_3$) spectrum of ethyl 2,3,4,6-tetra-*O*-methyl-1,5-di-thio-*β*-D-glucopyranoside (44): 220

^{13}C NMR (125.67 MHz, $CDCl_3$) spectrum of ethyl 2,3,4,6-tetra-*O*-methyl-1,5-di-thio-*β*-D-glucopyranoside (44): 221

COSY ($CDCl_3$) spectrum of ethyl 2,3,4,6-tetra-*O*-methyl-1,5-di-thio-*β*-D-glucopyranoside (44): 222

HSQC ($CDCl_3$) spectrum of ethyl 2,3,4,6-tetra-*O*-methyl-1,5-di-thio-*β*-D-glucopyranoside (44): 223

^{1}H NMR (500 MHz, CD_2Cl_2) spectrum of (R_S),(S_S)-ethyl 2,3,4,6-tetra-*O*-methyl-1,5-dithio-*β*-D-glucopyranoside-1-*S*-Oxides (45): 224

^{13}C NMR (125.67 MHz, CD_2Cl_2) spectrum of (R_S),(S_S)-ethyl 2,3,4,6-tetra-*O*-methyl-1,5-dithio-*β*-D-glucopyranoside-1-*S*-Oxides (45): 225

DEPT-90 (CD_2Cl_2) spectrum of (*R_S*),(*S_S*)-ethyl 2,3,4,6-tetra-*O*-methyl-1,5-dithio-*β*-D-glucopyranoside-1-*S*-Oxides (45): 226

COSY (CD_2Cl_2) spectrum of (*R_S*),(*S_S*)-ethyl 2,3,4,6-tetra-*O*-methyl-1,5-dithio-*β*-D-glucopyranoside-1-*S*-Oxides (45): 227

HSQC (CD_2Cl_2) spectrum of (*R_S*),(*S_S*)-ethyl 2,3,4,6-tetra-*O*-methyl-1,5-dithio-*β*-D-glucopyranoside-1-*S*-Oxides (45): 228

^{1}H NMR (500 MHz, $CDCl_3$) spectrum of (*R_S*),(*S_S*)-ethyl 2,3,4,6-tetra-*O*-methyl-1,5-dithio-*β*-D-glucopyranoside-5-*S*-Oxides (46):........ 229

^{13}C NMR (125.67 MHz, $CDCl_3$) spectrum of (*R_S*),(*S_S*)-ethyl 2,3,4,6-tetra-*O*-methyl-1,5-dithio-*β*-D-glucopyranoside-5-*S*-Oxides (46):........ 230

COSY ($CDCl_3$) spectrum of (*R_S*),(*S_S*)-ethyl 2,3,4,6-tetra-*O*-methyl-1,5-dithio-*β*-D-glucopyranoside-5-*S*-Oxides (46): 231

HSQC ($CDCl_3$) spectrum of (*R_S*),(*S_S*)-ethyl 2,3,4,6-tetra-*O*-methyl-1,5-dithio-*β*-D-glucopyranoside-5-*S*-Oxides (46): 232

^{1}H NMR (500 MHz, $CDCl_3$) spectrum of ethyl 2,3,4,6-tetra-*O*-methyl-1,5-di-thio-*α*-D-glucopyranoside (47): 233

^{13}C NMR (125.67 MHz, $CDCl_3$) spectrum of ethyl 2,3,4,6-tetra-*O*-methyl-1,5-di-thio-*α*-D-glucopyranoside (47):........ 234

COSY ($CDCl_3$) spectrum of ethyl 2,3,4,6-tetra-*O*-methyl-1,5-di-thio-*α*-D-glucopyranoside (47): 235

HSQC ($CDCl_3$) spectrum of ethyl 2,3,4,6-tetra-*O*-methyl-1,5-di-thio-*α*-D-glucopyranoside (47): 236

^{1}H NMR (500 MHz, CD_2Cl_2) spectrum of (*R_S*),(*S_S*)-ethyl 2,3,4,6-tetra-*O*-methyl-1,5-di-thio-*α*-D-glucopyranosyl-1-*S*-Oxides (48):........ 237

^{13}C NMR (125.67 MHz, CD_2Cl_2) spectrum of (*R_S*),(*S_S*)-ethyl 2,3,4,6-tetra-*O*-methyl-1,5-di-thio-*α*-D-glucopyranosyl-1-*S*-Oxides (48):........ 238

DEPT-135 (CD_2Cl_2) spectrum of (*R_S*),(*S_S*)-ethyl 2,3,4,6-tetra-*O*-methyl-1,5-di-thio-*α*-D-glucopyranosyl-1-*S*-Oxides (48):........ 239

COSY (CD_2Cl_2) spectrum of (R_S),(S_S)-ethyl 2,3,4,6-tetra-*O*-methyl-1,5-di-thio-*α*-D-glucopyranosyl-1-*S*-Oxides (48): 240

HSQC (CD_2Cl_2) spectrum of (R_S),(S_S)-ethyl 2,3,4,6-tetra-*O*-methyl-1,5-di-thio-*α*-D-glucopyranosyl-1-*S*-Oxides (48): 241

^{1}H NMR (500 MHz, $CDCl_3$) spectrum of (R_S),(S_S)-ethyl 2,3,4,6-tetra-*O*-acetyl-1-thio-*β*-D-glucopyranosyl-1-*S*-oxides (50): 242

^{13}C NMR (125.67 MHz, $CDCl_3$) spectrum of (R_S),(S_S)-ethyl 2,3,4,6-tetra-*O*-acetyl-1-thio-*β*-D-glucopyranosyl-1-*S*-oxides (50): 243

^{1}H NMR (500 MHz, $CDCl_3$) spectrum of (R_S)-ethyl 2,3,4,6-tetra-*O*-acetyl-1-thio-*α*-D-glucopyranosyl-1-*S*-oxide (51): 244

^{13}C NMR (125.67 MHz, $CDCl_3$) spectrum of (R_S)-ethyl 2,3,4,6-tetra-*O*-acetyl-1-thio-*α*-D-glucopyranosyl-1-*S*-oxide (51): 245

^{1}H NMR (500 MHz, CD_2Cl_2) spectrum of (R_S),(S_S)-Ethyl 2,3,4,6-tetra-*O*-methyl-1-thio-*β*-D-glucopyranoside-1-*S*-Oxides (53): 246

^{13}C NMR (125.67 MHz, CD_2Cl_2) spectrum of (R_S),(S_S)-Ethyl 2,3,4,6-tetra-*O*-methyl-1-thio-*β*-D-glucopyranoside-1-*S*-Oxides (53): 247

DEPT-90 (CD_2Cl_2) spectrum of (R_S),(S_S)-Ethyl 2,3,4,6-tetra-*O*-methyl-1-thio-*β*-D-glucopyranoside-1-*S*-Oxides (53): 248

COSY (CD_2Cl_2) spectrum of (R_S),(S_S)-Ethyl 2,3,4,6-tetra-*O*-methyl-1-thio-*β*-D-glucopyranoside-1-*S*-Oxides (53): 249

HSQC (CD_2Cl_2) spectrum of (R_S),(S_S)-Ethyl 2,3,4,6-tetra-*O*-methyl-1-thio-*β*-D-glucopyranoside-1-*S*-Oxides (53): 250

^{1}H NMR (500 MHz, CD_2Cl_2) spectrum of ethyl 2,3,4,6-tetra-*O*-methyl-1-thio-*α*-D-glucopyranoside-1-*S*-oxide (55): 251

^{13}C NMR (125.67 MHz, CD_2Cl_2) spectrum of ethyl 2,3,4,6-tetra-*O*-methyl-1-thio-*α*-D-glucopyranoside-1-*S*-oxide (55): 252

COSY (CD_2Cl_2) spectrum of ethyl 2,3,4,6-tetra-*O*-methyl-1-thio-*α*-D-glucopyranoside-1-*S*-oxide (55): 253

HSQC (CD_2Cl_2) spectrum of ethyl 2,3,4,6-tetra-*O*-methyl-1-thio-*α*-D-glucopyranoside-1-*S*-oxide (55): 254

^{1}H NMR (500 MHz, $CDCl_3$) spectrum of 2,3,4,6-tetra-*O*-acetyl-*α*-D-glucopyranosyl trichloroacetoimidate (57): 255

^{13}C NMR (125.67 MHz, $CDCl_3$) spectrum of 2,3,4,6-tetra-*O*-acetyl-*α*-D-glucopyranosyl trichloroacetoimidate (57): 256

^{1}H NMR (500 MHz, $CDCl_3$) spectrum of 2,3,4,6-tetra-*O*-methyl-*α,β*-D-glucopyranosyl trichloroacetimidate (60): 257

^{13}C NMR (125.67 MHz, $CDCl_3$) spectrum of 2,3,4,6-tetra-*O*-methyl-*α,β*-D-glucopyranosyl trichloroacetimidate (60): 258

DEPT-90 ($CDCl_3$) spectrum of 2,3,4,6-tetra-*O*-methyl-*α,β*-D-glucopyranosyl trichloroacetimidate (60): 259

COSY ($CDCl_3$) spectrum of 2,3,4,6-tetra-*O*-methyl-*α,β*-D-glucopyranosyl trichloroacetimidate (60): 260

HSQC ($CDCl_3$) spectrum of 2,3,4,6-tetra-*O*-methyl-*α,β*-D-glucopyranosyl trichloroacetimidate (60): 261

^{1}H NMR (500 MHz, $CDCl_3$) spectrum of 2,3,4,6-tetra-*O*-acetyl-5-thio-*α*,*β*-D-glucopyranosyl trichloroacetoimidate (1): 262

^{13}C NMR (125.67 MHz, $CDCl_3$) spectrum of 2,3,4,6-tetra-*O*-acetyl-5-thio-*α*,*β*-D-glucopyranosyl trichloroacetoimidate (1): 263

^{1}H NMR (500 MHz, CD_2Cl_2) spectrum of 2,3,4,6-tetra-*O*-methyl-5-thio-*α*,*β*-D-glucopyranoside (63): 264

^{13}C NMR (125.67 MHz, CD_2Cl_2) spectrum of 2,3,4,6-tetra-*O*-methyl-5-thio-*α*,*β*-D-glucopyranoside (63): 265

DEPT-90 (CD_2Cl_2) spectrum of 2,3,4,6-tetra-*O*-methyl-5-thio-*α*,*β*-D-glucopyranoside (63): 266

COSY (CD_2Cl_2) spectrum of 2,3,4,6-tetra-*O*-methyl-5-thio-*α*,*β*-D-glucopyranoside (63): 267

HSQC (CD_2Cl_2) spectrum of 2,3,4,6-tetra-*O*-methyl-5-thio-*α*,*β*-D-glucopyranoside (63): 268

^{1}H NMR (500 MHz, $CDCl_3$) spectrum of 2,3,4,6-tetra-*O*-methyl-5-thio-*α*-D-glucopyranosyl trichloroacetoimidate (64): 269

^{13}C NMR (125.67 MHz, $CDCl_3$) spectrum of 2,3,4,6-tetra-*O*-methyl-5-thio-*α*-D-glucopyranosyl trichloroacetoimidate (64): 270

DEPT-90 ($CDCl_3$) spectrum of 2,3,4,6-tetra-*O*-methyl-5-thio-*α*-D-glucopyranosyl trichloroacetoimidate (64): 271

COSY ($CDCl_3$) spectrum of 2,3,4,6-tetra-*O*-methyl-5-thio-*α*-D-glucopyranosyl trichloroacetoimidate (64): 272

HSQC ($CDCl_3$) spectrum of 2,3,4,6-tetra-*O*-methyl-5-thio-*α*-D-glucopyranosyl trichloroacetoimidate (64): 273

^{1}H NMR (500 MHz, $CDCl_3$) spectrum of mixture of decomposition products 11 and 69: 274

^{13}C NMR (125.67 MHz, $CDCl_3$) spectrum of decomposition products 11 and 69: 275

COSY ($CDCl_3$) spectrum of decomposition products 11 and 69: 276

HMQC ($CDCl_3$) spectrum of decomposition products 11 and 69: 277

^{1}H NMR (500 MHz, $CDCl_3$) spectrum of decomposition product 9: 278

^{13}C NMR (125.67 MHz, $CDCl_3$) spectrum of decomposition product 9: 279

^{1}H NMR (500 MHz, $CDCl_3$) spectrum of mixture of decomposition products 11, 78: 280

^{13}C NMR (125.67 MHz, $CDCl_3$) spectrum of mixture of decomposition products 11, 78: 281

COSY ($CDCl_3$) spectrum of mixture of decomposition products 11, 78: 282

HSQC ($CDCl_3$) spectrum of mixture of decomposition products 11, 78: 283

HMBC ($CDCl_3$) spectrum of mixture of decomposition products 11, 78: 284

1H NMR (500 MHz, CDCl3) spectrum of the decomposition product 84: 285

^{13}C NMR (125.67 MHz, $CDCl_3$) spectrum of mixture of the decomposition product 84: 286
^{1}H NMR (500 MHz, $CDCl_3$) spectrum of decomposition product 90: 287
^{13}C NMR (125.67 MHz, $CDCl_3$) spectrum of decomposition product 90: 288
HSQC ($CDCl_3$) spectrum of decomposition product 90: 289
HMBC ($CDCl_3$) spectrum of decomposition product 90: 290
FTIR ($CHCl_3$) spectrum of decomposition product 90: 291
^{1}H NMR (500 MHz, $CDCl_3$) spectrum of decomposition product 92: 292
^{13}C NMR (125.67 MHz, $CDCl_3$) spectrum of decomposition product 92: 293
DEPT-90 ($CDCl_3$) spectrum of decomposition product 92: 294
COSY ($CDCl_3$) spectrum of decomposition product 92: 295
HSQC ($CDCl_3$) spectrum of decomposition product 92: 296
^{1}H NMR (500 MHz, $CDCl_3$) spectrum of decomposition product 94: 297
^{13}C NMR (125.67 MHz, $CDCl_3$) spectrum of decomposition product 94: 298
COSY ($CDCl_3$) spectrum of decomposition product 94: 299
HSQC ($CDCl_3$) spectrum of decomposition product 94: 300
References 301

1. Experimental protocols

1.1. General Experimental

All reactions were conducted in flame/oven dried glassware capped with rubber septa under an atmosphere of argon unless otherwise stated. Commercially available starting materials were used without purification, unless otherwise stated. All organic solutions were concentrated under reduced pressure on a rotary evaporator and water bath. Flash-column chromatography was performed using COMBIFLASH® NextGen system, unless otherwise stated. Thin-layer chromatography (TLC) was carried out with 250 µm glass backed silica (XHL) plates with fluorescent indicator (254 nm). TLC plates were visualized by submersion in ceric ammonium molybdate solution (CAM), or aqueous potassium permanganate solution ($KMnO_4$), or 10% sulfuric acid in ethanol followed by heating on a hot plate (120 °C). Nuclear Magnetic Resonance (NMR) spectra of all compounds were obtained in $CDCl_3$ (δ 7.27 and 77.0 ppm, respectively), CD_2Cl_2 (δ 5.32 and 53.5 ppm, respectively), using a 500 MHz EZC500 JEOL instrument. Various temperature NMR spectra were recorded using a 500 MHz EZC500 JEOL instrument. The chemical shifts (δ) are calculated with respect to residual solvent peak and are given in ppm. Multiplicities are abbreviated as follows:s (singlet), m (multiplet), br (broad), d (doublet), t (triplet), q (quartet) and comp (complex). Assignments were made with the help of COSY, HMBC, HMQC or HSQC, DEPT-135 and DEPT-90 spectra. Specific optical rotations were recorded in $CHCl_3$, at 589 nm and 20-22 °C on a digital polarimeter with a path length of 10 cm. High resolution mass spectra were obtained on a ThermoFisher Orbitrap Q-Exactive using electrospray ionization (ESI).

1.2. Preparation of thiopyran derivatives

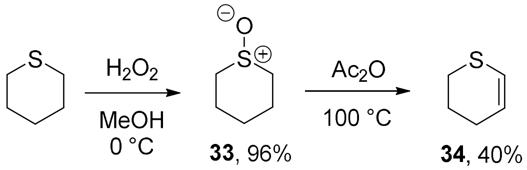

Thiane 1-oxide (33):

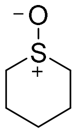

Thiane (5 g, 48.92 mmol) was dissolved in MeOH (60 mL) and the reaction mixture was cooled down to 0 °C before H_2O_2 (50% aq solution, 30 mL, 53.82 mmol, 1.1 equiv) was added. The reaction mixture then was stirred for 2h at 0 →20 °C. After such time the reaction mixture was quenched with 1M $NaHSO_3$ (10 mL), and methanol was removed under reduced pressure. The remains were distributed between $CHCl_3$ (60 mL) and brine (30 mL). The organic layer was collected, dried over $MgSO_4$ and concentrated to dryness to give desired sulfoxide **33** as a colorless viscous liquid (5.21 g, 96%) with spectral data identical to that reported in the literature.[1]

***R**f* 0.21 and 0.34 (EtOAc ($KMnO_4$)).

^{1}H NMR (500 MHz, $CDCl_3$): δ 2.93 – 2.79 (m, 2H), 2.79 – 2.61 (m, 2H), 2.27 – 2.14 (m, 2H), 1.70 – 1.43 (m, 4H).

^{13}C NMR (126 MHz, $CDCl_3$): δ 49.0, 24.7, 19.2.

ESI-HRMS (m/z): $[M+H]^+$ calcd. for $C_5H_{11}SO^+$ 119.0525, found 119.0521.

2,3-dihydro-4*H*-thiopyran (34):

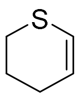

Sulfoxide **33** (5.2 g, 44 mmol) was dissolved in Ac_2O (21 mL) and the reaction mixture was stirred at 100 °C for 1h. After such time the reaction mixture was allowed to cool down to room temperature (20 °C), and was poured into sat aq $NaHCO_3$ solution with ice (100 mL) and stirred for 1h. After such time the reaction mixture was extracted with Et_2O (3×30 mL). The combined organic layers were washed with sat aq $NaHCO_3$ (3×100 mL),

brine (100 mL), dried over $MgSO_4$, and concentrated under reduced pressure (no lower than 200 mbar). The residue then was purified by flash column chromatography on silica gel eluting with hexanes:EtOAc (0→5%, EtOAc) to give the product as a colorless liquid (1.76 g, 40%) with spectral data identical to that reported in the literature.[2]

R_f 0.32 (hexanes ($KMnO_4$)).

^{1}H NMR (500 MHz, $CDCl_3$): δ 5.99 (d, *J* = 10.2, 1H), 5.70 (dt, *J* = 10.1, 4.2 Hz, 1H), 2.88 – 2.82 (m, 2H), 2.24 – 2.07 (m, 2H), 2.07 – 1.88 (m, 2H).

^{13}C NMR (126 MHz, $CDCl_3$): δ 121.2, 119.2, 26.2, 23.7, 22.4.

ESI-HRMS (m/z): $[M+H]^+$ calcd. for $C_5H_9S^+$ 101.0420, found 101.0417.

1.3. Preparation of 5-thioglucosyl sulfoxides

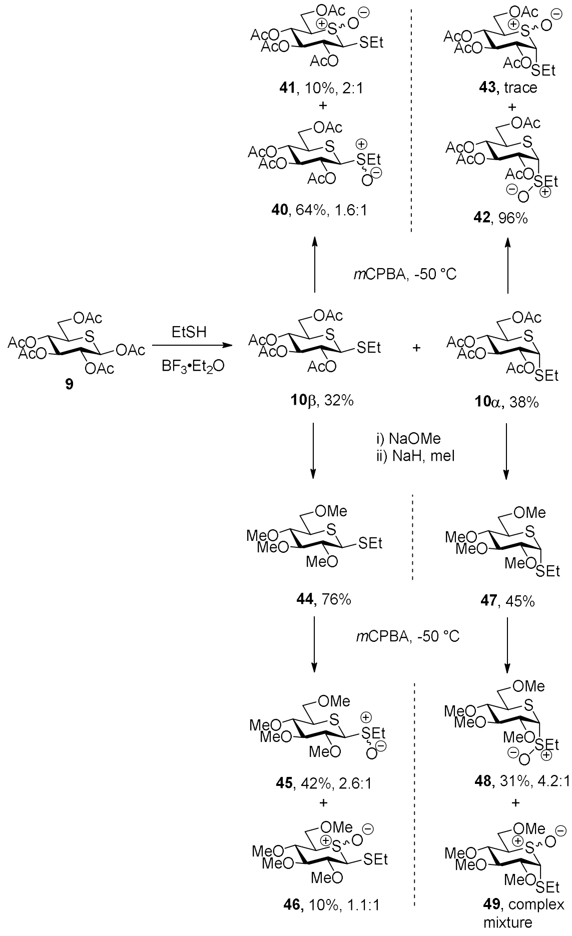

Ethyl 2,3,4,6-tetra-*O*-acetyl-1,5-dithio-D-glucopyranoside (10α, 10β):

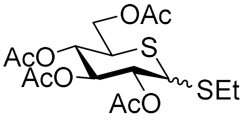

Pentaacetate **9** (3.46 g) was dissolved in anhydrous CH_2Cl_2 (50 mL) and HSEt (1.89 mL, 25.56 mmol, 3 equiv) was added followed by BF_3•Et_2O (4.2 mL, 34.08 mmol, 4 equiv) at 0 °C and the reaction mixture was then stirred with warming from 0→ 20 °C for 12h. After such time the reaction mixture was quenched with H_2O (10 mL), diluted with CH_2Cl_2 (40 mL). The organic layer was washed with sat aq $NaHCO_3$ (2×50 mL), brine (50 mL) and dried over $MgSO_4$. The crude mixture of anomers (*α : β* = 1.2 : 1) was purified by flash column chromatography on silica gel eluting with hexanes:EtOAc (0→50%, EtOAc) to give *α*-thioglycoside **10α** (1.32 g, 38%) as a yellowish syrup and *β*-thioglycoside **10β** (1.1 g, 32%) as a yellowish syrup with spectral data identical to that reported in the literature.[3]

α*:*β = 1.2:1

α-thioglycoside **10α:**

R_f 0.37 (hexanes:EtOAc 3:2 (H_2SO_4/EtOH)).

^{1}H NMR (500 MHz, $CDCl_3$): δ 5.38 (dd, *J* = 10.2, 9.3 Hz, 1H), 5.31 – 5.18 (m, 2H), 4.51 (d, *J* = 4.6 Hz, 1H), 4.42 (dd, *J* = 12.1, 4.8 Hz, 1H), 4.09 (dd, *J* = 12.1, 3.2 Hz, 1H), 3.70 (ddd, *J* = 10.8, 4.8, 3.2 Hz, 1H), 2.67 (qd, *J* = 7.4, 4.3 Hz, 2H), 2.07 (s, 3H), 2.06 (s, 3H), 2.03 (s, 3H), 2.00 (s, 3H), 1.25 (t, *J* = 7.4 Hz, 3H).

^{13}C NMR (126 MHz, $CDCl_3$): δ 170.5, 169.9, 169.5, 169.5, 77.3, 77.0, 76.7, 74.6, 72.3, 71.3, 61.2, 49.0, 39.4, 25.7, 20.7, 20.6, 20.5, 14.0.

ESI-HRMS (m/z): $[M+Na]^+$ calcd. for $C_{16}H_{24}O_8NaS_2^+$ 431.0804, found 431.0797.

β-thioglycoside **10β:**

R_f 0.32 (hexanes:EtOAc 3:2 (H_2SO_4/EtOH)).

^{1}H NMR (500 MHz, $CDCl_3$): δ 5.27 (dd, *J* = 10.7, 9.6 Hz, 1H), 5.18 (dd, *J* = 10.7, 9.5 Hz, 1H), 5.05 (t, *J* = 9.6 Hz, 1H), 4.25 (dd, *J* = 12.0, 5.7 Hz, 1H), 4.13 (dd, *J* = 12.0, 3.4 Hz, 1H), 3.83 (d, *J* = 10.7 Hz, 1H), 3.28 (ddd, *J* = 10.7, 5.7, 3.4 Hz, 1H), 2.80 – 2.63 (m, 2H), 2.08 (s, 3H), 2.07 (s, 3H), 2.03 (s, 3H), 2.00 (s, 3H), 1.26 (t, *J* = 7.4 Hz, 3H).

^{13}C NMR (126 MHz, $CDCl_3$): δ 169.8, 169.7, 169.6, 169.3, 74.5, 73.1, 71.9, 61.3, 47.7, 44.4, 24.9, 20.6, 20.5, 20.5, 20.4, 14.5.

ESI-HRMS (m/z): $[M+Na]^+$ calcd. for $C_{16}H_{24}O_8NaS_2^+$ 431.0804, found 431.0795.

Ethyl 2,3,4,6-tetra-*O*-acetyl-1,5-dithio-*β*-D-glucopyranoside-*S*-Oxides (40,41):

Thioglycoside **10β** (377 mg, 0.923 mmol) was dissolved in anhydrous CH_2Cl_2 (15 mL) and the reaction mixture was cooled down to –50 °C before mCPBA (77% wt) (227 mg, 1.015 mmol, 1.1 equiv.) solution in anhydrous CH_2Cl_2 (5 mL) was added and the reaction mixture was stirred for 1h at –50 °C. After such time the reaction mixture was quenched

with aq. sat. $NaHCO_3$ (5 mL) at –30 °C and gradually warmed up to 20 °C. After that the reaction mixture was diluted with EtO CH_2Cl_2 (40 mL), washed with aq. sat. $NaHCO_3$ (40 mL), 1M $NaHSO_3$ (60 mL), brine (60 mL), dried over $MgSO_4$ and concentrated to dryness. Crude product was purified by a flash column chromatography on a silica gel eluting with hexanes:EtOAc (20→100%, EtOAc) to give mixture of (R_s),(S_s)-1-*S*-sulfoxides **40** as a white solid (251 mg, 64%) and (R_s),(S_s)-5-*S*-sulfoxides **41** (40 mg, 10%) as a white solid.

(*R*$_s$),(*S*$_s$)-1-*S*-sulfoxides 40:

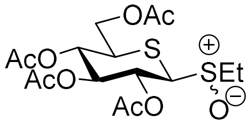

d.r. = 1.6:1

***R*$_f$** 0.61 (100% EtOAc, (H_2SO_4/EtOH)).

^{1}H NMR (500 MHz, $CDCl_3$): Major diastereomer: δ 5.58 (dd, *J* = 10.8, 9.4 Hz, 1H, H-2), 5.42 – 5.23 (m, 1H, H-4), 5.16 (t, *J* = 9.1, 1H, H-3), 4.34 (dt, *J* = 11.2, 5.5 Hz, 1H, H-6a), 4.23 – 4.15 (m, 2H, H-6b, H-1), 3.30 (ddd, *J* = 9.7, 5.8, 3.8 Hz, 1H, H-5), 3.15 (dq, *J* = 13.0, 7.5 Hz, 1H, $SC\underline{H}_2CH_3$), 2.86 – 2.73 (m, 1H, $SC\underline{H}_2CH_3$), 2.08 (s, 3H), 2.06 (s, 3H), 2.04 (s, 3H), 2.02 (s, 3H, each $C\underline{H}_3CO$), 1.43 – 1.34 (m, 3H, $SCH_2C\underline{H}_3$); **Minor diastereomer:** δ 5.42 – 5.23 (m, 2H, H-2, H-4), 5.16 (t, *J* = 9.1, 1H, H-3), 4.34 (dd, *J* = 11.2, 5.5 Hz, 1H, H-6a), 4.23 – 4.15 (m, 1H, H-6b), 3.90 (d, *J* = 10.8 Hz, 1H, H-1), 3.37 (ddd, *J* = 10.5, 5.4, 3.3 Hz, 1H, H-5), 3.03 – 2.87 (m, 1H, $SC\underline{H}_2CH_3$), 2.86 – 2.73 (m, 1H, $SC\underline{H}_2CH_3$), 2.09 (s, 3H, $C\underline{H}_3CO$), 2.07 (s, 3H, $C\underline{H}_3CO$), 2.05 (s, 3H, $C\underline{H}_3CO$), 2.04 (s, 3H, $C\underline{H}_3CO$), 1.43 – 1.34 (m, 3H, $OSCH_2C\underline{H}_3$).

^{13}C NMR (126 MHz, $CDCl_3$): Major diastereomer: δ 170.6, 170.0, 169.4, 169.0 (4×CO), 73.5 (C-3), 71.2 (C-2), 70.7 (C-4), 61.8 (C-1), 61.5 (C-6), 45.1 ($CH_3\underline{C}H_2SO$), 42.7 (C-5), 20.7, 20.6, 20.6, 20.5 (4×$\underline{C}H_3CO$), 7.7 ($\underline{C}H_3CH_2SO$); **Minor diastereomer:** δ 170.6, 170.0, 169.7, 169.5 (4×CO), 74.6 (C-3), 71.5 (C-2), 70.8 (C-4), 62.4 (C-1), 61.7 (C-6), 43.3 ($CH_3\underline{C}H_2SO$), 43.2 (C-5), 20.7, 20.7, 20.6, 20.6 (4×$\underline{C}H_3CO$), 7.32 ($\underline{C}H_3CH_2SO$).

ESI-HRMS (m/z): $[M+Na]^+$ calcd. for $C_{16}H_{24}O_{10}NaS^+$ 447.0754, found 447.0744.

(R_s),(S_s)-5-*S*-sulfoxides 41:

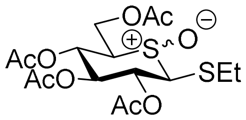

d.r. = 2:1

R_f 0.74 (100% EtOAc, (H_2SO_4/EtOH)).

^{1}H NMR (500 MHz, $CDCl_3$): (peaks assigned for the major diastereomer) δ 5.37 – 5.24 (m, 2H, H-3, H-4), 5.10 (dd, *J* = 11.9, 9.1 Hz, 1H, H-2), 4.70 (dd, *J* = 12.5, 2.5 Hz, 1H, H-6a), 4.44 – 4.37 (m, 1H, H-6b), 3.81 (d, *J* = 11.8 Hz, 1H, H-1), 3.18 (dt, *J* = 11.3, 2.4 Hz, 1H, H-5), 2.96 – 2.81 (m, 2H, $SC\underline{H}_2CH_3$), 2.10 (s, 3H), 2.07 (s, 3H), 2.03 (s, 3H), 1.99 (s, 3H, each $C\underline{H}_3CO$), 1.31 – 1.24 (m, 3H, $SCH_2C\underline{H}_3$).

^{13}C NMR (126 MHz, $CDCl_3$): δ 170.4, 169.6, 169.2, 169.0 (4×CO), 74.1 (C-4), 69.7 (C-1), 66.0 (C-2), 64.2 (C-5), 63.8 (C-3), 56.0 (C-6), 26.4 ($CH_3\underline{C}H_2SO$), 25.6 ($CH_3\underline{C}H_2SO$), 20.8, 20.6, 20.5, 20.5 (4×$\underline{C}H_3CO$), 14.4 ($\underline{C}H_3CH_2SO$).

ESI-HRMS (m/z): $[M+Na]^+$ calcd. for $C_{16}H_{24}O_{10}NaS^+$ 447.0754, found 447.0744.

Ethyl 2,3,4,6-tetra-*O*-acetyl-1,5-dithio-*α*-D-glucopyranoside-1-*S*-Oxide (42):

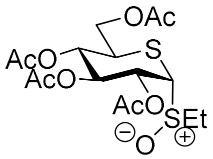

Thioglycoside **10α** (500 mg, 1.22 mmol) was dissolved in anhydrous CH_2Cl_2 (20 mL) and the reaction mixture was cooled down to –50 °C before *m*CPBA (77% wt, 300 mg, 1.342 mmol, 1.1 equiv.) solution in anhydrous CH_2Cl_2 (6 mL) was added and the reaction mixture was stirred for 1h at –50 °C. After such time the reaction mixture was quenched with aq. sat. $NaHCO_3$ (10 mL) at –50 °C, and gradually warmed up to 20 °C. After that the reaction mixture was diluted with CH_2Cl_2 (60 mL), washed with aq. sat. $NaHCO_3$ (40 mL), 1M $NaHSO_3$ (40 mL), brine (40 mL), dried over $MgSO_4$ and concentrated to dryness. The crude product was purified by flash column chromatography on silica gel eluting with

hexanes:EtOAc (0→100%, EtOAc) to give single diastereomer of exo-sulfoxide **42** as a white solid (494 mg, 96%).

Rf 0.33 (hexanes:EtOAc 1:9 (H_2SO_4/EtOH)).

[*α*]$_D^{22}$ +146.4 ($CHCl_3$, 0.0033).

^{1}H NMR (500 MHz, $CDCl_3$): δ 5.71 (d, *J* = 9.6 Hz, 1H, H-3), 5.45 (dd, *J* = 9.9, 4.7 Hz, 1H, H-2), 5.28 (dd, *J* = 10.8, 9.0 Hz, 1H, H-4), 4.26 (dd, *J* = 12.2, 5.3 Hz, 1H, H-6a), 4.15 (d, *J* = 4.6 Hz, 1H, H-1), 4.09 (dd, *J* = 12.2, 3.3 Hz, 1H, H-6b), 3.57 (ddd, *J* = 10.7, 5.3, 3.3 Hz, 1H, H-5), 3.17 (dq, *J* = 13.2, 7.6 Hz, 1H, $CH_3C\underline{H_2}SO$), 2.93 (dq, *J* = 13.2, 7.4 Hz, 1H, $CH_3C\underline{H_2}SO$), 2.09 (s, 6H, 2×$C\underline{H_3}CO$), 2.05 (s, 6H, 2×$C\underline{H_3}CO$), 1.39 (t, *J* = 7.5 Hz, 3H, $C\underline{H_3}CH_2SO$).

^{13}C NMR (126 MHz, $CDCl_3$): δ 170.5, 169.5, 169.3, 169.3 (4×CO), 73.6 (C-2), 71.7 (C-4), 70.8 (C-3), 61.1 (C-6), 57.5 (C-1), 45.7 ($CH_3\underline{C}H_2SO$), 41.0 (C-5), 20.9, 20.7, 20.6, 20.6 (4×$\underline{C}H_3CO$), 6.2 ($\underline{C}H_3CH_2SO$).

ESI-HRMS (m/z): $[M+Na]^+$ calcd. for $C_{16}H_{24}O_9NaS_2^+$ 447.0754, found 447.0742.

Ethyl 2,3,4,6-tetra-*O*-methyl-1,5-dithio-*β*-D-glucopyranoside (44):

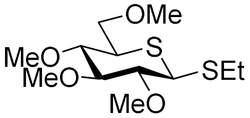

Thioglycoside **10β** (1.1 g, 2.69 mmol) was dissolved in anhydrous MeOH (40 m L) and NaOMe (58 mg, 1.077 mmol, 0.4 equiv) was added (pH 10) and the reaction mixture was stirred for 1h at 20 °C. After such time the reaction was quenched by addition of the Amberlyst 15 (H^+) ion-exchange resin until pH 3-4. The reaction mixture was filtered, and the filtrate was concentrated to dryness. The crude product was redissolved in anhydrous DMF (40 mL) and NaH (60% dispersion in the mineral oil, 540 mg, 13.47 mmol, 5 equiv) was added at 0 °C followed by addition of MeI (1.0 mL, 16.5 mmol, 6 equiv) and the reaction mixture was stirred at 0→20 °C until completion (detected by TLC and LCMS). After completion the reaction mixture was quenched with MeOH (15 mL) at 0 °C, diluted with EtOAc (70 mL), washed with H_2O (70 mL), brine (70 mL), dried over $MgSO_4$ and

concentrated to dryness. The crude product was purified by flash column chromatography on silica gel eluting with hexanes:EtOAc (0→40%, EtOAc) to give the product as a colorless syrup (604 mg, 76%).

R_f 0.58 (hexanes:EtOAc 7:3 (H_2SO_4/EtOH)).

[*α*]$_D^{22}$ +12.3 ($CHCl_3$, 0.0047).

^{1}H NMR (500 MHz, $CDCl_3$): δ 3.71 – 3.61 (m, 9H, 2×OMe, H-1, H-6a, H-6b), 3.57 (s, 3H, OMe), 3.37 (s, 3H, OMe), 3.30 (dd, *J* = 10.5, 9.0 Hz, 1H, H-4), 3.15 (dd, *J* = 10.4, 8.8 Hz, 1H, H-2), 2.96 (t, *J* = 9.0 Hz, 1H, H-3), 2.85 – 2.67 (m, 3H, H-5, $SC\underline{H}_2CH_3$), 1.29 (td, *J* = 7.4, 0.9 Hz, 3H, $SCH_2C\underline{H}_3$).

^{13}C NMR (126 MHz, $CDCl_3$): δ 90.3 (C-3), 87.2 (C-2), 83.9 (C-4), 70.7 (C-6), 61.8, 61.6, 61.1, 59.3 (4×OMe), 49.3 (C-1), 47.0 (C-5), 26.1 ($S\underline{C}H_2CH_3$), 14.8 ($SCH_2\underline{C}H_3$).

ESI-HRMS (m/z): $[M+Na]^+$ calcd. for $C_{12}H_{24}O_4NaS_2^+$ 319.1008, found 319.0995.

Ethyl 2,3,4,6-tetra-*O*-methyl-1,5-dithio-*β*-D-glucopyranoside-*S*-Oxides (45, 46):

Thioglycoside **44** (450 mg, 1.52 mmol) was dissolved in anhydrous CH_2Cl_2 (40 mL) and the reaction mixture was cooled down to -60 °C before mCPBA (77% wt, 412 mg, 1.84 mmol, 1.2 equiv) solution in CH_2Cl_2 (10 mL) was added. The reaction mixture then was stirred at -60 °C for 3h. After such time the reaction mixture was quenched with saturated aq $NaHCO_3$ and allowed to warm up to 20 °C, and then diluted with CH_2Cl_2. The organic layer was separated and washed with 1M $NaHSO_3$ (50 mL), brine (50 mL), dried over $MgSO_4$ and concentrated to dryness. The crude was purified by a flash column chromatography on a silica gel eluting with hexanes:acetone (0→60%, acetone) to give mixture of (R_s),(S_s)-1-*S*-sulfoxides **45** (200 mg, 42%) as a colorless syrup, and a mixture of (R_s),(S_s)-5-*S*-sulfoxides **46** (46 mg, 10%) as a colorless syrup.

(R_s),(S_s)-1-*S*-sulfoxides 45:

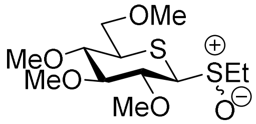

d.r. = 2.6:1

Rf 0.58 and 0.63 (hexanes:acetone 1:1 (H_2SO_4/EtOH))

^{1}H NMR (500 MHz, CD_2Cl_2):Major diastereomer:δ 3.88 – 3.77 (m, 2H, H-1, H-2), 3.70 – 3.60 (m, 1H, H-6a), 3.59 – 3.46 (m, 10H, H-6b, 3xOMe), 3.37 – 3.19 (m, 5H, H-3, H-4, OMe), 3.09 – 3.03 (m, 1H, H-3), 3.03 – 2.73 (m, 3H, H-5, SC<u>H</u>$_2$CH$_3$), 1.37 – 1.25 (m, 3H, SCH$_2$C<u>H</u>$_3$); **Minor diastereomer**:δ 3.70 – 3.60 (m, 7H, H-6a, 2xOMe), 3.59 – 3.46 (m, 5H, H-1, H-6b, OMe), 3.37 – 3.19 (m, 4H, H-4, OMe), 3.09 – 3.03 (m, 1H, H-3), 3.03 – 2.73 (m, 3H, H-5, SC<u>H</u>$_2$CH$_3$), 1.37 – 1.25 (m, 3H, SCH$_2$C<u>H</u>$_3$).

^{13}C NMR (126 MHz, CD_2Cl_2):**Major diastereomer:**δ 88.0 (C-3), 82.6 (C-4), 79.7 (C-2), 72.0 (C-6), 61.4 (C-1), 60.4 (OMe), 60.2 (OMe), 59.2 (2xOMe), 44.8 (S<u>C</u>H$_2$CH$_3$), 44.4 (C-5), 7.4 (SCH$_2$<u>C</u>H$_3$); **Minor diastereomer:**90.6 (C-3), 83.9 (C-4), 82.9 (C-2), 71.4 (C-6), 64.1 (C-1), 61.7 (OMe), 61.6 (OMe), 61.2 (OMe), 59.3 (OMe), 45.6 (C-5), 45.2 (S<u>C</u>H$_2$CH$_3$), 7.9 (SCH$_2$<u>C</u>H$_3$).

ESI-HRMS (m/z):calcd. for $C_{12}H_{24}O_5NaS_2^+$ $[M+Na]^+$ 335.0957, found 335.0933.

(*R*$_s$),(*S*$_s$)-5-*S*-sulfoxides 44:

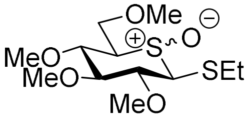

d.r. = 1.1:1

Rf 0.40 (hexanes:acetone 3:2 (H_2SO_4/EtOH))

^{1}H NMR (500 MHz, $CDCl_3$): Major diastereomer: δ 3.94 – 3.84 (m, 1H, H-6a), 3.79 (t, *J* = 10.1 Hz, 1H, H-6b), 3.66 – 3.61 (m, 6H, 2xOMe), 3.57 (s, 3H, OMe), 3.54 – 3.49 (m, 4H, OMe, H-1), 3.37 – 3.26 (m, 1H, H-4), 3.20 (t, *J* = 9.1 Hz, 1H, H-3), 2.99 (dd, *J* = 11.2, 8.7 Hz, 1H, H-2), 2.92 (q, *J* = 7.4 Hz, 2H, SC<u>H</u>$_2$CH$_3$), 2.68 – 2.56 (m, 1H, H-5), 1.29 (t, *J* = 7.4 Hz, 3H, SCH$_2$C<u>H</u>$_3$); **Minor diastereomer:** δ 3.94 – 3.84 (m, 2H, H-6b, H-6a), 3.66 – 3.61 (m, 8H, 2xOMe, H-1, H-2), 3.43 – 3.36 (m, 6H, 2xOMe), 3.37 – 3.26 (m, 1H, H-4), 3.20 (t, *J* = 9.1 Hz, 1H, H-3), 2.84 – 2.75 (m, 2H, SC<u>H</u>$_2$CH$_3$), 2.68 – 2.56 (m, 1H, H-5’), 1.29 (t, *J* = 7.4 Hz, 3H, 2xSCH$_2$C<u>H</u>$_3$).

^{13}C NMR (126 MHz, CDCl$_3$): Major diastereomer: δ 89.2 (C-3), 78.9 (C-2), 75.1 (C-4), 70.7 (C-1), 67.7 (C-6), 62.0, 61.9, 61.6, 61.3 (4×O$\underline{C}$H$_3$), 61.0 (C-5), 27.3 (S$\underline{C}$H$_2$CH$_3$), 14.6 (SCH$_2\underline{C}$H$_3$); **Minor diastereomer:** δ 89.6 (C-3), 79.5 (C-2), 67.0 (C-5), 70.7 (C-1), 64.9 (C-6), 64.6 (C-4), 61.4, 61.4 (2×O$\underline{C}$H$_3$), 61.0 (C-5), 59.4, 59.2 (2×O$\underline{C}$H$_3$), 26.5 (S$\underline{C}$H$_2$CH$_3$), 14.5 (SCH$_2\underline{C}$H$_3$).

ESI-HRMS (m/z): [M+Na]$^+$ calcd. for $C_{12}H_{24}O_5NaS_2^+$ 335.0957, found 335.0931.

Ethyl 2,3,4,6-tetra-*O*-methyl-1,5-dithio-*α*-D-glucopyranoside (47):

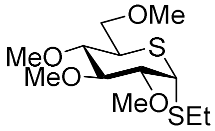

Thioglycoside **10α** (1.3 g, 3.18 mmol) was dissolved in anhydrous MeOH (30 mL) and NaOMe (68 mg, 1.272 mmol. 0.4 equiv) was added and the reaction mixture was stirred at 20 °C for 1 h. After such time the reaction mixture was quenched with Amberlyst 15 (H$^+$) ion-exchange resin until pH 5. The reaction mixture was then filtered, and the filtrate was concentrated to dryness. The crude residue was taken up in anhydrous DMF (20 mL) and the resulting solution was cooled down to 0 °C before NaH (60% dispersion in the mineral oil, 636 mg, 15.9 mmol, 5 equiv) was added followed by addition of MeI (1.2 mL, 19.08 mmol, 6 equiv). The reaction mixture was then stirred for 12h with warming from 0→20 °C. After such time the reaction mixture was quenched with MeOH 910 mL), diluted with EtOAc (80 mL), washed with H_2O (80 mL), brine (80 mL), dried over $MgSO_4$ and concentrated to dryness. The crude product was purified by a flash column chromatography eluting with hexanes:EtOAc (0→60%, EtOAc) to give the product as a yellowish syrup (425 mg, 45%).

R$_f$ 0.28 (hexanes:EtOAc 3:2 (H_2SO_4/EtOH)).

[*α*]$_D^{22}$ +202.6 (CHCl$_3$, 0.0127).

^{1}H NMR (500 MHz, CDCl$_3$): δ 4.27 (d, *J* = 4.5 Hz, 1H, H-1), 3.80 (dd, *J* = 10.2, 3.5 Hz, 1H, H-6a), 3.62 (dd, *J* = 8.8, 4.3 Hz, 1H, H-2), 3.59 – 3.50 (m, 7H, 2×OCH$_3$, H-6b), 3.47

(s, 3H, OCH_3), 3.36 (s, 3H, OCH_3), 3.33 – 3.24 (m, 3H, H-3, H-4, H-5), 2.67 (qd, J = 10.3, 6.3 Hz, 2H, SC<u>H</u>$_2$CH$_3$), 1.25 (t, J = 7.3 Hz, 3H, SCH$_2$C<u>H</u>$_3$).

^{13}C NMR (126 MHz, $CDCl_3$): δ 85.8 (C-2), 85.5 (C-3), 83.9 (C-4), 70.6 (C-6), 61.5, 61.0, 59.1, 57.8 (4×OCH_3), 49.6 (C-1), 41.9 (C-5), 25.6 (S<u>C</u>H$_2$CH$_3$), 14.2 (SCH$_2$<u>C</u>H$_3$).

ESI-HRMS (m/z): $[M+Na]^+$ calcd. for $C_{12}H_{24}O_4NaS_2^+$ 319.1008, found 319.0997.

Ethyl 2,3,4,6-tetra-*O*-methyl-1,5-di-deoxy-1,5-dithio-*α*-D-glucopyranoside-1-*S*-Oxides (48):

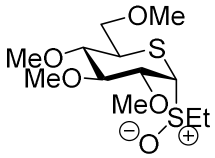

Thioglycoside **47** (246 mg, 0.830 mmol) was dissolved in anhydrous CH_2Cl_2 (10 mL) and the reaction mixture was cooled down to -60 °C before *m*CPBA (77% wt, 205 mg, 0.913 mmol, 1.1 equiv) solution in anhydrous CH_2Cl_2 (5 mL) was added. The reaction mixture then was stirred at -60 °C for 2h. After such time the reaction mixture was quenched with saturated aq $NaHCO_3$ (5 mL), allowed to warm up to 20 °C. The resulting emulsion was diluted with CH_2Cl_2 (50 mL) and the organic layer was separated, washed with saturated aq $NaHCO_3$ (50 mL), 1M $NaHSO_3$ (50 mL), brine (50 mL), dried over $MgSO_4$ and concentrated to dryness. The crude was purified by flash column chromatography on silica gel eluting with hexanes:acetone (0→80%, acetone) to give a complex mixture of (R_s),(S_s)-5-*S*-sulfoxides **49** (82 mg, 32%) as a colorless syrup and (R_s),(S_s)-1-*S*-sulfoxides **48** (81 mg, 31%) as a colorless syrup.

(R_s),(S_s)-1-*S*-sulfoxides 272:

d.r. = 4.2 : 1.

R_f 0.48 (hexanes : acetone 1 : 1 (H_2SO_4/EtOH)).

^{1}H NMR (500 MHz, CD_2Cl_2): Major diastereomer: δ 3.99 (dd, J = 6.0, 4.2 Hz, 1H, H-2), 3.84 (d, J = 4.1 Hz, 1H, H-1), 3.61 – 3.22 (m, 17H, H-5, H-4, H-3, H-6a, H-6b, 4×OCH_3), 2.98 – 2.83 (m, 1H, SC<u>H</u>$_2$CH$_3$), 2.75 – 2.64 (m, 1H, SC<u>H</u>$_2$CH$_3$), 1.28 (t, J = 7.5 Hz, 3H,

$SCH_2C\underline{H}_3$); **Minor diastereomer:** δ 3.87 (dd, *J* = 10.0, 4.7 Hz, 1H, H-2), 3.61 – 3.22 (m, 18H, H-1, H-3, H-4, H-5, H-6a, H-6b, 4×OCH_3), 2.98 – 2.83 (m, 1H, $SC\underline{H}_2CH_3$), 2.75 – 2.64 (m, 1H, $SC\underline{H}_2CH_3$), 1.35 (t, *J* = 7.4 Hz, 3H, $SCH_2C\underline{H}_3$).

^{13}C NMR (126 MHz, CD_2Cl_2): Major diastereomer: δ 83.0 (C-4), 76.8 (C-3), 72.5 (C-2), 66.4 (C-6), 60.2, 59.3, 58.5, 58.1 (4×OMe), 57.2 (C-1), 46.3 ($S\underline{C}H_2CH_3$), 42.5 (C-5), 6.69 ($SCH_2\underline{C}H_3$); **Minor diastereomer:** δ 83.1 (C-4), 82.6 (C-3), 80.3 (C-2), 71.5 (C-6), 59.5, 59.4, 59.1, 58.9 (4×OMe), 58.8 (C-1), 44.5 ($S\underline{C}H_2CH_3$), 42.5 (C-5), 6.7 ($SCH_2\underline{C}H_3$);

ESI-HRMS (m/z): $[M+Na]^+$ calcd. for $C_{12}H_{24}O_5NaS_2^+$ 335.0957, found 335.0932.

1.4. Summarized comparison of the key ^{13}C NMR peaks of 5-thioglucosyl endo- and exo-sulfoxides.

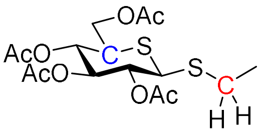

10β

δ(C-5) = 44.4 ppm
δ(SCH_2CH_3) = 24.9 ppm

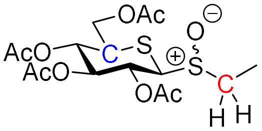

40

δ(C-5) = 43.2 and 42.7 ppm
δ(SCH_2CH_3) = 43.3 and 45.5 ppm

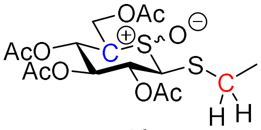

41

δ(C-5) = 64.2 ppm
δ(SCH_2CH_3) = 26.4 ppm

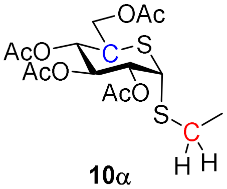

10α

δ(C-5) = 39.5 ppm
δ(SCH_2CH_3) = 25.9 ppm

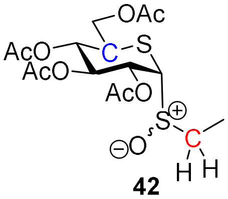

42

δ(C-5) = 41.0 ppm
δ(SCH_2CH_3) = 45.7 ppm

Armed sulfoxides

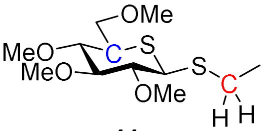

44

δ(C-5) = 47.0 ppm
δ(SCH_2CH_3) = 26.1 ppm

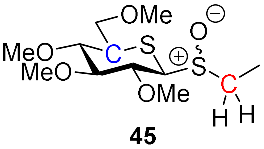

45

δ(C-5) = 44.4 and 45.6 ppm
δ(SCH_2CH_3) = 44.8 and 45.2 ppm

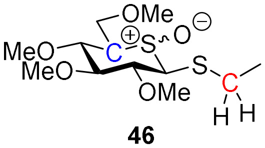

46

δ(C-5) = 61.0 ppm
δ(SCH_2CH_3) = 26.5 and 27.3 ppm

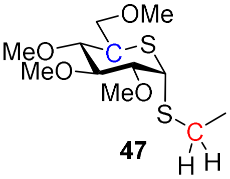

47

δ(C-5) = 41.9 ppm
δ(SCH_2CH_3) = 25.6 ppm

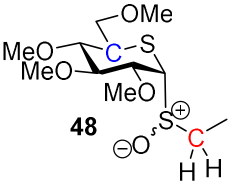

48

δ(C-5) = 42.5 ppm
δ(SCH_2CH_3) = 44.5 and 46.3 ppm

Figure S1. Comparison of the key ^{13}C NMR peaks of the starting materials and sulfoxides

1.5. Preparation of glucosyl sulfoxides

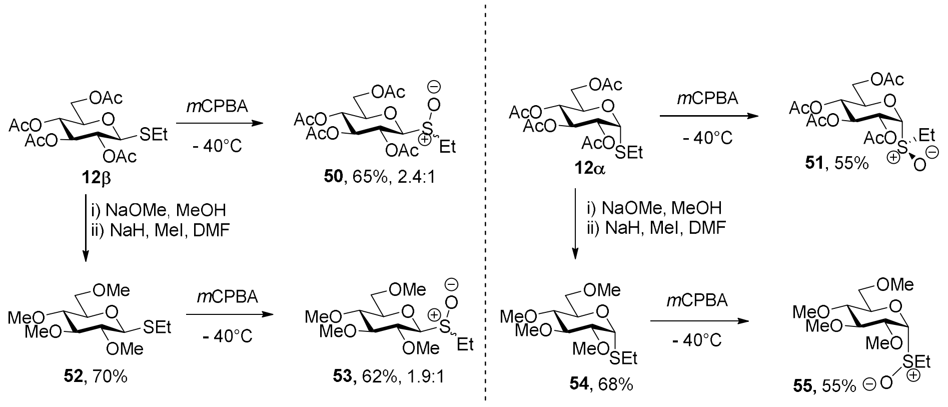

(*R*$_s$),(*S*$_s$)-Ethyl 2,3,4,6-tetra-*O*-acetyl-1-thio-*β*-D-glucopyranoside-*S*-Oxides (50):

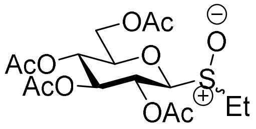

Thioglycoside **12β** (1 g, 2.55 mmol) was dissolved in anhydrous CH_2Cl_2 (10 mL) and the reaction mixture was cooled down to –40 °C before *m*CPBA (77% wt, 440 mg, 2.55 mmol, 1.1 equiv.) solution in anhydrous CH_2Cl_2 (10 mL) was added and the reaction mixture was stirred for 1h at –40 °C. After such time the reaction mixture was quenched with aq. sat. $NaHCO_3$ (10 mL) at –40 °C, and gradually warmed up to 20 °C. After that the reaction mixture was diluted with CH_2Cl_2 (60 mL), washed with aq. sat. $NaHCO_3$ (30 mL), 1M $NaHSO_3$ (30 mL), brine (50 mL), dried over $MgSO_4$ and concentrated to dryness. The crude product was purified by flash column chromatography on silica gel eluting with hexanes:EtOAc (50→100%, EtOAc) to give a mixture of (*R*$_s$),(*S*$_s$)-sulfoxides **50** as a white solid (674 mg, 65%) with spectral data identical to that reported in the literature.[4]

d.r. (**50*S*** : **50*R***) **=** 2.4:1.

R$_f$ 0.11 (hexanes:acetone 1:1 (H_2SO_4/EtOH));

50*S*:

^{1}H NMR (500 MHz, $CDCl_3$): δ 5.31 – 5.20 (m, 2H, H-3), 5.15 – 5.02 (m, 1H), 4.31 (d, *J* = 9.7 Hz, 1H), 4.26 (dd, *J* = 12.6, 4.7 Hz, 1H), 4.23 – 4.13 (m, 1H), 3.85 – 3.70 (m, 1H), 2.98 – 2.83 (m, 2H), 2.06 (s, 3H), 2.05 (s, 3H), 2.02 (s, 3H), 2.01 (s, 3H), 1.36 (t, *J* = 7.5 Hz, 3H).

^{13}C NMR (126 MHz, $CDCl_3$): δ 170.4, 170.0, 169.7, 169.3, 89.9, 76.9, 73.2, 68.4, 67.7, 61.4, 41.3, 20.6, 20.5, 20.5, 20.5, 6.5.

50*R*:

^{1}H NMR (500 MHz, $CDCl_3$): δ 5.43 (dd, *J* = 10.0, 9.3 Hz, 1H), 5.34 (t, *J* = 9.3 Hz, 1H), 5.15 – 5.02 (m, 1H), 4.26 (dd, *J* = 12.6, 4.7 Hz, 1H), 4.23 – 4.13 (m, 2H), 3.85 – 3.70 (m, 1H), 3.11 (dq, *J* = 12.8, 7.6 Hz, 1H), 2.83 – 2.71 (m, 1H), 2.06 (s, 3H), 2.05 (s, 3H), 2.02 (s, 3H), 2.01 (s, 3H), 1.32 (t, *J* = 7.6 Hz, 3H).

^{13}C NMR (126 MHz, $CDCl_3$): δ 170.5, 170.4, 169.7, 169.3, 86.6, 76.9, 73.8, 67.8, 66.9, 61.9, 41.2, 20.7, 20.6, 20.5, 20.5, 7.3.

ESI-HRMS (m/z): $[M+Na]^+$ calcd. for $C_{16}H_{24}O_{10}NaS^+$ 431.0982, found 431.0965.

(*R_S*)-Ethyl 2,3,4,6-tetra-*O*-acetyl-1-thio-*α*-D-glucopyranoside-*S*-Oxide (51):

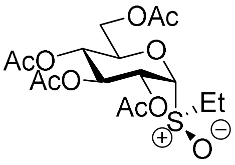

Thioglycoside **12α** (360 mg, 0.917 mmol) was dissolved in anhydrous CH_2Cl_2 (20 mL) and the reaction mixture was cooled down to –40 °C before *m*CPBA (77% wt, 226 mg, 1.0087 mmol, 1.1 equiv.) solution in anhydrous CH_2Cl_2 (3 mL) was added and the reaction mixture was stirred for 2h at –40 °C. After such time the reaction mixture was quenched with aq. sat. $NaHCO_3$ (10 mL) at –40 °C, and gradually warmed up to 20 °C. After that the reaction mixture was diluted with CH_2Cl_2 (40 mL), washed with aq. sat. $NaHCO_3$ (30 mL), 1M $NaHSO_3$ (30 mL), brine (30 mL), dried over $MgSO_4$ and concentrated to dryness. The crude product was purified by flash column chromatography on silica gel eluting with hexanes:EtOAc (0→100%, EtOAc) to give single diastereomer of sulfoxide **51** as a white solid (230 mg, 55%) with spectral data identical to that reported in the literature.[5]

R_f 0.44 (EtOAc (H_2SO_4/EtOH)).

^{1}H NMR (500 MHz, $CDCl_3$): δ 5.58 (t, *J* = 7.8 Hz, 1H, H-3), 5.34 (dd, *J* = 8.2, 5.2 Hz, 1H, H-2), 5.01 (dd, *J* = 9.4, 7.4 Hz, 1H, H-4), 4.80 (d, *J* = 5.1 Hz, 1H, H-1), 4.19 (dd, *J* = 12.4, 5.9 Hz, 1H, H-6a), 4.09 (dd, *J* = 12.4, 2.6 Hz, 1H, H-6b), 3.91 (ddd, *J* = 8.9, 5.9, 2.6 Hz, 1H, H-5), 2.94 (dq, *J* = 13.3, 7.5 Hz, 1H, $SC\underline{H}_2CH_3$), 2.77 (dq, *J* = 13.2, 7.4 Hz, 1H, $SC\underline{H}_2CH_3$), 2.11 (s, 3H, CH_3CO), 2.08 (s, 6H, 2×CH_3CO), 2.05 (s, 3H, CH_3CO), 1.39 (t, *J* = 7.5 Hz, 3H, $SCH_2C\underline{H}_3$);

^{13}C NMR (126 MHz, $CDCl_3$): δ 170.2, 169.9, 169.3, 169.3 (4×CO), 87.1 (C-1), 73.6 (C-5), 69.8 (C-3), 68.7 (C-2), 68.0 (C-4), 61.9 (C-6), 42.4 ($S\underline{C}H_2CH_3$), 20.6, 20.6, 20.5, 20.5 (4×CH_3CO), 5.6 ($SCH_2\underline{C}H_3$).

ESI-HRMS (m/z): $[M+Na]^+$ calcd. for $C_{16}H_{24}O_{10}NaS^+$ 431.0982, found 431.0960.

Ethyl 2,3,4,6-tetra-*O*-methyl-1-thio-*β*-D-glucopyranoside (52):

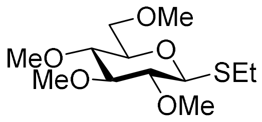

Thioglycoside **12β** (1.08 g, 2.75 mmol) was dissolved in anhydrous MeOH (60 mL) and NaOMe (59 mg, 1.1 mmol, 0.4 equiv) was added (pH 10) and the reaction mixture was stirred for 1h at 20 °C. After such time the reaction mixture was quenched with Amberlyst 15 (H^+) ion-exchange resin until pH 3. The resulting mixture was filtered, and the filtrate was concentrated to dryness. The crude intermediate was redissolved in anhydrous DMF (40 mL) and the resulting solution was cooled down to 0 °C before NaH (60% dispersion in mineral oil, 550 mg, 13.75 mmol, 5 equiv) was added followed by MeI (1.0 mL, 16.5 mmol, 6 equiv) and the reaction mixture was stirred with warming from 0 → 20 °C until completion (detected by LCMS and TLC analysis). After completion the reaction mixture was quenched with MeOH (until gas formation was no longer observed) at 0 °C, diluted with EtOAc (100 mL), washed with H_2O (80 mL), brine (80 mL), dried over $MgSO_4$ and concentrated to dryness. The crude product was purified by flash column chromatography on silica gel eluting with hexanes:EtOAc (0→50%, EtOAc) to give the product as a colorless oil (544 mg, 70%) with spectral data identical to that reported in the literature.[6]

R_f 0.48 (hexanes:EtOAc 7:3 (H_2SO_4/EtOH)).

^{1}H NMR (500 MHz, $CDCl_3$): δ 4.26 (dd, *J* = 9.8, 1.0 Hz, 1H), 3.66 – 3.55 (m, 7H), 3.56 – 3.49 (m, 4H), 3.36 (d, *J* = 1.0 Hz, 3H), 3.27 – 3.21 (m, 1H), 3.17 (t, *J* = 8.8 Hz, 1H), 3.10 (t, *J* = 9.3 Hz, 1H), 2.95 (dd, *J* = 9.7, 8.5 Hz, 1H), 2.76 – 2.65 (m, 2H), 1.27 (t, *J* = 7.4 Hz, 3H).

^{13}C NMR (126 MHz, $CDCl_3$): δ 88.5, 84.8, 83.4, 79.5, 78.8, 71.5, 60.9, 60.8, 60.4, 59.3, 24.9, 14.9.

ESI-HRMS (m/z): $[M+Na]^+$ calcd. for $C_{12}H_{24}O_5NaS^+$ 303.1237, found 303.1223.

(*R_S*),(*S_S*)-Ethyl 2,3,4,6-tetra-*O*-methyl-1-thio-*β*-D-glucopyranoside-1-*S*-Oxides (53):

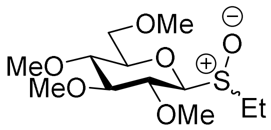

Thioglycoside **52** (190 mg, 0.678 mmol) was dissolved in anhydrous CH_2Cl_2 (10 mL) and the reaction mixture was cooled down to –60 °C before *m*CPBA (77% wt, 167 mg, 0.7458 mmol, 1.1. equiv) solution in anhydrous CH_2Cl_2 (5 mL) was added. The reaction mixture then was stirred at –60 °C for 1.5 h. After such time the reaction mixture was quenched with sat aq $NaHCO_3$ (5 mL) and allowed to warm up to 20 °C. The reaction mixture was then diluted with CH_2Cl_2 (40 mL), washed with sat aq $NaHCO_3$ (50 mL), 1M $NaHSO_3$ (30 mL), brine (50 mL), dried over $MgSO_4$ and concentrated to dryness. The crude product was purified by flash column chromatography on silica gel eluting with hexanes:EtOAc (0→100%, EtOAc) and EtOAc:MeOH (0→50%, MeOH) to give the product as a white solid (124 mg, 62%).

d.r. = 1.9 : 1.

R_f 0.37, 0.51 (EtOAc (H_2SO_4/EtOH)).

^{1}H NMR (500 MHz, CD_2Cl_2): Major isomer: δ 4.03 (d, *J* = 9.1 Hz, 1H, H-1), 3.64 – 3.42 (m, 11H, 3×OCH_3, H-2, H-6a), 3.37 – 3.24 (m, 6H, OCH_3, H-3, H-4, H-6b), 3.18 – 3.03 (m, 1H, H-5), 3.03 – 2.91 (m, 1H, $SC\underline{H}_2CH_3$), 2.80 – 2.61 (m, 1H, $SC\underline{H}_2CH_3$), 1.32 – 1.17 (m, 3H, $SCH_2C\underline{H}_3$); **Minor isomer:** δ 3.67 (d, *J* = 10.2 Hz, 1H, H-1), 3.64 – 3.42 (m, 11H,

3×OCH_3, H-2, H-6a), 3.37 – 3.24 (m, 6H, OCH_3, H-3, H-4, H-6b), 3.18 – 3.03 (m, 1H, H-5), 3.03 – 2.91 (m, 1H, $SC\underline{H}_2CH_3$), 2.80 – 2.61 (m, 1H, $SC\underline{H}_2CH_3$), 1.32 – 1.17 (m, 3H, $SCH_2C\underline{H}_3$).

^{13}C NMR (126 MHz, CD_2Cl_2): Major isomer: δ 91.8 (C-1), 88.5 (C-3), 79.3 (C-4), 79.0 (C-5), 77.5 (C-2), 71.3 (C-6), 60.6, 60.2, 59.9, 59.0 (4×OCH_3), 42.5 ($S\underline{C}H_2CH_3$), 7.4 ($SCH_2\underline{C}H_3$); **Minor isomer:** δ 88.9 (C-1), 88.5 (C-3), 79.8 (C-4), 79.0 (C-5), 77.3 (C-2), 71.1 (C-6), 60.7, 60.6, 60.4, 59.0 (4×OCH_3), 40.9 ($S\underline{C}H_2CH_3$), 7.2 ($SCH_2\underline{C}H_3$).

ESI-HRMS (m/z): $[M+Na]^+$ calcd. for $C_{12}H_{24}O_5NaS^+$ 319.1186, found 319.1174.

Ethyl 2,3,4,6-tetra-*O*-methyl-1-thio-*α*-D-glucopyranoside (54):

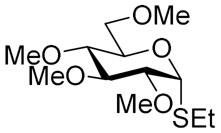

Thioglycoside **12α** (1.5 g, 3.82 mmol) was dissolved in anhydrous MeOH (30 mL) and NaOMe (83 mg, 1,53 mmol, 0.4 equiv) was added (pH 10) and the reaction mixture was stirred for 1h at 20 °C. After such time the reaction mixture was quenched with Amberlyst 15 (H^+) ion-exchange resin until pH 3. The resulting mixture was filtered, and the filtrate was concentrated to dryness. The crude intermediate was redissolved in anhydrous DMF (30 mL) and the resulting solution was cooled down to 0 °C before NaH (60% dispersion in mineral oil, 765 mg, 19.1 mmol, 5 equiv) was added followed by MeI (1.43 mL, 22.92 mmol, 6 equiv) and the reaction mixture was stirred at 0 → 20 °C until completion (detected by LCMS and TLC analysis). After completion the reaction mixture was quenched with MeOH (until gas formation was no longer observed) at 0 °C, diluted with EtOAc (100 mL), washed with H_2O (80 mL), brine (80 mL), dried over $MgSO_4$ and concentrated to dryness. The crude product was purified by flash column chromatography on silica gel eluting with hexanes:EtOAc (0→50%, EtOAc) to give the product as a yellowish syrup (732 mg, 68%) with spectral data identical to that reported in the literature.[7]

R_f 0.51 (hexanes:EtOAc 7:3 (H_2SO_4/EtOH)).

¹H NMR (500 MHz, CDCl$_3$): δ 5.47 (d, *J* = 5.4 Hz, 1H), 4.01 (ddd, *J* = 10.0, 3.7, 2.1 Hz, 1H), 3.70 – 3.30 (m, 18H), 3.20 (dd, *J* = 10.1, 8.8 Hz, 1H), 2.64 – 2.47 (m, 2H), 1.27 (t, *J* = 7.4 Hz, 3H).

¹³C NMR (126 MHz, CDCl$_3$): δ 84.0, 82.8, 81.6, 79.3, 71.1, 70.3, 61.0, 60.5, 59.3, 58.2, 23.9, 14.8.

ESI-HRMS (m/z): $[M+Na]^+$ calcd. for $C_{12}H_{24}O_5NaS^+$ 303.1237, found 303.1225.

Ethyl 2,3,4,6-tetra-*O*-methyl-1-thio-*α*-D-glucopyranoside-1-*S*-oxide (55):

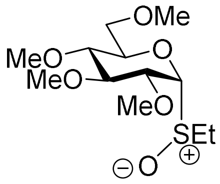

Thioglycoside **54** (548 mg, 1.95 mmol) was dissolved in anhydrous CH_2Cl_2 (20 mL) and the reaction mixture was cooled down to –50 °C and *m*CPBA (77% wt, 480 mg, 2.145 mmol, 1.1 equiv) solution in anhydrous CH_2Cl_2 (10 mL) was added and the reaction mixture was stirred for 1.5h at –50 °C. After such time sat aq $NaHCO_3$ (10 mL) was added to quench the reaction, and the reaction mixture was allowed to warm up to 20 °C. After that the reaction mixture was diluted with CH_2Cl_2 (30 mL), washed with $NaHCO_3$ (60 mL), 1M $NaHSO_3$ (60 mL), brine (60 mL), dried over $MgSO_4$ and concentrated to dryness. The crude product was purified by flash column chromatography on silica gel eluting with hexanes:EtOAc (0→100%, EtOAc) to give the product as a colorless syrup (316 mg, 55%).

R$_f$ 0.57 (EtOAc (H_2SO_4/EtOH)).

[*α*]$_D^{20}$ +101.7 ($CHCl_3$, 0.0047).

¹H NMR (500 MHz, CDCl$_3$): δ 4.92 (d, *J* = 6.1 Hz, 1H, H-1), 4.16 (ddd, *J* = 10.0, 4.4, 2.1 Hz, 1H, H-5), 3.96 (t, *J* = 8.1 Hz, 1H, H-3), 3.78 – 3.71 (m, 1H, H-2), 3.68 – 3.48 (m, 11H, H-6a, H-6b, 3×OCH_3), 3.38 (s, 3H, OCH_3), 3.23 – 3.08 (m, 2H, H-4, $SC\underline{H}_2CH_3$), 3.08 – 2.94 (m, 1H, $SC\underline{H}_2CH_3$), 1.50 – 1.32 (t, *J* = 7.5 Hz, 3H, $SCH_2C\underline{H}_3$).

^{13}C NMR (126 MHz, $CDCl_3$): δ 86.5 (C-1), 81.6 (C-3), 80.4 (C-2), 78.2 (C-4), 74.9 (C-5), 70.9 (C-6), 60.4, 59.9, 59.7, 59.2 (4×OCH_3), 46.6 (S<u>C</u>H_2CH_3), 5.9 (SCH_2<u>C</u>H_3).

ESI-HRMS (m/z): $[M+Na]^+$ calcd. for $C_{12}H_{24}O_5NaS^+$ 319.1186, found 319.1175.

1.6. Preparation glucosyl trichloroacetimidates

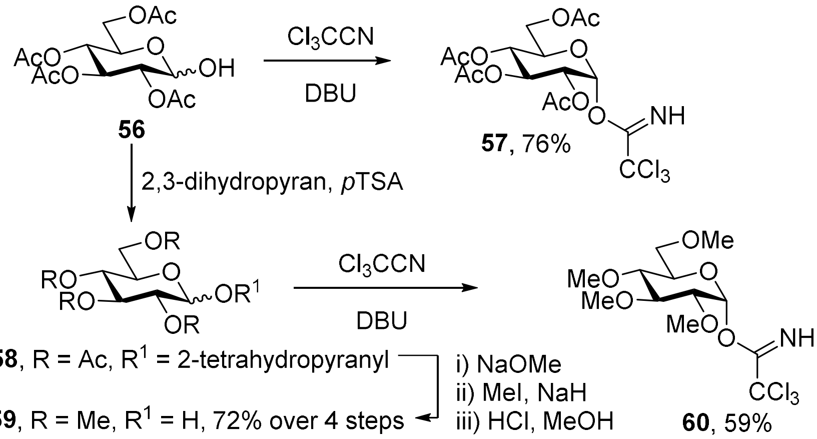

2,3,4,6-tetra-*O*-acetyl-*α*-D-glucopyranosyl trichloroacetimidate (57):

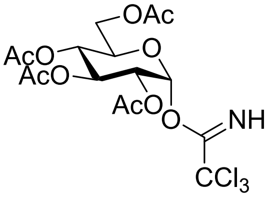

2,3,4,6-Tetra-*O*-acetyl-D-glucopyranose **56** (105 mg, 0.301 mmol) was dissolved in anhydrous CH_2Cl_2 (5 mL) and CCl_3CN (150 µL, 1.505 mmol, 5 equiv) was added followed by DBU (5 µL, 0.0334 mmol, 0.11 equiv) and the reaction mixture was stirred for 1h at 20 °C. After such time the reaction mixture was diluted with CH_2Cl_2 (30 mL), washed with 1M HCl (20 mL), saturated aqueous $NaHCO_3$ (50 mL), brine (50 mL), dried over $MgSO_4$ and concentrated to dryness. The crude product was purified by a column chromatography eluting with hexanes:EtOAc (10→50%, EtOAc) to give the product as a colorless syrup (112 mg, 76%) with spectra data identical to that reported in the literature.[8]

R_f 0.60 (hexanes:EtOAc 1:1 (H_2SO_4/EtOH)).

^{1}H NMR (500 MHz, $CDCl_3$): δ 8.68 (s, 1H), 6.55 (d, *J* = 3.7 Hz, 1H), 5.56 (t, *J* = 9.9 Hz, 1H), 5.17 (t, *J* = 9.9 Hz, 1H), 5.12 (dd, *J* = 10.2, 3.6 Hz, 1H), 4.32 – 4.17 (m, 2H), 4.15 – 4.07 (m, 1H), 2.07 (s, 3H), 2.04 (s., 3H), 2.02 (s, 3H), 2.01 (s, 3H).

^{13}C NMR (126 MHz, $CDCl_3$): δ 170.7, 170.1, 169.9, 169.6, 160.9, 93.0, 70.1, 70.0, 69.8, 67.9, 61.5, 20.7, 20.5.

ESI-HRMS (m/z): $[M+Na]^+$ calcd. for $C_{16}H_{20}O_{10}NCl_3Na^+$ 514.0045, found 514.0020.

2,3,4,6-tetra-*O*-methyl-*α,β*-D-glucopyranose (59):

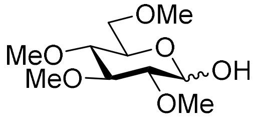

2,3,4,6-Tetra-*O*-acetyl-D-glucopyranose **56** (2.06 g, 5.91 mmol) was dissolved in anhydrous THF (20 mL) and 3,4-dihydropyran (1.6 mL, 17.73 mmol, 3 equiv) was added followed by PTSA (112 mg, 0.591 mmol, 0.1 equiv) and the reaction mixture was stirred until completion (detected by LCMS and TLC). After completion, the reaction mixture was quenched with saturated aqueous $NaHCO_3$ (10 mL), and diluted with EtOAc (60 mL). The organic layer was separated, washed with brine (60 mL), dried over $MgSO_4$ and concentrated to dryness. The crude was used in the next step without further purification. Thus, 2-tetrahydropyranyl 2,3,4,6-tetra-*O*-acetyl-D-glucopyranoside **58** (2.28 g, 5.25 mmol) was suspended in anhydrous MeOH (40 mL) and NaOMe (1M solution in MeOH, 1.58 mL, 1.58 mmol, 0.3 equiv) and the reaction mixture was stirred for 1h at 20 °C. After such time the reaction mixture was quenched with Amberlite IR-120 (H^+) until pH 6. The resulting mixture was filtered, and the filtrate was concentrated to dryness. The crude was taken up in DMF (30 mL) and NaH (60% dispersion in mineral oil, 1.15 g, 31.5 mmol, 6 equiv) was added at 0 °C and the reaction mixture was stirred for 10 minutes before MeI (2 mL, 32.1 mmol, 6.1 equiv) was added. The reaction mixture then was stirred at 0→20 °C for 16h. After such time the reaction mixture was quenched with MeOH (10 mL) at 0 °C and the resulting solution was distributed between EtOAc (100 mL) and H_2O (100 mL). The organic layer was collected, and the aqueous layer was extracted with EtOAc (50 mL). The combined organic layers were washed with brine (60 mL), dried over $MgSO_4$ and concentrated to dryness and co-evaporated with toluene (3×10 mL). The crude was taken into MeOH (20 mL) and cat HCl (37% aq. solution) was added, and the reaction mixture was stirred for 1h at 20 °C. After such time the reaction mixture was concentrated to dryness. The crude product was purified by column chromatography eluting with hexanes:EtOAc (10→60%, EtOAc) to give a mixture of anomeric hemiacetals as a white

solid (1.01 g, 72% over 4 steps) with spectral data identical to those reported in the literature.[9]

α : β = 2.3 : 1

R_f 0.22 (hexanes:EtOAc 1:1 (H_2SO_4/EtOH)).

^{1}H NMR (500 MHz, $CDCl_3$): δ 5.31 (d, *J* = 3.6 Hz, 1H), 4.56 (d, *J* = 7.7 Hz, 1H), 3.88 (dt, *J* = 10.2, 3.4 Hz, 1H), 3.68 – 3.46 (m, 18H), 3.39 – 3.35 (m, 5H), 3.35 (ddd, *J* = 9.8, 5.7, 1.9 Hz, 1H), 3.22 – 3.03 (m, 3H), 2.95 (dd, *J* = 9.0, 7.7 Hz, 1H).

^{13}C NMR (126 MHz, $CDCl_3$): δ 97.2, 90.7, 86.5, 84.9, 83.2, 82.0, 79.8, 79.7, 74.4, 71.7, 71.4, 69.9, 60.9, 60.8, 60.6, 60.5, 60.5, 59.2, 59.2, 58.9.

ESI-HRMS (m/z): $[M+Na]^+$ calcd. for $C_{16}H_{24}O_{10}NaS^+$ 259.1152, found 259.1140.

2,3,4,6-tetra-*O*-methyl-*α,β*-D-glucopyranosyl trichloroacetimidate (60):

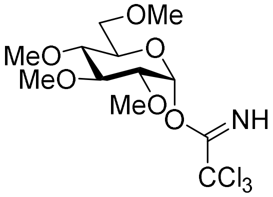

Hemiacetal **59** (85 mg, 0.36 mmol) was dissolved in anhydrous CH_2Cl_2 (6 mL) and the reaction mixture was cooled down to 0 °C before CCl_3CN (360 μL, 3.6 mmol, 10 equiv) was added followed by DBU (5 μL, 0.037 mmol, 0.1 equiv). The reaction mixture then was stirred for 1h at 20 °C. After such time the reaction mixture was concentrated to dryness and the crude product was purified by flash column chromatography on silica gel eluting with hexanes:EtOAc (0→40%, EtOAc) to give a mixture of anomeric imidates as a colorless syrup (80 mg, 59%) with spectral data matching that reported in the literature.[10]

α* : *β = 2 : 1

R_f 0.57, 0.51 (hexanes:EtOAc 1:1 (H_2SO_4/EtOH)).

60*α*:

^{1}H NMR (500 MHz, CDCl$_3$): δ 8.58 (s, 1H, NH), 6.47 (d, *J* = 3.5 Hz, 1H, H-1*α*), 3.80 (dt, *J* = 10.4, 2.7 Hz, 1H, H-5*α*), 3.67 – 3.50 (m, 9H, H-6a, H-6b, H-4*α*, 2×OCH$_3$), 3.47 (s, 3H, OCH$_3$), 3.38 (s, 3H, OCH$_3$), 3.37 – 3.22 (m, 5H).

^{13}C NMR (126 MHz, CDCl$_3$): δ 161.3 (C=NH), 94.0 (C-1), 91.3 (CCl$_3$), 83.0 (C-4), 81.1 (C-2), 78.6 (C-3), 72.8 (C-5), 70.6 (C-6), 61.0, 60.6, 59.2, 58.8 (4×OCH$_3$).

60*β*:

^{1}H NMR (500 MHz, CDCl$_3$): δ 8.63 (s, 1H, NH), 5.59 (d, *J* = 7.3 Hz, 1H, H-1), 3.67 – 3.50 (m, 12H, H-6a, H-6b, H-4, 3×OCH$_3$), 3.46 – 3.39 (m, 1H, H-5), 3.38 (s, 3H, OCH$_3$), 3.37 – 3.22 (m, 2H, H-3, H-2).

^{13}C NMR (126 MHz, CDCl$_3$): 161.4 (C=NH), 98.3 (C-1), 91.0 (CCl$_3$), 86.4 (C-4), 82.9 (C-2), 78.8 (C-3), 75.6 (C-5), 70.8 (C-6), 61.0, 60.8, 59.4 (4×OCH$_3$).

ESI-HRMS (m/z): [M+Na]$^+$calcd. for $C_{12}H_{20}O_6NCl_3Na^+$ 402.0248, found 402.0229.

1.7. Preparation 5-thioglucosyl trichloroacetimidates

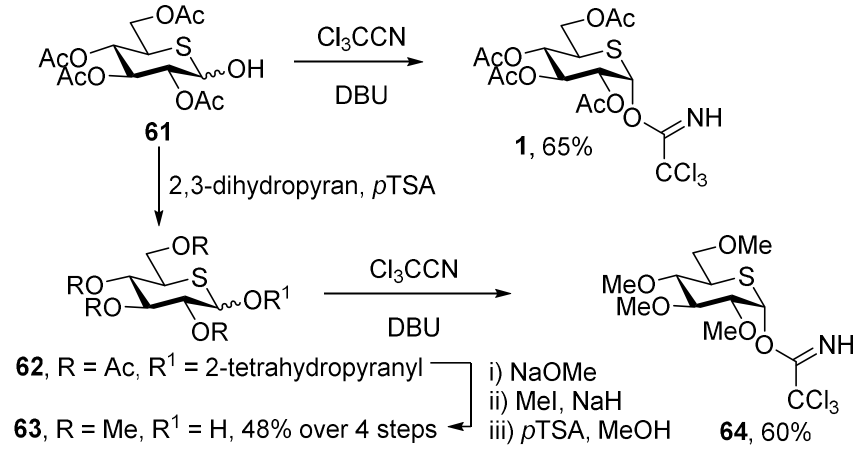

2,3,4,6-tetra-*O*-acetyl-5-thio-*α,β*-D-glucopyranosyl trichloroacetimidate (1):

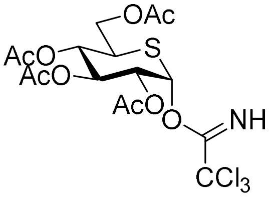

2,3,4,6-Tetra-*O*-acetyl-5-thio-5-deoxy-D-glucopyranose **61** (99 mg, 0.271 mmol) was dissolved in anhydrous CH_2Cl_2 (10 mL) and the reaction mixture was cooled down to 0 °C and CCl_3CN (270 µL, 2.69 mmol, 10 equiv) was added followed by DBU (4 µL, 0.0271 mmol, 0.11 equiv) and the reaction mixture was stirred for 2h at 20 °C. After such time the reaction mixture was diluted with CH_2Cl_2 (20 mL), washed with 1M HCl (10 mL), saturated aqueous $NaHCO_3$ (20 mL), dried over $MgSO_4$ and concentrated to dryness. The crude product was purified by a column chromatography eluting with hexanes:EtOAc (0→40%, EtOAc) to give a mixture of anomeric imidates as a colorless syrup (89 mg, 65%) with spectral data identical to that reported in the literature.[11]

α* : *β = 17 : 1

R_f 0.52 (hexanes:EtOAc 1:1 (H_2SO_4/EtOH)).

^{1}H NMR (500 MHz, $CDCl_3$): δ 8.71 (s, 1H), 6.36 (d, *J* = 3.2 Hz, 1H), 5.57 (t, *J* = 9.9 Hz, 1H), 5.38 (dd, *J* = 10.9, 9.6 Hz, 1H), 5.31 (dd, *J* = 10.2, 3.1 Hz, 1H), 4.39 (dd, *J* = 12.2, 4.9 Hz, 1H), 4.08 (dd, *J* = 12.1, 3.0 Hz, 1H), 3.64 (ddd, *J* = 10.8, 4.9, 3.1 Hz, 1H), 2.07 (s, 3H), 2.06 (s, 3H), 2.03 (s, 3H), 2.00 (s, 3H).

^{13}C NMR (126 MHz, CDCl$_3$): δ 170.5, 169.8, 169.6, 169.6, 160.8, 75.9, 73.6, 71.7, 70.7, 60.9, 40.1, 20.7, 20.6, 20.6.

ESI-HRMS (m/z): $[M+Na]^+$ calcd. for $C_{16}H_{20}O_9NCl_3NaS^+$ 529.9817, found 529.9804.

2,3,4,6-tetra-*O*-methyl-5-thio-*α*,*β*-D-glucopyranoside (63):

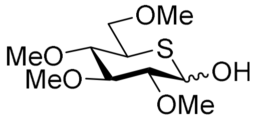

Hemiacetal **61** (1.2 g, 3.29 mmol) was dissolved in anhydrous THF (20 mL) and 3,4-dihydropyran (0.9 mL, 9.87 mmol, 3 equiv) was added followed by PTSA (63 mg, 0.329 mmol, 0.1 equiv) and the reaction mixture was stirred for 1h at 20 °C. After 1h additional PTSA (63 mg, 0.329 mmol, 0.1 equiv) was added followed by 3,4-dihydropyran (0.5 mL, 3.29 mmol, 1 equiv) and the reaction mixture was stirred for 1h. After such time the reaction mixture was quenched with saturated aqueous $NaHCO_3$ (10 mL), and diluted with EtOAc (60 mL). The organic layer was separated, washed with brine (60 mL), dried over $MgSO_4$ and concentrated to dryness. The crude was used in the next step without further purification. Accordingly, tetrahydropyranyl glycoside **62** (820 mg, 1.828 mmol) was suspended in anhydrous MeOH (15 mL) and NaOMe (29 mg, 0.548 mmol, 0.3 equiv) was added and the reaction mixture was stirred for 1h at 20 °C. After such time the reaction mixture was quenched with Amberlite IRC120 (H^+) (portion-wise addition until pH 5-6). After that the reaction mixture was filtered and the filtrate was concentrated to dryness. The crude product was taken up in anhydrous DMF (15 mL) and the reaction mixture was cooled down to 0 °C before NaH (60% dispersion in mineral oil, 474 mg, 11.85 mmol, 6.5 equiv) was added and the reaction mixture was stirred for 10 minutes at 0 °C. After such time MeI (682 µL, 10.97 mmol, 6 equiv) was added and the reaction mixture was stirred until completion (monitored by TLC and LCMS). After 3h the reaction mixture was quenched with MeOH (10 mL) at 0 °C, and the resulting mixture was distributed between EtOAc (100 mL) and H_2O (50 mL). The organic layer was collected, and the aqueous layer was additionally extracted with EtOAc (50 mL). The combined organic layers were washed with brine (50 mL), dried over $MgSO_4$ and concentrated to

dryness and co-evaporated with toluene (3×10 mL). The crude residue was taken into MeOH (20 mL) followed by addition of PTSA (35 mg, 0.1828 mmol, 0.1 equiv) and the reaction mixture was stirred for 1h at 45 °C. After such time the reaction mixture was allowed to cool down to 20 °C, and subsequently quenched with saturated aq $NaHCO_3$ (5 mL). After that the reaction mixture was concentrated to dryness, redissolved in EtOAc (40 mL), washed with saturated aqueous $NaHCO_3$ (20 mL), brine (20 mL), dried over $MgSO_4$ and concentrated to dryness. The crude was purified by column chromatography on a silica gel eluting with hexanes:EtOAc (0→40%, EtOAc) to give a mixture of anomeric hemiacetals as a colorless syrup (395 mg, 48% over 4 steps).

α* : *β = 16:1

R_f 0.21 (hexanes:EtOAc 1:1 (H_2SO_4/EtOH)).

^{1}H NMR (500 MHz, CD_2Cl_2): δ 4.97 (d, *J* = 3.4 Hz, 1H, H-1), 3.72 (dd, *J* = 9.9, 5.0 Hz, 1H, H-6a), 3.56 (s, 3H, OCH_3), 3.54 – 3.51 (m, 4H, H-6b, OCH_3), 3.47 (s, 3H, OCH_3), 3.38 – 3.27 (m, 7H, OCH_3, H-2, H-3, H-4), 3.23 – 3.17 (m, 1H, H-5), 2.75 (br, 1H, OH).

^{13}C NMR (126 MHz, CD_2Cl_2): δ 86.7 (C-2), 84.9 (C-3), 84.0 (C-4), 71.1 (C-1), 70.9 (C-6), 61.4, 61.0, 59.1, 58.3 (4×OCH_3), 41.4 (C-5); The minor *β*-isomer was identified in mixture by the following diagnostic signal: 4.61 (dd, *J* = 7.3, 5.0 Hz, 1H, H-1).

ESI-HRMS (m/z): $[M+Na]^+$ calcd. for $C_{10}H_{20}O_5NaS^+$ 275.0924, found 275.0918.

2,3,4,6-tetra-*O*-methyl-5-thio-*α*-D-glucopyranosyl trichloroacetimidate (64):

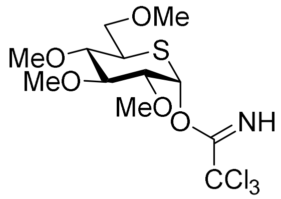

Hemiacetal **63** (87 mg, 0.345 mmol) was dissolved in anhydrous CH_2Cl_2 (6 mL) and the reaction mixture was cooled down to 0 °C before CCl_3CN (350 μL, 3.45 mmol, 10 equiv) was added followed by DBU (5 μL, 0.0345 mmol, 0.1 equiv). The reaction mixture then was stirred at 20 °C for 1h. After such time the reaction mixture was concentrated to

dryness, and the crude product was purified by flash column chromatography eluting with hexanes:EtOAc (0→40%, EtOAc) to give the product as a colorless syrup (82 mg, 60%).[1]

***R*$_f$** 0.53 (hexanes:EtOAc 3:2 (H_2SO_4/EtOH)).

[*α*]$_D^{20}$ +141.5 ($CHCl_3$, 0.031).

^{1}H NMR (500 MHz, $CDCl_3$): δ 8.61 (s, 1H, NH), 6.26 (d, *J* = 3.0 Hz, 1H, H-1), 3.81 (dd, *J* = 10.0, 4.5 Hz, 1H, H-6a), 3.63 (s, 3H, OCH_3), 3.61 (s, 3H, OCH_3), 3.57 – 3.40 (m, 7H, H-6b, H-2, H-4, H-3, OCH_3), 3.36 (s, 3H, OCH_3), 3.24 (ddd, *J* = 10.2, 4.6, 2.7 Hz, 1H, H-5).

^{13}C NMR (126 MHz, $CDCl_3$): δ 161.1 (C=NH), 91.3 (CCl_3), 85.3 (C-2), 84.8 (C-3), 83.3 (C-4), 75.9 (C-5), 70.1 (C-6), 61.6, 61.2, 59.1, 58.3 (4×OCH_3), 42.6 (C-5).

ESI-HRMS (m/z): $[M+Na]^+$ calcd. for $C_{12}H_{20}O_5NCl_3NaS^+$ 418.0020, found 418.0003.

[1] Imidate slowly undergoing rearrangement into the trichloroacetamide.

2. Computational Methods

2.1. NMR chemical shift calculations

NMR chemical shift calculations were performed for the thienium ion **35** and TMS at BP86/6-311+G(2d,p)[12-17] with an optimized geometry at the same level with SMD[18] in dichloromethane (DCM) using QCHEM.[19] The chemical shift (δ_c) of the sp^2-hybridized carbon of the thienium ion **35** was computed relative to that of tetramethylsilane (TMS) (δ_{TMS}) (Eq 1 and Eq 2), where δ and σ are chemical shift and isotropic shifts, respectively,[20, 21] and was found to be 246.04 ppm.

$$\sigma_{TMS} - \sigma_c + \delta_{TMS} = \delta_c \qquad \text{Eq 1}$$

$$181.92 - (-64.52) + 0 = 246.04 \qquad \text{Eq 2}$$

2.2. Transition state energies calculations

Tetrahydropyranyl triflate **39** and tetrahydrothiopyranyl **38** triflate were studied in three positions: covalently bound, the contact ion pair (CIP), and the transition state (TS). To compute the accurate Gibbs Free enthalpies (ΔG), a series of calculations were performed in gaseous and solvated phases. All structures were optimized using Orca. [22, 23]

2.3. Explicit calculations

For gas phase calculations, the studied structures were computed at M06-2X with an aug-cc-pvtz basis set and cc-pvtz basis set on H, C, and S.[24-27] Four dichloromethane molecules were added to all analyzed structures to stabilize the transition state (TS) and contact ion pair (CIP) intermediates. Vibrational frequencies were calculated using analytic gradients. All minima have zero imaginary modes, and the transition states have one. The calculations of δG were performed at STP using Grimme's QRRHO correction for vibrational modes lower than 150 cm^{-1}.[28]

2.4. Implicit calculations

For solvent calculations, the studied structures were optimized using SMD with dichloromethane as a solvent at M06-2x/6-31G*.[14, 29-32] Frequencies were calculated using analytic gradients with the same corrections for low vibrational modes.

2.5. Gibbs Free Energy calculations

The Gibbs free enthalpy was calculated using Grimme's scheme (Eq 3) where ΔG is the summation of the relative difference of the electronic energies with zero-point vibrational correction (ZPVE) (δE), Gibbs free enthalpy in the gas phase (δG_g), and Gibbs free enthalpy in solution (δG_s).

$$\Delta G = \delta E + \delta G_g + \delta G_s \qquad \text{Eq 3}$$

2.6. Cartesian coordinates of computed structres

Computational results are provided in Tables S1-S12. All geometries are in Cartesian Coordinates in Å calculated. Explicit structures are computed at the M06-2x with an aug-cc-pvtz basis with an cc-pvtz for H,C, and S. The six structures optimized in SMD with DCM as the solvent were computed at M06-2x with a 6-31G* basis set

Table S1. Cartesian Coordinates for tetrahydropyranyl triflate (**39_{solv}**):

C	-2.442275616838	1.278239636503	1.184825542657
C	-3.688442408857	0.490619191919	1.572148505537
C	-4.448582131314	0.114364019889	0.303074090900
C	-3.532348876845	-0.650467679678	-0.631593322651
C	-1.638613562161	0.549800657275	0.134347468565
H	-1.797871330620	1.495007755324	2.035740962784
O	-2.337597949352	0.091306097231	-0.934369074473
F	1.445730643994	-1.193099588185	3.553769547543
F	-0.360103861559	-0.009299615990	3.550682049406
F	-0.469715104299	-2.141784525137	3.247967855193
H	-4.310266047940	1.081979619699	2.242275021928
H	-3.400661167545	-0.416026065958	2.110615733082
H	-2.718380949328	2.236099007692	0.737872528618
H	-3.246227008588	-1.607287983993	-0.185649149236
H	-0.794864818730	1.127724348417	-0.229171908298
H	-4.806401370420	1.016814941913	-0.197738148941
H	-3.989651451459	-0.843211111020	-1.597783476043
H	-5.315990617075	-0.505307058844	0.530420133619
O	-1.049423423287	-0.685917296879	0.805381437799
C	0.245569997446	-1.059347895960	3.016654424912
S	0.446045005879	-0.803956849004	1.196915267682
O	1.135805988695	0.449672882801	1.028485031437
O	1.002181443823	-2.021423895567	0.685074149134

CL	-2.025177806431	4.841055601110	-0.452136273873
CL	0.324922060541	3.553450072792	0.760708119576
H	0.209873334161	4.908225601436	-1.147231413475
H	-0.066384721361	5.833822034750	0.370265978397
C	-0.278003177281	4.927766786015	-0.181616543889
Cl	-0.185495070286	2.456513345156	-2.628676669609
Cl	0.888939988278	-0.251418530897	-2.252603861950
C	1.211300521997	1.488144229627	-2.118678170565
H	1.422762406585	1.707092237745	-1.078592641072
H	2.046888634421	1.722117249379	-2.764040671093
Cl	5.248312951754	-1.859674166952	-0.268344423923
Cl	4.237925416203	0.850113535437	-0.776944116217
C	3.892141949468	-0.742402711058	-0.069456468183
H	3.711546393412	-0.601839624967	0.987847630663
H	3.026284239239	-1.151614056483	-0.572017474380
Cl	-1.770527630003	-3.937526776154	0.211417033167
Cl	-2.213681107242	-3.506222312124	-2.661860106460
C	-1.156820093595	-3.190259794582	-1.274286667432
H	-1.117762083887	-2.118971084129	-1.125989831691
H	-0.179592989104	-3.606758465533	-1.479471768131

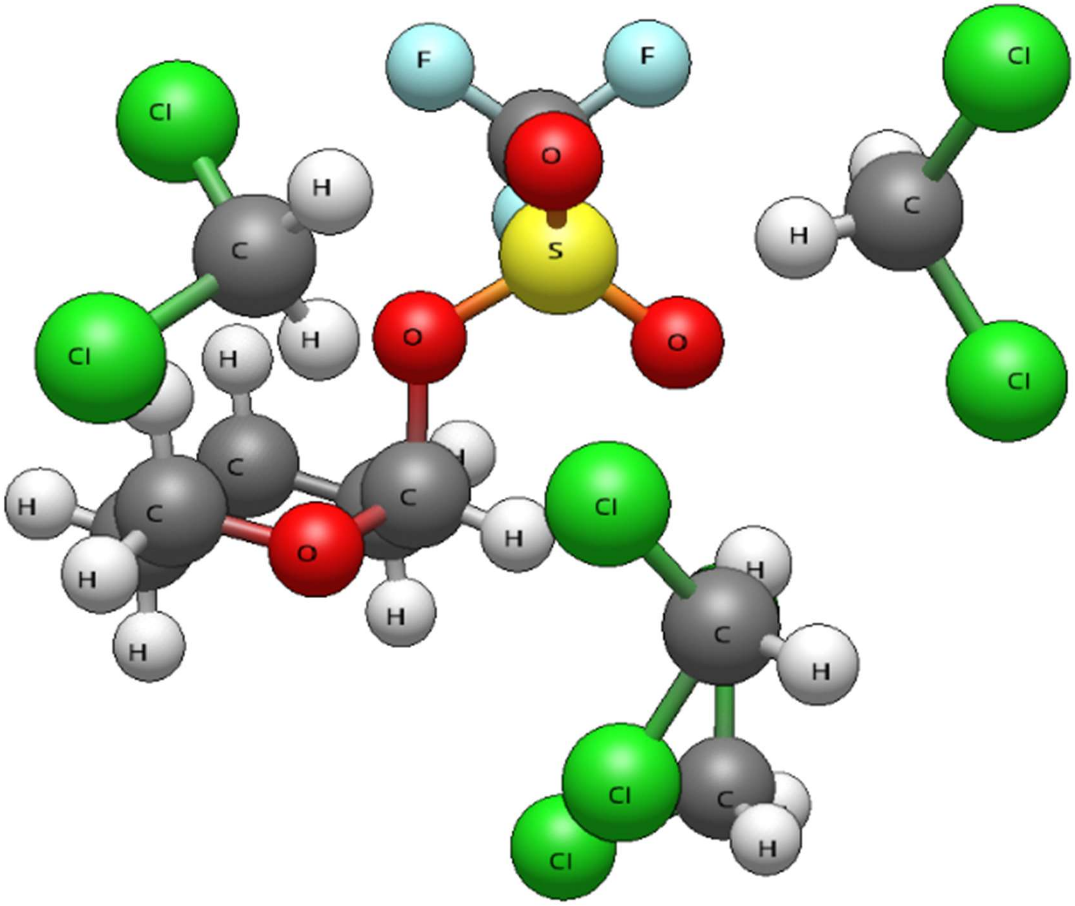

Figure S2. Structure of solvated tetrahydropyranyl triflate (**39**$_{solv}$)

Table S2. Cartesian Coordinates for tetrahydropyranyl triflate in transition state (**95**$^{‡}_{solv}$):

C	-2.347390	1.251841	1.129756
C	-3.527708	0.396343	1.562824
C	-4.490088	0.262755	0.386201
C	-3.769941	-0.338466	-0.797645
C	-1.826015	0.870139	-0.200287
H	-1.517114	1.263312	1.831302

O	-2.541213	0.383161	-1.124631
F	1.463882	-1.226786	3.328862
F	-0.519285	-0.374773	3.319453
F	-0.247796	-2.475276	2.915198
H	-4.019206	0.847742	2.421900
H	-3.163086	-0.588043	1.860048
H	-2.644953	2.301135	0.992018
H	-3.439616	-1.357659	-0.600416
H	-0.882570	1.279846	-0.546949
H	-4.901012	1.238589	0.117534
H	-4.344815	-0.308753	-1.717262
H	-5.327986	-0.389465	0.631120
O	-0.958172	-0.955858	0.470148
C	0.279033	-1.271874	2.737417
S	0.449911	-0.908602	0.935180
O	1.013160	0.433959	0.888479
O	1.267345	-1.993610	0.438236
Cl	-2.010656	4.600398	-0.612012
Cl	0.355862	3.467424	0.714632
H	0.256058	5.014595	-1.040577
H	-0.288337	5.718729	0.523630
C	-0.318249	4.862007	-0.136269
Cl	0.215518	2.700998	-2.765691
Cl	0.779127	-0.158664	-2.458512
C	1.384226	1.481086	-2.195675
H	1.527306	1.622221	-1.129503
H	2.302611	1.611062	-2.752678
Cl	5.298068	-1.298356	-0.261299
Cl	4.253642	1.445191	-0.222362
C	3.889602	-0.274996	0.053543
H	3.595759	-0.391643	1.087497
H	3.091167	-0.571041	-0.614215
Cl	-2.702407	-3.835869	0.416744
Cl	-1.616861	-2.941062	-2.167096
C	-1.298653	-3.840593	-0.673182
H	-0.468265	-3.366121	-0.167012
H	-1.095517	-4.868914	-0.940213

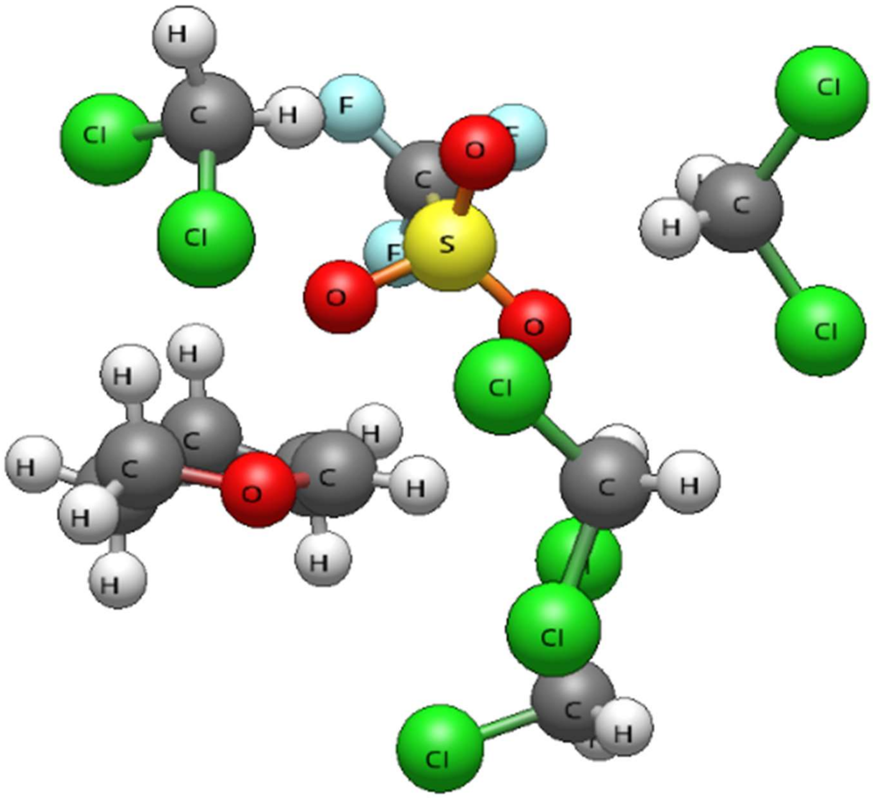

Figure S3. Structure of solvated tetrahydropyranyl triflate in transition state (**95**$^{‡}_{\mathbf{solv}}$)

Table S3. Cartesian Coordinates for oxocarbenium triflate in SSIP form (**37**$_{\mathbf{SSIP}}$):

C	3.23437	-1.41966	1.41622
C	2.79391	-2.75188	0.816823
C	1.304695	-2.94152	1.071562
C	0.518901	-1.70227	0.952824
C	2.452085	-0.30122	0.779904
H	2.563477	0.665807	1.259898

O	1.004448	-0.55594	0.867308
H	3.350418	-3.58154	1.245932
H	1.101335	-3.27807	2.096919
H	4.291996	-1.23398	1.23508
H	0.829038	-3.67924	0.42248
H	2.977826	-2.74793	-0.25902
H	3.072391	-1.4075	2.496696
H	-0.56982	-1.70225	0.96119
H	2.64359	-0.20316	-0.28814
F	-2.55689	3.145979	-1.0807
F	-3.59928	1.260476	-1.221
S	-1.35211	1.149753	0.117195
F	-3.61941	2.37083	0.629247
O	-0.67015	0.858996	-1.14322
O	-1.87529	-0.03219	0.807011
O	-0.66371	2.09679	0.984979
C	-2.87535	2.0401	-0.41821
H	-1.94325	-1.1962	-1.45589
Cl	-3.21691	-2.38967	-3.0314
C	-2.76076	-1.90233	-1.39537
H	-3.62048	-1.48074	-0.89393
Cl	-2.21475	-3.30349	-0.43151
Cl	3.09504	-0.89375	-2.91305
C	1.329484	-0.73879	-2.84436
H	0.954308	-0.67615	-3.85695
H	1.0615	0.127285	-2.24983
Cl	0.583023	-2.16391	-2.08509
Cl	2.635906	3.486457	1.06003
C	1.807277	3.390404	-0.50421
Cl	2.679868	2.33762	-1.64145
H	1.778163	4.383843	-0.93053
H	0.824748	2.969523	-0.3328
Cl	-0.46487	-1.399	3.868568
Cl	1.376557	0.885941	3.846908
H	-0.7079	0.679382	2.774517
H	-0.85461	0.805055	4.567299
C	-0.31488	0.372518	3.736078

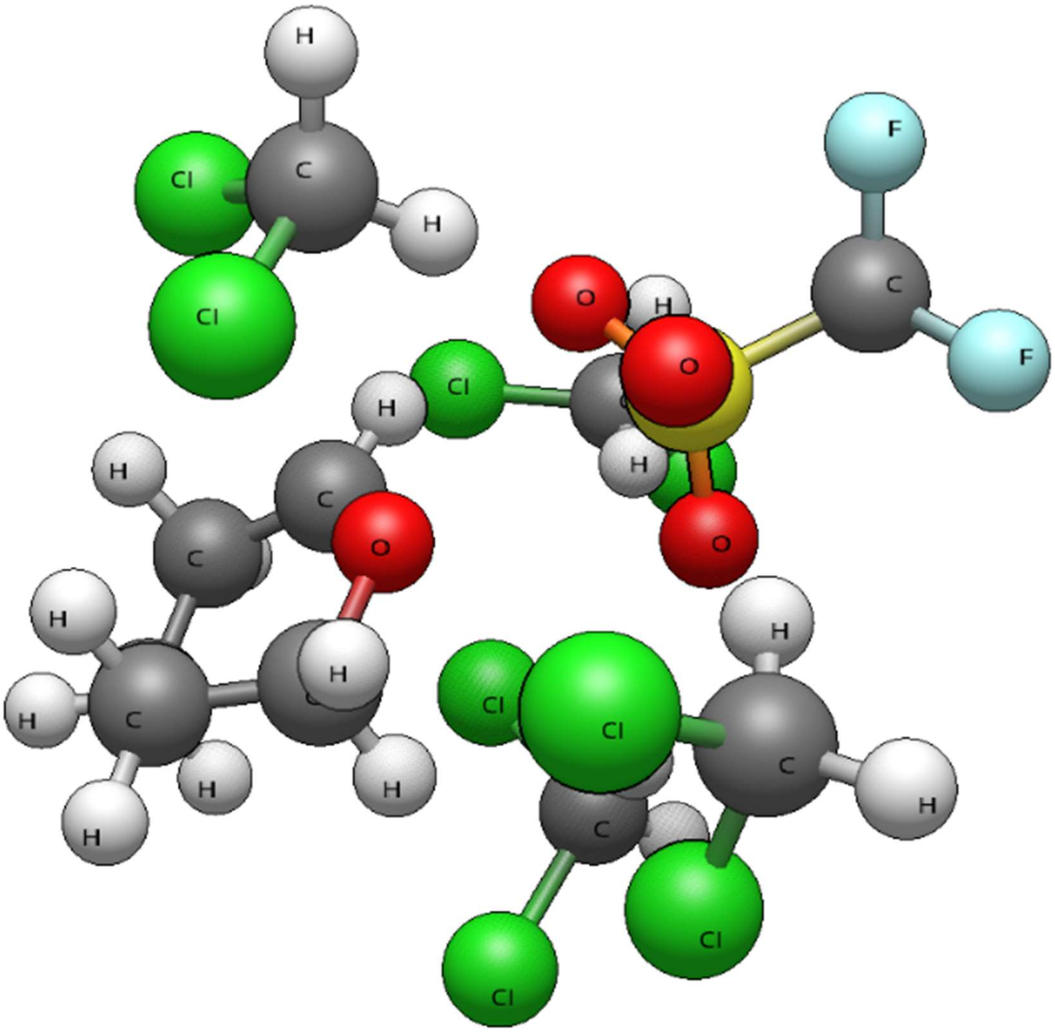

Figure S4. Structure of oxocarbenium triflate in SSIP form (**37$_{SSIP}$**)

Table S4. Cartesian Coordinates for tetrahydropyranyl triflate in gas phase (**39**):

S	-0.013808	-0.006678	0.032355
O	-0.090348	-0.022984	1.473591
O	1.260127	-0.022681	-0.654999
O	-0.991247	-1.092773	-0.543353
C	-0.603567	-1.988978	-1.705974
H	0.446763	-2.242948	-1.559822
O	-1.343761	-3.119235	-1.530431

C	-2.765231	-2.952335	-1.691744
H	-3.178201	-3.957246	-1.593677
H	-3.140872	-2.340103	-0.862926
C	-0.870507	-1.243223	-2.996688
H	-0.283473	-0.321843	-3.015237
H	-0.491115	-1.886853	-3.798814
C	-3.112586	-2.320068	-3.029031
H	-4.195954	-2.176262	-3.080090
H	-2.826957	-3.004825	-3.836057
C	-2.366208	-0.994388	-3.185742
H	-2.550543	-0.553180	-4.168504
H	-2.721593	-0.279483	-2.433512
C	-0.915022	1.508073	-0.492907
F	-0.367256	2.549963	0.112091
F	-2.187409	1.401005	-0.145281
F	-0.828235	1.660895	-1.805774

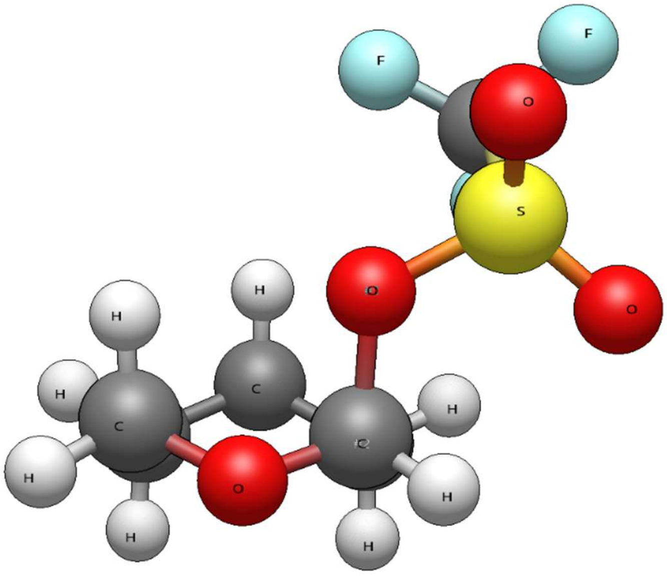

Figure S5. Structure of tetrahydropyranyl triflate in gas phase (**39**)

Table S5. Cartesian Coordinates for tetrahydropyranyl triflate transition state in gas phase:

C	-2.347390	1.251841	1.129756
C	-3.527708	0.396343	1.562824
C	-4.490088	0.262755	0.386201
C	-3.769941	-0.338466	-0.797645
C	-1.826015	0.870139	-0.200287
H	-1.517114	1.263312	1.831302
O	-2.541213	0.383161	-1.124631
F	1.463882	-1.226786	3.328862
F	-0.519285	-0.374773	3.319453
F	-0.247796	-2.475276	2.915198
H	-4.019206	0.847742	2.421900
H	-3.163086	-0.588043	1.860048
H	-2.644953	2.301135	0.992018
H	-3.439616	-1.357659	-0.600416
H	-0.882570	1.279846	-0.546949
H	-4.901012	1.238589	0.117534
H	-4.344815	-0.308753	-1.717262
H	-5.327986	-0.389465	0.631120
O	-0.958172	-0.955858	0.470148
C	0.279033	-1.271874	2.737417
S	0.449911	-0.908602	0.935180
O	1.013160	0.433959	0.888479
O	1.267345	-1.993610	0.438236

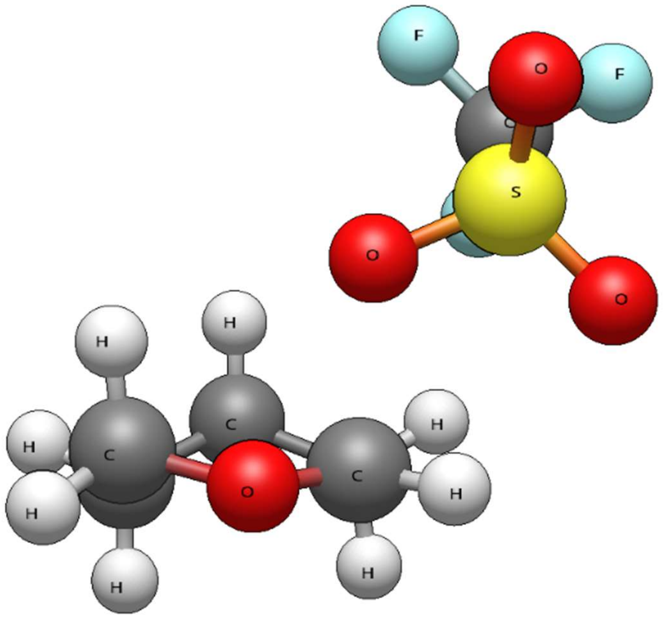

Figure S6. Structure of tetrahydropyranyl triflate transition state gas phase:

Table S6. Cartesian Coordinates for oxocarbenium triflate in gas phase (**37**):

C	2.915457	-1.695422	0.718330
C	1.547920	-1.995618	0.106850
C	0.503621	-1.963170	1.216873
C	0.681844	-0.831989	2.150814
C	2.902453	-0.331356	1.370677
H	3.753031	-0.155369	2.027517
O	1.744933	-0.163554	2.274854
H	1.538949	-2.977455	-0.371083
H	0.590915	-2.849092	1.867968
H	3.700204	-1.683878	-0.043381
H	-0.528788	-1.941188	0.861412
H	1.304562	-1.244048	-0.649121
H	3.176472	-2.460251	1.458545
H	-0.043870	-0.644641	2.936518
H	2.796412	0.479946	0.651397
F	-0.999052	1.285516	-2.563261
F	-1.012962	-0.613837	-1.528465
S	-0.377987	1.426388	-0.025193

F	-2.616890	0.800883	-1.213664
O	1.027001	1.244915	-0.412521
O	-0.798242	0.567804	1.109146
O	-0.863869	2.801214	0.038495
C	-1.306248	0.685138	-1.415571

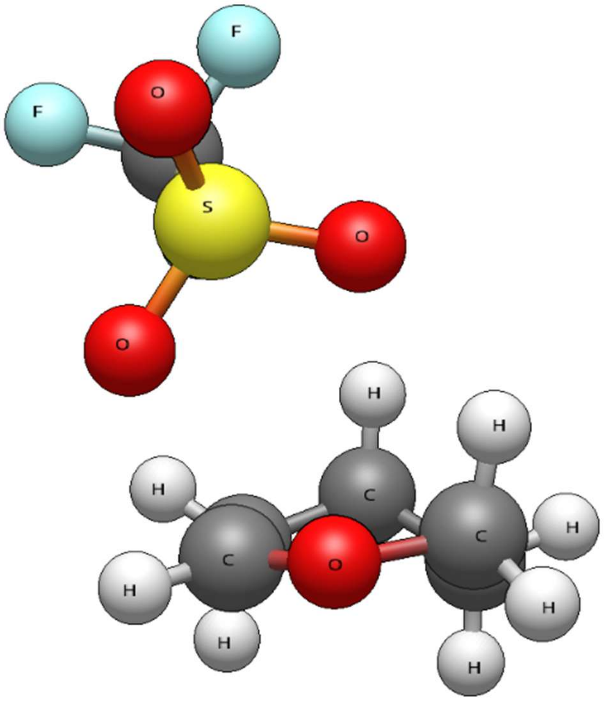

Figure S7. Structure of oxocarbenium triflate in gas phase (**37**)

Table S7. Cartesian Coordinates for solvated tetrahydrothiopyranyl triflate (**38_{solv}**):

C	-2.257030	1.240147	1.252419
C	-3.431043	0.394745	1.737154
C	-4.441271	0.114680	0.626459
C	-3.824626	-0.672047	-0.520200
C	-1.488568	0.598250	0.116987
H	-1.560253	1.448398	2.065535
S	-2.460725	0.219135	-1.336487
F	1.343796	-1.357721	3.556168
F	-0.441320	-0.146962	3.500467
F	-0.556266	-2.251944	3.053449

H	-3.920048	0.912889	2.561707
H	-3.060407	-0.554089	2.135644
H	-2.606554	2.205109	0.882037
H	-3.449101	-1.635741	-0.173591
H	-0.660001	1.220971	-0.211090
H	-4.844113	1.056645	0.247065
H	-4.542371	-0.862618	-1.315704
H	-5.277515	-0.459620	1.029407
O	-0.915352	-0.691092	0.606075
C	0.187439	-1.170280	2.943969
S	0.536936	-0.814510	1.160637
O	1.222137	0.450610	1.115019
O	1.137052	-2.008461	0.646491
Cl	-2.033868	4.539444	-0.692421
Cl	0.375604	3.649781	0.741964
H	0.214476	4.957229	-1.196354
H	-0.334284	5.836541	0.273680
C	-0.353273	4.905367	-0.276688
Cl	0.148110	2.456605	-2.655199
Cl	1.272868	-0.212584	-2.195430
C	1.530792	1.533965	-2.033605
H	1.646339	1.759107	-0.979909
H	2.409162	1.796991	-2.607062
Cl	5.501539	-1.744608	0.164262
Cl	4.451565	0.924431	-0.466448
C	4.091038	-0.679058	0.208975
H	3.793487	-0.542301	1.240054
H	3.299552	-1.121331	-0.381110
Cl	-1.781242	-3.873563	0.274965
Cl	-2.528983	-3.428362	-2.530622
C	-1.245814	-3.298665	-1.314827
H	-0.977736	-2.253128	-1.225190
H	-0.412828	-3.912902	-1.629496

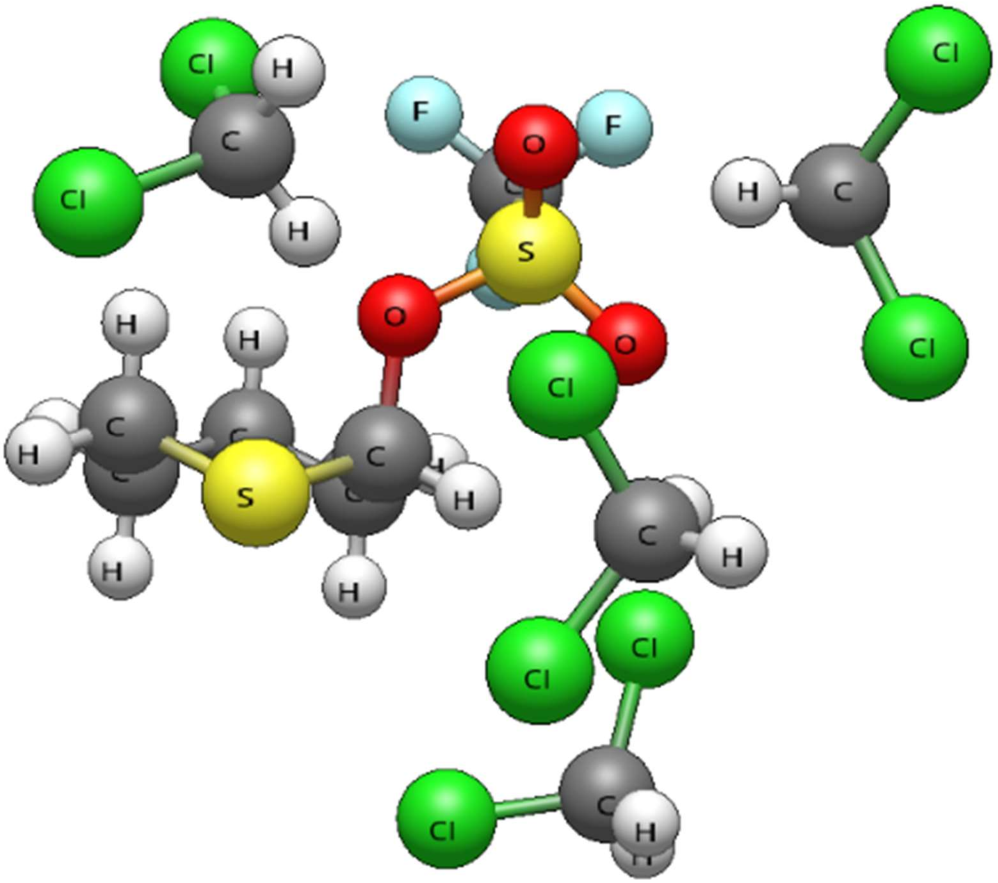

Figure S8. Structure of solvated tetrahydrothiopyranyl triflate(**38**$_{solv}$)

Table S8. Cartesian Coordinates for tetrahydrothiopyranyl triflate in transition state (**96**$^{‡}_{solv}$):

C	-2.369181	1.146206	1.200346
C	-3.557387	0.244243	1.492883
C	-4.565060	0.259118	0.347555
C	-3.997638	-0.358628	-0.918917
C	-1.706039	0.889971	-0.100296
H	-1.611030	1.105206	1.979111

S	-2.445658	0.402939	-1.496905
F	1.739825	-1.183013	3.357924
F	-0.208076	-0.255703	3.404536
F	-0.032847	-2.374684	3.044498
H	-4.034624	0.576973	2.413098
H	-3.194991	-0.772763	1.656177
H	-2.681665	2.197479	1.132313
H	-3.762574	-1.414157	-0.777965
H	-0.737722	1.350936	-0.266797
H	-4.891555	1.282356	0.147685
H	-4.677438	-0.264816	-1.763445
H	-5.451434	-0.313968	0.621918
O	-0.781111	-0.914921	0.582449
C	0.530856	-1.196034	2.814562
S	0.642718	-0.886596	0.997292
O	1.241990	0.436716	0.894704
O	1.410938	-2.010239	0.503947
Cl	-2.017631	4.540726	-0.408190
Cl	0.411555	3.512077	0.891947
H	0.224148	5.031425	-0.881280
H	-0.318472	5.734850	0.683664
C	-0.329044	4.869557	0.034518
Cl	0.171119	2.691680	-2.602987
Cl	1.095465	-0.084810	-2.386027
C	1.494836	1.610502	-2.088615
H	1.641877	1.737467	-1.022115
H	2.375910	1.864785	-2.661401
Cl	5.521784	-1.572207	0.012465
Cl	4.506062	1.107257	-0.625989
C	4.113428	-0.500774	0.028938
H	3.784880	-0.370566	1.050745
H	3.335411	-0.936474	-0.582498
Cl	-2.714952	-3.658606	0.628660
Cl	-1.539670	-3.017549	-1.988669
C	-1.288445	-3.803227	-0.419247
H	-0.453536	-3.317183	0.067218
H	-1.117721	-4.856096	-0.598651

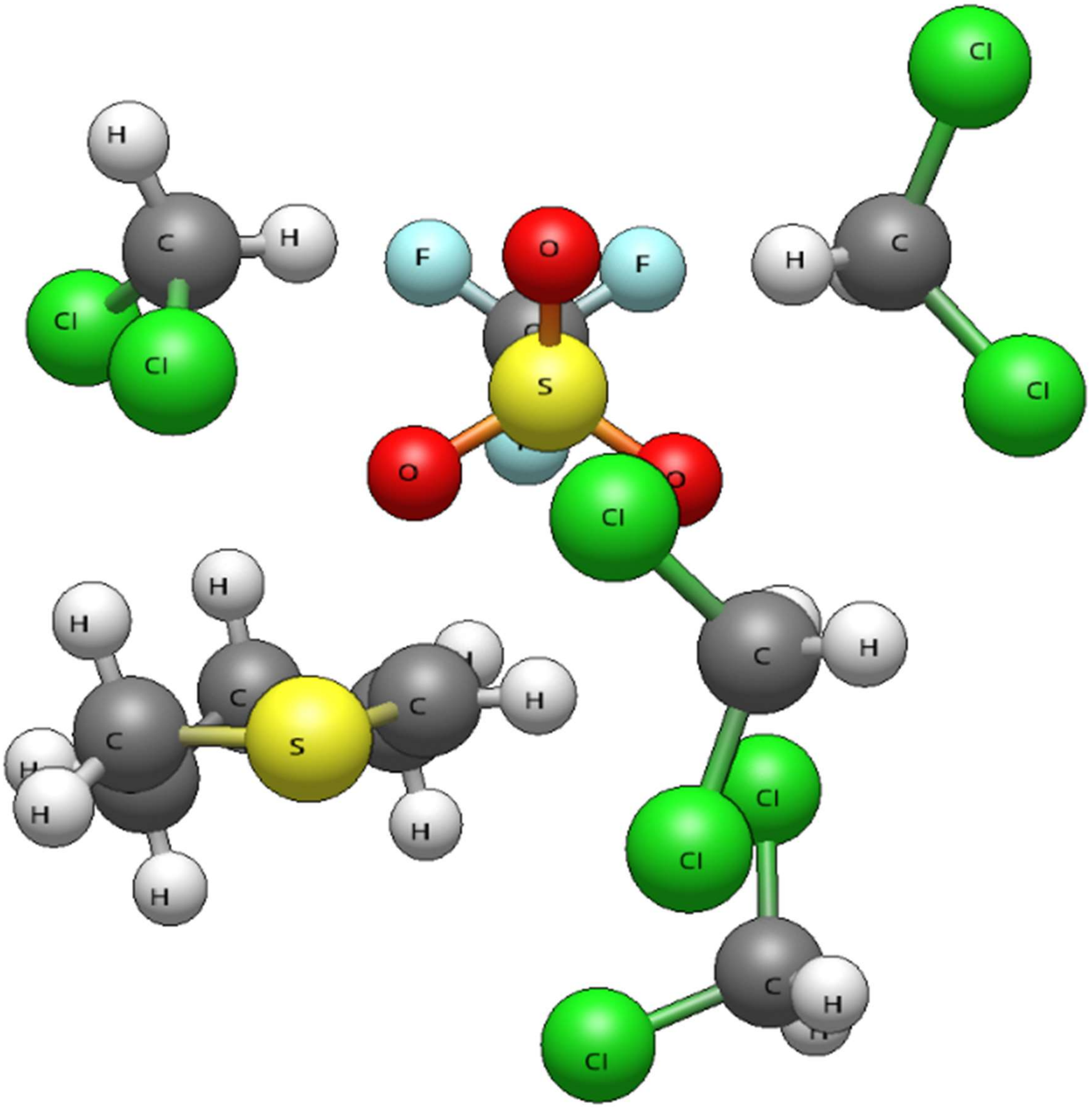

Figure S9. Structure of solvated tetrahydrothiopyranyl triflate in transition state (**96‡$_{solv}$**)

Table S9. Cartesian Coordinates for thiocarbenium triflate in SSIP form (**35$_{SSIP}$**):

C	3.491022620126	-1.660468955053	1.692390421254
C	3.024420741191	-2.797642887981	0.789996832350
C	1.513230338661	-2.785739172155	0.603797421054
C	0.878295674047	-1.464265640673	0.475687437885
C	3.282840687139	-0.307146385354	1.037590293557
H	3.530650720117	0.526391282581	1.692499879123
S	1.546467646291	0.005995411197	0.610002578333

H	3.320636485283	-3.757518837765	1.208498547742
H	0.990117271997	-3.260387299002	1.444719532034
H	4.553774903891	-1.764464893958	1.909818998926
H	1.193489044414	-3.353182051790	-0.277236444062
H	3.508750642626	-2.707614439228	-0.183318683864
H	2.957613821432	-1.685810041725	2.645779262417
H	-0.189160365648	-1.410143335360	0.282029954510
H	3.840896338942	-0.195253328821	0.107684409692
F	-2.993728985054	3.273355707126	-1.211385588982
F	-3.543101633735	1.213103734336	-1.551290877252
S	-1.351209130696	1.518397283720	-0.157195469786
F	-3.847644418660	2.105503757010	0.388753618822
O	-0.575999026368	1.565276526315	-1.393947730814
O	-1.582899767348	0.163560961130	0.356493035347
O	-0.930426545768	2.471642610061	0.860473201601
C	-3.037741709206	2.066845461881	-0.662837930957
H	-2.565699081475	-1.398015708396	-0.919405764056
Cl	-3.428857754728	-3.026965715532	-2.394906388821
C	-3.058616255253	-2.354601169355	-0.803470952440
H	-3.977725836838	-2.273489120397	-0.239366897278
Cl	-1.962279753251	-3.420267004932	0.115767981149
Cl	2.743262600409	-1.134705070899	-2.584090521011
C	1.102807930993	-0.541266801357	-2.955270086183
H	1.065027396095	-0.334777254107	-4.016236109777
H	0.899166442203	0.337141403181	-2.353124986984
Cl	-0.125930128660	-1.760868732419	-2.585207017840
Cl	2.482356747761	3.236997897279	1.310036096788
C	1.962132208885	3.391749476888	-0.378040907980
Cl	2.883872359827	2.304579381923	-1.445492799188
H	2.152959835443	4.406979110115	-0.697674632623
H	0.916934036590	3.114751308169	-0.437172109394
Cl	-0.666141239531	-1.362509331336	3.311796227146
Cl	1.335311391064	0.701790129859	3.907998870365
H	-0.569845735805	0.858049650357	2.515970184028
H	-0.982587270743	0.757297619276	4.263051936006
C	-0.358528490184	0.377800517620	3.465621300958

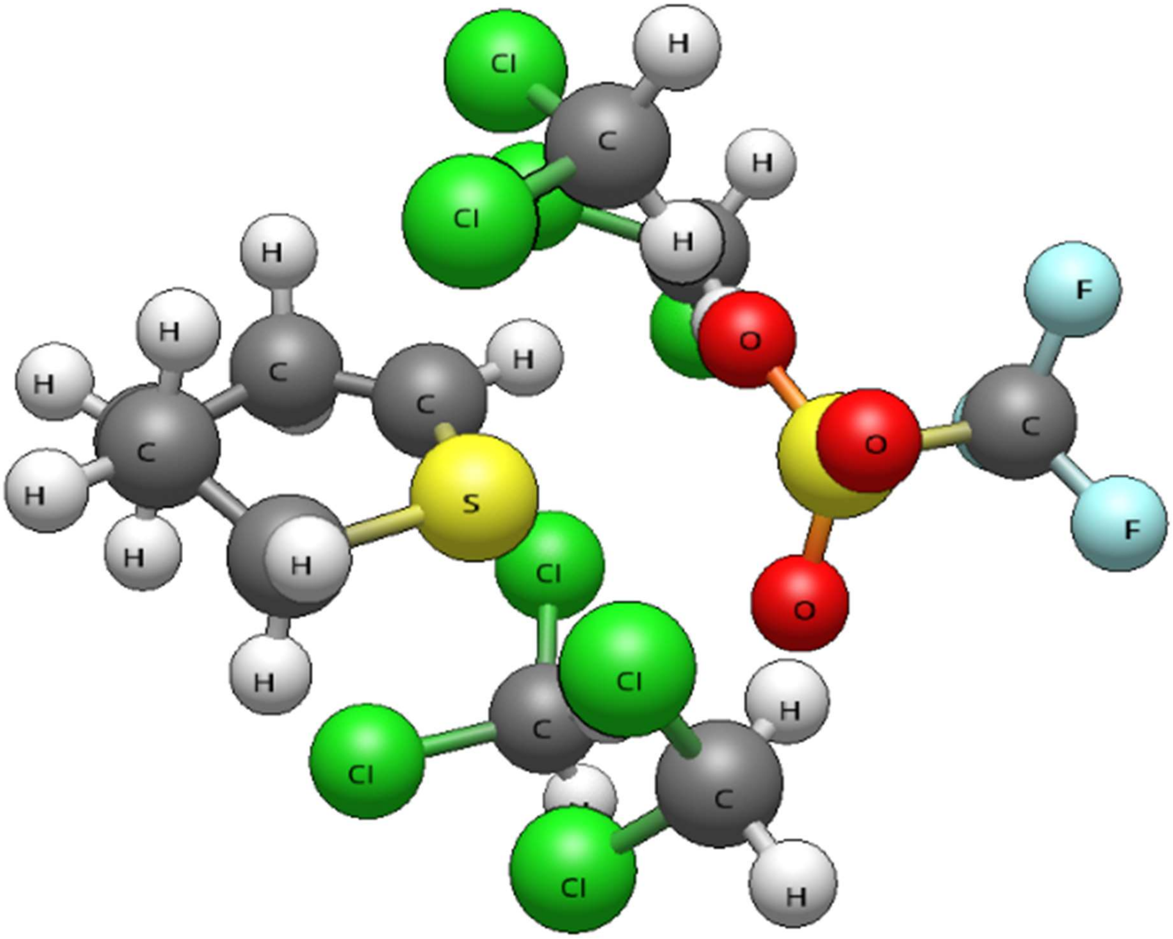

Figure S10. Structure of solvated thiocarbenium triflate in SSIP form (**35**$_{SSIP}$)

Table S10. Cartesian Coordinates for tetrahydrothiopyranyl triflate in gas phase (**38**):

S	0.041459	0.077939	-0.052159
O	0.043778	0.125192	1.386138
O	1.259643	0.149889	-0.821241
O	-0.827227	-1.169017	-0.492568
C	-0.425471	-2.014529	-1.642621
H	0.645902	-2.197680	-1.559351
S	-1.248346	-3.606366	-1.430956
C	-2.963513	-3.030118	-1.727934
H	-3.572626	-3.935663	-1.746929
H	-3.272710	-2.433607	-0.864629
C	-0.782571	-1.286153	-2.927833

H	-0.183062	-0.372225	-2.984174
H	-0.470990	-1.922150	-3.762116
C	-3.133198	-2.227992	-3.015507
H	-4.189307	-1.948505	-3.101966
H	-2.891343	-2.856105	-3.880186
C	-2.272555	-0.966008	-3.037893
H	-2.447099	-0.407584	-3.962282
H	-2.576241	-0.308180	-2.215345
C	-1.032930	1.479770	-0.581215
F	-0.593517	2.567333	0.021907
F	-2.280151	1.237736	-0.223046
F	-0.964928	1.637872	-1.890619

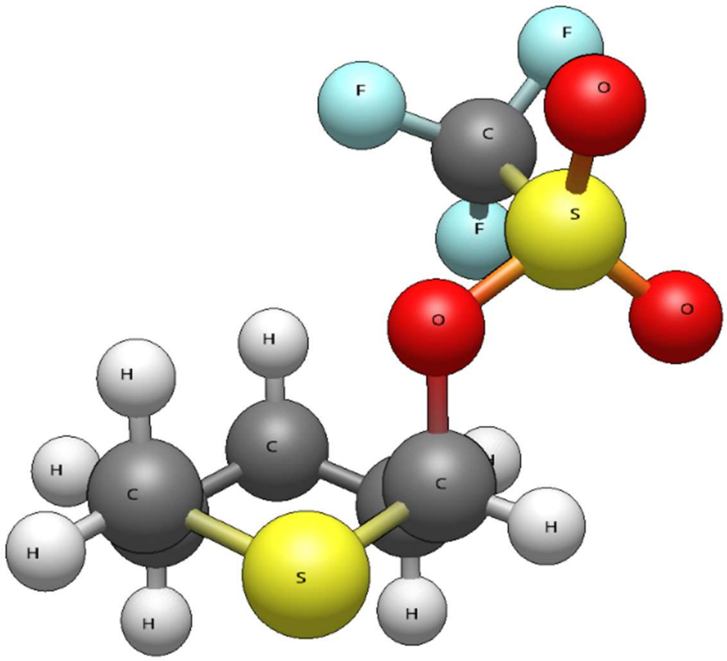

Figure S11. Structure of tetrahydrothiopyranyl triflate in gas phase (**38**):

Table S11. Cartesian Coordinates for tetrahydrothiopyranyl triflate transition state in gas phase:

C	-2.369181	1.146206	1.200346
C	-3.557387	0.244243	1.492883

C	-4.565060	0.259118	0.347555
C	-3.997638	-0.358628	-0.918917
C	-1.706039	0.889971	-0.100296
H	-1.611030	1.105206	1.979111
S	-2.445658	0.402939	-1.496905
F	1.739825	-1.183013	3.357924
F	-0.208076	-0.255703	3.404536
F	-0.032847	-2.374684	3.044498
H	-4.034624	0.576973	2.413098
H	-3.194991	-0.772763	1.656177
H	-2.681665	2.197479	1.132313
H	-3.762574	-1.414157	-0.777965
H	-0.737722	1.350936	-0.266797
H	-4.891555	1.282356	0.147685
H	-4.677438	-0.264816	-1.763445
H	-5.451434	-0.313968	0.621918
O	-0.781111	-0.914921	0.582449
C	0.530856	-1.196034	2.814562
S	0.642718	-0.886596	0.997292
O	1.241990	0.436716	0.894704
O	1.410938	-2.010239	0.503947

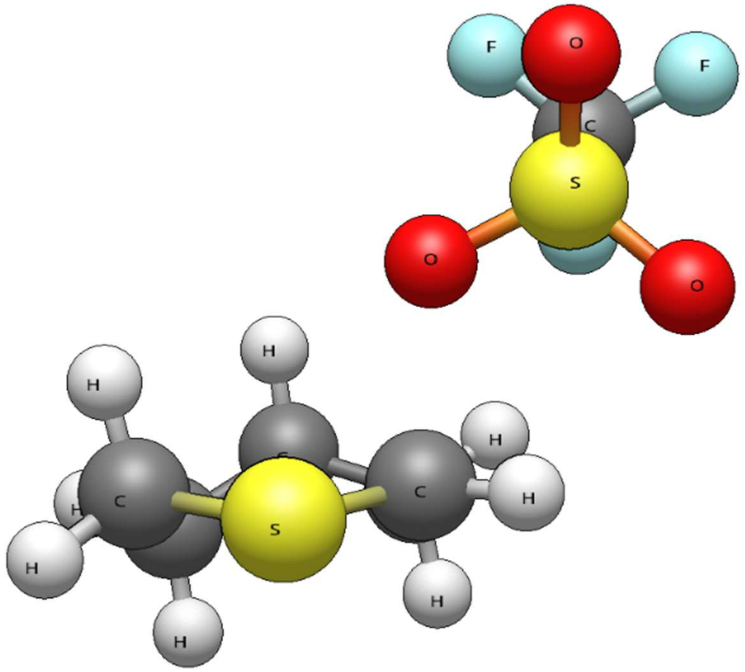

Figure S12. Structure of tetrahydrothiopyranyl triflate transition state in gas phase

Table S12. Cartesian Coordinates for thiocarbenium triflate in gas phase (**35**):

C	2.949794	-1.735992	0.710938
C	1.555905	-1.949343	0.124462
C	0.475816	-1.934029	1.201882
C	0.595911	-0.893290	2.242586
C	3.115066	-0.335515	1.283120
H	4.079908	-0.198823	1.777342
S	1.886028	0.057046	2.575687
H	1.509688	-2.910315	-0.393981
H	0.467539	-2.876361	1.777127
H	3.707302	-1.850187	-0.070263
H	-0.532641	-1.841528	0.784238
H	1.344834	-1.164045	-0.606029
H	3.161371	-2.483031	1.484858
H	-0.217837	-0.795550	2.957625
H	2.980160	0.438048	0.522596
F	-0.836583	1.191913	-2.546118

F	-0.972428	-0.653687	-1.430853
S	-0.538389	1.467732	0.041433
F	-2.611662	0.754439	-1.391860
O	0.903815	1.265121	-0.170929
O	-1.094582	0.692027	1.167474
O	-1.009404	2.848485	-0.059006
C	-1.283747	0.647822	-1.415196

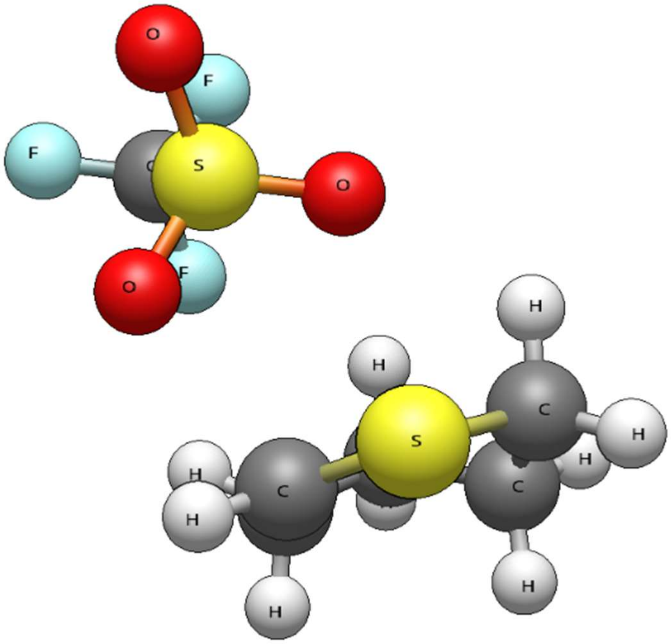

Figure S13. Structure of thiocarbenium triflate in gas phase (**35**)

3. Variable temperature NMR experiments

3.1. Experimental protocols of Variable Temperature NMR experiments with 2,3-dihydro-4*H*-thiopyran, 2,3-dihydro-4*H*-pyran, and phenyl vinyl and ethyl vinyl sulfides

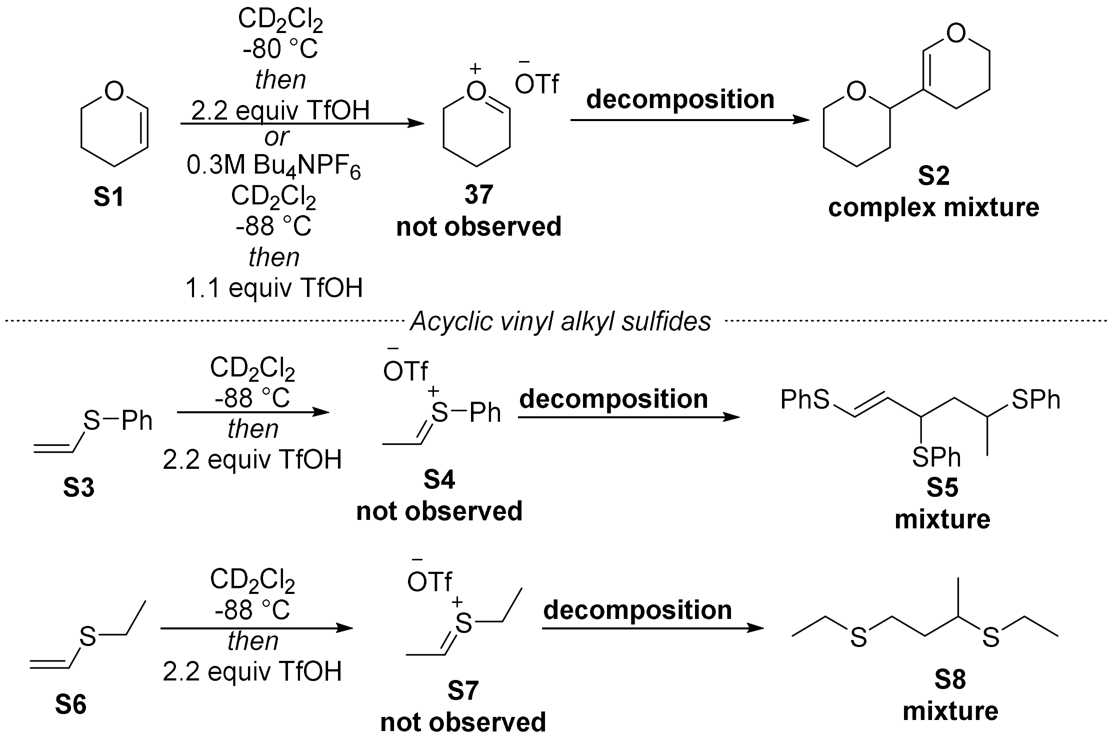

Scheme S1. Additional VT NMR experiments performed with non-carbohydrate-based substrates

Protonation of 2,3-dihydro-4*H*-thiopyran with TfOH at -88 °C:

2,3-Dihydro-4*H*-thiopyran **34** (30 mg, 0.3 mmol) was dissolved in CD_2Cl_2 (0.75 mL), and the reaction mixture was transferred into a vacuum dried NMR tube and sealed with a septum cap under an Ar atmosphere. The NMR tube then was placed into the NMR probe and cooled down to -88 °C and ^1H, ^{13}C and spectra were collected. The tube was quickly removed from the probe and precooled TfOH (58 µL, 0.66 mmol, 2.2 equiv) was quickly added and the tube quickly shaken before the tube was returned to the cold NMR probe. ^1H, DEPT, ^{13}C, HMQC, HMBC, and ^{19}F spectra were recorded as soon as possible at -88 °C. The temperature then was increased to -78 °C and ^1H and ^{19}F spectra were recorded after 5 minutes. After that the temperature was increased -60 °C and ^1H and ^{19}F spectra were recorded after 5 mins. After that the temperature was increased by 10

°C increments and ^1H and ^{19}F spectra were recorded after 5 minutes at each temperature. After termination of the VT NMR experiment the reaction mixture was diluted with EtOAc (30 mL), washed with $NaHCO_3$ (10 mL), brine (20 mL), dried over $MgSO_4$ and concentrated to dryness to give a complex mixture (18 mg) of self-condensation products containing dimer **36**:[33]

^1H NMR (500 MHz, $CDCl_3$): δ 5.99 (s, 1H), 3.27 – 3.21 (m, 1H).

^{13}C NMR (126 MHz, $CDCl_3$): δ 132.4, 115.4.

HRMS (m/z): $[M+H]^+$ calcd. for $C_{10}H_{17}S_2^+$ 201.0766, found 201.0760.

Protonation of 2,3-dihydro-4*H*-thiopyran with TfOH at -78 °C in the presence of quaternary ammonium salt:

2,3-dihydro-4*H*-thiopyran **34** (40 mg, 0.399 mmol) and Bu_4NPF_6 (87 mg, 0.225 mmol, 1.77 equiv) were dissolved in CD_2Cl_2 (0.75 mL), and the reaction mixture was transferred into a vacuum dried NMR tube and sealed with a septum cap under an Ar atmosphere. The NMR tube containing glycosyl donor solution then was placed into the NMR probe and cooled down to -78 °C. The first ^1H, ^{13}C spectra were collected, then the sample was quickly removed from the probe and precooled TfOH (39 µL, 0.089 mmol, 1.1 equiv) was quickly added and the tube quickly shaken. The sample then was returned to the cold NMR probe, and ^1H, ^{13}C, DEPT, HMQC, and ^{19}F spectra were recorded as soon as possible. The temperature then was increased to -60 °C and ^1H, ^{19}F spectra were recorded after 5 minutes. After that the temperature was increased by 10 °C increments and ^1H, ^{19}F spectra were recorded at each temperature after 5 minutes. After termination of the VT NMR experiment the sample was collected and diluted with EtOAc (30 mL), washed with $NaHCO_3$ (10 mL), brine (20 mL), dried over $MgSO_4$ and concentrated to dryness to give complex mixture (25 mg) of self-condensation products containing dimer[33] **36** and quaternary ammonium salt identified by the following diagnostic signals.

^1H NMR (500 MHz, $CDCl_3$): δ 5.99 (s, 1H), 3.34 – 2.95 (m, 2H), 3.27 – 3.21 (m, 1H), 1.62 – 1.52 (m, 2H), 1.37 (h, *J* = 7.4 Hz, 2H), 0.95 (t, *J* = 7.4 Hz, 3H).

^{13}C NMR (126 MHz, $CDCl_3$): δ 132.4, 115.4, 58.5, 23.8, 19.6, 13.5.

HRMS (m/z): $[M+H]^+$ calcd. for $C_{10}H_{17}S_2^+$ 201.0766, found 201.0760.

Diagnostic signals of thienium cation (35):

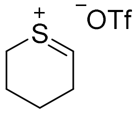

^{1}H NMR (500 MHz, CD_2Cl_2): δ 11.23 (s, 1H, H-1), 3.72 – 3.60 (m, 1H, H-5), 2.44 – 1.57 (m, 1H, H-2).

^{13}C NMR (126 MHz, CD_2Cl_2): δ 238.3 (<u>C</u>=S^+), 40.2 (C-5), 20.7 (C-2).

Protonation of 2,3-dihydro-4*H*-pyran with TfOH at -88 °C:

2,3-dihydro-4*H*-pyran **S1** (44 mg, 0.523 mmol) was dissolved in CD_2Cl_2 (0.75 mL), and the reaction mixture was transferred into a vacuum dried NMR tube and sealed with a septum cap under an Ar atmosphere. The NMR tube containing glycosyl donor solution then was placed into the NMR probe and cooled down to -88 °C. The first ^{1}H, ^{13}C spectra were collected, then the sample was quickly removed from the probe and precooled TfOH (102 µL, 1.15 mmol, 2.2 equiv) was quickly added and the tube quickly shaken. The sample then was returned to the cold NMR probe, and ^{1}H, ^{13}C and ^{19}F spectra were recorded as soon as possible at-88 °C. The temperature then was increased to -78 °C and ^{1}H, ^{19}F spectra were recorded after 5 minutes. After that the temperature was increased to -60 °C and ^{1}H, ^{13}C, and ^{19}F spectra were recorded after 5 minutes. After that the temperature was increased by 10 °C increments and ^{1}H, ^{13}C, and ^{19}F spectra were recorded at each temperature after 5 minutes. After termination of the VT NMR experiment the sample was collected and diluted with EtOAc (40 mL), washed with $NaHCO_3$ (30 mL), brine (30 mL), dried over $MgSO_4$ and concentrated to dryness to give mixture (21 mg) of self-condensation products containing dimer **S2**.

HRMS (m/z): $[M+H]^+$ calcd. for $C_{10}H_{17}O_2^+$ 169.1223, found 169.1215.

Protonation of 2,3-dihydro-4*H*-pyran with TfOH at -78 °C in the presence of quaternary ammonium salt:

2,3-dihydro-4*H*-pyran (45 mg, 0.535 mmol) and Bu_4NPF_6 (87 mg, 0.225 mmol, 0.42 equiv) were dissolved in CD_2Cl_2 (0.75 mL), and the reaction mixture was transferred into a vacuum dried NMR tube and sealed with a septum cap under an Ar atmosphere. The NMR tube containing glycosyl donor solution then was placed into the NMR probe and cooled down to -78 °C. The first ^1H, ^{13}C spectra were collected, then the sample was quickly removed from the probe and precooled TfOH (52 µL, 0.589 mmol, 1.1 equiv) was quickly added and the tube quickly shaken. The sample then was returned to the cold NMR probe, and ^1H, ^{13}C and ^{19}F spectra were recorded as soon as possible at -78 °C. The temperature then was increased to -60 °C and ^1H, ^{19}F spectra were recorded after 5 minutes After that the temperature was increased by 10 °C increments and ^1H, ^{13}C, and ^{19}F spectra were recorded at each temperature after 5 minutes. After termination of the VT NMR experiment the sample was collected and diluted with EtOAc (40 mL), washed with $NaHCO_3$ (30 mL), brine (30 mL), dried over $MgSO_4$ and concentrated to dryness to give mixture (27 mg) of self-condensation products containing dimer and mainly quaternary ammonium salt identified by diagnostic signals identical to those described above.

Protonation of phenyl vinyl sulfide S3 with TfOH at -88 °C:

Phenyl vinyl sulfide **S4** (40 mg, 0.293 mmol) was dissolved in CD_2Cl_2 (0.75 mL), and the reaction mixture was transferred into a vacuum dried NMR tube and sealed with a septum cap under an Ar atmosphere. The NMR tube containing glycosyl donor solution then was placed into the NMR probe and cooled down to -88 °C. The first ^1H, ^{13}C spectra were collected, then the sample was quickly removed from the probe and precooled TfOH (57 µL, 0.645 mmol, 2.2 equiv) was quickly added and the tube quickly shaken. The sample then was returned to the cold NMR probe, and ^1H, ^{13}C and ^{19}F spectra were recorded as soon as possible at-88 °C. The temperature then was increased to -78 °C and ^1H, ^{19}F spectra were recorded after 5 minutes. After that the temperature was increased to -60 °C and ^1H, ^{13}C, and ^{19}F spectra were recorded after 5 minutes. After that the temperature

was increased by 10 °C increments and ^{1}H, ^{13}C, and ^{19}F spectra were recorded at each temperature after 5 minutes. After termination of the VT NMR experiment the sample was collected and diluted with EtOAc (30 mL), washed with $NaHCO_3$ (20 mL), brine (20 mL), dried over $MgSO_4$ and concentrated to dryness to give mixture (26 mg) of self-condensation products containing dimer **S5**.

HRMS (m/z): $[M+H]^+$ calcd. for $C_{24}H_{25}S_3^+$ 409.1113, found 409.1090.

Protonation of ethyl vinyl sulfide S6 with TfOH at -88 °C:

Ethyl vinyl sulfide **S7** (30 mg, 0.316 mmol) was dissolved in CD_2Cl_2 (0.75 mL), and the reaction mixture was transferred into a vacuum dried NMR tube and sealed with a septum cap under an Ar atmosphere. The NMR tube containing glycosyl donor solution then was placed into the NMR probe and cooled down to -88 °C. The first ^{1}H, ^{13}C spectra were collected, then the sample was quickly removed from the probe and precooled TfOH (61 μL, 0.695 mmol, 2.2 equiv) was quickly added and the tube quickly shaken. The sample then was returned to the cold NMR probe, and ^{1}H, ^{13}C and ^{19}F spectra were recorded as soon as possible at-88 °C. The temperature then was increased to -78 °C and ^{1}H, ^{19}F spectra were recorded after 5 minutes. After that the temperature was increased to -60 °C and ^{1}H, ^{13}C, and ^{19}F spectra were recorded after 5 minutes. After that the temperature was increased by 10 °C increments and ^{1}H, ^{13}C, and ^{19}F spectra were recorded at each temperature after 5 minutes. After termination of the VT NMR experiment the sample was collected and diluted with EtOAc (30 mL), washed with $NaHCO_3$ (20 mL), brine (20 mL), dried over $MgSO_4$ and concentrated to dryness to give mixture (26 mg) of self-condensation products containing dimer **S8**.

HRMS (m/z): $[M+H]^+$ calcd. for $C_8H_{17}S_2^+$ 177.0766, found 177.0757.

3.2. Experimental protocols of Variable Temperature NMR experiments with various glucosyl and 5-thioglucosyl donors and characterization data of reactive intermediates

Activation of (*R_S*),(*S_S*)-Ethyl 2,3,4,6-tetra-*O*-acetyl-1-thio-*β*-D-glucopyranoside-*S*-Oxides (50) with Tf_2O:

Glycosyl sulfoxide **50** (32 mg, 0.079 mmol) was dissolved in CD_2Cl_2 (0.75 mL), and the reaction mixture was transferred into a vacuum dried NMR tube and sealed with a septum cap under an Ar atmosphere. The NMR tube containing glycosyl donor solution then was placed into the NMR probe and cooled down to -78 °C. The first ^{1}H, ^{19}F spectra were collected, then the sample was quickly removed from the probe and precooled Tf_2O (15 μL, 0.089 mmol, 1.13 equiv) was quickly added and the tube quickly shaken. The sample then was returned to the cold NMR probe, and ^{1}H, ^{13}C spectra were recorded shortly after. The temperature then was increased to -60 °C and ^{1}H, ^{19}F spectra were recorded after 5 minutes. After that the temperature was increased by 10 °C increments and 1H, 19F spectra were recorded at each temperature after 5 minutes. Additionally, at -30 °C ^{13}C, DEPT, COSY, HMQC, and HMBC spectra were recorded. After termination of the VT NMR experiment the sample was collected and diluted with EtOAc (30 mL). The resulting solution was washed with sat aq $NaHCO_3$ (20 mL), brine (20 mL), dried over $MgSO_4$ and concentrated to dryness. The crude product was purified by flash column chromatography eluting with hexanes:EtOAc (0→50%, EtOAc) to give a mixture containing anomeric acetates **11** ($\alpha : \beta$ = 9 : 1, 10.1 mg, 33%) and enone **69** (1 mg, 5.5%) with molar ratio **11** : **69** = 5.9 : 1[2] as yellowish syrup (11 mg) with spectral data identical to that reported in the literature:[34, 35]

[2] Determined spectroscopically

1,2,3,4,6-penta-*O*-acetyl-*α,β*-D-glucopyranoside (11):

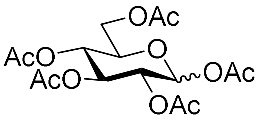

^{1}H NMR (500 MHz, $CDCl_3$): δ 6.31 (d, *J* = 3.7 Hz, 1H), 5.50 – 5.40 (m, 1H), 5.16 – 5.05 (m, 2H), 4.37 (dd, *J* = 11.7, 5.2 Hz, 1H), 4.30 – 4.17 (m, 1H), 4.14 – 4.02 (m, 1H), 2.16 (s, 3H), 2.07 (s, 3H), 2.02 (s, 3H), 2.01 (s, 3H), 2.00 (s, 3H).

^{13}C NMR (126 MHz, $CDCl_3$): δ 170.7, 170.3, 169.7, 169.5, 168.8, 89.2, 69.9, 69.3, 68.0, 64.4, 20.9, 20.8, 20.7, 20.6, 20.5.

The minor *β*-isomer was identified by characteristic signals: δ 5.70 (d, *J* = 8.3 Hz, 1H), 5.23 (t, *J* = 9.4 Hz, 1H).

ESI-HRMS (m/z): $[M+Na]^+$ calcd. for $C_{16}H_{32}O_{11}Na^+$ 413.1054, found 413.1045.

1,6-di-*O*-acetyl-3,4-dideoxy-*β*-D-glycero-hex-3-enopyranos-2-ulose (69):

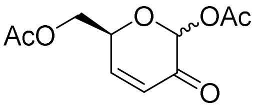

^{1}H NMR (500 MHz, $CDCl_3$): δ 7.03 (dd, *J* = 10.7, 1.7 Hz, 1H), 6.27 (dd, *J* = 10.8, 2.6 Hz, 1H), 6.18 – 6.13 (m, 1H), 4.84 – 4.77 (m, 1H), 4.30 – 4.17 (m, 1H), 4.14 – 4.02 (m, 1H), 2.11 (s, 3H), 2.08 (s, 3H).

^{13}C NMR (126 MHz, $CDCl_3$): δ 188.5, 170.7, 168.8, 147.3, 126.6, 89.5, 68.2, 61.5, 20.9, 20.8.

ESI-HRMS (m/z): $[M+Na]^+$ calcd. for $C_{10}H_{12}O_6Na^+$ 251.0526, found 251.0520.

Activation of *(R$_S$)*-Ethyl 2,3,4,6-tetra-*O*-acetyl-1-thio-*α*-D-glucopyranoside-*S*-Oxide (51) with Tf_2O:

Glycosyl sulfoxide **51** (37 mg, 0.081 mmol) was dissolved in CD_2Cl_2 (0.75 mL), and the reaction mixture was transferred into a vacuum dried NMR tube and sealed with a septum cap under an Ar atmosphere. The NMR tube containing glycosyl donor solution then was placed into the NMR probe and cooled down to -78 °C. The first ^{1}H, ^{13}C spectra were collected, then the sample was quickly removed from the probe and precooled Tf_2O (15

μL, 0.089 mmol, 1.1 equiv) was quickly added and the tube quickly shaken. The sample then was returned to the cold NMR probe, and ^{1}H, ^{19}F spectra were recorded shortly after. The temperature then was increased to -60 °C and ^{1}H, ^{19}F spectra were recorded after 5 minutes. After that the temperature was increased by 10 °C increments and ^{1}H, ^{19}F spectra were recorded at each temperature after 5 minutes. Additionally, at -60° C ^{13}C, DEPT, COSY, HMQC, and HMBC spectra were recorded. After termination of the VT NMR experiment the sample was collected and diluted with EtOAc (20 mL). The resulting solution was washed with sat aq $NaHCO_3$ (15 mL), brine (15 mL), dried over $MgSO_4$ and concentrated to dryness. The crude product was purified by flash column chromatography eluting with hexanes:EtOAc (0→50%, EtOAc) to give a mixture containing anomeric acetates **11** (*α : β* = 9 : 1, 8 mg, 26%) and enone **69** (2 mg, 11%) with molar ratio **11** : **69** = 2.36 : 1[3] as yellowish syrup (10 mg) with spectral data identical to that presented above.[34, 35]

Diagnostic signals of 3,4,6-tri-O-acetyl-*α*-D-glucopyranose-1,2-O-acetoxonium trifluoromethanesulfonate intermediate (67):

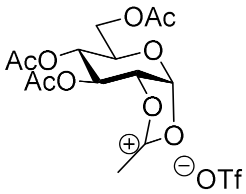

^{1}H NMR (500 MHz, CD_2Cl_2): δ 7.24 (d, *J* = 7.5 Hz, 1H, H-1), 5.70 – 5.60 (m, 1H, H-2), 5.00 (d, *J* = 9.9 Hz, 1H, H-3), 5.34 – 5.25 (m, 1H, H-4), 2.85 (s, 3H, CH_3C^+).
^{13}C NMR (126 MHz, CD_2Cl_2): δ 191.6 ($CH_3\underline{C}^+$), 112.0 (C-1), 81.4 (C-2), 68.0 (C-3), 66.8 (C-4), 16.3 ($\underline{C}H_3C^+$).

[3] Determined spectroscopically

Diagnostic signals of 1-*O*-trifluoromethanesulfonyl-2,3,4,6-tetra-*O*-acetyl-*α*-D-glucopyranose intermediate (68):

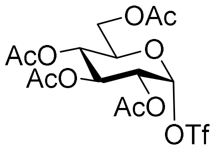

^{1}H NMR (500 MHz, CD_2Cl_2): δ 6.16 (d, *J* = 3.4 Hz, 1H, H-1), 5.16 – 5.11 (m, 2H, H-2, H-3).
^{13}C NMR (126 MHz, CD_2Cl_2): 103.5 (C-1), 68.4 (C-2).
^{19}F NMR (470 MHz, CD_2Cl_2): δ -75.51.

Activation of (*Rs*), (*Ss*)-Ethyl 2,3,4,6-tetra-*O*-acetyl-1,5-di-thio-*β*-D-glucopyranoside-1-*S*-Oxides (40) with Tf_2O:

Glycosyl sulfoxide **40** (30 mg, 0.071 mmol) was dissolved in CD_2Cl_2 (0.75 mL), and the reaction mixture was transferred into a vacuum dried NMR tube and sealed with a septum cap under an Ar atmosphere. The NMR tube containing glycosyl donor solution then was placed into the NMR probe and cooled down to -78 °C. The first ^{1}H, ^{13}C spectra were collected, then the sample was quickly removed from the probe and precooled Tf_2O (13 µL, 0.078 mmol, 1.1 equiv) was quickly added and the tube quickly shaken. The sample then was returned to the cold NMR probe, and ^{1}H, ^{19}F spectra were recorded shortly after. The temperature then was increased to -60 °C and ^{1}H, ^{19}F spectra were recorded after 5 minutes. After that the temperature was increased by 10 °C increments and ^{1}H, ^{19}F spectra were recorded at each temperature after 5 minutes. Additionally, at -50 °C ^{13}C, DEPT, COSY, HMQC, and HMBC spectra were recorded. After termination of the VT NMR experiment the sample was collected and diluted with EtOAc (20 mL). The resulting solution was washed with sat aq $NaHCO_3$ (10 mL), brine (10 mL), dried over $MgSO_4$ and concentrated to dryness. The crude product was purified by a flash column chromatography eluting with hexanes:EtOAc (0→50%, EtOAc) to give α-acetate **9α** as a syrup (9 mg, 31%) with spectral data identical to that reported in the literature.[36, 37]
^{1}H NMR (500 MHz, $CDCl_3$): δ 6.14 (d, *J* = 3.2 Hz, 1H), 5.42 (t, *J* = 9.9 Hz, 1H), 5.31 (dd, *J* = 10.8, 9.5 Hz, 1H), 5.23 (dd, *J* = 10.2, 3.2 Hz, 1H), 4.43 – 4.33 (m, 1H), 4.06 (dd, *J* =

12.1, 3.1 Hz, 1H), 3.58 (ddd, J = 10.8, 4.9, 3.1 Hz, 1H), 2.17 (s, 3H), 2.06 (s, 3H), 2.03 (s, 3H), 2.00 (s, 3H), 1.98 (s, 3H).

^{13}C NMR (126 MHz, $CDCl_3$): δ 170.6, 169.9, 169.7, 169.5, 169.1, 73.2, 71.8, 70.7, 70.7, 61.0, 39.9, 21.0, 20.7, 20.6, 20.6, 20.6.

ESI-HRMS (*m/z*): $[M+Na]^+$ calcd. for $C_{16}H_{22}NaO_{10}S^+$ 429.0826, found 429.0820.

Activation of Ethyl 2,3,4,6-tetra-*O*-acetyl-1,5-di-thio-α-D-glucopyranoside-1-*S*-Oxide (41) with Tf_2O:

Glycosyl sulfoxide **41** (32 mg, 0.075 mmol) was dissolved in CD_2Cl_2 (0.75 mL), and the reaction mixture was transferred into a vacuum dried NMR tube and sealed with a septum cap under an Ar atmosphere. The NMR tube containing glycosyl donor solution then was placed into the NMR probe and cooled down to -78 °C. The first ^{1}H, ^{13}C spectra were collected, then the sample was quickly removed from the probe and precooled Tf_2O (14 μL, 0.0825 mmol, 1.1 equiv) was quickly added and the tube quickly shaken. The sample then was returned to the cold NMR probe, and ^{1}H, ^{19}F spectra were recorded shortly after. The temperature then was increased to -60 °C and ^{1}H, ^{19}F spectra were recorded after 5 minutes. After that the temperature was increased by 10 °C increments and ^{1}H, ^{19}F spectra were recorded at each temperature after 5 minutes. Additionally, at -50 °C ^{13}C, DEPT, COSY, HMQC, and HMBC spectra were recorded. After termination of the VT NMR experiment the sample was collected and diluted with EtOAc (20 mL). The resulting solution was washed with sat aq $NaHCO_3$ (20 mL), brine (20 mL), dried over $MgSO_4$ and concentrated to dryness. The crude product was purified by a flash column chromatography eluting with hexanes:EtOAc (0→50%, EtOAc) to give α-acetate **9α** as a syrup (11 mg, 36%) with spectral data identical to that presented above. [36, 37]

Diagnostic signals of 3,4,6-tri-O-acetyl-5-thio-*α*-D-glucopyranose-1,2-O-acetoxonium trifluoromethanesulfonate intermediate (76):

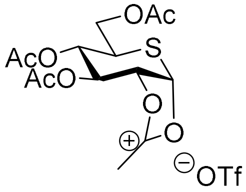

^{1}H NMR (500 MHz, CD_2Cl_2): δ 7.22 (d, *J* = 9.5 Hz, 1H, H-1), 2.89 (s, 3H, $C\underline{H}_3C^+$).
^{13}C NMR (126 MHz, CD_2Cl_2): δ 191.4 ($CH_3\underline{C}^+$), 16.4 ($\underline{C}H_3C^+$).

Diagnostic signals of 1-trifluoromethanesulfonyl-2,3,4,6-tetra-*O*-acetyl-5-thio-*α*-D-glucopyranoside intermediate (77):

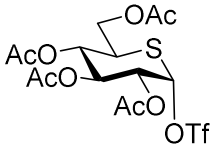

^{1}H NMR (500 MHz, CD_2Cl_2): δ 6.05 (d, *J* = 3.0 Hz, 1H, H-1), 5.24 – 5.15 (m, 1H, H-2).
^{13}C NMR (126 MHz, CD_2Cl_2): δ 87.9 (C-1), 73.3 (C-2).
^{19}F NMR (470 MHz, CD_2Cl_2): δ -75.35

Activation of 2,3,4,6-tetra-*O*-acetyl-*α*-D-glucopyranosyl trichloroacetimidate (57) with TMSOTf:

Trichloroacetimidate **57** (57 mg, 0.116 mmol) was dissolved in CD_2Cl_2 (0.75 mL), and the reaction mixture was transferred into a vacuum dried NMR tube and sealed with a septum cap under an Ar atmosphere. The NMR tube containing glycosyl donor solution then was placed into the NMR probe and cooled down to -78 °C. The first ^{1}H, ^{13}C, and DEPT spectra were collected, then the sample was quickly removed from the probe and precooled TMSOTf (23 µL, 0.128 mmol, 1.1 equiv) was quickly added and the tube quickly shaken. The sample then was returned to the cold NMR probe, and ^{1}H, ^{19}F, DEPT spectra were recorded shortly after. The temperature then was increased to -60 °C and ^{1}H, ^{19}F, DEPT spectra were recorded after 5 minutes. After that the temperature was increased by 10 °C increments and ^{1}H, ^{19}F, and DEPT spectra were recorded at each temperature

after 5 minutes. Additionally, at -40° C COSY, HSQC, and HMBC spectra were recorded. After termination of the VT NMR experiment the sample was collected and diluted with CH_2Cl_2 (20 mL). The resulting solution was washed with sat aq $NaHCO_3$ (20 mL), brine (20 mL), dried over $MgSO_4$ and concentrated to dryness. The crude product was purified by a flash column chromatography eluting with hexanes:EtOAc (0→50%, EtOAc) to give mixture containing anomeric acetates **11** (*α : β* = 3 : 1, 12 mg, 27%) and enone **78** (4 mg, 10%) with molar ratio **11** : **78** = 2.56 : 1[4] as yellowish syrup (16 mg) with spectral data identical to that reported in the literature:[34, 35]

1-*N*-trichloroacetamidyl-6-di-*O*-acetyl-1,3,4-trideoxy-*β*-D-glycero-hex-3-enopyranos-2-ulose intermediate (78):

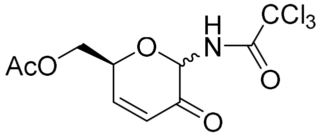

^{1}H NMR (500 MHz, $CDCl_3$): δ 7.47 (d, *J* = 7.5 Hz, 1H, $CCl_3CON\underline{H}$), 7.06 (dd, *J* = 10.5, 3.0 Hz, 1H, H-4), 6.37 – 6.31 (m, 1H, H-3), 5.87 (d, *J* = 7.4 Hz, 1H, H-1), 4.83 (appear s, 1H, H-5), 4.58 (dd, *J* = 12.1, 5.3 Hz, 1H, H-6a), 4.39 (dd, *J* = 12.0, 4.1 Hz, 1H, H-6b), 2.17 (s, 3H), 2.06 (s, 3H).

^{13}C NMR (126 MHz, $CDCl_3$): δ 188.5 (C-2), 170.7, 168.8 (2×$CH_3\underline{C}O$), 162.3 ($CCl_3\underline{C}ONH$), 147.7 (C-4), 127.1 (C-3), 77.6 (C-1), 71.5 (C-5), 63.8 (C-6), 20.5, 20.4 (2×CH_3CO).

ESI-HRMS (*m/z*): $[M+Na]^+$ calcd. for $C_{10}H_{10}O_5NCl_3Na^+$ 351.9517, found 351.9496.

Activation of 2,3,4,6-tetra-*O*-acetyl-5-thio-*α*-D-glucopyranosyl trichloroacetimidate (1) with TMSOTf:

Trichloroacetimidate **1** (63 mg, 0.124 mmol) was dissolved in CD_2Cl_2 (0.75 mL), and the reaction mixture was transferred into a vacuum dried NMR tube and sealed with a septum cap under an Ar atmosphere. The NMR tube containing glycosyl donor solution then was placed into the NMR probe and cooled down to -78 °C. The first ^{1}H, ^{13}C, and DEPT spectra were collected, then the sample was quickly removed from the probe and

[4] Determined spectroscopically

precooled TMSOTf (25 µL, 0.138 mmol, 1.11 equiv) was quickly added and the tube quickly shaken. The sample then was returned to the cold NMR probe, and ^{1}H, ^{19}F, and DEPT spectra were recorded shortly after. The temperature then was increased to -60 °C and ^{1}H, ^{19}F, and DEPT spectra were recorded after 5 minutes. After that the temperature was increased by 10 °C increments and ^{1}H, ^{19}F, and DEPT spectra were recorded at each temperature after 5 minutes. Additionally, at -40° C COSY, HSQC, and HMBC spectra were recorded. After termination of the VT NMR experiment the sample was collected and diluted with CH_2Cl_2 (20 mL). The resulting solution was washed with sat aq $NaHCO_3$ (20 mL), brine (20 mL), dried over $MgSO_4$ and concentrated to dryness. The crude product was purified by a flash column chromatography eluting with hexanes:EtOAc (0→60%, EtOAc) to give α-acetate **9α** as a syrup (8 mg, 16%) with spectral data identical to that reported in the literature.[36, 37]

Activation of (*R*$_S$),(*S*$_S$)-Ethyl 2,3,4,6-tetra-*O*-methyl-1-thio-*β*-D-glucopyranoside-1-*S*-Oxides (53) with Tf_2O:

Glycosyl sulfoxide **53** (27 mg, 0.093 mmol) was dissolved in CD_2Cl_2 (0.75 mL), and the reaction mixture was transferred into a vacuum dried NMR tube and sealed with a septum cap under an Ar atmosphere. The NMR tube containing glycosyl donor solution then was placed into the NMR probe and cooled down to -78 °C. The first ^{1}H, ^{13}C spectra were collected, then the sample was quickly removed from the probe and precooled Tf_2O (17 µL, 0.1021 mmol, 1.1 equiv) was quickly added and the tube quickly shaken. The sample then was returned to the cold NMR probe, and ^{1}H, ^{19}F spectra were recorded shortly after. The temperature then was increased to -60 °C and ^{1}H, ^{19}F spectra were recorded after 5 minutes. After that the temperature was increased by 10 °C increments and ^{1}H, and ^{19}F spectra were recorded at each temperature after 5 minutes. Additionally, at -50 °C ^{13}C, DEPT, COSY, HMQC, and HMBC spectra were recorded. After termination of the VT NMR experiment the sample was collected and diluted with EtOAc (30 mL). The resulting solution was washed with sat aq $NaHCO_3$ (30 mL), brine (30 mL), dried over $MgSO_4$ and concentrated to dryness. The crude product was purified by flash column chromatography eluting with hexanes:EtOAc (0→60%, EtOAc) to give single anomer of pentamethyl

glucose **84** as a syrup (7 mg, 30%) with spectral data identical to that reported in the literature.[9]

^{1}H NMR (500 MHz, $CDCl_3$): δ 4.81 (d, J = 3.6 Hz, 1H), 3.61 (s, 3H), 3.60 – 3.55 (m, 3H), 3.53 (s, 3H), 3.51 – 3.45 (m, 5H), 3.40 (s, 3H), 3.40 (s, 3H), 3.23 – 3.12 (m, 2H).

^{13}C NMR (126 MHz, $CDCl_3$): δ 97.7, 83.6, 81.8, 79.5, 71.2, 69.9, 61.0, 60.5, 59.3, 59.1, 55.2.

ESI-HRMS (*m/z*): $[M+Na]^+$ calcd. for $C_{11}H_{22}O_6Na^+$ 273.1309, found 273.1300.

Activation of Ethyl 2,3,4,6-tetra-*O*-methyl-1-thio-*α*-D-glucopyranoside-1-*S*-oxide (55) with Tf_2O:

Glycosyl sulfoxide **55** (30 mg, 0.101 mmol) was dissolved in CD_2Cl_2 (0.75 mL), and the reaction mixture was transferred into a vacuum dried NMR tube and sealed with a septum cap under an Ar atmosphere. The NMR tube containing glycosyl donor solution then was placed into the NMR probe and cooled down to -78 °C. The first ^{1}H, ^{13}C spectra were collected, then the sample was quickly removed from the probe and precooled Tf_2O (19 μL, 0.113 mmol, 1.11 equiv) was quickly added and the tube quickly shaken. The sample then was returned to the cold NMR probe, and ^{1}H, ^{19}F spectra were recorded shortly after. The temperature then was increased to -60 °C and ^{1}H, ^{19}F spectra were recorded after 5 minutes. After that the temperature was increased by 10 °C increments and ^{1}H, and ^{19}F spectra were recorded at each temperature after 5 minutes. Additionally, at -50 °C and -30 °C ^{13}C, DEPT, COSY, HMQC, and HMBC spectra were recorded. After termination of the VT NMR experiment the sample was collected and diluted with EtOAc (20 mL). The resulting solution was washed with sat aq $NaHCO_3$ (20 mL), brine (20 mL), dried over $MgSO_4$ and concentrated to dryness. The crude product was purified by a flash column chromatography eluting with hexanes:EtOAc (0→60%, EtOAc) to give single anomer of pentamethyl glucose **84** as a syrup (8 mg, 32%) with spectral data identical to presented above.[9]

Diagnostic signals of 1-trifluoromethanesulfonyl-2,3,4,6-tetra-*O*-methyl-*α*-D-glucopyranoside intermediate (82):

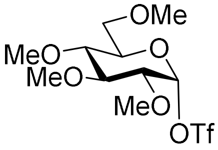

^{1}H NMR (500 MHz, CD_2Cl_2): δ 6.11 (s, 1H, H-1), 3.87 – 2.83 (m, 1H, H-2).
^{13}C NMR (126 MHz, CD_2Cl_2): δ 106.8 (C-1).
^{19}F NMR (470 MHz, CD_2Cl_2): δ -76.13.

Diagnostic signals of 2-methoxy-6-(methoxymethyl)pyrilium trifluoromethanesulfonate (83):

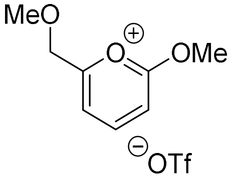

^{1}H NMR (500 MHz, CD_2Cl_2): δ 9.19 (d, *J* = 3.3 Hz, 1H), 8.62 (dd, *J* = 9.4, 3.3 Hz, 1H), 8.26 (d, *J* = 9.2 Hz, 1H).
^{13}C NMR (126 MHz, $CDCl_3$): δ 175.3, 157.1, 152.9, 145.6, 124.0.
ESI-HRMS (*m/z*): $[M]^+$ calcd. for $C_8H_{11}O_3^+$ 155.0703, found 155.0697.

Activation of (*R*$_S$),(*S*$_S$)-Ethyl 2,3,4,6-tetra-*O*-methyl-1,5-dithio-*β*-D-glucopyranoside-1-*S*-Oxides (45) with Tf_2O:

Glycosyl sulfoxide **45** (30 mg, 0.096 mmol) was dissolved in CD_2Cl_2 (0.75 mL), and the reaction mixture was transferred into a vacuum dried NMR tube and sealed with a septum cap under an Ar atmosphere. The NMR tube containing glycosyl donor solution then was placed into the NMR probe and cooled down to -78 °C. The first ^{1}H, ^{13}C spectra were collected, then the sample was quickly removed from the probe and precooled Tf_2O (18 µL, 0.107 mmol, 1.11 equiv) was quickly added and the tube quickly shaken. The sample then was returned to the cold NMR probe, and ^{1}H, ^{19}F spectra were recorded shortly after. The temperature then was increased to -60 °C and ^{1}H, ^{19}F spectra were recorded after 5

minutes. After that the temperature was increased by 10 °C increments and ^{1}H, and ^{19}F spectra were recorded at each temperature after 5 minutes. Additionally, at -50 °C and -30 °C ^{13}C, DEPT, COSY, HMQC, and HMBC spectra were recorded. After termination of the VT NMR experiment the sample was collected and diluted with EtOAc (20 mL). The resulting solution was washed with sat aq $NaHCO_3$ (20 mL), brine (20 mL), dried over $MgSO_4$ and concentrated to dryness. The crude product was purified by a flash column chromatography eluting with hexanes:EtOAc (0→60%, EtOAc) to give thiophene **90** as a syrup (6 mg, 34%).

FTIR ($CHCl_3$) cm^{-1}: 2924.5, 1642.5, 1549.0, 1461.3, 1396.2.

^{1}H NMR (500 MHz, $CDCl_3$): δ 9.94 (s, 1H, C<u>H</u>O), 6.79 (s, 1H, Aryl C<u>H</u>), 4.54 (s, 2H, C<u>H</u>$_2$OCH$_3$), 3.95 (s, 3H, OC<u>H</u>$_3$), 3.42 (s, 3H, OC<u>H</u>$_3$).

^{13}C NMR (126 MHz, $CDCl_3$): δ 181.0 (CHO), 164.5 (q<u>C</u>-OMe), 151.7 (<u>qC</u>-CH$_2$OCH$_3$), 120.6 (<u>qC</u>-CHO), 114.0 (Aryl CH), 69.9, 58.7 (2×OCH$_3$).

ESI-HRMS (*m/z*): $[M+H]^+$ calcd. for $C_8H_{11}O_3S^+$ 187.0423, found 187.0434.

Activation of Ethyl 2,3,4,6-tetra-*O*-methyl-1,5-dithio-*α*-D-glucopyranoside-1-*S*-Oxides (48) with Tf_2O:

Glycosyl sulfoxide **48** (30 mg, 0.096 mmol) was dissolved in CD_2Cl_2 (0.75 mL), and the reaction mixture was transferred into a vacuum dried NMR tube and sealed with a septum cap under an Ar atmosphere. The NMR tube containing glycosyl donor solution then was placed into the NMR probe and cooled down to -78 °C. The first ^{1}H, ^{13}C spectra were collected, then the sample was quickly removed from the probe and precooled Tf_2O (18 µL, 0.107 mmol, 1.11 equiv) was quickly added and the tube quickly shaken. The sample then was returned to the cold NMR probe, and ^{1}H, ^{19}F spectra were recorded shortly after. The temperature then was increased to -60 °C and ^{1}H, ^{19}F spectra were recorded after 5 minutes. After that the temperature was increased by 10 °C increments and ^{1}H, and ^{19}F spectra were recorded at each temperature after 5 minutes. Additionally, at -50 °C and -30 °C ^{13}C, DEPT, COSY, HMQC, and HMBC spectra were recorded. After termination of the VT NMR experiment the sample was collected and diluted with EtOAc (30 mL). The resulting solution was washed with sat aq $NaHCO_3$ (30 mL), brine (30 mL), dried over $MgSO_4$ and concentrated to dryness. The crude product was purified by flash column

chromatography eluting with hexanes:EtOAc (0→60%, EtOAc) to give thiophene **90** as a syrup (5 mg, 28%) with spectral data identical to that presented above.

Activation of 2,3,4,6-tetra-*O*-methyl-*α,β*-D-glucopyranosyl trichloroacetimidate (60) with TMSOTf:

Trichloroacetimidate **60** (20 mg, 0.053 mmol) was dissolved in CD_2Cl_2 (0.75 mL), and the reaction mixture was transferred into a vacuum dried NMR tube and sealed with a septum cap under an Ar atmosphere. The NMR tube containing glycosyl donor solution then was placed into the NMR probe and cooled down to -78 °C. The first ^1H, ^{13}C, and DEPT spectra were collected, then the sample was quickly removed from the probe and precooled TMSOTf (10 µL, 0.0583 mmol, 1.1 equiv) was quickly added and the tube quickly shaken. The sample then was returned to the cold NMR probe, and ^1H, ^{19}F, DEPT spectra were recorded shortly after. The temperature then was increased to -60 °C and ^1H, ^{19}F, DEPT spectra were recorded after 5 minutes. After that the temperature was increased by 10 °C increments and ^1H, ^{19}F, and DEPT spectra were recorded at each temperature after 5 minutes. Additionally, at -40° C COSY, HSQC, and HMBC spectra were recorded. After termination of the VT NMR experiment the sample was collected and diluted with EtOAc (20 mL). The resulting solution was washed with sat aq $NaHCO_3$ (20 mL), brine (20 mL), dried over $MgSO_4$ and concentrated to dryness. The crude product was purified by a flash column chromatography eluting with hexanes:EtOAc (0→50%, EtOAc) to give a mixture of anomeric amides **92** as a syrup (5 mg, 25%).

α* : *β = 4.8 : 1

$\boldsymbol{R_f}$ 0.42 (hexanes:EtOAc 1:1 (H_2SO_4/EtOH)).

^{1}H NMR (500 MHz, $CDCl_3$): δ 7.19 (d, *J* = 5.9 Hz, 1H, NH), 5.65 (t, *J* = 5.7 Hz, 1H, H-1), 3.62 (s, 3H, OCH_3), 3.60 – 3.54 (m, 2H, H-6a, H-6b), 3.53 (s, 3H, OCH_3), 3.52 – 3.45 (m, 2H, H-2, H-5), 3.43 (s, 3H, OCH_3), 3.38 (s, 3H, OCH_3), 3.33 (t, *J* = 9.0 Hz, 1H, H-4), 3.20 (t, *J* = 8.7 Hz, 1H, H-3).

^{13}C NMR (126 MHz, $CDCl_3$): δ 162.2 (CO), 92.4 (CCl_3), 83.2 (C-3), 79.1 (C-2), 78.1 (C-4), 76.4 (C-1), 71.6 (C-5), 70.4 (C-6), 60.8, 60.4, 59.2, 58.3 (4×OCH_3).

The minor *β*-isomer was identified by characteristic signals:

^{1}H NMR (500 MHz, $CDCl_3$): δ 7.06 (d, *J* = 9.3 Hz, 1H, NH), 4.97 (t, *J* = 9.1 Hz, 1H, H-1), 3.64 (s, 3H, OCH_3), 3.53 (s, 3H, OCH_3), 3.39 (s, 3H, OCH_3), 3.30 – 3.26 (m, 1H, H-4), 3.04 (t, *J* = 8.6 Hz, 1H, H-3).

^{13}C NMR (126 MHz, $CDCl_3$): δ 162.2 (CO), 92.4 (CCl_3), 86.9 (C-3), 83.3 (C-2), 80.9 (C-1), 78.6 (C-4), 60.6 (OCH_3), 60.4 (OCH_3).

ESI-HRMS (m/z): $[M+Na]^+$calcd. for $C_{12}H_{20}O_6NCl_3Na^+$ 402.0248, found 402.0237.

Activation of 2,3,4,6-tetra-*O*-methyl-*α,β*-D-glucopyranosyl trichloroacetimidate (64) with TMSOTf:

Trichloroacetimidate **64** (25 mg, 0.063 mmol) was dissolved in CD_2Cl_2 (0.75 mL), and the reaction mixture was transferred into a vacuum dried NMR tube and sealed with a septum cap under an Ar atmosphere. The NMR tube containing glycosyl donor solution then was placed into the NMR probe and cooled down to -78 °C. The first ^{1}H, ^{13}C, and DEPT spectra were collected, then the sample was quickly removed from the probe and precooled TMSOTf (13 µL, 0.0718 mmol, 1.14 equiv) was quickly added and the tube quickly shaken. The sample then was returned to the cold NMR probe, and ^{1}H, ^{19}F, DEPT spectra were recorded shortly after. The temperature then was increased to -60 °C and ^{1}H, ^{19}F, DEPT spectra were recorded after 5 minutes. After that the temperature was increased by 10 °C increments and ^{1}H, ^{19}F, and DEPT spectra were recorded at each temperature after 5 minutes. Additionally, at -30° C COSY, HSQC, and HMBC spectra were recorded. After termination of the VT NMR experiment the sample was collected and diluted with EtOAc (20 mL). The resulting solution was washed with sat aq $NaHCO_3$ (20 mL), brine (20 mL), dried over $MgSO_4$ and concentrated to dryness. The crude product was purified by flash column chromatography eluting with hexanes:EtOAc (0→50%, EtOAc) to give a mixture of anomeric amides **94** as a syrup (10 mg, 41%).

α* : *β = 3.3 : 1

$\boldsymbol{R_f}$ 0.37 and 0.29 (hexanes:EtOAc 3:2 (H_2SO_4/EtOH)).

^{1}H NMR (500 MHz, $CDCl_3$): δ 7.70 (d, *J* = 9.1 Hz, 1H, NH), 5.45 (dd, *J* = 9.2, 4.8 Hz, 1H, H-1), 3.98 – 3.94 (m, 1H, H-2), 3.94 – 3.87 (m, 1H, H-3), 3.55 (s, 3H, OCH_3), 3.51 (dd, *J* = 5.0, 3.7 Hz, 2H, H-6a, H-6b), 3.48 – 3.26 (m, 11H, H-4, H-5, 3×OCH_3).

^{13}C NMR (126 MHz, CDCl$_3$): δ 161.2 (CO), 92.6 (CCl$_3$), 89.6 (C-2), 82.9 (C-3), 81.4 (C-4), 72.2 (C-6), 59.6, 59.4, 58.5, 57.9 (4×OCH$_3$), 56.3 (C-1), 48.8 (C-5).

The minor *β*-isomer was identified by characteristic signals:

^{1}H NMR (500 MHz, CDCl$_3$): δ 7.82 (d, *J* = 9.5 Hz, 1H, NH), 5.55 (d, *J* = 9.5 Hz, 1H, H-1), 4.20 (appear s, 1H, H-4), 3.98 – 3.94 (m, 1H, H-2), 3.94 – 3.87 (m, 1H, H-3), 3.79 (d, *J* = 10.8 Hz, 1H, H-5), 3.65 – 3.58 (m, 2H, H-6a, H-6b), 3.55 (s, 4H).

^{13}C NMR (126 MHz, CDCl$_3$): δ 161.2 (CO), 92.6 (CCl$_3$), 87.3 (C-3), 70.7 (C-6), 62.6 (C-1), 59.4, 57.8, 57.8, 57.5 (4×OCH$_3$), 53.0 (C-5).

ESI-HRMS (m/z): $[M+Na]^+$ calcd. for $C_{12}H_{20}O_5NCl_3NaS^+$ 418.0020, found 418.0015

3.3. Summary of VT NMR experiments with disarmed glucosyl and 5-thioglucosyl donors

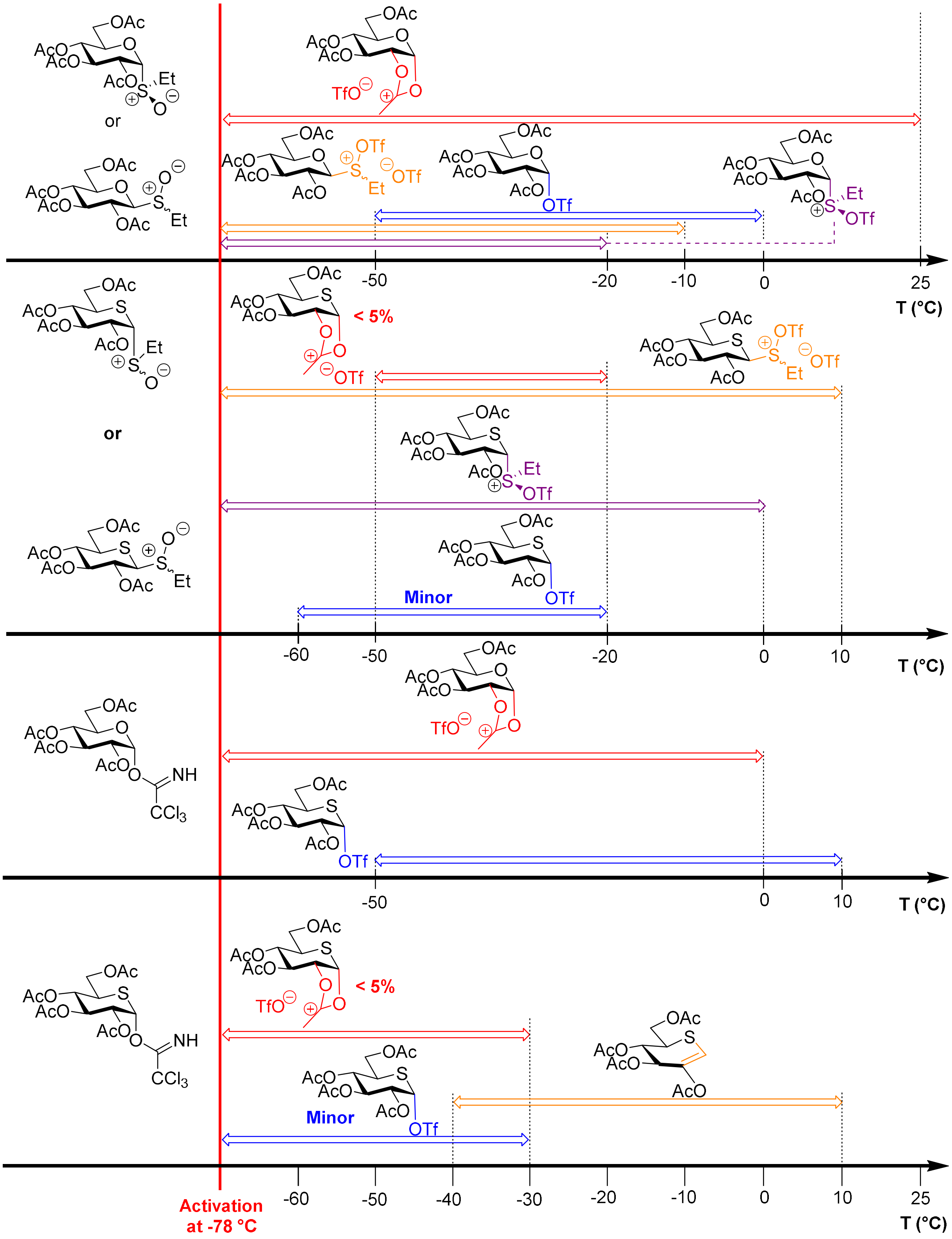

3.4. Summary of VT NMR experiments with armed glucosyl and 5-thioglucosyl donors

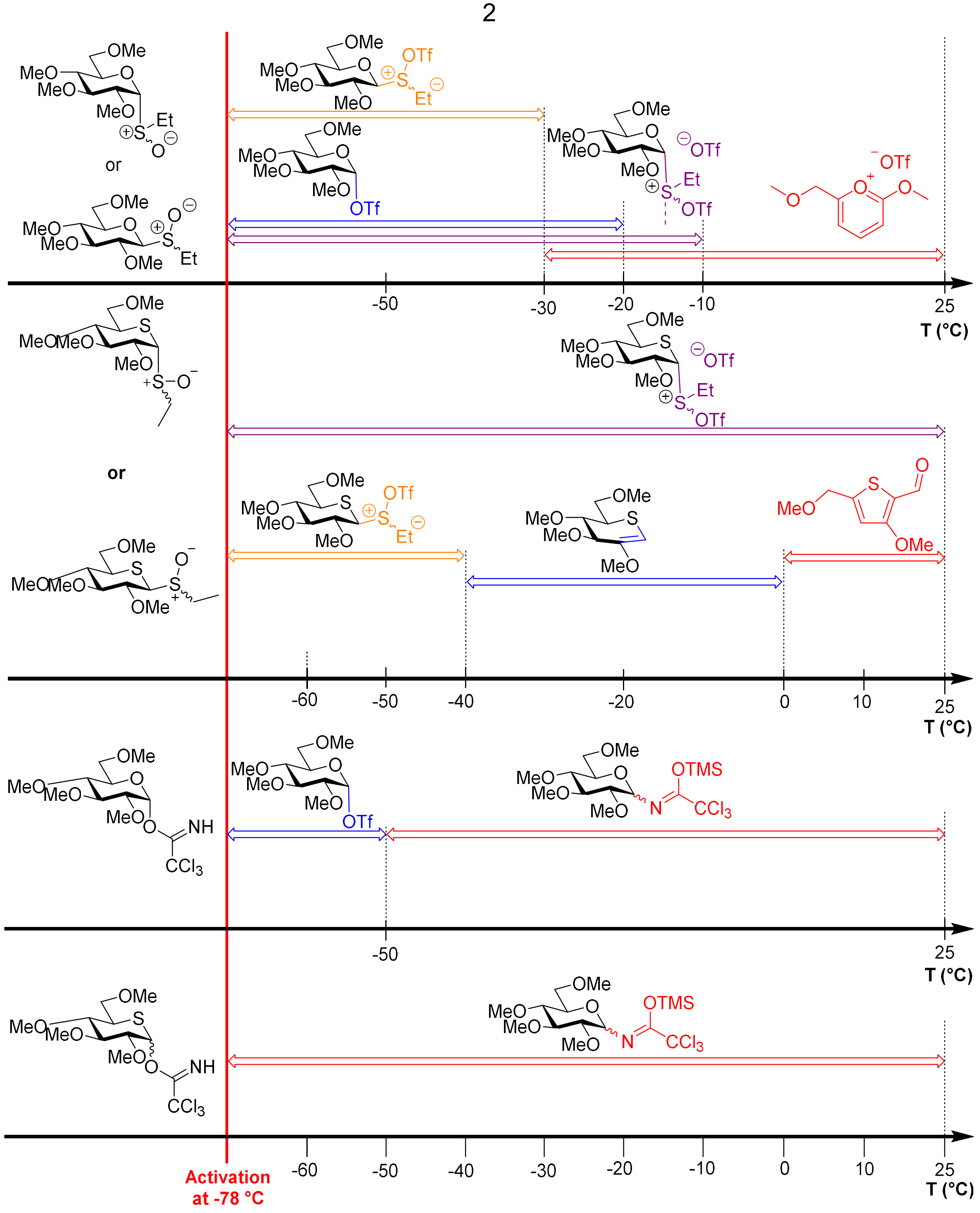

3.5. VT NMR spectra from experiments with 2,3-dihydro-4*H*-thiopyran (34) and 2,3-dihydro-4*H*-pyran (S1).

Stacked ^{1}H NMR (500 MHz, CD_2Cl_2) spectra of reaction mixture after protonation of 2,3-dihydro-4*H*-thiopyran (34) with 2.2 equiv of TfOH at -88 °C

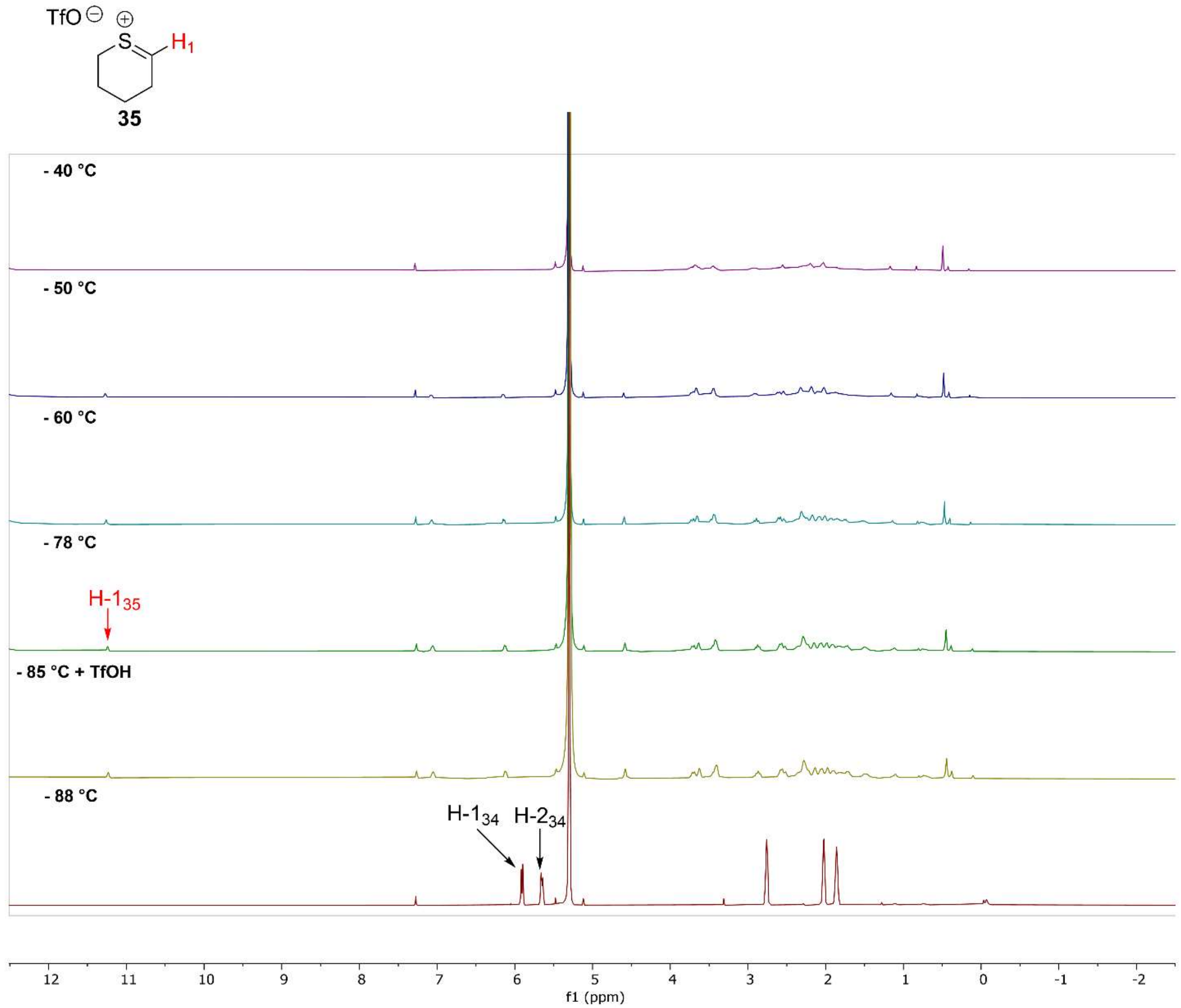

Stacked ^{19}F NMR (470 MHz, CD_2Cl_2) spectra of reaction mixture after protonation of 2,3-dihydro-4H-thiopyran (34) with 2.2 equiv of TfOH at -88 °C

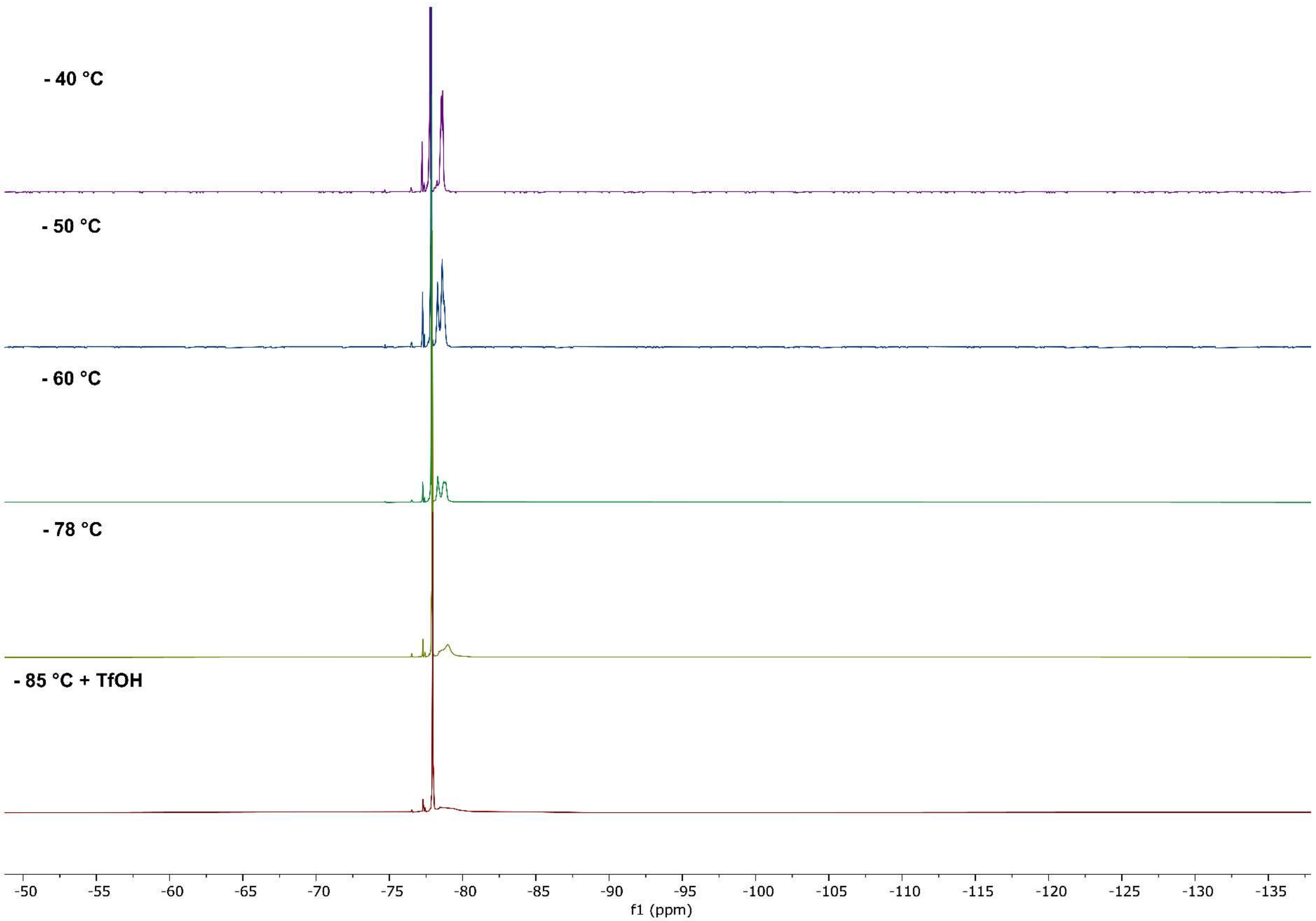

^{1}H NMR (500 MHz, CD_2Cl_2) spectrum of reaction mixture at -85 °C after protonation of 2,3-dihydro-4*H*-thiopyran (34) with 2.2 equiv of TfOH

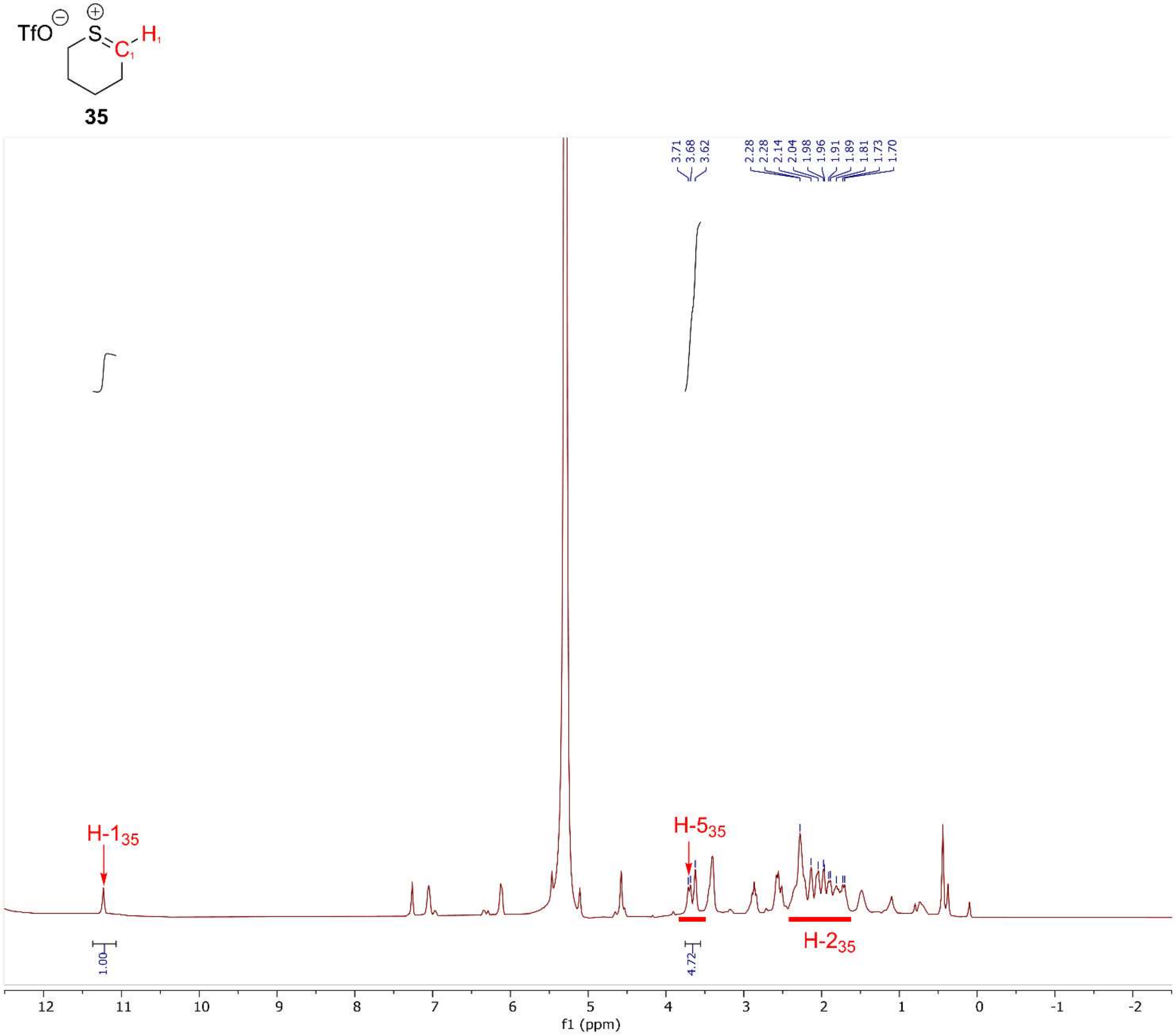

DEPT-135 (CD_2Cl_2) spectrum of reaction mixture at -85 °C after protonation of 2,3-dihydro-4*H*-thiopyran (34) with 2.2 equiv of TfOH

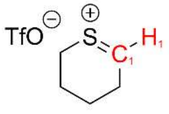

35

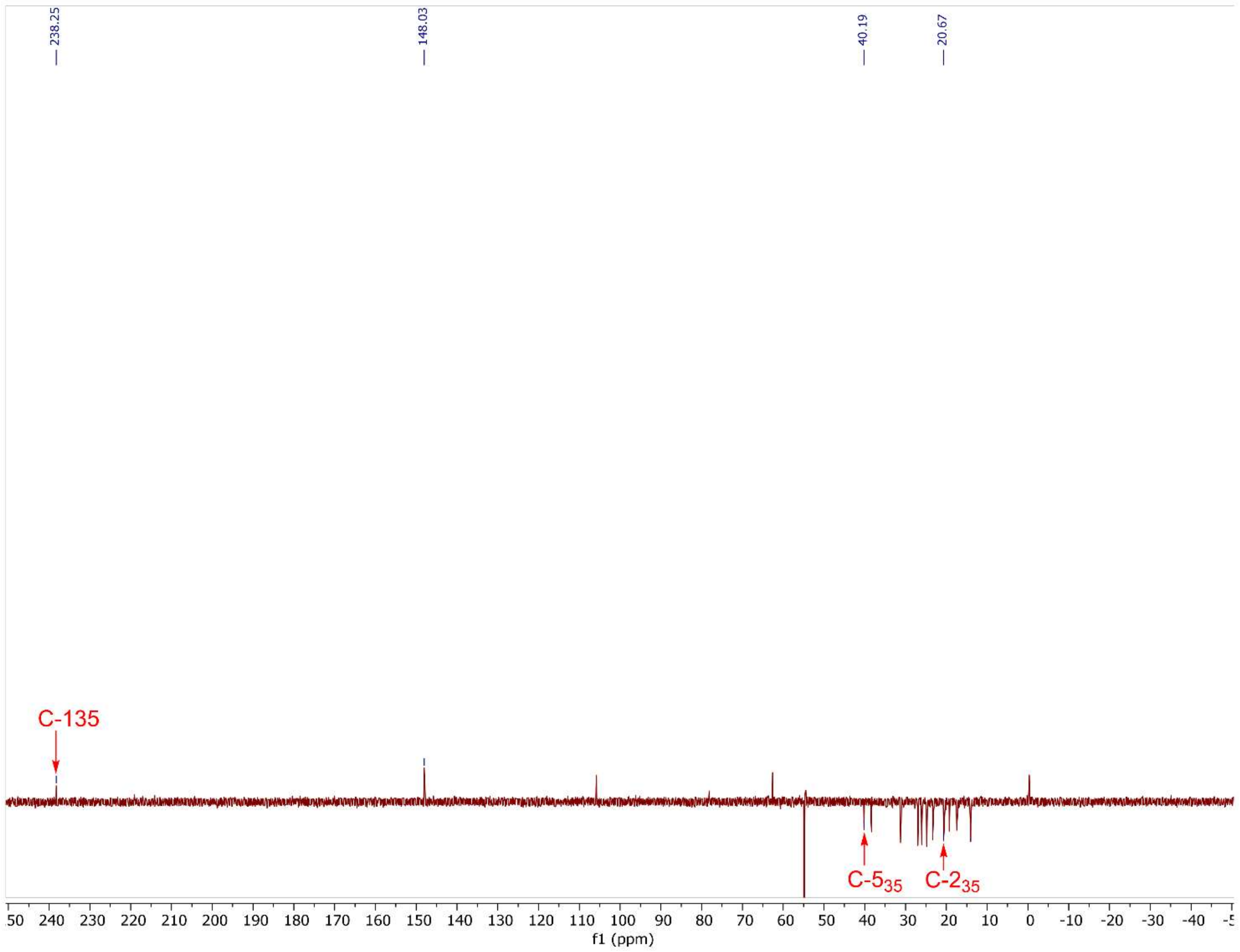

HMQC (CD_2Cl_2) spectrum of reaction mixture at -85 °C after protonation of 2,3-dihydro-4*H*-thiopyran (34) with 2.2 equiv of TfOH

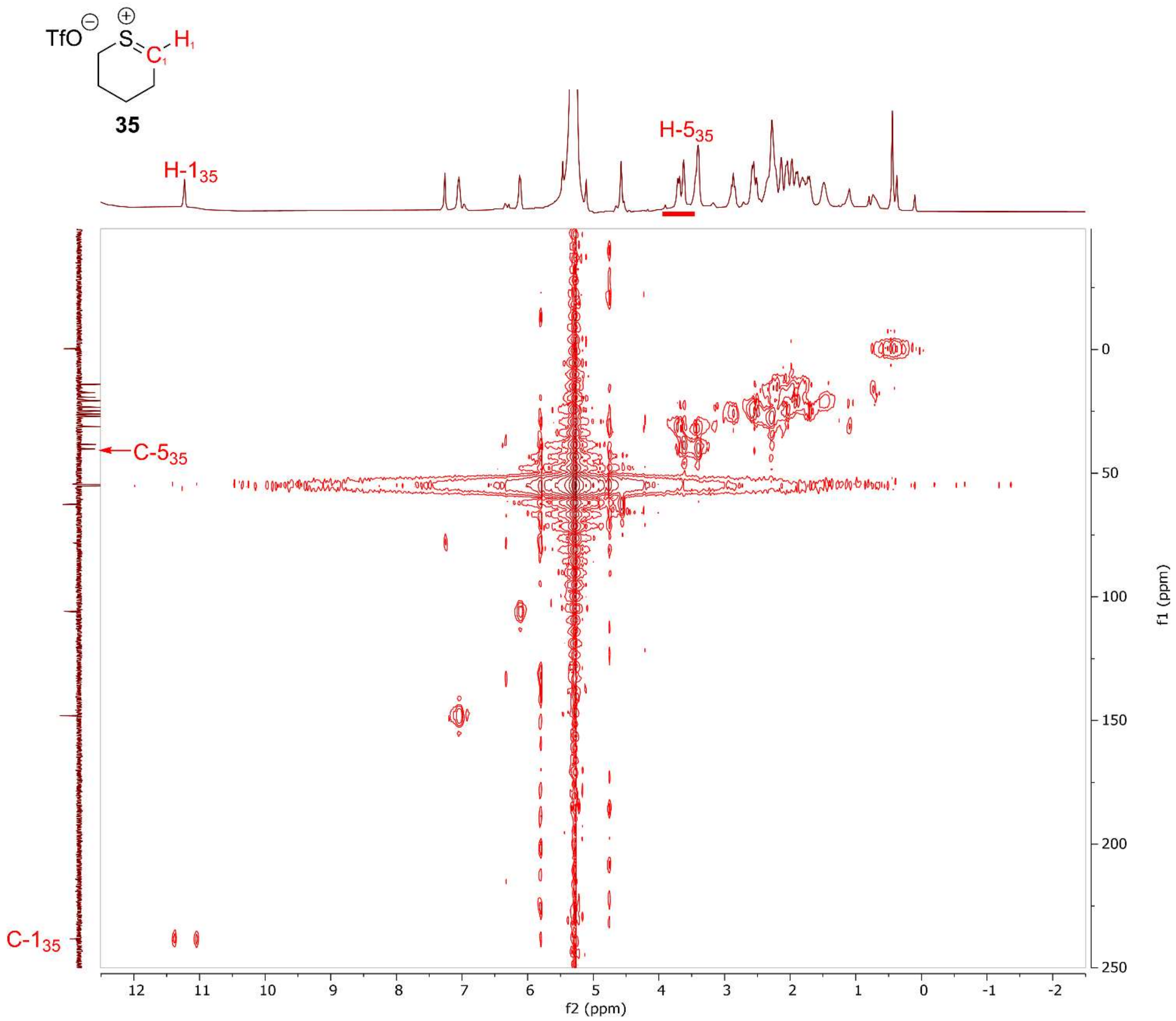

HMBC (CD_2Cl_2) spectrum of reaction mixture at -85 °C after protonation of 2,3-dihydro-4*H*-thiopyran (34) with 2.2 equiv of TfOH

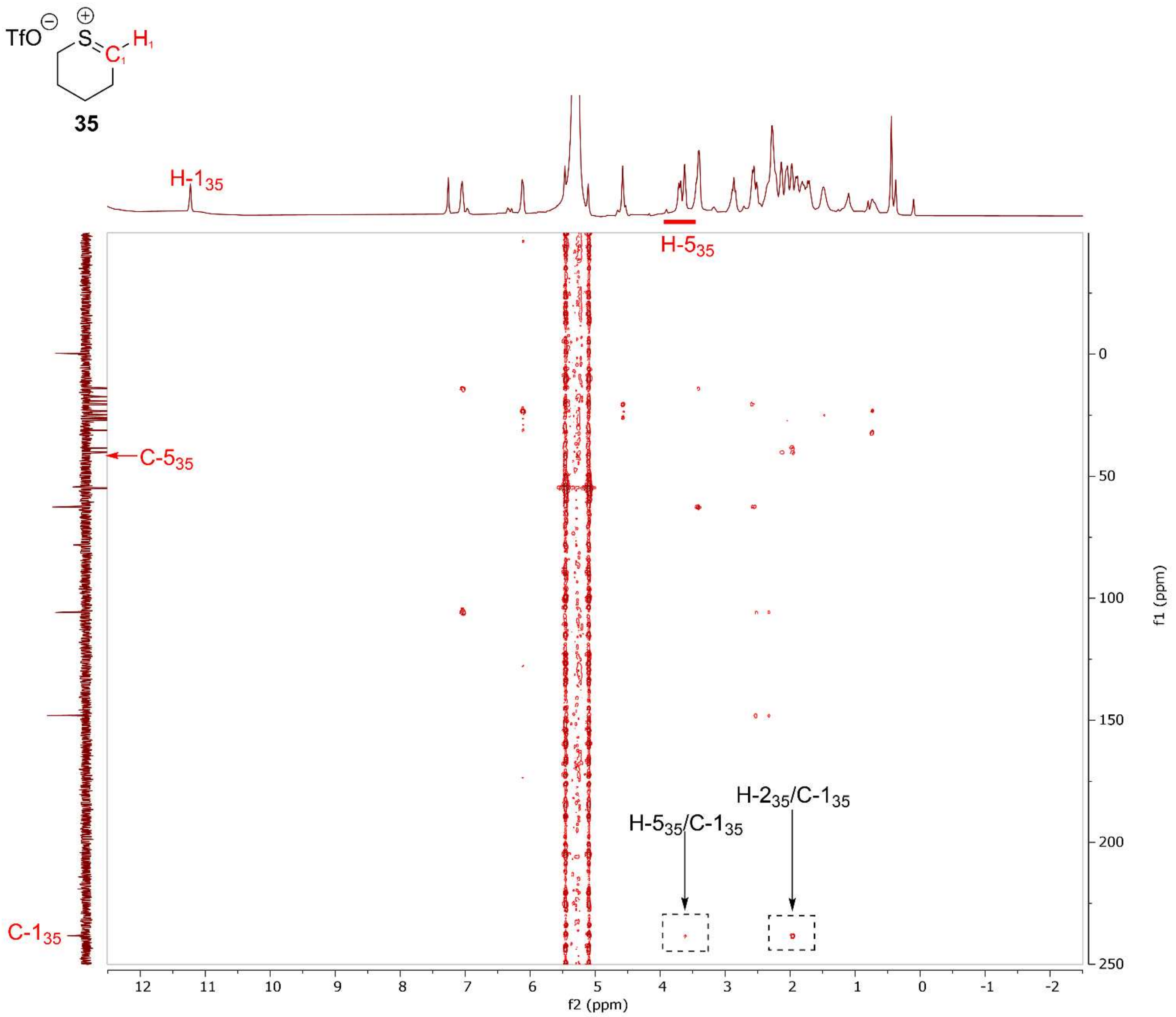

^{19}F NMR (470 MHz, CD_2Cl_2) spectrum of reaction mixture at -85 °C after protonation of 2,3-dihydro-4*H*-thiopyran (34) with 2.2 equiv of TfOH

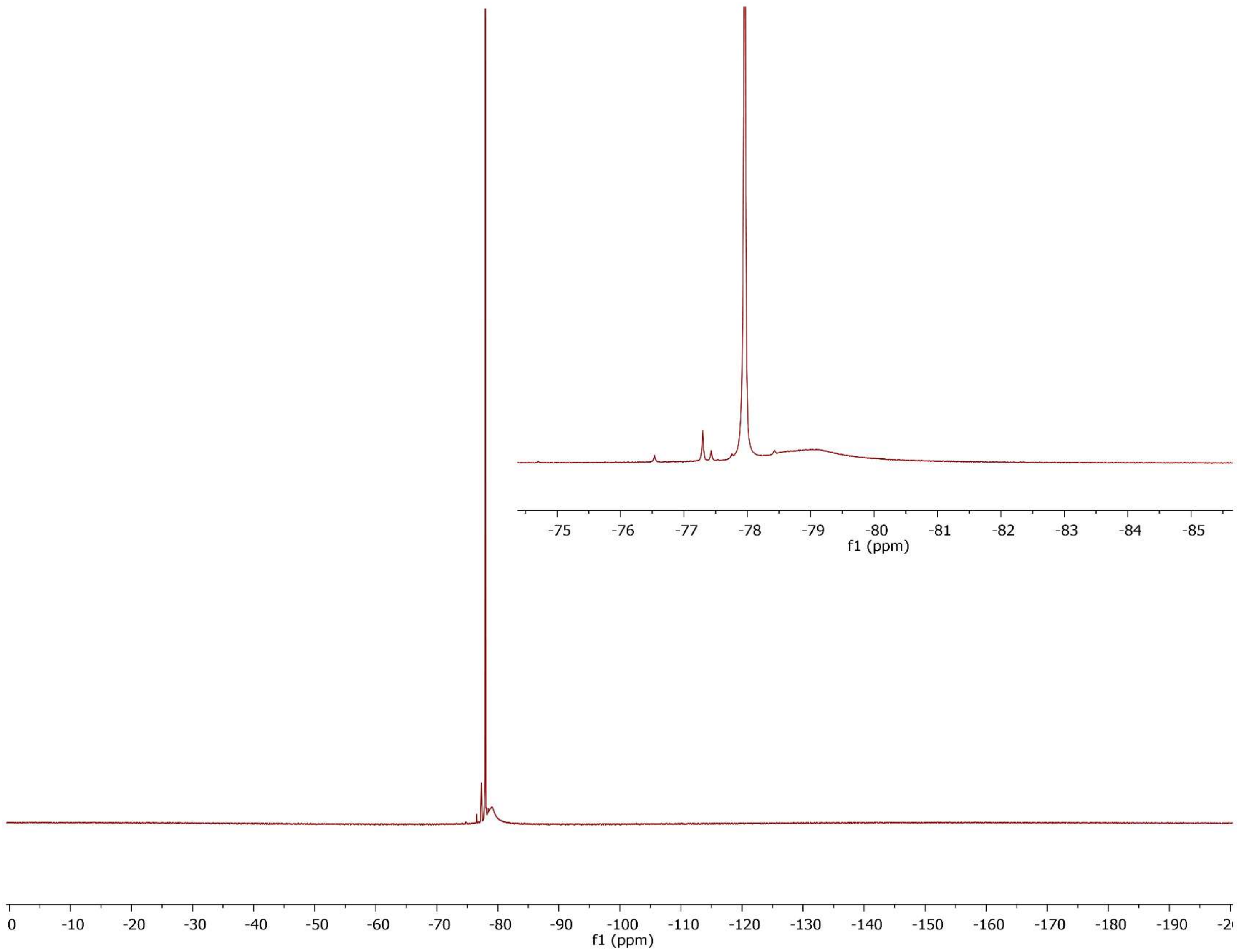

Stacked ^{1}H NMR (500 MHz, CD_2Cl_2) spectra of reaction mixture after protonation of 2,3-dihydro-4*H*-thiopyran (34) with 1.1 equiv of TfOH at -78 °C in presence of Bu_4NPF_6

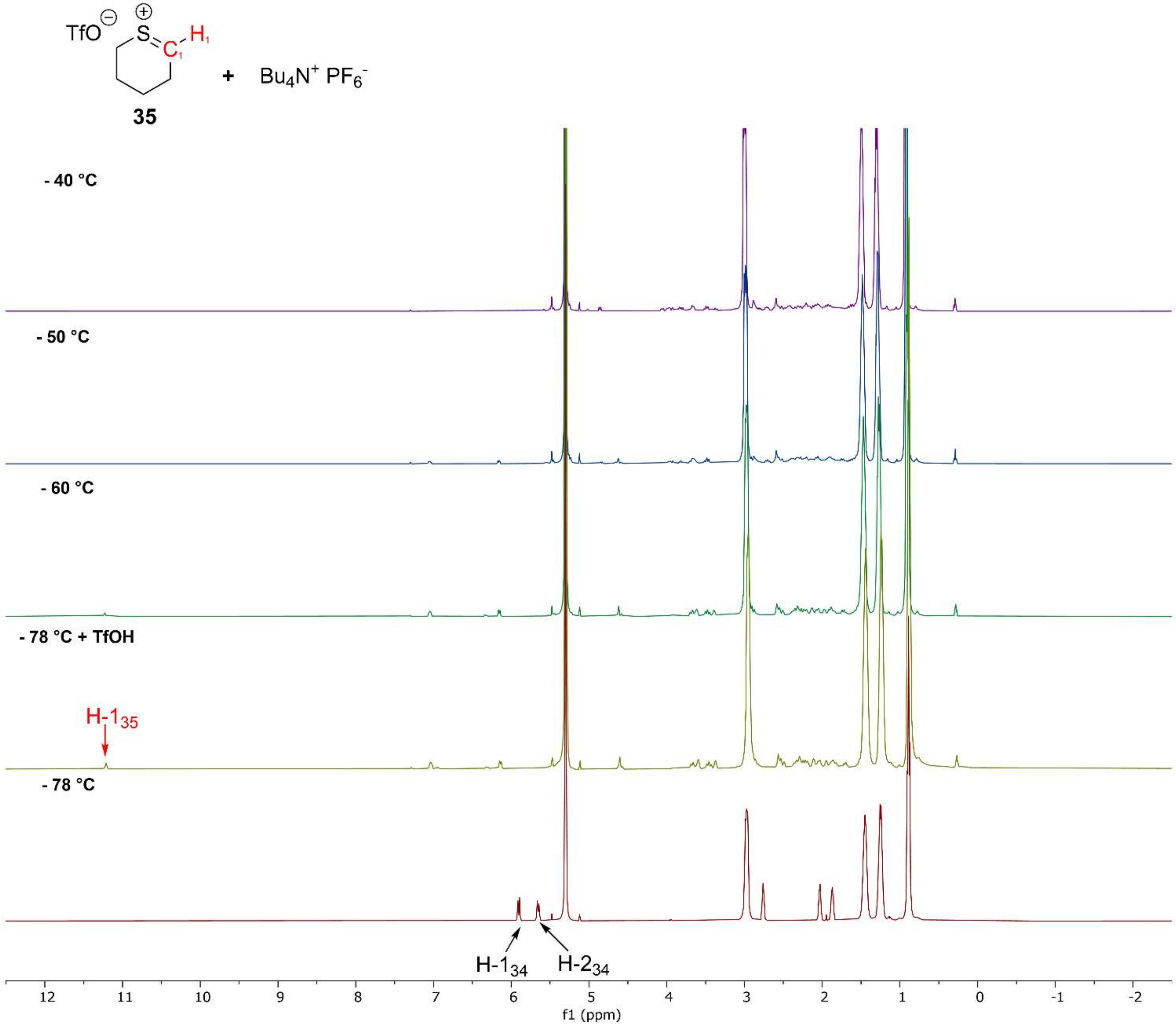

Stacked ^{19}F NMR (470 MHz, CD_2Cl_2) spectra of reaction mixture after protonation of 2,3-dihydro-*4H*-thiopyran (34) with 1.1 equiv of TfOH at -78 °C in presence of Bu_4NPF_6

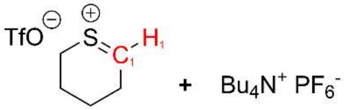

35

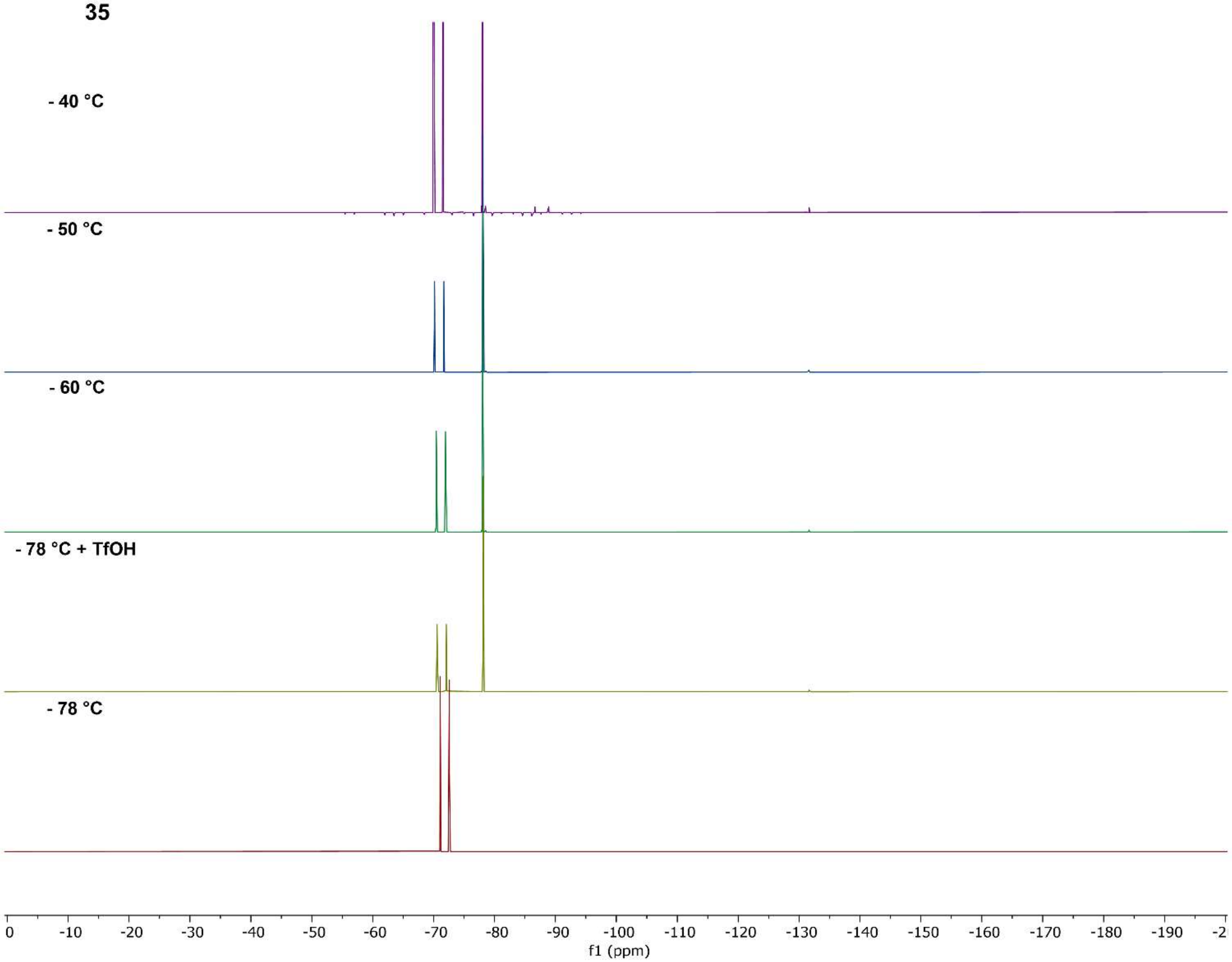

^{1}H NMR (500 MHz, CD_2Cl_2) spectrum of reaction mixture at -78 °C after protonation of 2,3-dihydro-4*H*-thiopyran (34) mixed with 1.1 equiv of TfOH in presence of Bu_4NPF_6

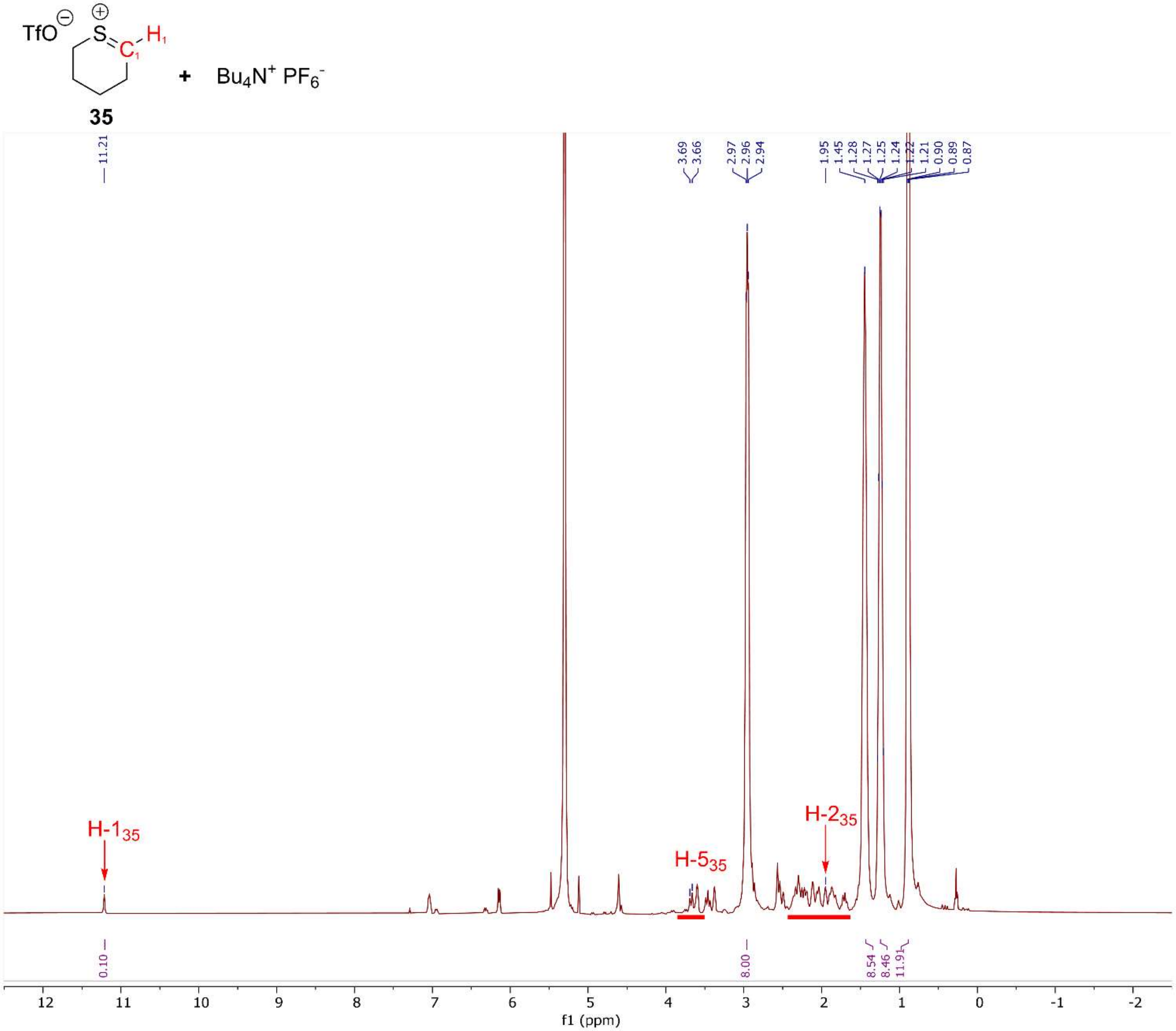

DEPT-135 (CD_2Cl_2) spectrum of reaction mixture at -78 °C after protonation of 2,3-dihydro-4*H*-thiopyran (34) mixed with 1.1 equiv of TfOH in presence of Bu_4NPF_6

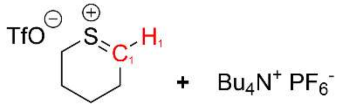

35

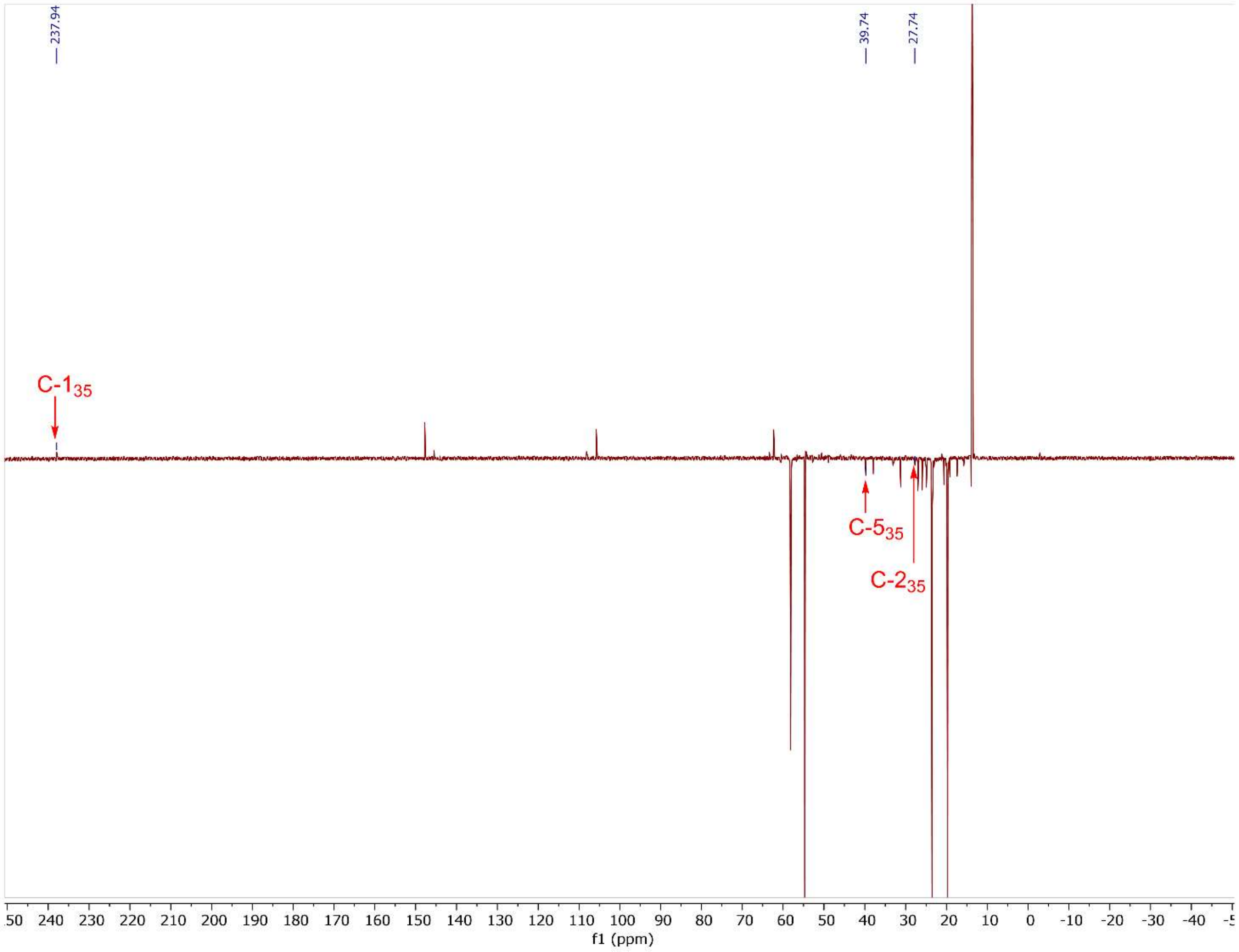

HMQC (CD_2Cl_2) spectrum of reaction mixture at -78 °C after protonation of 2,3-dihydro-4*H*-thiopyran (34) mixed with 1.1 equiv of TfOH in presence of Bu_4NPF_6

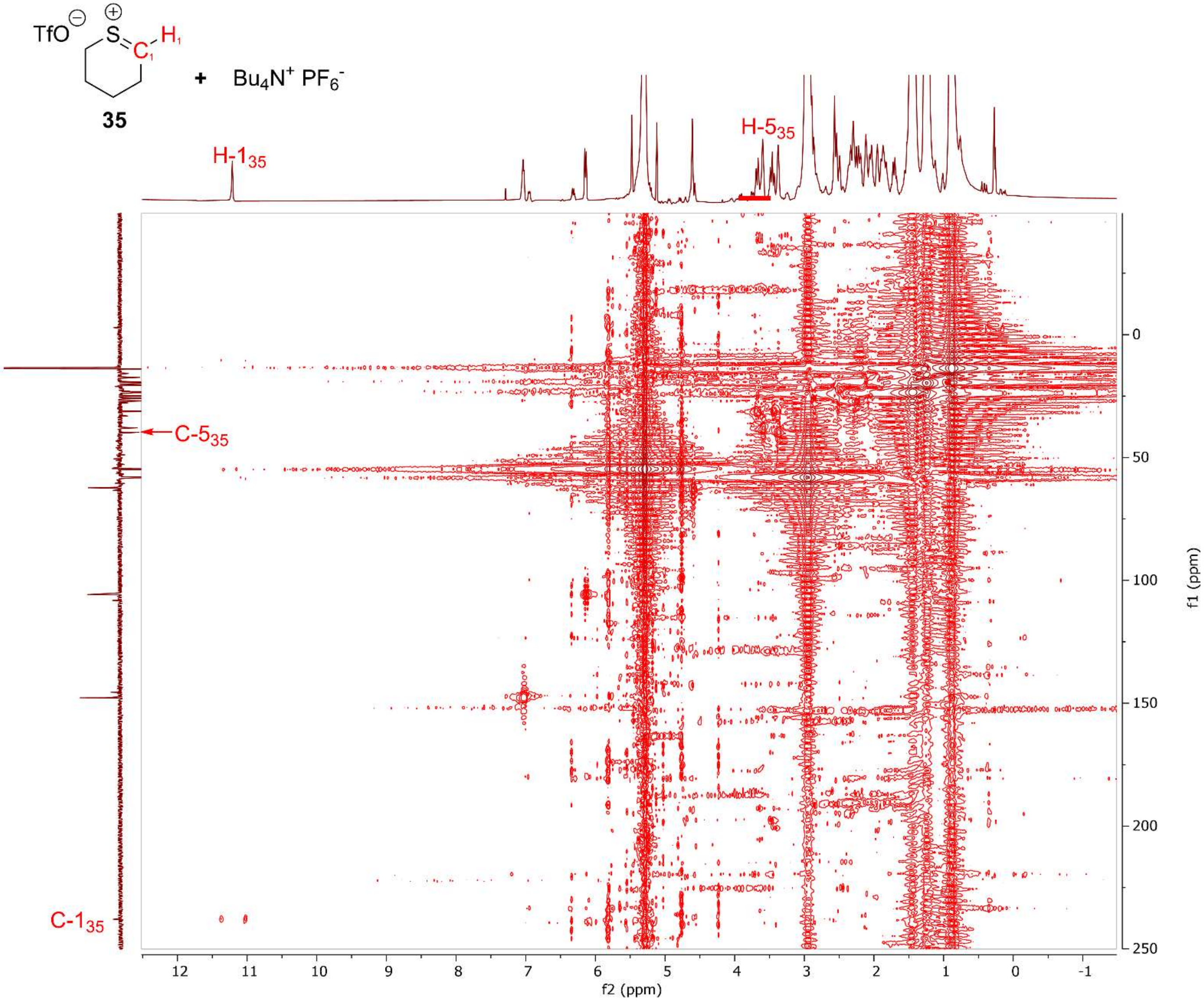

^{19}F NMR (470 MHz, CD_2Cl_2) spectrum of reaction mixture at -78 °C after protonation of 2,3-dihydro-4*H*-thiopyran (34) with 1.1 equiv of TfOH in presence of Bu_4NPF_6

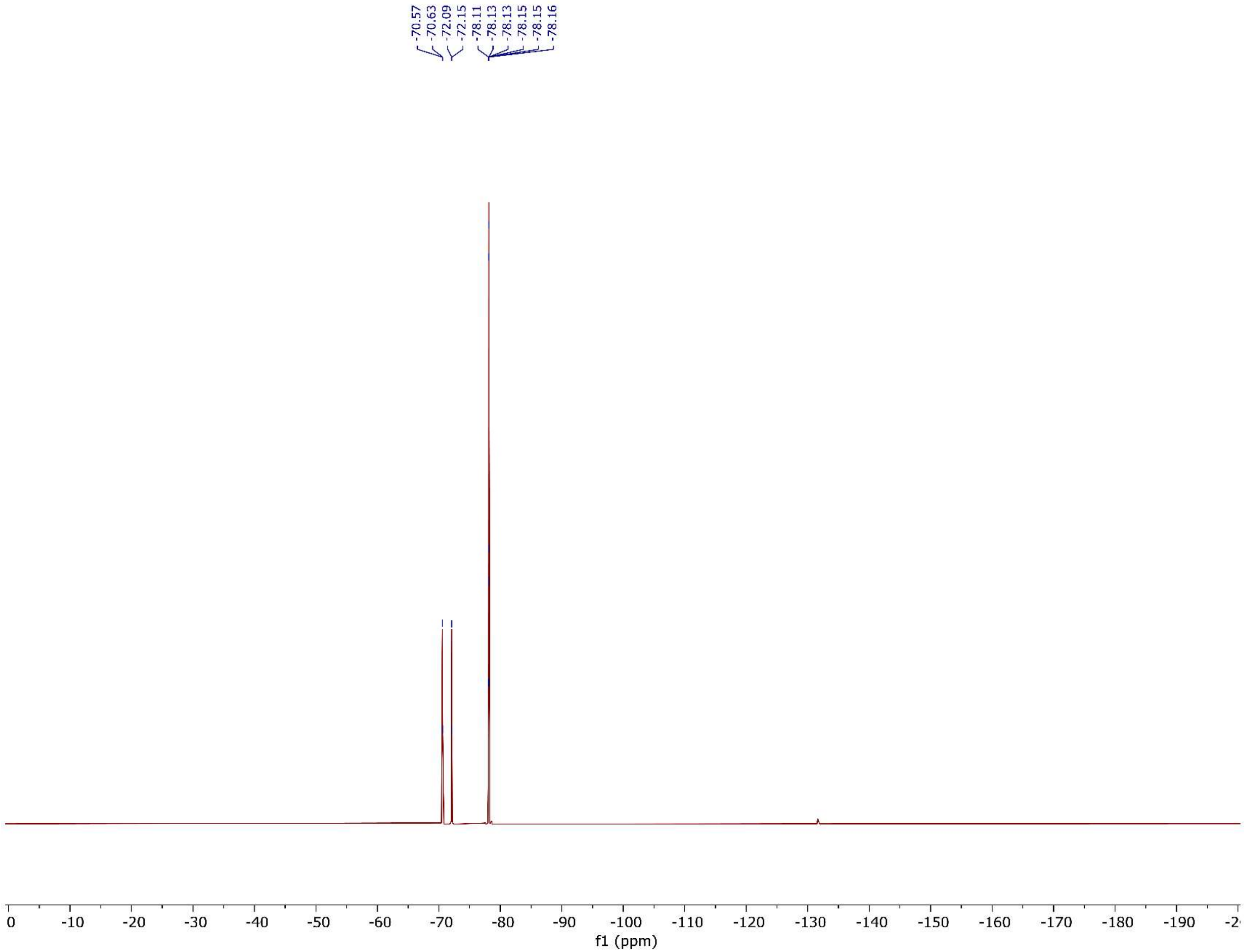

Stacked ^{1}H NMR (500 MHz, CD_2Cl_2) spectra of reaction mixture after protonation of 2,3-dihydro-4*H*-pyran (S1) with 2.2 equiv of TfOH at -88 °C

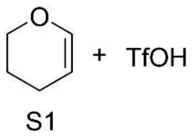

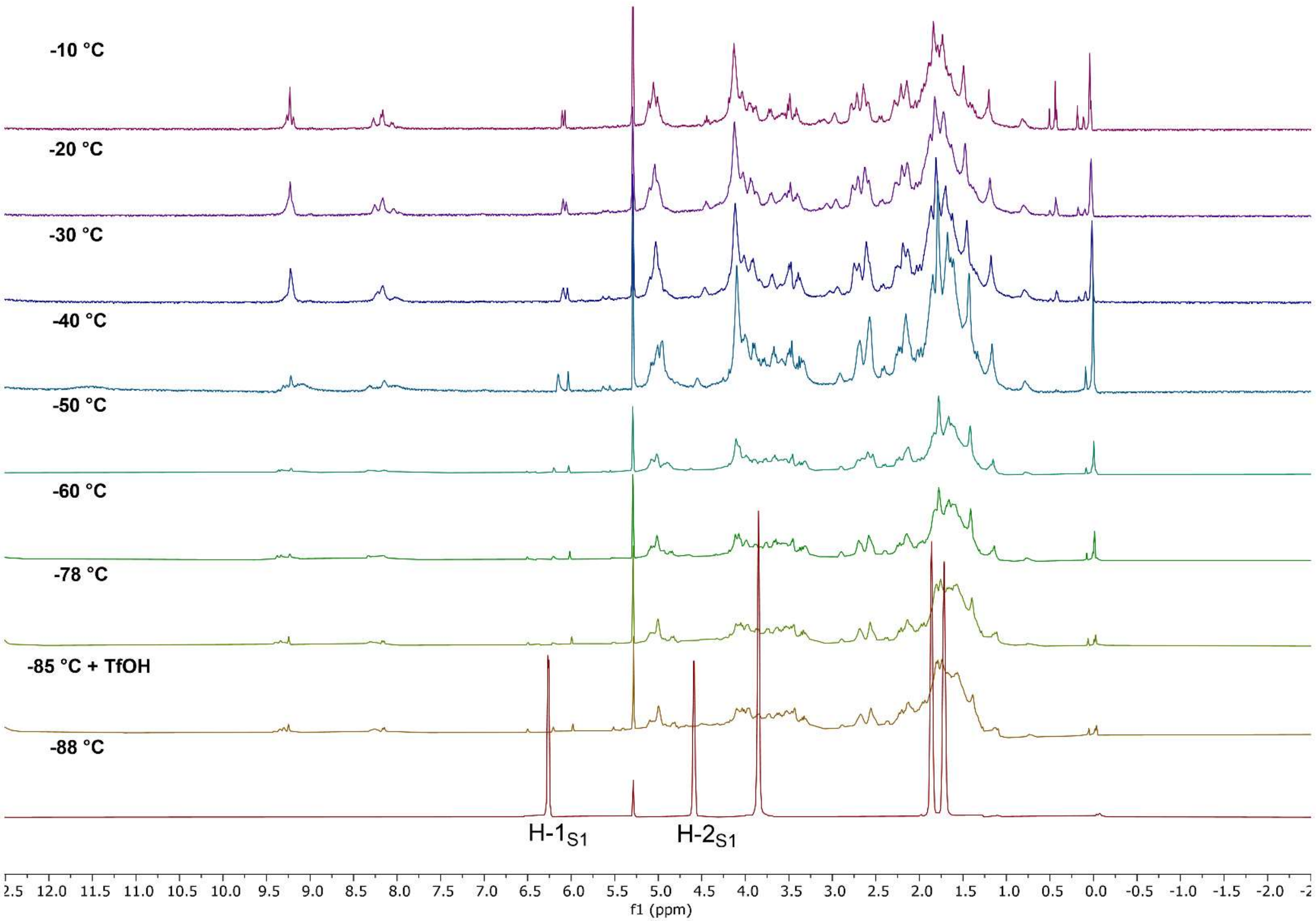

Stacked ^{19}F NMR (470 MHz, CD_2Cl_2) spectra of reaction mixture after protonation of 2,3-dihydro-4*H*-pyran (S1) with 2.2 equiv of TfOH at -88 °C

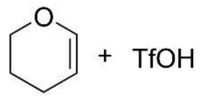

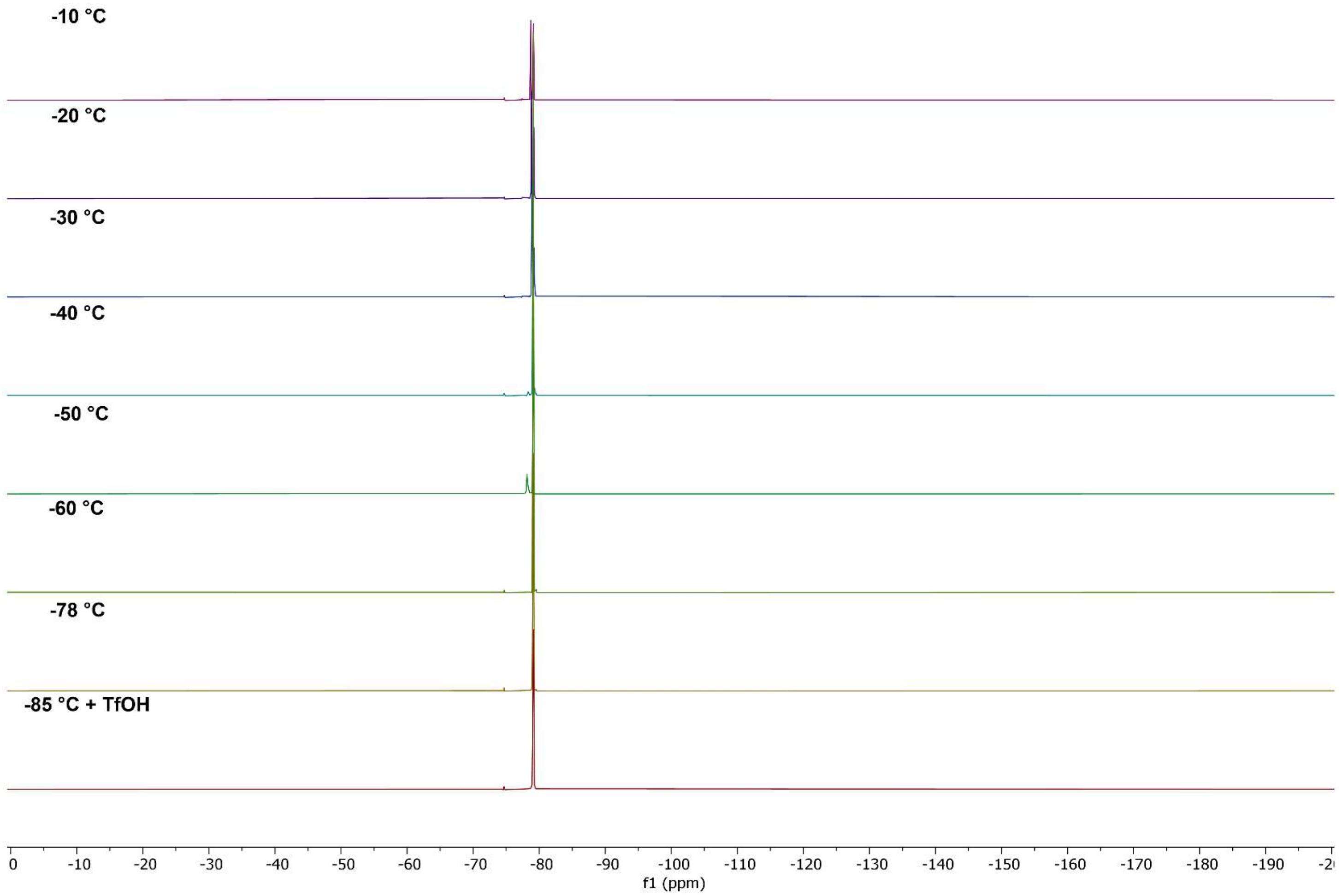

Stacked ^{1}H NMR (500 MHz, CD_2Cl_2) spectra of reaction mixture after protonation of 2,3-dihydro-4*H*-pyran (S1) with 1.1 equiv of TfOH at -78 °C in presence of Bu_4NPF_6

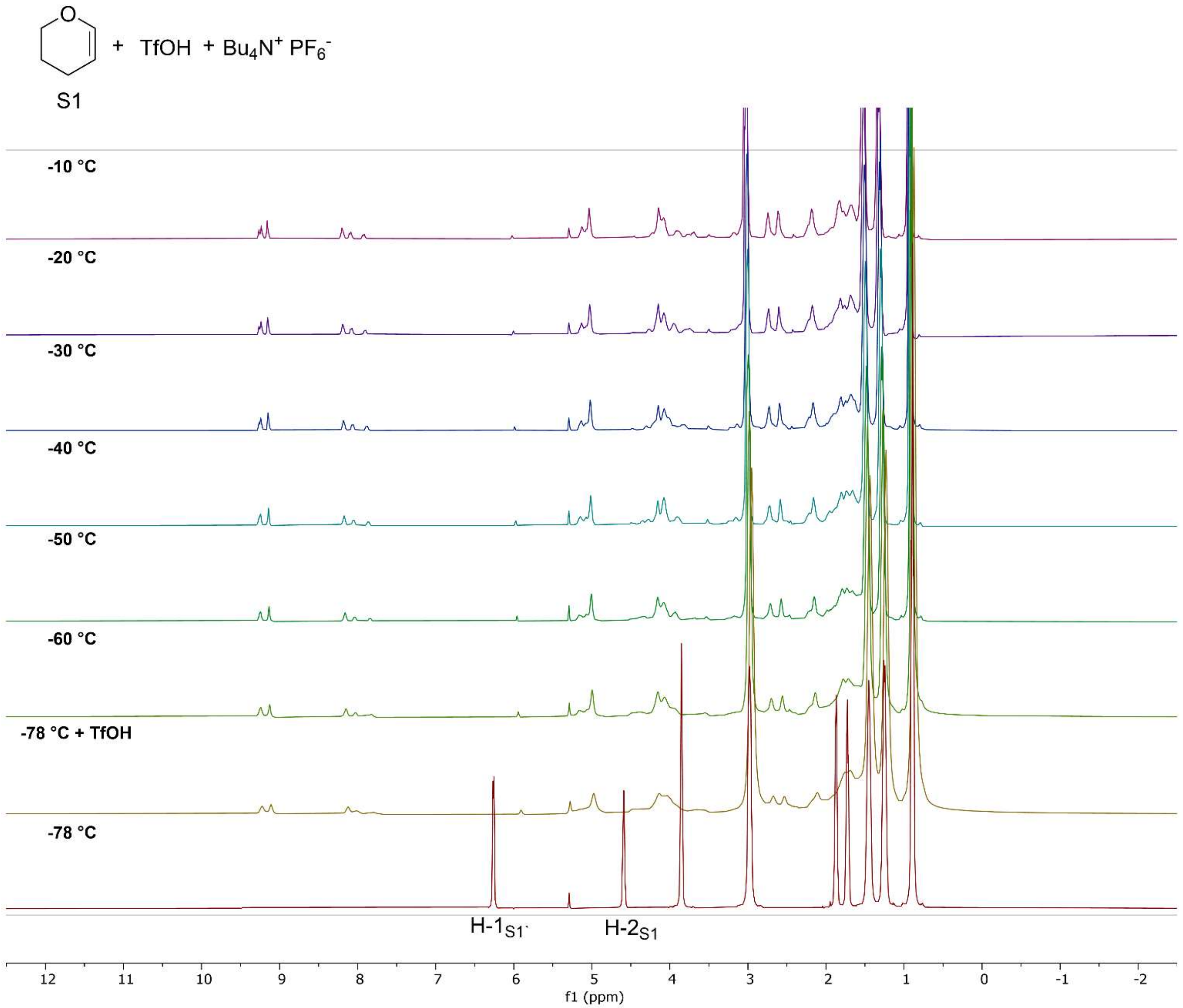

Stacked ^{19}F NMR (470 MHz, CD_2Cl_2) spectra of reaction mixture after protonation of 2,3-dihydro-4*H*-pyran (S1) with 1.1 equiv of TfOH at -78 °C in presence of Bu_4NPF_6

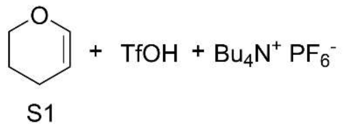

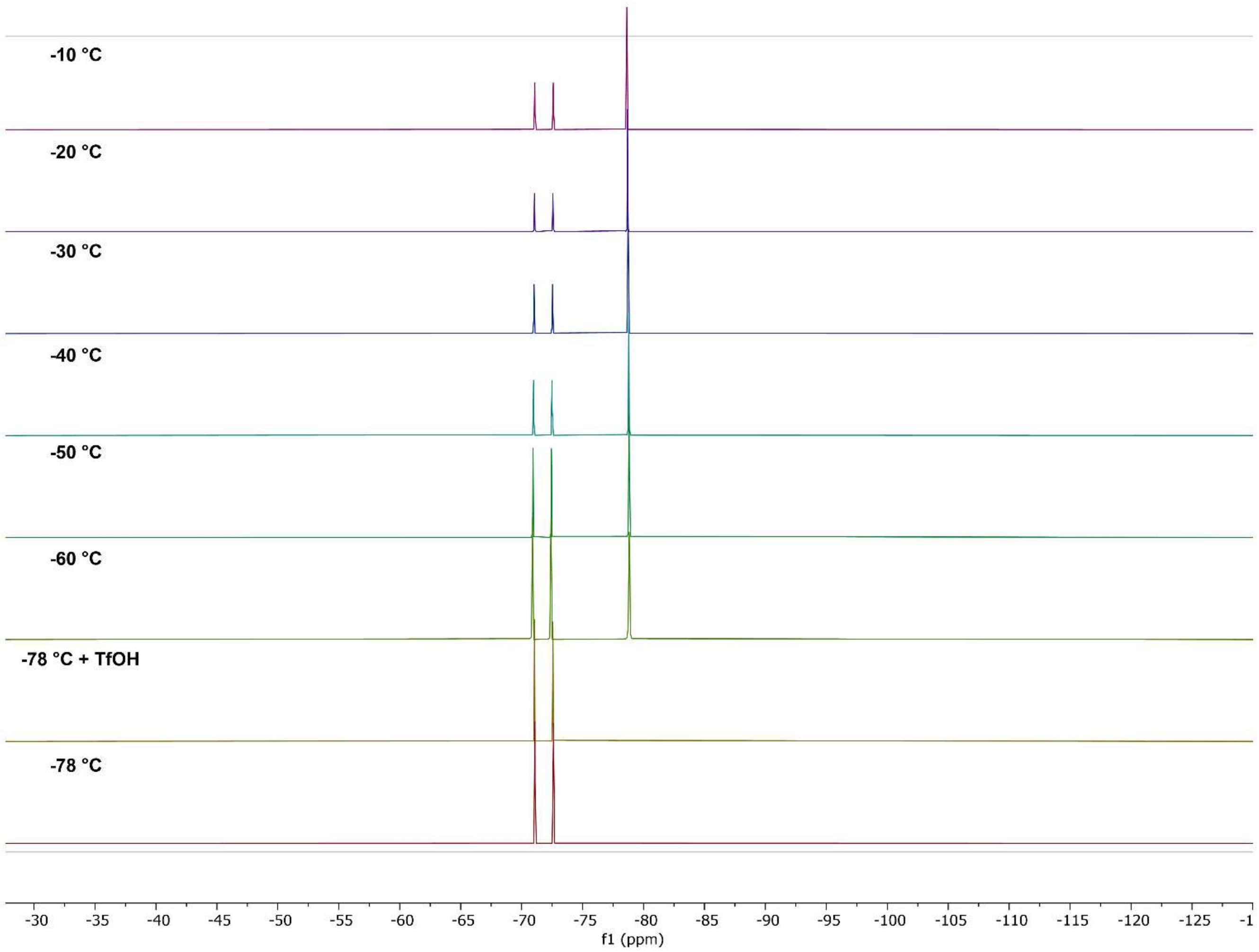

3.6. VT NMR spectra from experiments with phenyl vinyl and ethyl vinyl sulfides

Stacked ^{1}H NMR (500 MHz, CD_2Cl_2) spectra of reaction mixture after protonation of phenyl vinyl sulfide with 2.2 equiv of TfOH at -88 °C

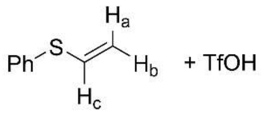

S3

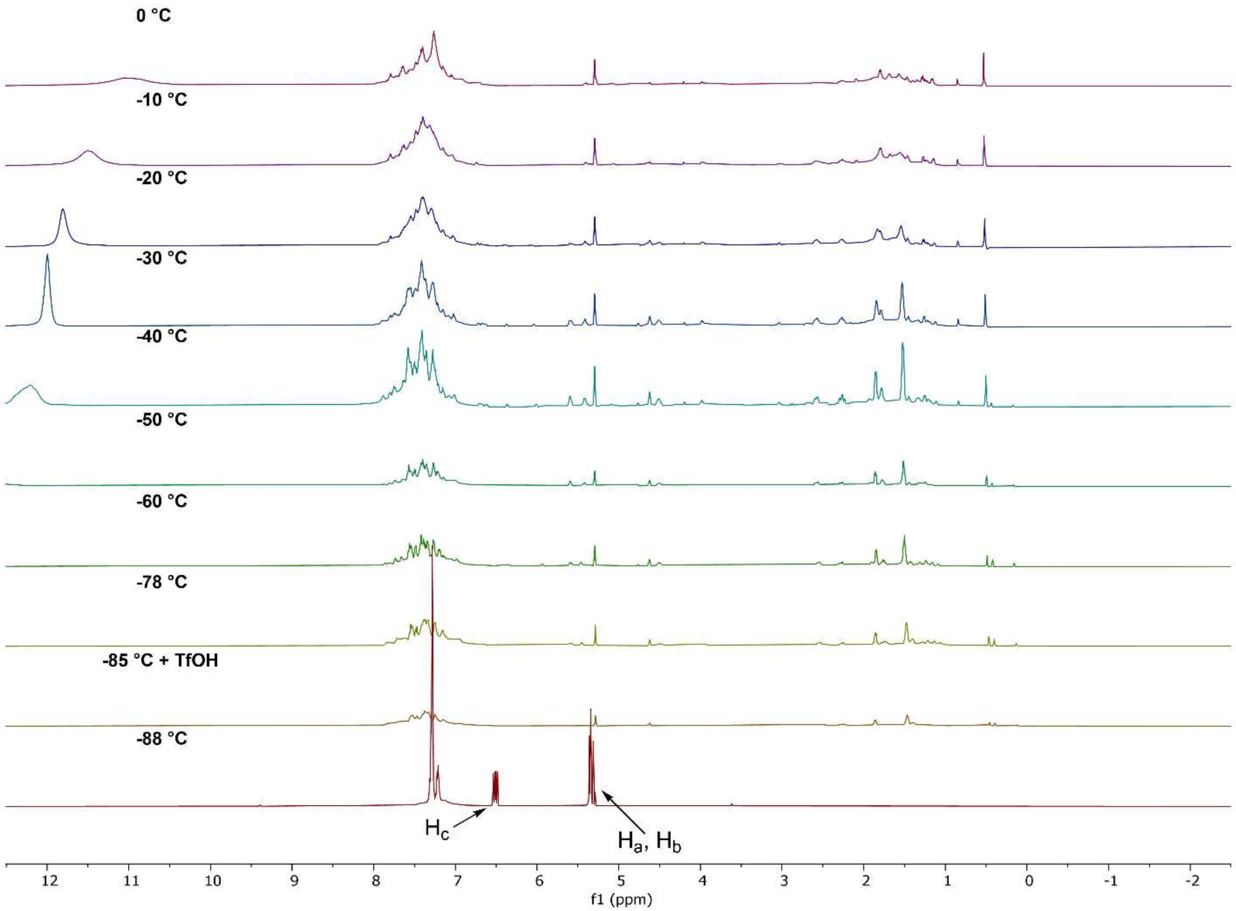

Stacked ^{19}F NMR (470 MHz, CD_2Cl_2) spectra of reaction mixture after protonation of phenyl vinyl sulfide with 2.2 equiv of TfOH at -88 °C

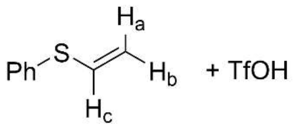

S3

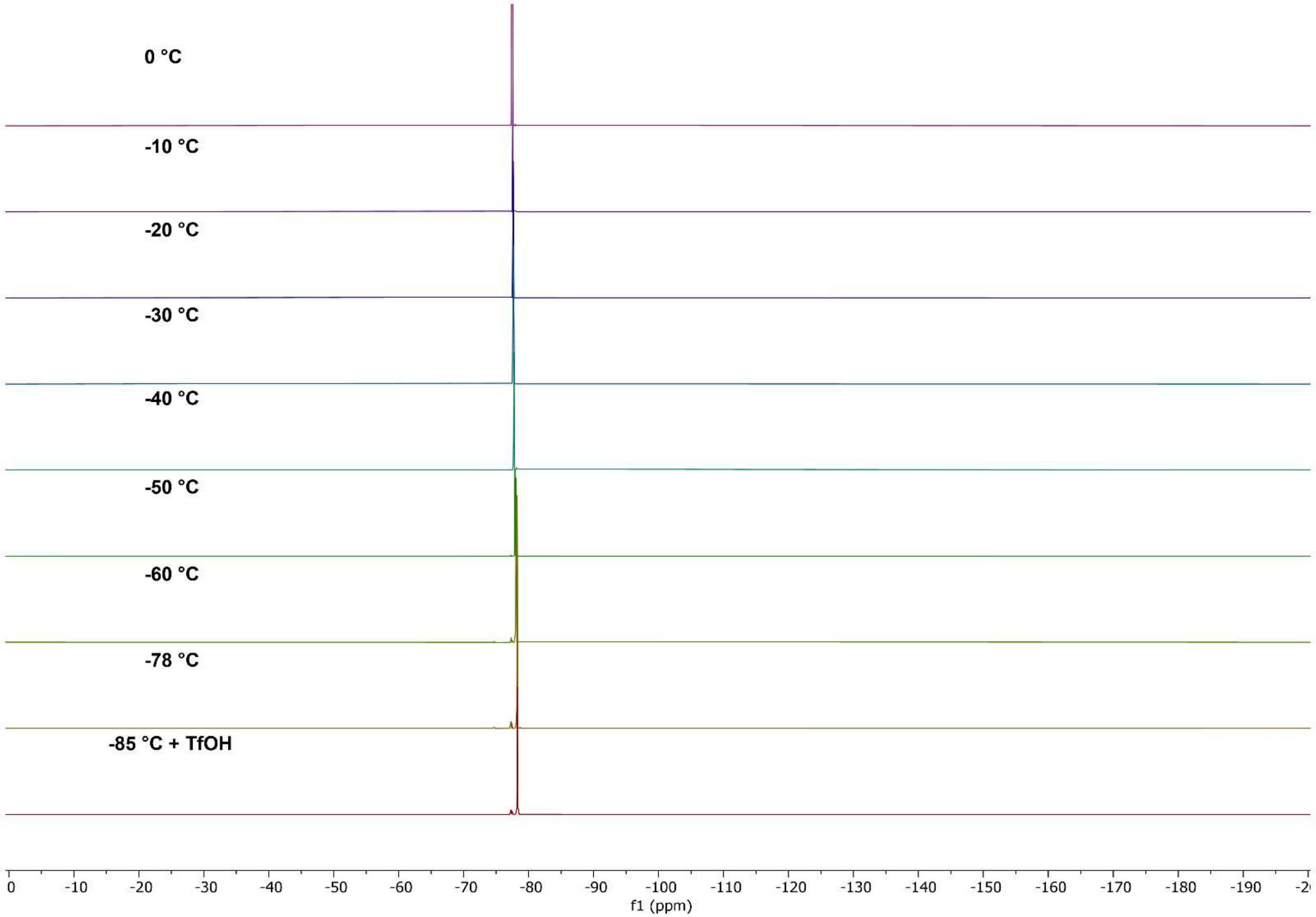

Stacked ^{1}H NMR (500 MHz, CD_2Cl_2) spectra of reaction mixture after protonation of ethyl vinyl sulfide (S7) with 2.2 equiv of TfOH at -88 °C

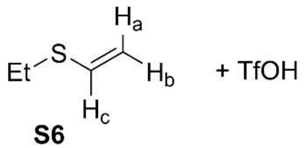

S6

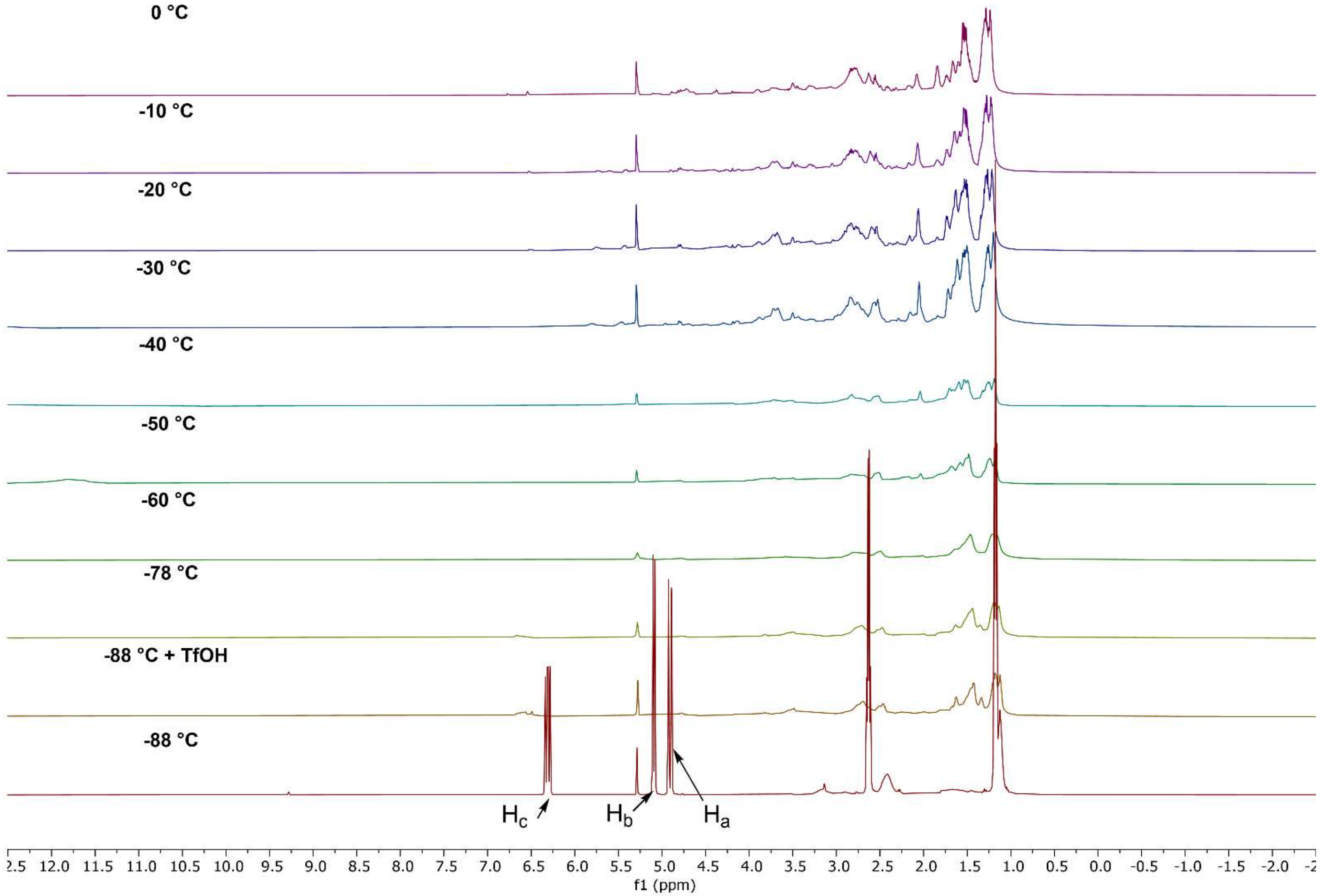

Stacked ^{19}F NMR (470 MHz, CD_2Cl_2) spectra of reaction mixture after protonation of ethyl vinyl sulfide (S7) with 2.2 equiv of TfOH at -88 °C

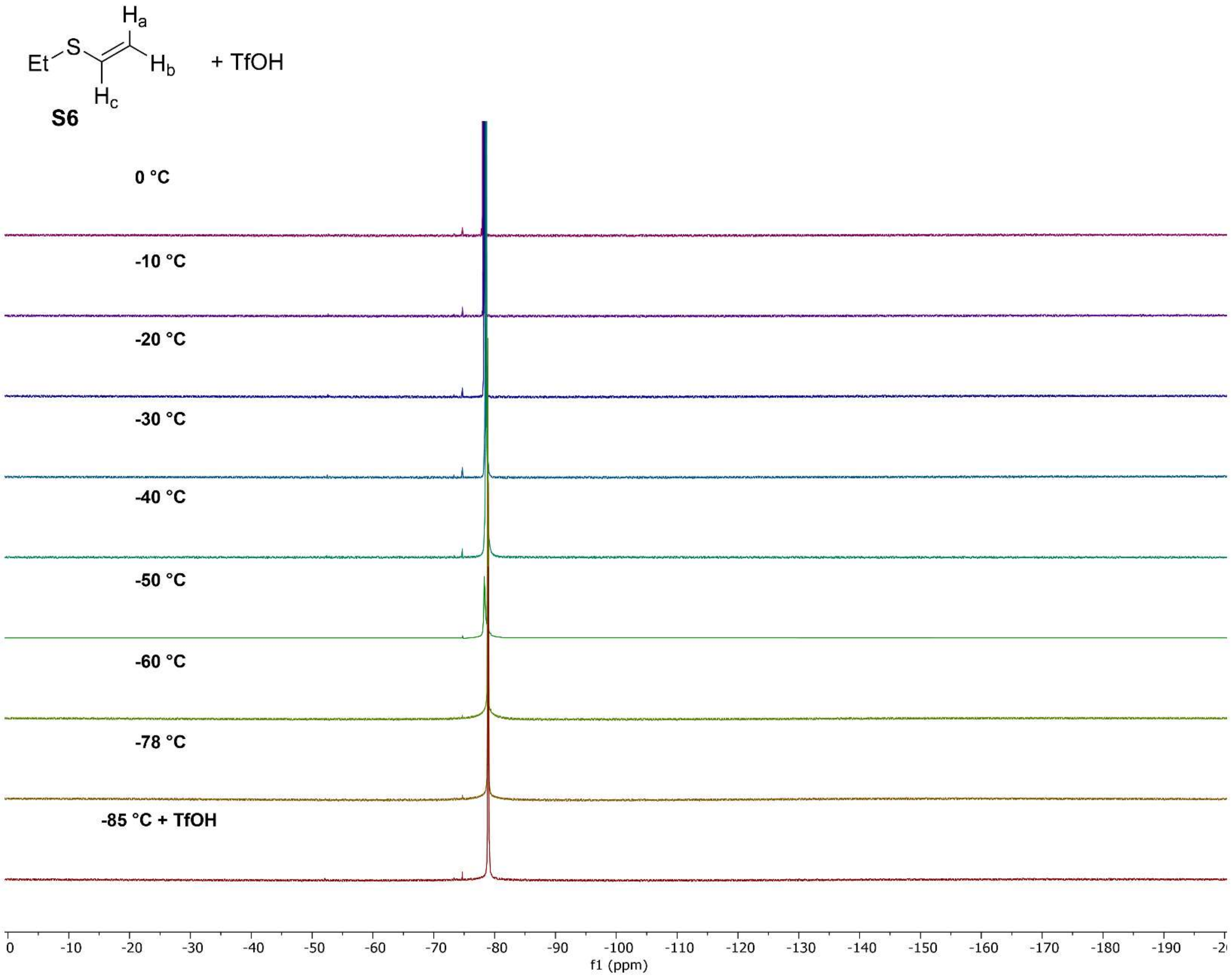

3.7. VT NMR spectra from experiments with peracetylated sulfoxides (50, 51)

Stacked ^{1}H NMR (500 MHz, CD_2Cl_2) spectra from VT NMR experiment with glucosyl sulfoxides 50:

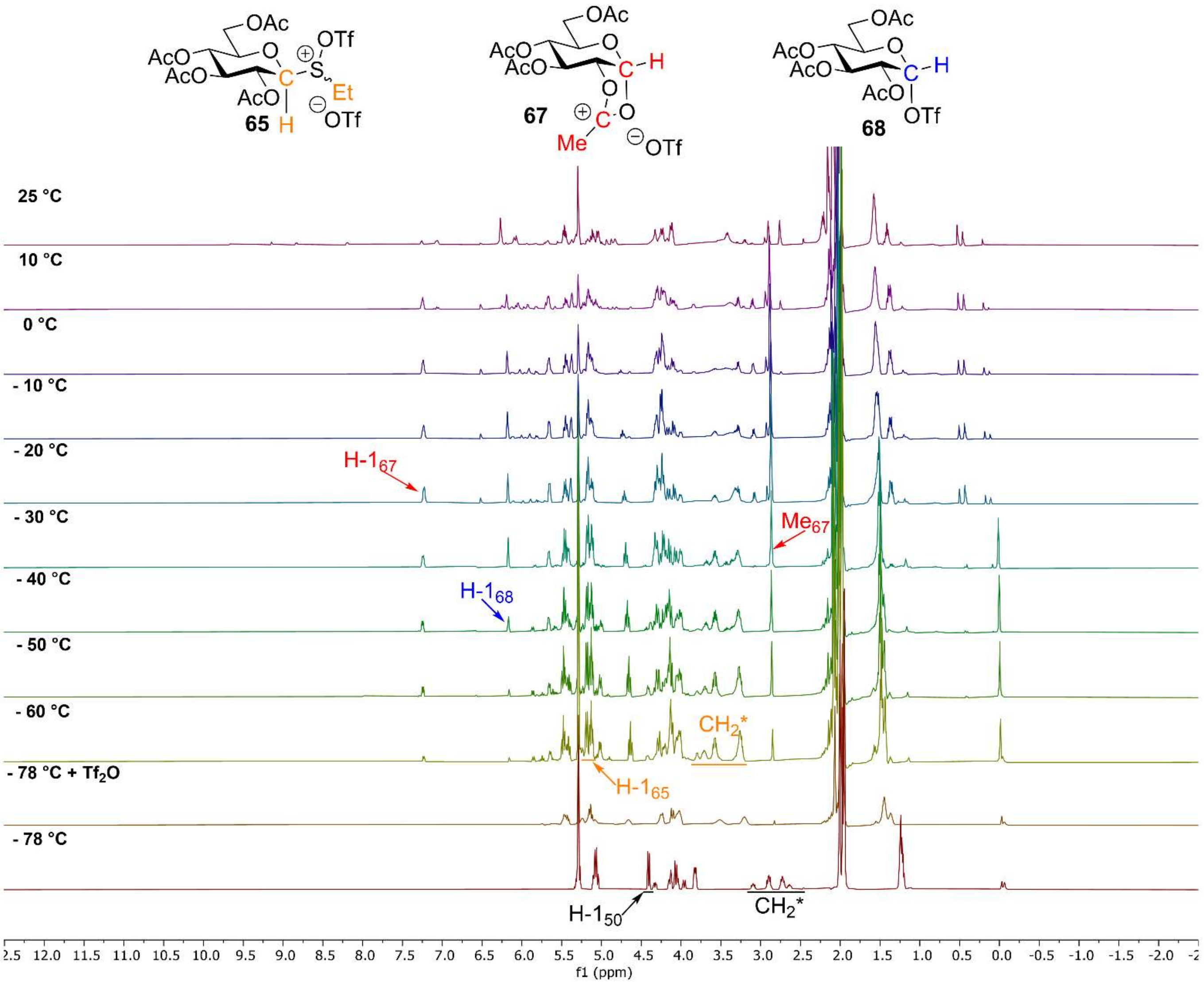

Stacked ^{19}F NMR (470 MHz, CD_2Cl_2) spectra from VT NMR experiment with glucosyl sulfoxides 50:

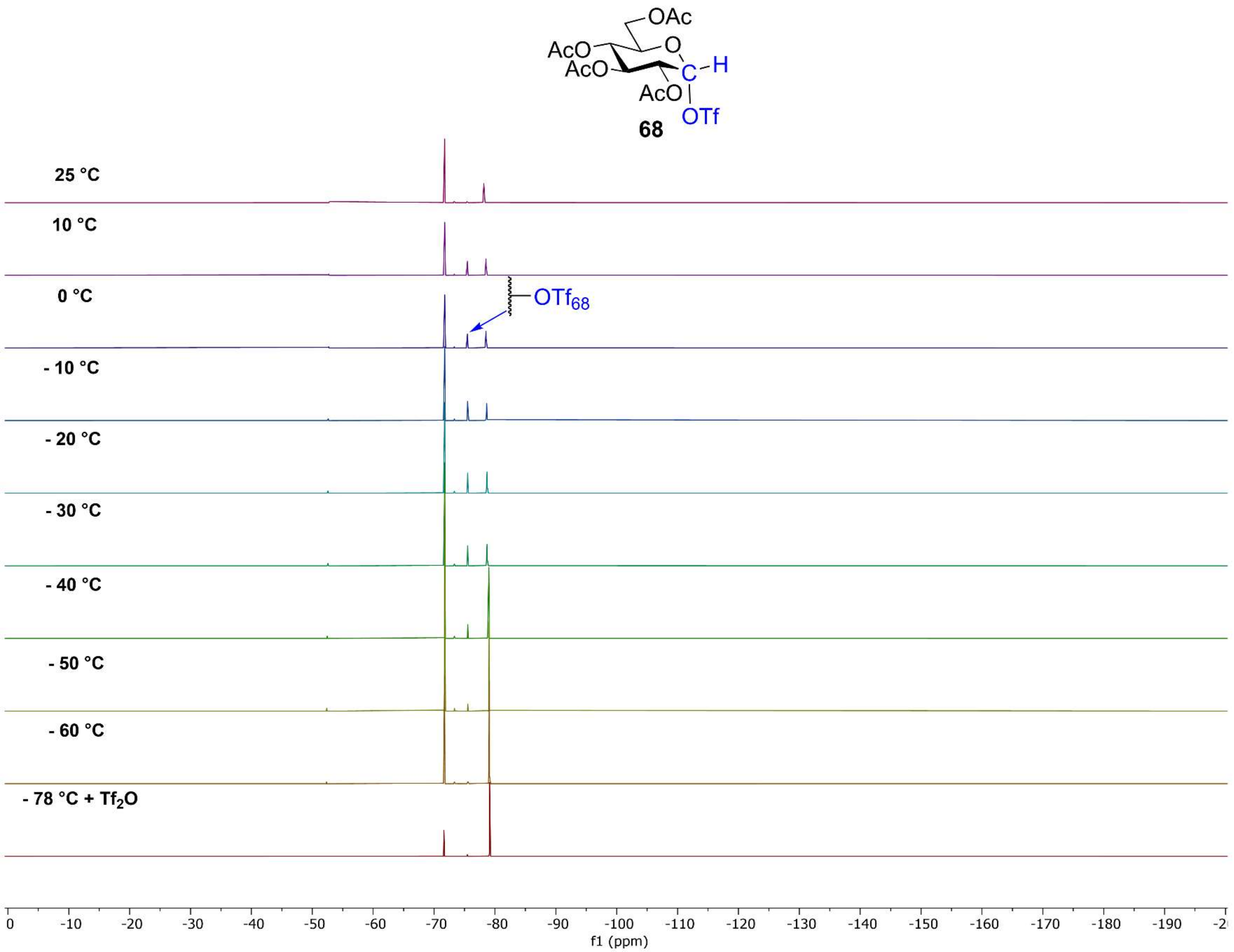

^{1}H NMR (500 MHz, CD_2Cl_2) spectrum of reaction mixture at -30 °C from VT NMR experiment with glucosyl sulfoxides 50:

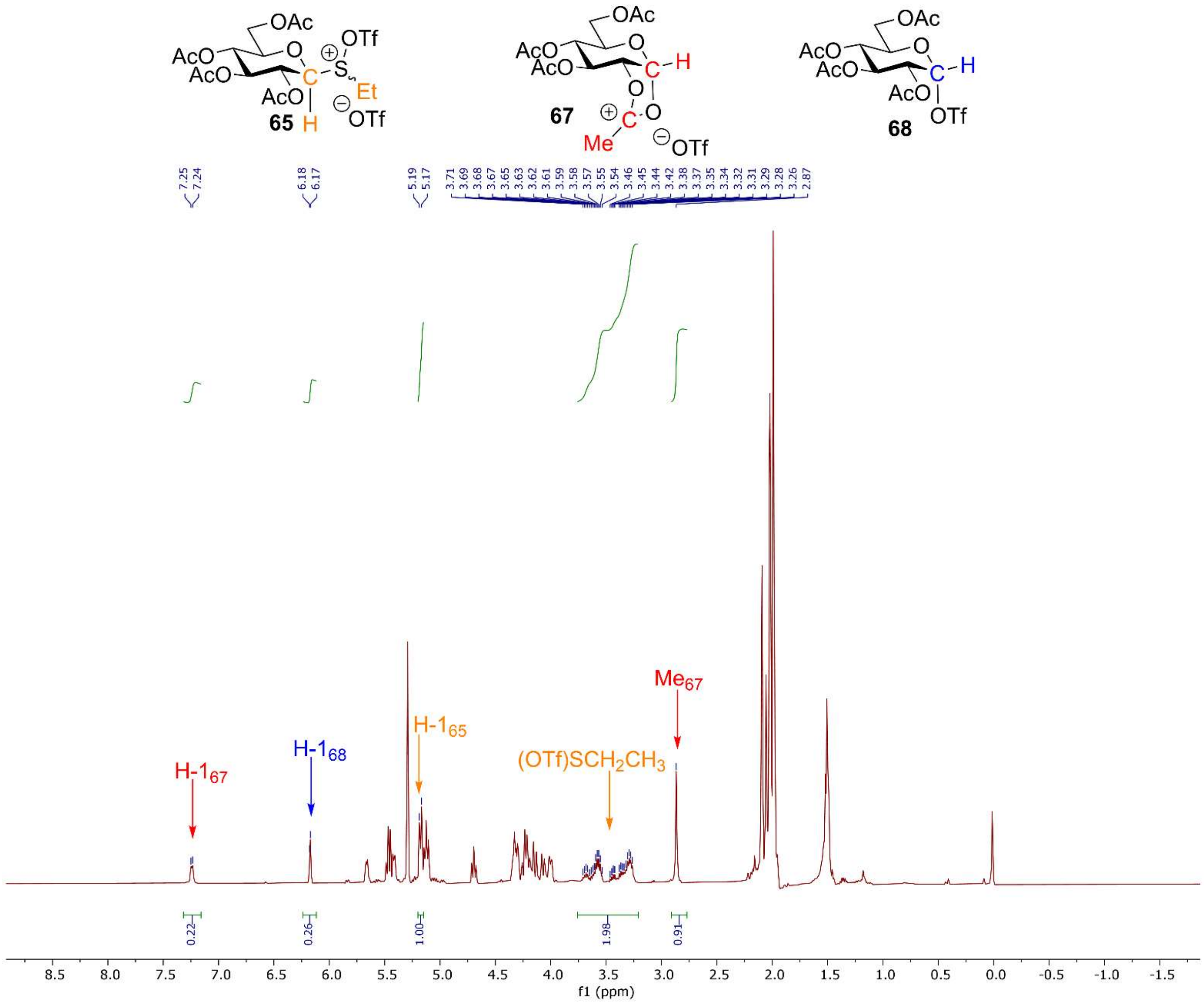

^{13}C NMR (125.67 MHz, CD_2Cl_2) spectrum of reaction mixture at -30 °C from VT NMR experiment with glucosyl sulfoxides 50:

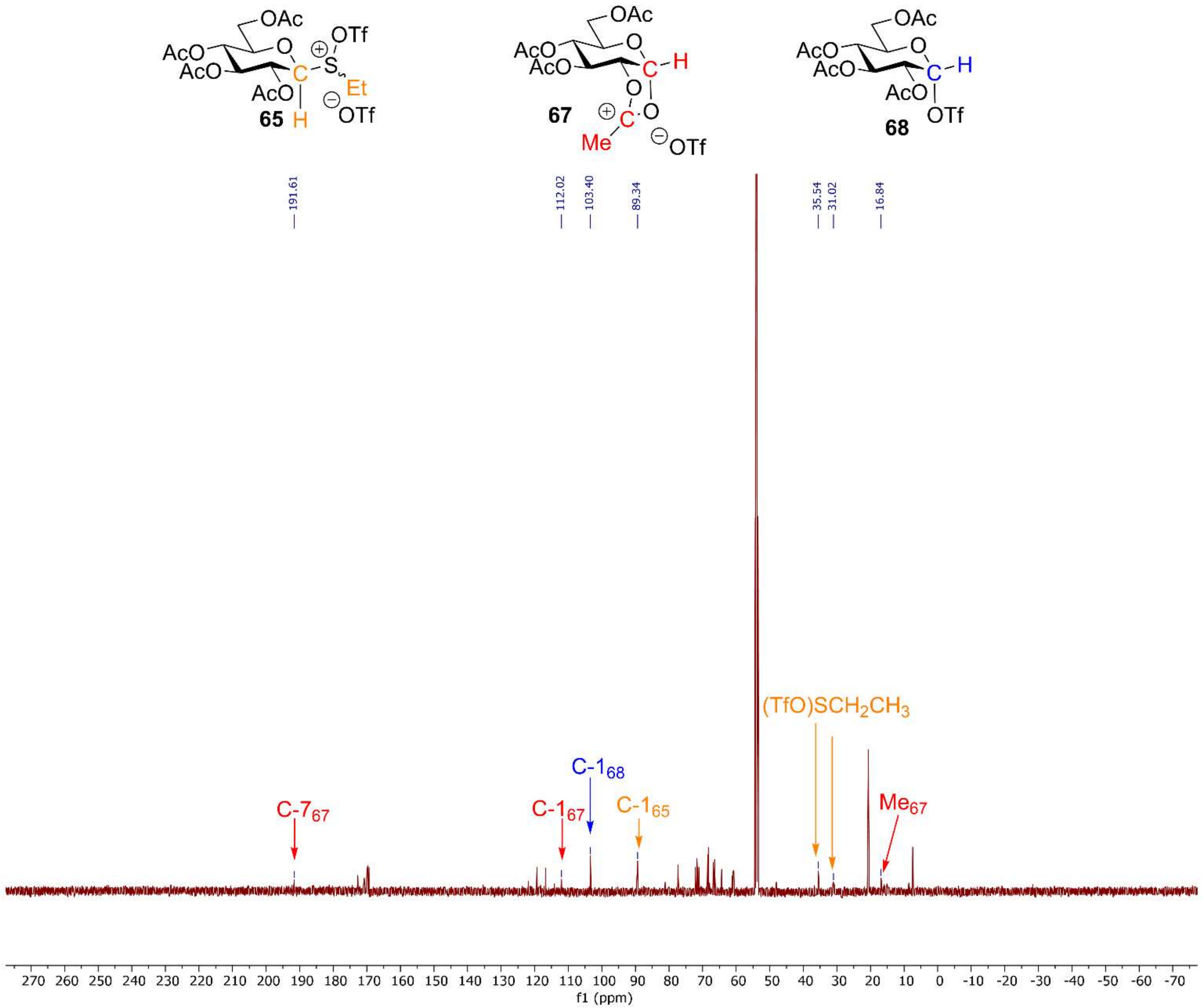

DEPT-135 (CD_2Cl_2) spectrum of reaction mixture at -30 °C from VT NMR experiment with glucosyl sulfoxides 50:

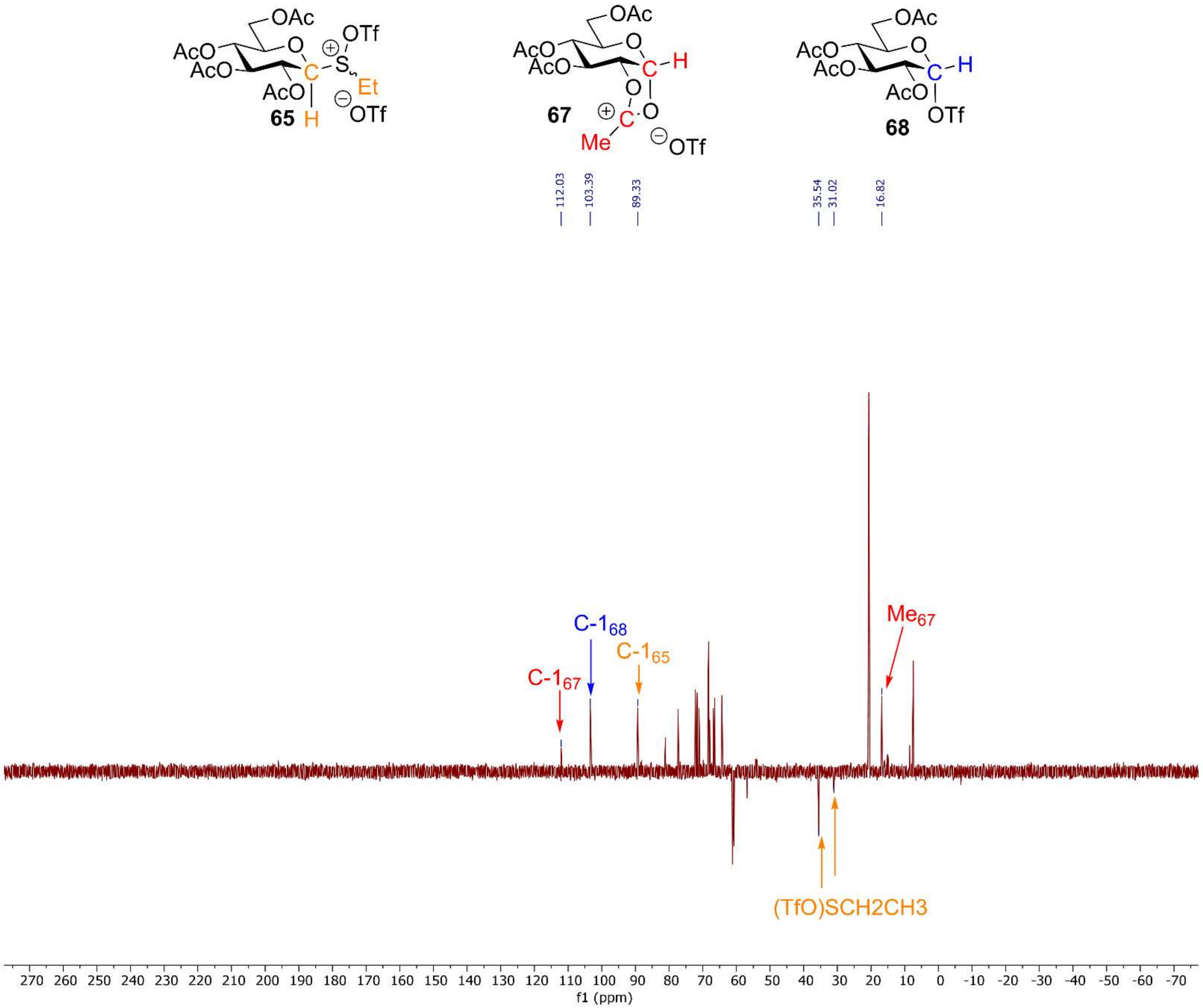

COSY (CD_2Cl_2) spectrum of reaction mixture at -30 °C from VT NMR experiment with glucosyl sulfoxides 50:

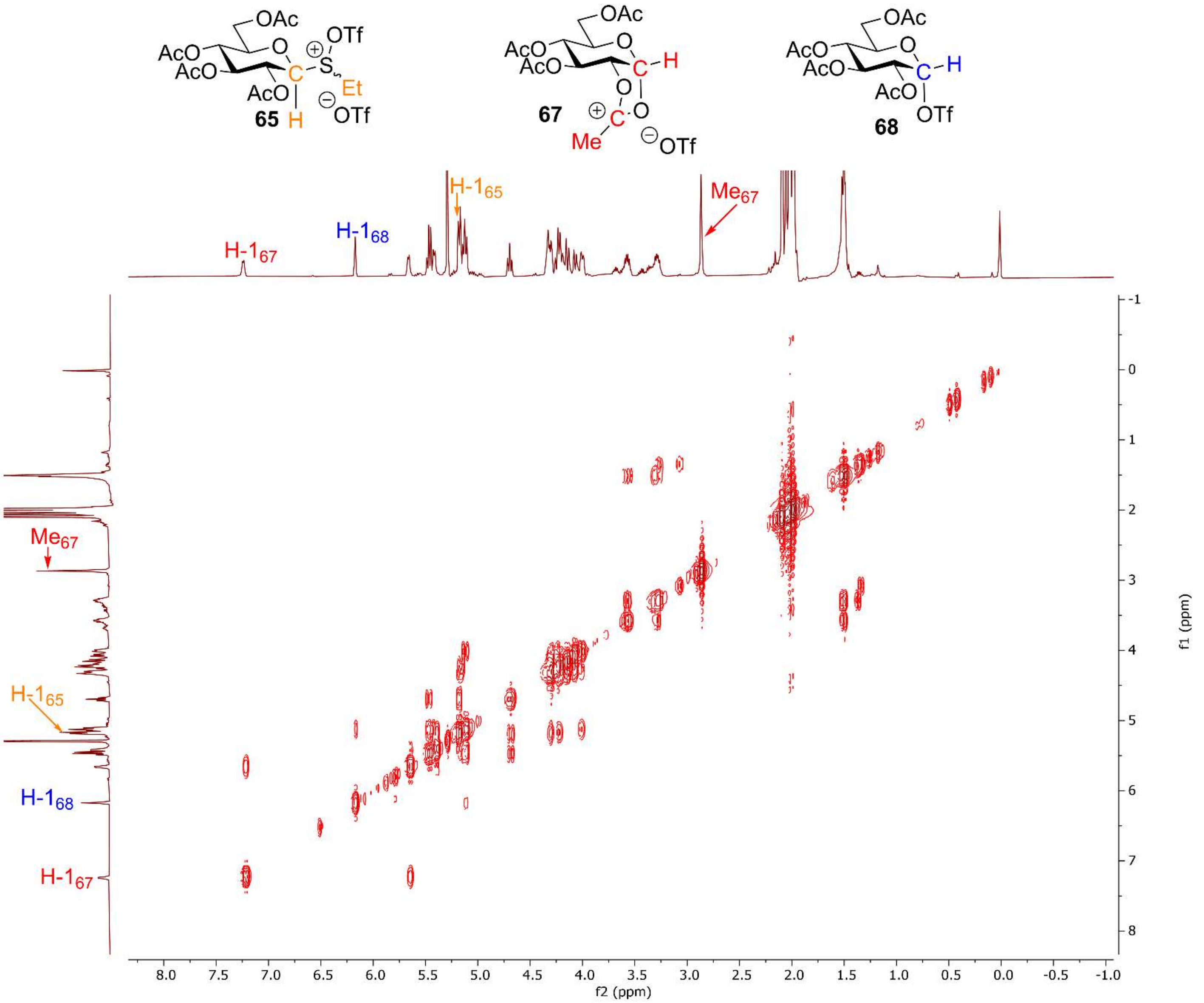

HMQC (CD_2Cl_2) spectrum of reaction mixture at -30 °C from VT NMR experiment with glucosyl sulfoxides 50:

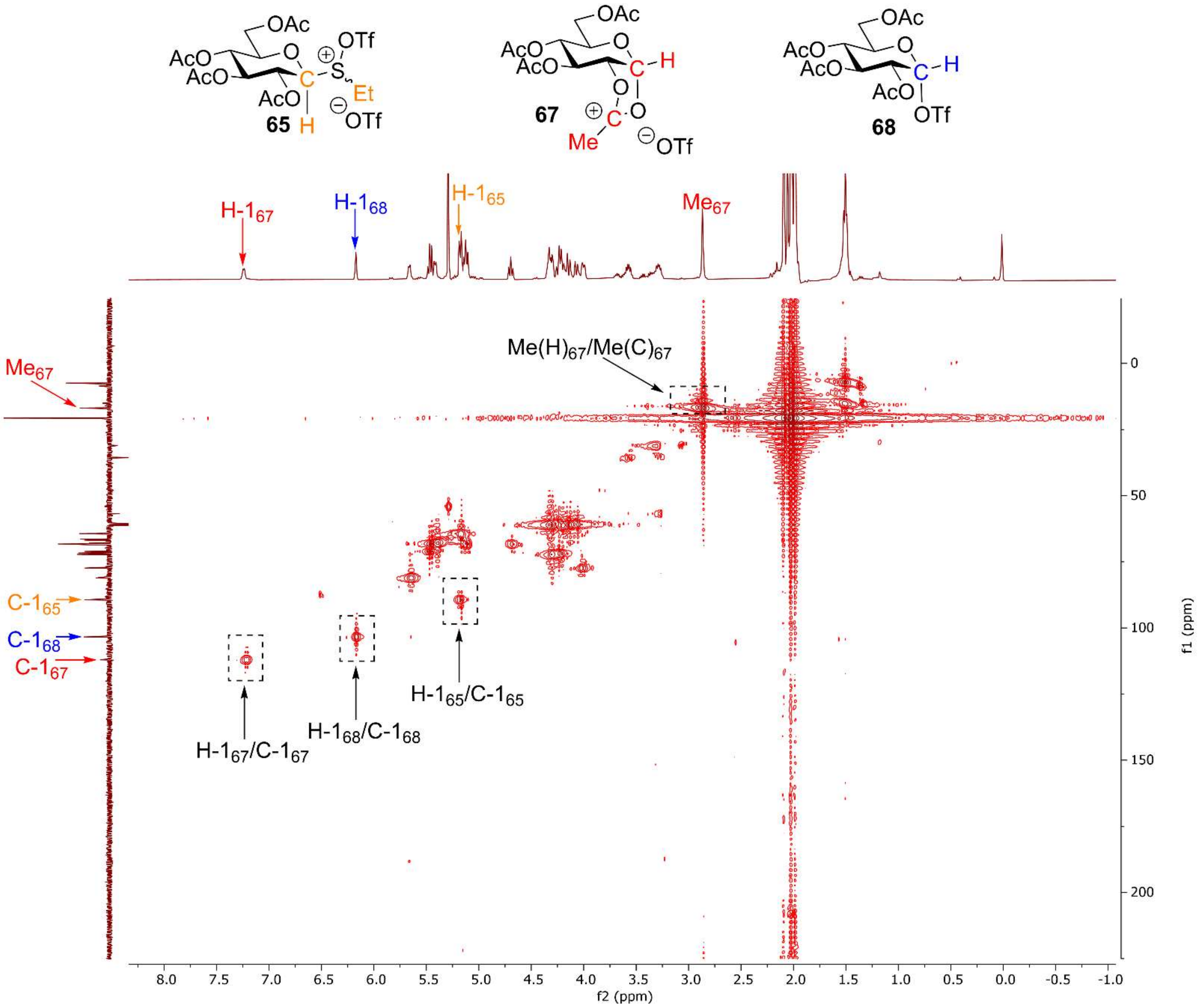

HMBC (CD_2Cl_2) spectrum of reaction mixture at -30 °C from VT NMR experiment with glucosyl sulfoxides 50:

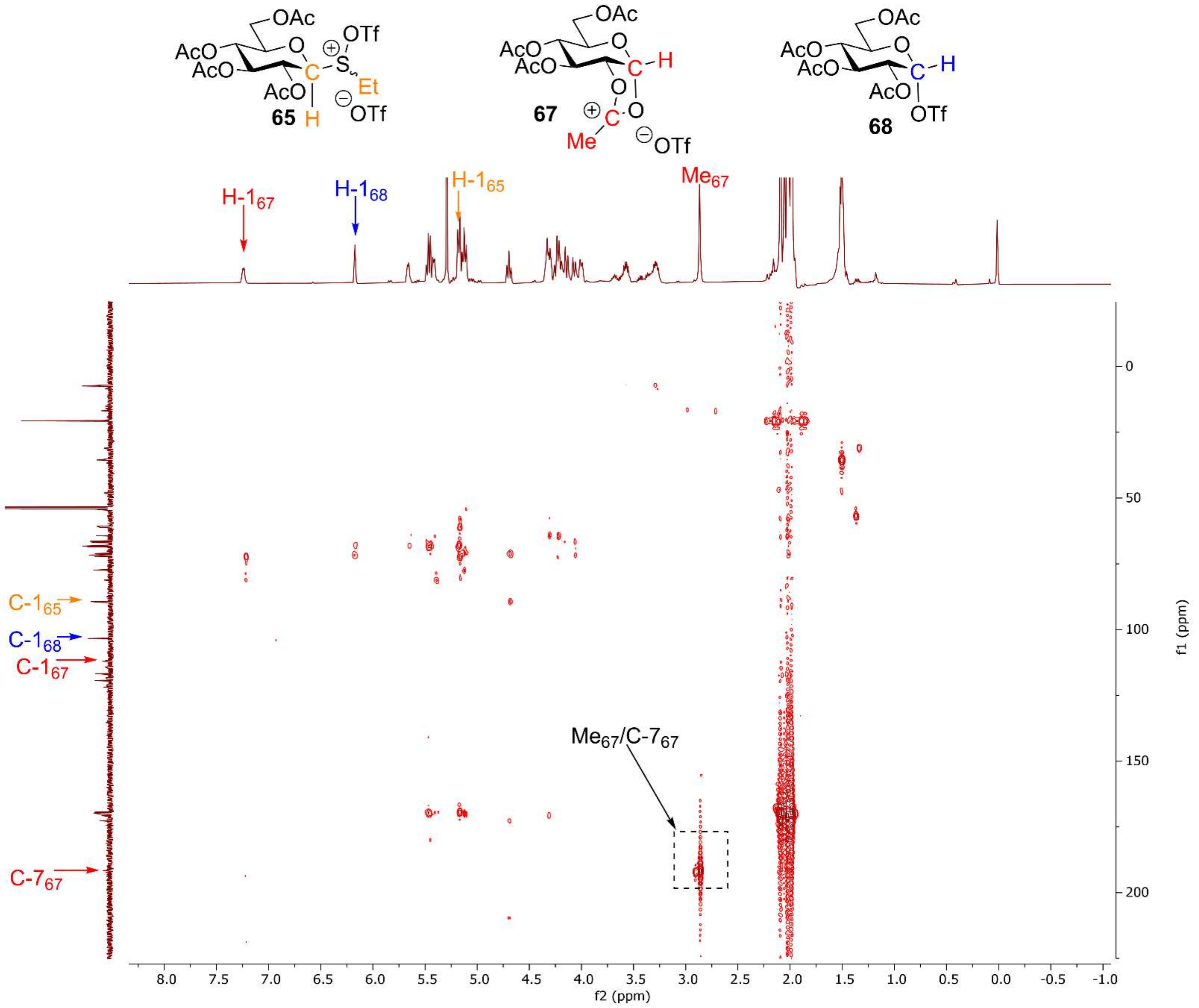

^{19}F NMR (470 MHz, CD_2Cl_2) spectrum of reaction mixture at -30 °C from VT NMR experiment with glucosyl sulfoxides 50:

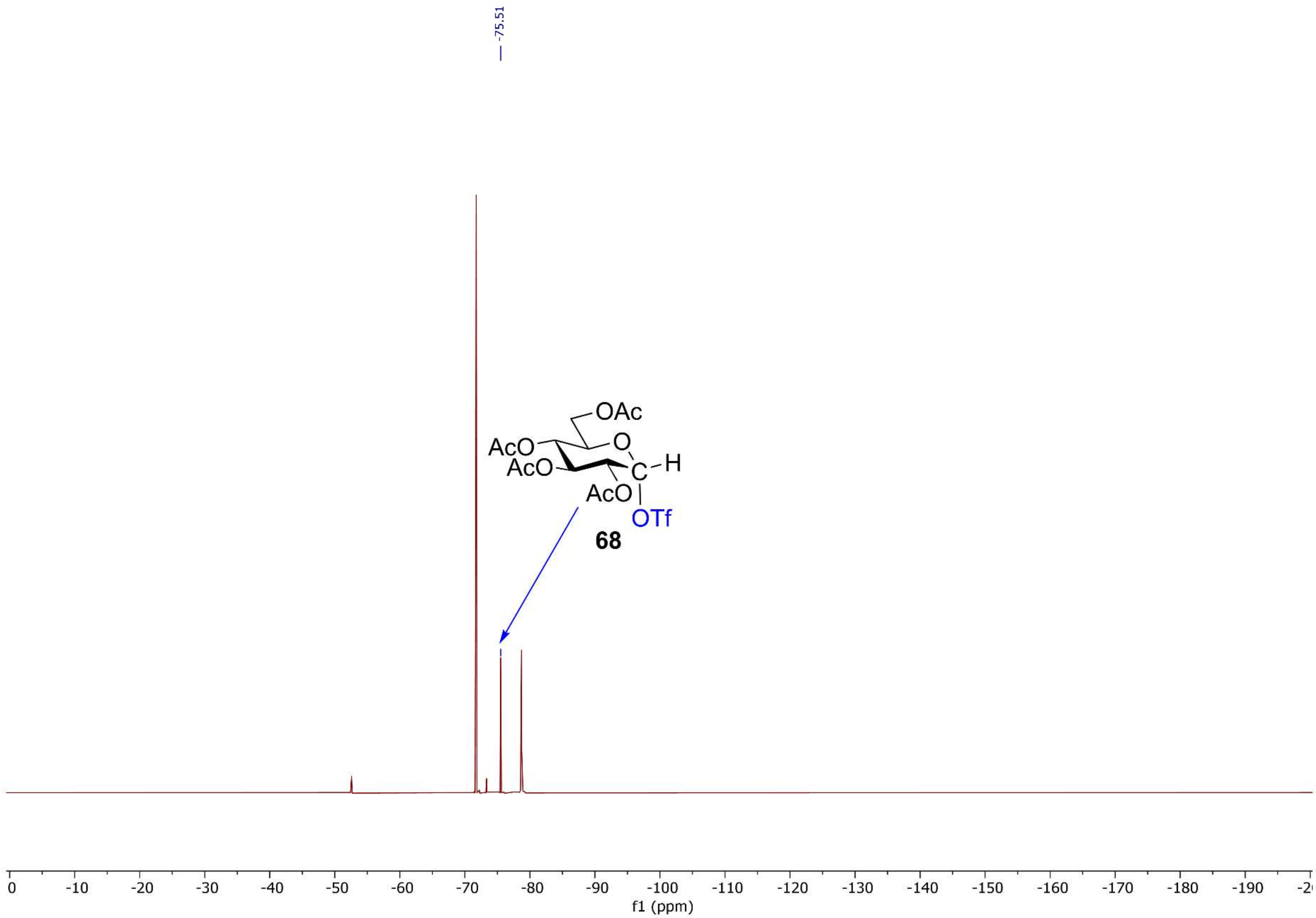

Stacked ^{1}H NMR (500 MHz, CD_2Cl_2) spectra from VT NMR experiment with glucosyl sulfoxides 51:

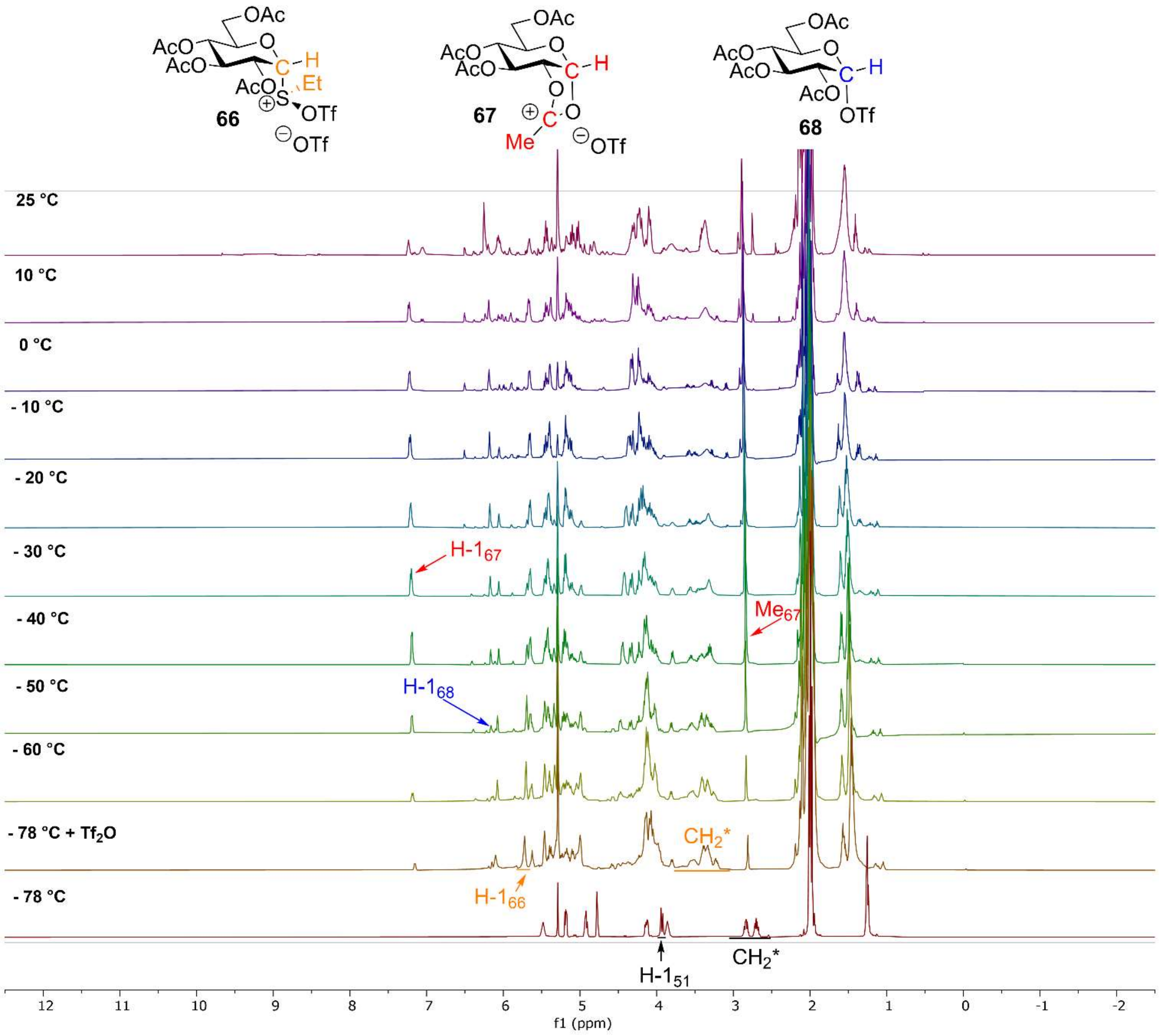

Stacked ^{19}F NMR (470 MHz, CD_2Cl_2) spectra from VT NMR experiment with glucosyl sulfoxides 51:

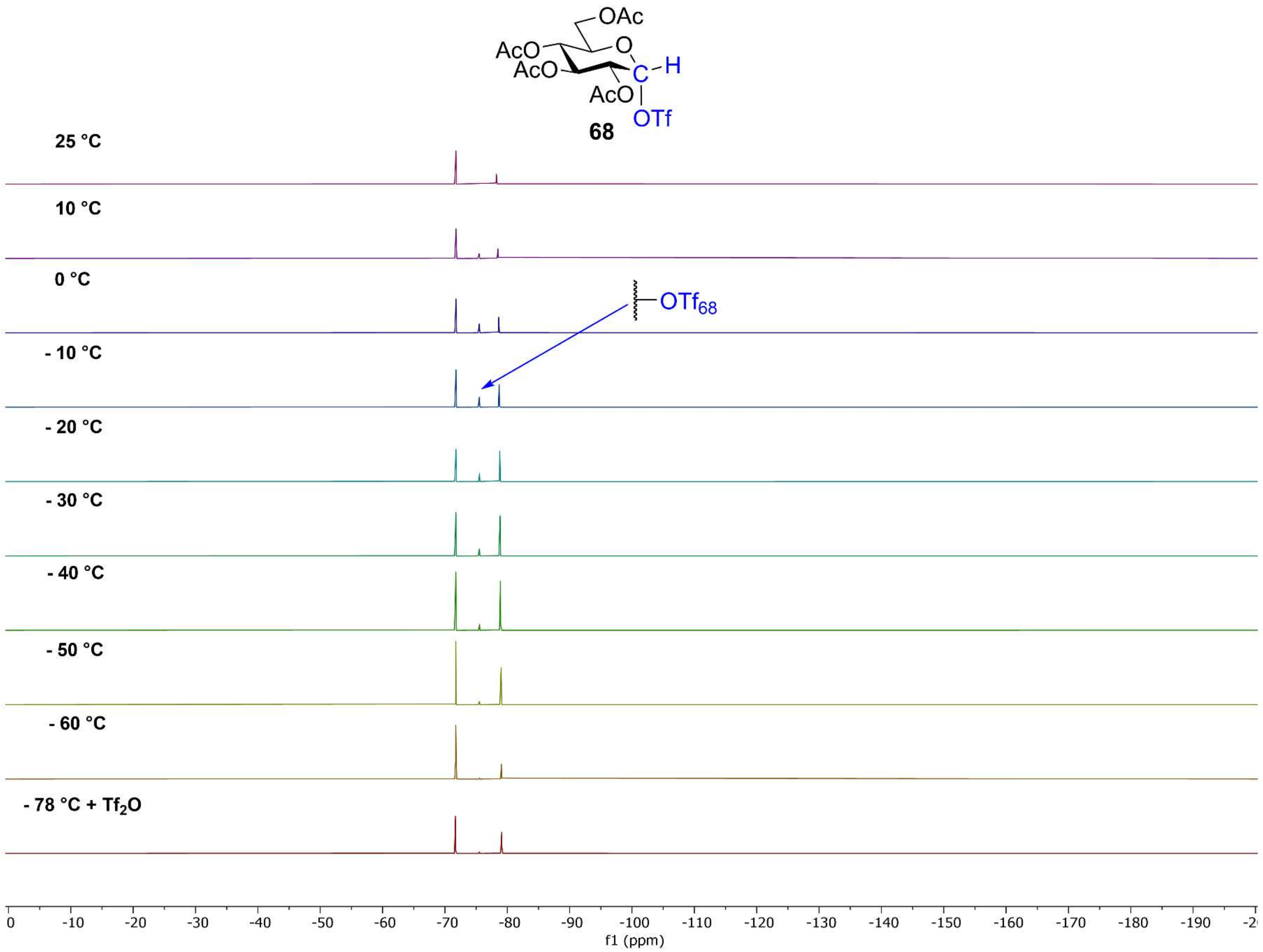

^{1}H NMR (500 MHz, CD_2Cl_2) spectrum of reaction mixture at -50 °C from VT NMR experiment with glucosyl sulfoxide 51:

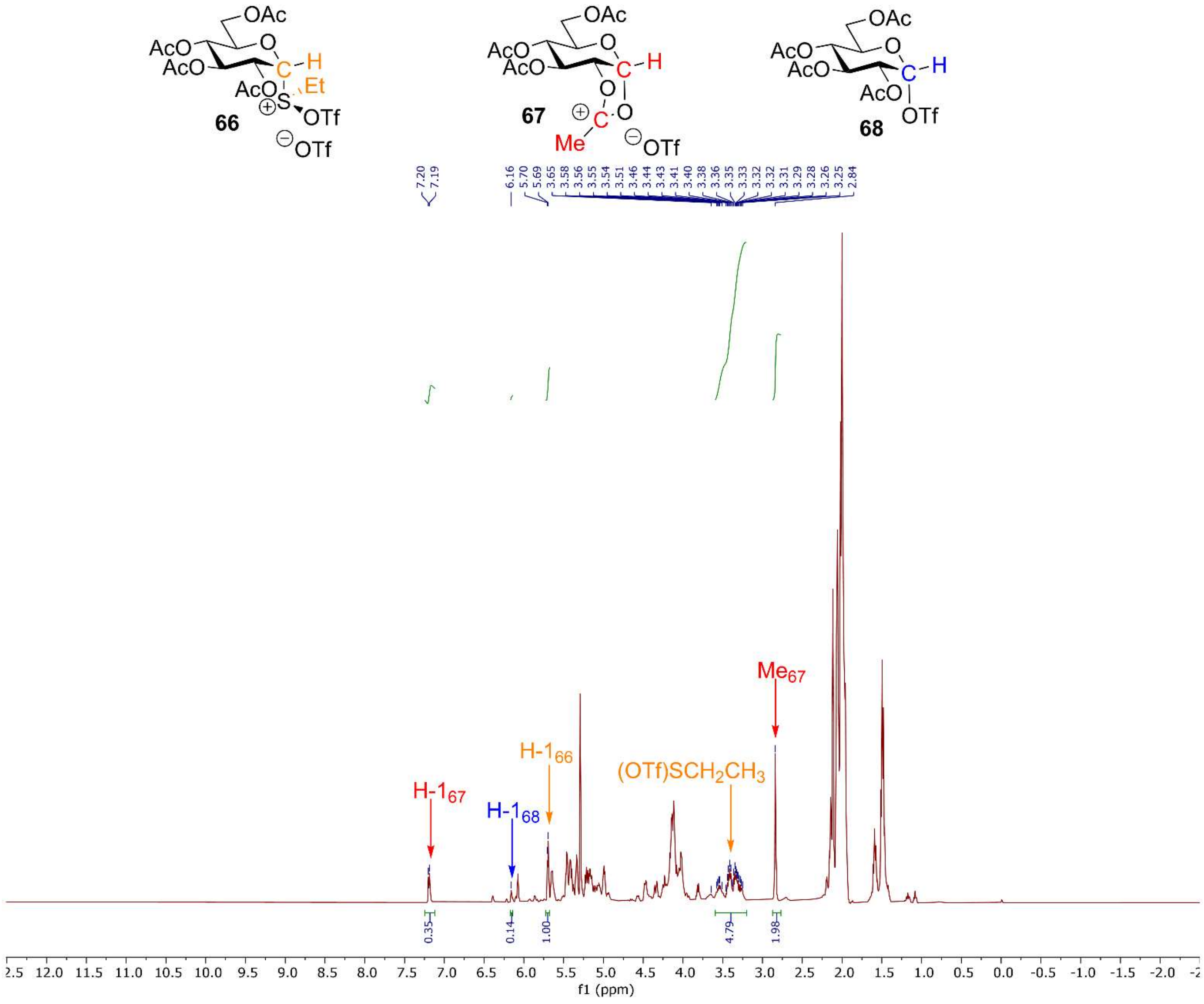

^{13}C NMR (125.67 MHz, CD_2Cl_2) spectrum of reaction mixture at -50 °C from VT NMR experiment with glucosyl sulfoxide 51:

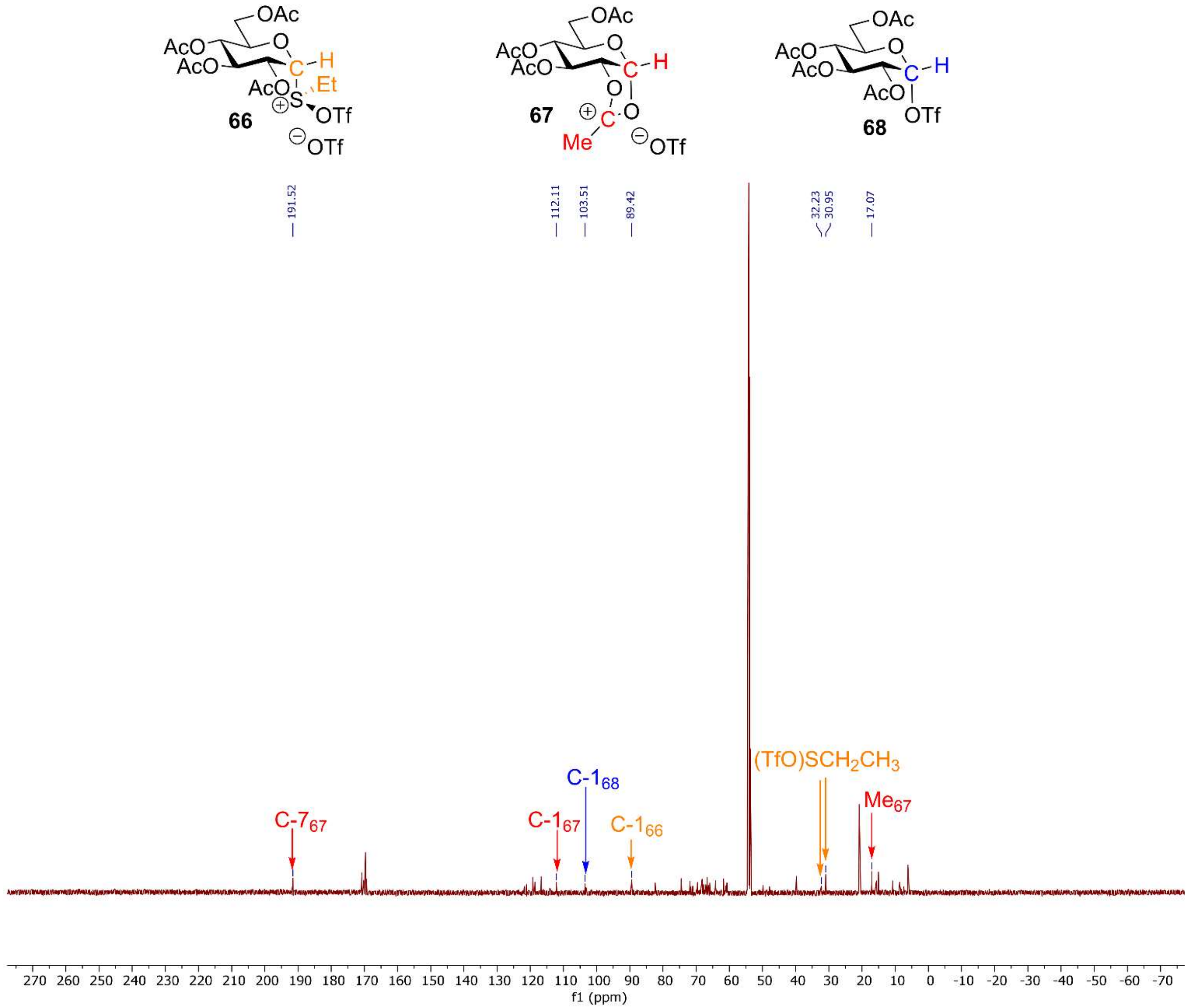

DEPT-135 (CD_2Cl_2) spectrum of reaction mixture at -50 °C from VT NMR experiment with glucosyl sulfoxide 51:

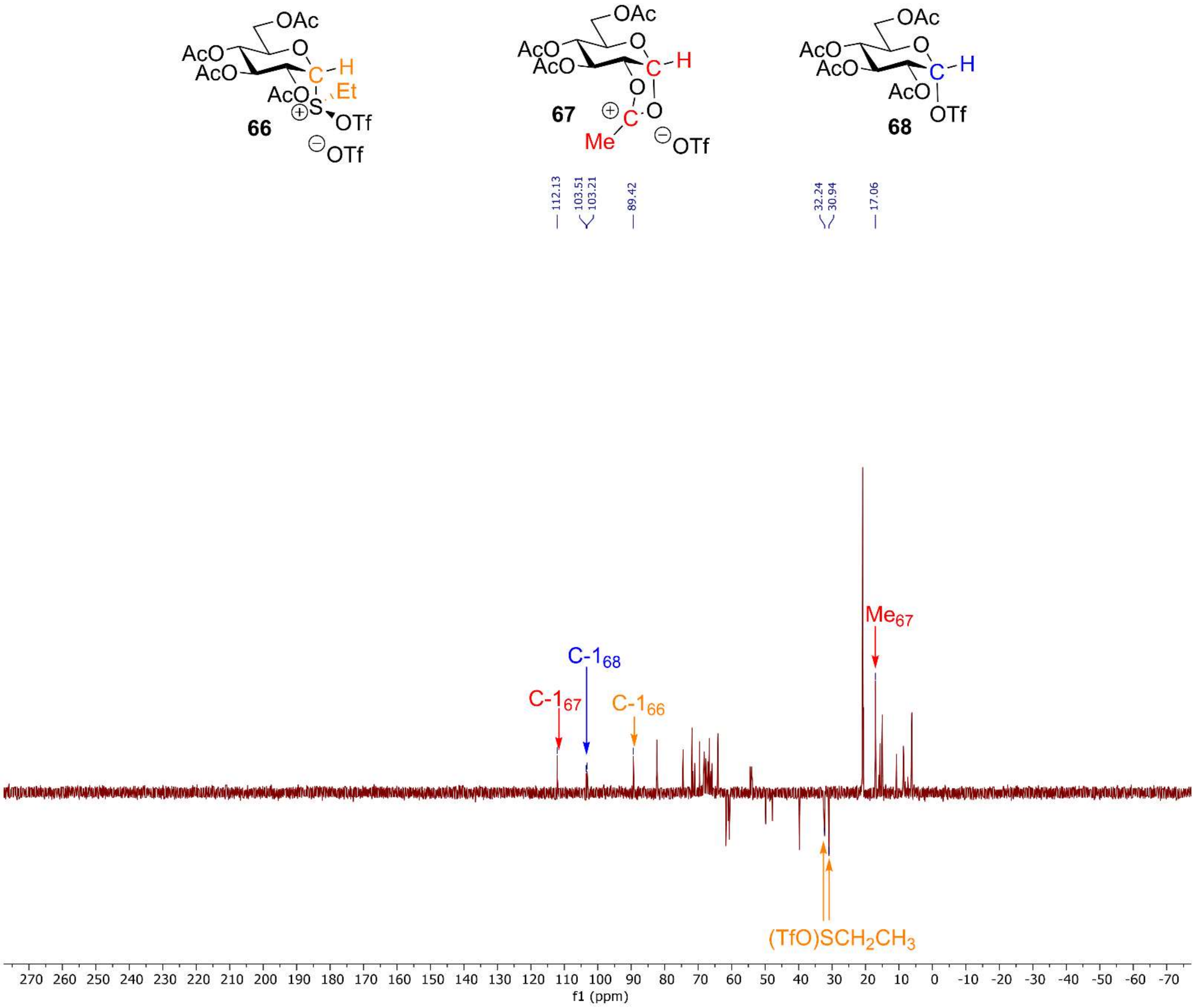

COSY (CD_2Cl_2) spectrum of reaction mixture at -50 °C from VT NMR experiment with glucosyl sulfoxide 51:

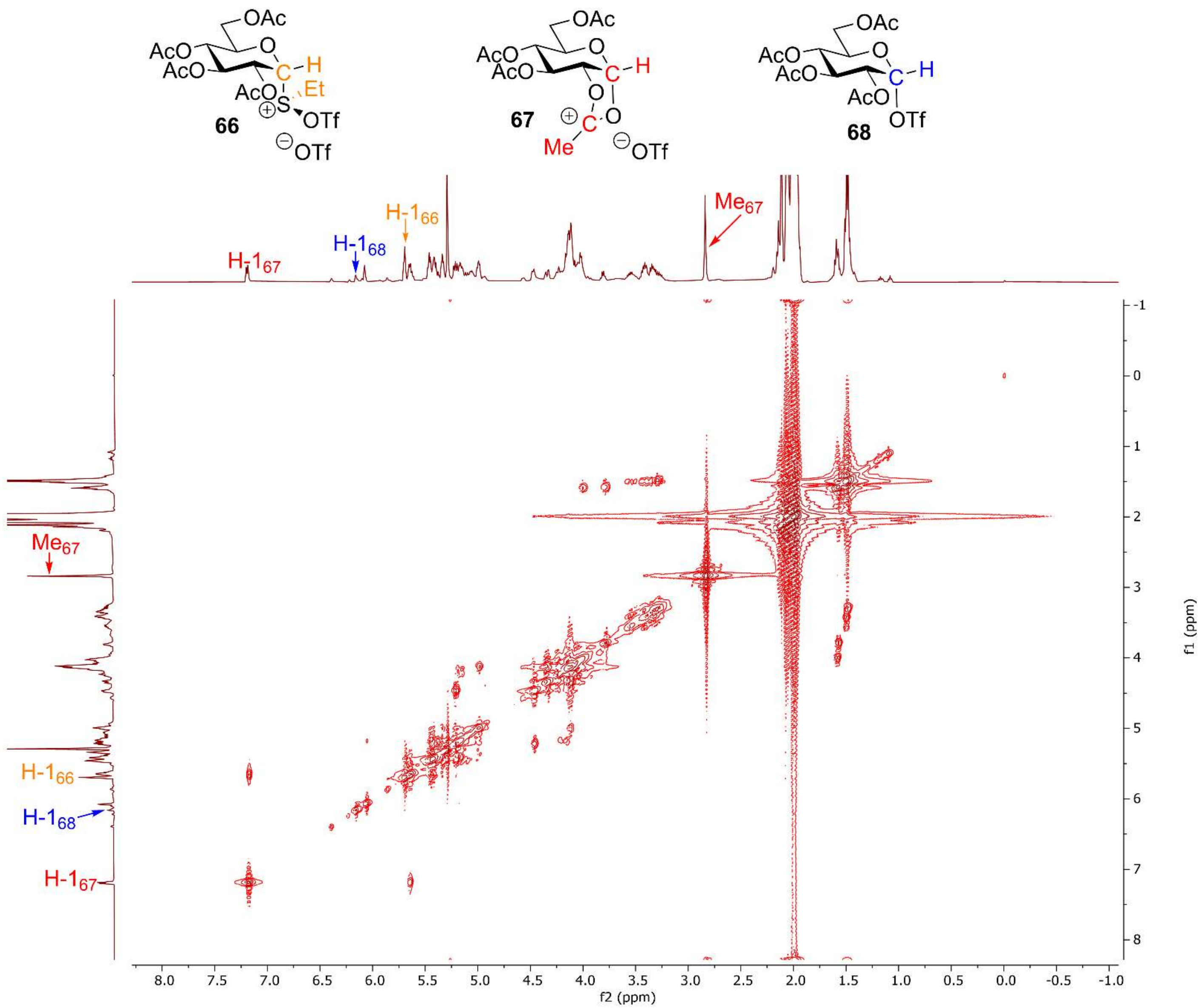

HMQC (CD_2Cl_2) spectrum of reaction mixture at -50 °C from VT NMR experiment with glucosyl sulfoxide 51:

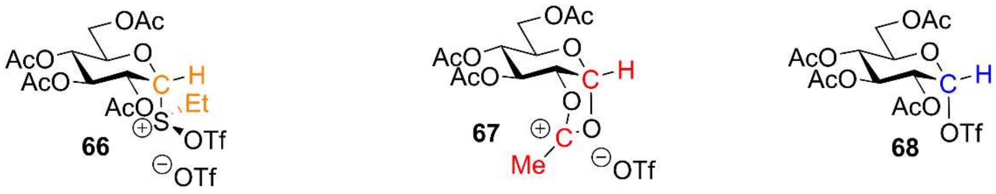

66

67

68

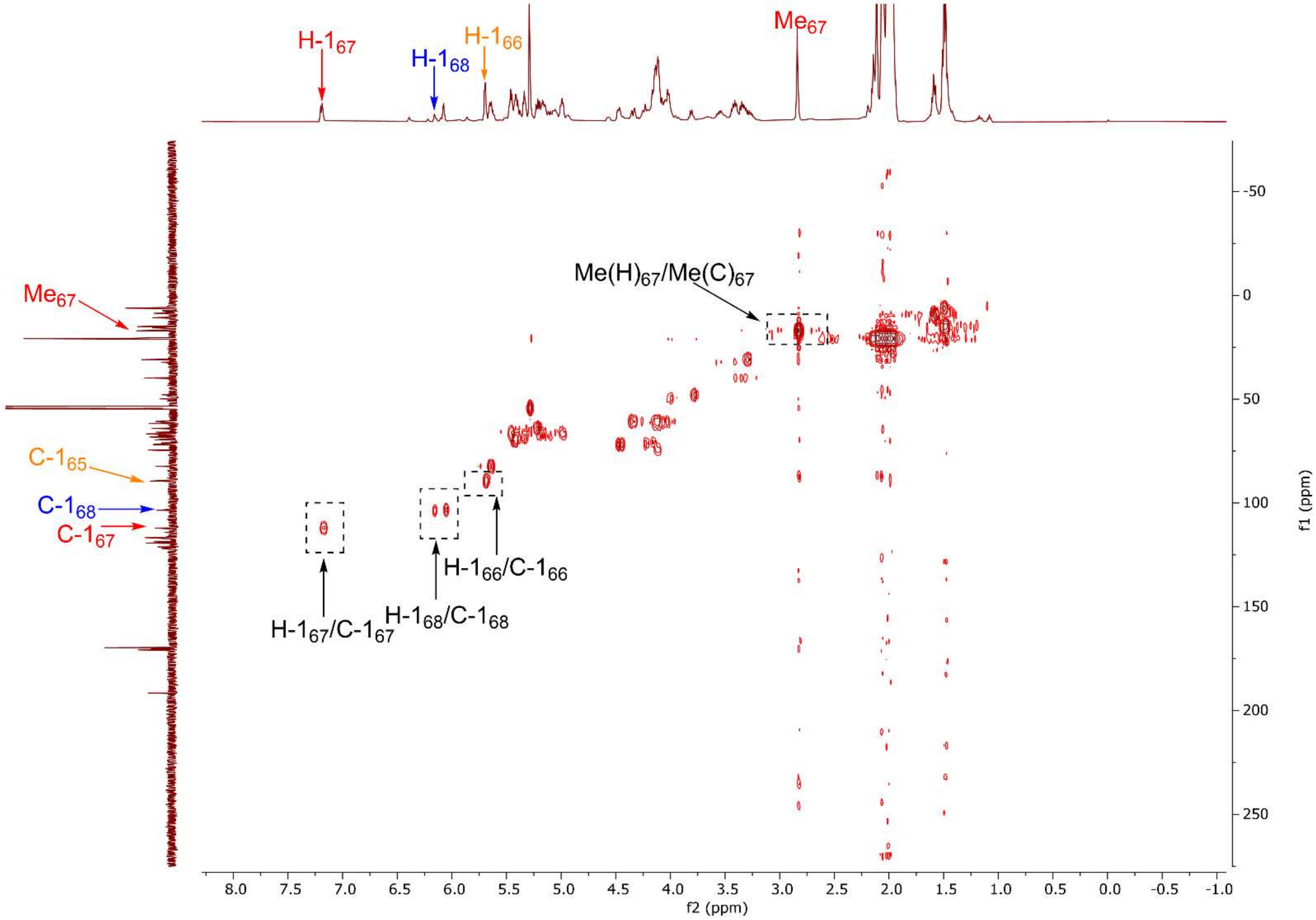

HMBC (CD_2Cl_2) spectrum of reaction mixture at -50 °C from VT NMR experiment with glucosyl sulfoxide 51:

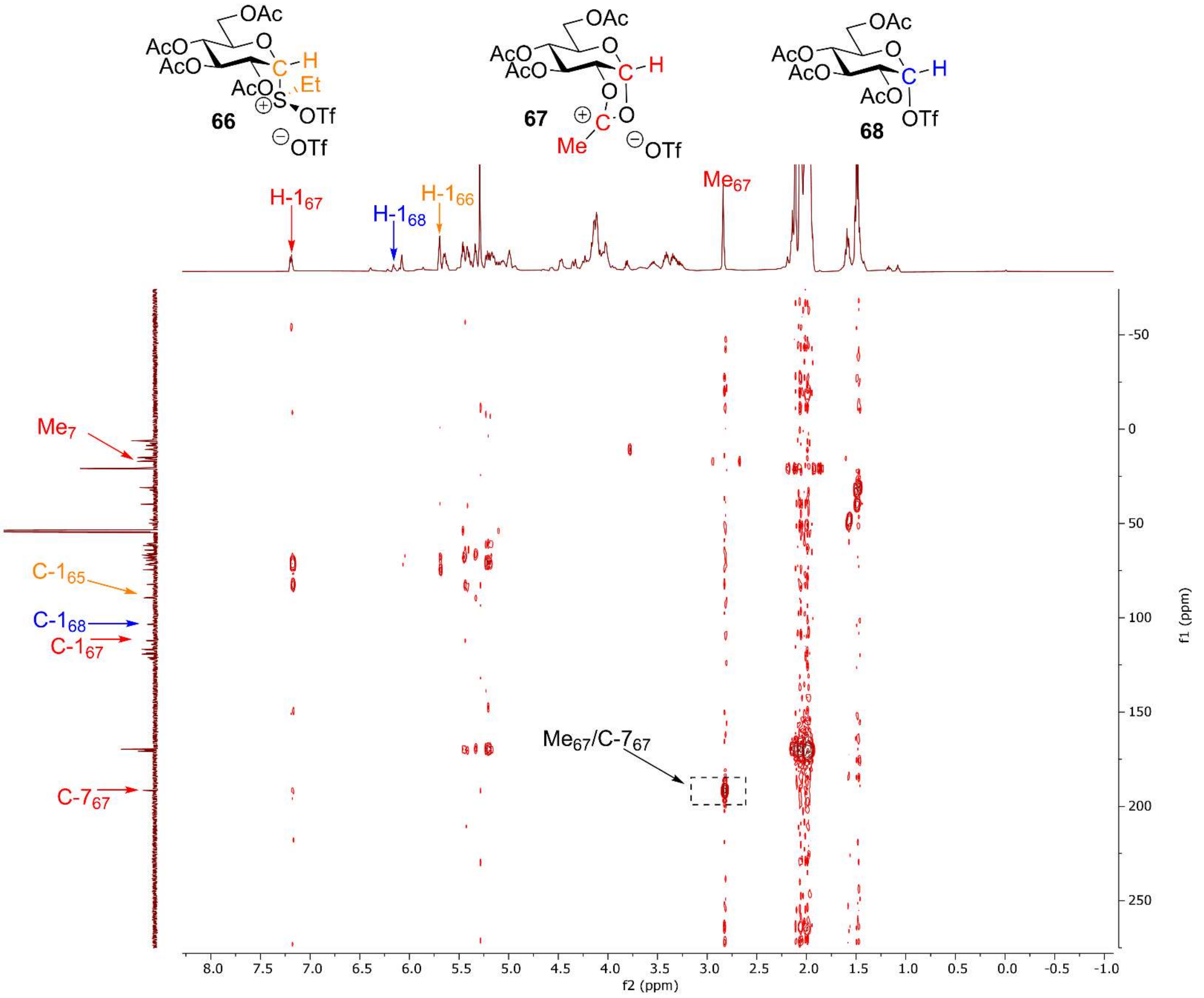

^{19}F NMR (470 MHz, CD_2Cl_2) spectrum of reaction mixture at -50 °C from VT NMR experiment with glucosyl sulfoxide 51:

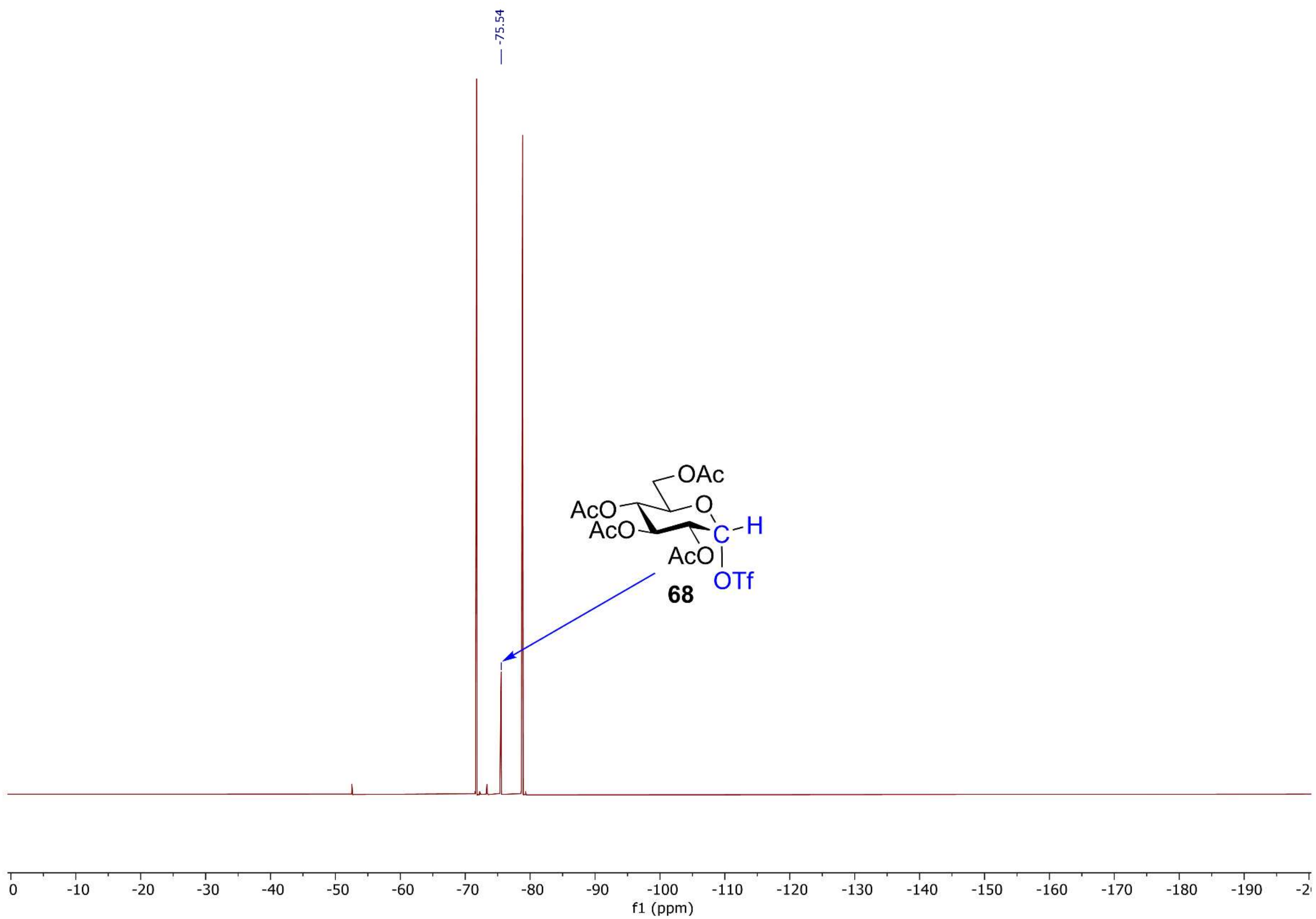

3.8. VT NMR spectra from experiments with peracetylated 5-thioglycosyl sulfoxides (40, 42)

Stacked ^{1}H NMR (500 MHz, CD_2Cl_2) spectra from VT NMR experiment with 5-thioglucosyl sulfoxides 40:

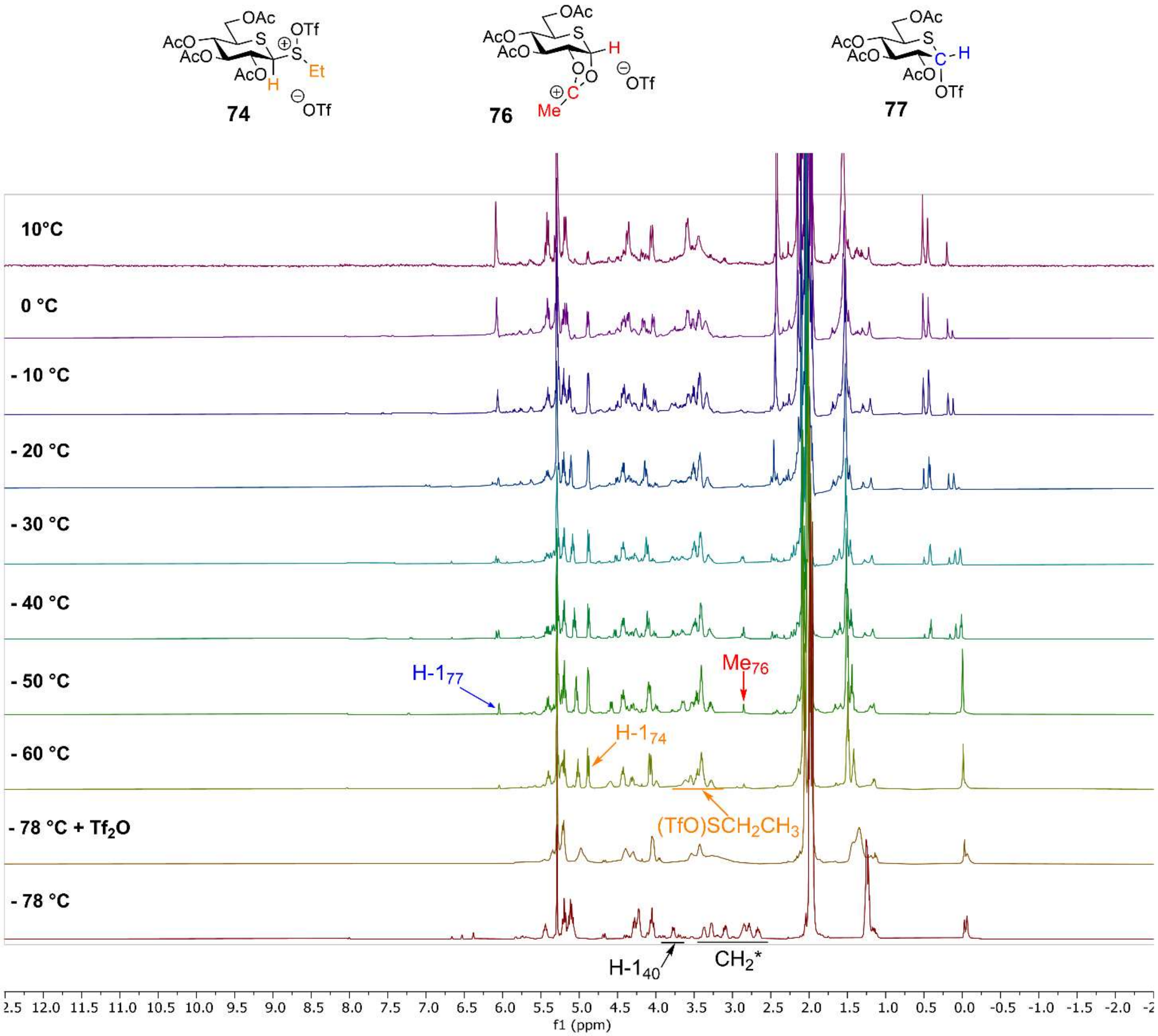

Stacked ^{19}F NMR (470 MHz, CD_2Cl_2) spectra from VT NMR experiment with 5-thioglucosyl sulfoxides 40:

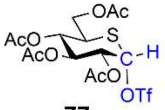

77

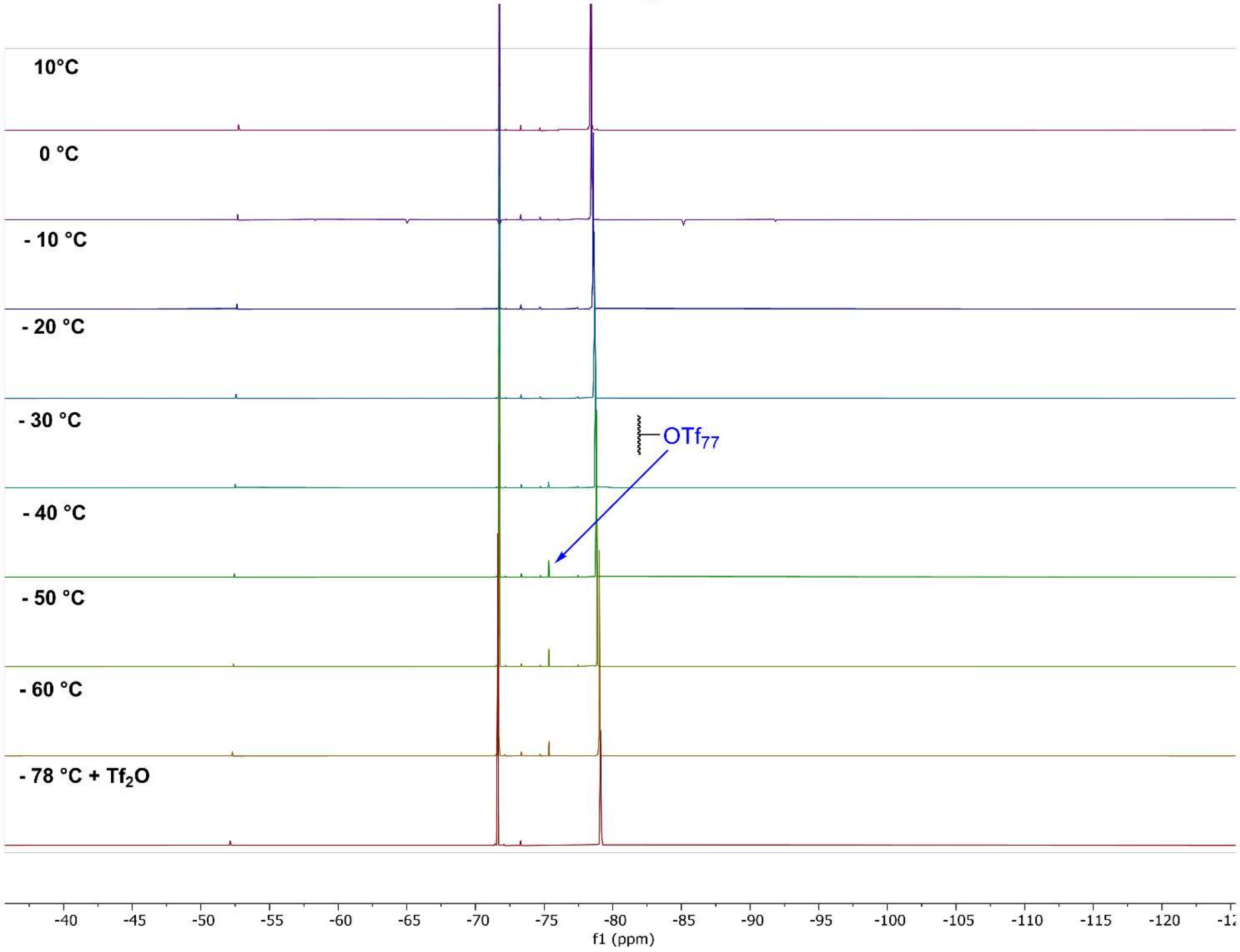

^1H NMR (500 MHz, CD_2Cl_2) spectrum of reaction mixture at -50 °C from VT NMR experiment with 5-thioglucosyl sulfoxides 40:

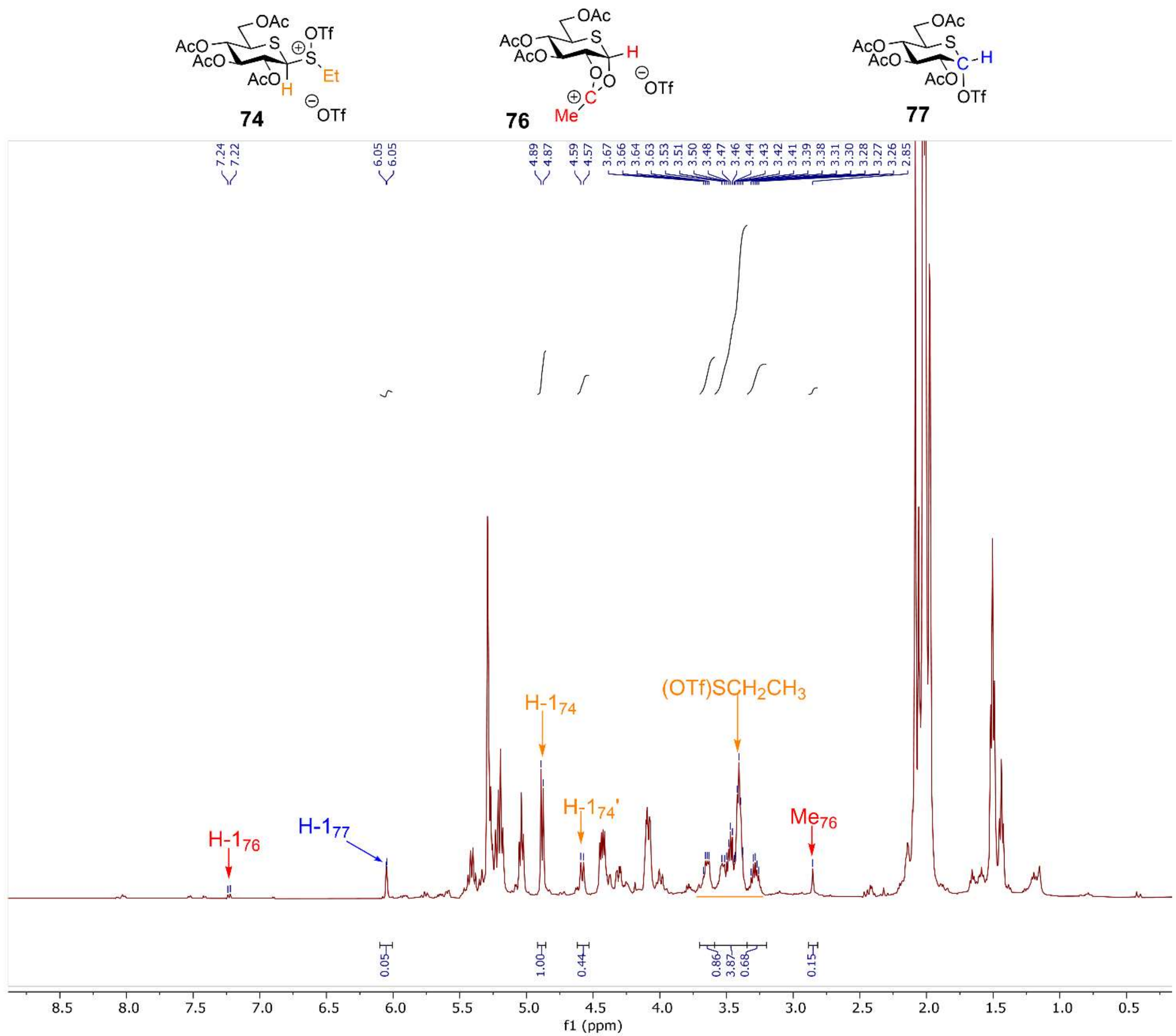

^{13}C NMR (125.67 MHz, CD_2Cl_2) spectrum of reaction mixture at -50 °C from VT NMR experiment with 5-thioglucosyl sulfoxides 40:

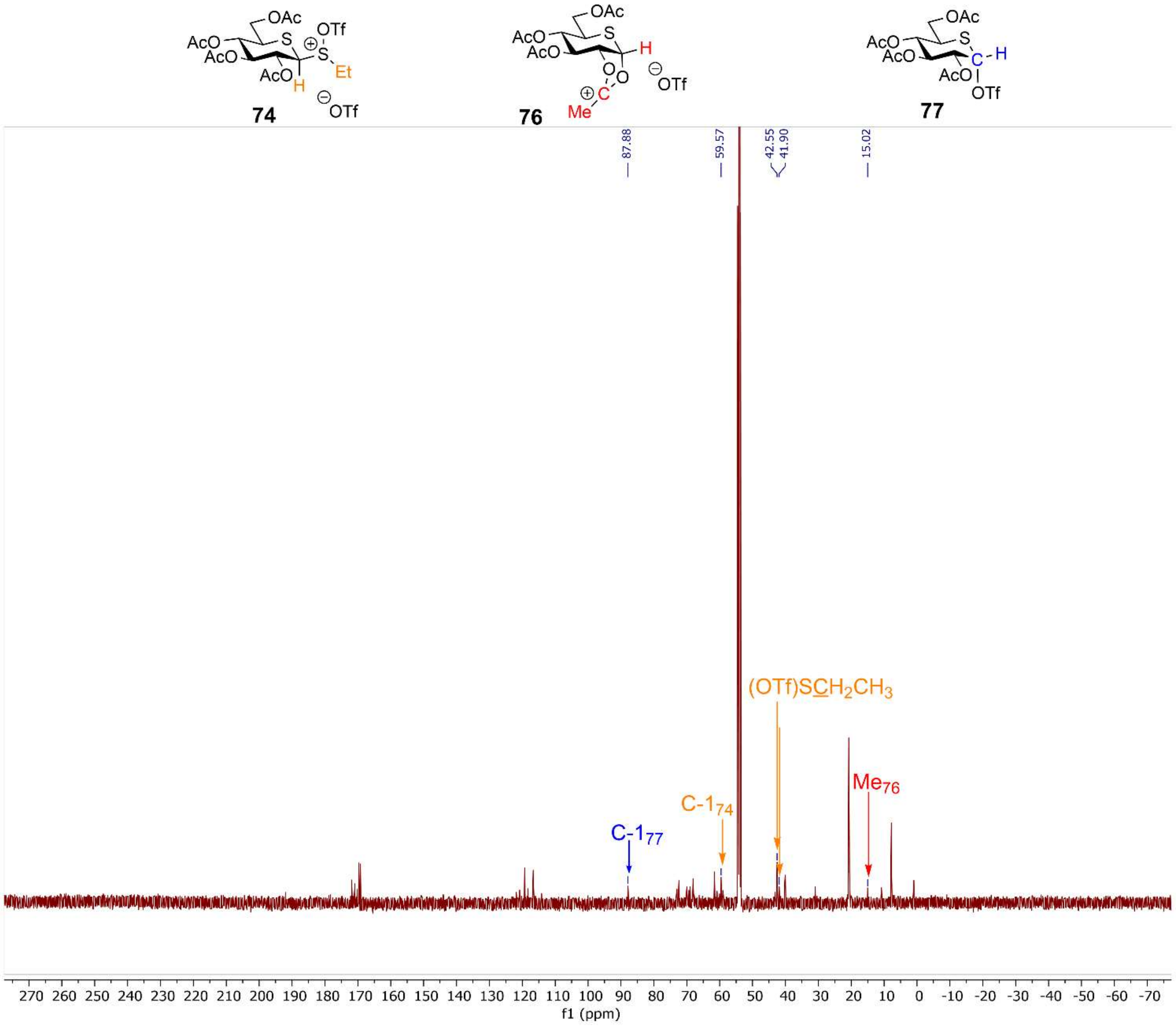

DEPT-135 (CD_2Cl_2) spectrum of reaction mixture at -50 °C from VT NMR experiment with 5-thioglucosyl sulfoxides 40:

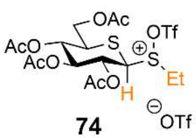

74

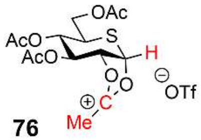

76

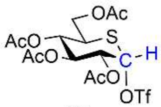

77

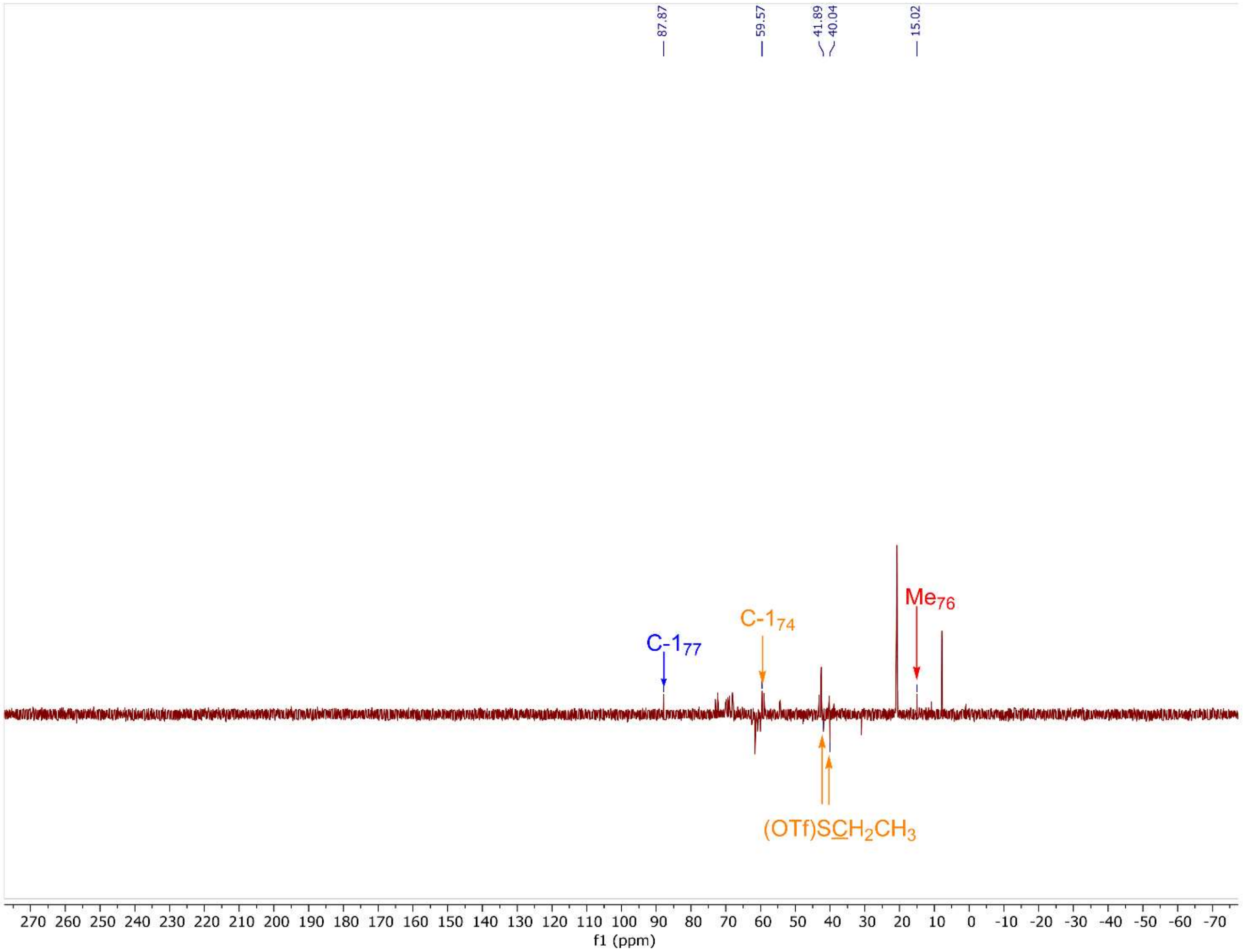

COSY (CD_2Cl_2) spectrum of reaction mixture at -50 °C from VT NMR experiment with 5-thioglucosyl sulfoxides 40:

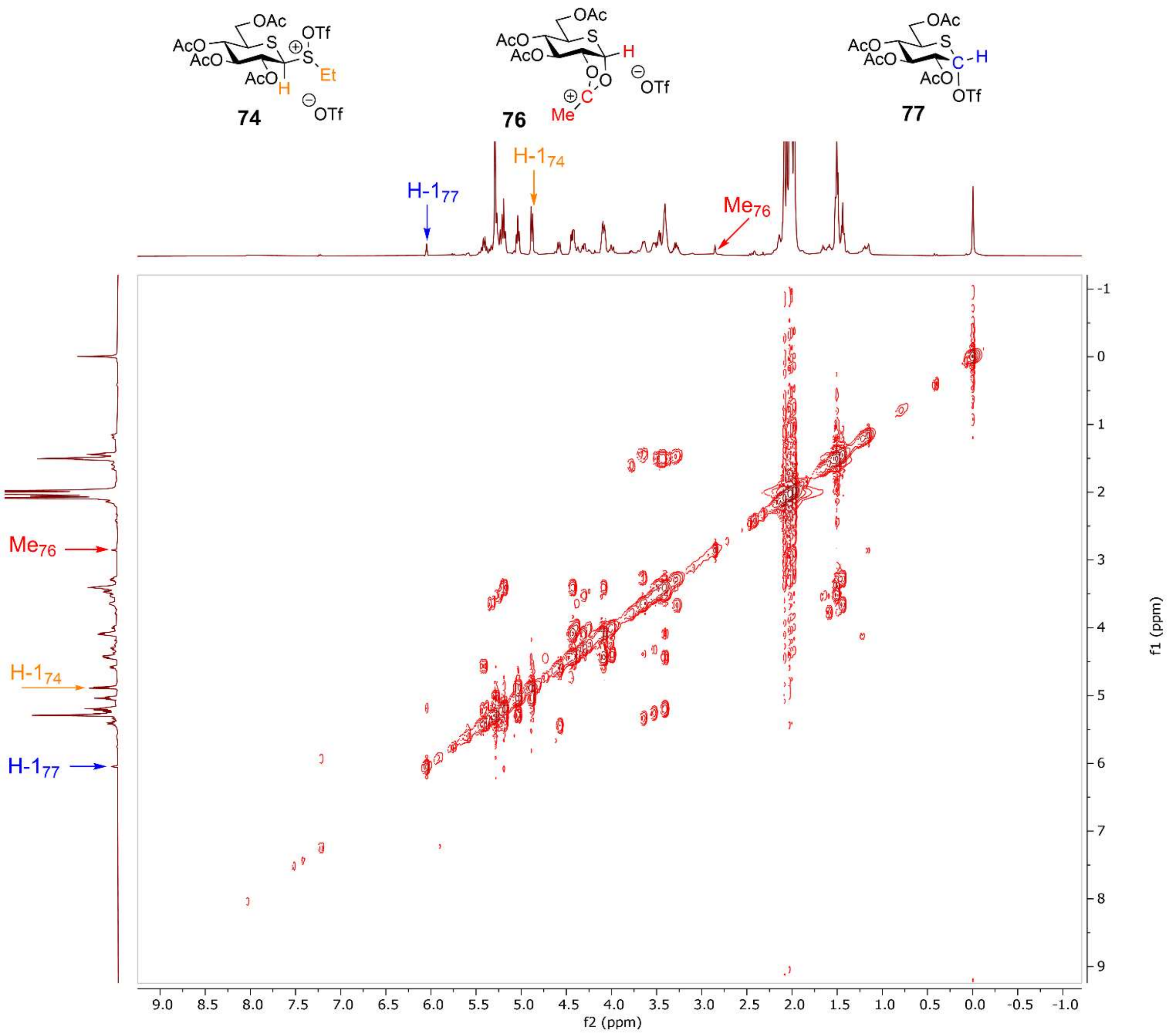

HMQC (CD_2Cl_2) spectrum of reaction mixture at -50 °C from VT NMR experiment with 5-thioglucosyl sulfoxides 40:

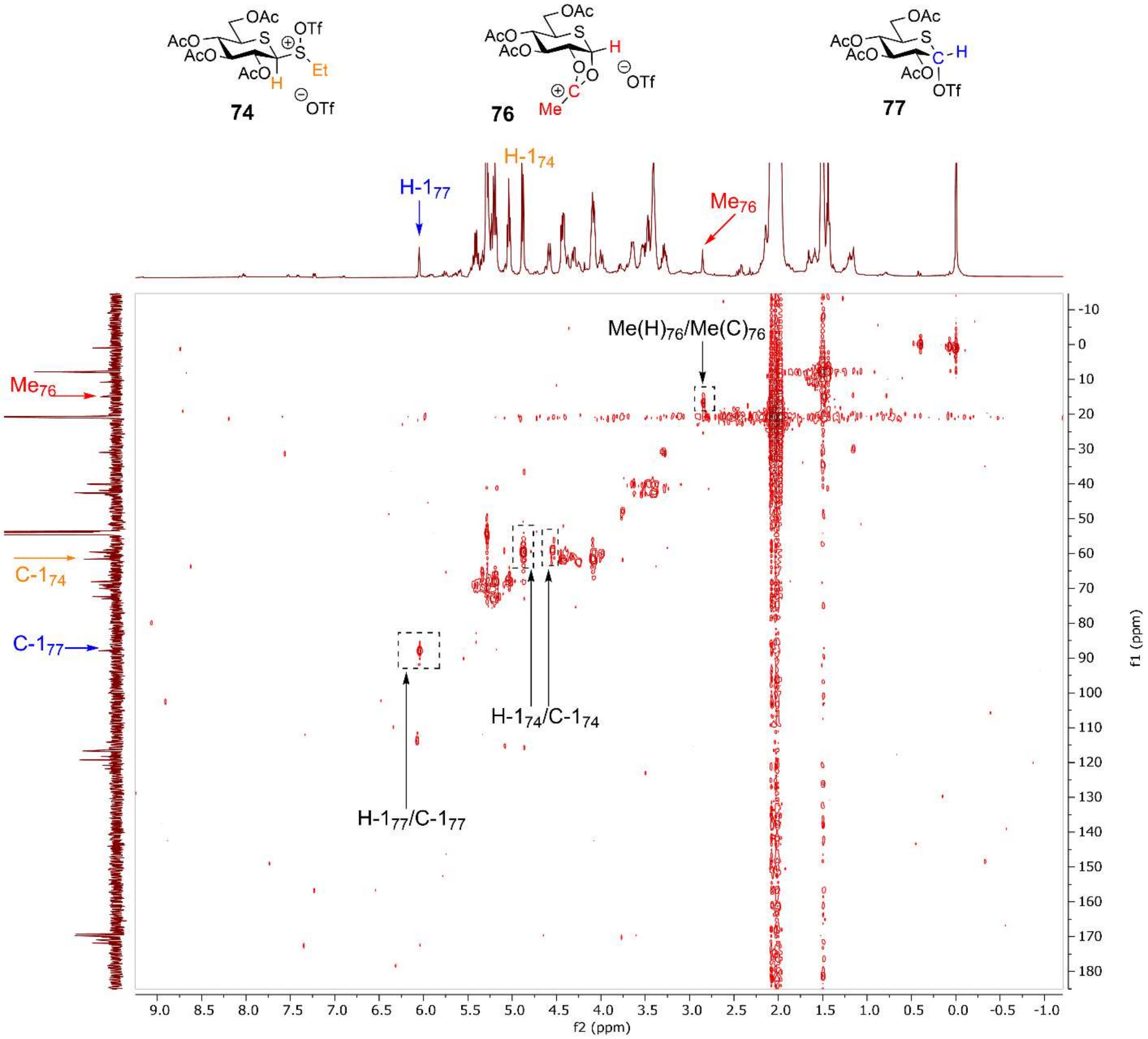

HMBC (CD_2Cl_2) spectrum of reaction mixture at -50 °C from VT NMR experiment with 5-thioglucosyl sulfoxides 40:

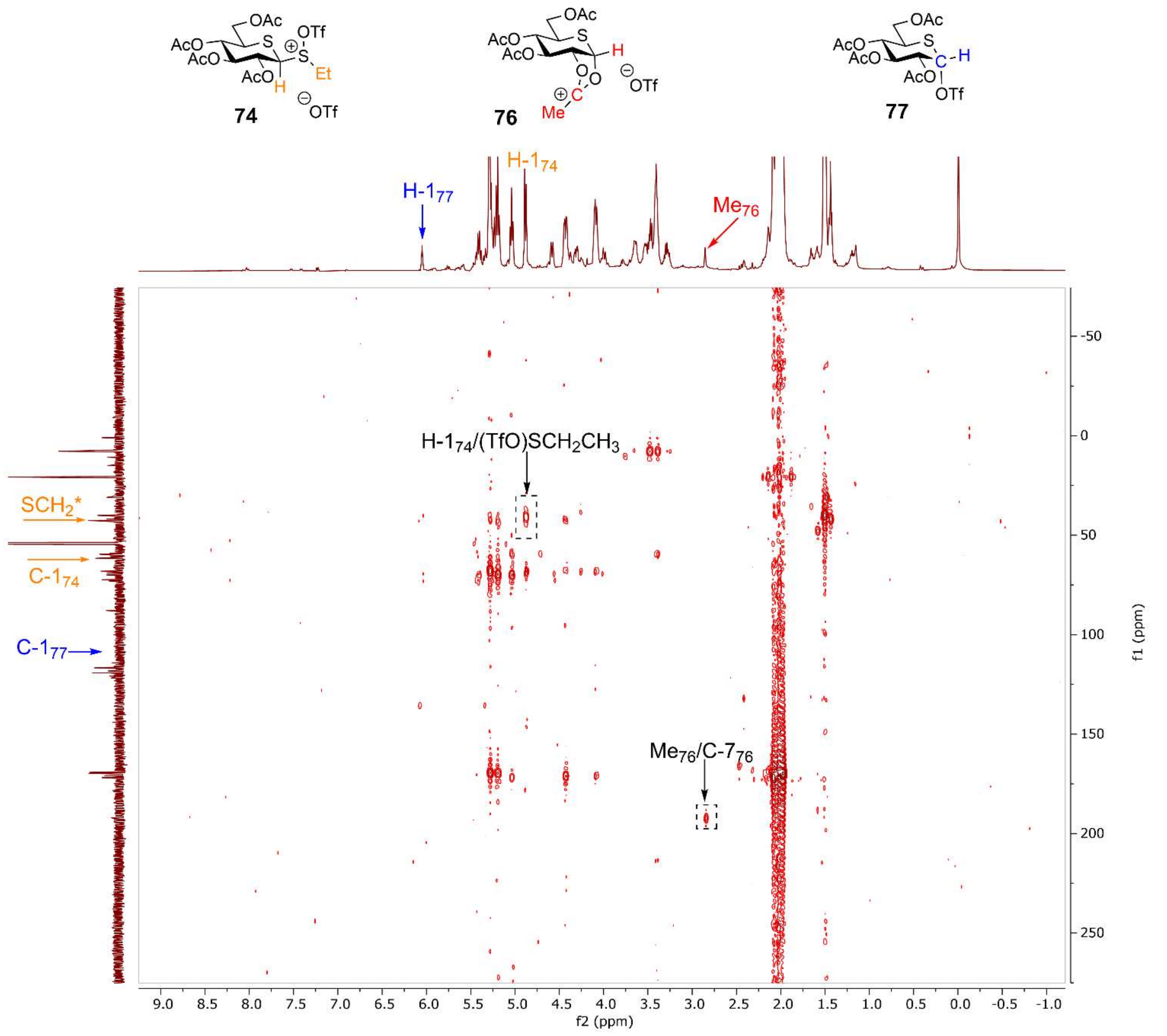

^{19}F NMR (470 MHz, CD_2Cl_2) spectrum of reaction mixture at -50 °C from VT NMR experiment with 5-thioglucosyl sulfoxides 40:

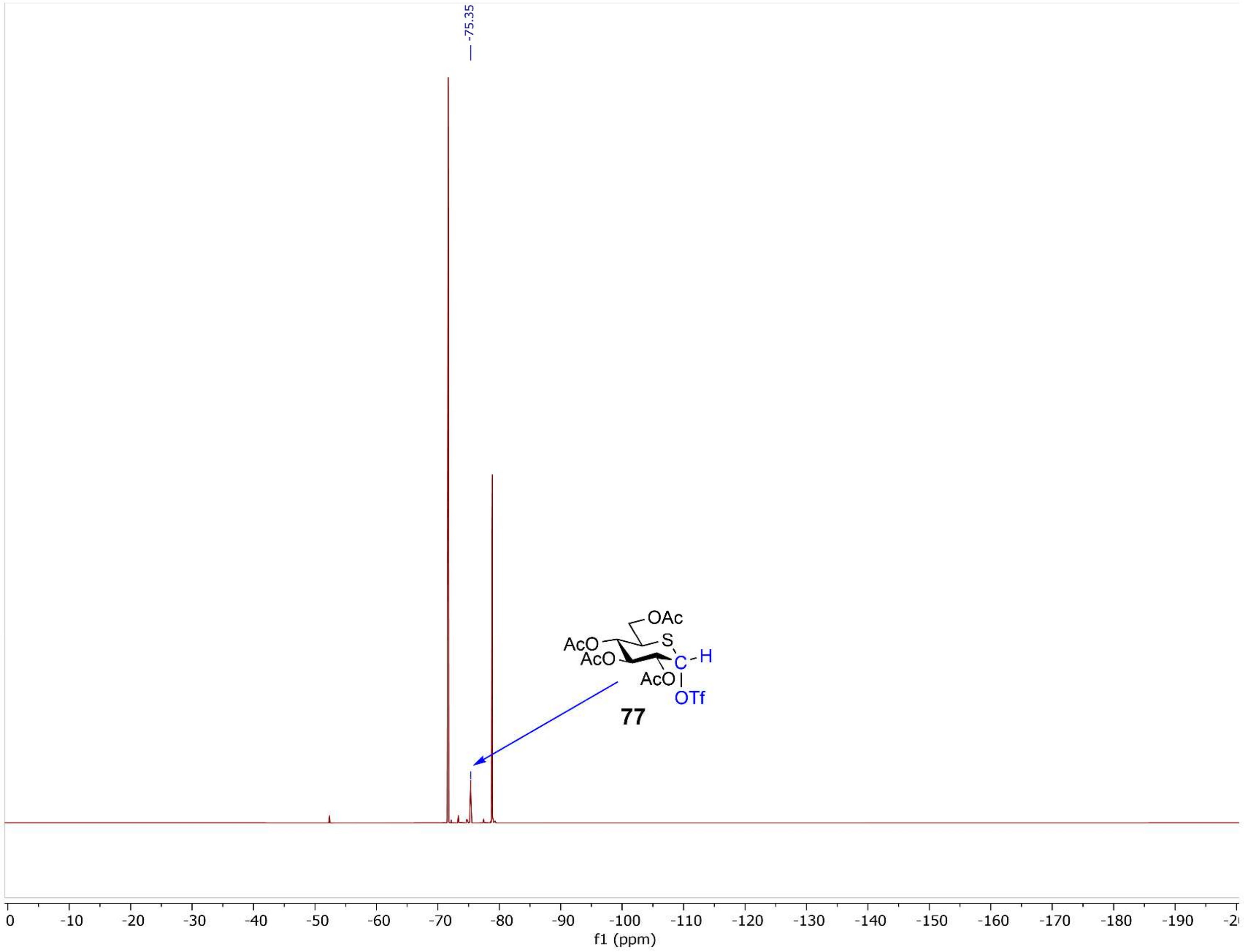

Stacked ^{1}H NMR (500 MHz, CD_2Cl_2) spectra from VT NMR experiment with 5-thioglucosyl sulfoxides 42:

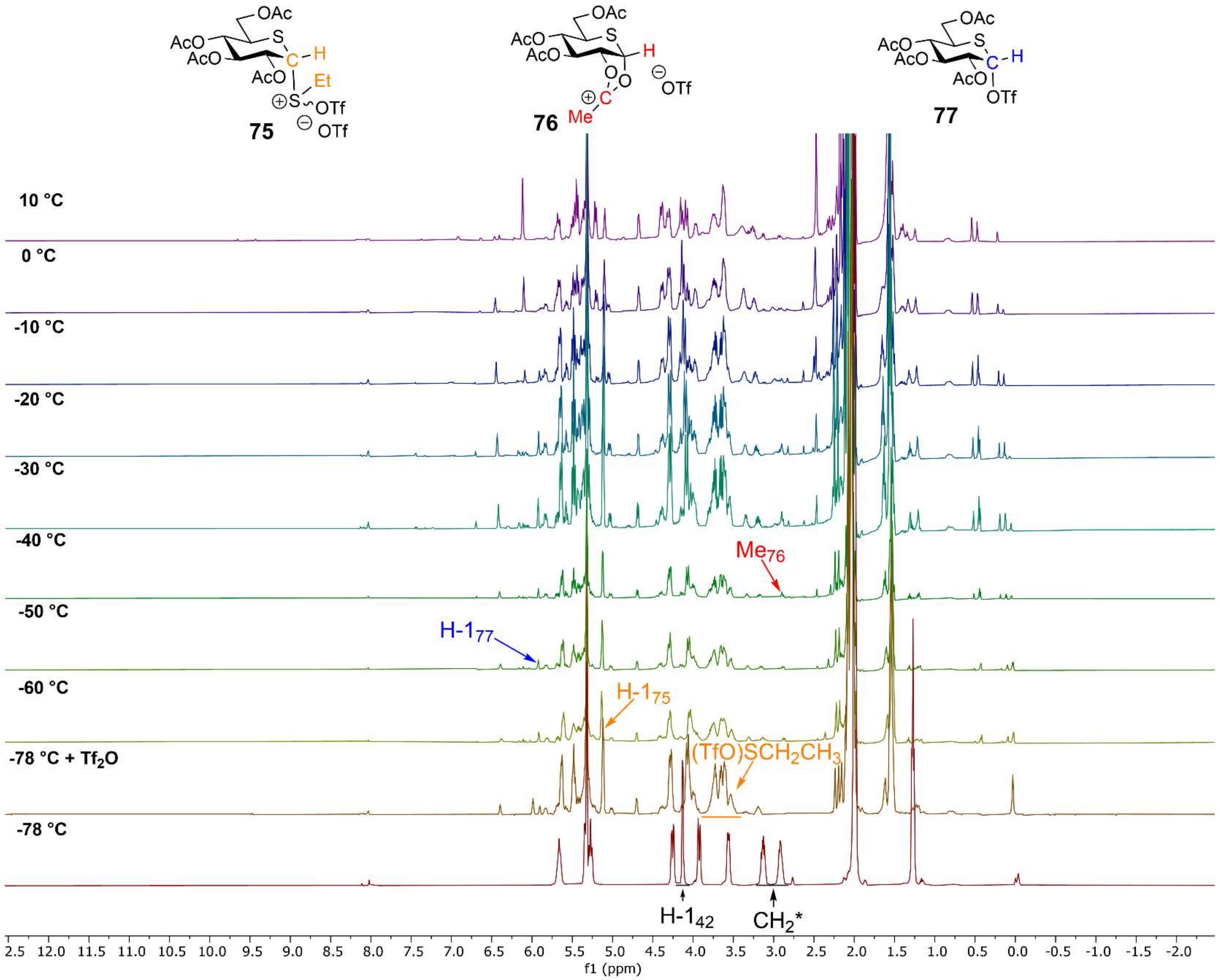

Stacked ^{19}F NMR (470 MHz, CD_2Cl_2) spectra from VT NMR experiment with 5-thioglucosyl sulfoxides 42:

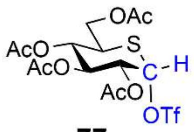

77

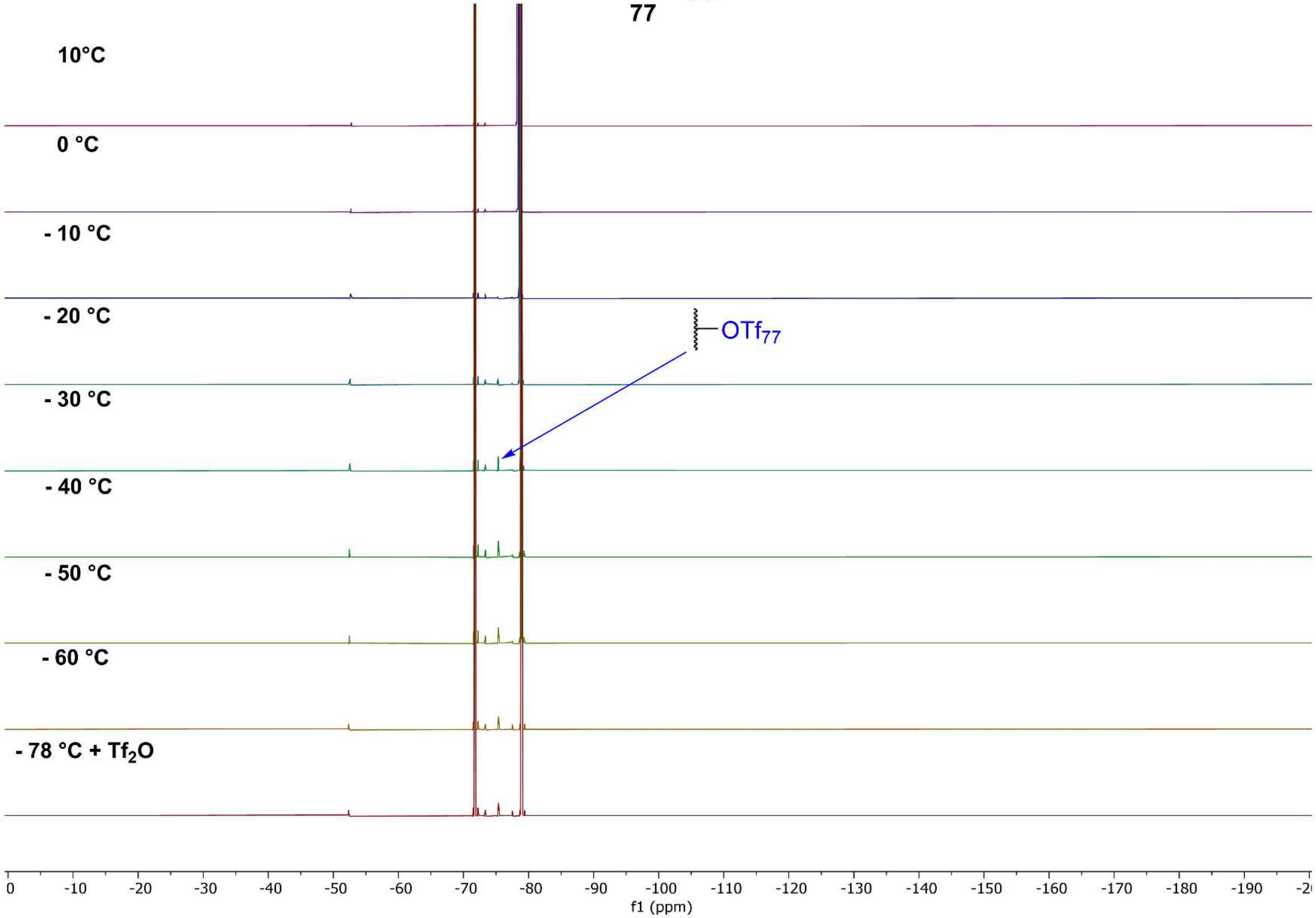

^{1}H NMR (500 MHz, CD_2Cl_2) spectrum of reaction mixture at -50 °C from VT NMR experiment with 5-thioglucosyl sulfoxides 42:

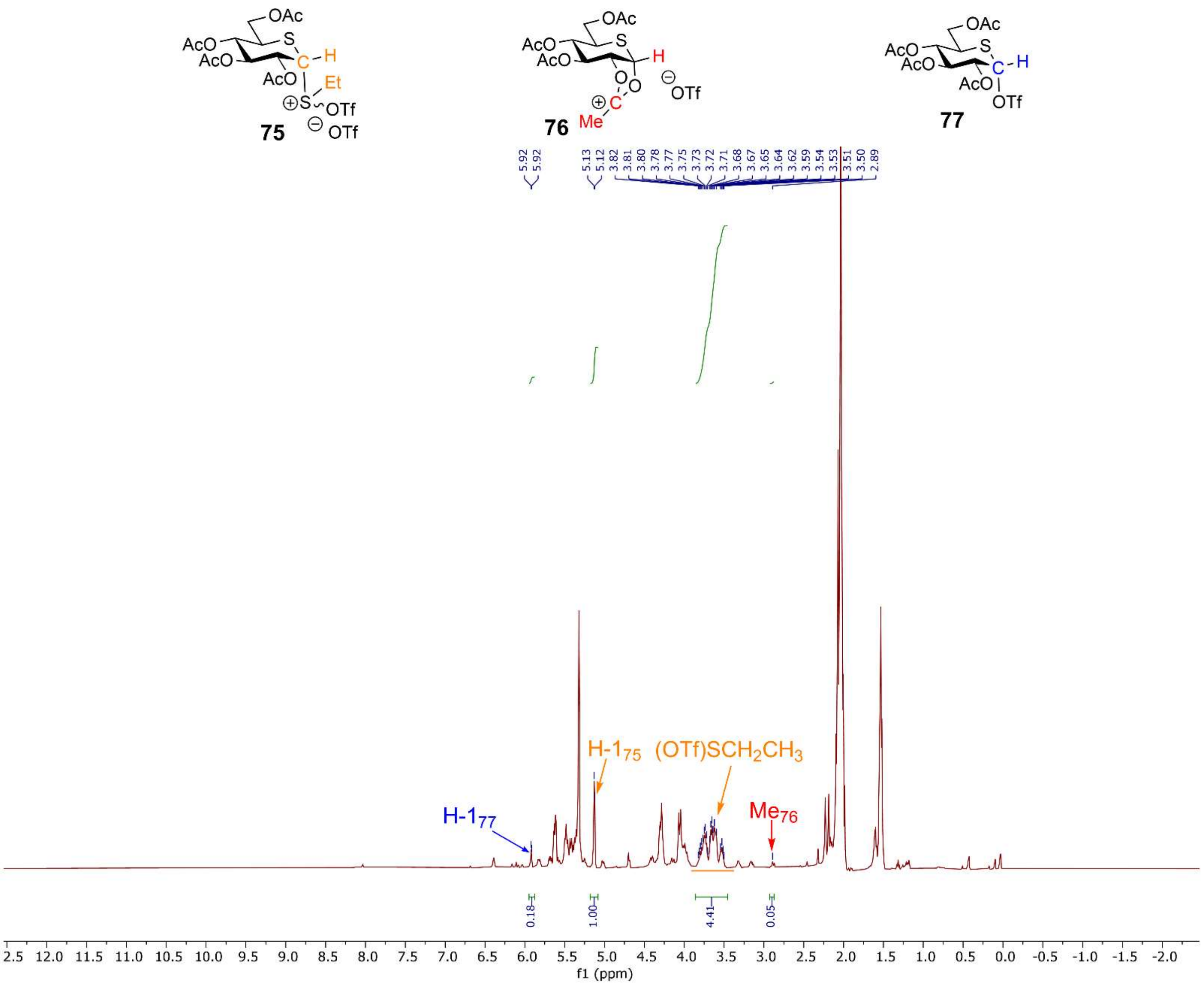

^{13}C NMR (125.67 MHz, CD_2Cl_2) spectrum of reaction mixture at -50 °C from VT NMR experiment with 5-thioglucosyl sulfoxides 42:

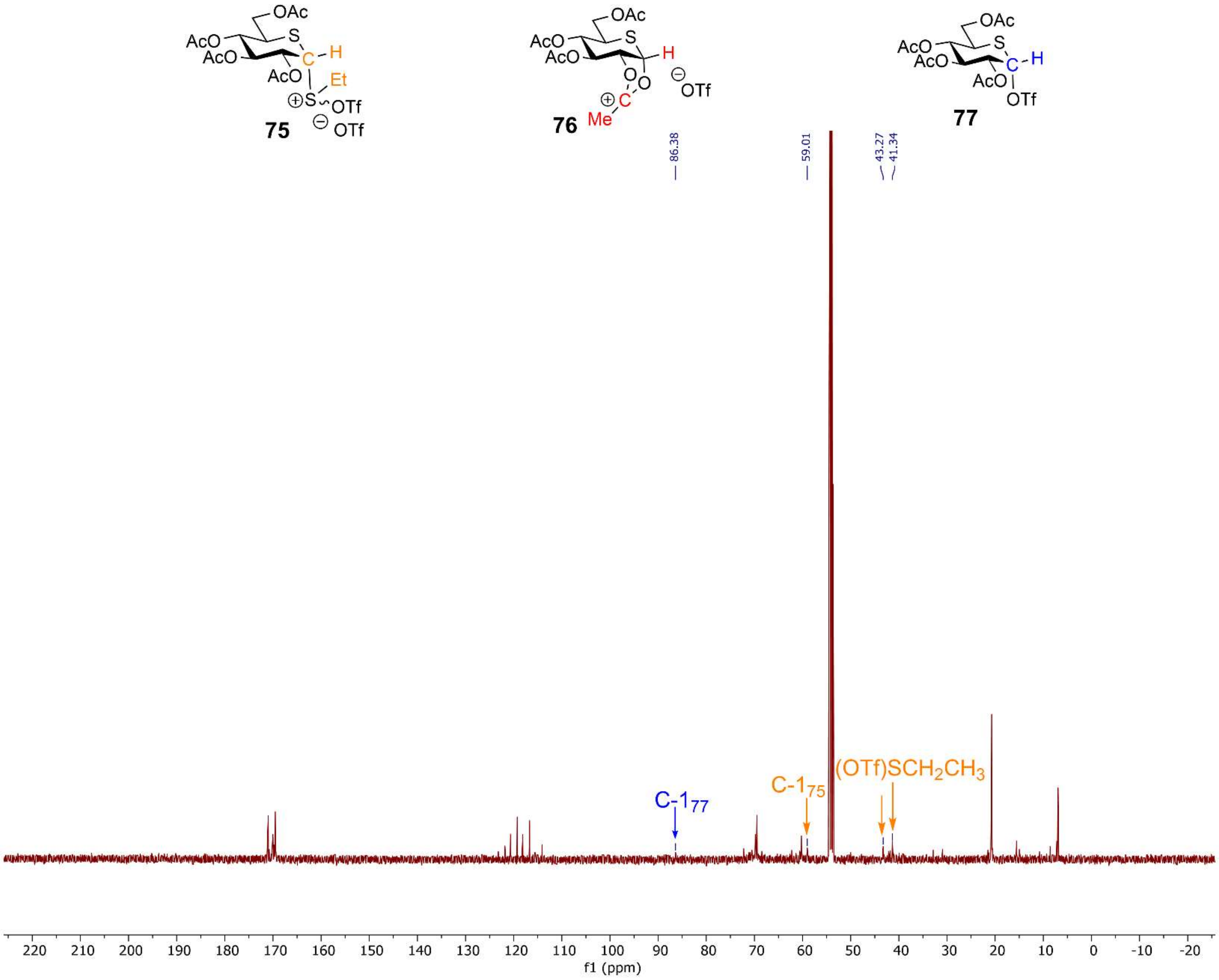

DEPT-135 (CD_2Cl_2) spectrum of reaction mixture at -50 °C from VT NMR experiment with 5-thioglucosyl sulfoxides 42:

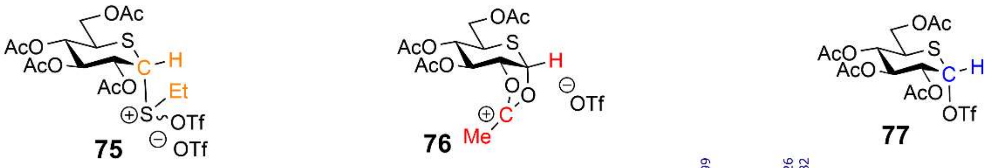

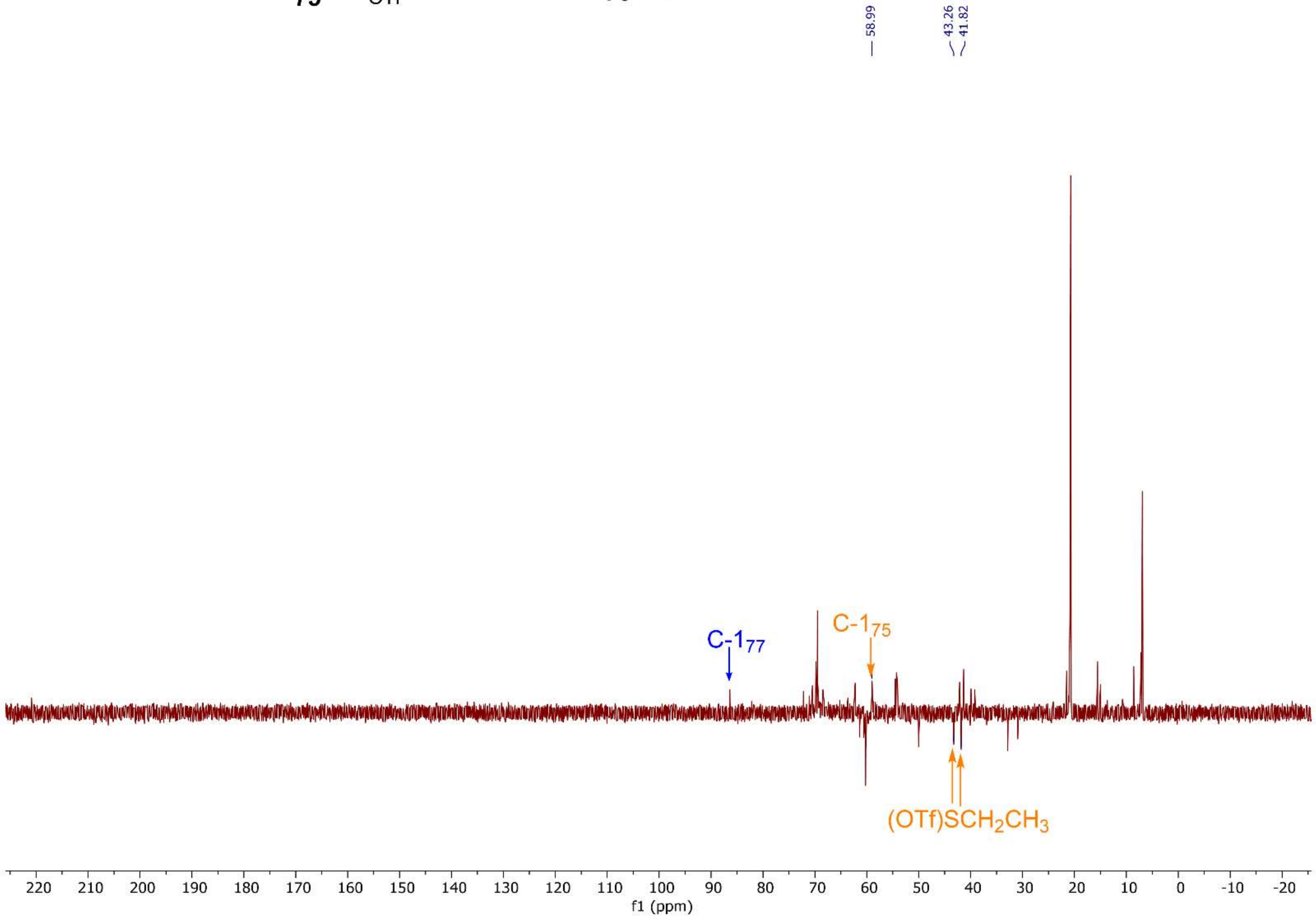

COSY (CD₂Cl₂) spectrum of reaction mixture at -50 °C from VT NMR experiment with 5-thioglucosyl sulfoxides 42:

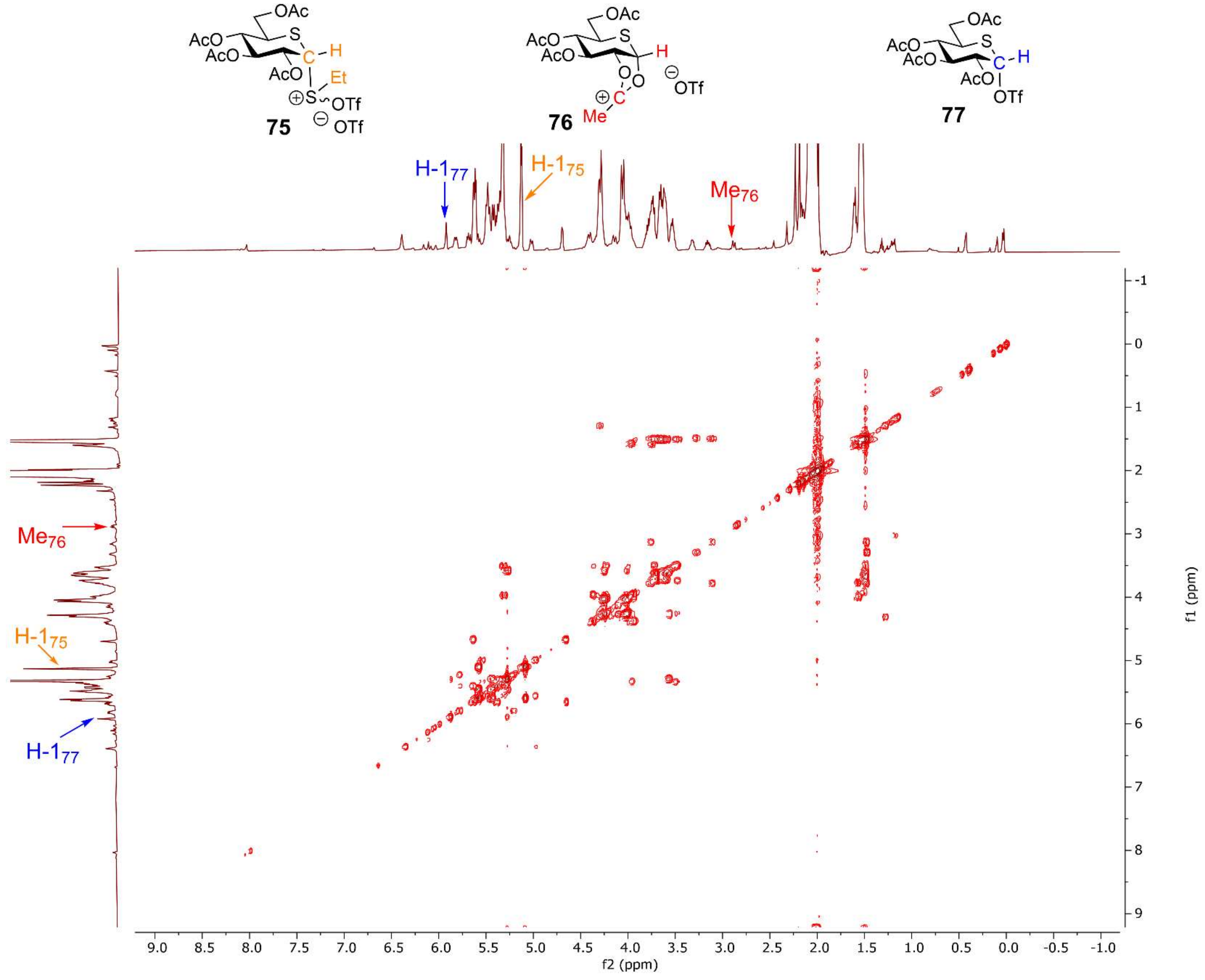

HMQC (CD_2Cl_2) spectrum of reaction mixture at -50 °C from VT NMR experiment with 5-thioglucosyl sulfoxides 42:

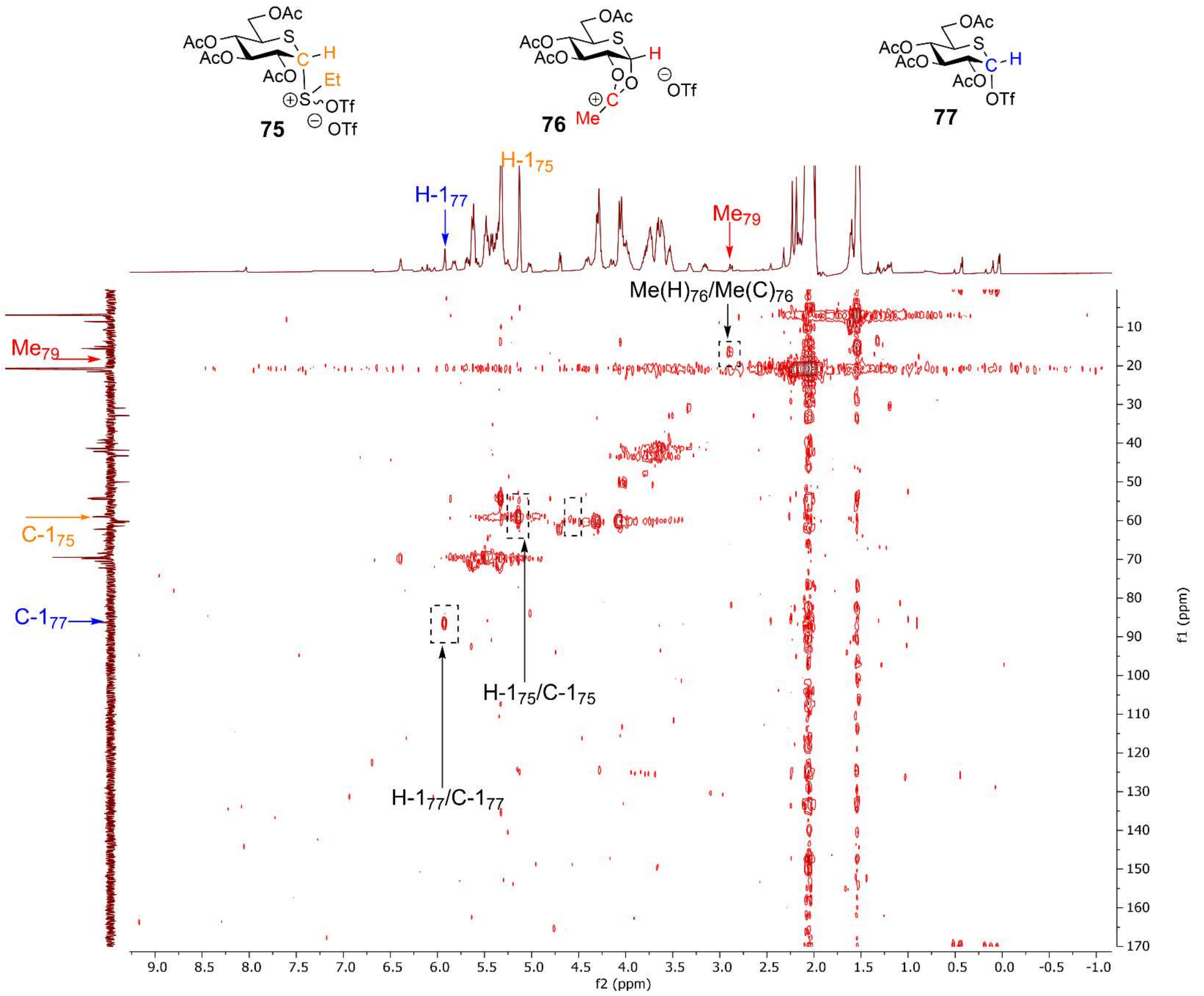

HMBC (CD_2Cl_2) spectrum of reaction mixture at -50 °C from VT NMR experiment with 5-thioglucosyl sulfoxides 42:

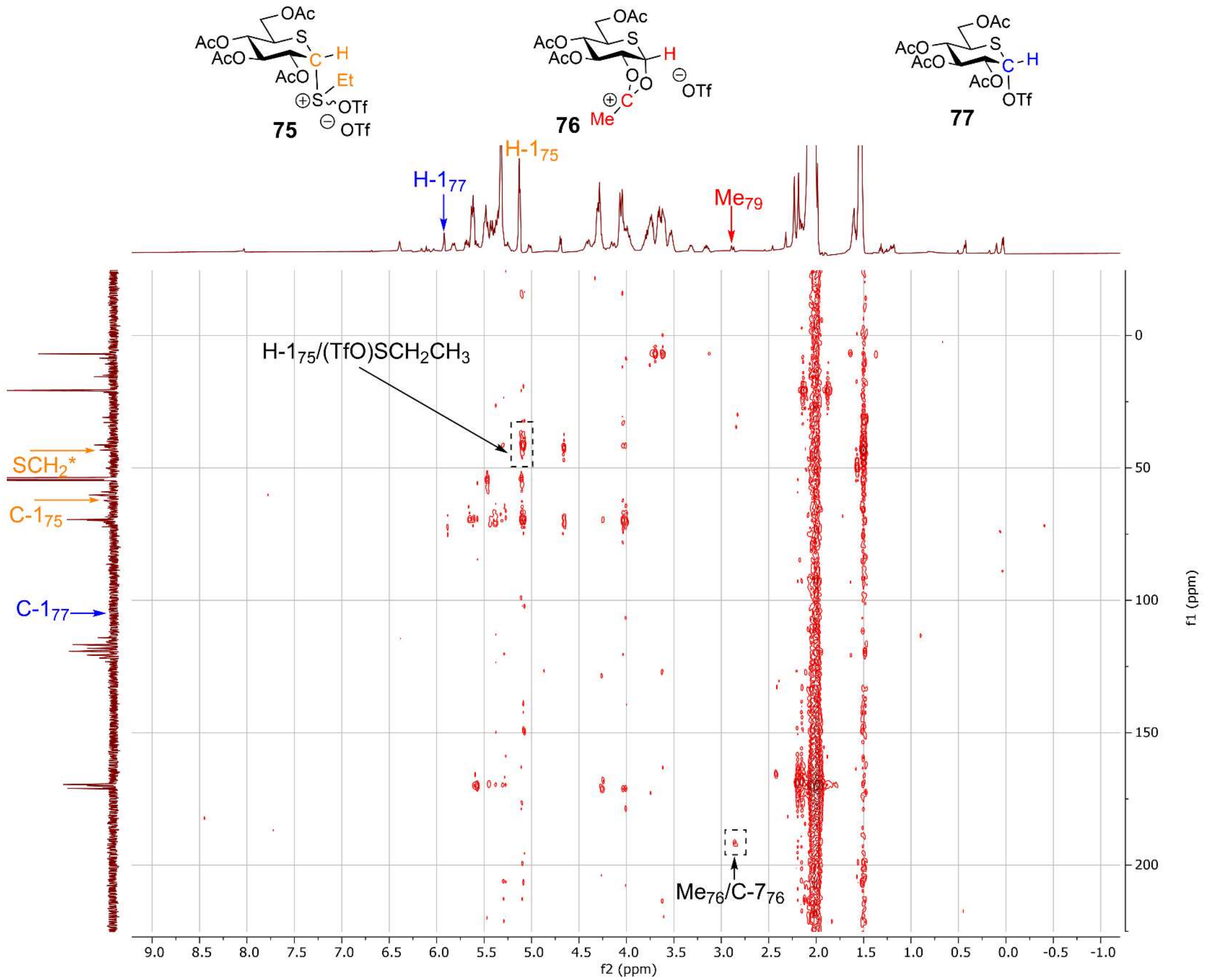

^{19}F NMR (470 MHz, CD_2Cl_2) spectrum of reaction mixture at -50 °C from VT NMR experiment with 5-thioglucosyl sulfoxides 42:

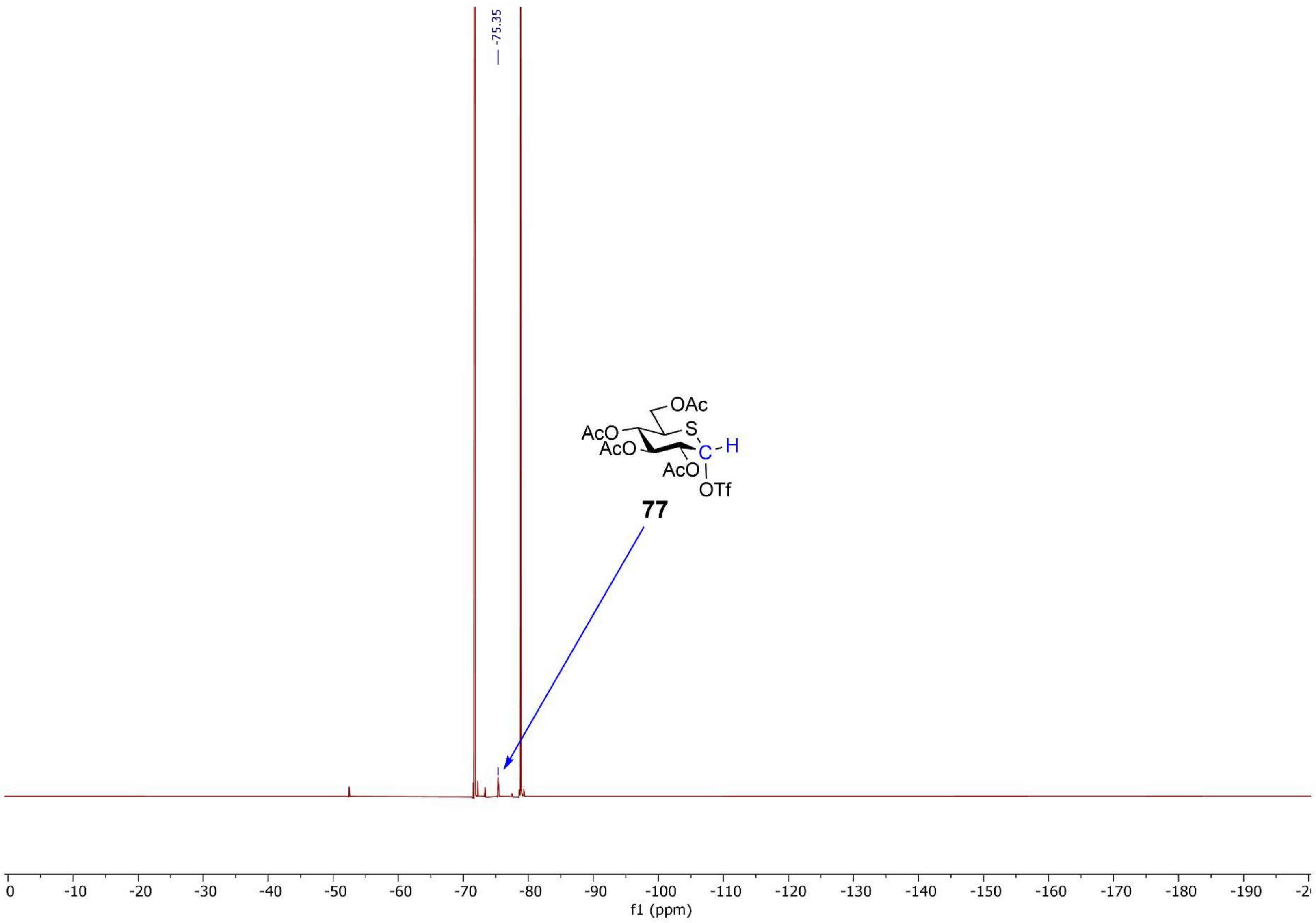

3.9. VT NMR spectra from experiments with peracetylated trichloroacetimidates (57, 1)

Stacked ^{1}H NMR (500 MHz, CD_2Cl_2) spectra from VT NMR experiment with glucosyl trichloroacetimidate 57:

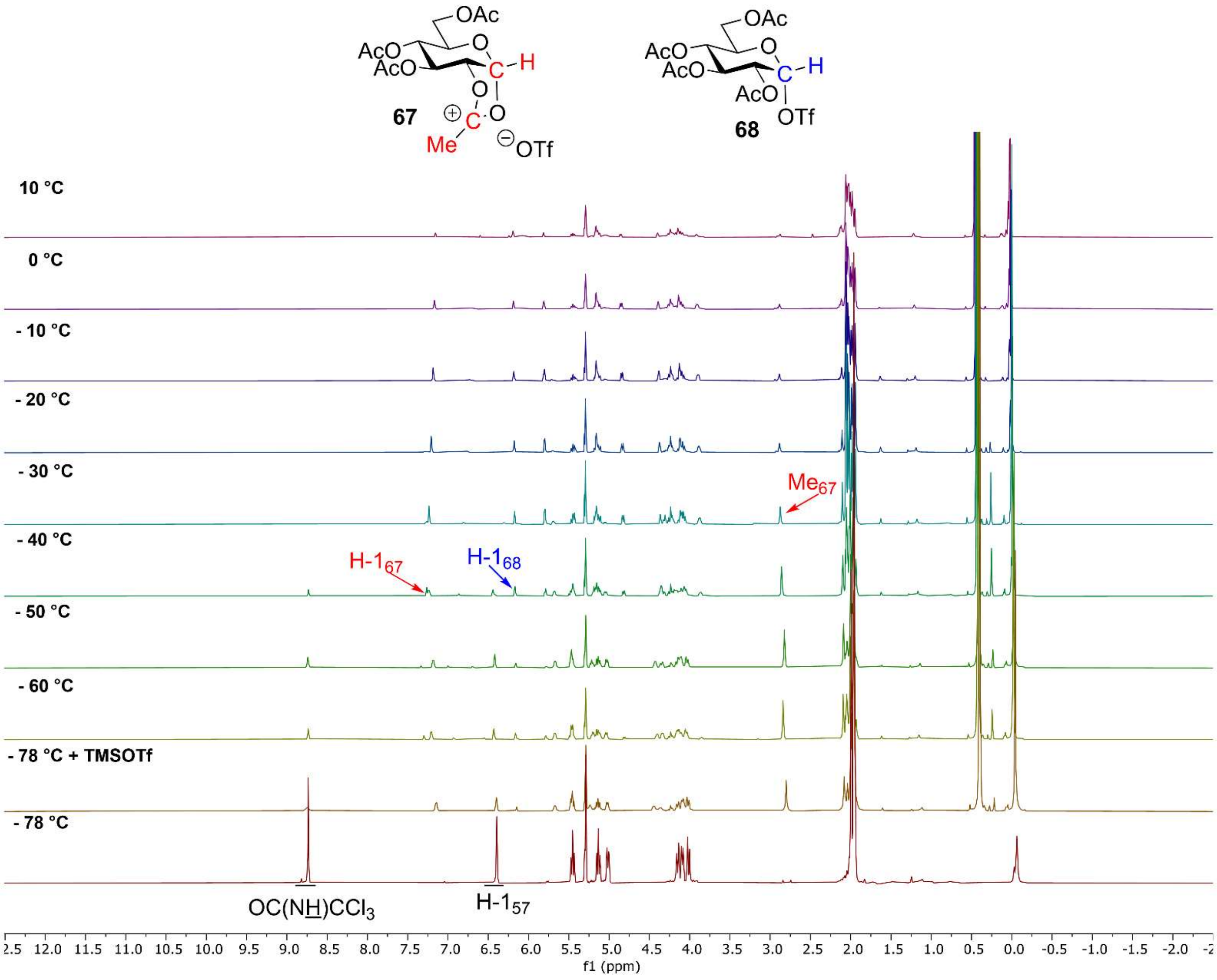

Stacked ^{19}F NMR (470 MHz, CD_2Cl_2) spectra from VT NMR experiment with glucosyl trichloroacetimidate 57:

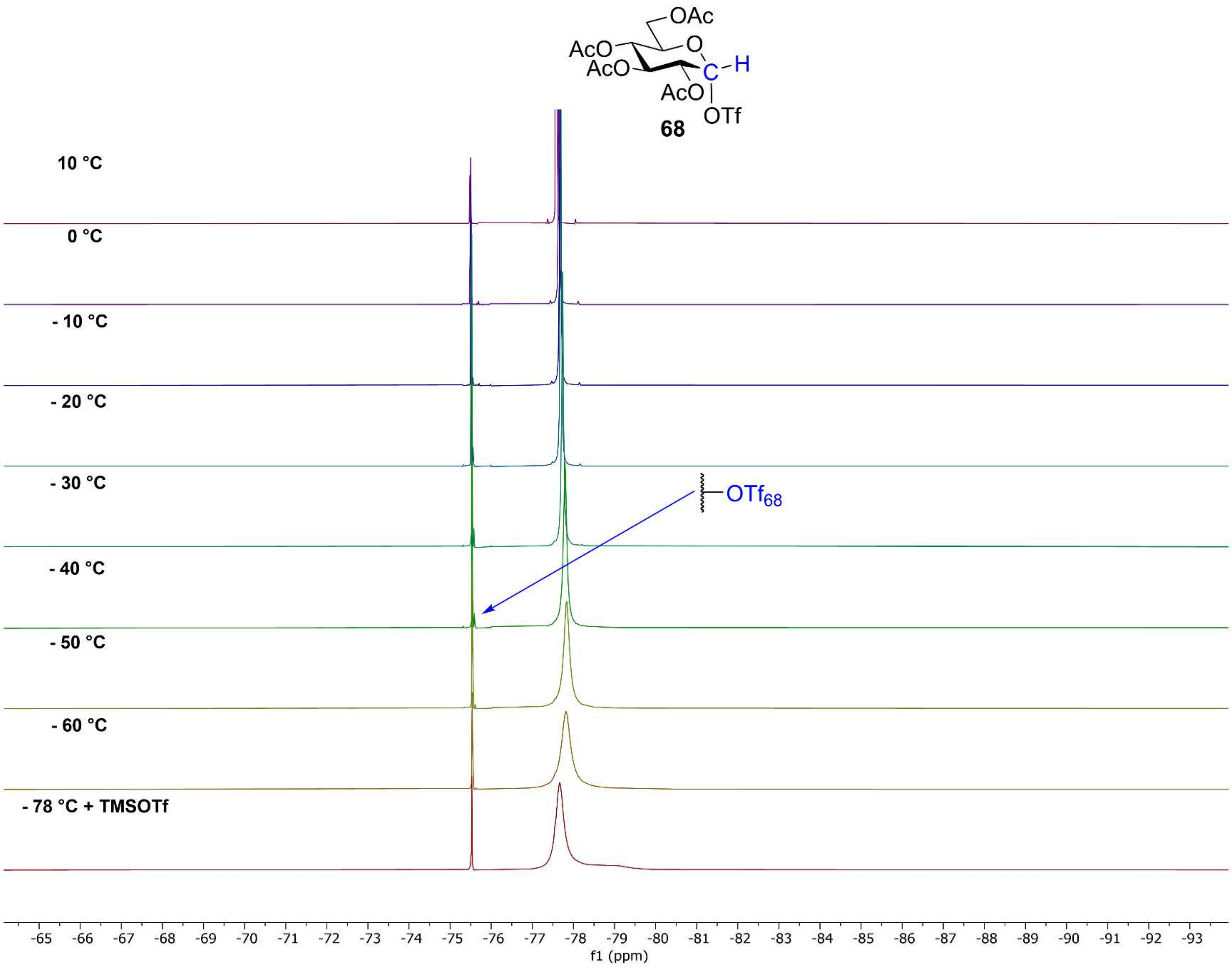

^{1}H NMR (500 MHz, CD_2Cl_2) spectrum of reaction mixture at -40 °C from VT NMR experiment with glucosyl trichloroacetimidate 57:

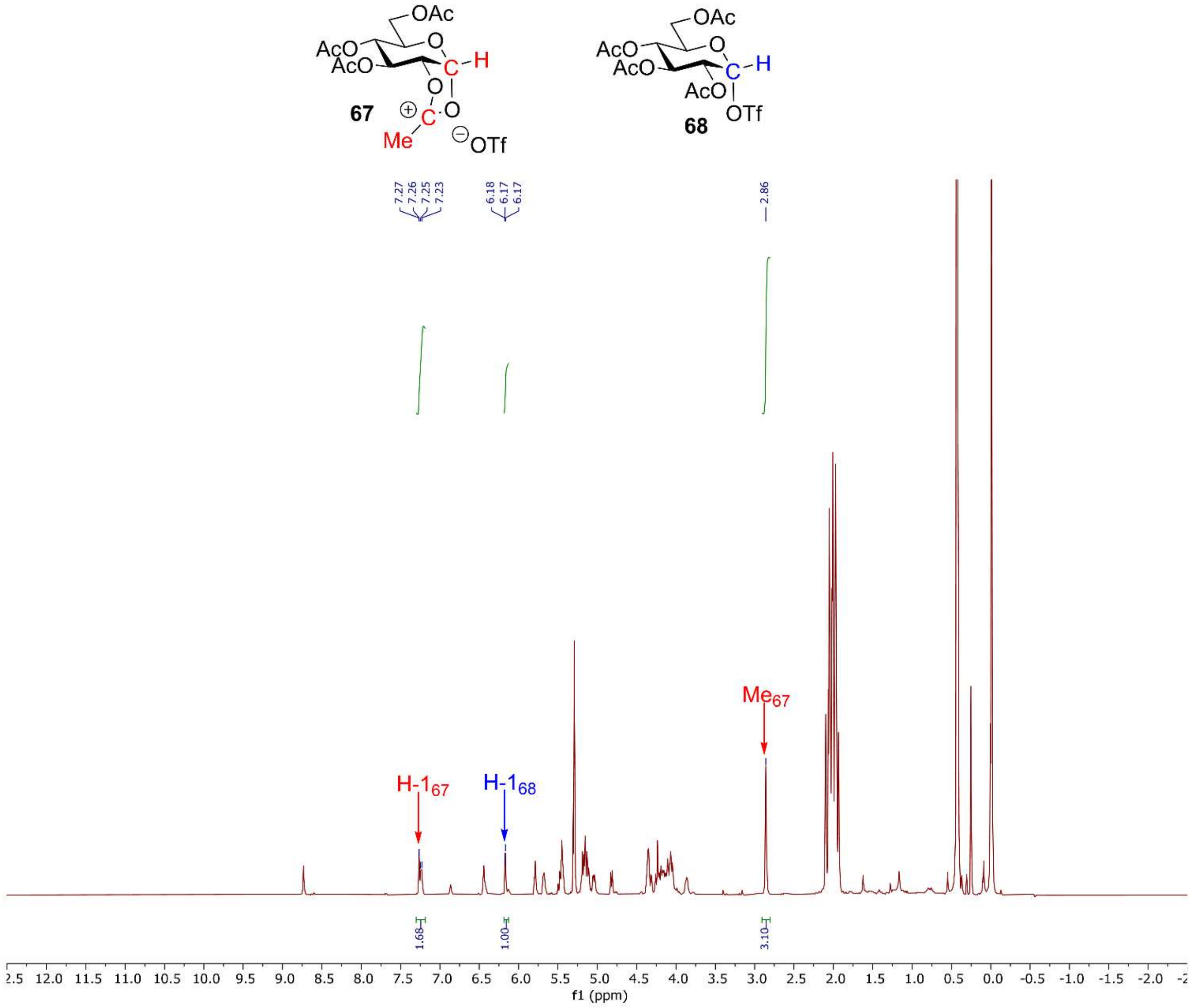

^{13}C NMR (125.67 MHz, CD_2Cl_2) spectrum of reaction mixture at -40 °C from VT NMR experiment with glucosyl trichloroacetimidate 57:

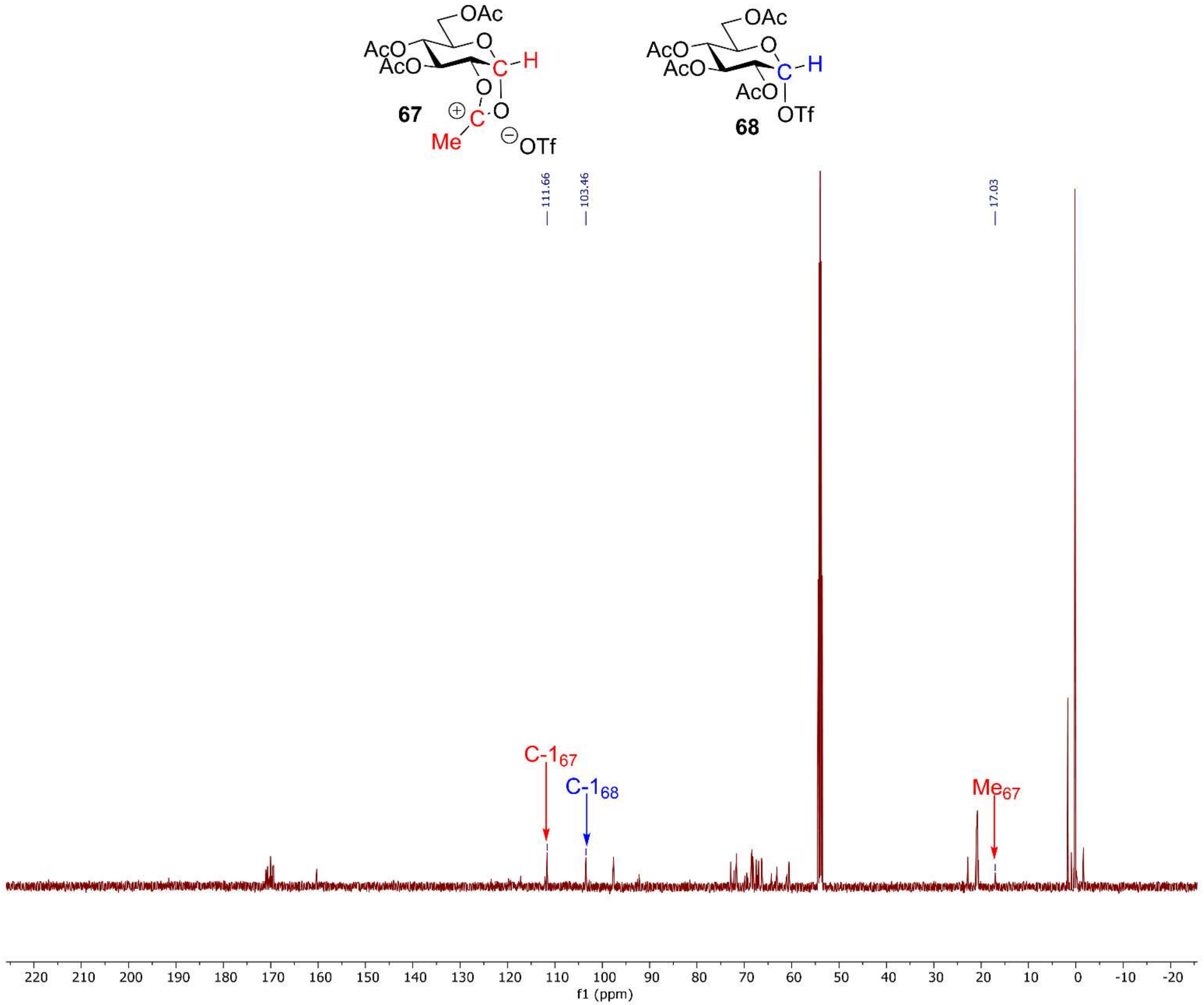

DEPT-135 (CD_2Cl_2) spectrum of reaction mixture at -40 °C from VT NMR experiment with glucosyl trichloroacetimidate 57:

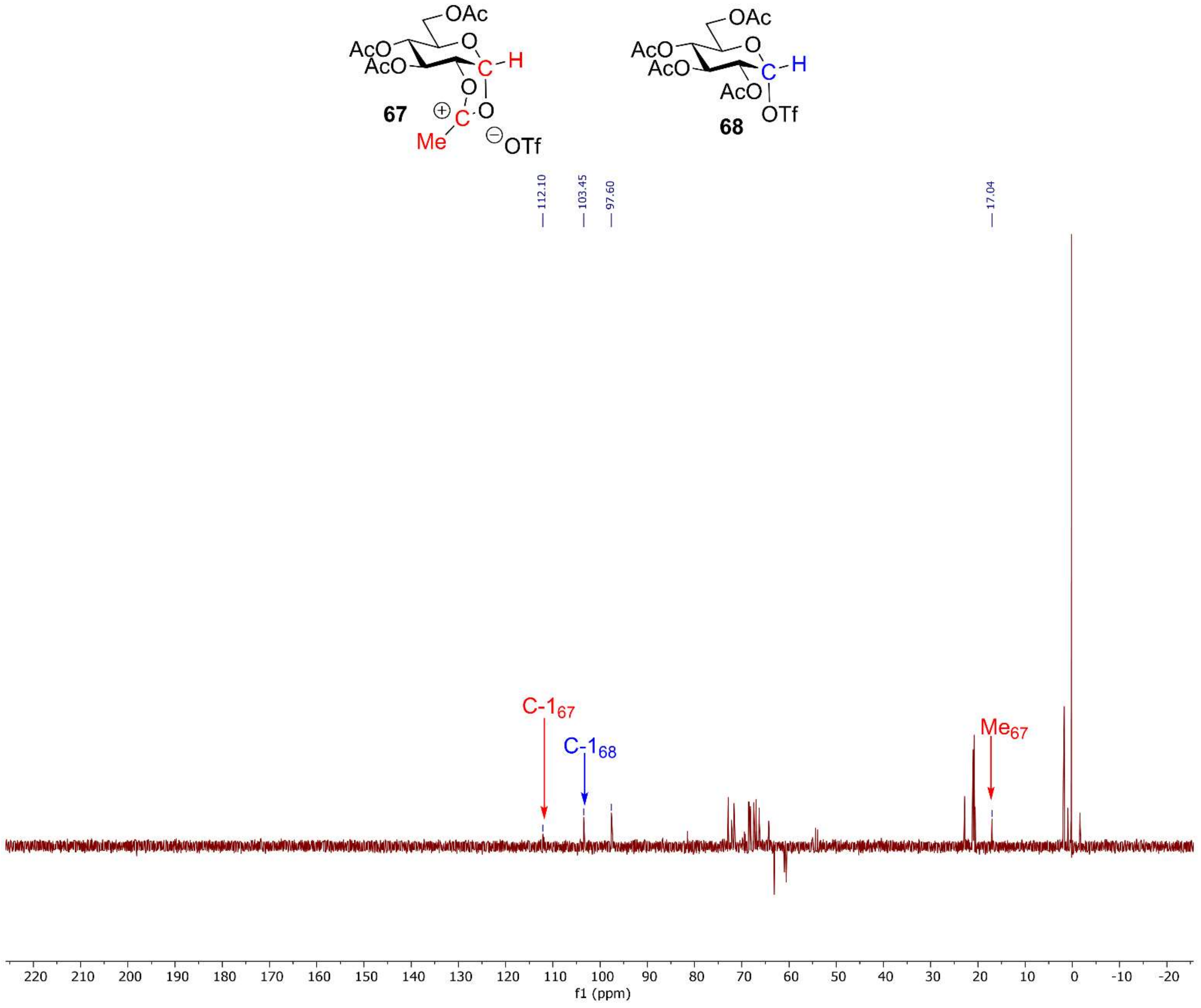

HSQC (CD_2Cl_2) spectrum of reaction mixture at -40 °C from VT NMR experiment with glucosyl trichloroacetimidate 57:

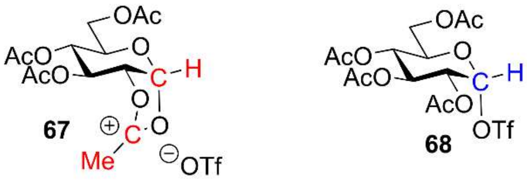

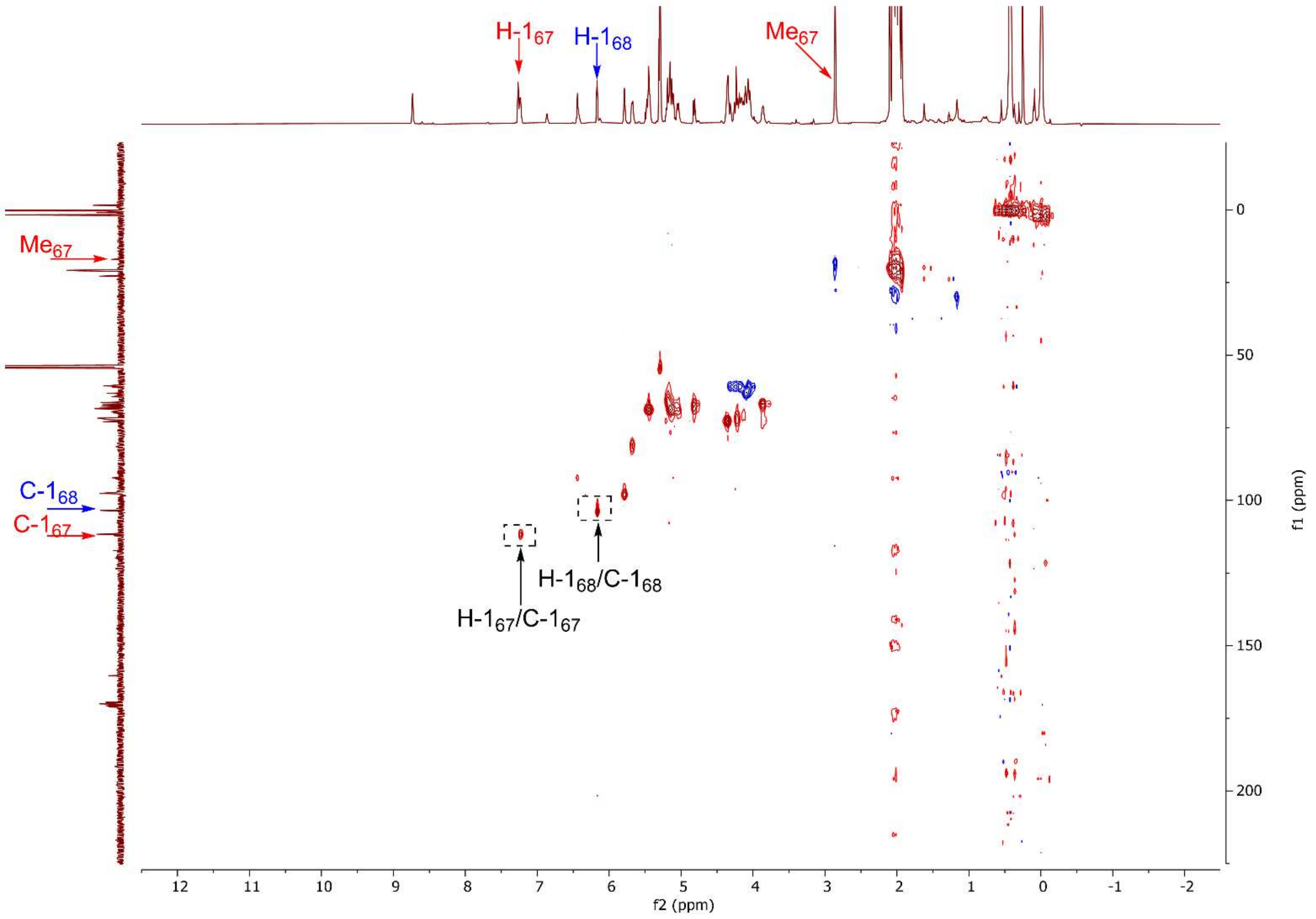

HMBC (CD_2Cl_2) spectrum of reaction mixture at -40 °C from VT NMR experiment with glucosyl trichloroacetimidate 57:

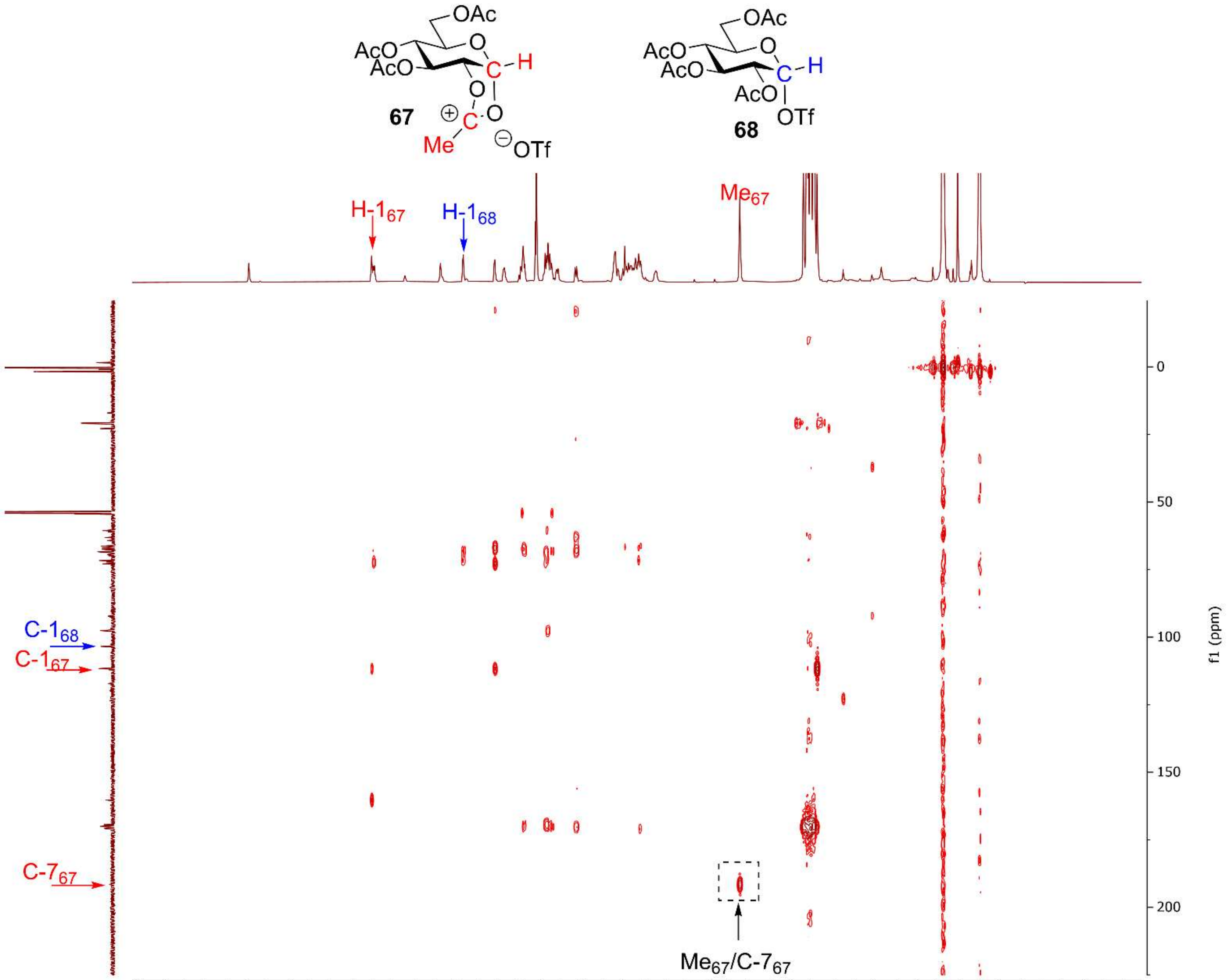

^{19}F NMR (470 MHz, CD_2Cl_2) spectrum of reaction mixture at -40 °C from VT NMR experiment with glucosyl trichloroacetimidate 57:

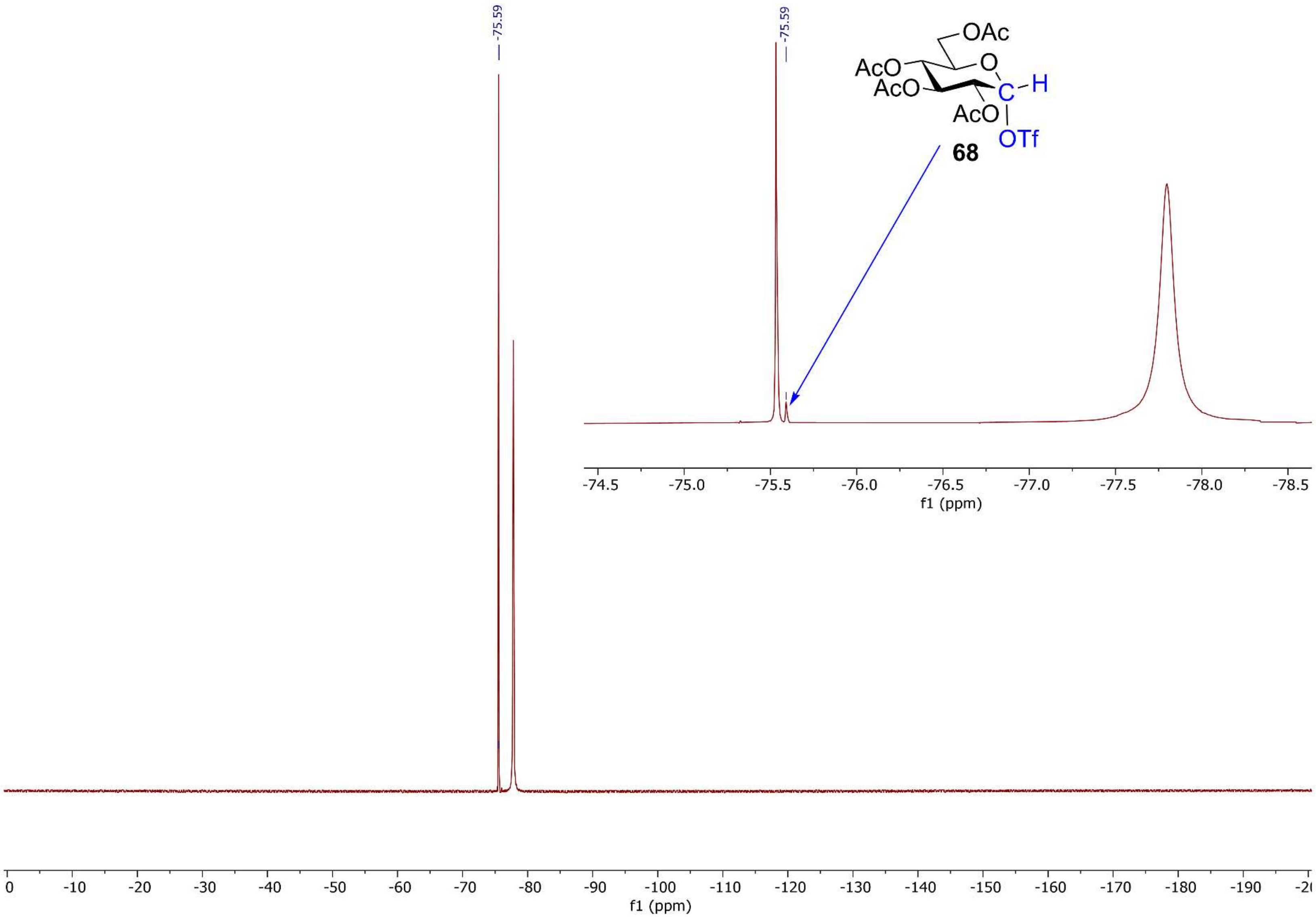

Stacked ^{1}H NMR (500 MHz, CD_2Cl_2) spectra from VT NMR experiment with 5-thioglucosyl trichloroacetimidate 1:

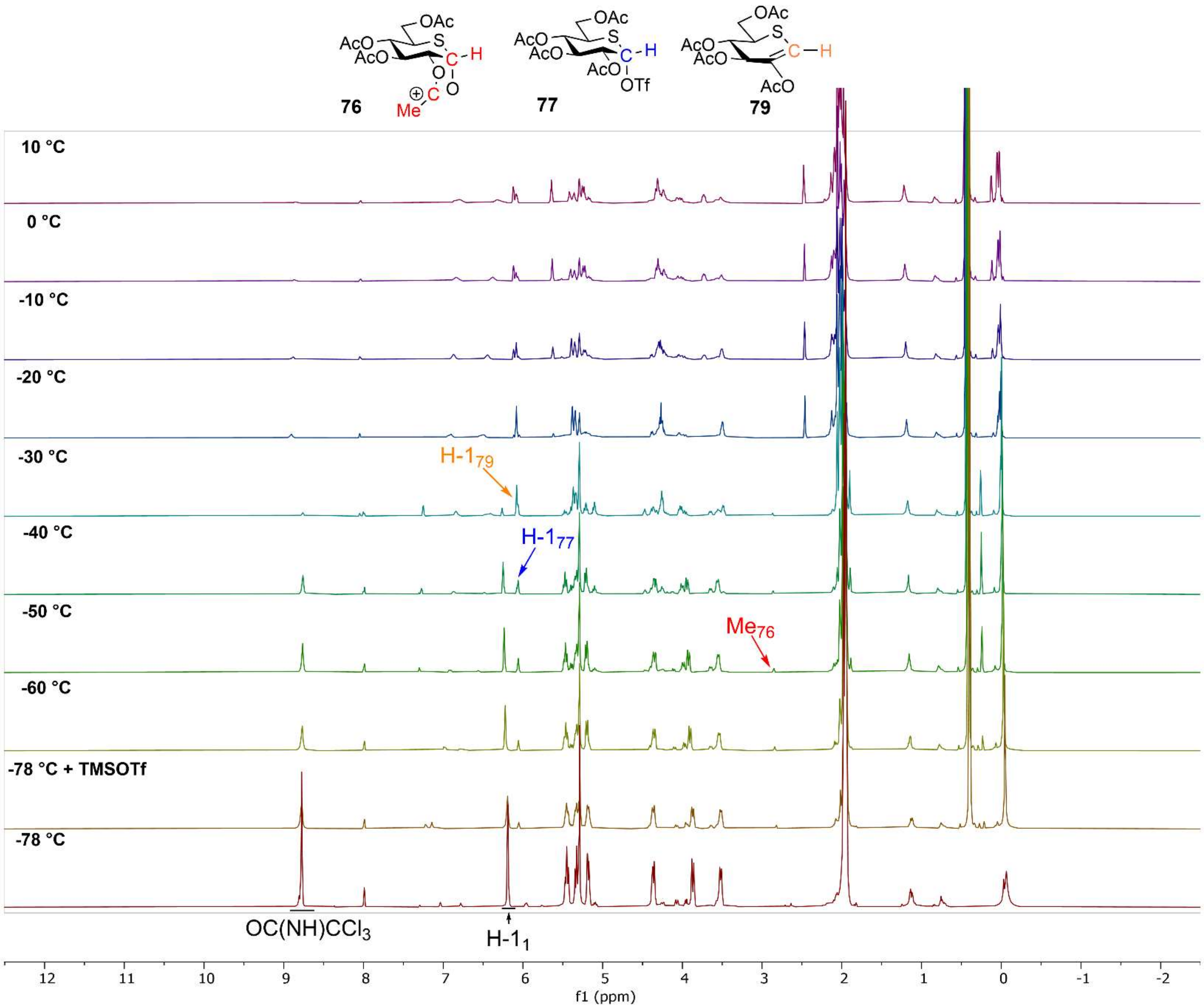

Stacked ^{19}F NMR (470 MHz, CD_2Cl_2) spectra from VT NMR experiment with 5-thioglucosyl trichloroacetimidate 1:

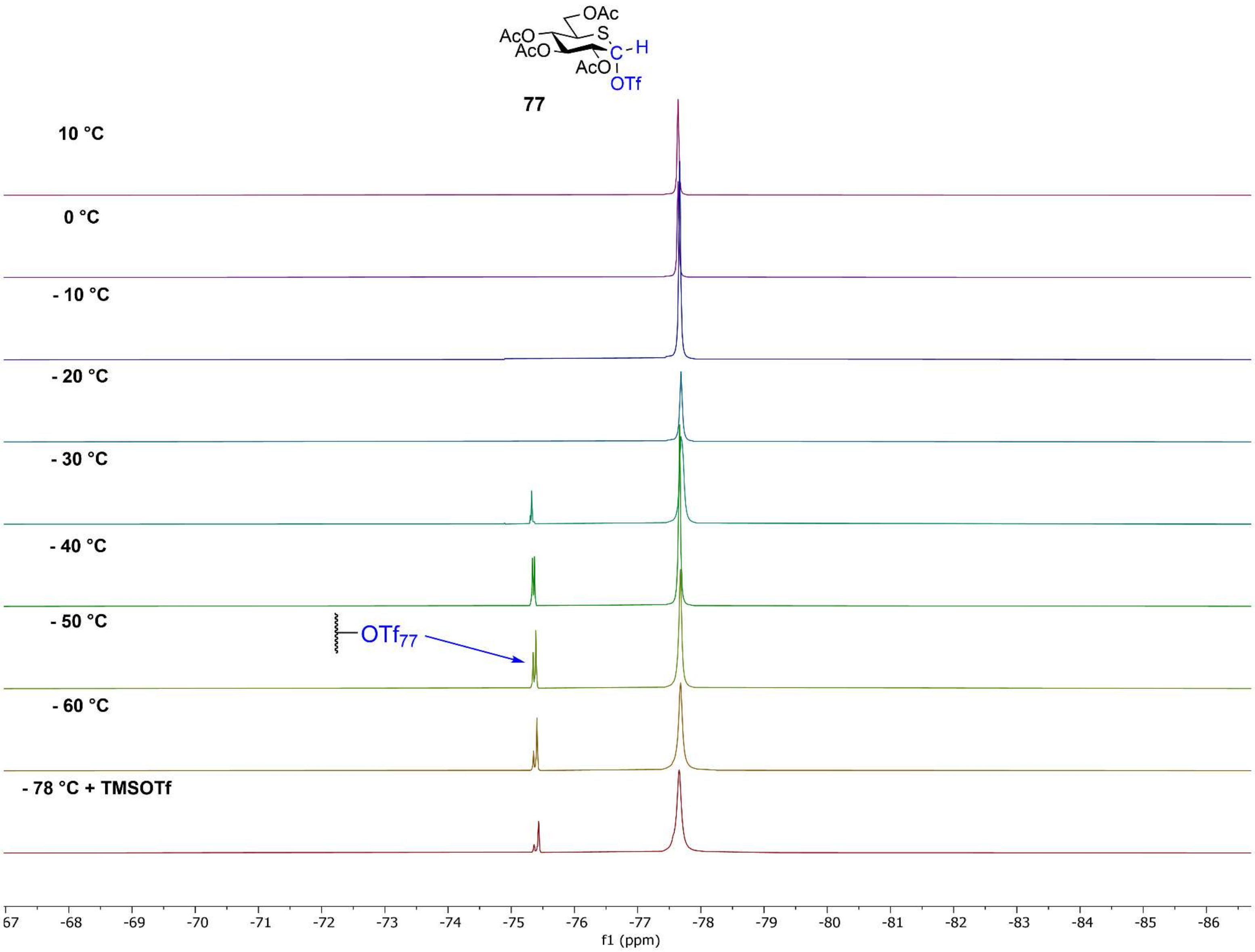

^{1}H NMR (500 MHz, CD_2Cl_2) spectrum of reaction mixture at -40 °C from VT NMR experiment with 5-thioglucosyl trichloroacetimidate 1:

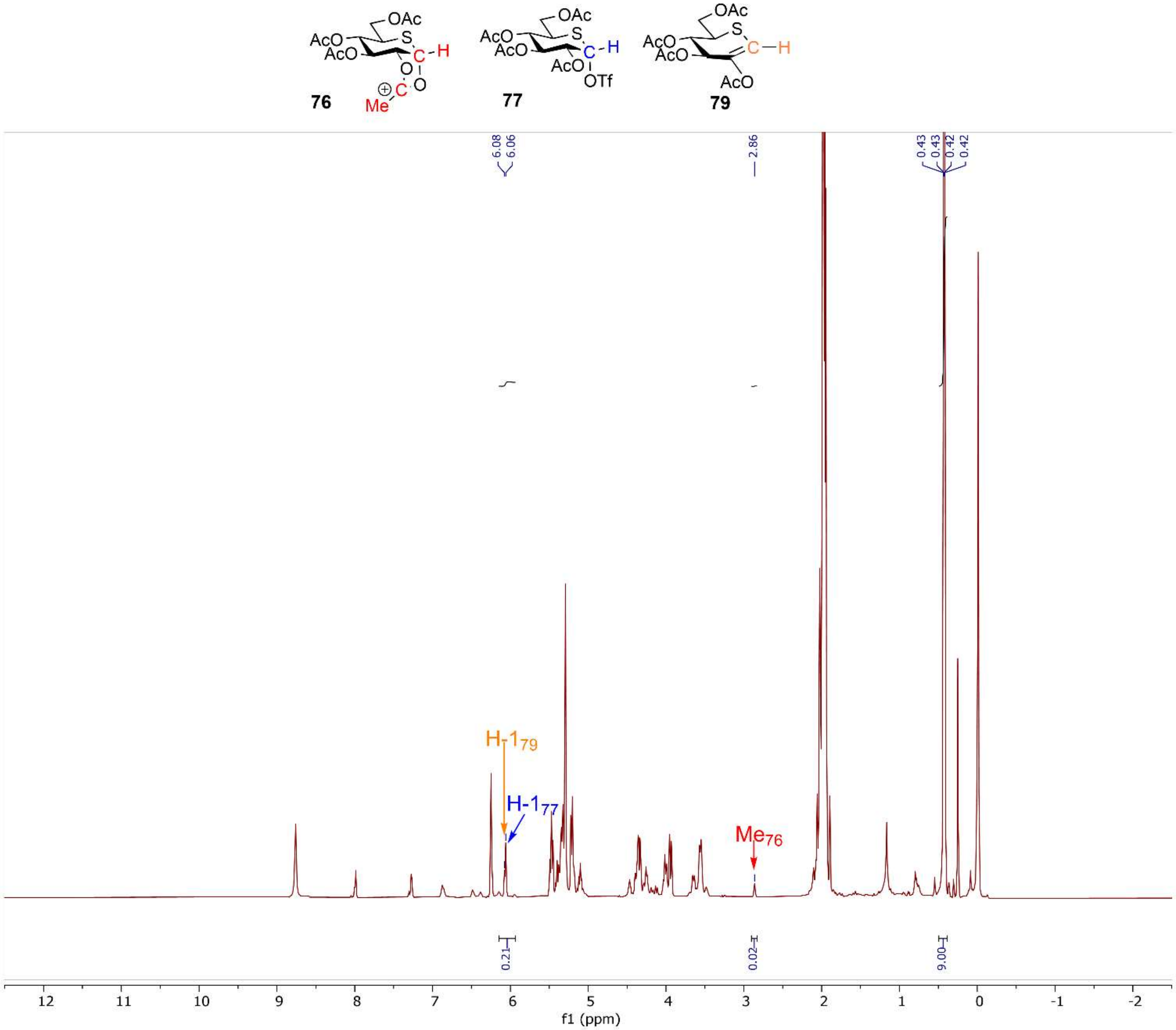

^{13}C NMR (125.67 MHz, CD_2Cl_2) spectrum of reaction mixture at -40 °C from VT NMR experiment with 5-thioglucosyl trichloroacetimidate 1:

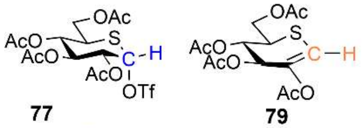

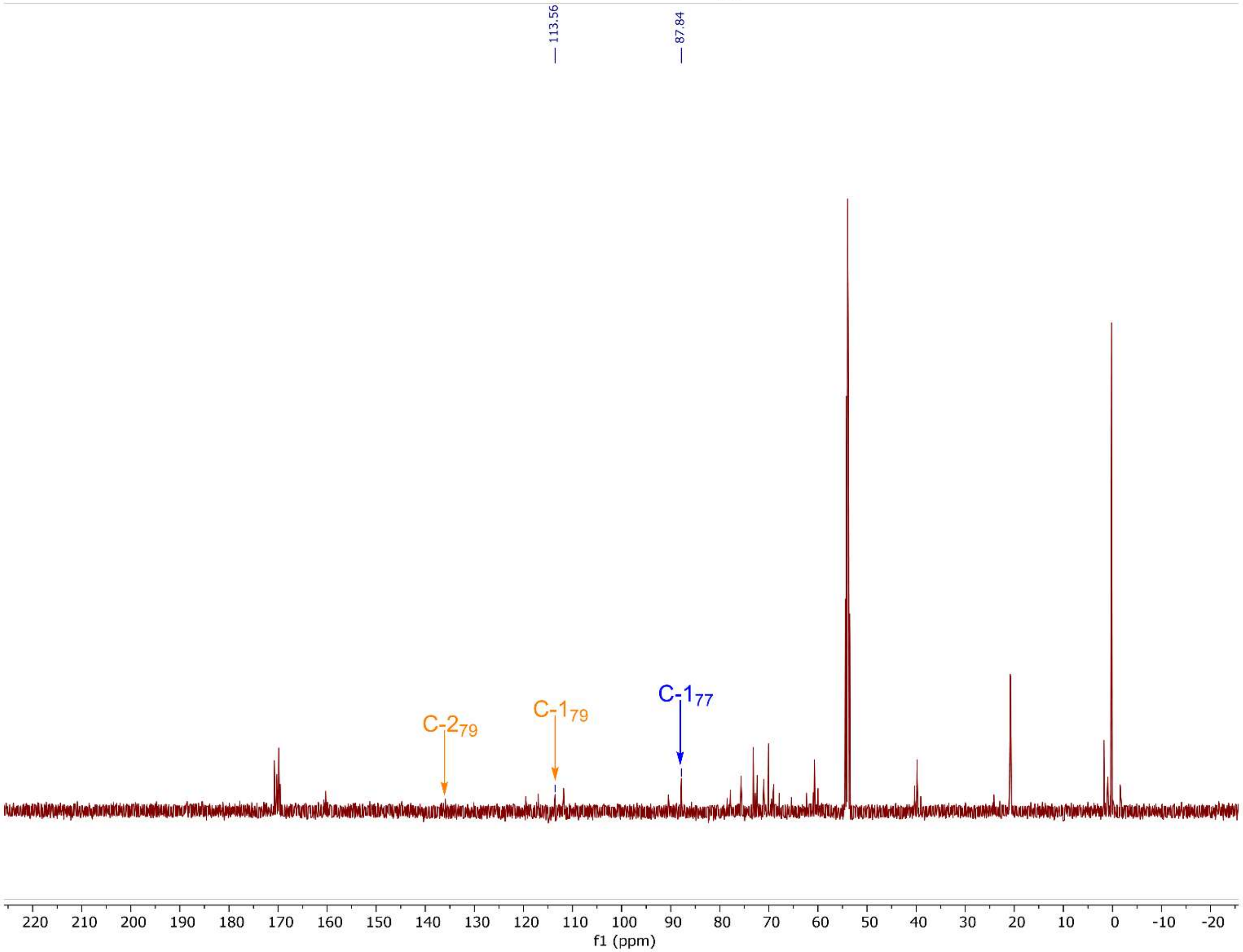

DEPT-135 (CD_2Cl_2) spectrum of reaction mixture at -40 °C from VT NMR experiment with 5-thioglucosyl trichloroacetimidate 1:

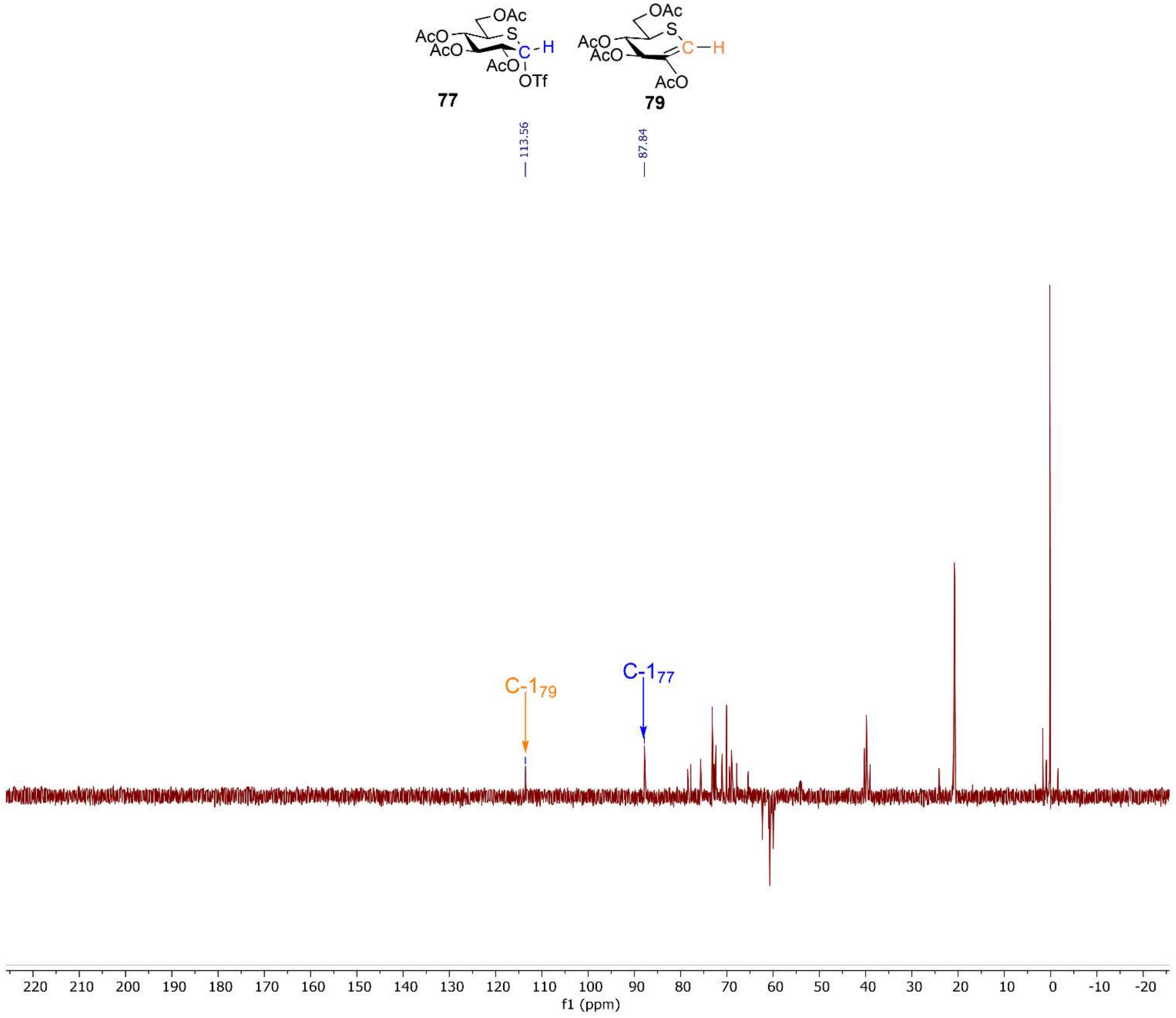

HSQC (CD_2Cl_2) spectrum of reaction mixture at -40 °C from VT NMR experiment with 5-thioglucosyl trichloroacetimidate 1:

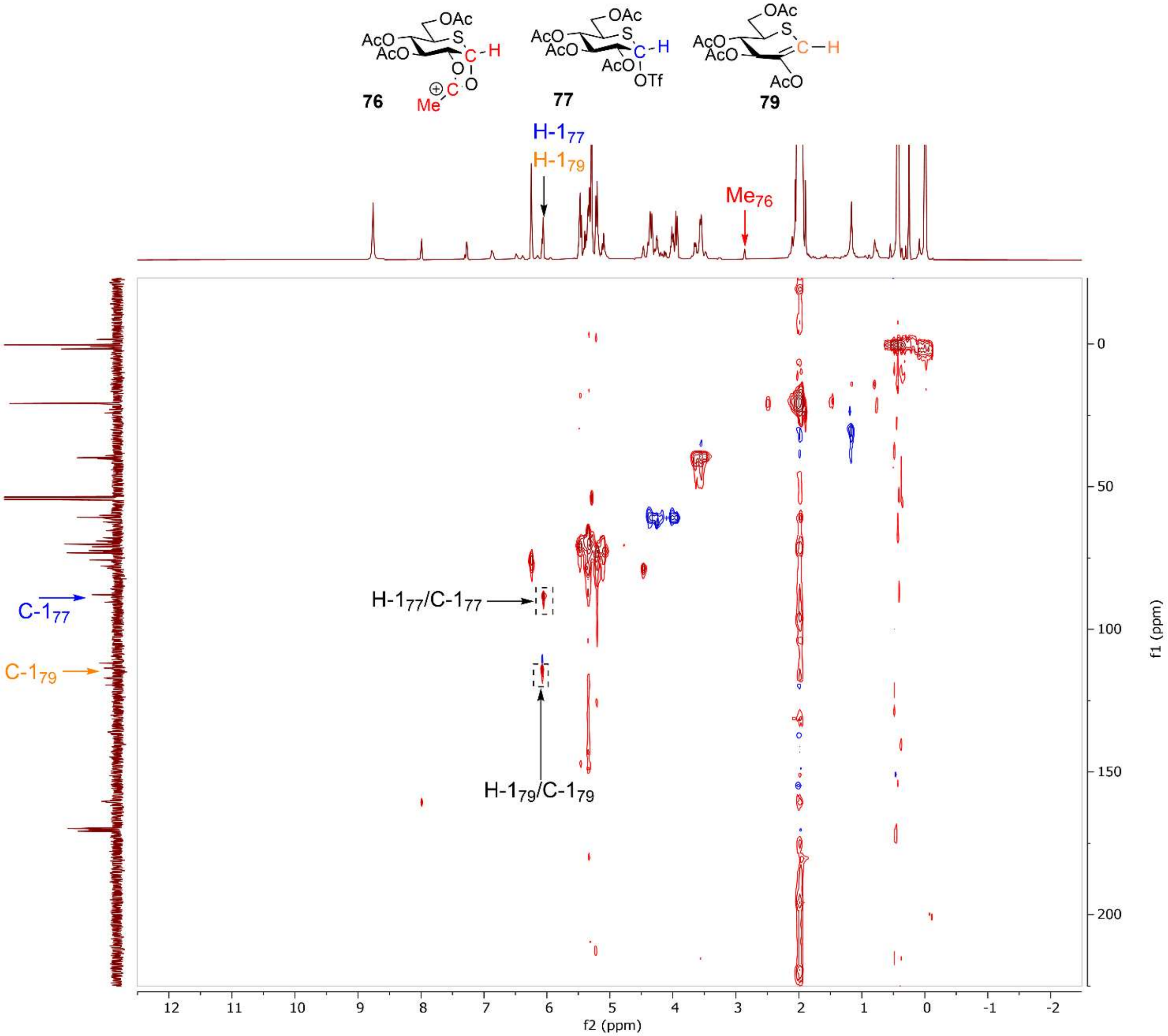

HMBC (CD_2Cl_2) spectrum of reaction mixture at -40 °C from VT NMR experiment with 5-thioglucosyl trichloroacetimidate 1:

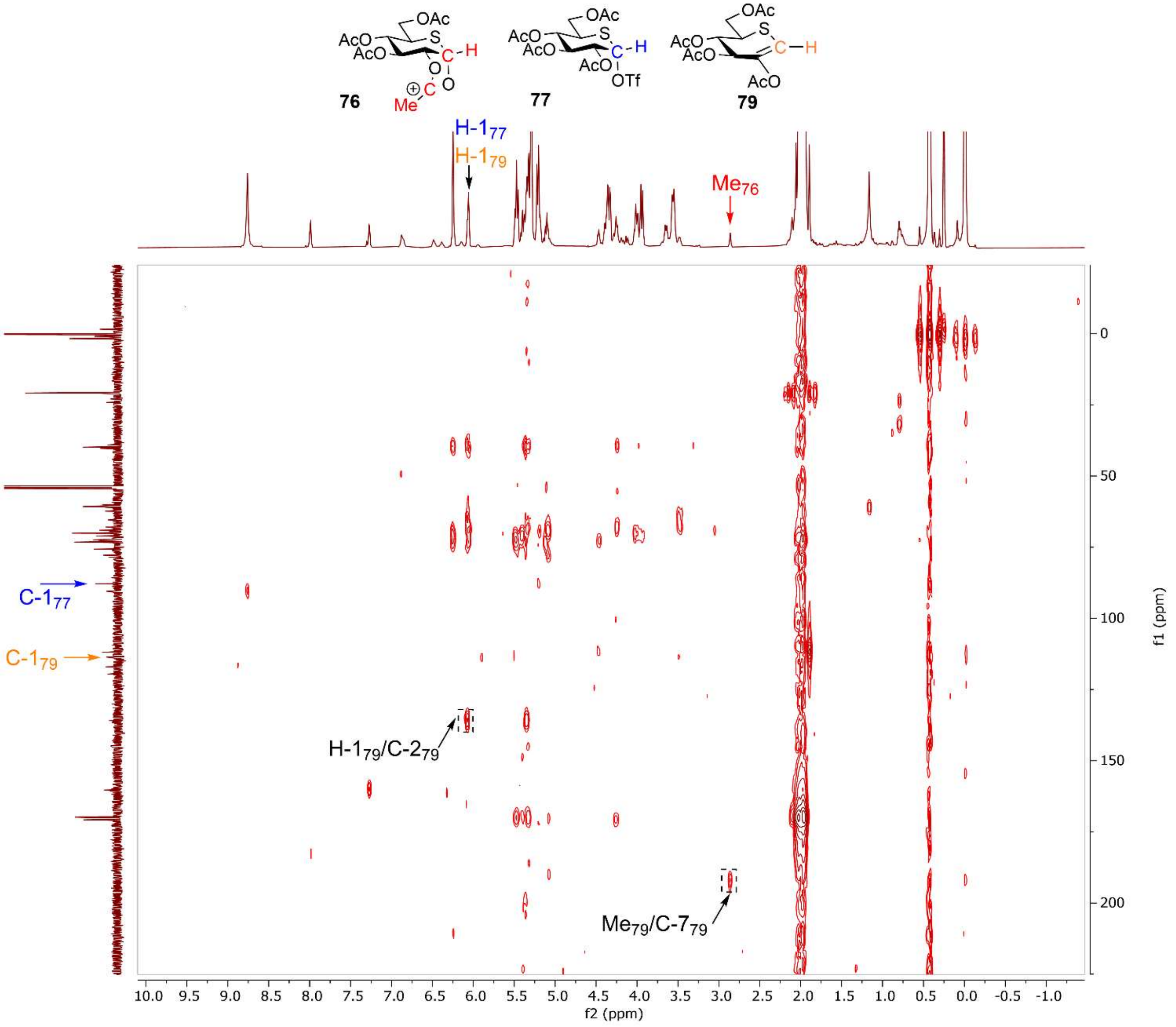

^{19}F NMR (470 MHz, CD_2Cl_2) spectrum of reaction mixture at -40 °C from VT NMR experiment with 5-thioglucosyl trichloroacetimidate 1:

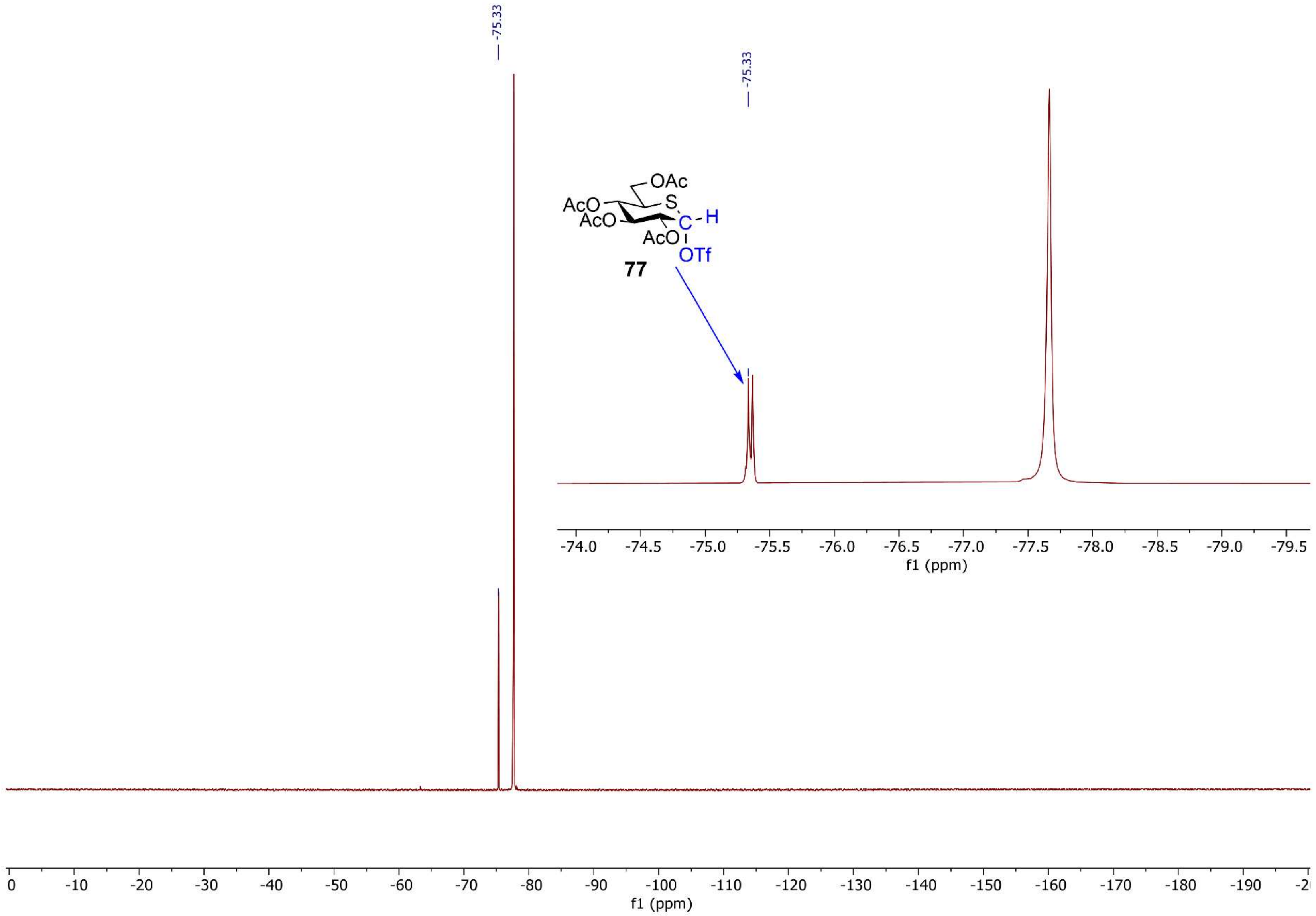

3.10. VT NMR spectra from experiments with permethylated glucosyl sulfoxides (53, 55):

Stacked ^{1}H NMR (500 MHz, CD_2Cl_2) spectra from VT NMR experiment with glucosyl sulfoxides 53:

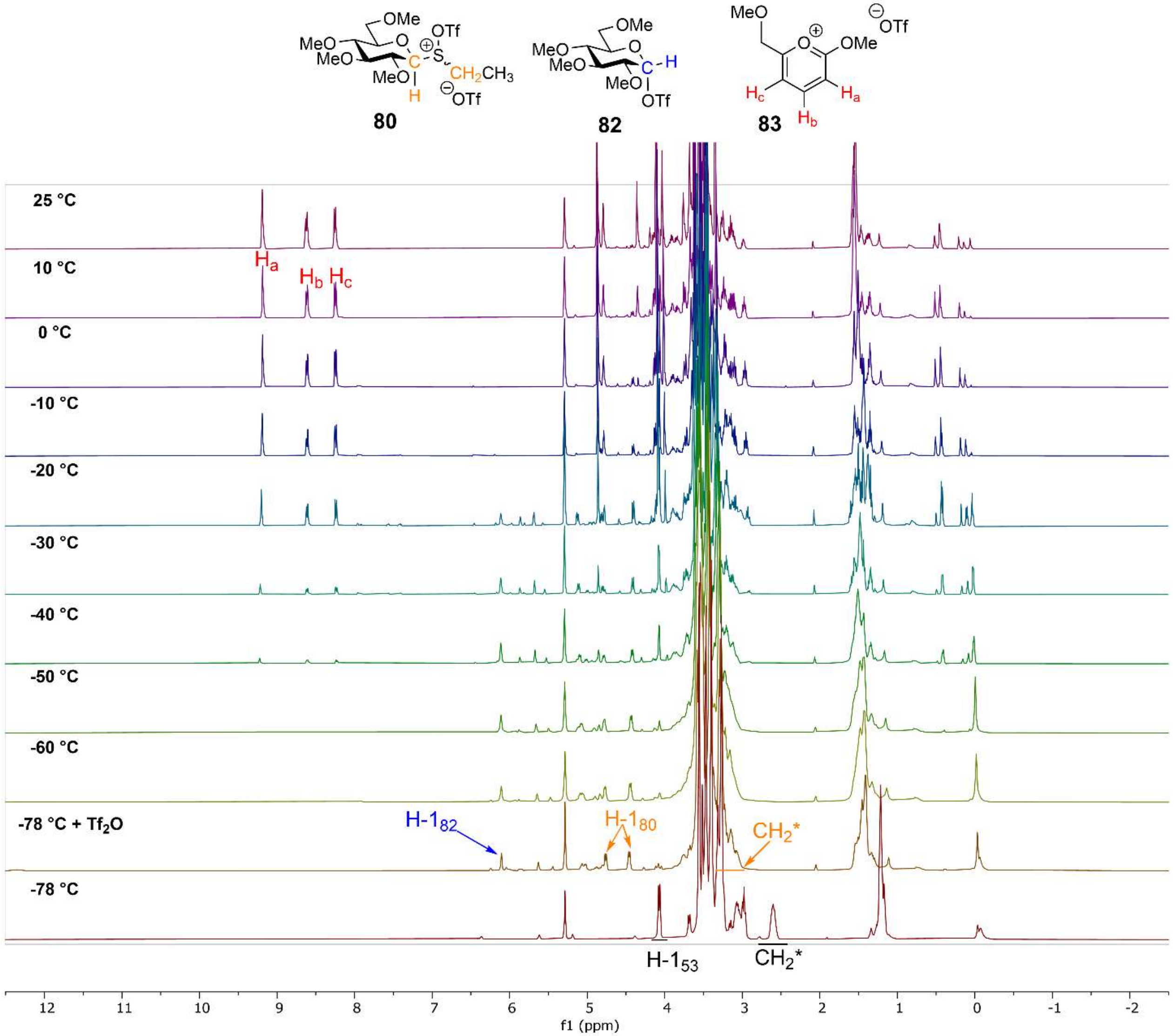

Stacked ^{19}F NMR (470 MHz, CD_2Cl_2) spectra from VT NMR experiment with glucosyl sulfoxides 53:

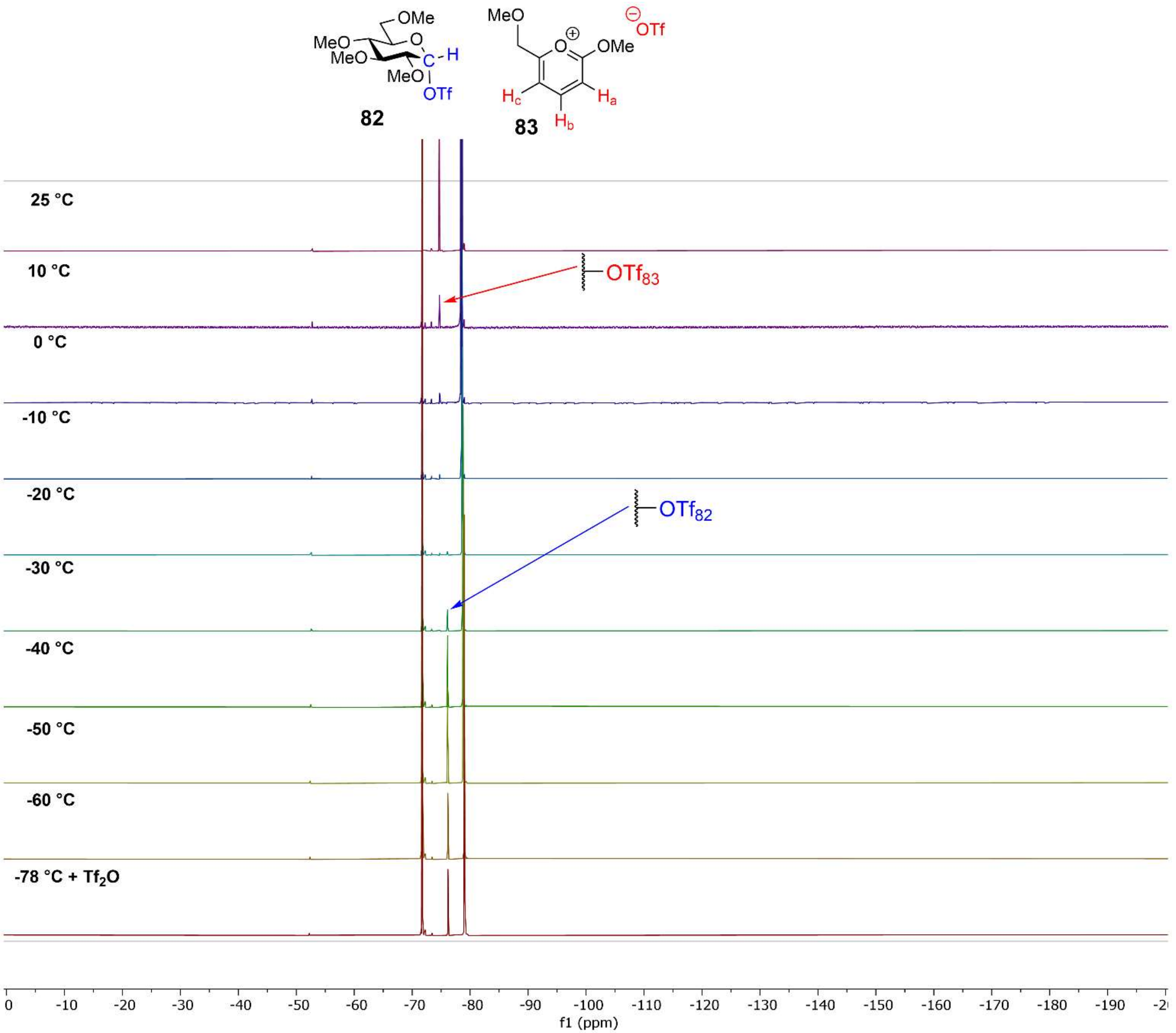

^1H NMR (500 MHz, CD_2Cl_2) spectrum of reaction mixture at -50 °C from VT NMR experiment with glucosyl sulfoxide 53:

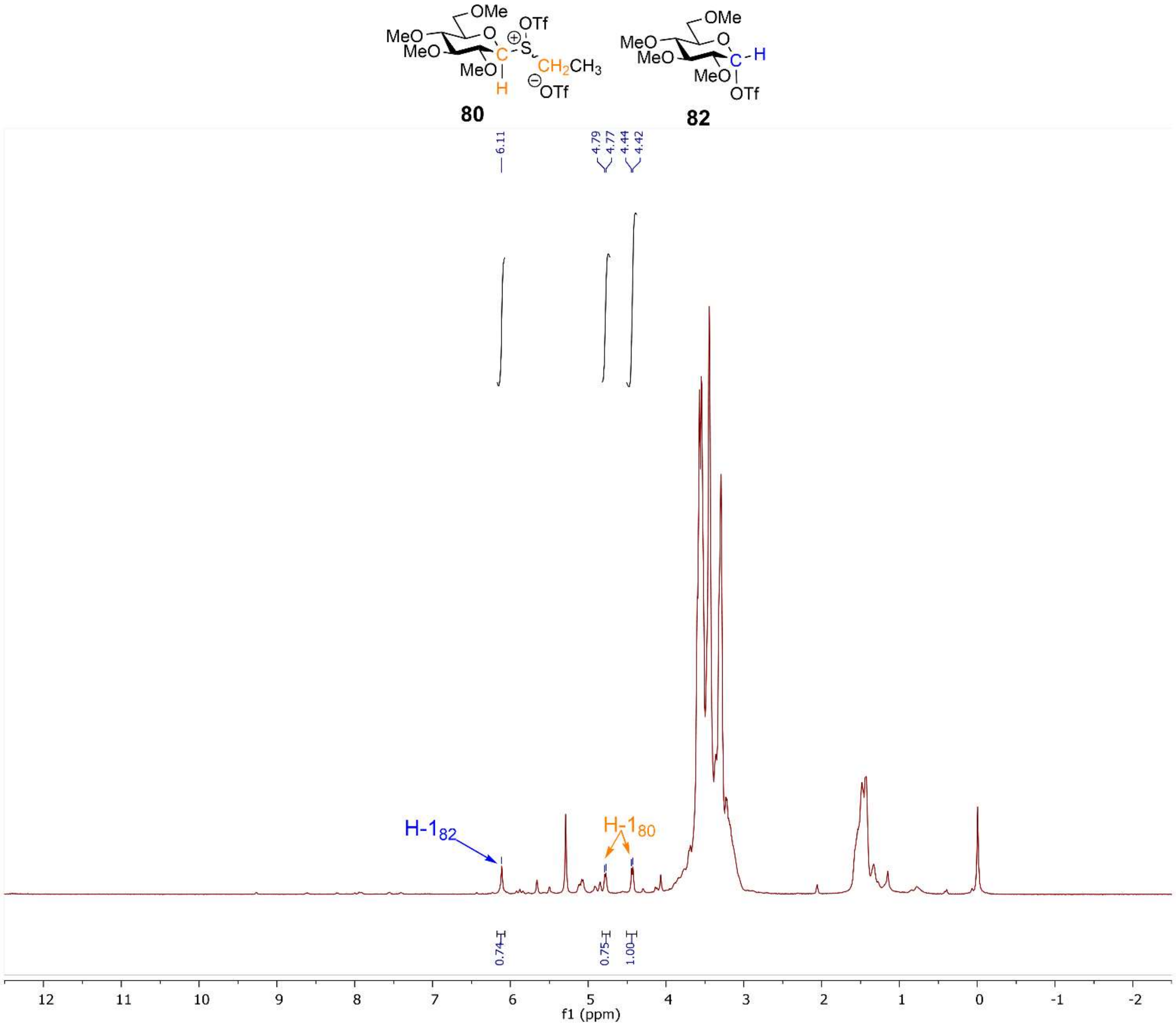

^{13}C NMR (125.67 MHz, CD_2Cl_2) spectrum of reaction mixture at -50 °C from VT NMR experiment with glucosyl sulfoxide 53:

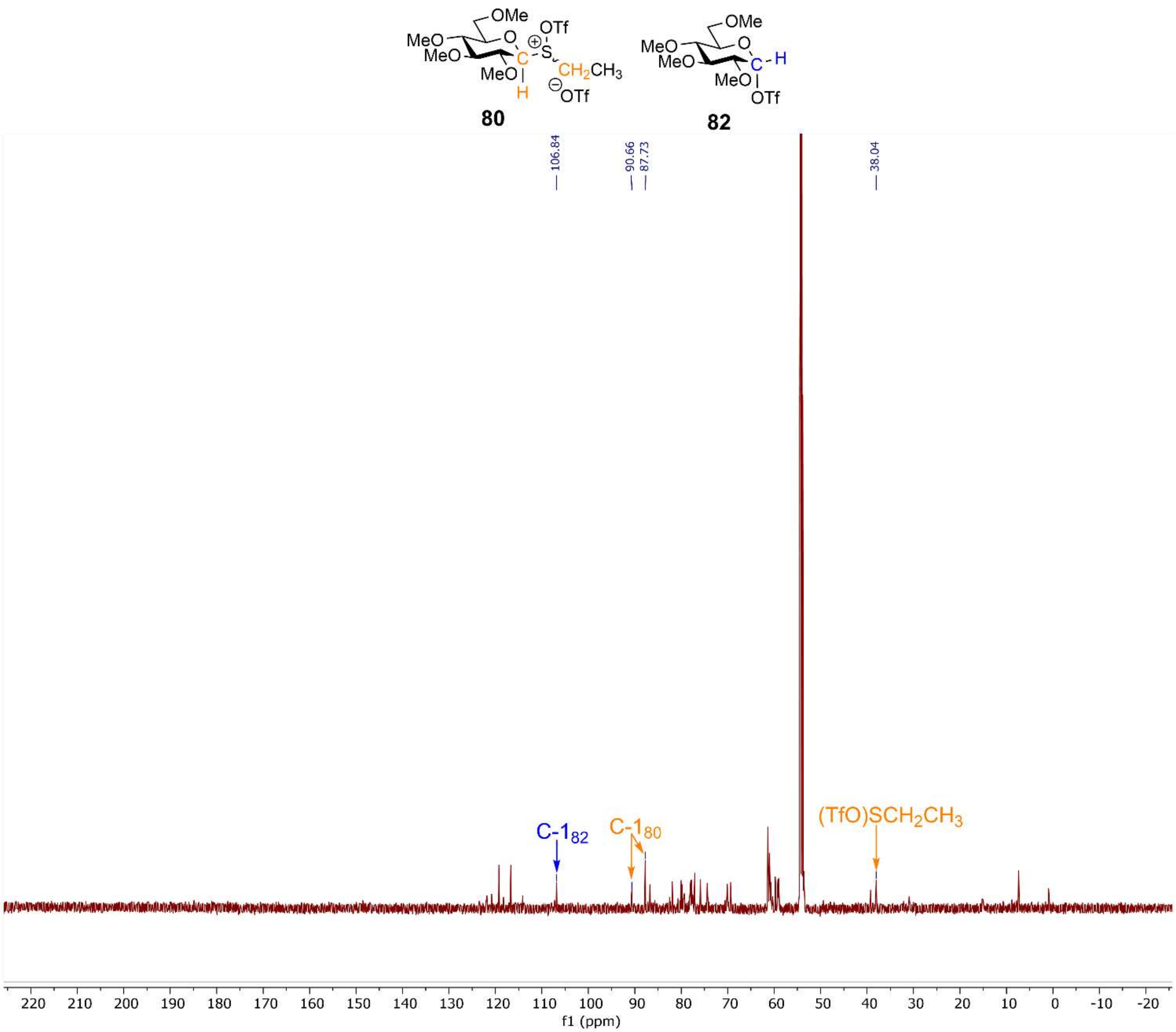

DEPT-135 (CD_2Cl_2) spectrum of reaction mixture at -50 °C from VT NMR experiment with glucosyl sulfoxide 53:

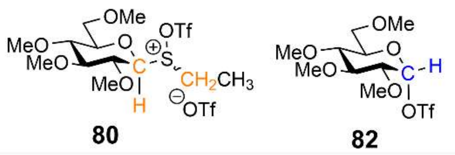

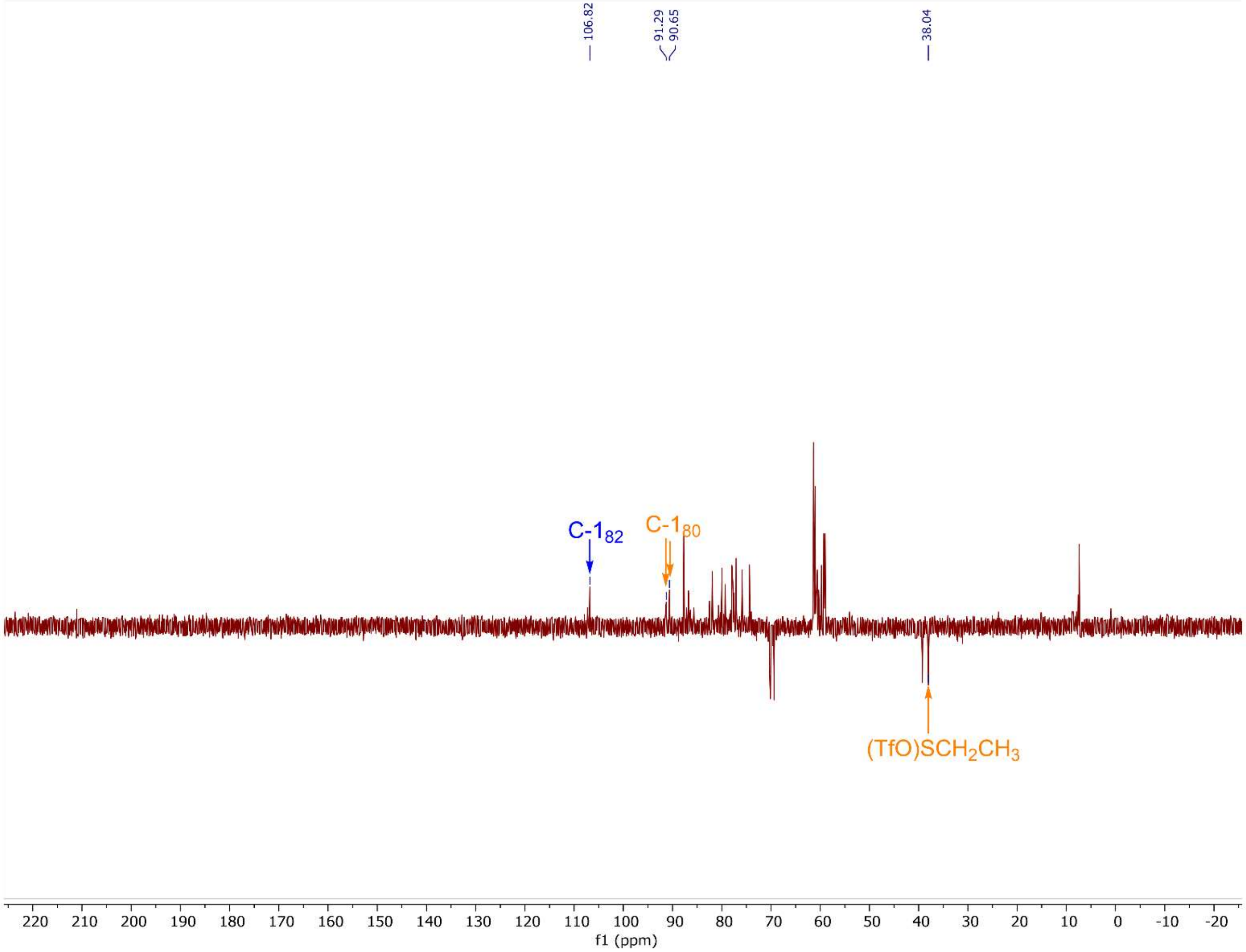

HSQC (CD_2Cl_2) spectrum of reaction mixture at -50 °C from VT NMR experiment with glucosyl sulfoxide 53:

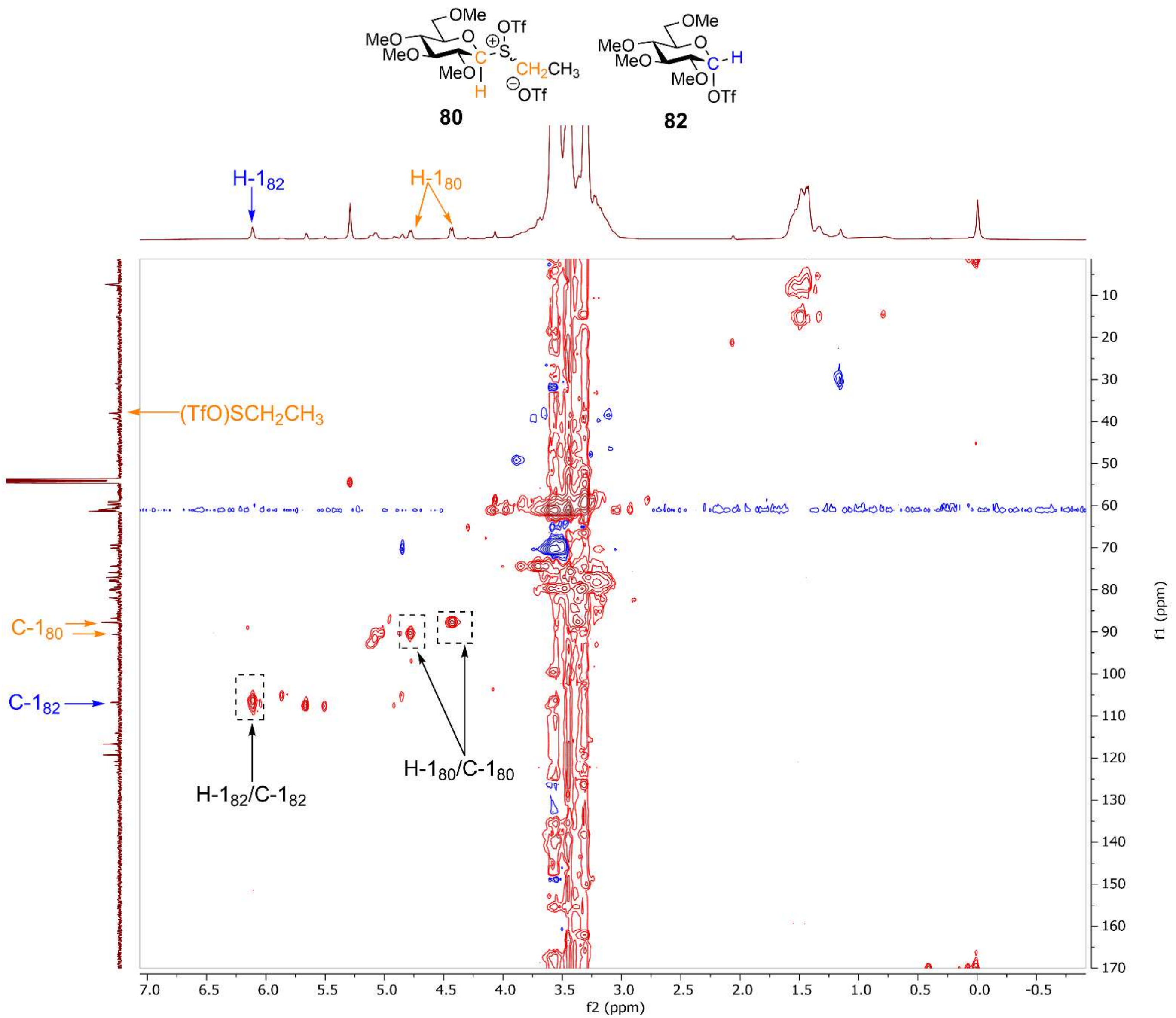

HMBC (CD_2Cl_2) spectrum of reaction mixture at -50 °C from VT NMR experiment with glucosyl sulfoxide 53:

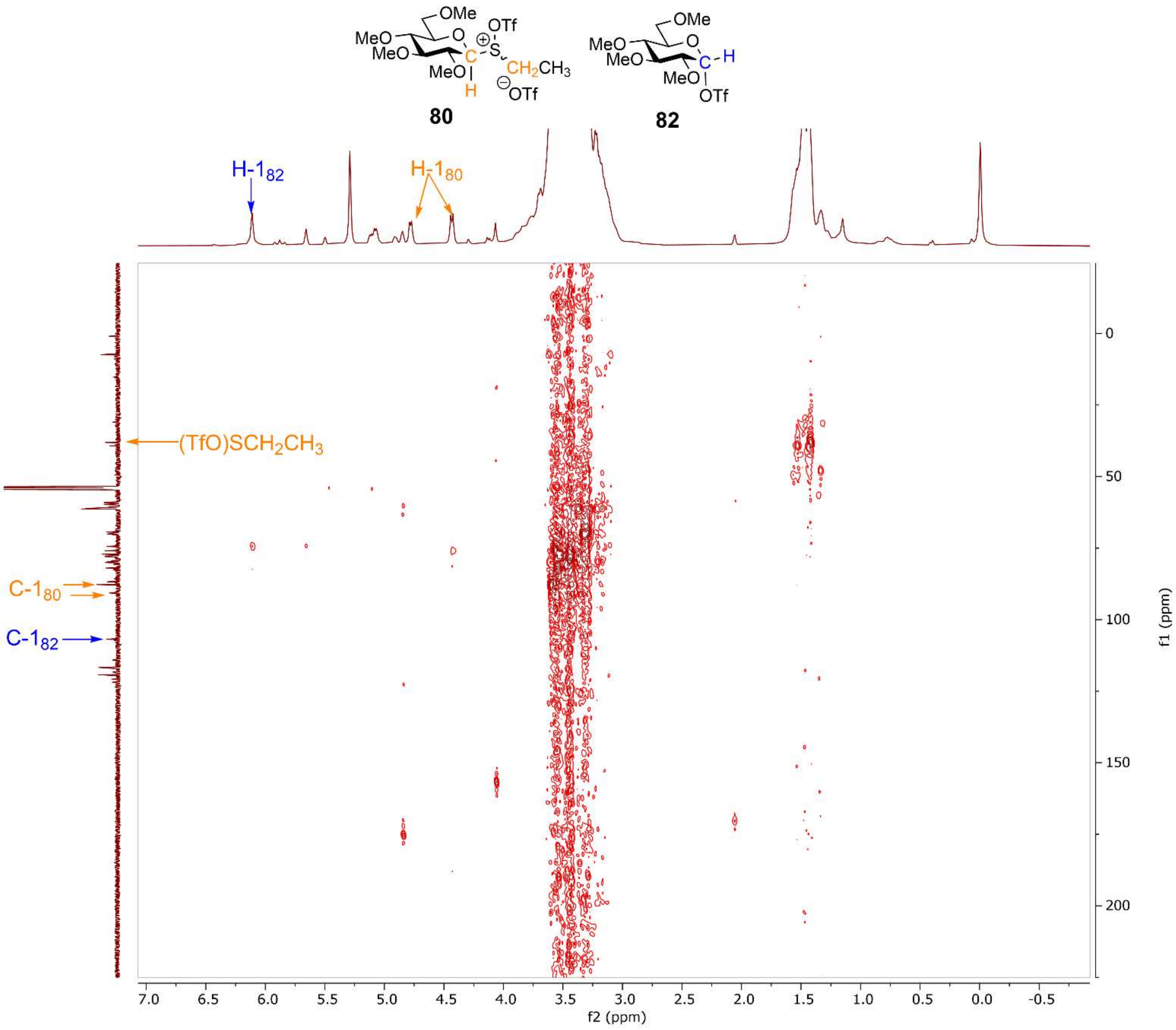

^{19}F NMR (470 MHz, CD_2Cl_2) spectrum of reaction mixture at -50 °C from VT NMR experiment with glucosyl sulfoxide 53:

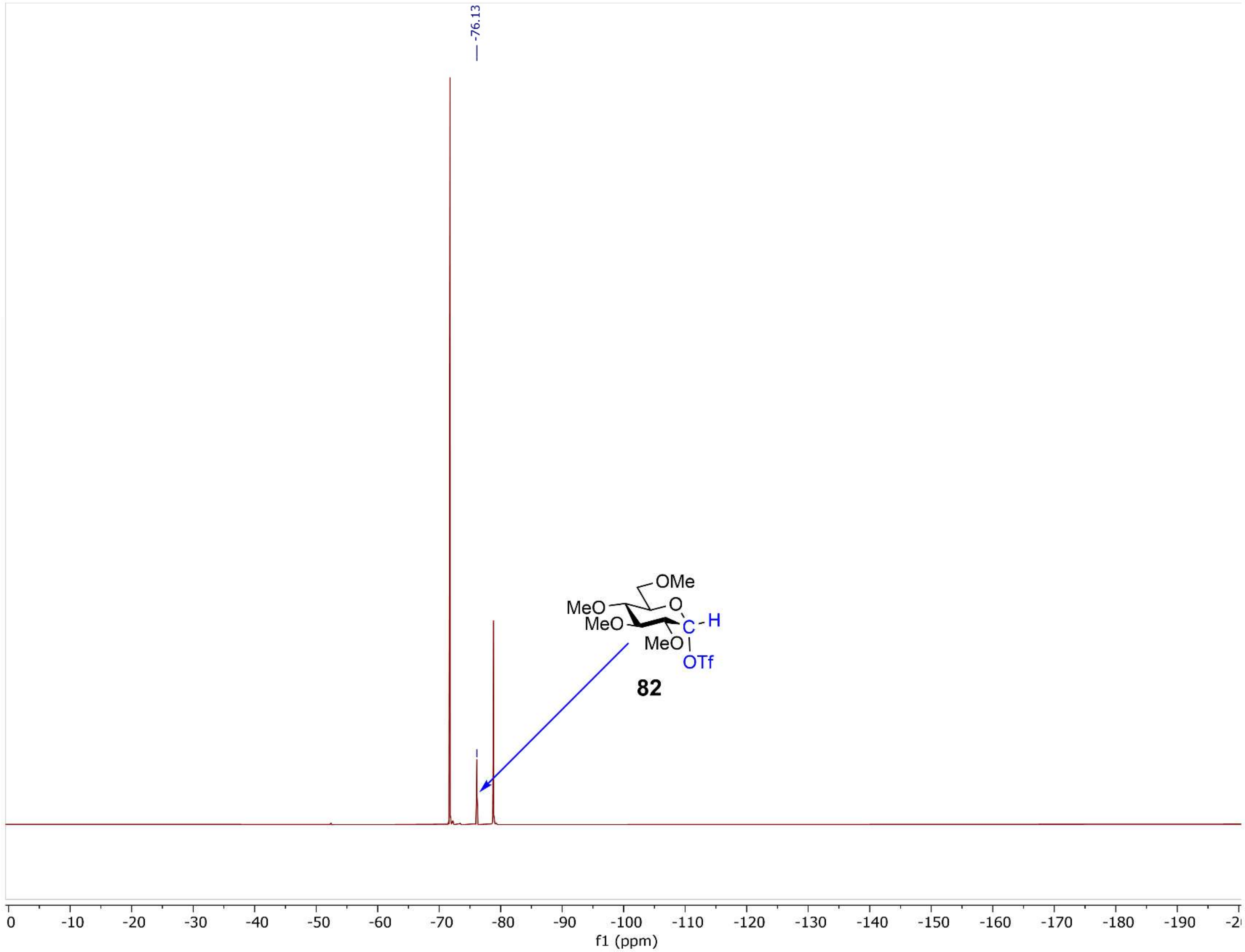

^{1}H NMR (500 MHz, CD_2Cl_2) spectrum of reaction mixture at 25 °C from VT NMR experiment with glucosyl sulfoxide 53:

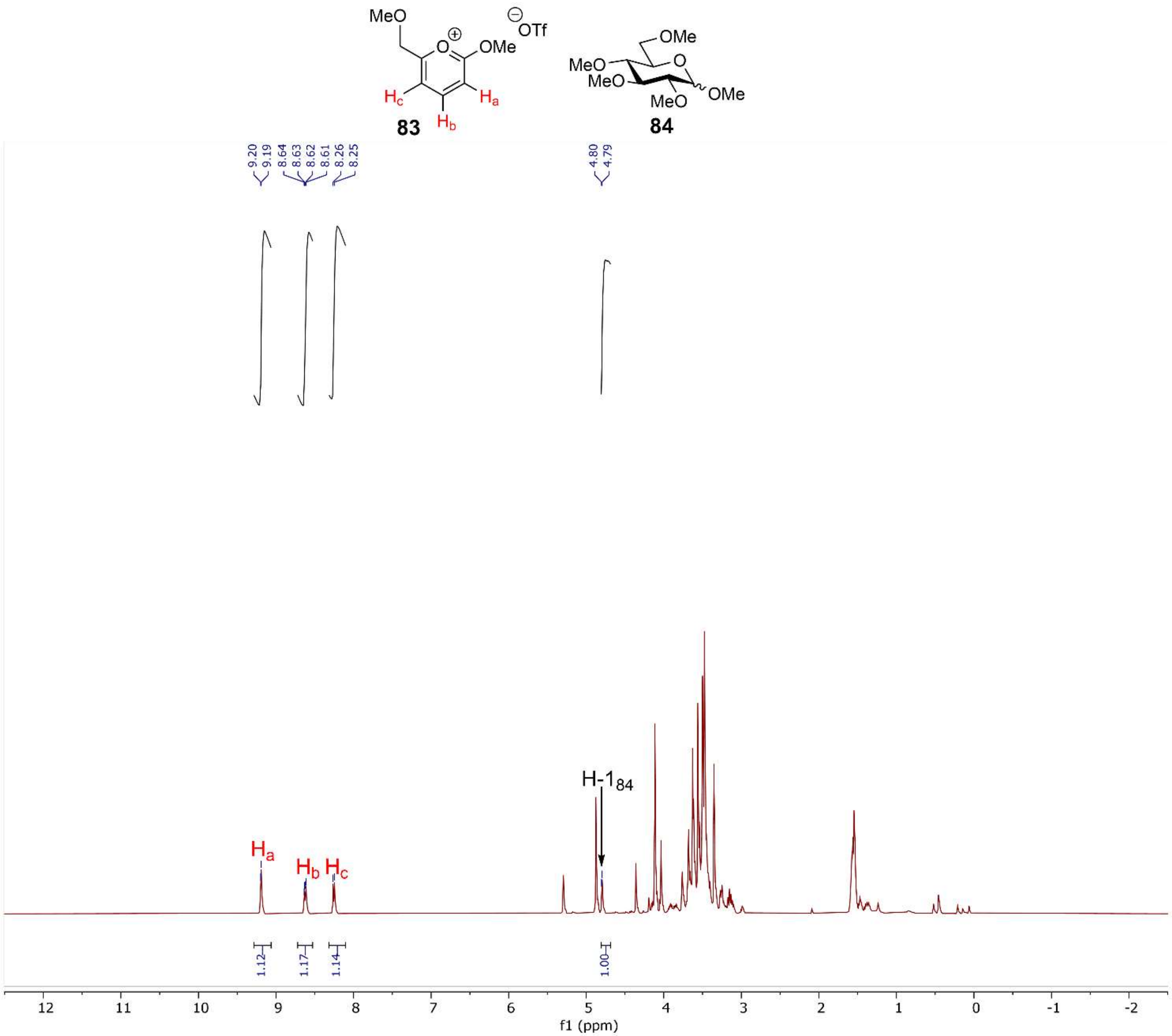

^{13}C NMR (125.67 MHz, CD_2Cl_2) spectrum of reaction mixture at 25 °C from VT NMR experiment with glucosyl sulfoxide 53:

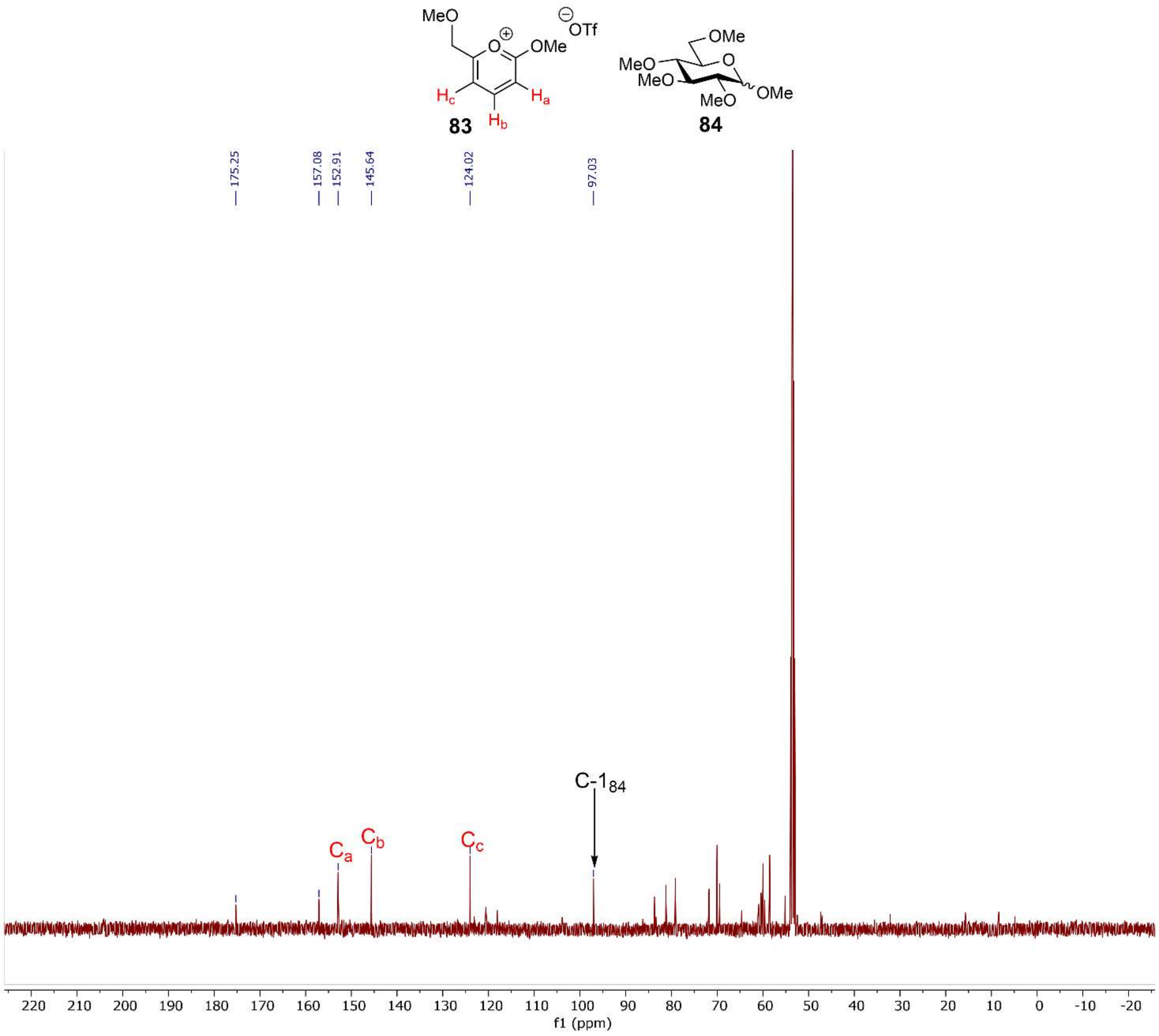

DEPT-135 (CD_2Cl_2) spectrum of reaction mixture at 25 °C from VT NMR experiment with glucosyl sulfoxide 53:

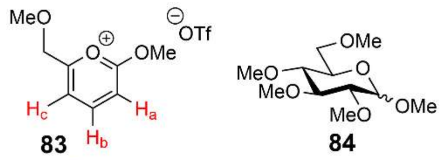

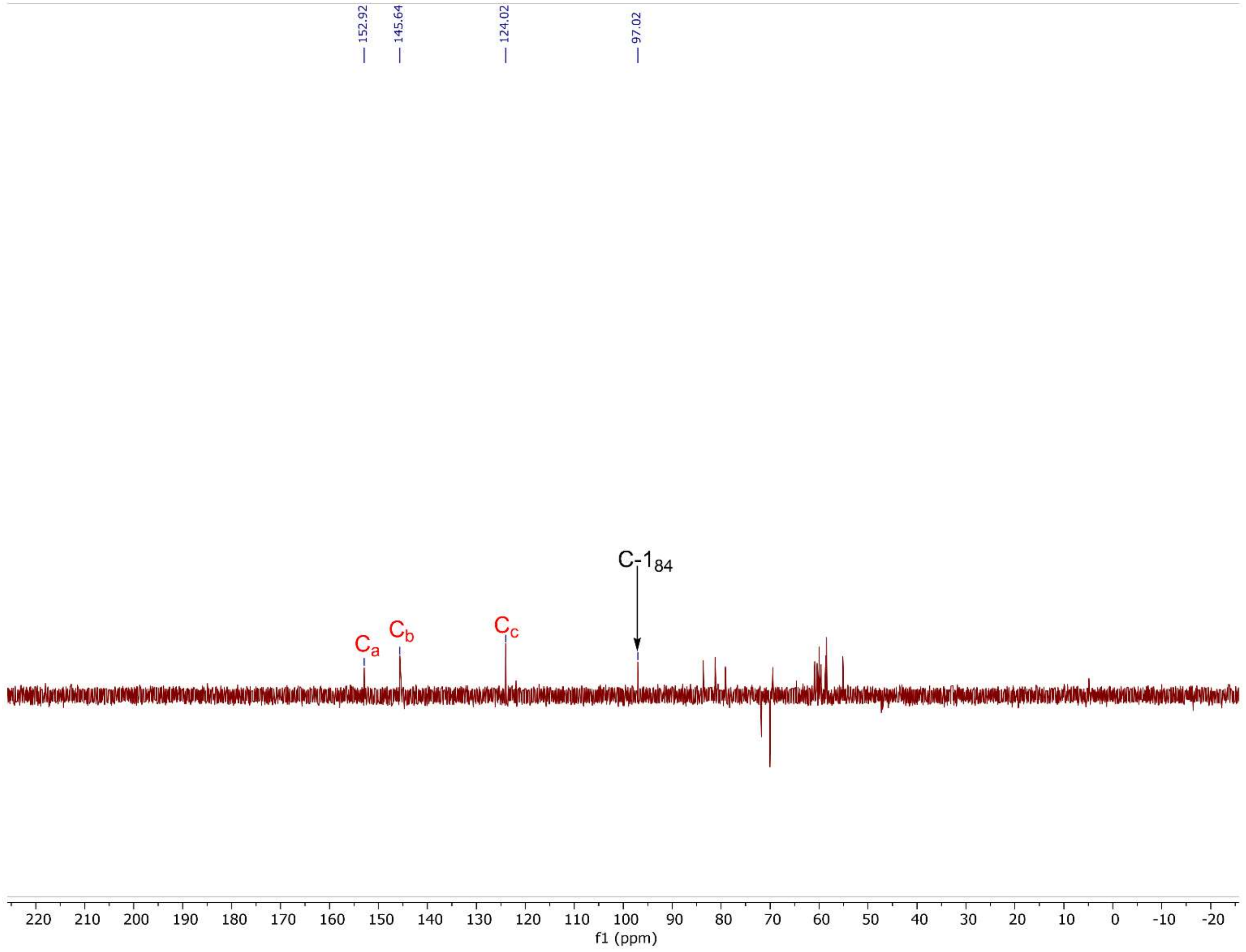

COSY (CD_2Cl_2) spectrum of reaction mixture at 25 °C from VT NMR experiment with glucosyl sulfoxide 53:

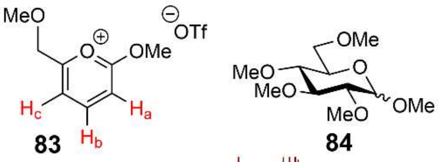

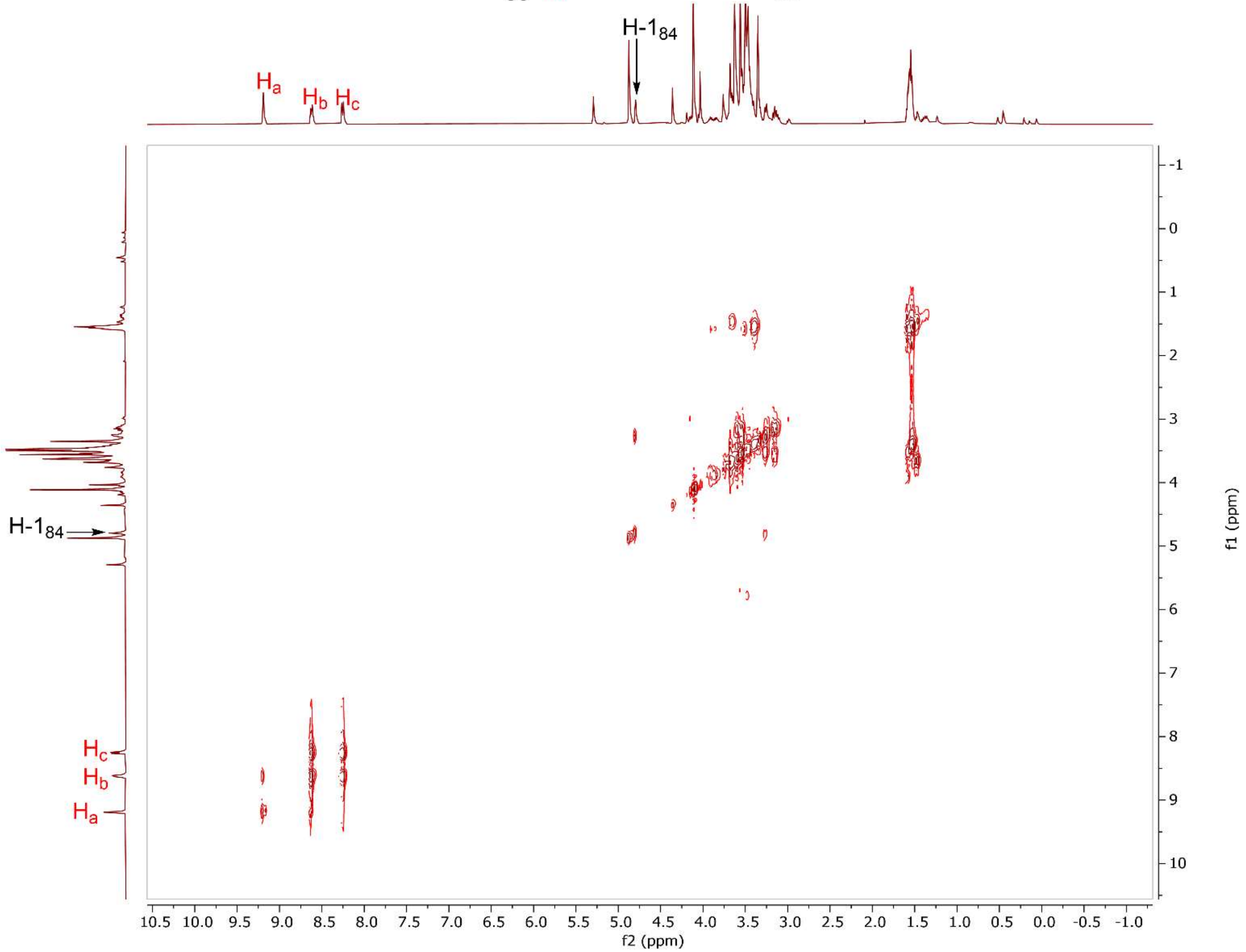

HSQC (CD_2Cl_2) spectrum of reaction mixture at 25 °C from VT NMR experiment with glucosyl sulfoxide 53:

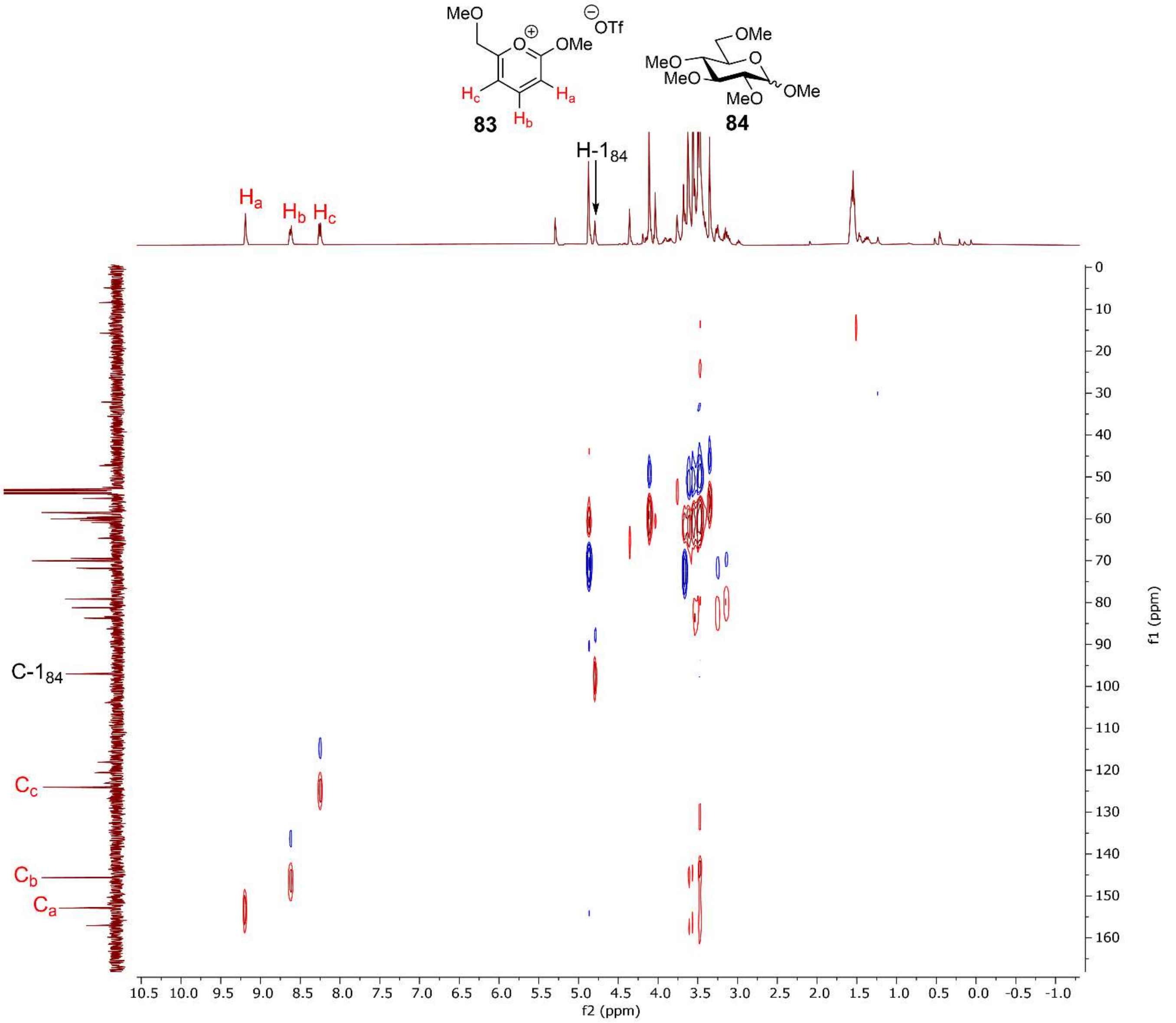

^{19}F NMR (470 MHz, CD_2Cl_2) spectrum of reaction mixture at 25 °C from VT NMR experiment with glucosyl sulfoxide 53:

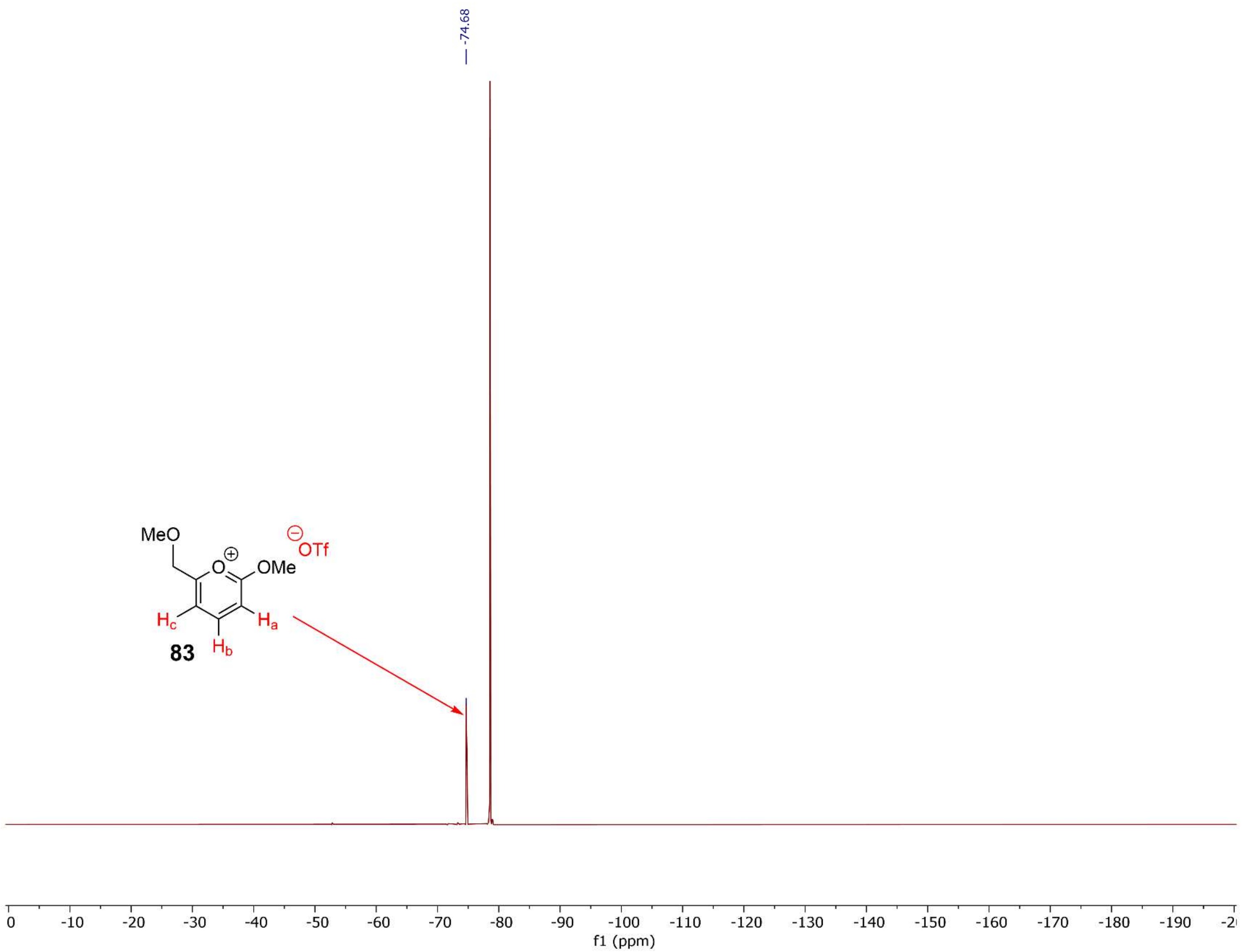

Stacked ^{1}H NMR (500 MHz, CD_2Cl_2) spectra from VT NMR experiment with glucosyl sulfoxides 55:

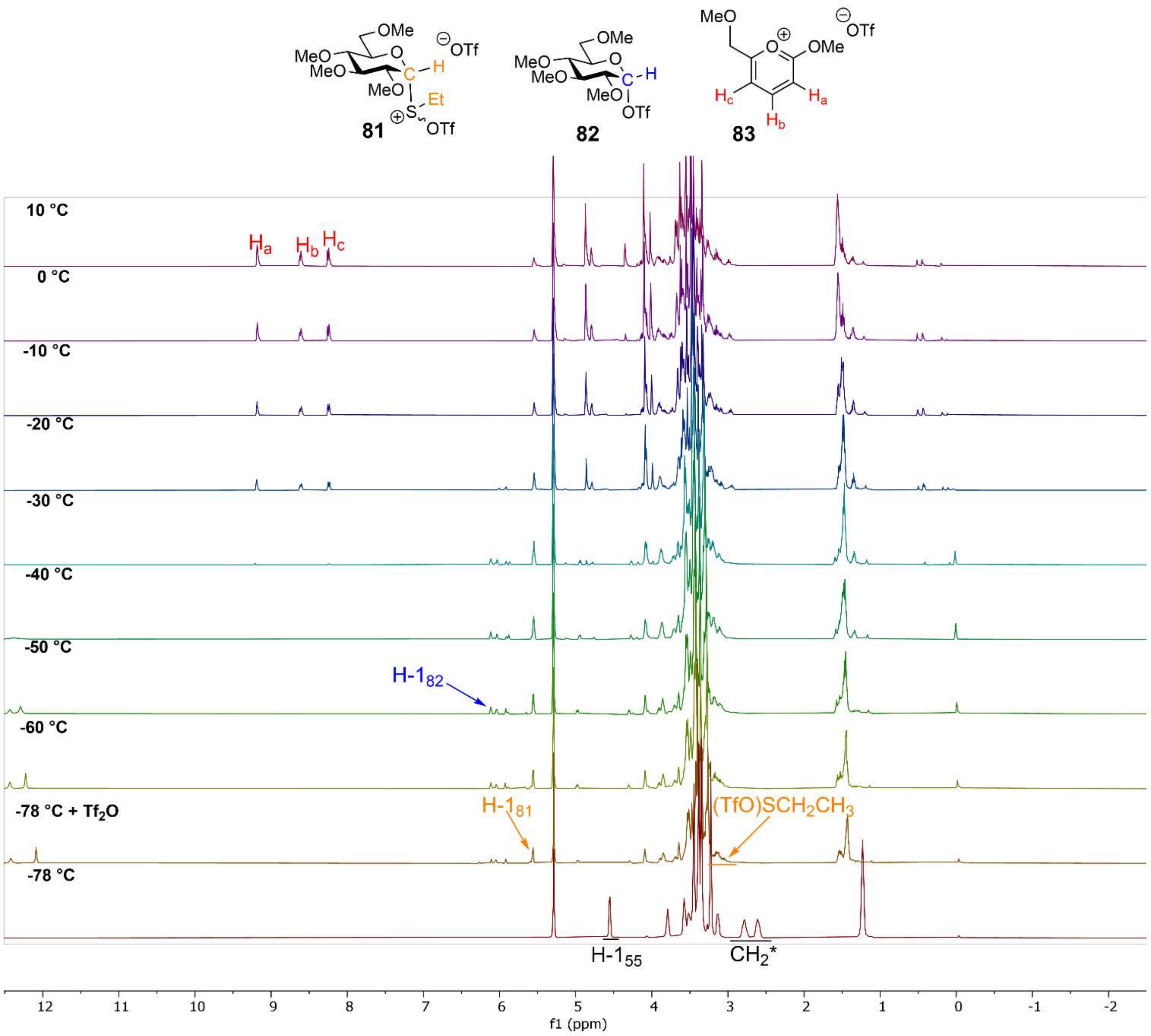

Stacked ^{19}F NMR (470 MHz, CD_2Cl_2) spectra from VT NMR experiment with glucosyl sulfoxides 53:

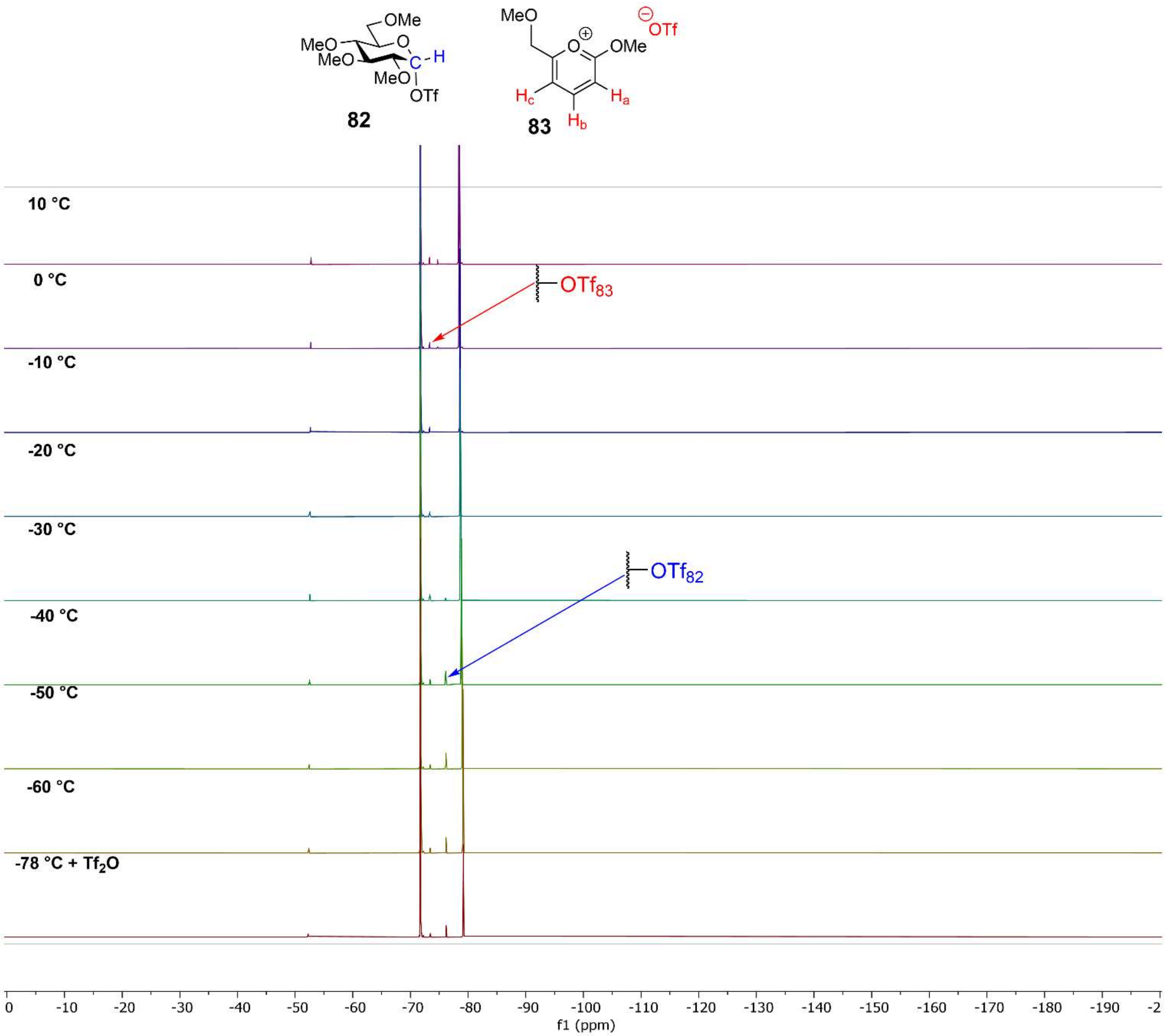

^{1}H NMR (500 MHz, CD_2Cl_2) spectrum of reaction mixture at -50 °C from VT NMR experiment with glucosyl sulfoxide 55:

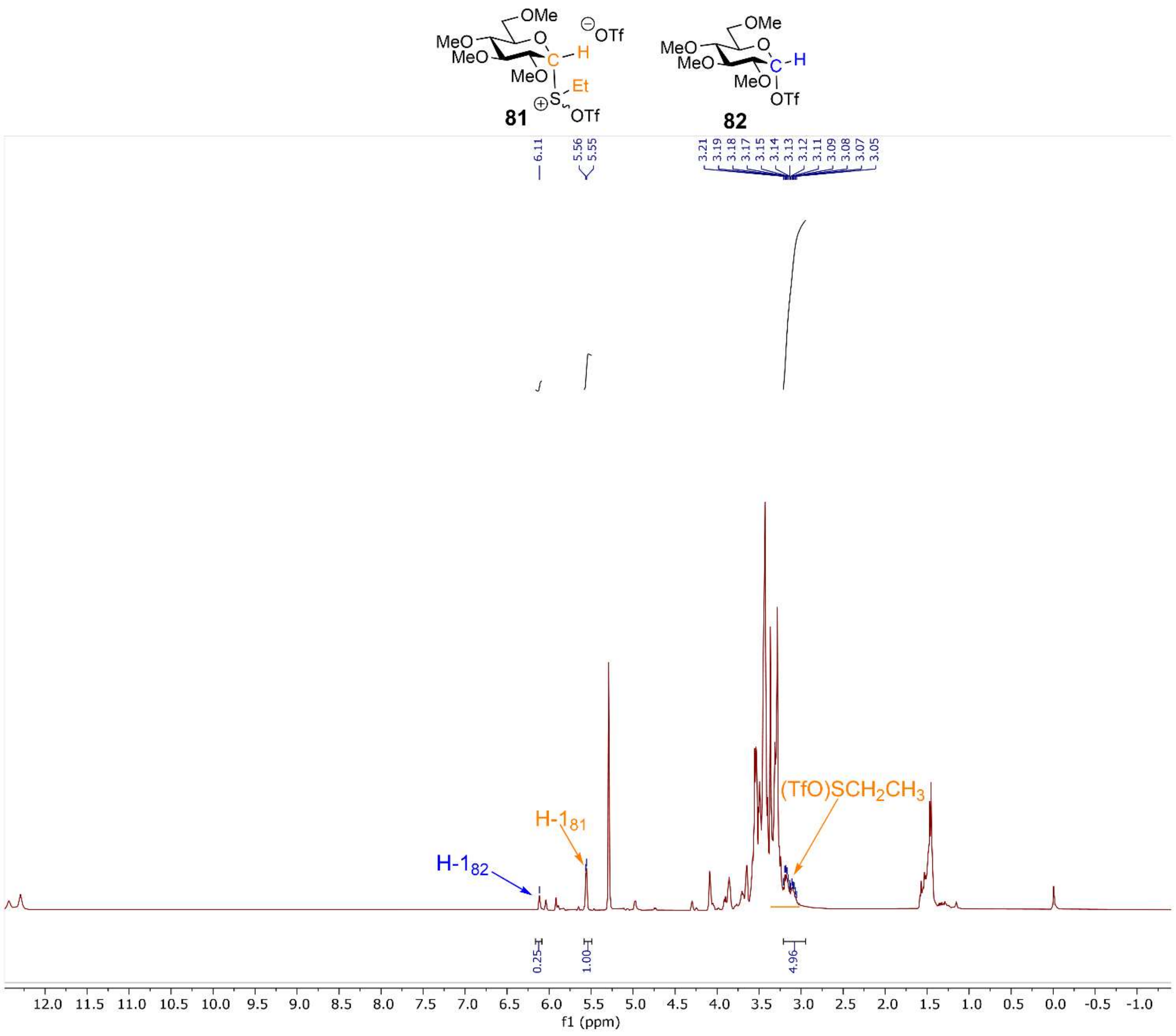

^{13}C NMR (125.67 MHz, CD_2Cl_2) spectrum of reaction mixture at -50 °C from VT NMR experiment with glucosyl sulfoxide 55:

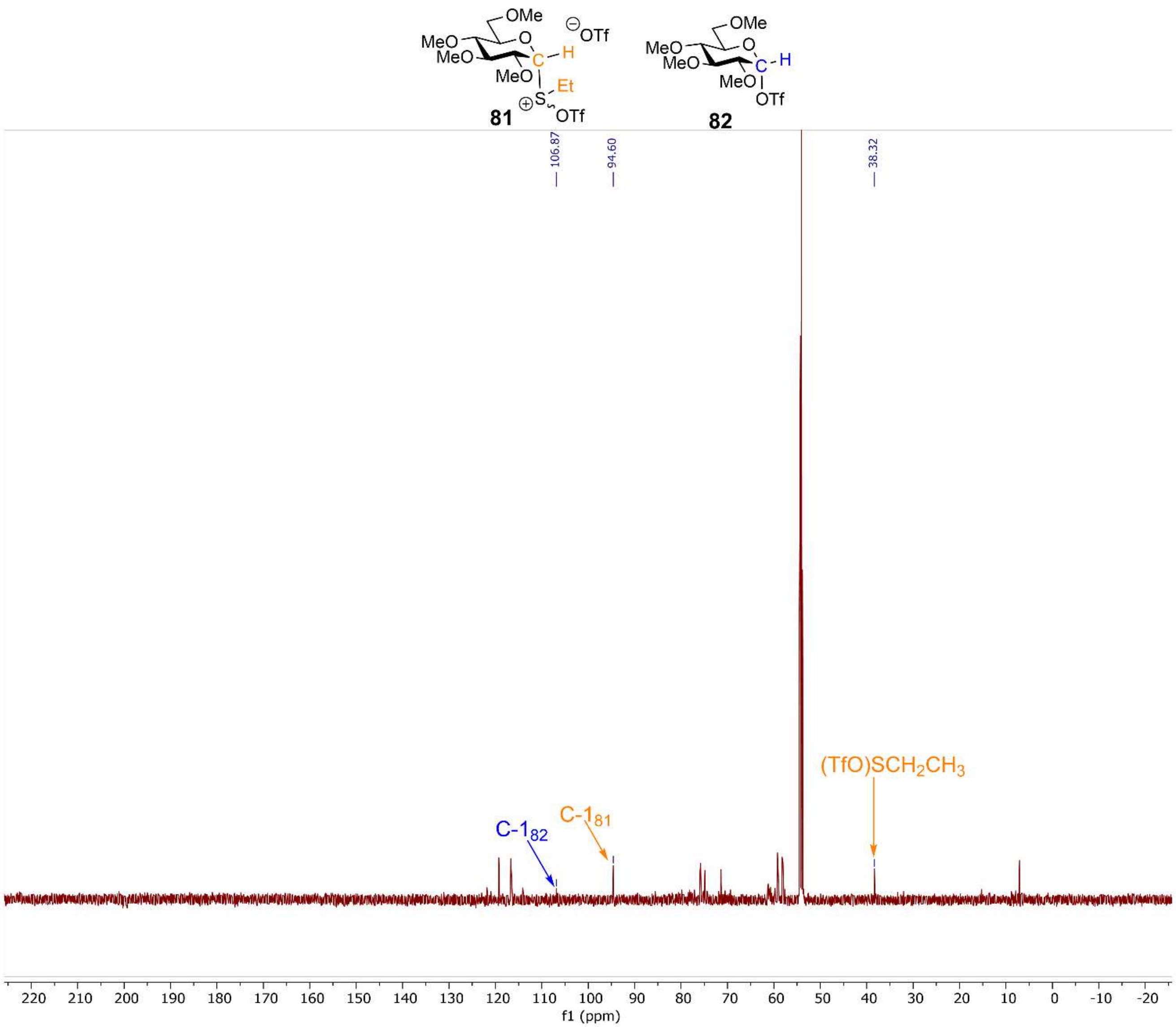

DEPT-90 (CD_2Cl_2) spectrum of reaction mixture at -50 °C from VT NMR experiment with glucosyl sulfoxide 55:

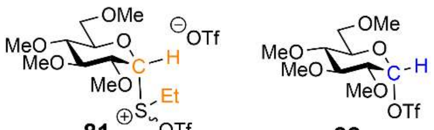

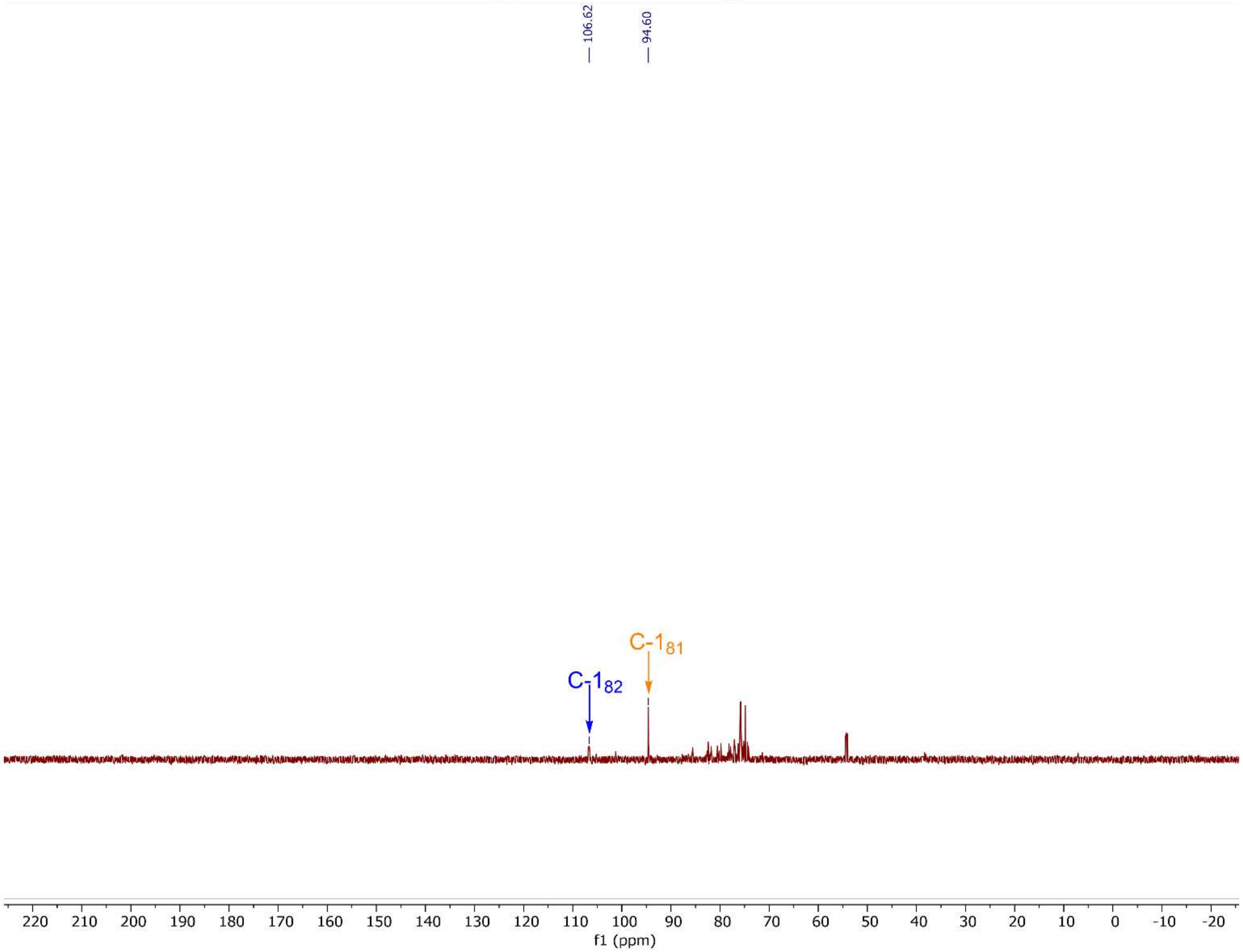

HSQC (CD_2Cl_2) spectrum of reaction mixture at -50 °C from VT NMR experiment with glucosyl sulfoxide 55:

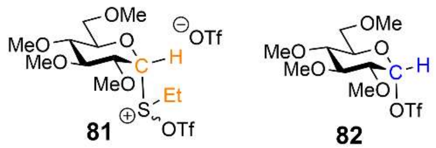

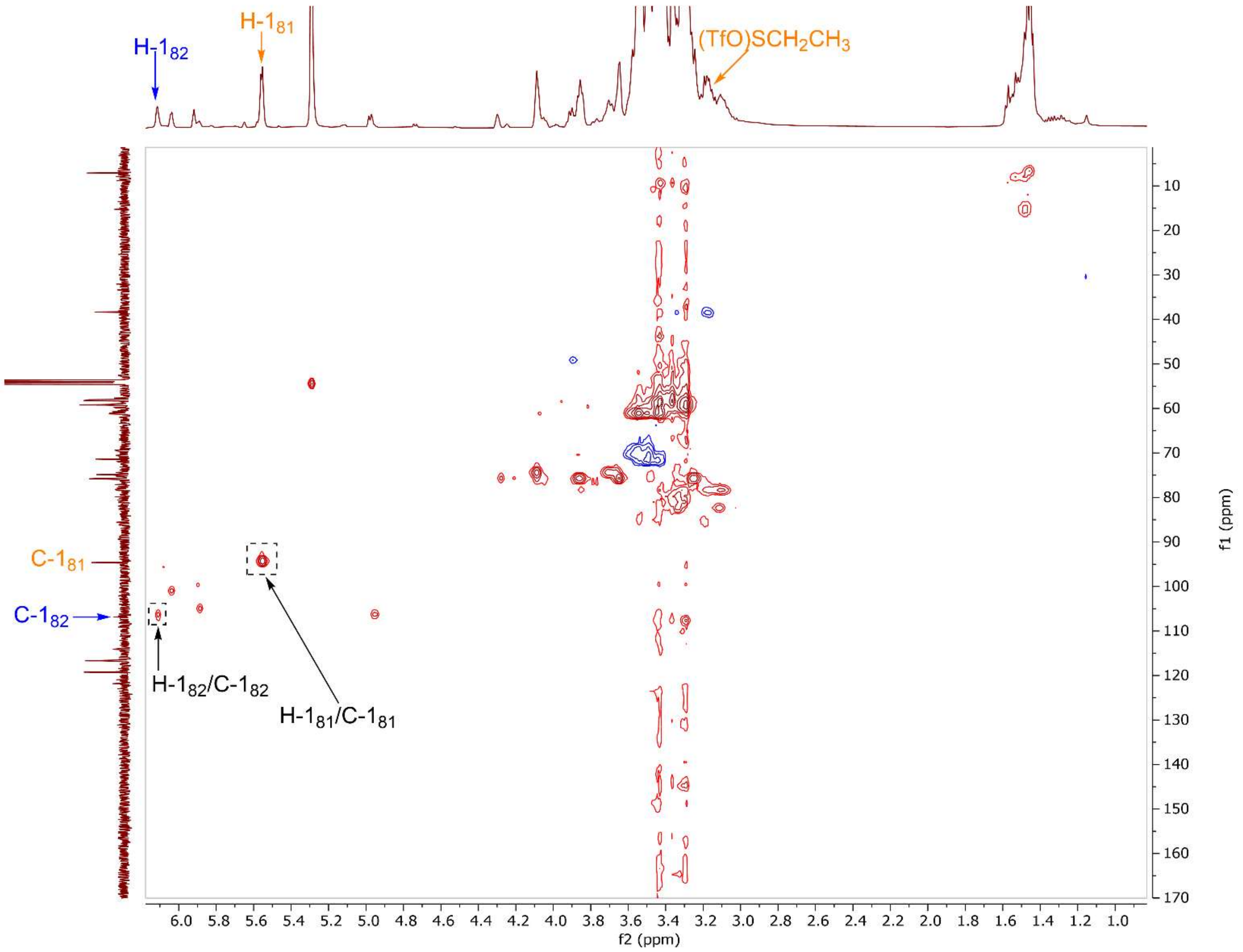

^{19}F NMR (470 MHz, CD_2Cl_2) spectrum of reaction mixture at -50 °C from VT NMR experiment with glucosyl sulfoxide 55:

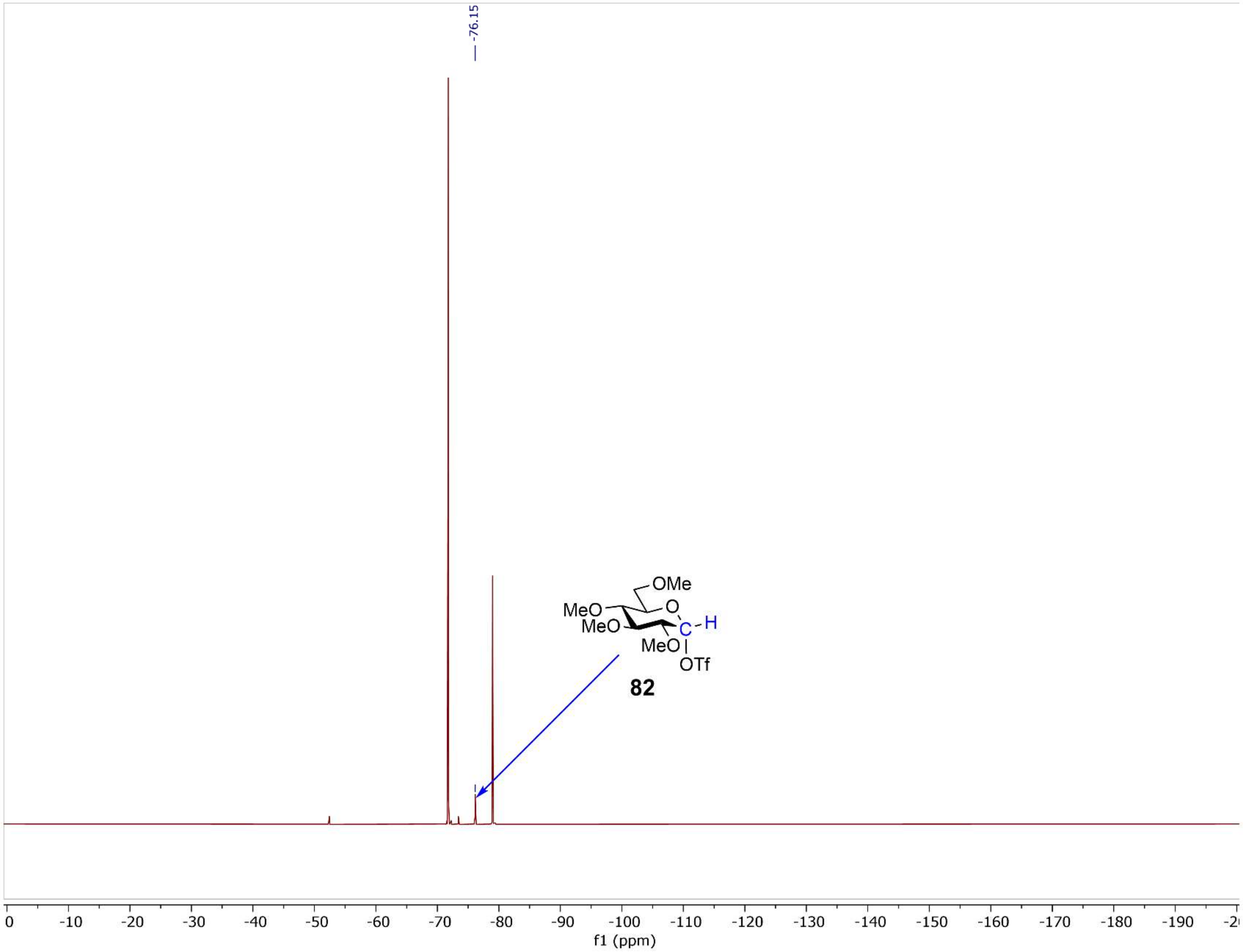

^{1}H NMR (500 MHz, CD_2Cl_2) spectrum of reaction mixture at 25 °C from VT NMR experiment with glucosyl sulfoxide 55:

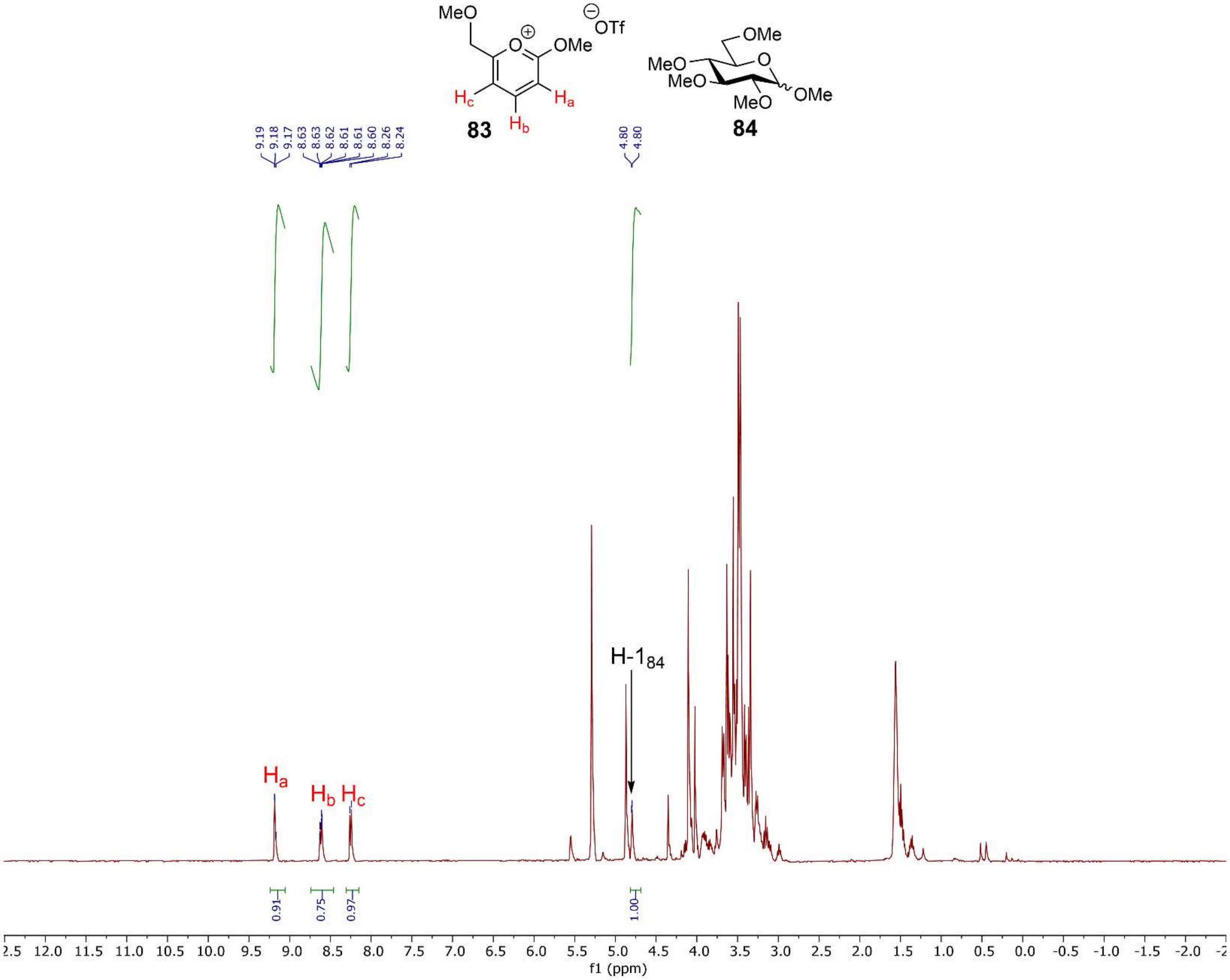

^{19}F NMR (470 MHz, CD_2Cl_2) spectrum of reaction mixture at 25 °C from VT NMR experiment with glucosyl sulfoxide 55:

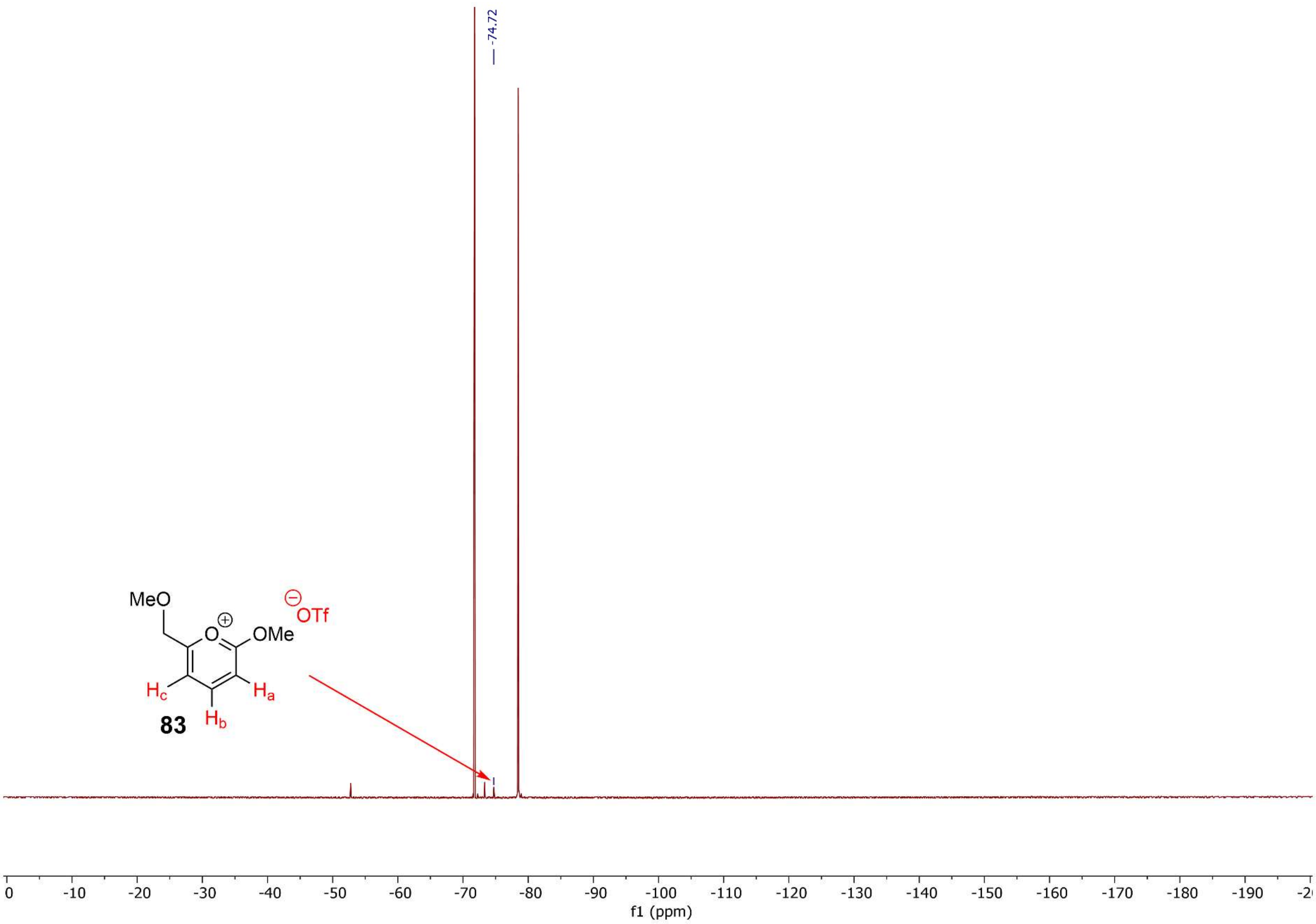

3.11. VT NMR spectra from experiments with permethylated 5-thioglucosyl sulfoxides (45, 48):

Stacked ^1H NMR (500 MHz, CD_2Cl_2) spectra from VT NMR experiment with 5-thioglucosyl sulfoxides 45:

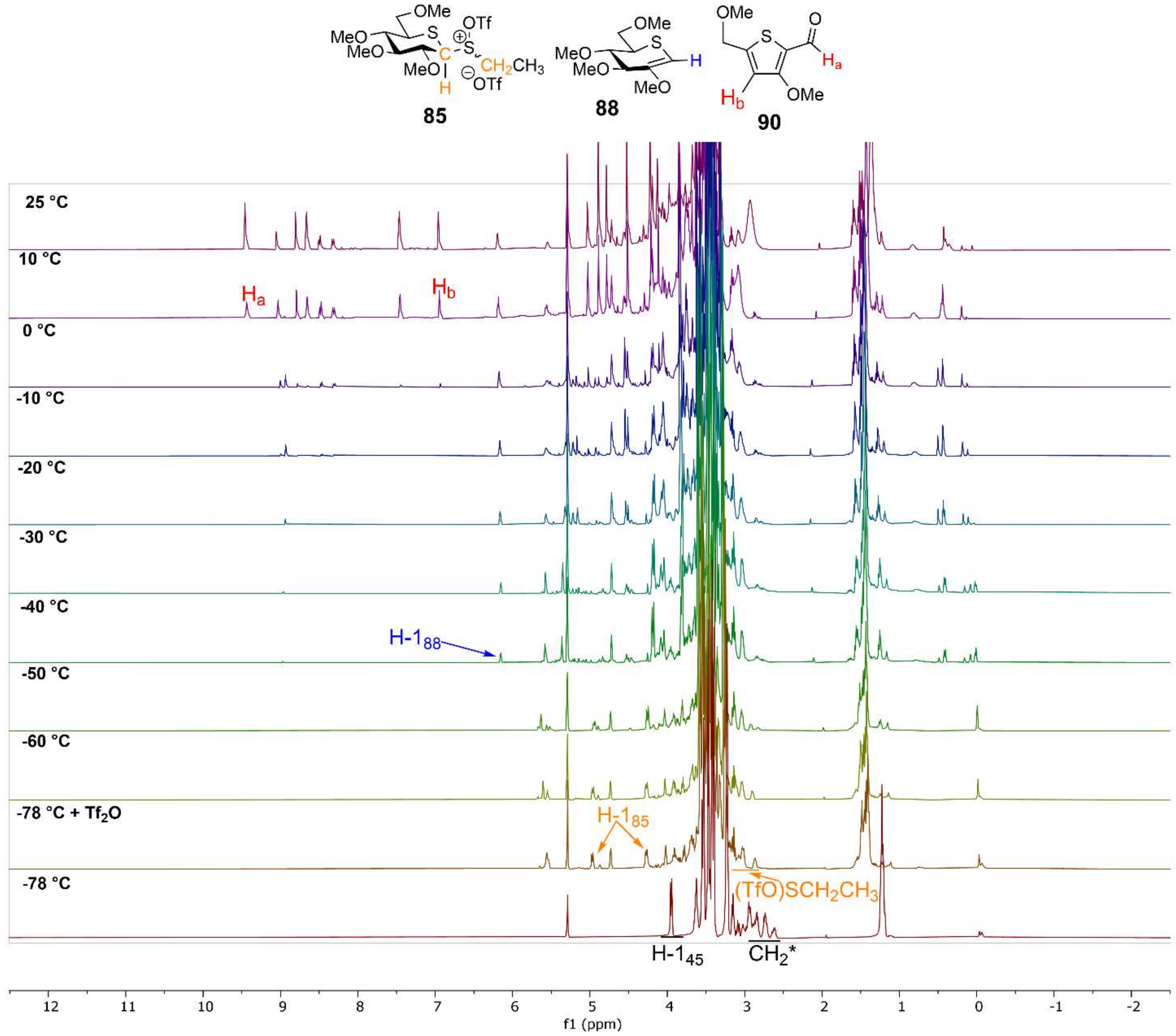

Stacked ^{19}F NMR (470 MHz, CD_2Cl_2) spectra from VT NMR experiment with 5-thioglucosyl sulfoxides 45:

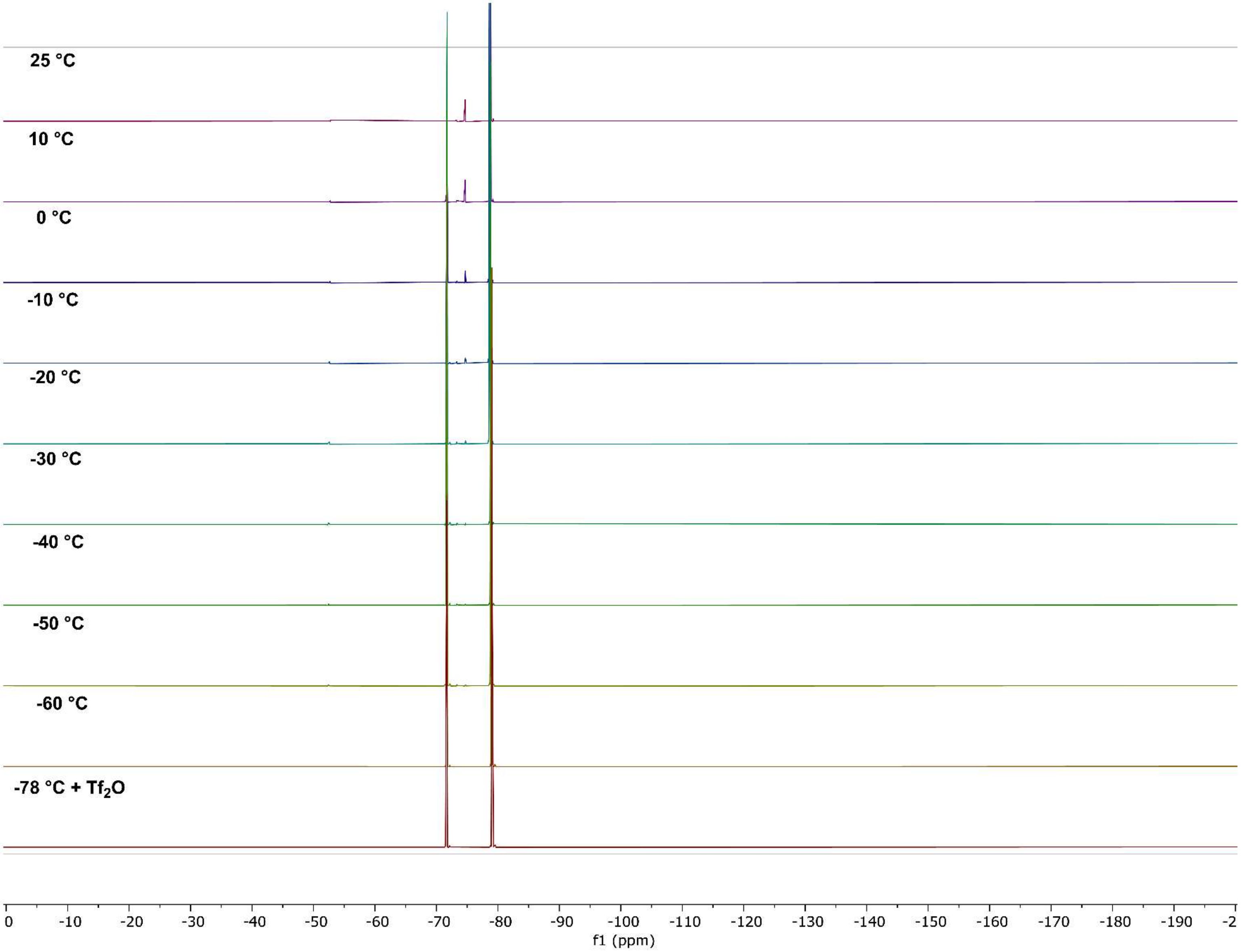

^{1}H NMR (500 MHz, CD_2Cl_2) spectrum of reaction mixture at -50 °C from VT NMR experiment with 5-thioglucosyl sulfoxide 45:

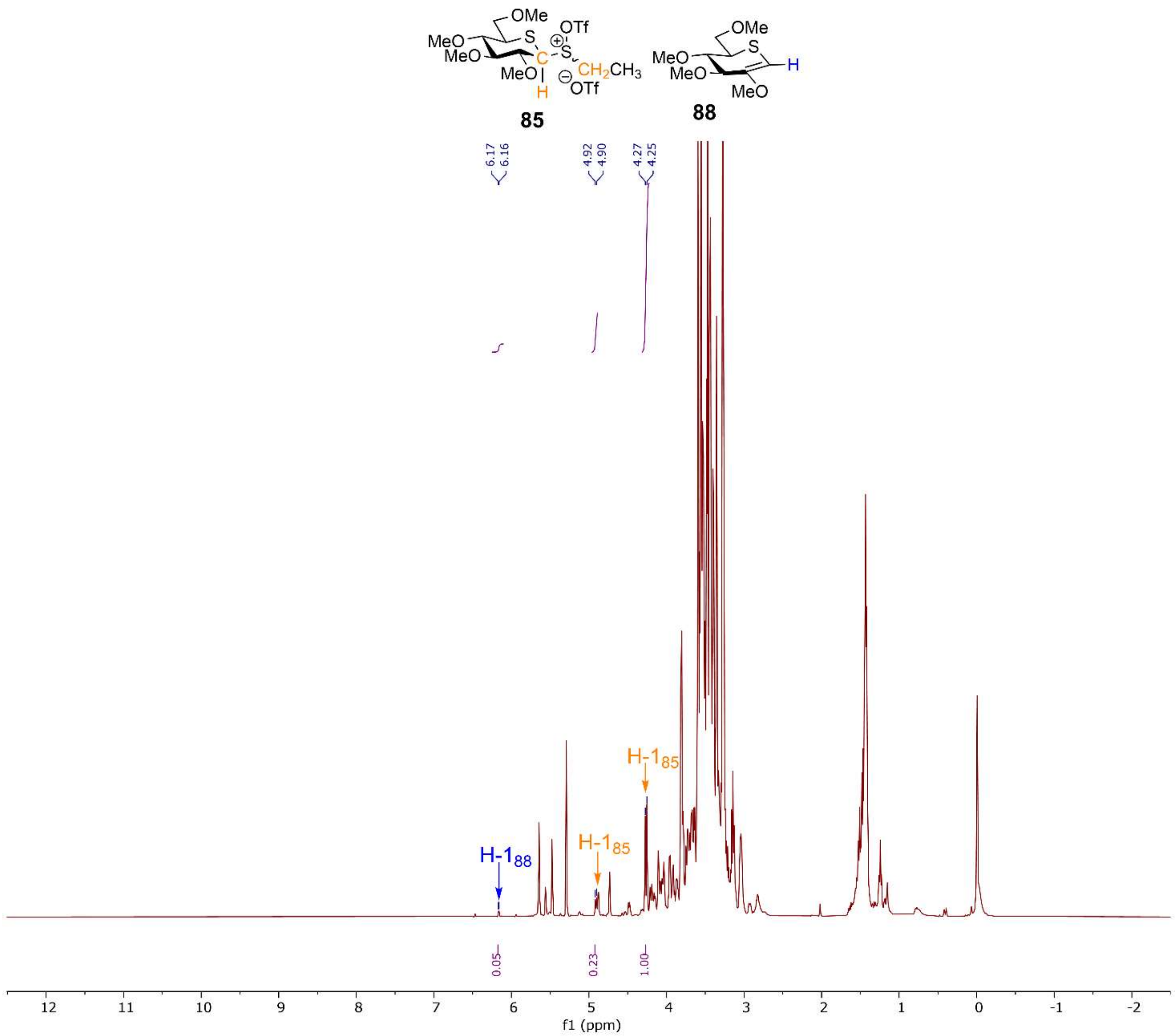

^{13}C NMR (125.67 MHz, CD_2Cl_2) spectrum of reaction mixture at -50 °C from VT NMR experiment with 5-thioglucosyl sulfoxide 45:

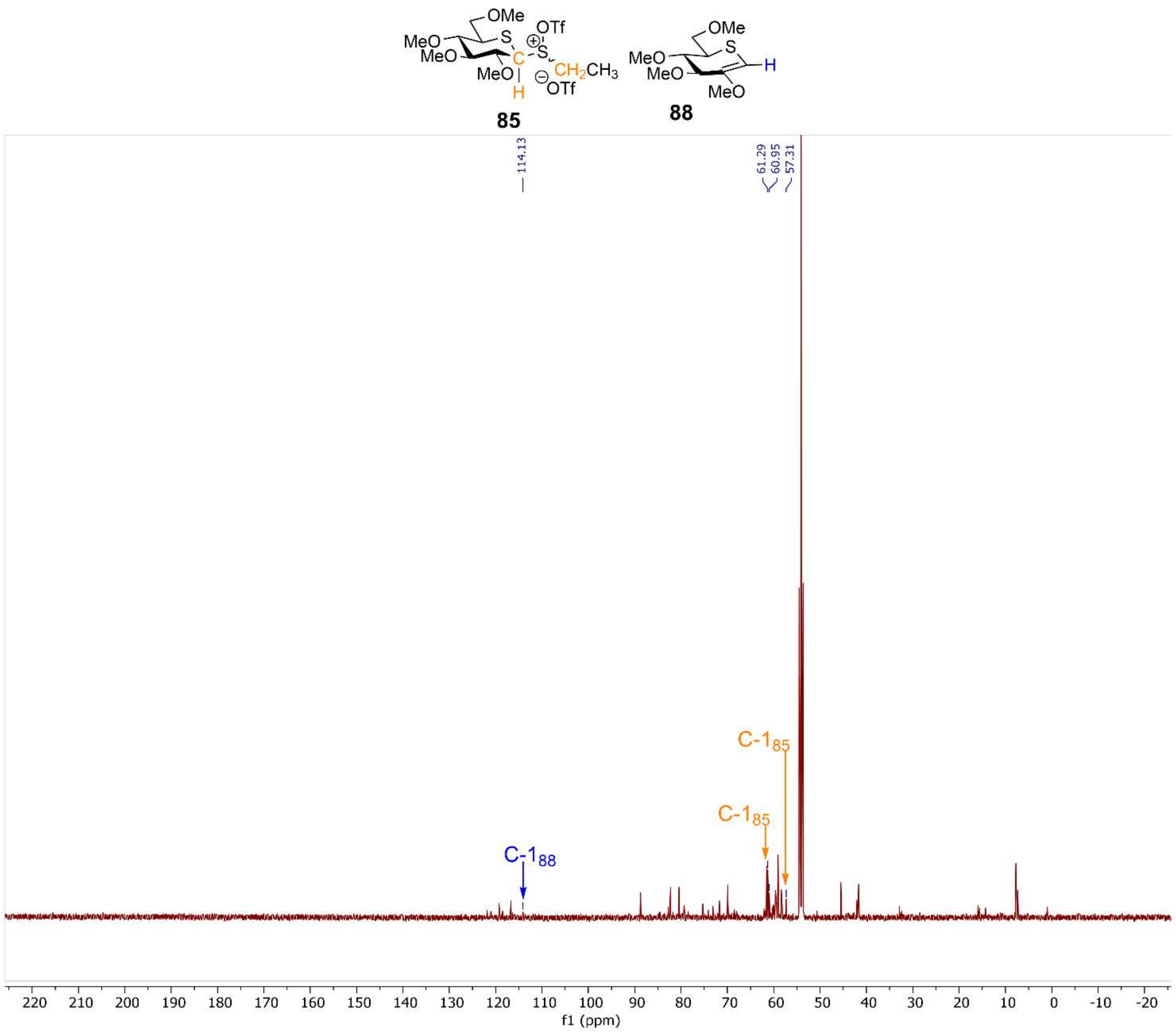

DEPT-90 (CD_2Cl_2) spectrum of reaction mixture at -50 °C from VT NMR experiment with 5-thioglucosyl sulfoxide 45:

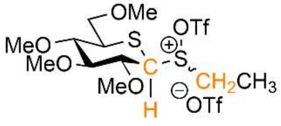

85

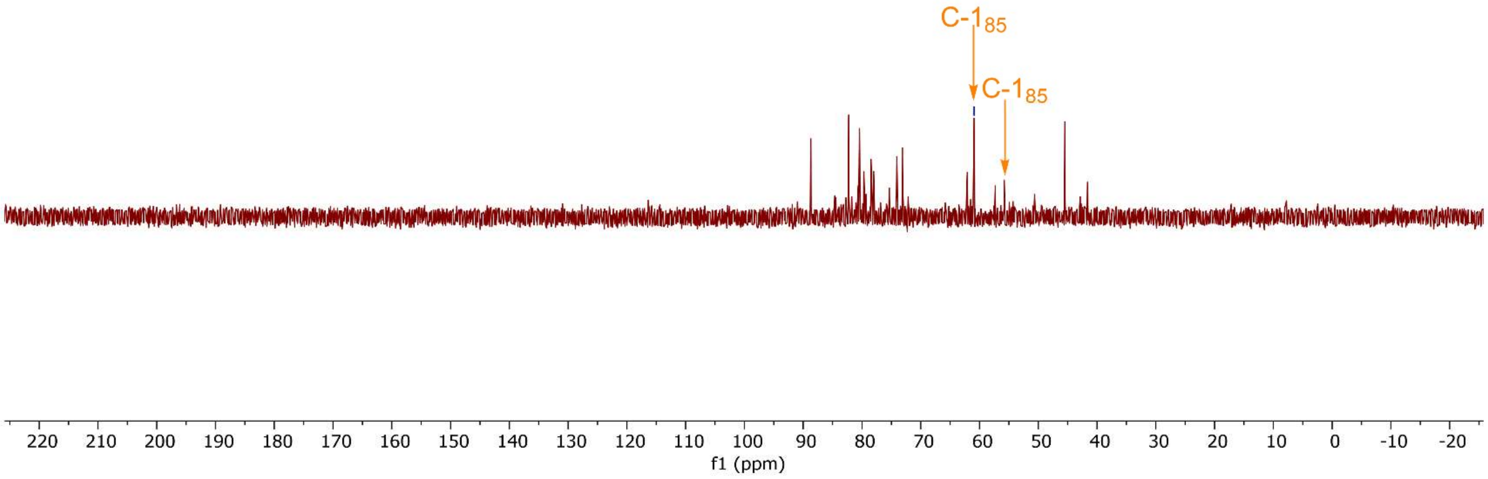

HMQC (CD_2Cl_2) spectrum of reaction mixture at -50 °C from VT NMR experiment with 5-thioglucosyl sulfoxide 45:

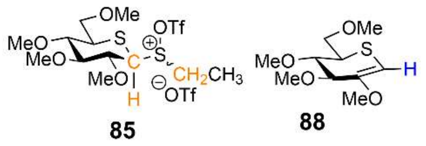

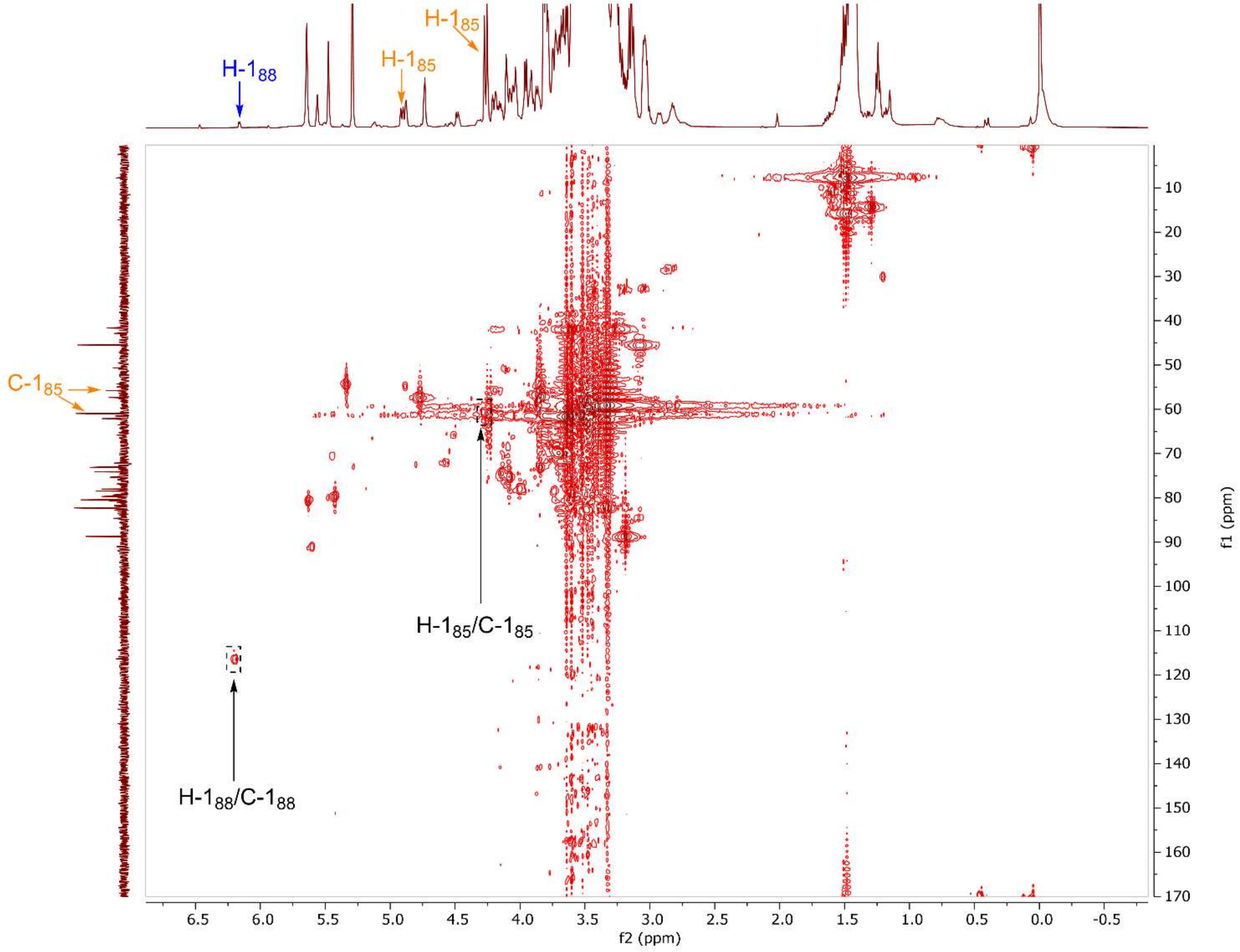

^{19}F (470 MHz, CD_2Cl_2) spectrum of reaction mixture at -50 °C from VT NMR experiment with 5-thioglucosyl sulfoxide 45:

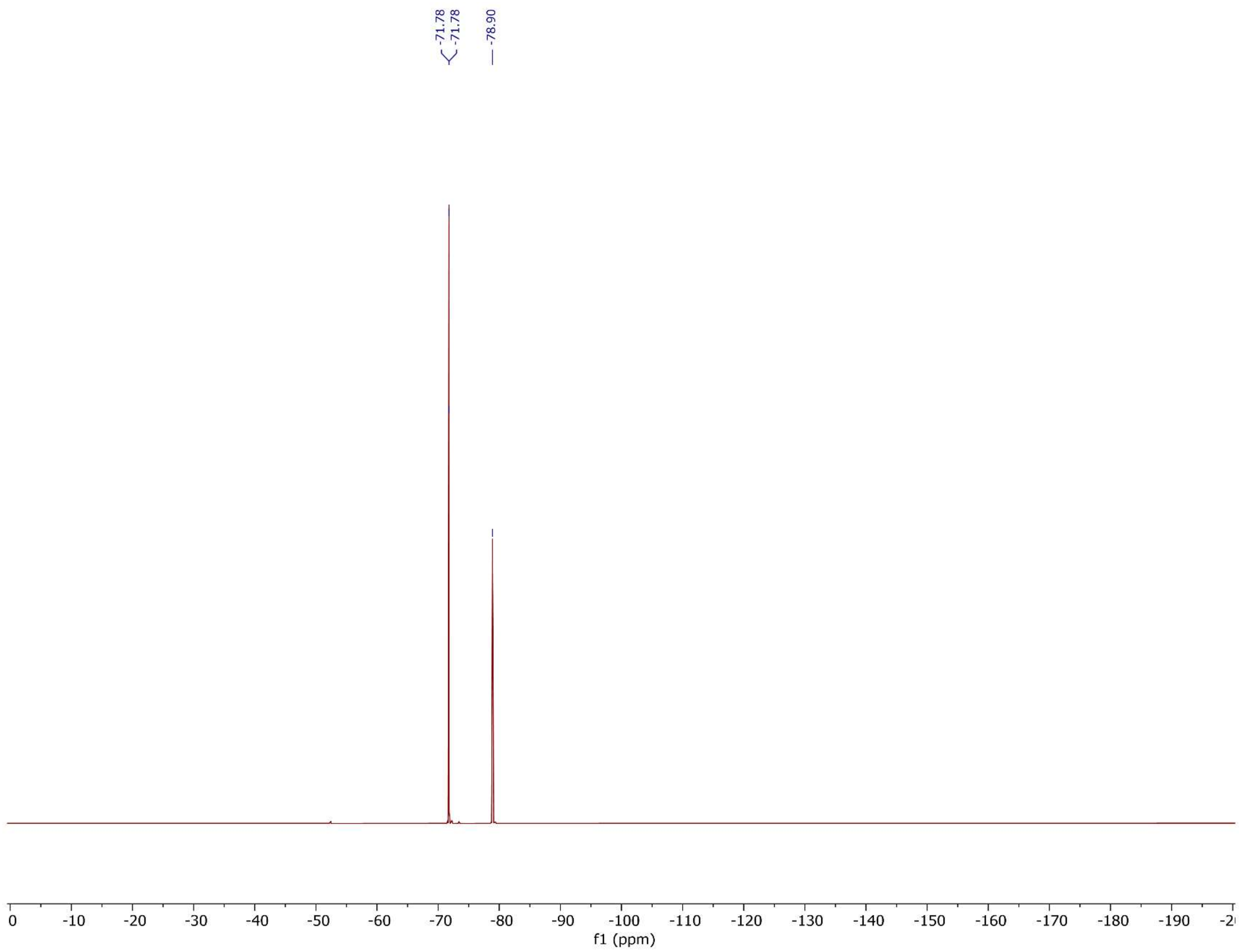

Stacked ^{1}H NMR (500 MHz, CD_2Cl_2) spectra from VT NMR experiment with 5-thioglucosyl sulfoxides 48:

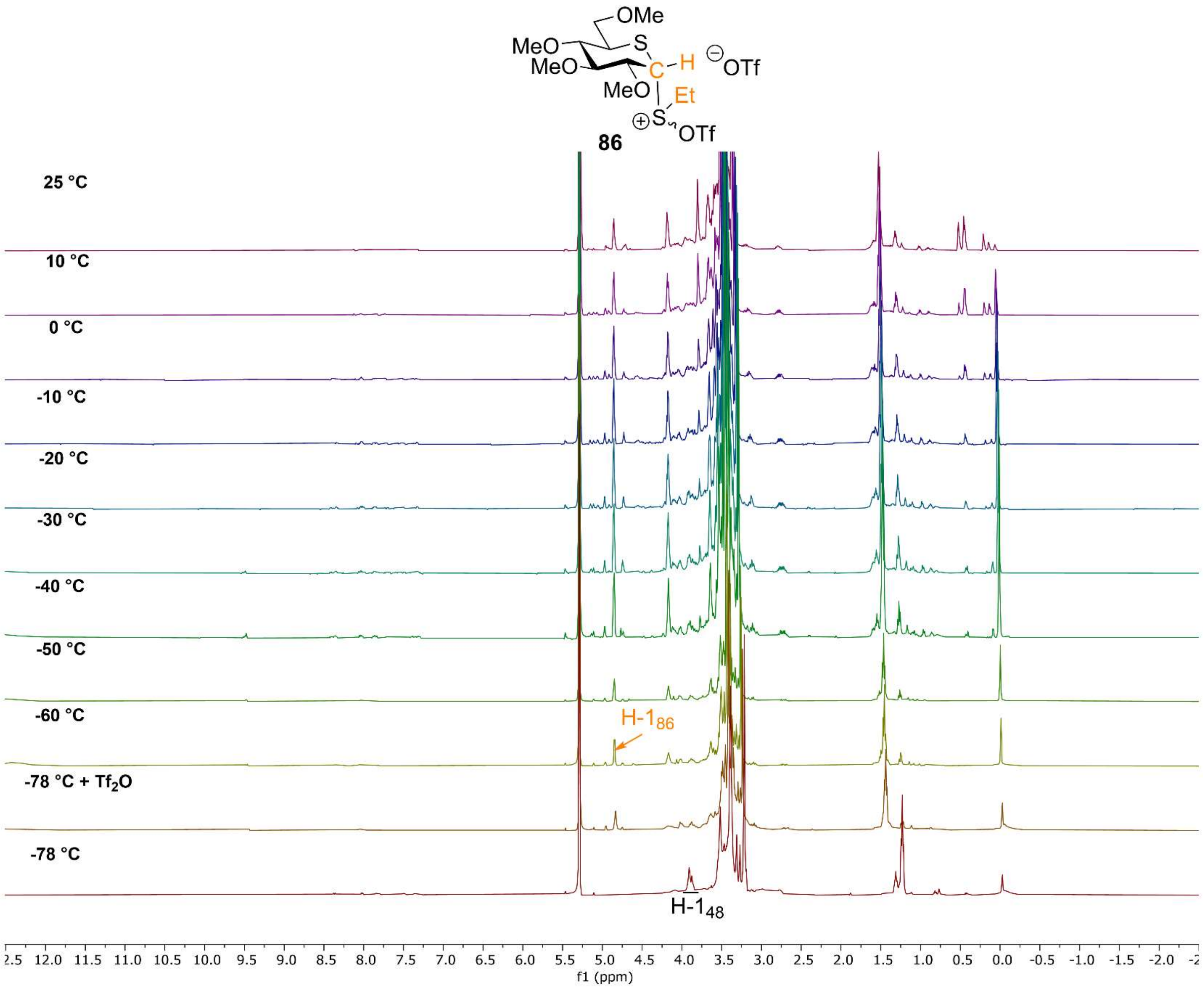

Stacked ^{19}F NMR (470 MHz, CD_2Cl_2) spectra from VT NMR experiment with 5-thioglucosyl sulfoxides 48:

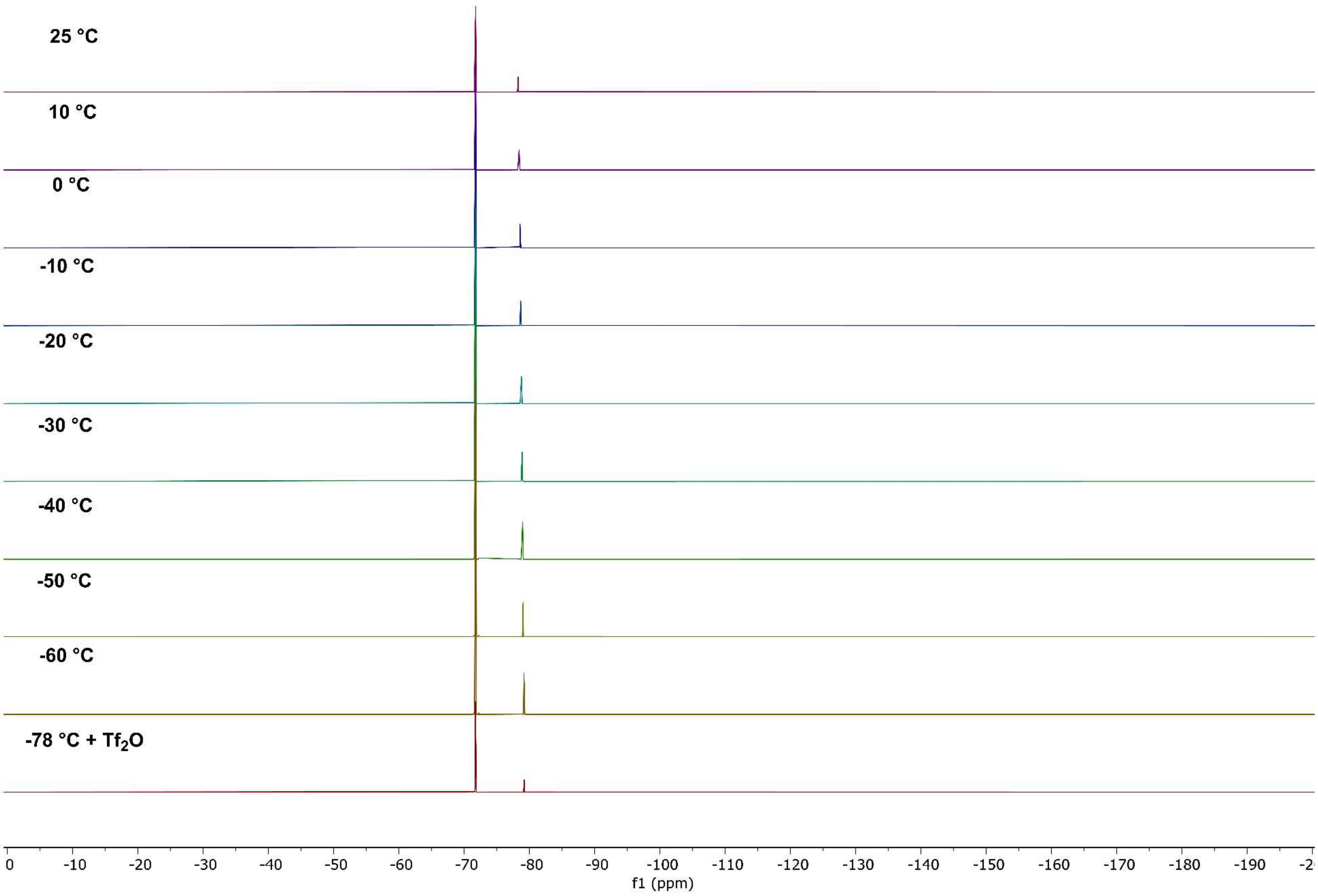

^{1}H NMR (500 MHz, CD_2Cl_2) spectrum of reaction mixture at -50 °C from VT NMR experiment with 5-thioglucosyl sulfoxide 45:

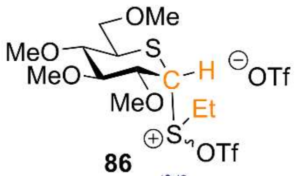

86

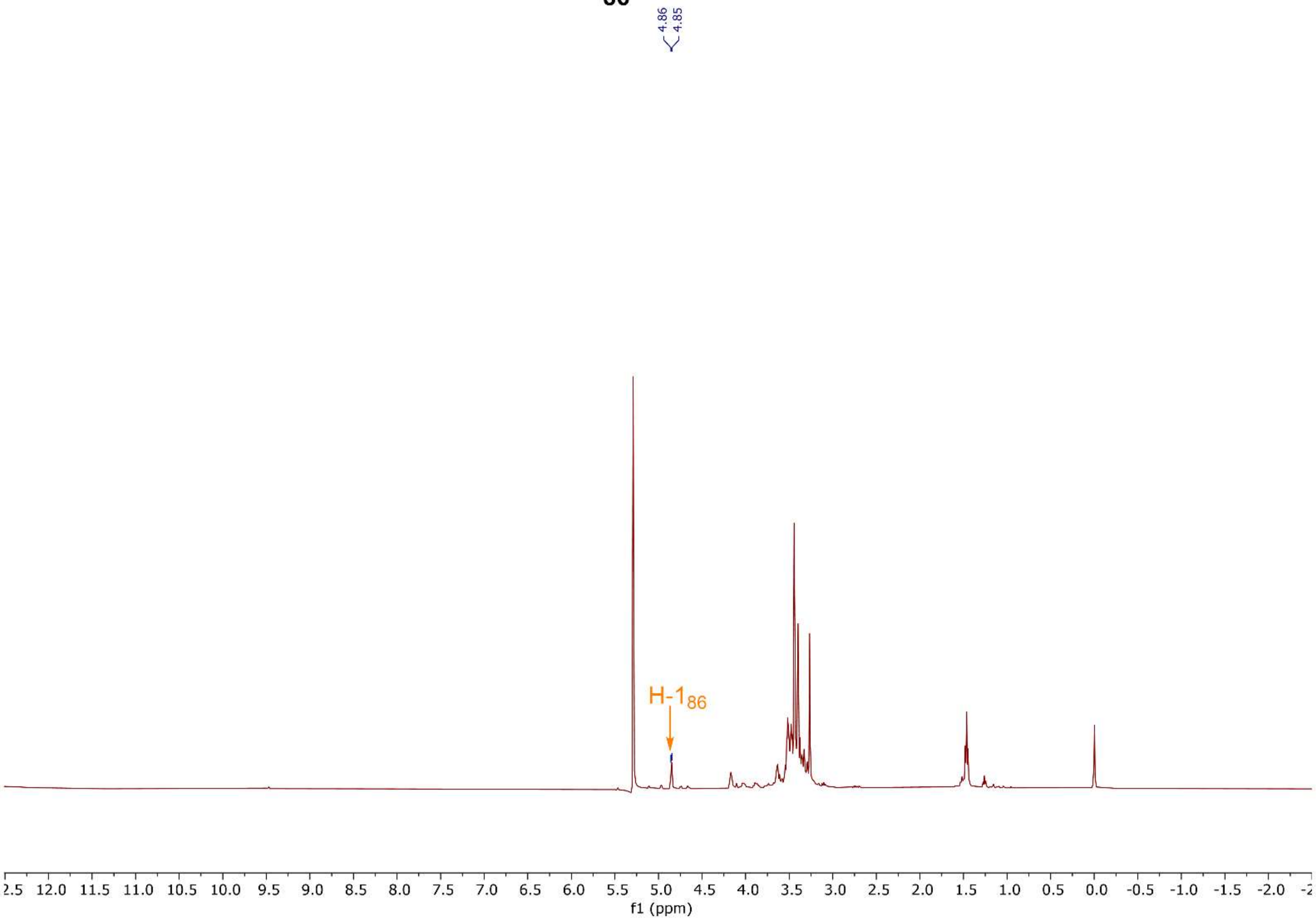

^{13}C NMR (125.67 MHz, CD_2Cl_2) spectrum of reaction mixture at -50 °C from VT NMR experiment with 5-thioglucosyl sulfoxide 45:

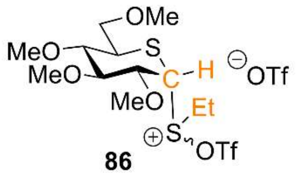

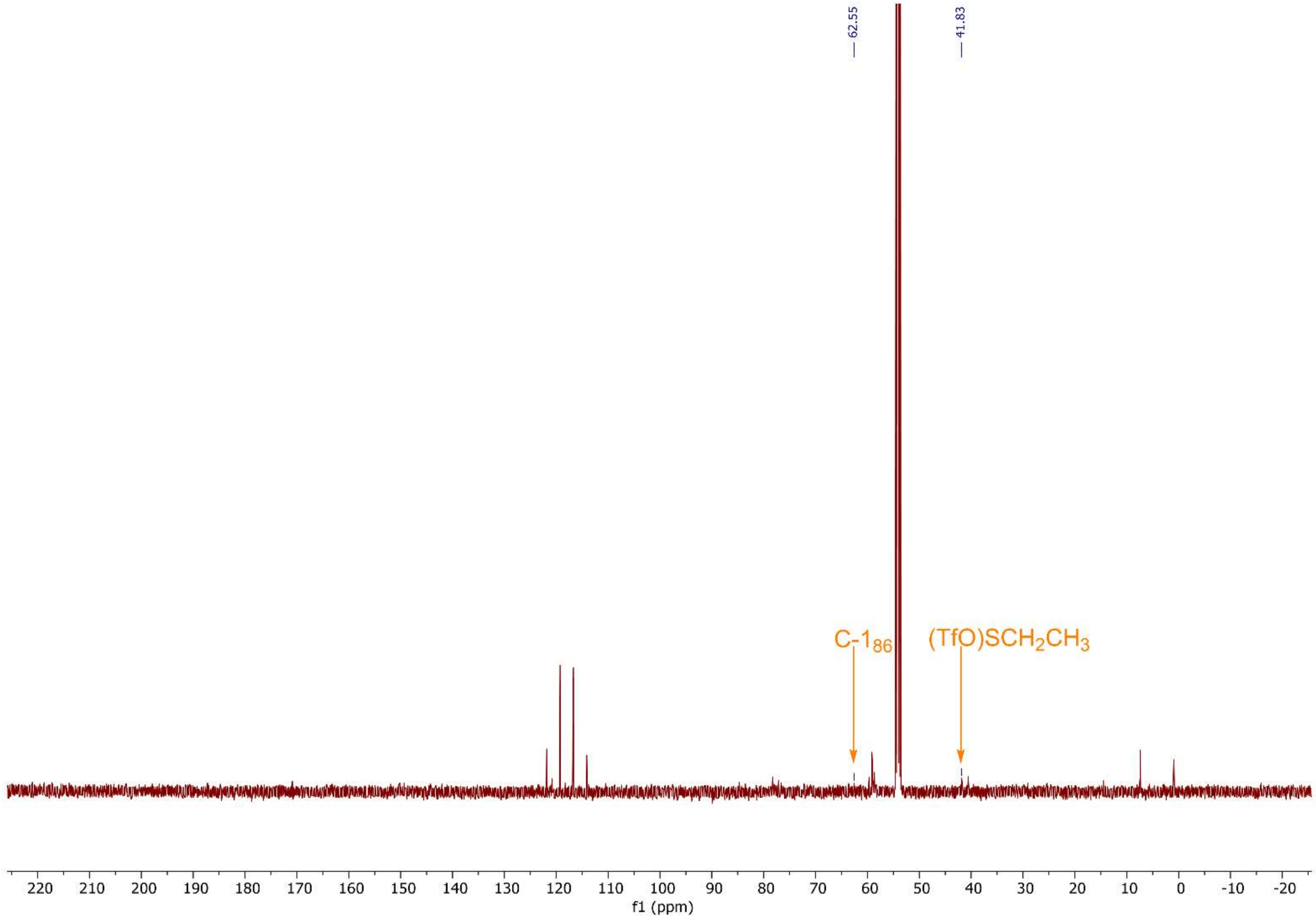

HMQC (CD_2Cl_2) spectrum of reaction mixture at -50 °C from VT NMR experiment with 5-thioglucosyl sulfoxide 45:

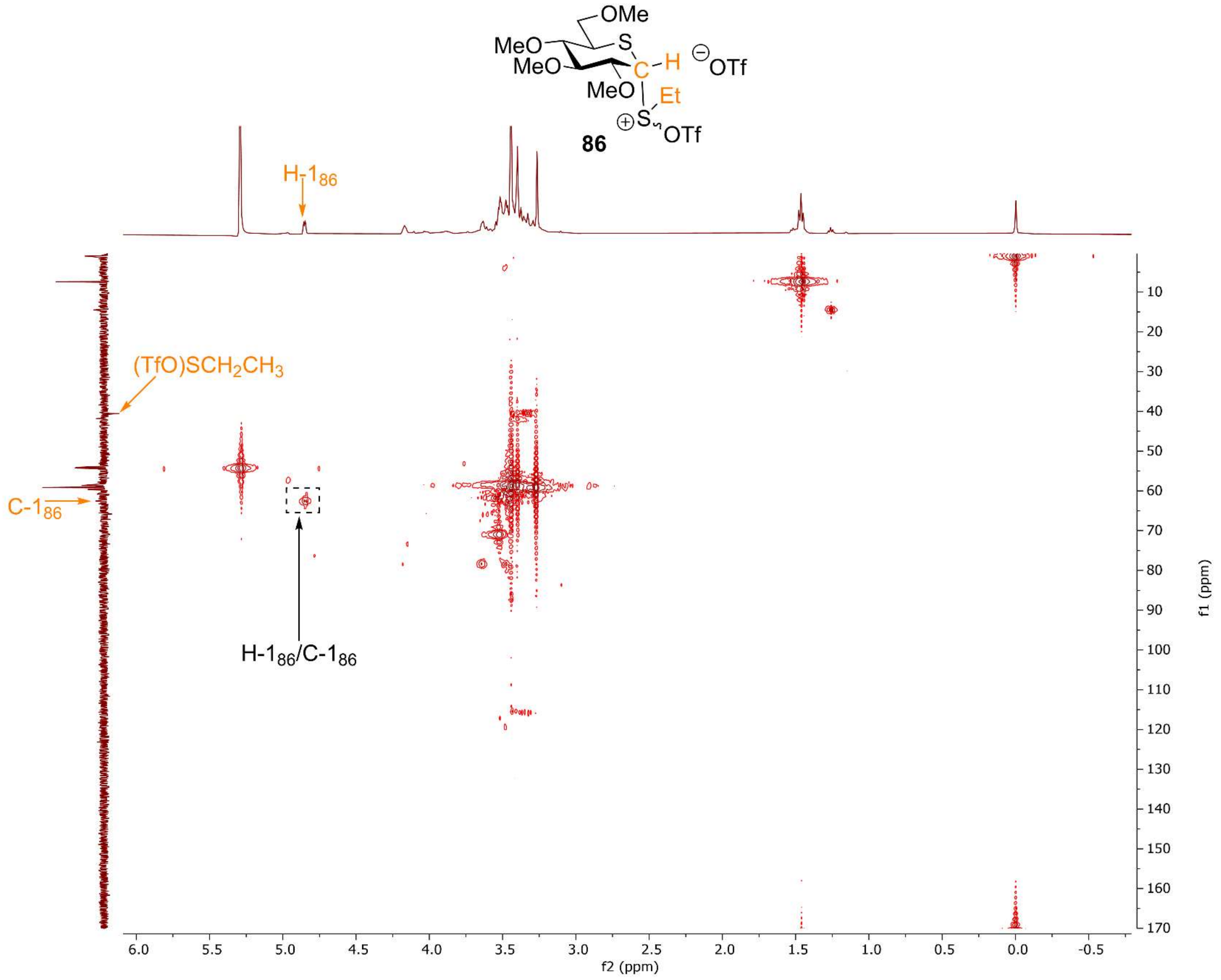

HMBC (CD_2Cl_2) spectrum of reaction mixture at -50 °C from VT NMR experiment with 5-thioglucosyl sulfoxide 45:

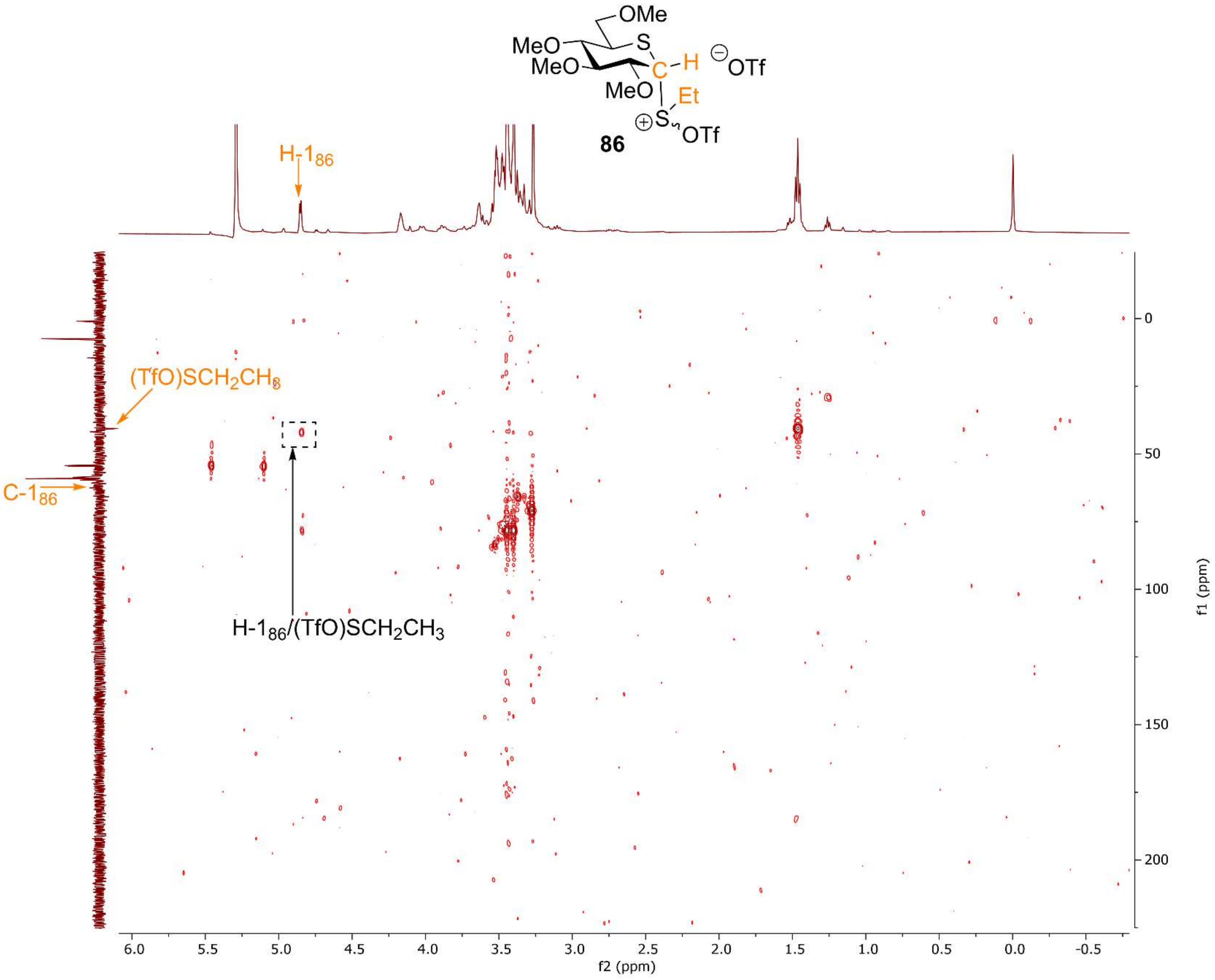

^{19}F NMR (470 MHz, CD_2Cl_2) spectrum of reaction mixture at -50 °C from VT NMR experiment with 5-thioglucosyl sulfoxide 45:

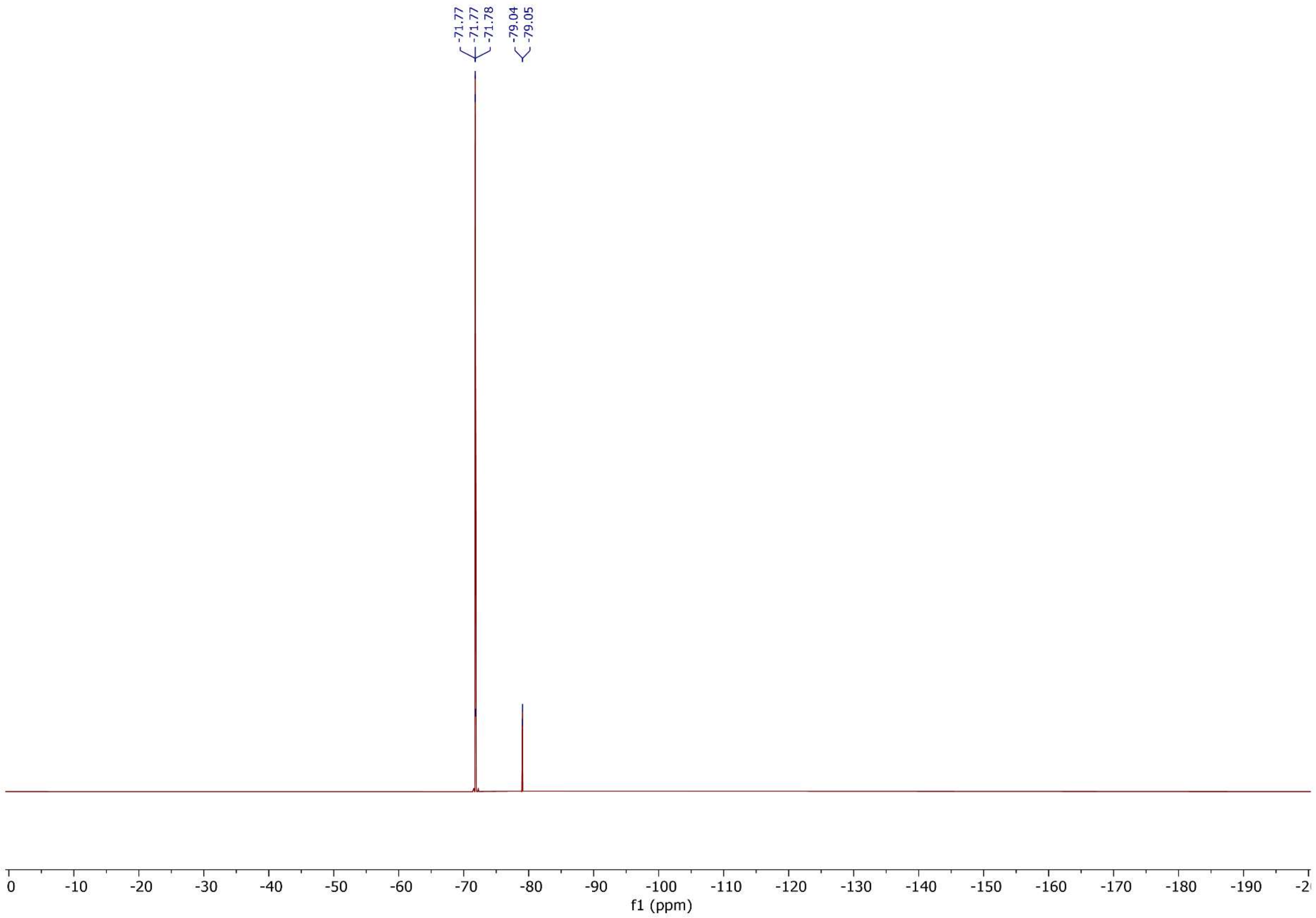

3.12. VT NMR spectra from experiments with permethylated glycosyl trichloroacetimidates (60 and 64):

Stacked ^1H NMR (500 MHz, CD_2Cl_2) spectra from VT NMR experiment with 5-thioglucosyl trichloroacetimidate (60):

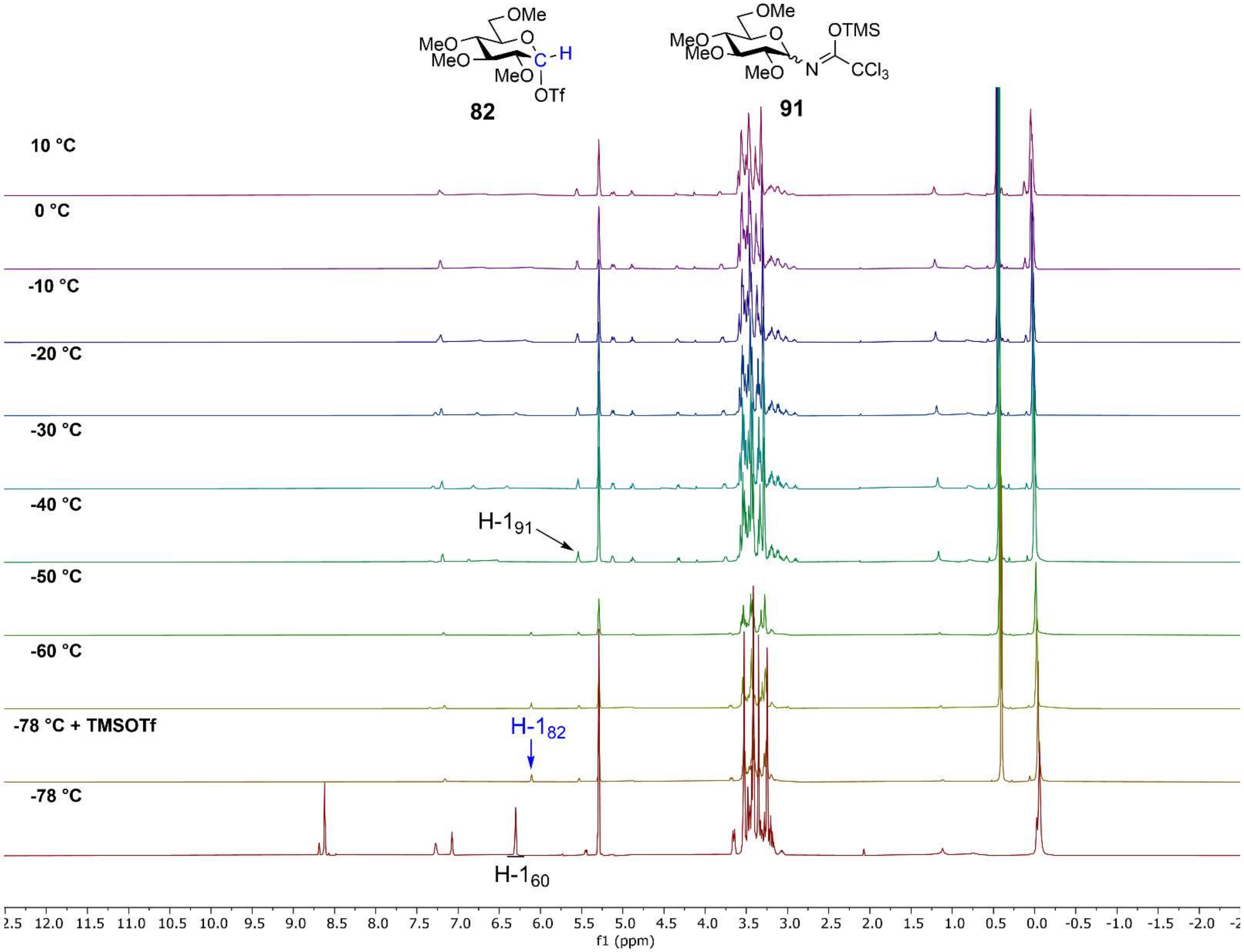

Stacked ^{19}F NMR (470 MHz, CD_2Cl_2) spectra from VT NMR experiment with 5-thioglucosyl trichloroacetimidate 60:

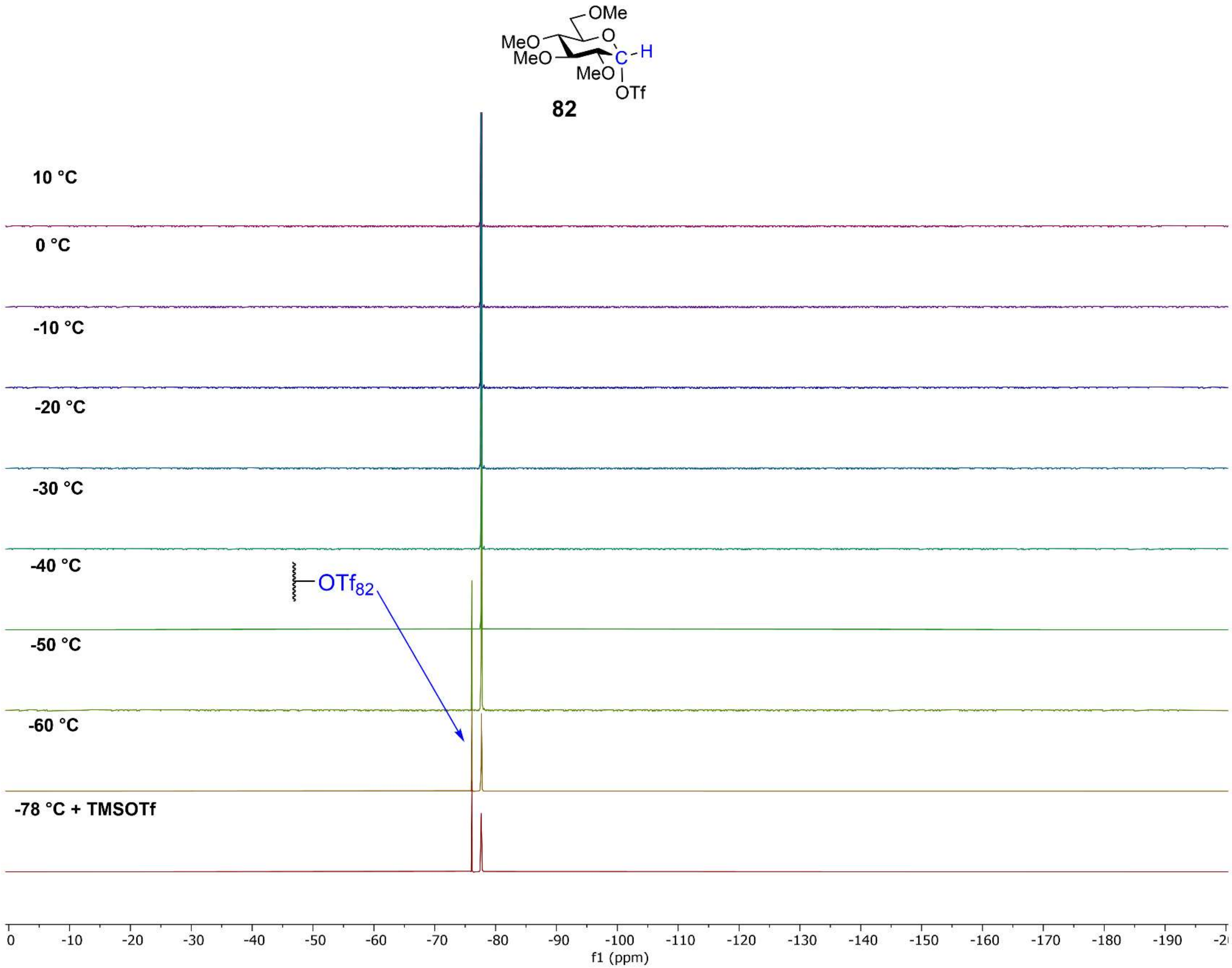

^{1}H NMR (500 MHz, CD_2Cl_2) spectrum of reaction mixture at -78 °C from VT NMR experiment with glucosyl trichloroacetimidate 60:

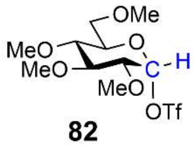

82

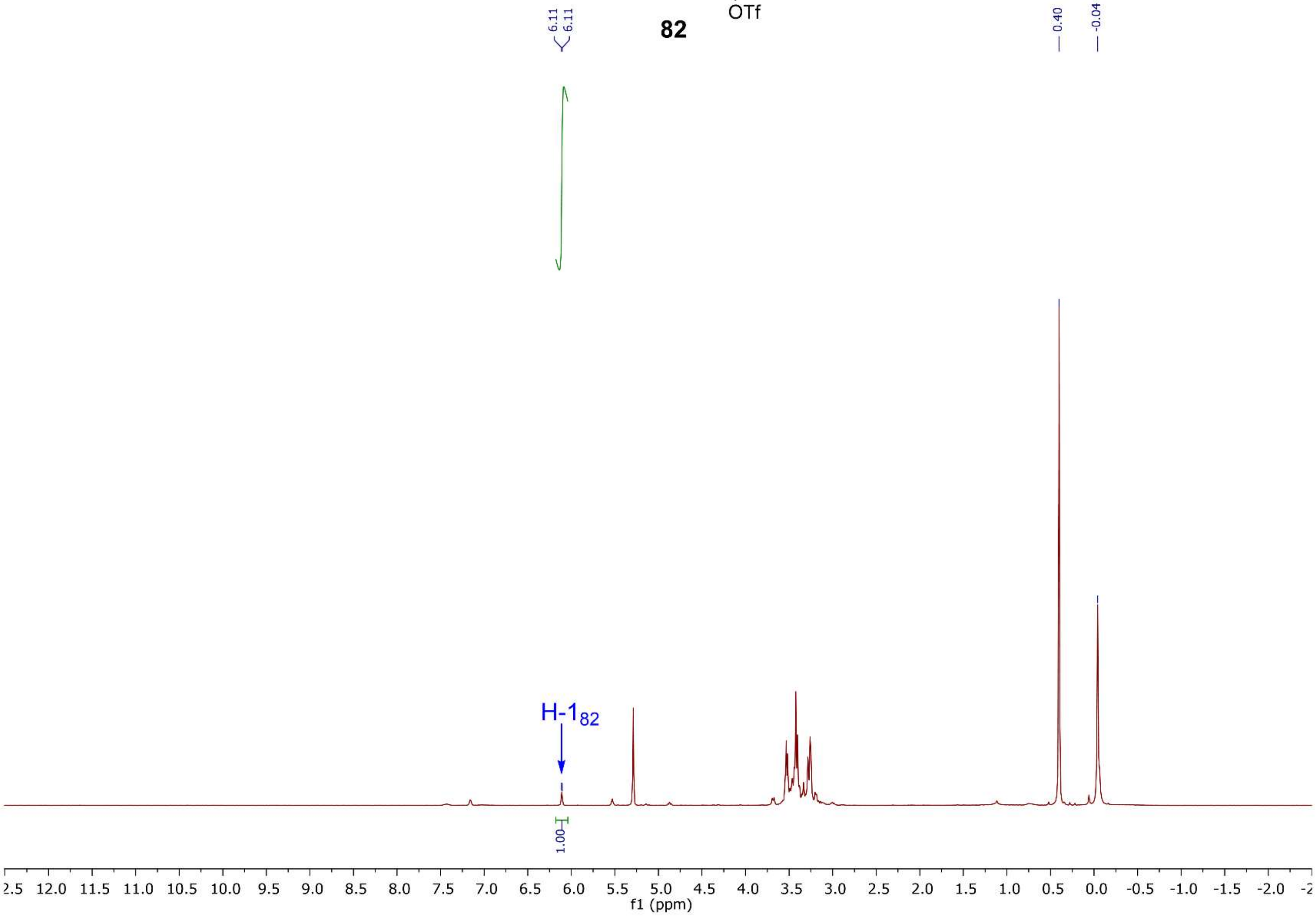

^{13}C NMR (125.67 MHz, CD_2Cl_2) spectrum of reaction mixture at -78 °C from VT NMR experiment with glucosyl trichloroacetimidate 60:

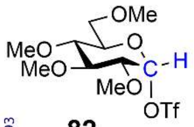

82

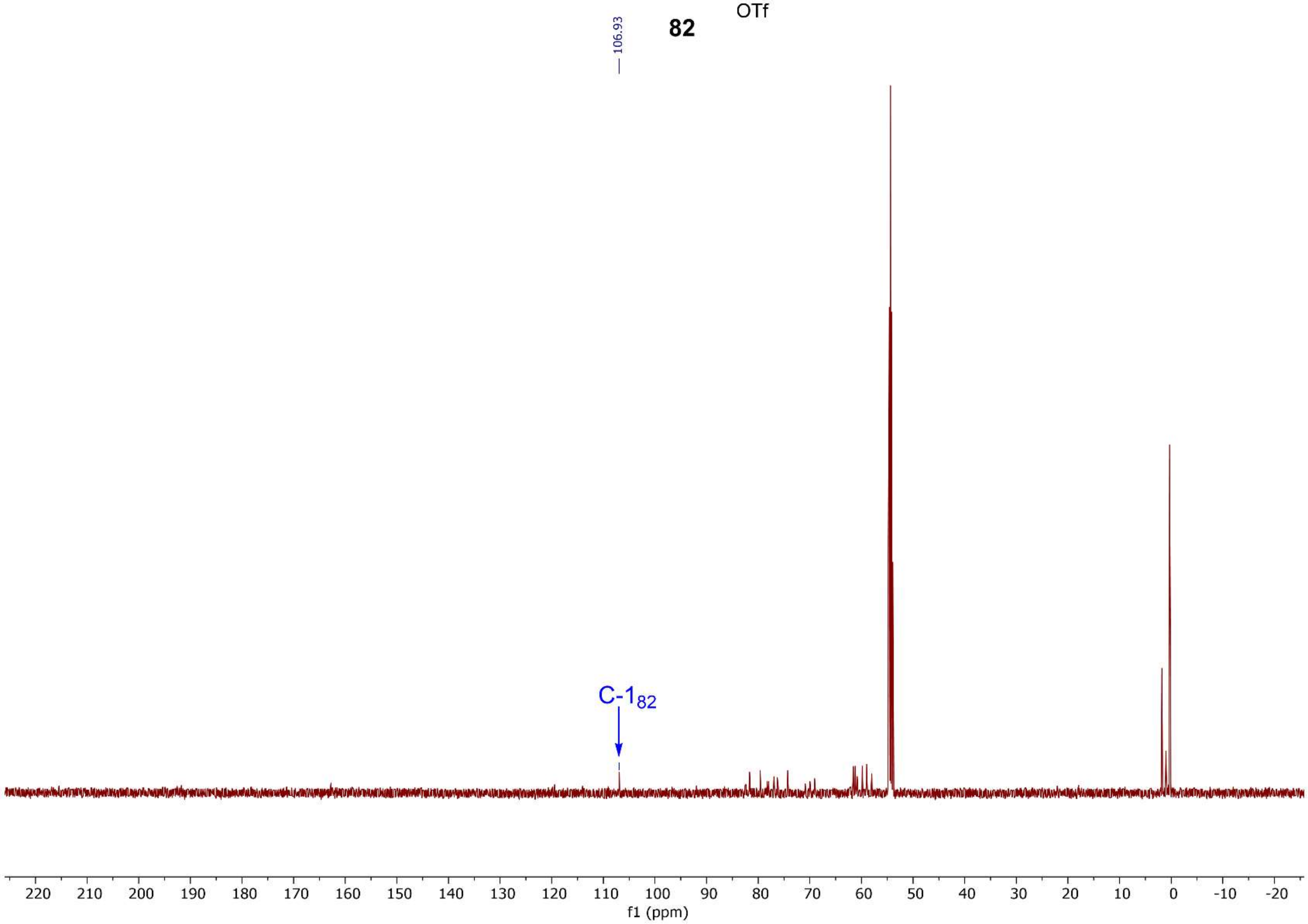

DEPT1-135 (CD_2Cl_2) spectrum of reaction mixture at -78 °C from VT NMR experiment with glucosyl trichloroacetimidate 60:

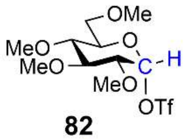

82

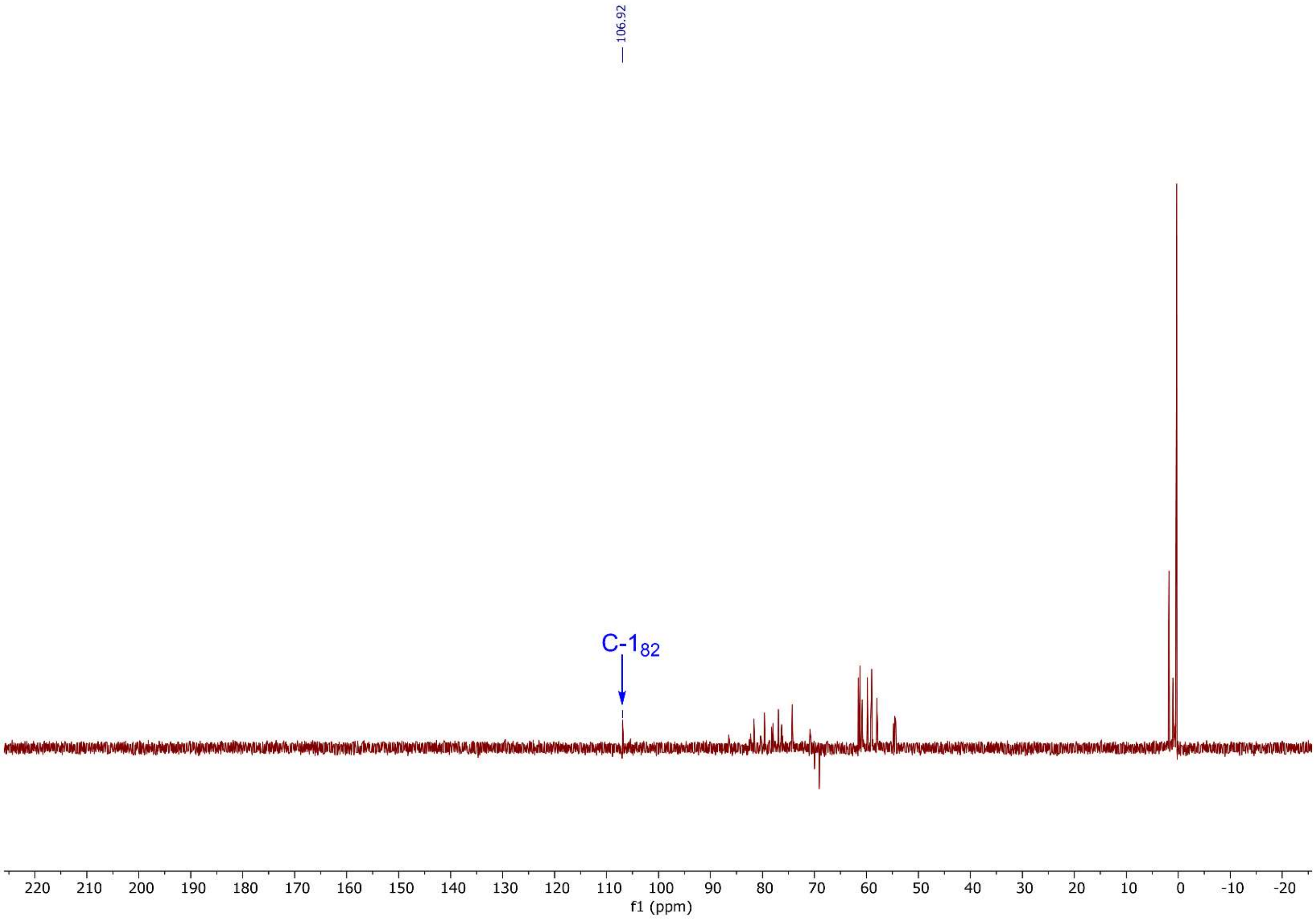

HSQC (CD_2Cl_2) spectrum of reaction mixture at -78 °C from VT NMR experiment with glucosyl trichloroacetimidate 60:

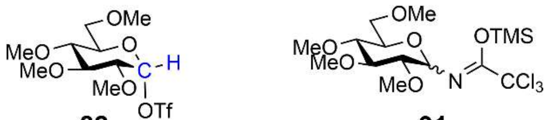

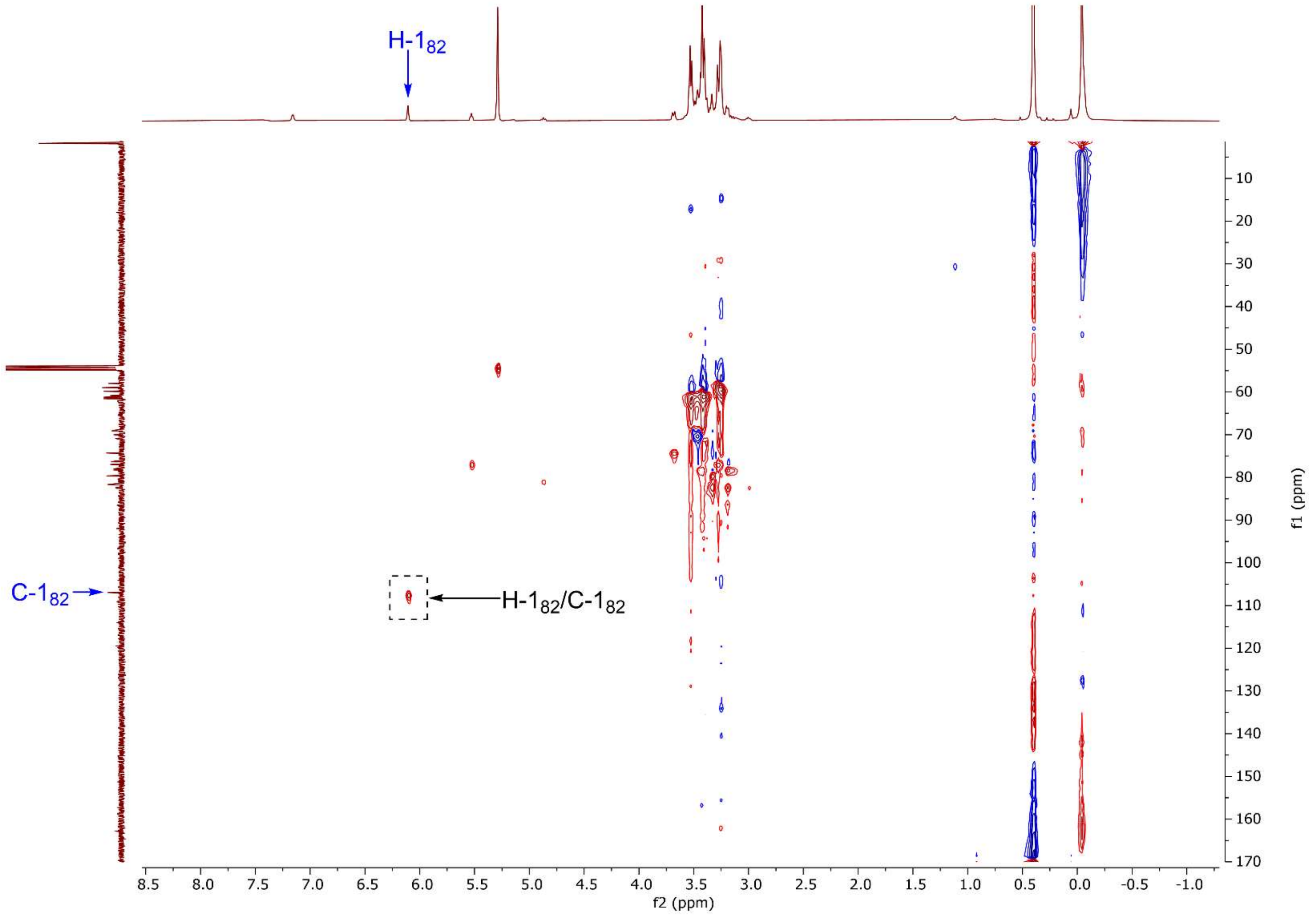

^{19}F NMR (470 MHz, CD_2Cl_2) spectrum of reaction mixture at -78 °C from VT NMR experiment with glucosyl trichloroacetimidate 60:

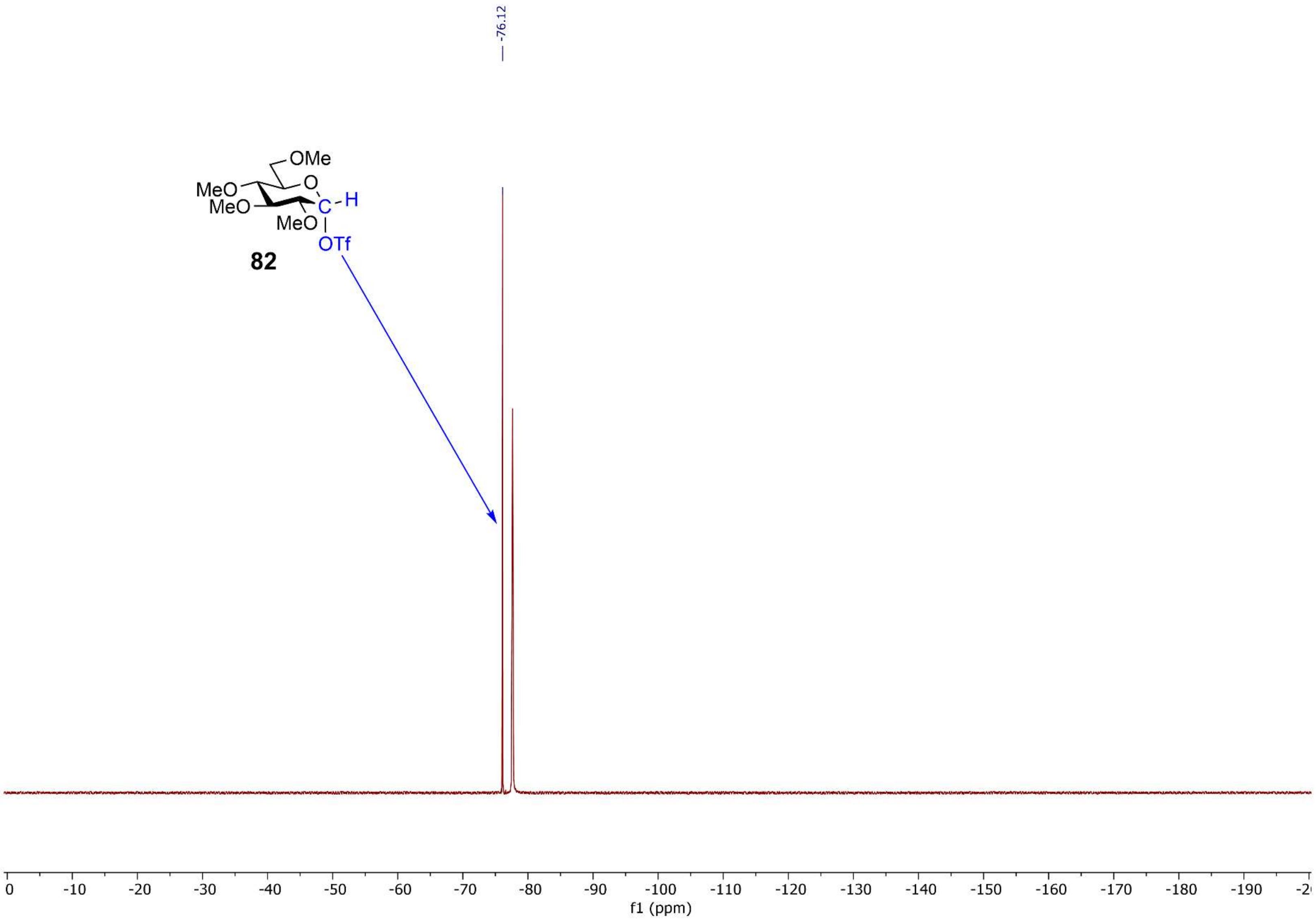

Stacked ^{1}H NMR (500 MHz, CD_2Cl_2) spectra from VT NMR experiment with 5-thioglucosyl trichloroacetimidate 64:

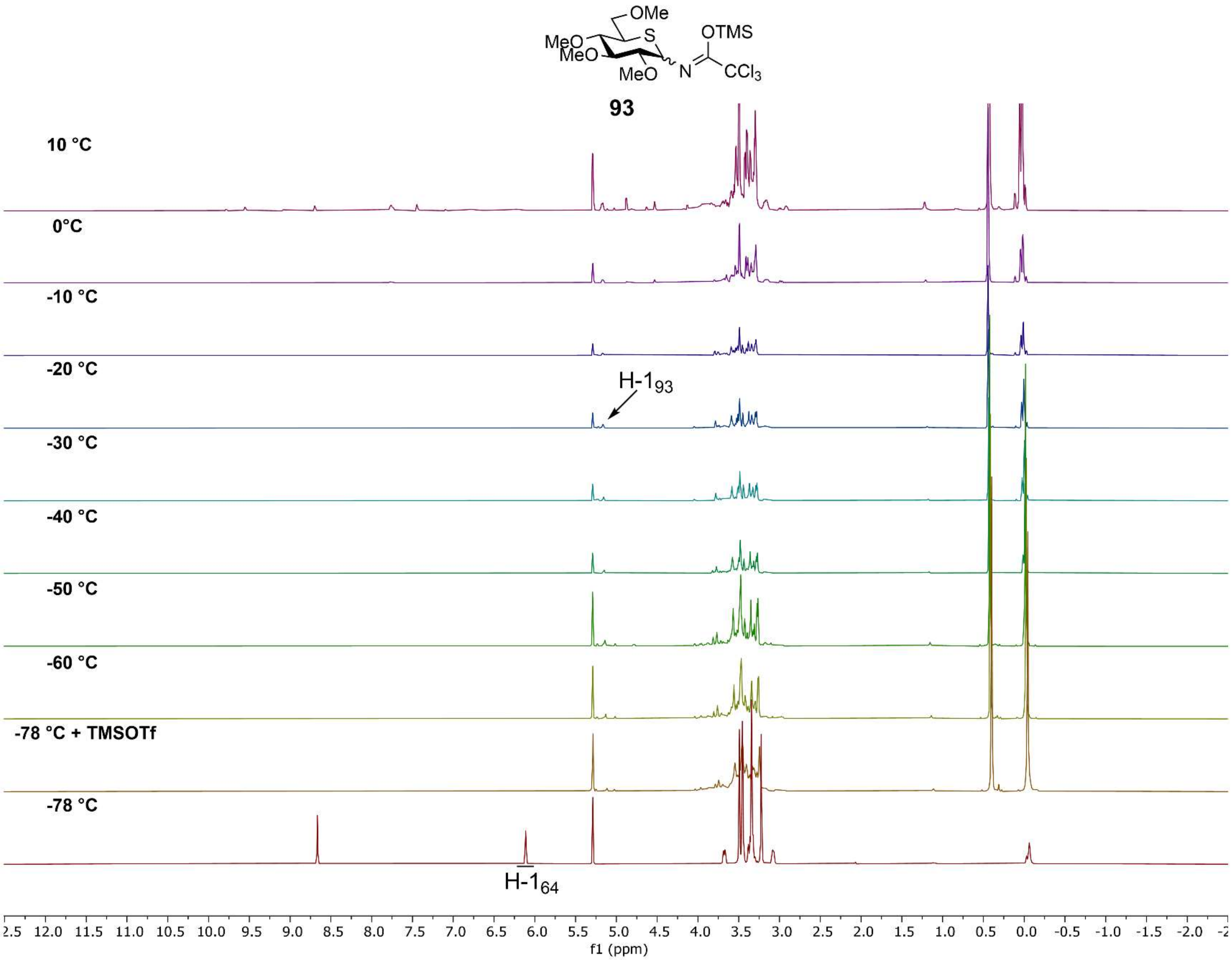

Stacked ^{19}F NMR (470 MHz, CD_2Cl_2) spectra from VT NMR experiment with 5-thioglucosyl trichloroacetimidate 64:

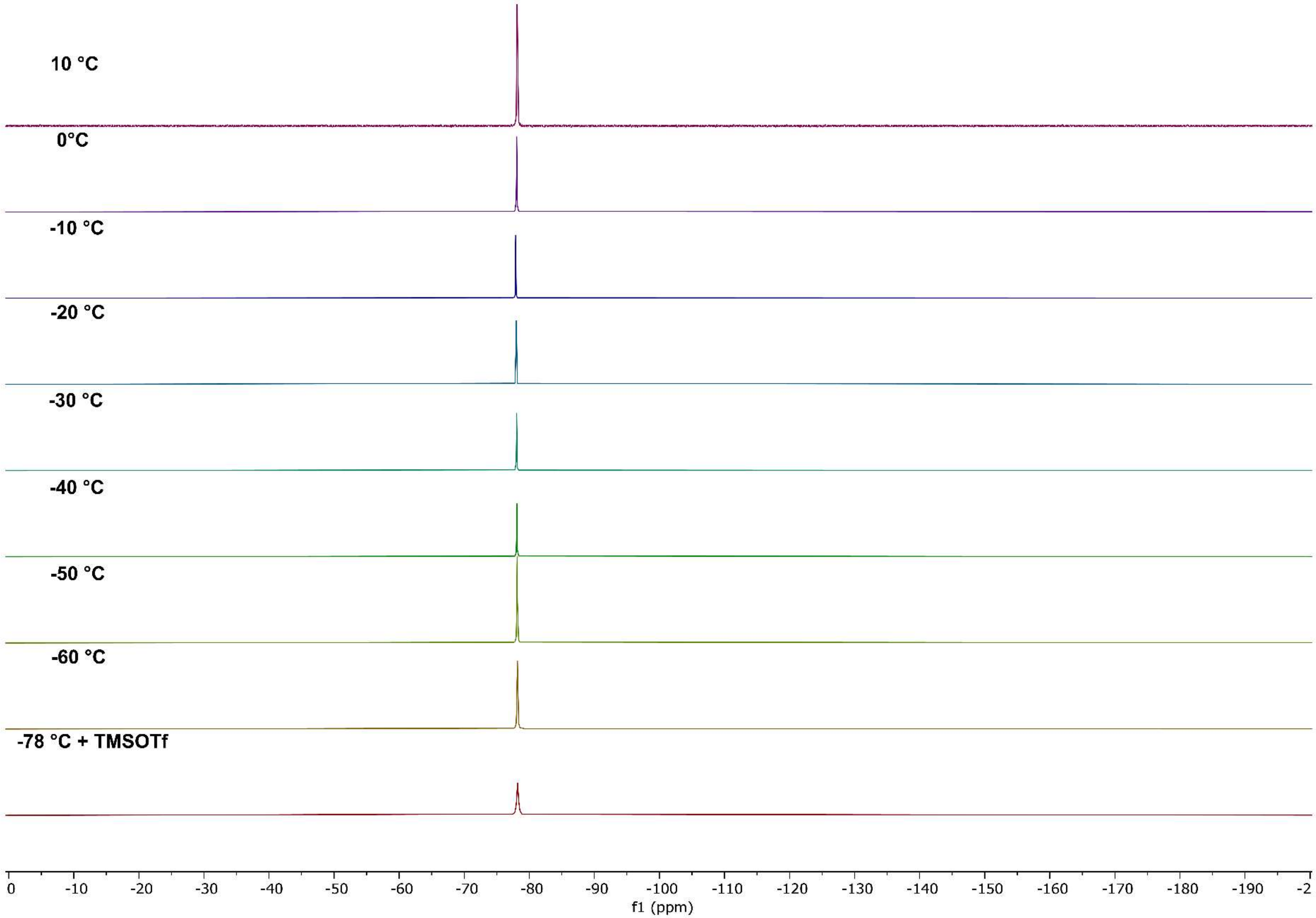

^{1}H NMR (500 MHz, CD_2Cl_2) spectrum of reaction mixture at -50 °C from VT NMR experiment with glucosyl trichloroacetimidate 64:

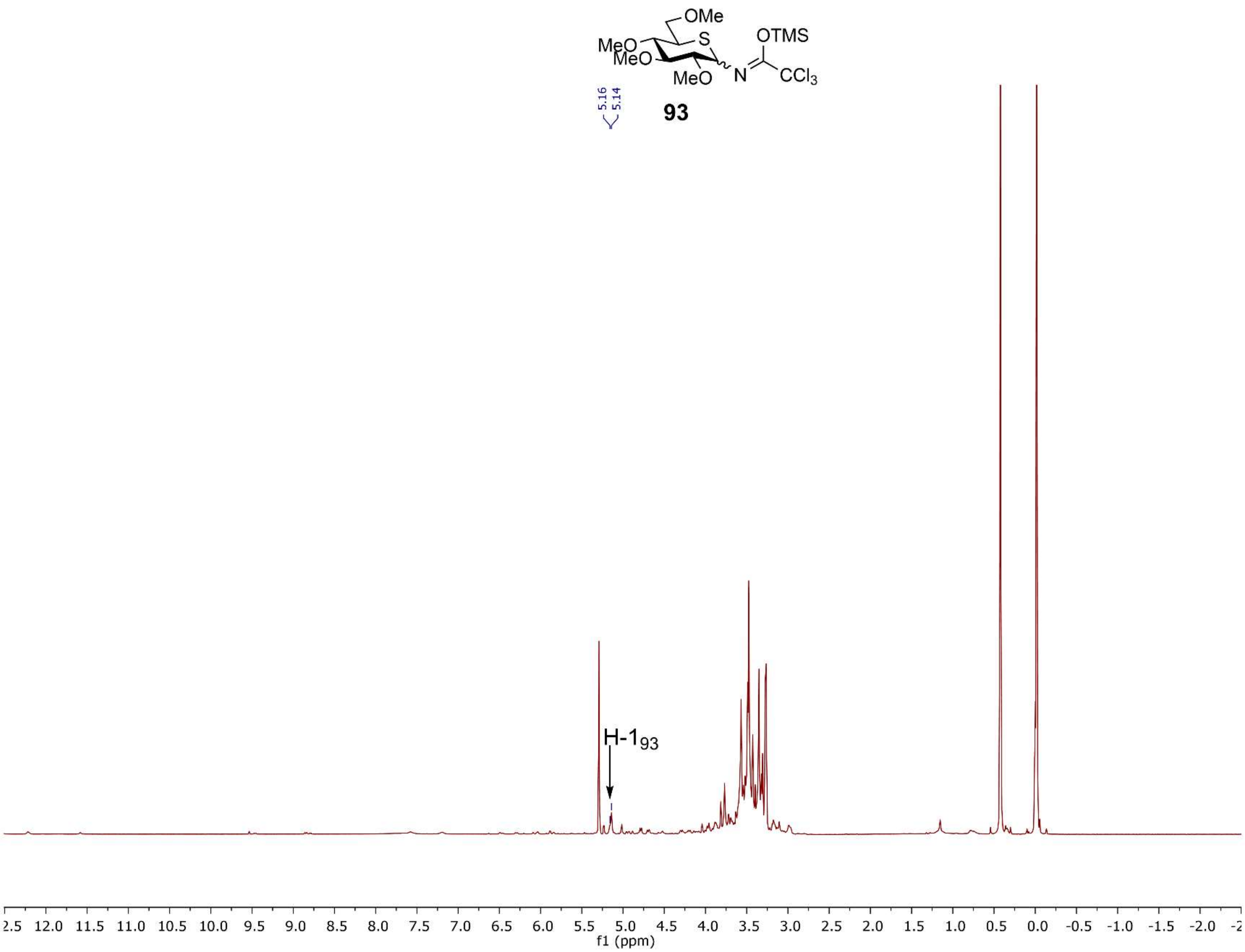

^{13}C NMR (125.67 MHz, CD_2Cl_2) spectrum of reaction mixture at -50 °C from VT NMR experiment with glucosyl trichloroacetimidate 64:

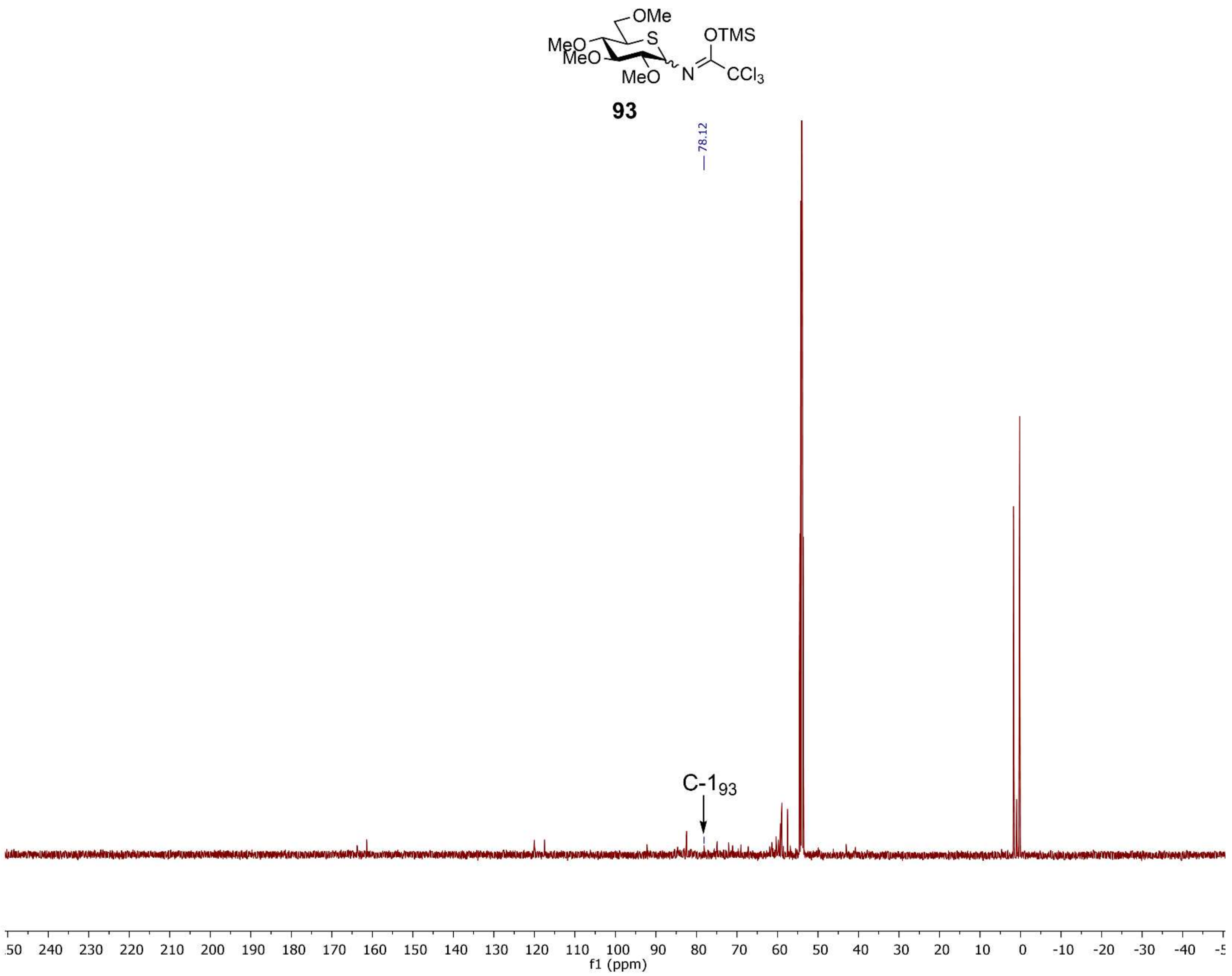

DEPT-90 (CD_2Cl_2) spectrum of reaction mixture at -50 °C from VT NMR experiment with glucosyl trichloroacetimidate 64:

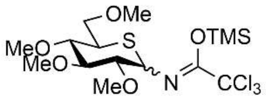

93

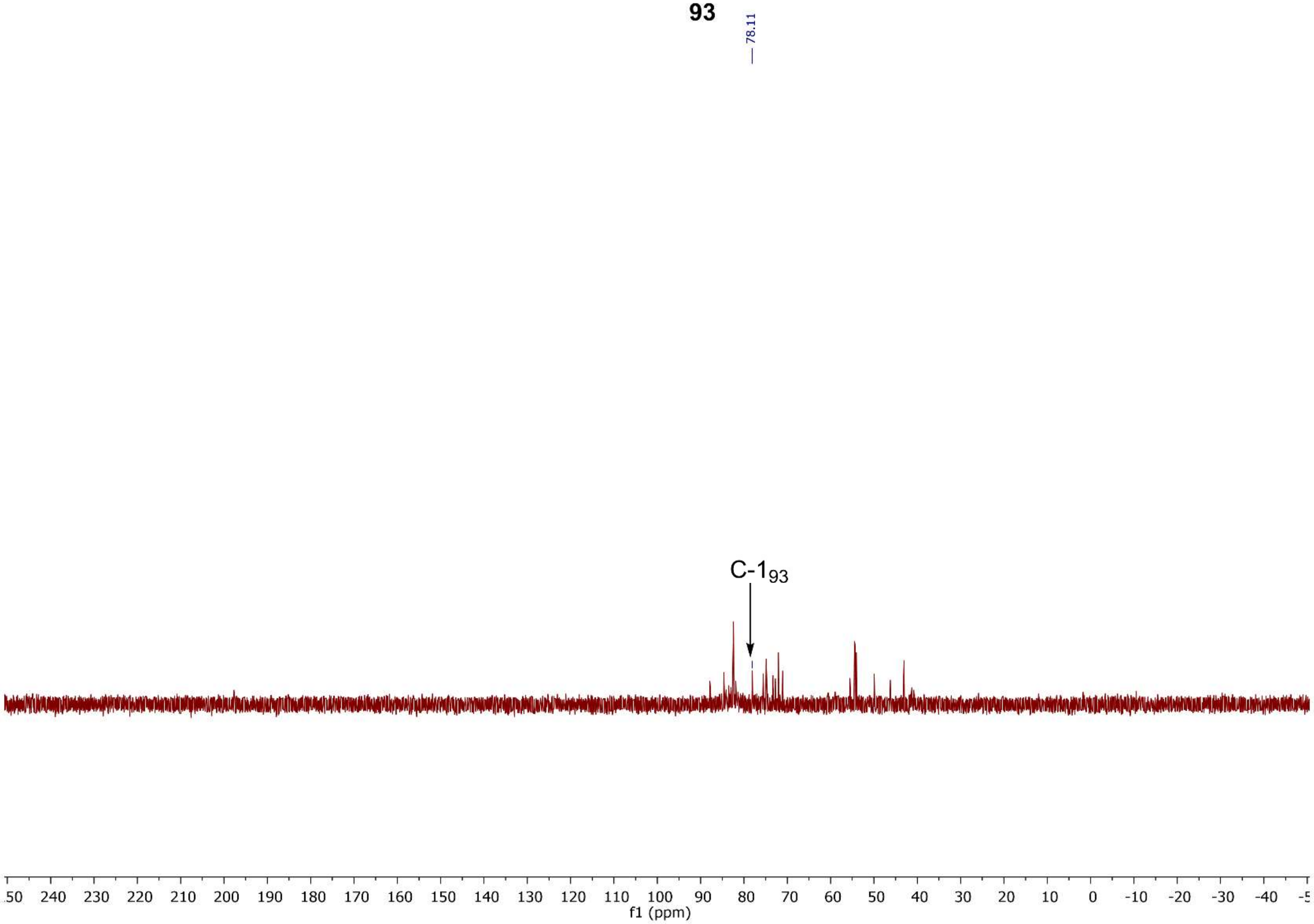

HSQC (CD_2Cl_2) spectrum of reaction mixture at -50 °C from VT NMR experiment with glucosyl trichloroacetimidate 64:

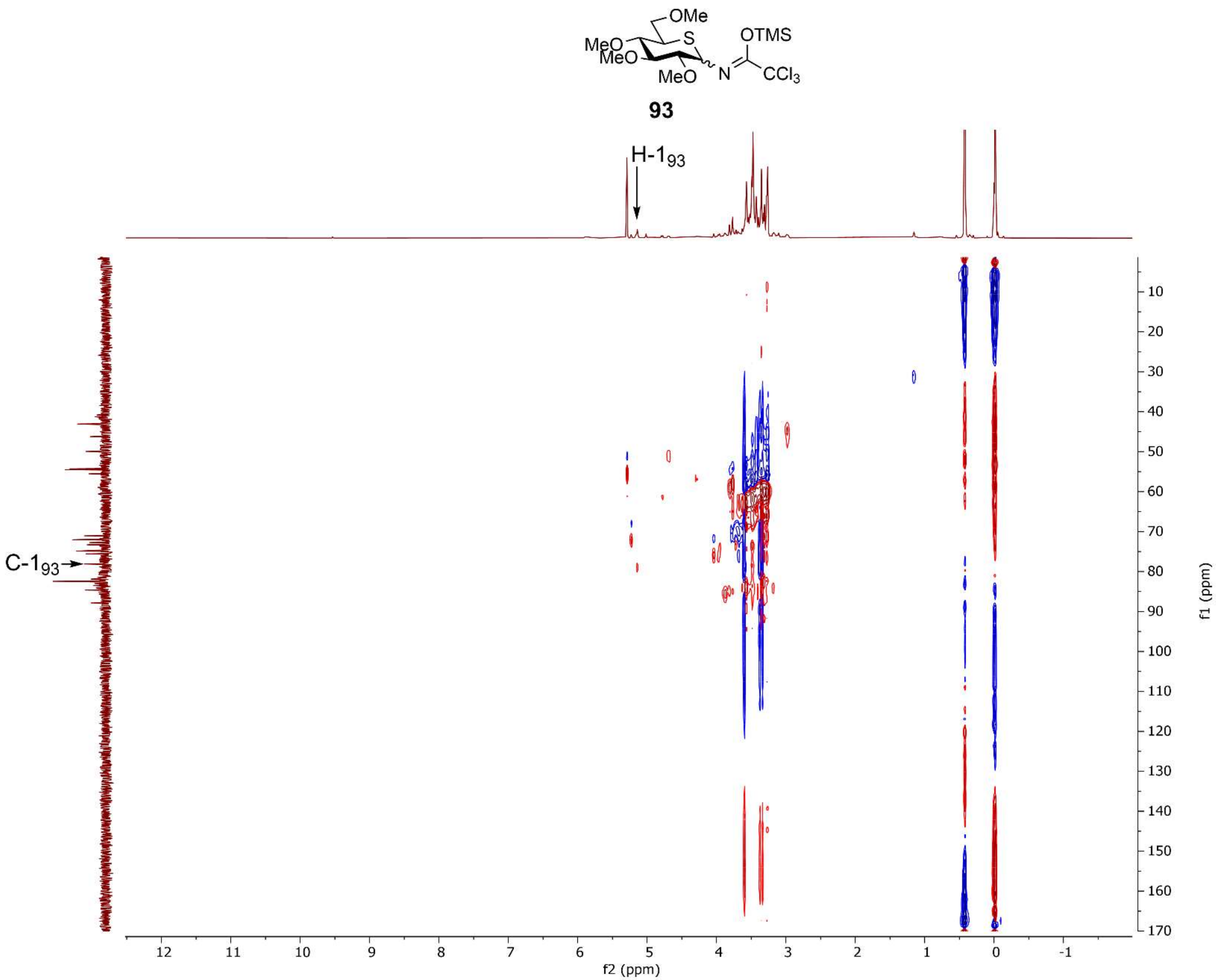

^{19}F NMR (470 MHz, CD_2Cl_2) spectrum of reaction mixture at -50 °C from VT NMR experiment with glucosyl trichloroacetimidate 64:

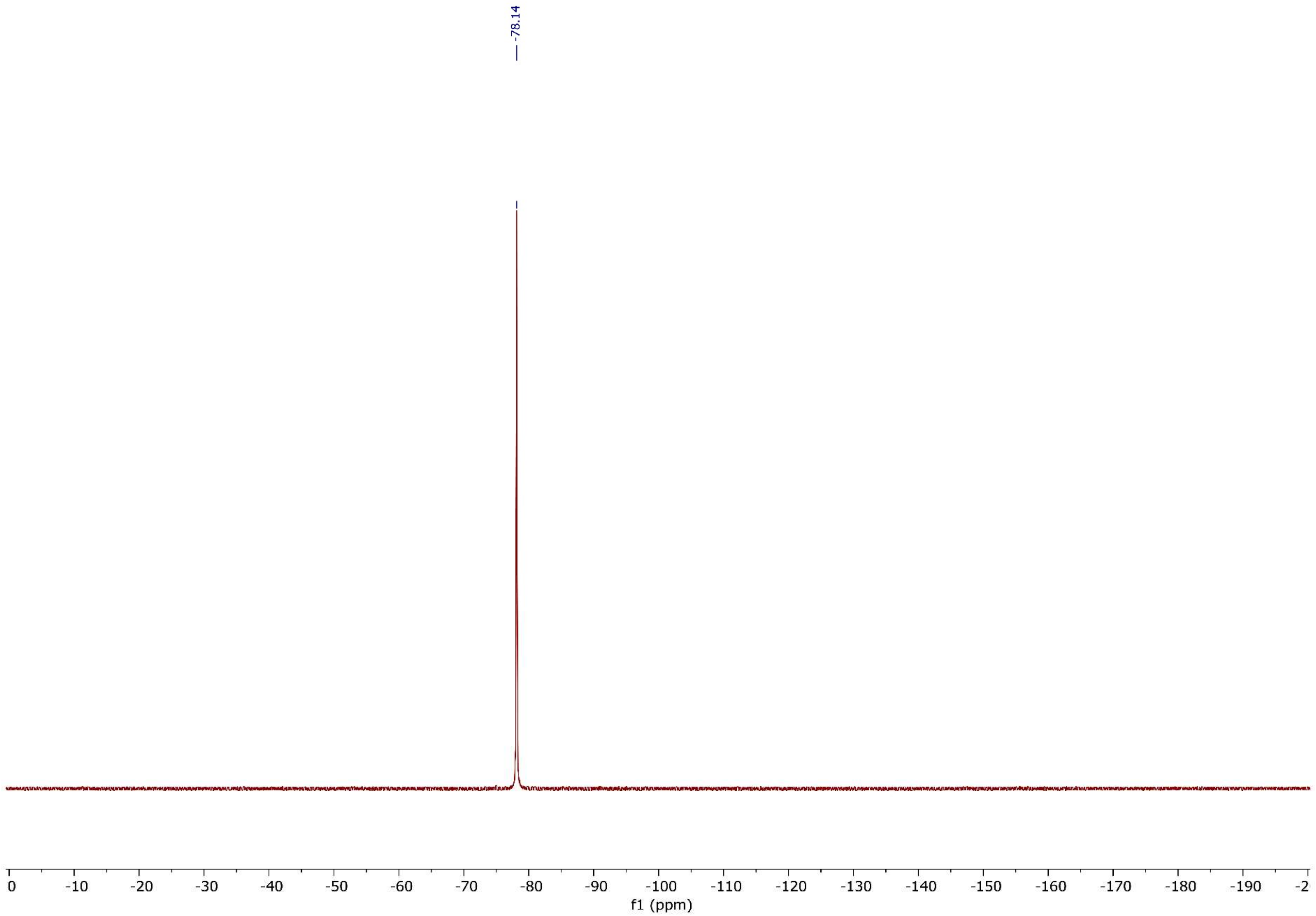

4. NMR spectra of synthesized compounds and isolated decomposition products

^{1}H NMR (500 MHz, $CDCl_3$) spectrum of 2,3-dihydro-4H-thiopyran (**34**):

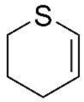

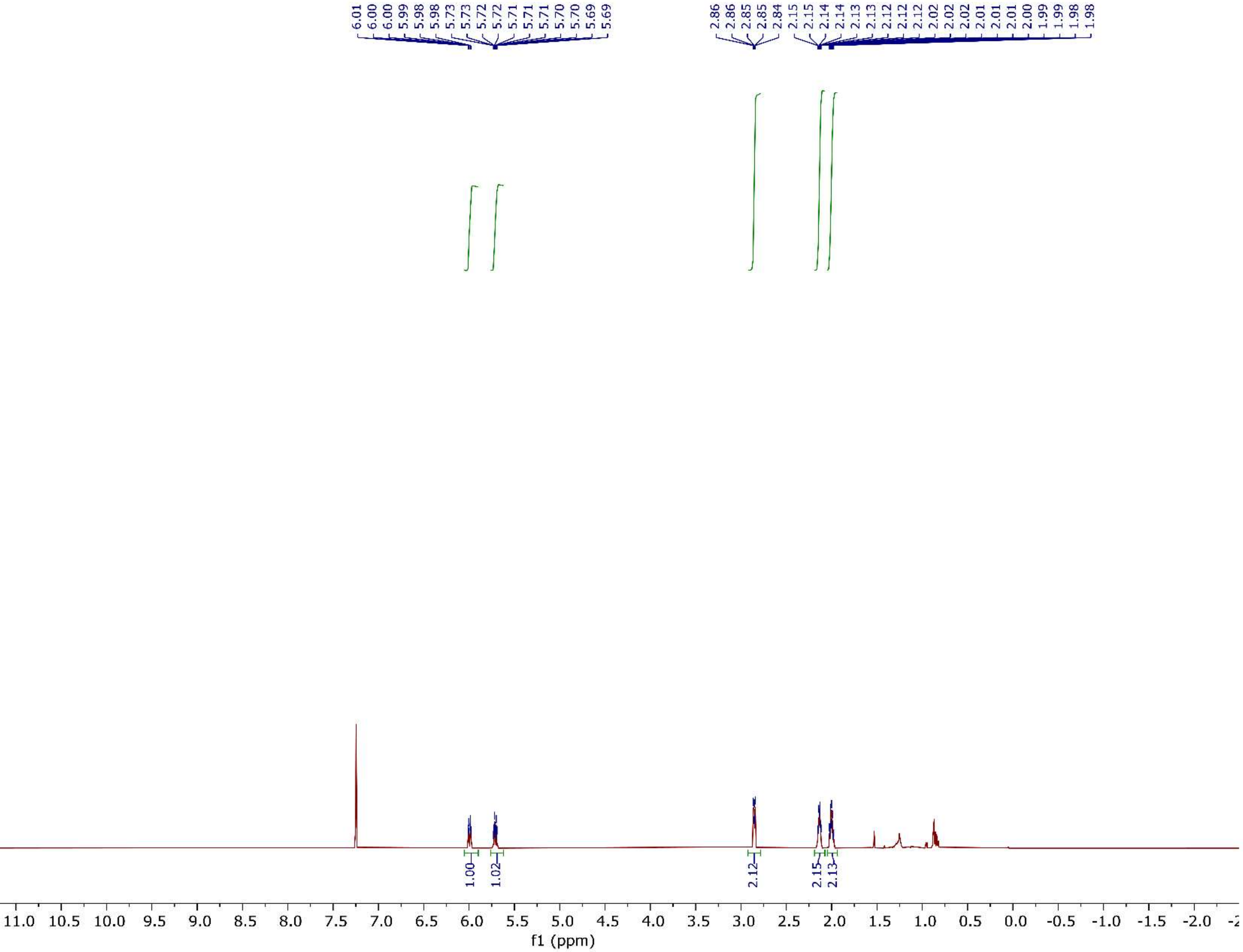

^{13}C NMR (125.67 MHz, $CDCl_3$) spectrum of 2,3-dihydro-4H-thiopyran (**34**):

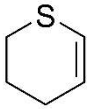

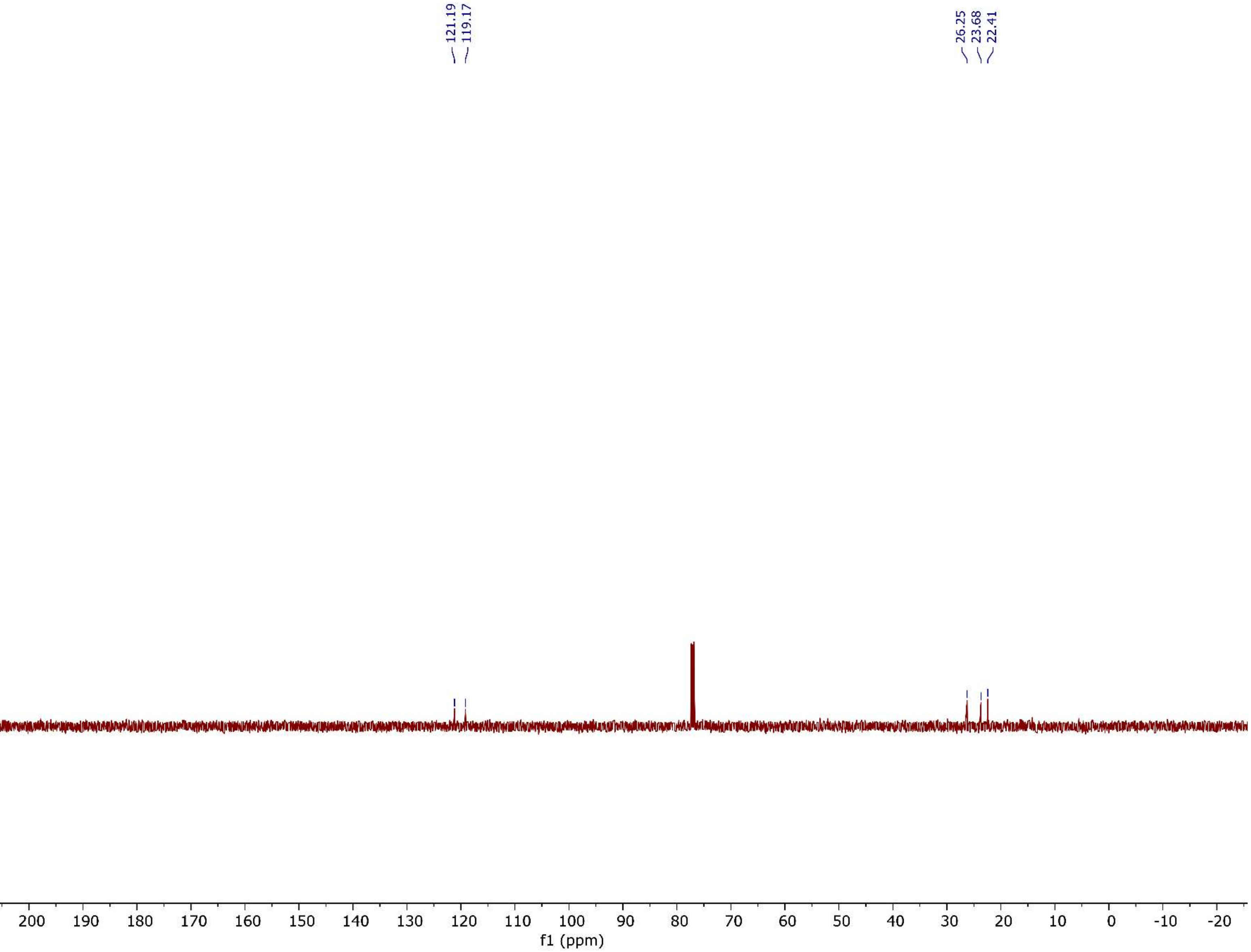

^{1}H NMR (500 MHz, $CDCl_3$) spectrum of (R_S),(S_S)-ethyl 2,3,4,6-tetra-*O*-acetyl-1,5-dithio-*β*-D-glucopyranosyl-1-*S*-oxides (**40**):

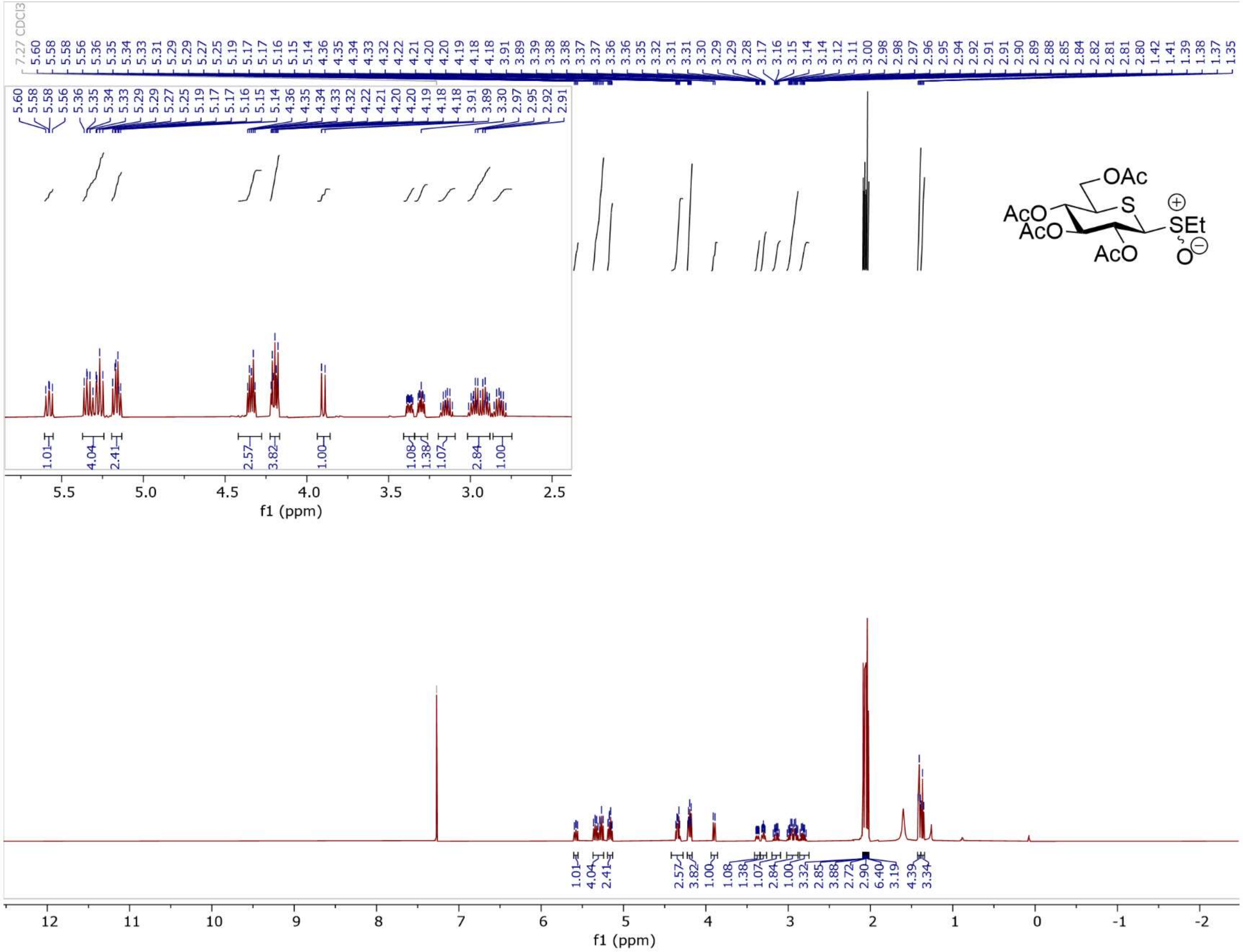

^{13}C NMR (125.67 MHz, $CDCl_3$) spectrum of (R_S),(S_S)-ethyl 2,3,4,6-tetra-*O*-acetyl-1,5-dithio-*β*-D-glucopyranosyl-1-*S*-oxides (**40**):

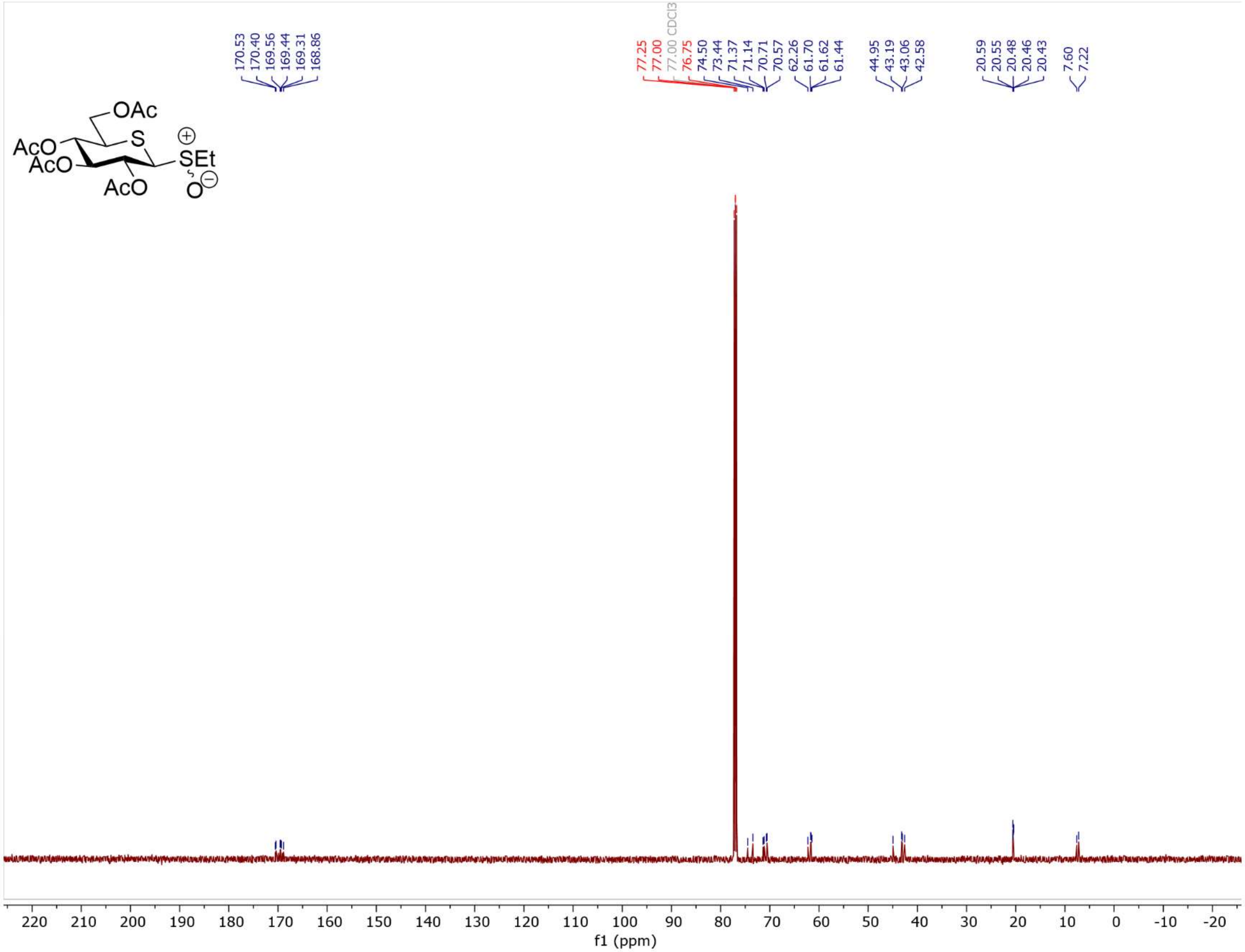

COSY ($CDCl_3$) spectrum of (R_S),(S_S)-ethyl 2,3,4,6-tetra-*O*-acetyl-1,5-dithio-*β*-D-glucopyranosyl-1-*S*-oxides (**40**):

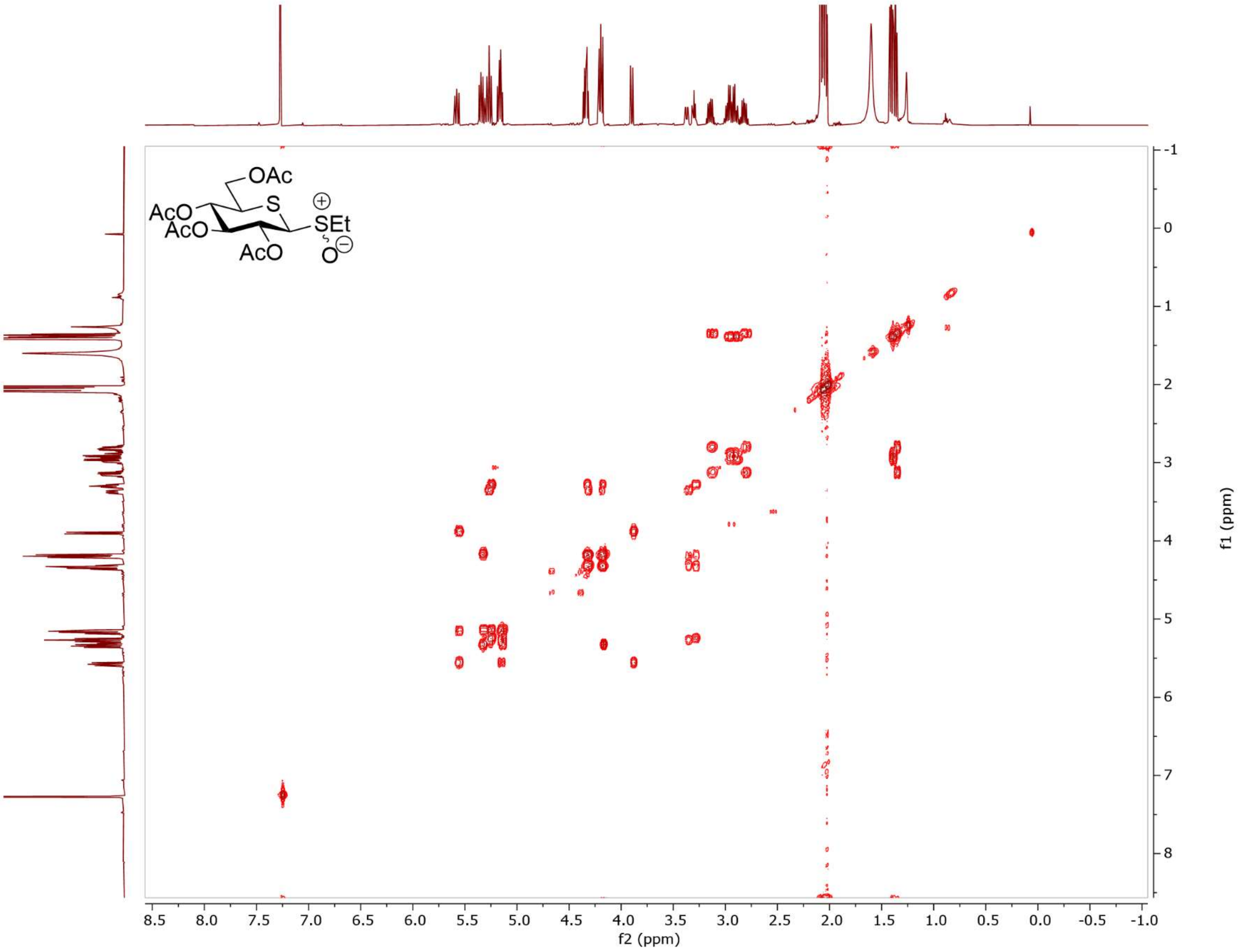

HSQC ($CDCl_3$) spectrum of (R_S),(S_S)-ethyl 2,3,4,6-tetra-*O*-acetyl-1,5-dithio-*β*-D-glucopyranosyl-1-*S*-oxides (**40**):

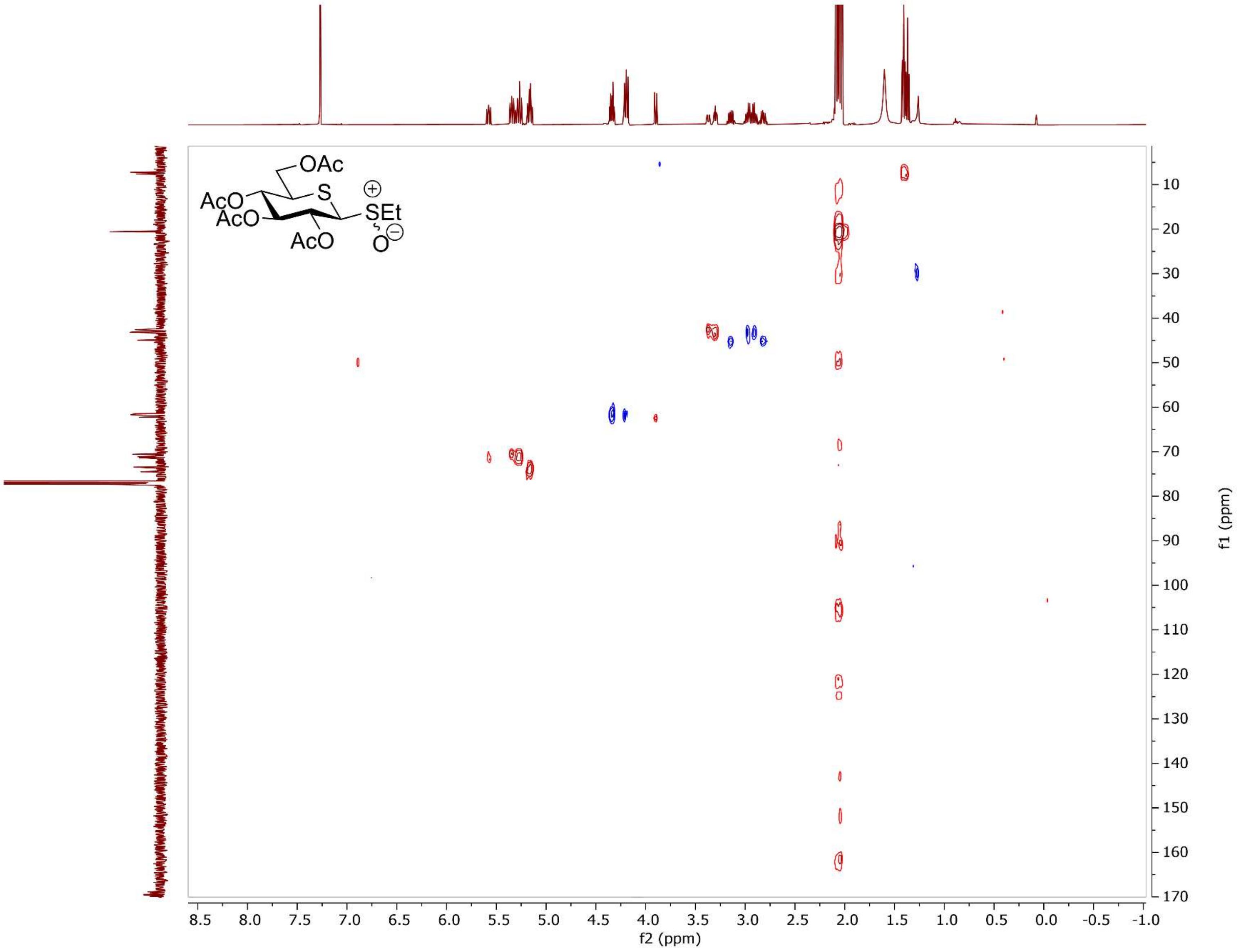

^{1}H NMR (500 MHz, $CDCl_3$) spectrum of (R_S),(S_S)-ethyl 2,3,4,6-tetra-*O*-acetyl-1,5-dithio-*β*-D-glucopyranosyl-5-*S*-oxides (**41**):

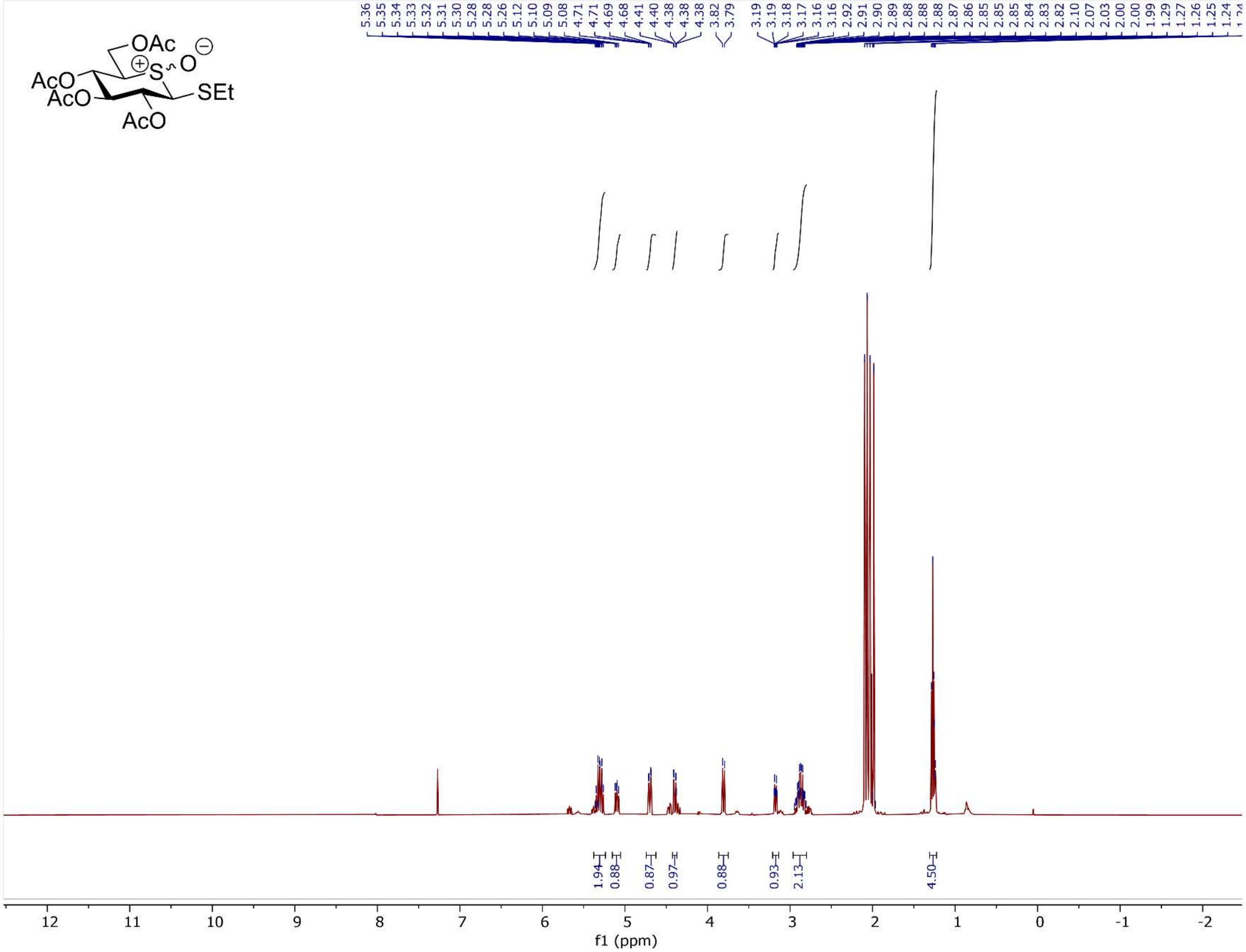

^{13}C NMR (125.67 MHz, $CDCl_3$) spectrum of (R_S),(S_S)-ethyl 2,3,4,6-tetra-*O*-acetyl-1,5-dithio-*β*-D-glucopyranosyl-5-*S*-oxides (**41**):

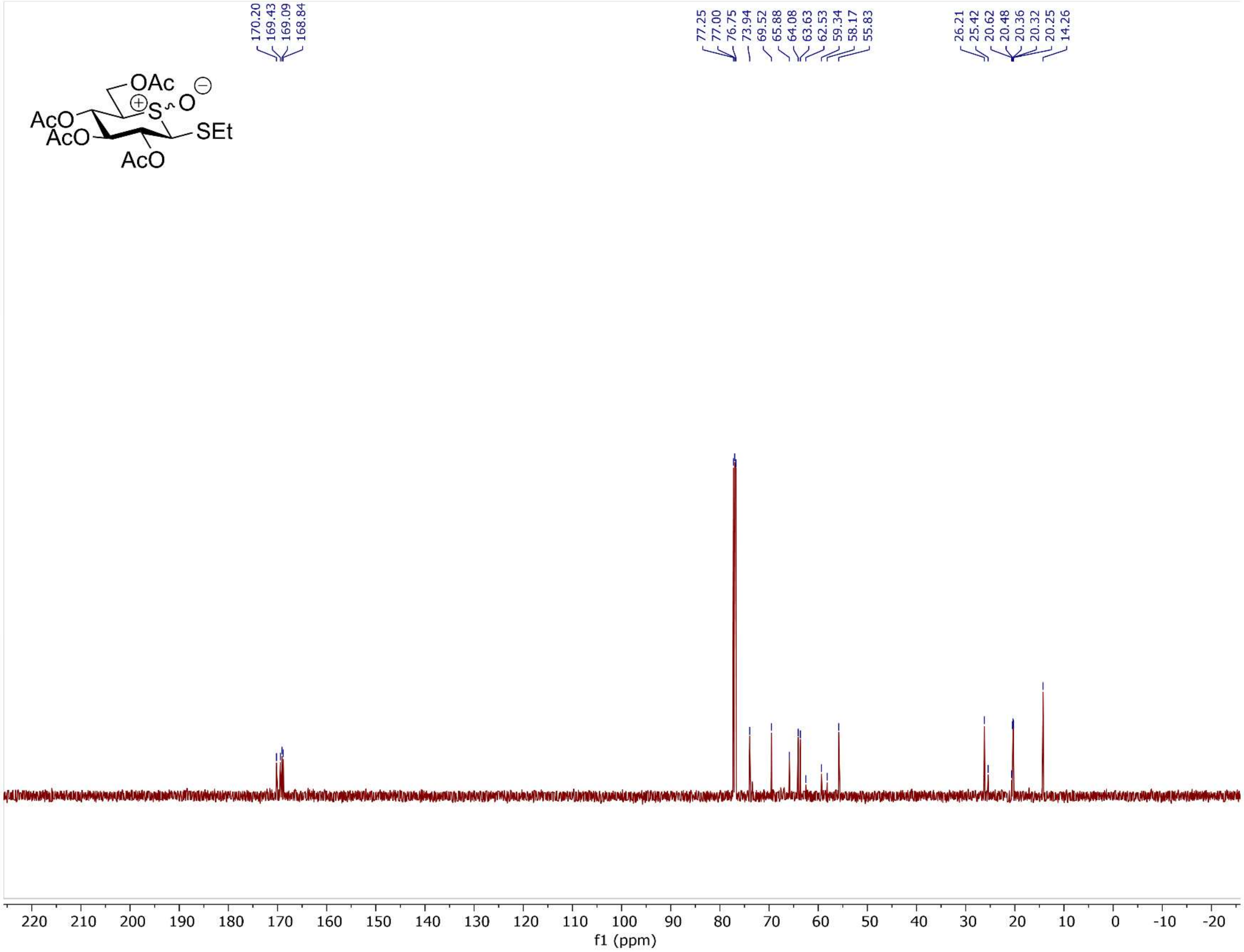

COSY ($CDCl_3$) spectrum of (R_S),(S_S)-ethyl 2,3,4,6-tetra-*O*-acetyl-1,5-dithio-*β*-D-glucopyranosyl-5-*S*-oxides (**41**):

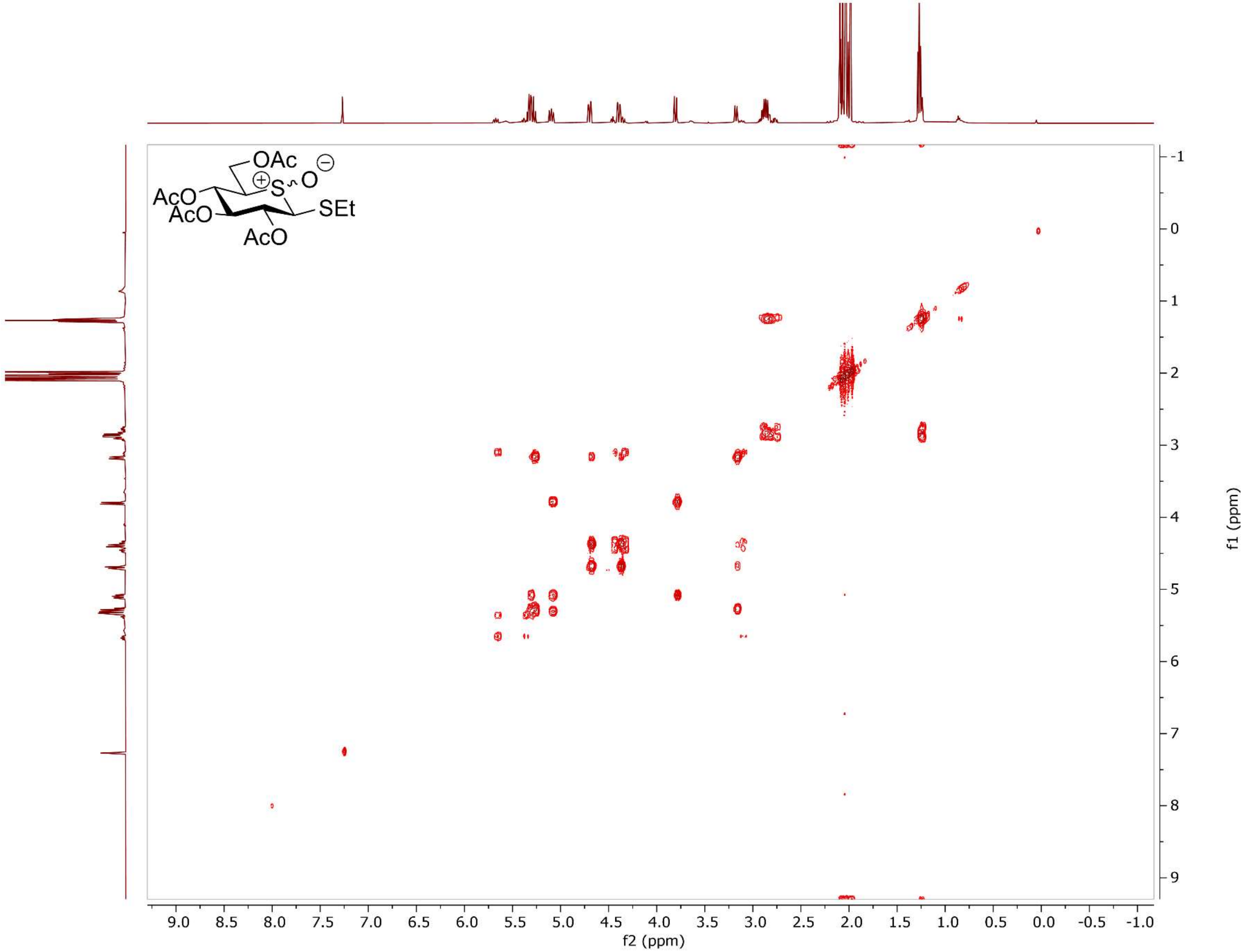

HMQC ($CDCl_3$) spectrum of (*R*S),(*S*S)-ethyl 2,3,4,6-tetra-*O*-acetyl-1,5-dithio-*β*-D-glucopyranosyl-5-*S*-oxides (**41**):

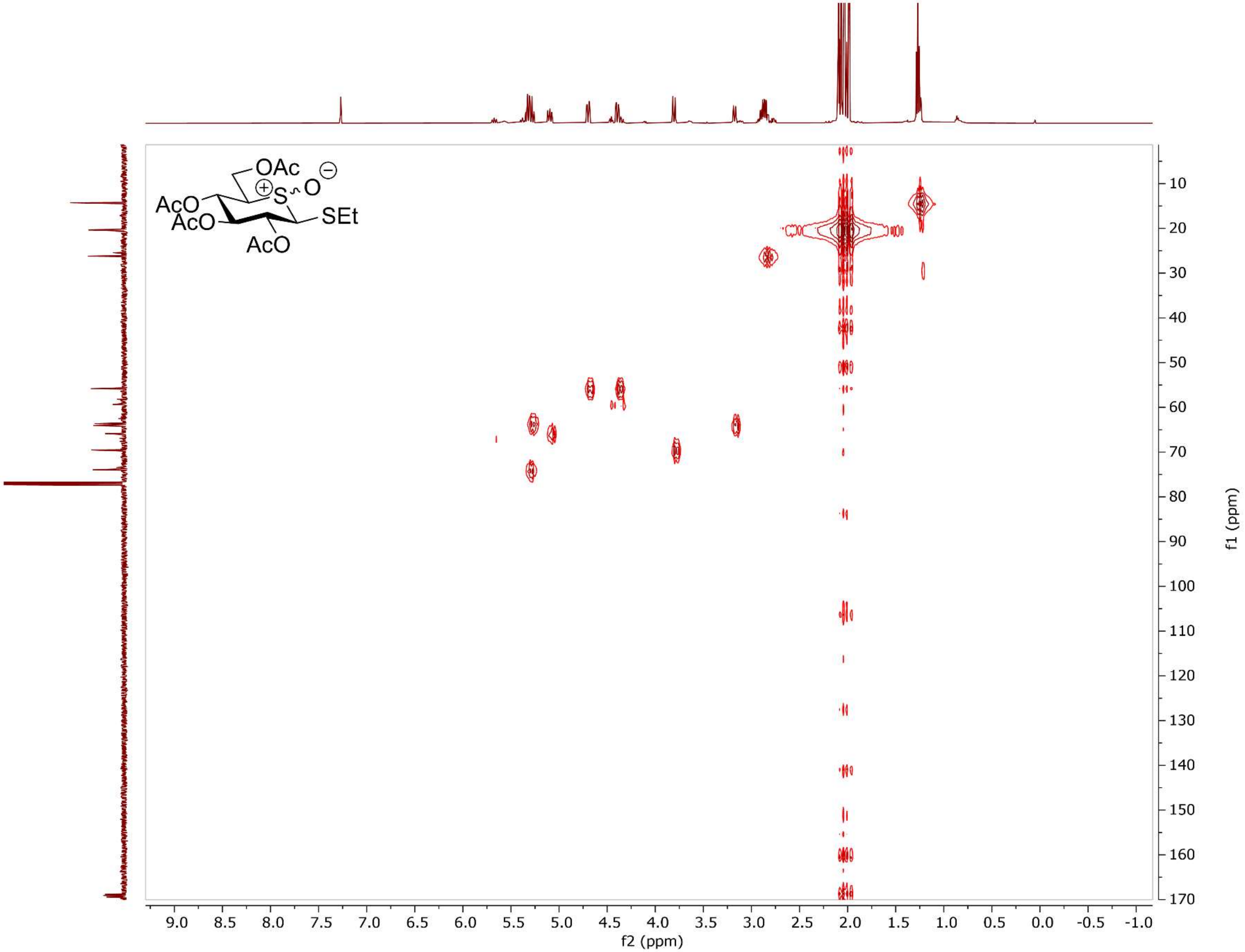

¹H NMR (500 MHz, $CDCl_3$) spectrum of ethyl 2,3,4,6-tetra-*O*-acetyl-1,5-di-thio-*α*-D-glucopyranosyl-1-*S*-Oxide (**42**):

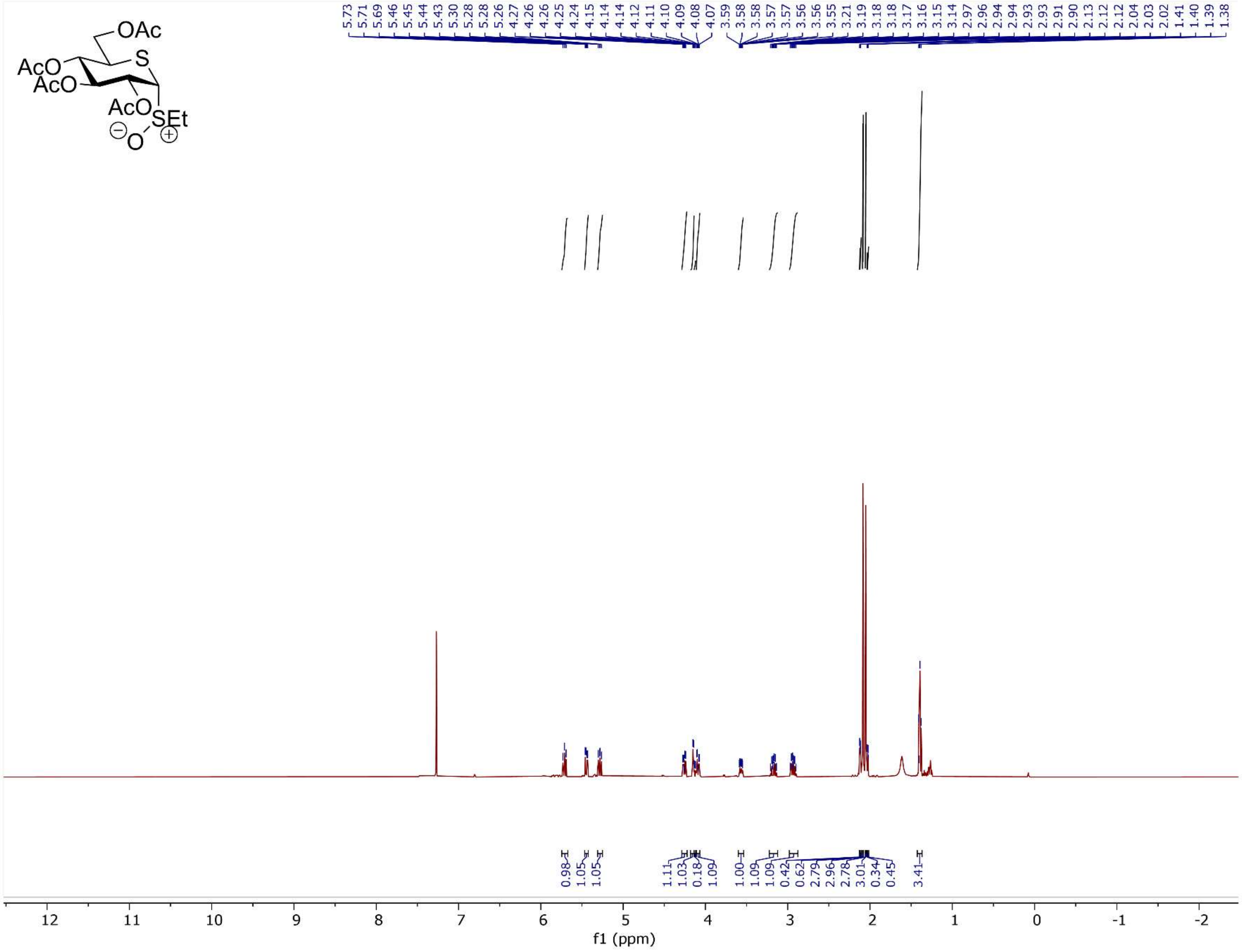

^{13}C NMR (125.67 MHz, $CDCl_3$) spectrum of ethyl 2,3,4,6-tetra-*O*-acetyl-1,5-di-thio-*α*-D-glucopyranosyl-1-*S*-Oxide (**42**):

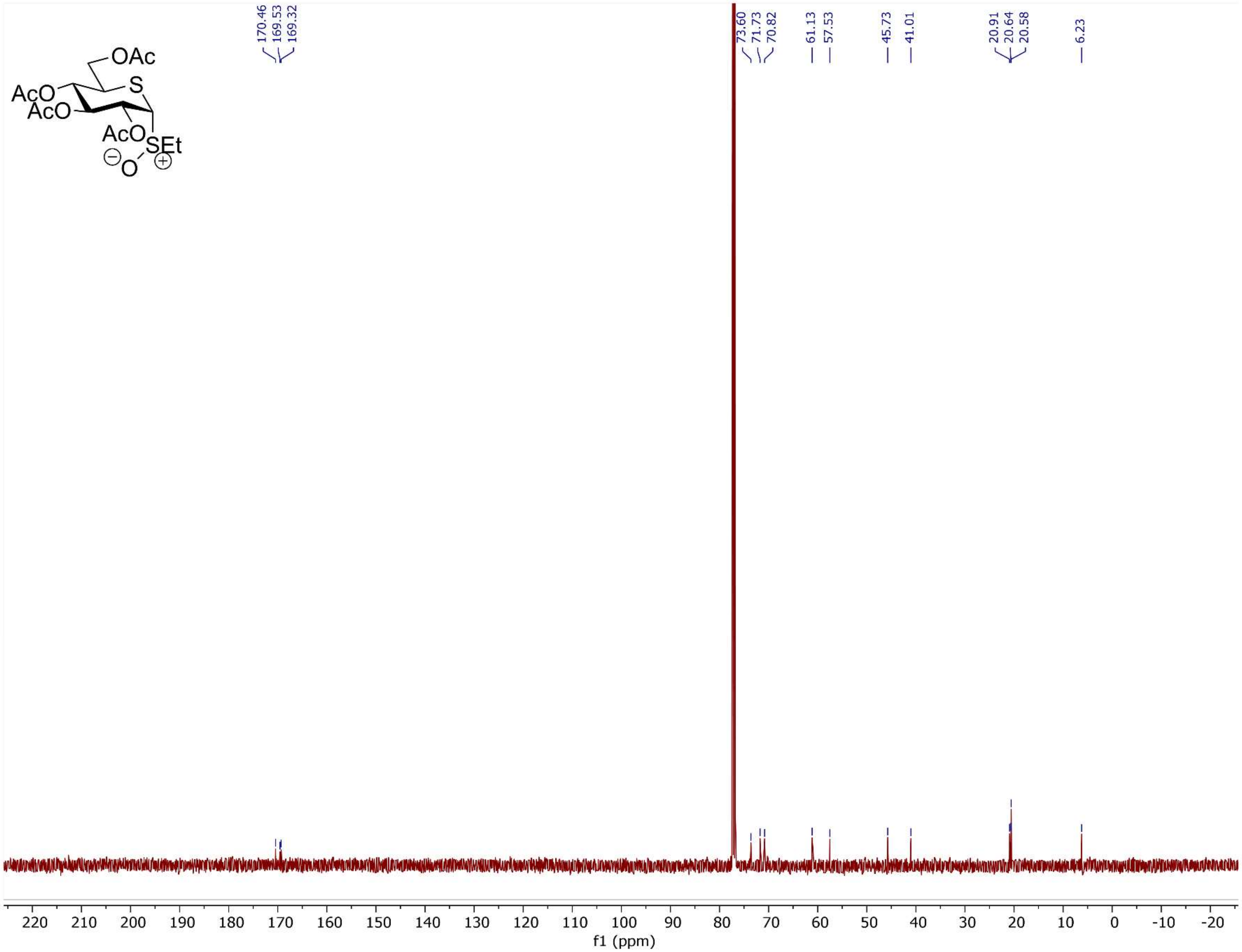

COSY ($CDCl_3$) spectrum of ethyl 2,3,4,6-tetra-*O*-acetyl-1,5-di-thio-*α*-D-glucopyranosyl-1-*S*-Oxide (**42**):

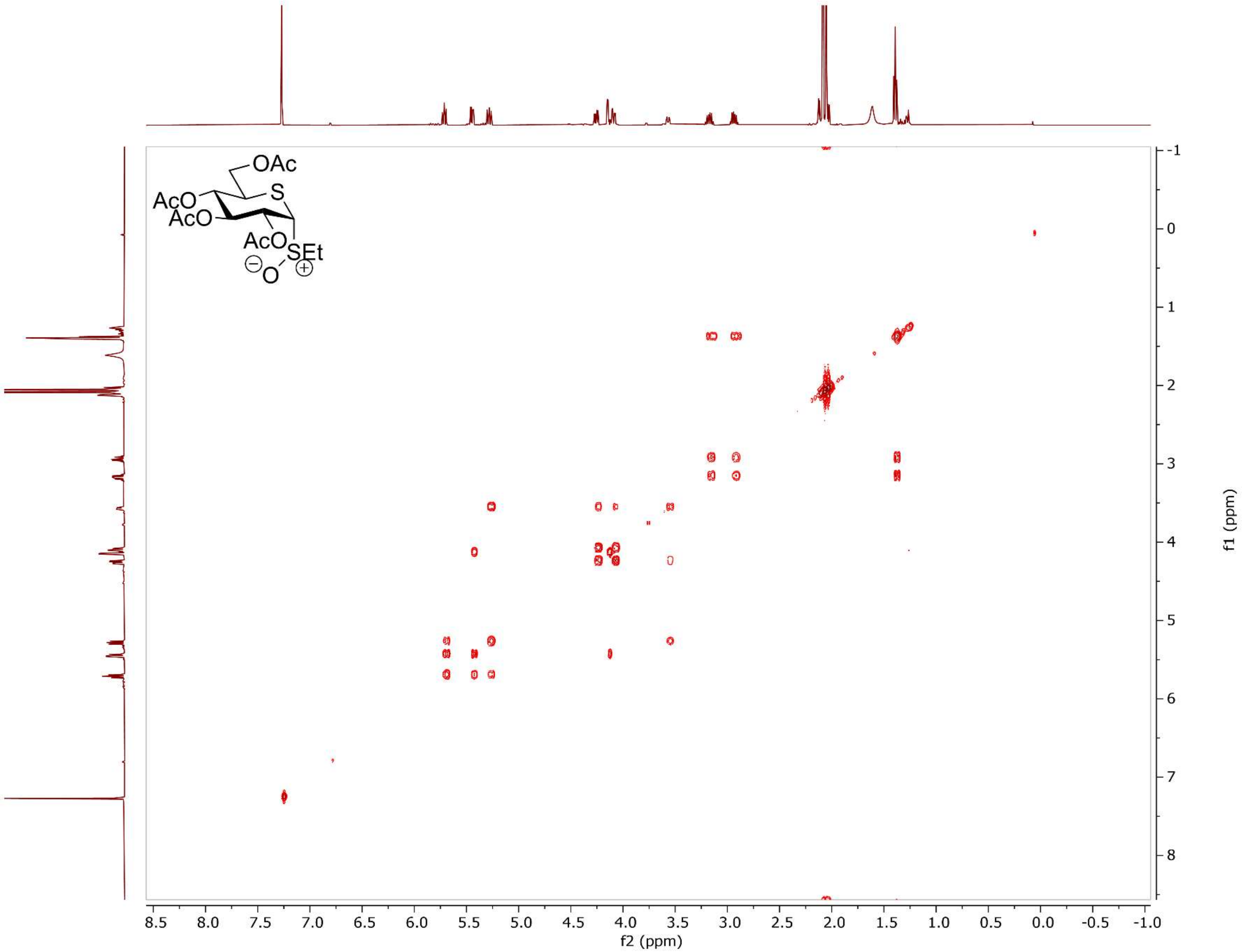

HSQC ($CDCl_3$) spectrum of ethyl 2,3,4,6-tetra-*O*-acetyl-1,5-di-thio-*α*-D-glucopyranosyl-1-*S*-Oxide (**42**):

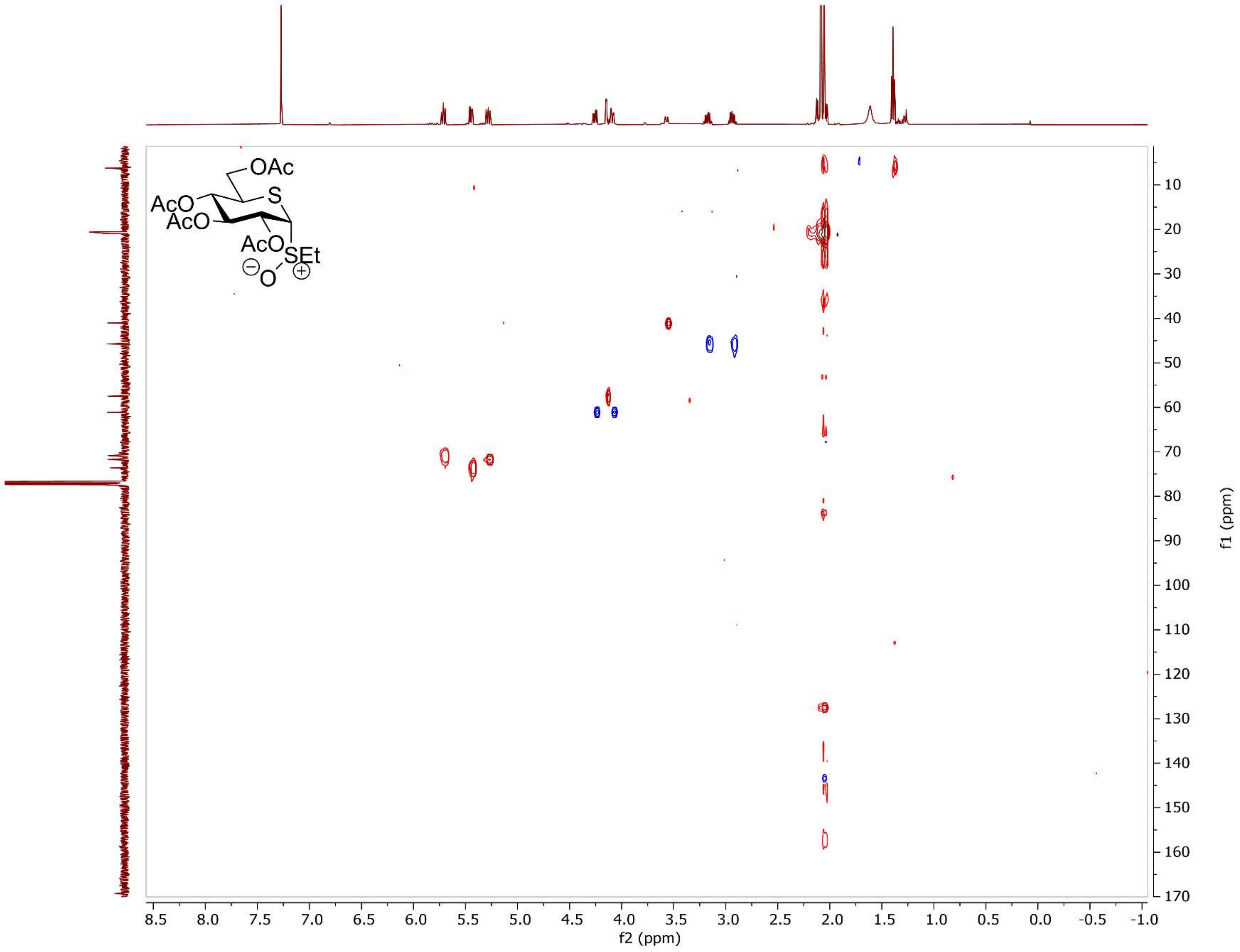

^{1}H NMR (500 MHz, $CDCl_3$) spectrum of ethyl 2,3,4,6-tetra-*O*-methyl-1,5-di-thio-*β*-D-glucopyranoside (**44**):

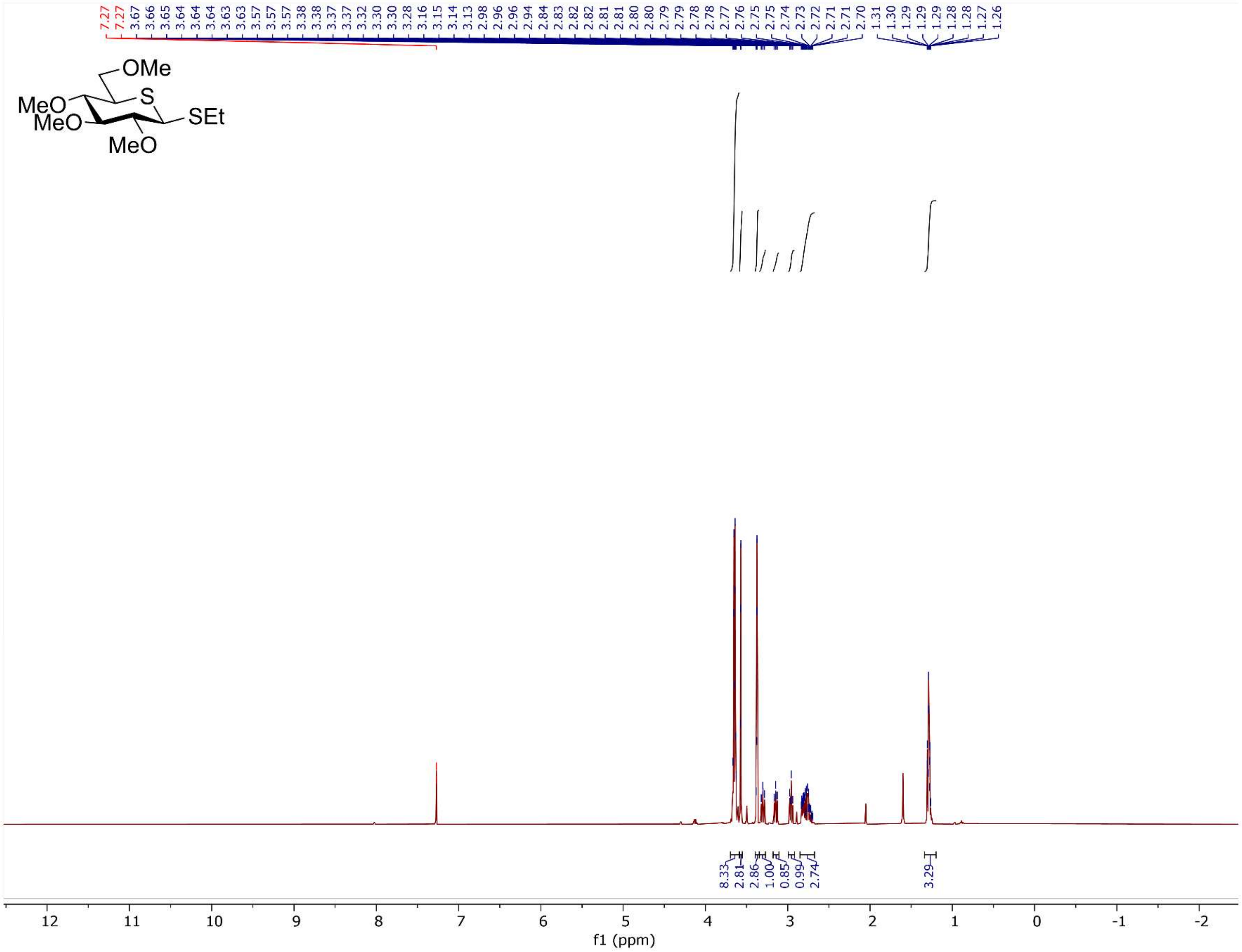

^{13}C NMR (125.67 MHz, $CDCl_3$) spectrum of ethyl 2,3,4,6-tetra-*O*-methyl-1,5-di-thio-*β*-D-glucopyranoside (**44**):

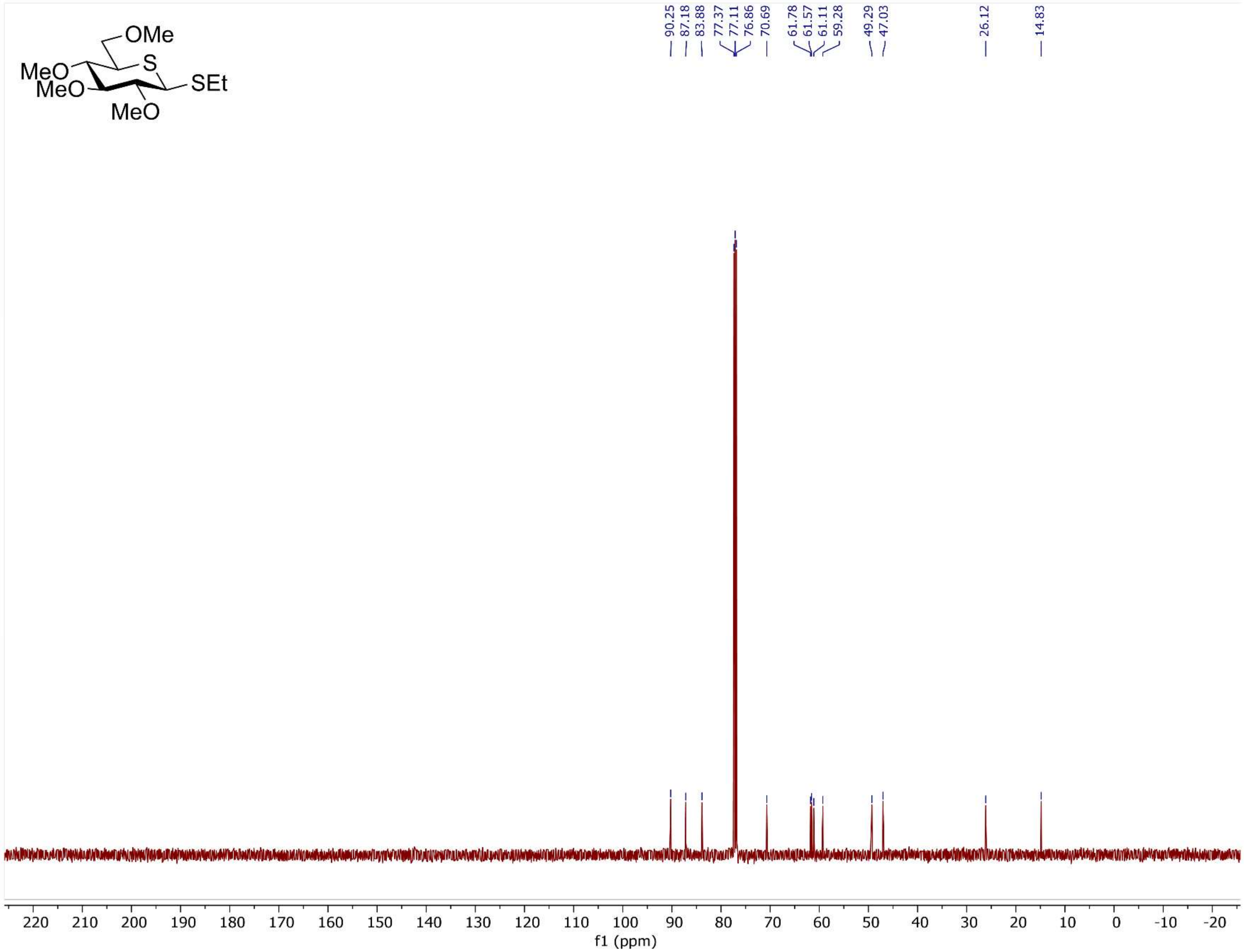

COSY ($CDCl_3$) spectrum of ethyl 2,3,4,6-tetra-*O*-methyl-1,5-di-thio-*β*-D-glucopyranoside (**44**):

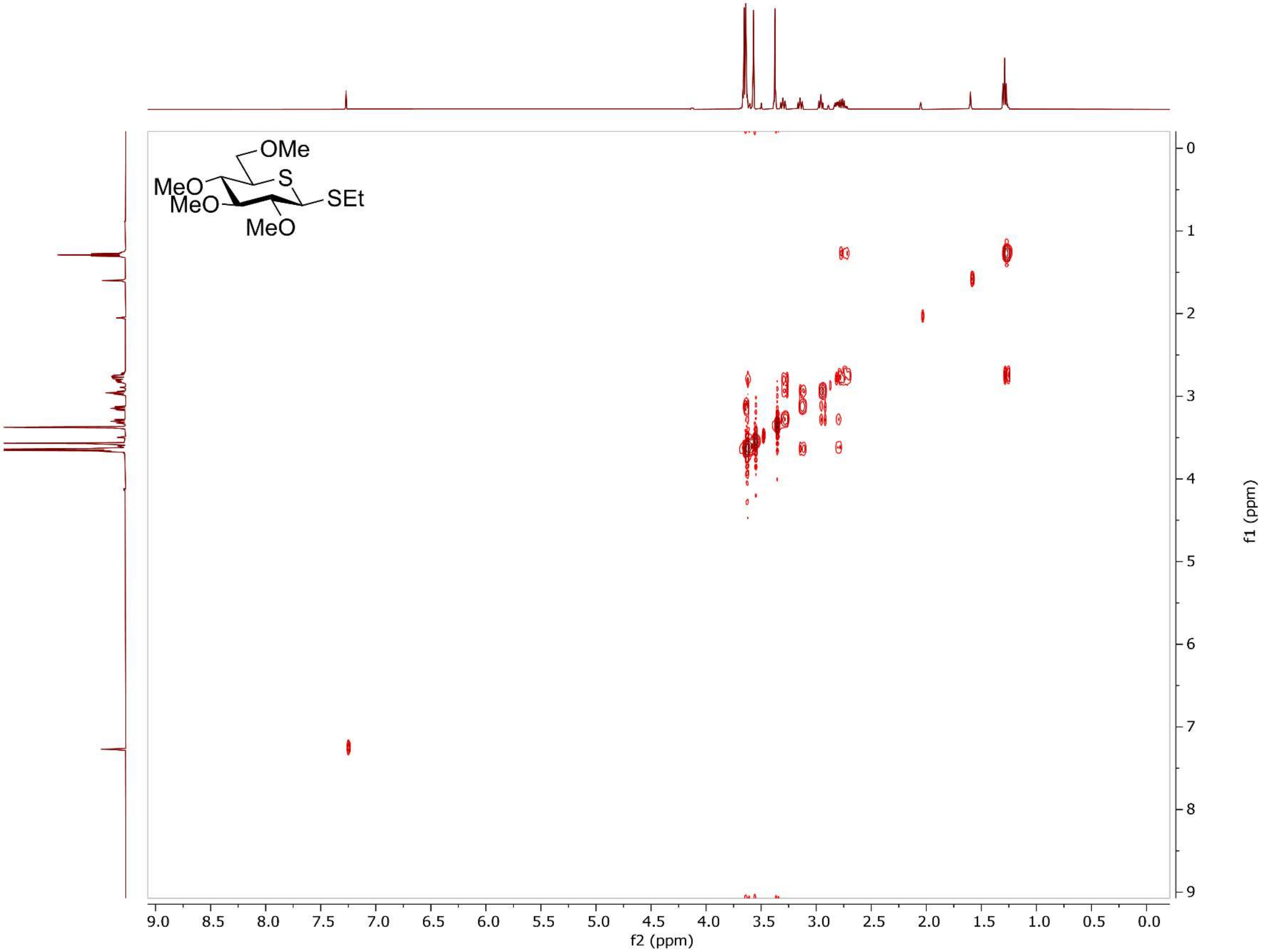

HSQC (CDCl₃) spectrum of ethyl 2,3,4,6-tetra-*O*-methyl-1,5-di-thio-*β*-D-glucopyranoside (**44**):

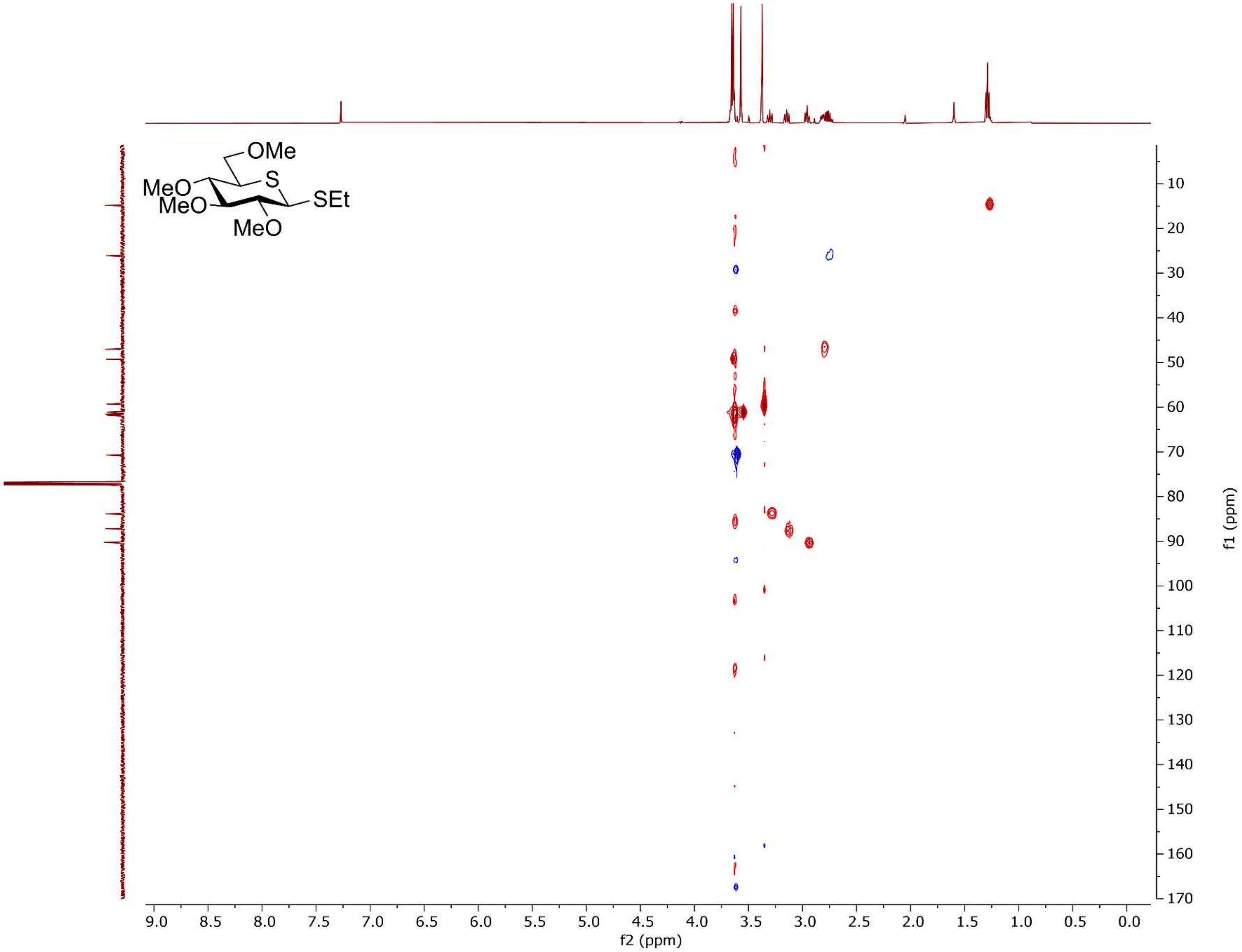

^{1}H NMR (500 MHz, CD_2Cl_2) spectrum of (R_S),(S_S)-ethyl 2,3,4,6-tetra-*O*-methyl-1,5-dithio-*β*-D-glucopyranoside-1-*S*-Oxides (**45**):

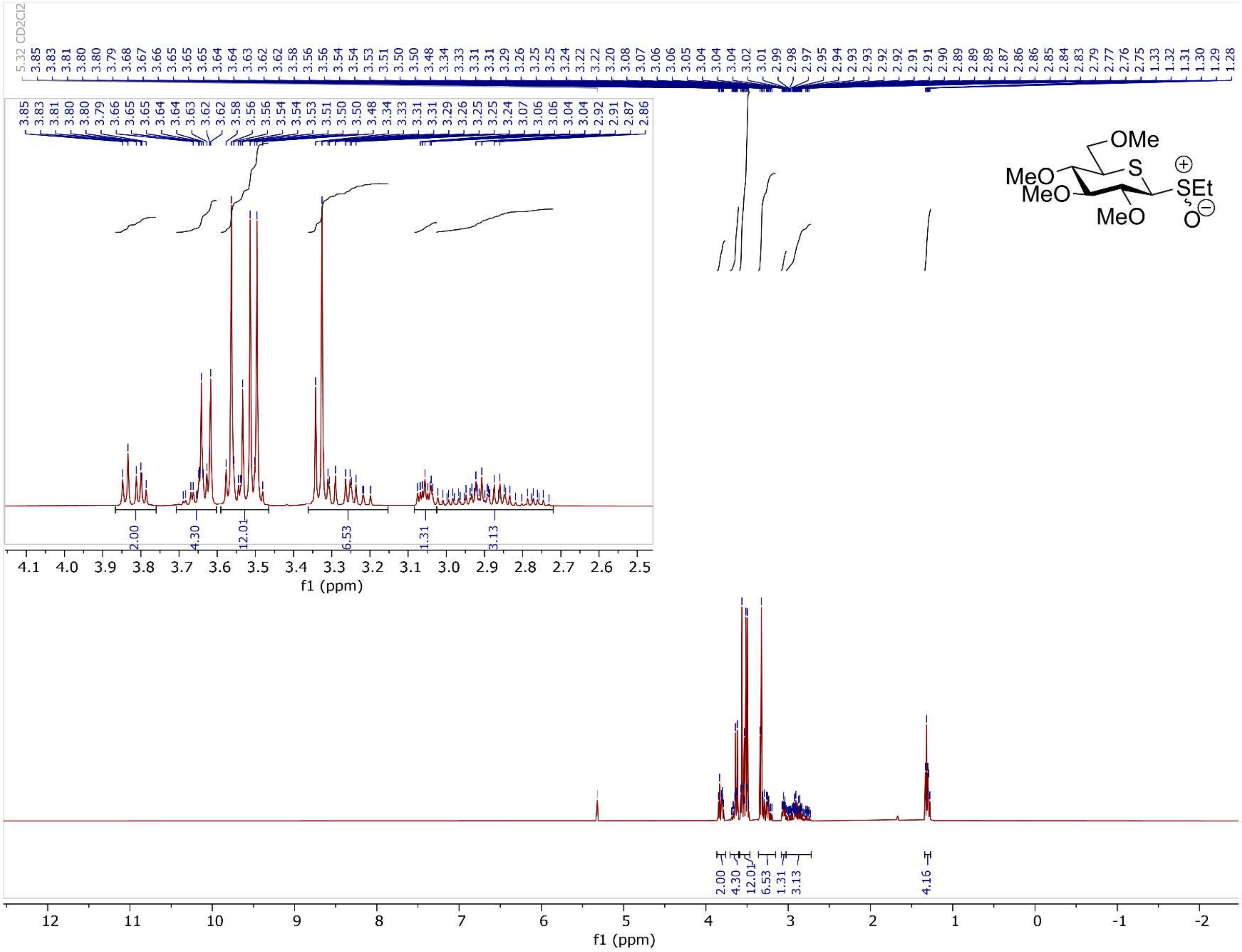

^{13}C NMR (125.67 MHz, CD_2Cl_2) spectrum of (R_S),(S_S)-ethyl 2,3,4,6-tetra-*O*-methyl-1,5-dithio-*β*-D-glucopyranoside-1-*S*-Oxides (**45**):

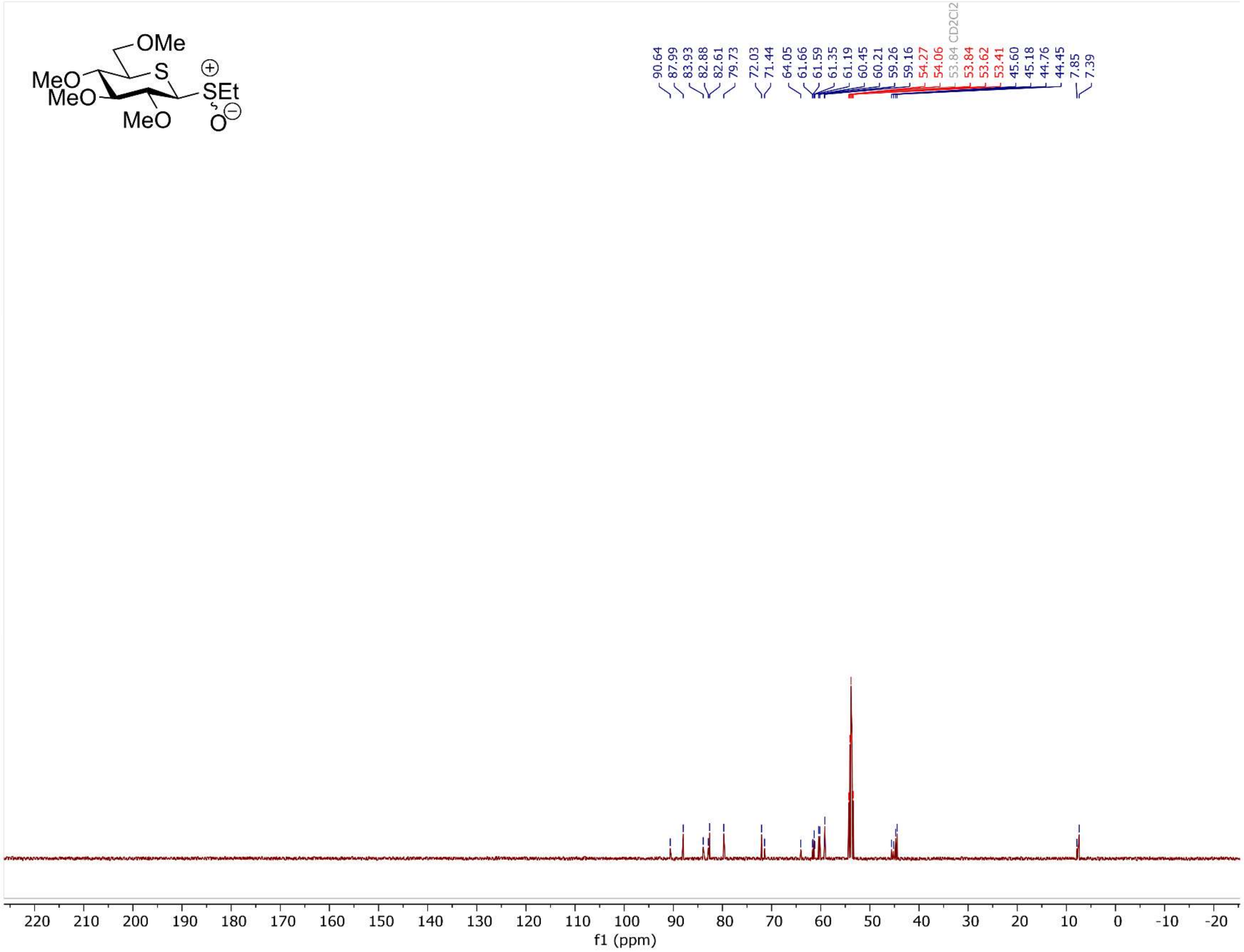

DEPT-90 (CD_2Cl_2) spectrum of (R_S),(S_S)-ethyl 2,3,4,6-tetra-*O*-methyl-1,5-dithio-*β*-D-glucopyranoside-1-*S*-Oxides (**45**):

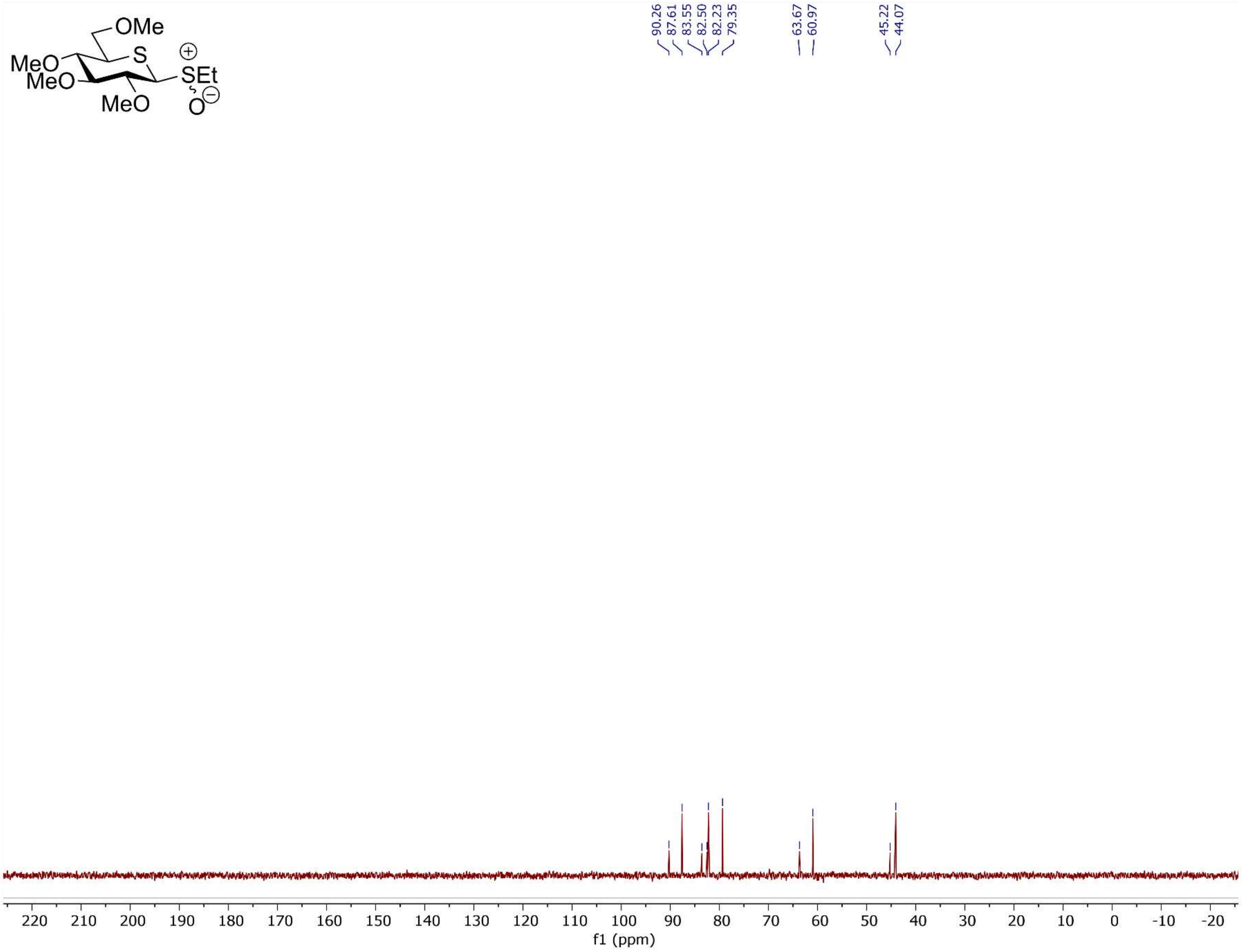

COSY (CD_2Cl_2) spectrum of (R_S),(S_S)-ethyl 2,3,4,6-tetra-*O*-methyl-1,5-dithio-*β*-D-glucopyranoside-1-*S*-Oxides (**45**):

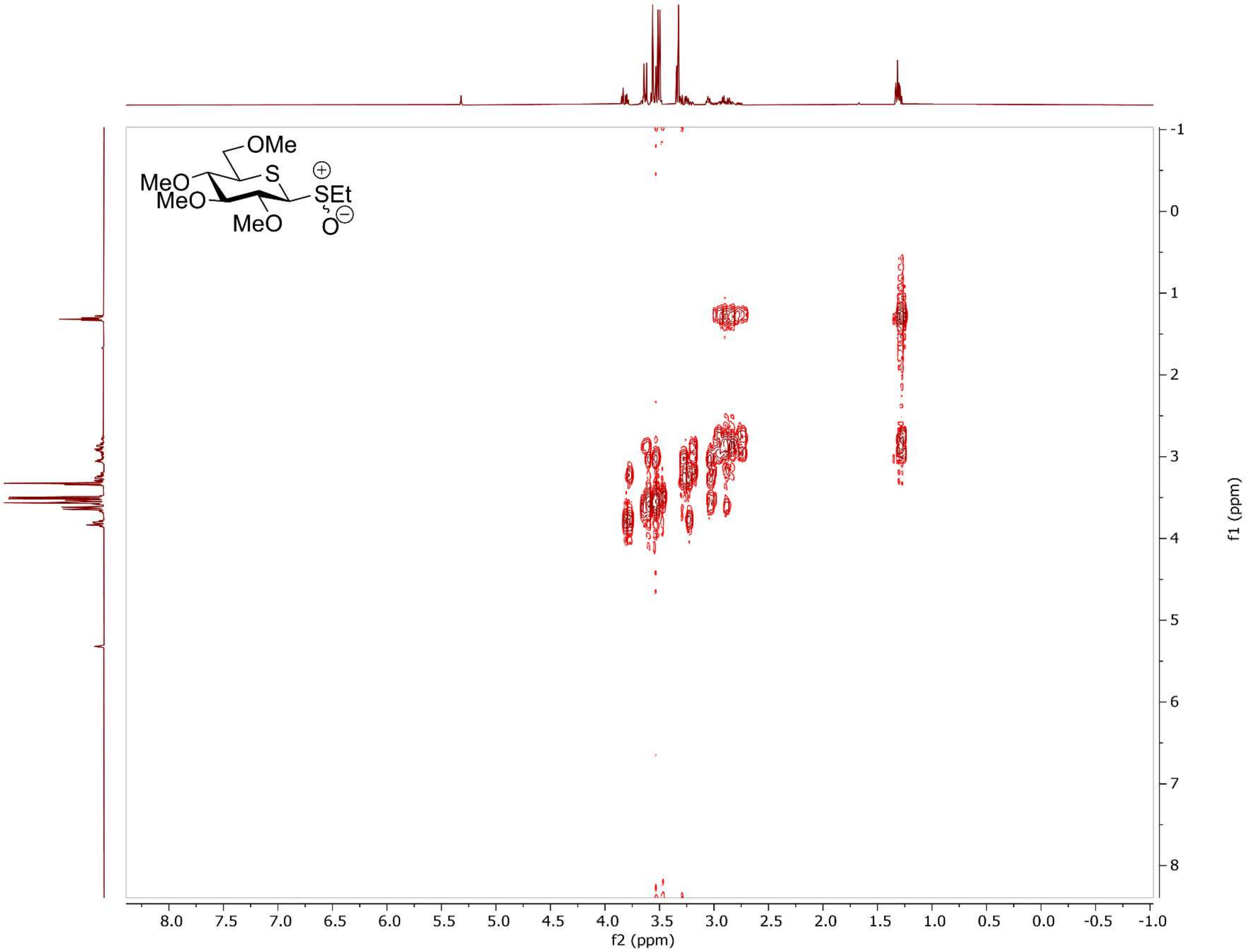

HSQC (CD_2Cl_2) spectrum of (*R*$_S$),(*S*$_S$)-ethyl 2,3,4,6-tetra-*O*-methyl-1,5-dithio-*β*-D-glucopyranoside-1-*S*-Oxides (**45**):

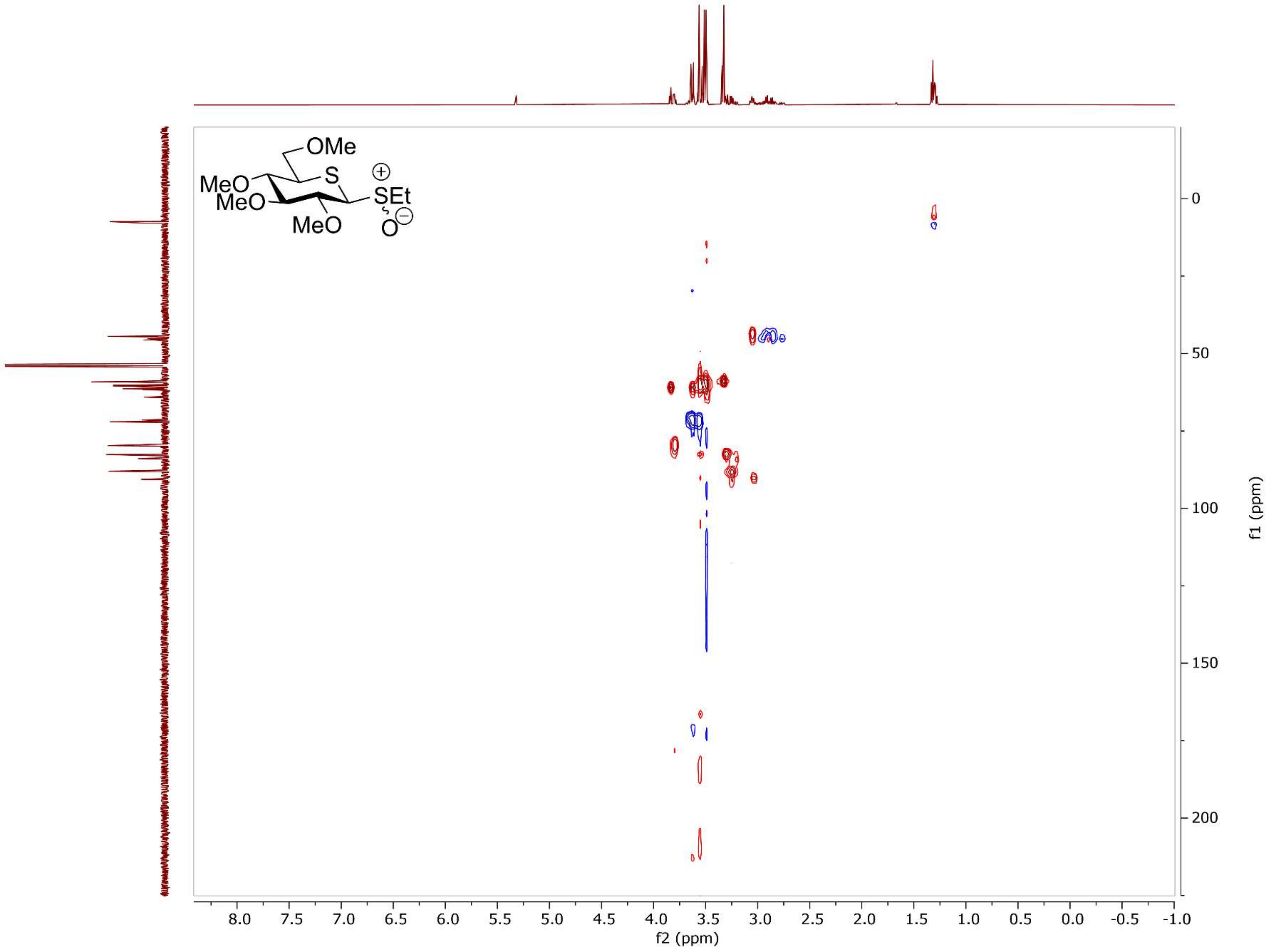

^{1}H NMR (500 MHz, $CDCl_3$) spectrum of (R_S),(S_S)-ethyl 2,3,4,6-tetra-*O*-methyl-1,5-dithio-*β*-D-glucopyranoside-5-*S*-Oxides (**46**):

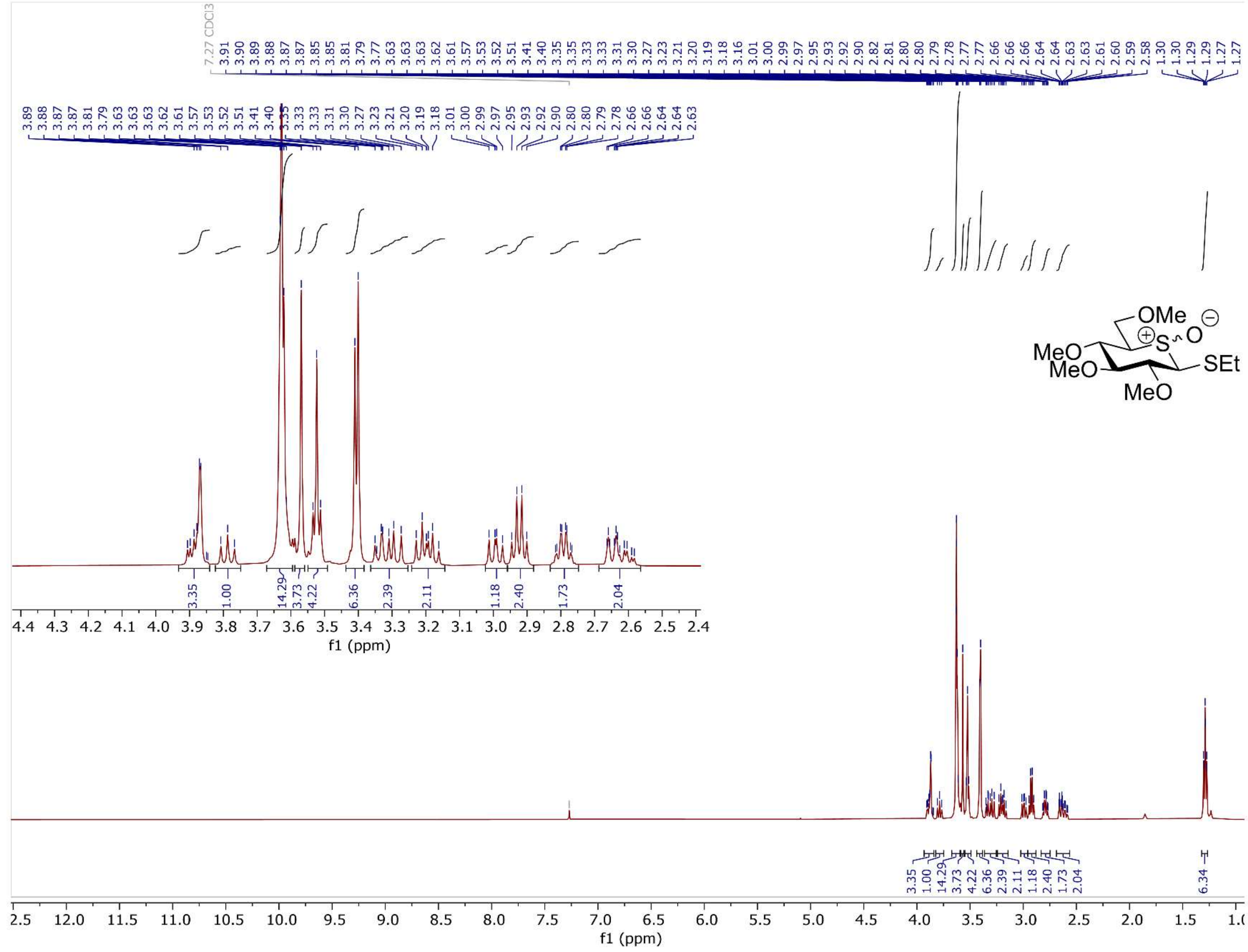

^{13}C NMR (125.67 MHz, $CDCl_3$) spectrum of (R_S),(S_S)-ethyl 2,3,4,6-tetra-*O*-methyl-1,5-dithio-*β*-D-glucopyranoside-5-*S*-Oxides (**46**):

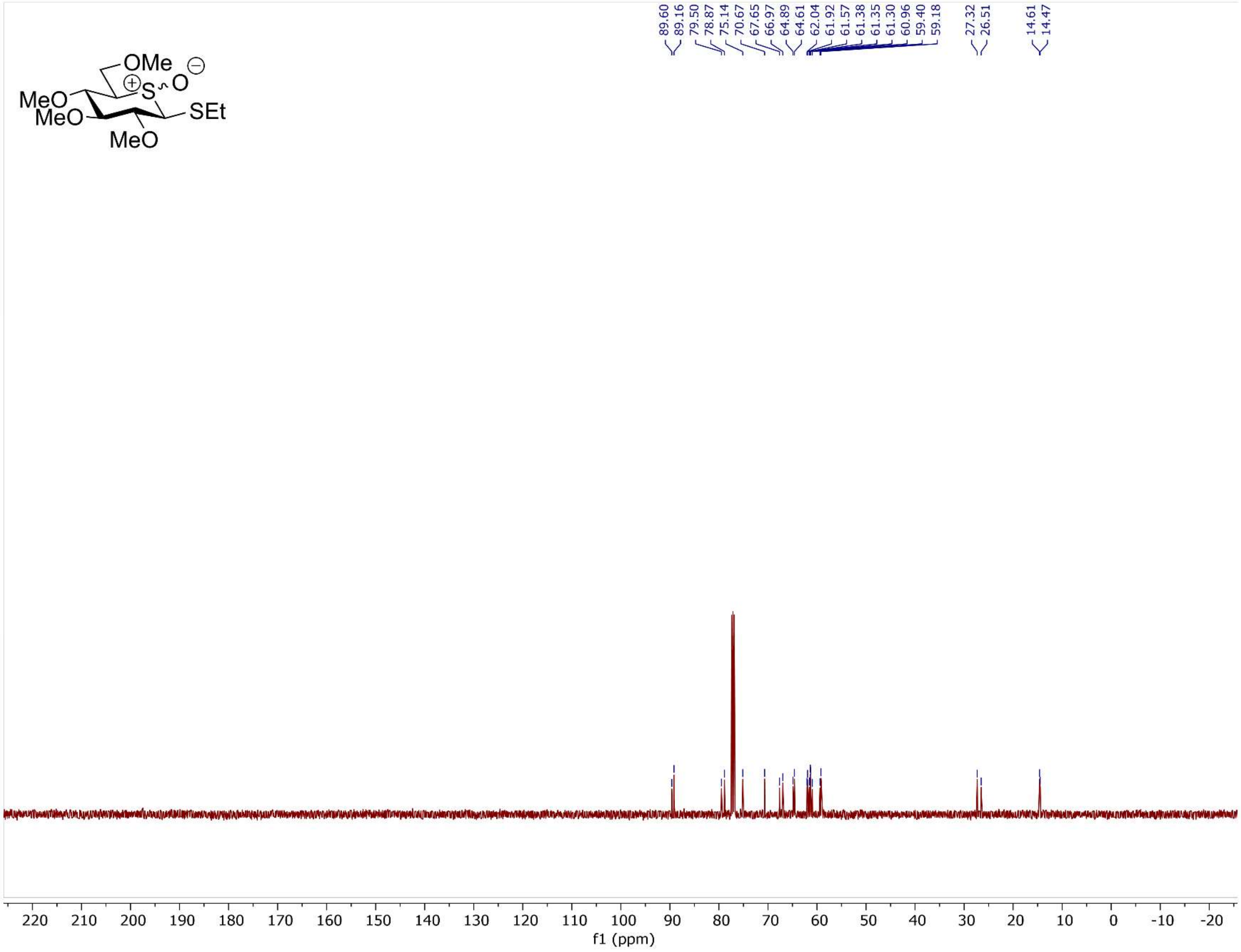

COSY ($CDCl_3$) spectrum of (R_S),(S_S)-ethyl 2,3,4,6-tetra-*O*-methyl-1,5-dithio-*β*-D-glucopyranoside-5-*S*-Oxides (**46**):

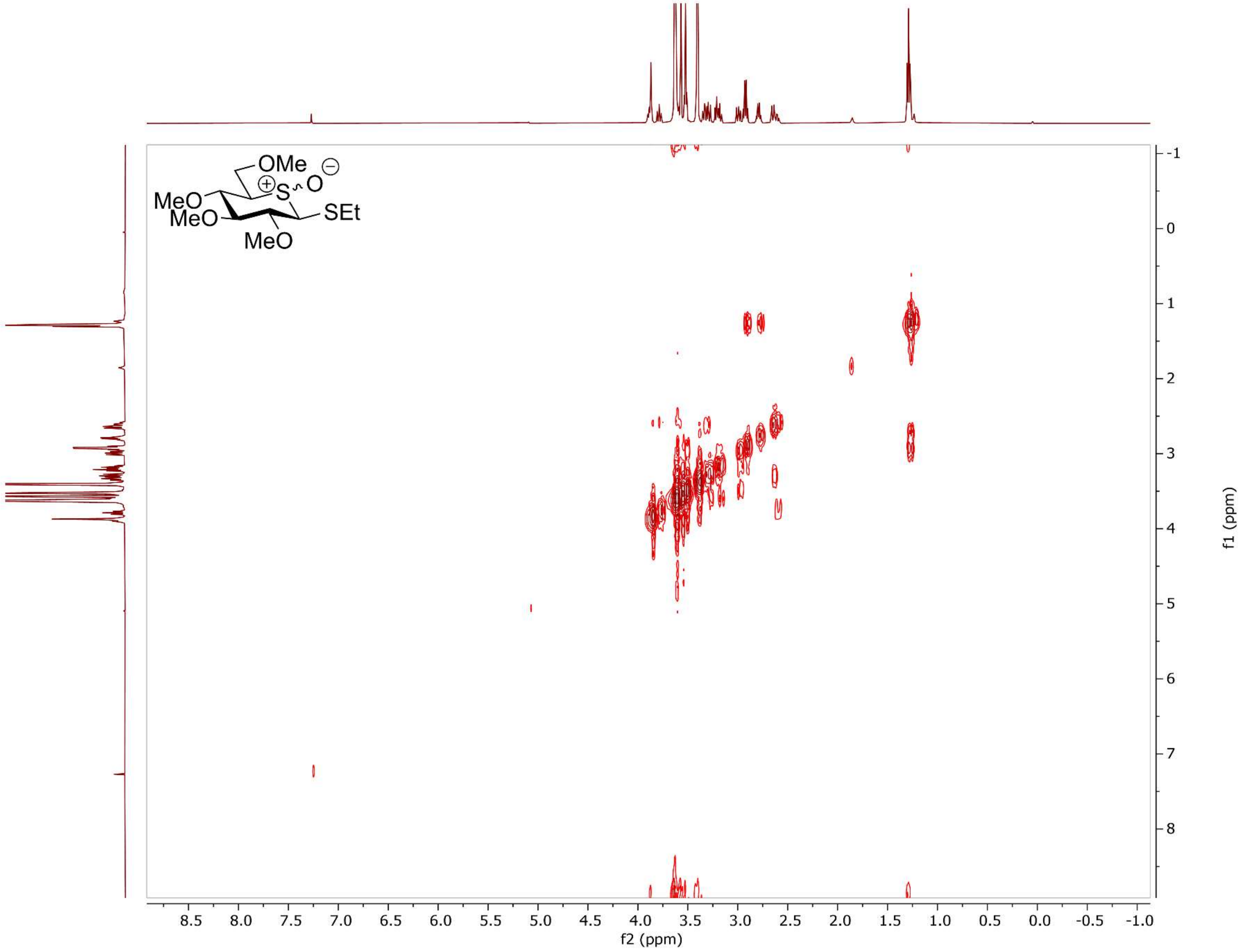

HSQC ($CDCl_3$) spectrum of (R_S),(S_S)-ethyl 2,3,4,6-tetra-*O*-methyl-1,5-dithio-*β*-D-glucopyranoside-5-*S*-Oxides (**46**):

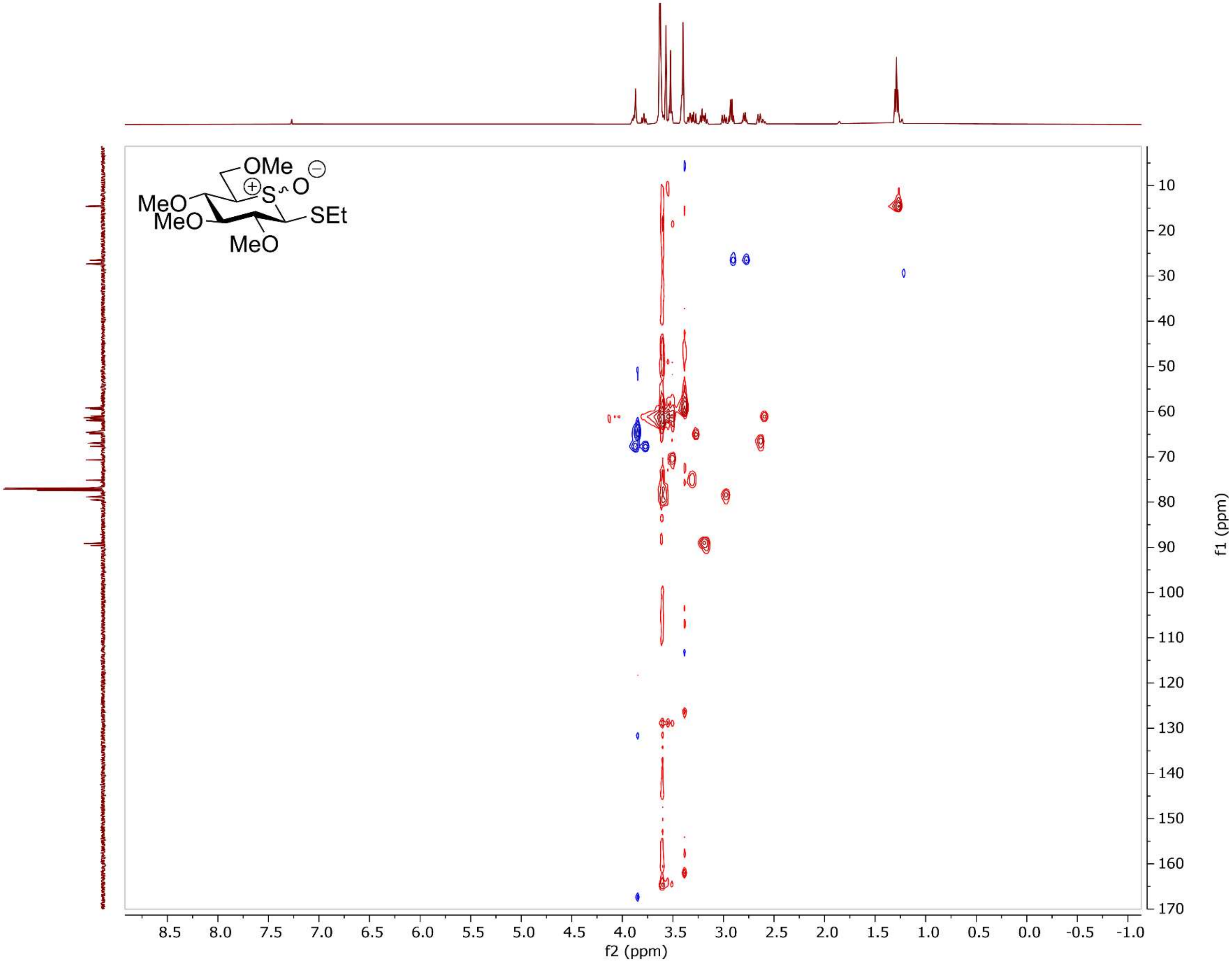

^{1}H NMR (500 MHz, $CDCl_3$) spectrum of ethyl 2,3,4,6-tetra-*O*-methyl-1,5-di-thio-*α*-D-glucopyranoside (**47**):

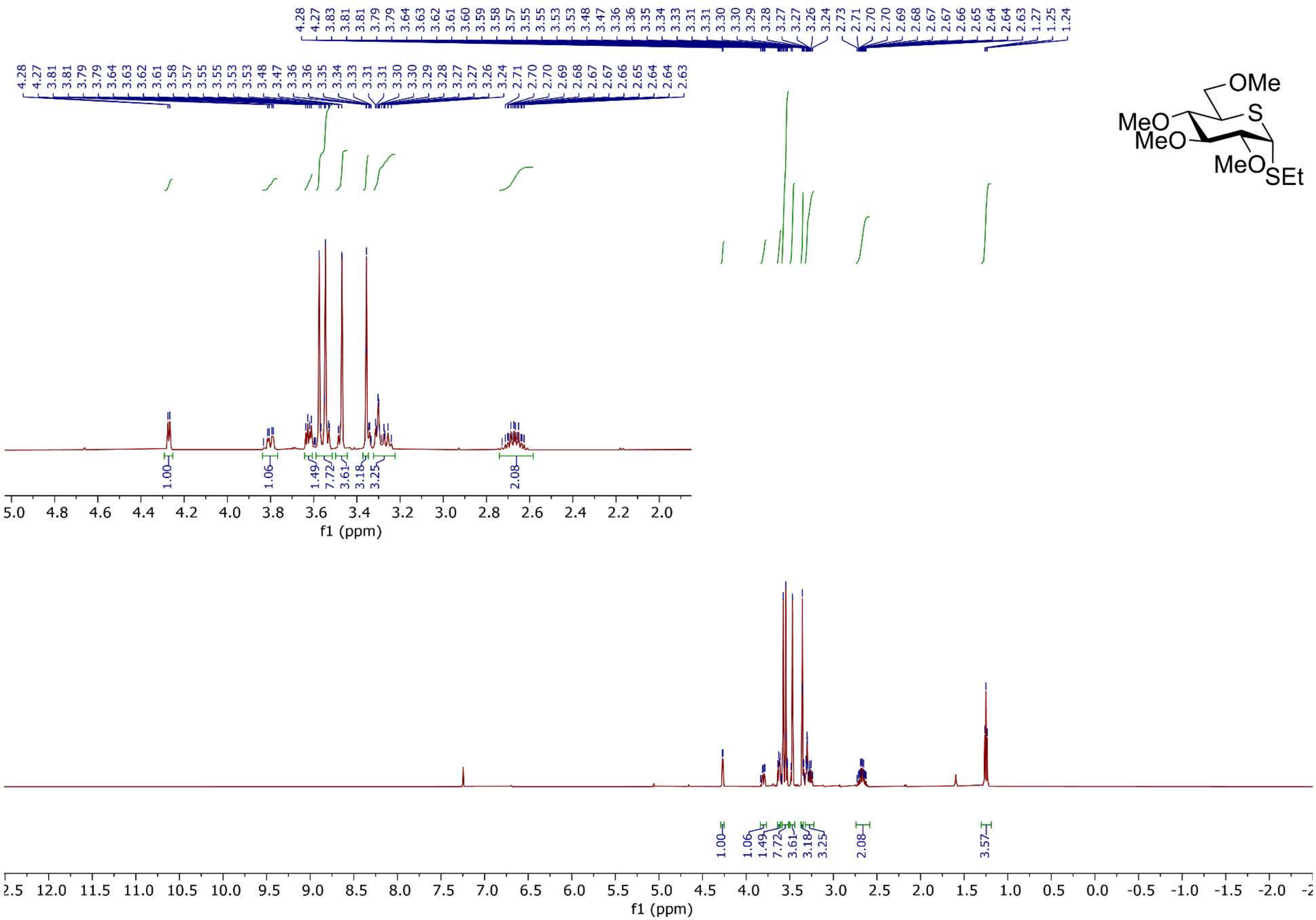

^{13}C NMR (125.67 MHz, $CDCl_3$) spectrum of ethyl 2,3,4,6-tetra-*O*-methyl-1,5-di-thio-*α*-D-glucopyranoside (**47**):

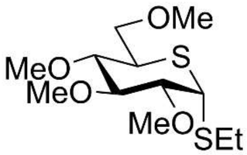

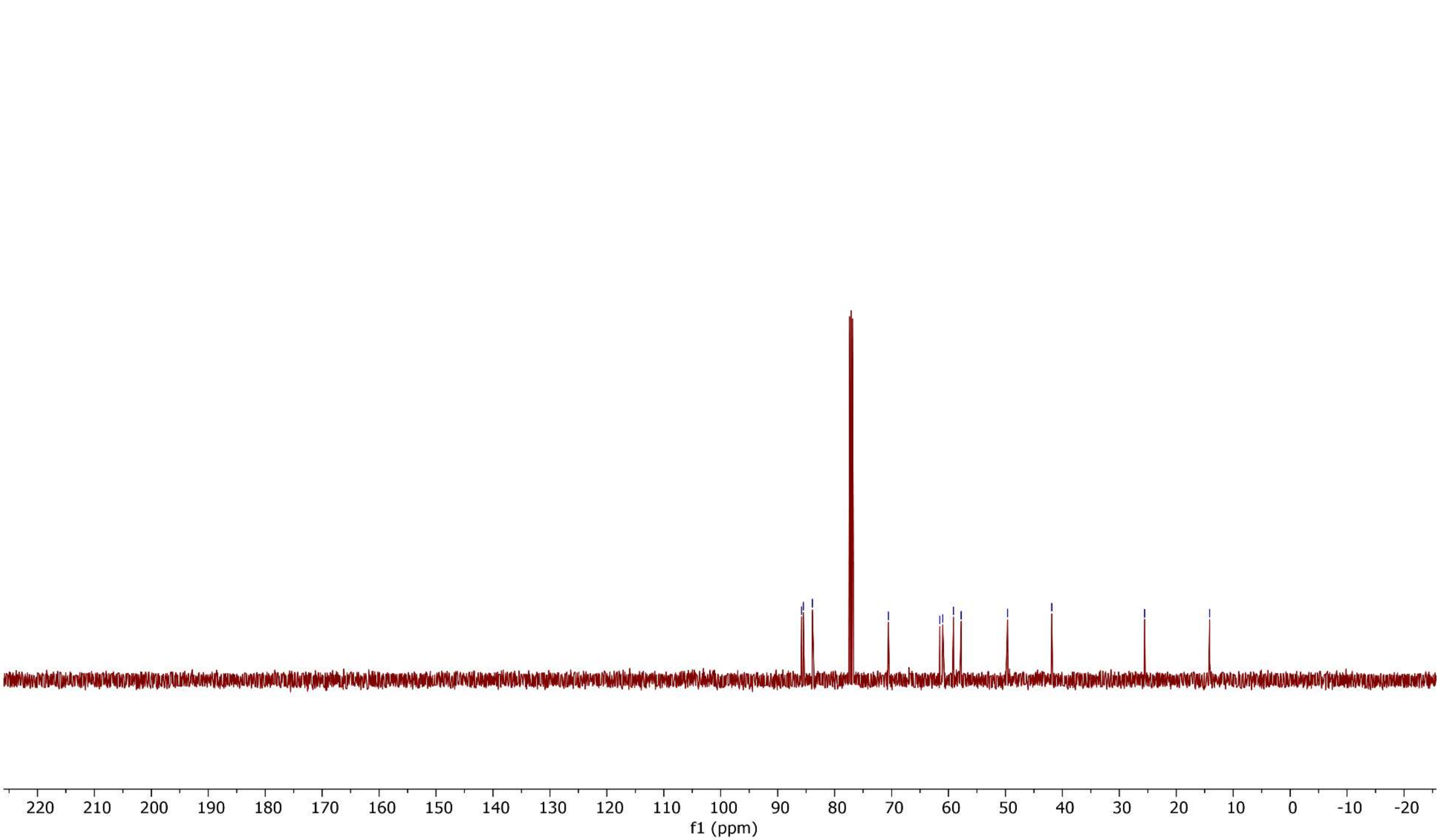

COSY ($CDCl_3$) spectrum of ethyl 2,3,4,6-tetra-*O*-methyl-1,5-di-thio-*α*-D-glucopyranoside (**47**):

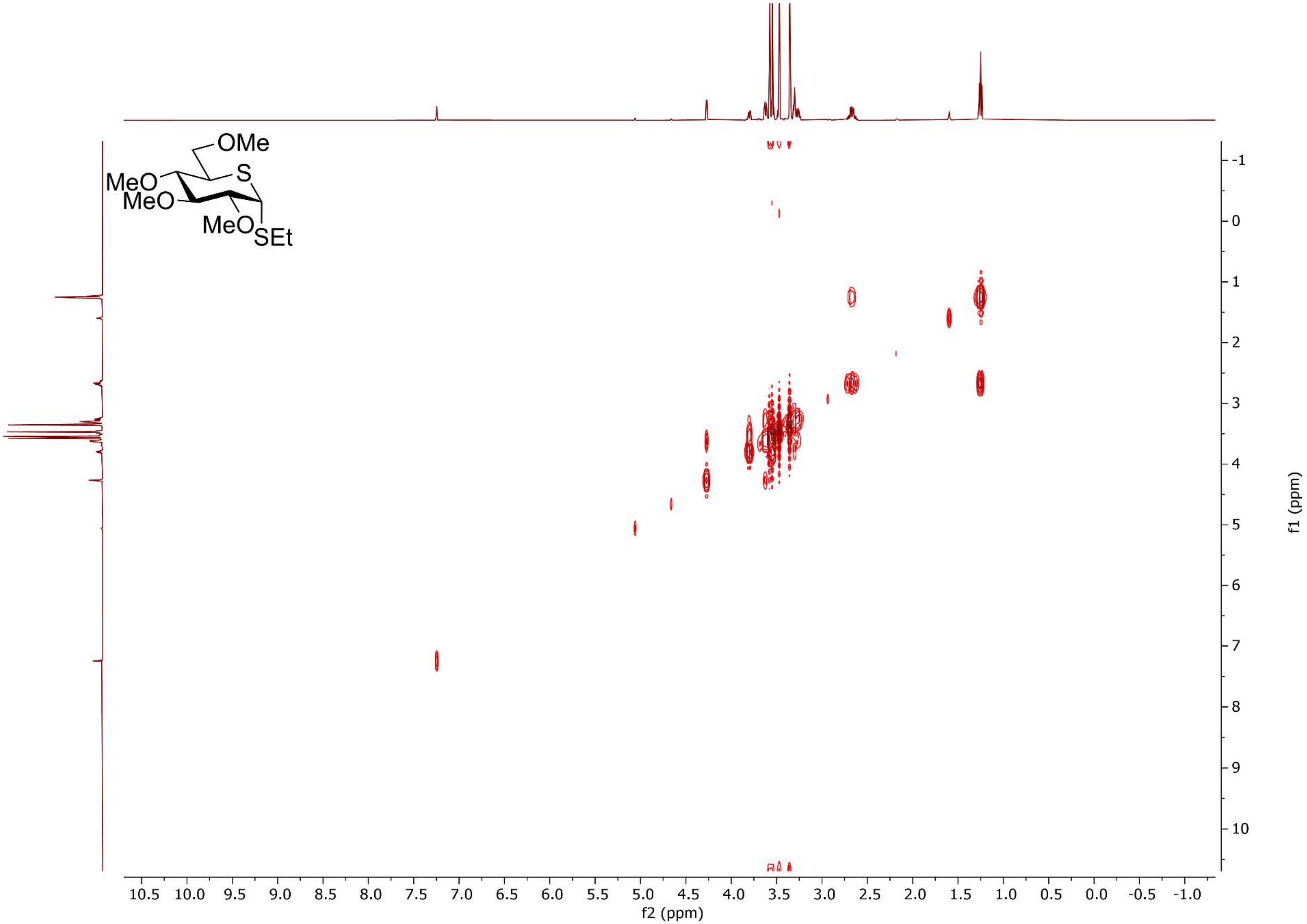

HSQC (CDCl3) spectrum of ethyl 2,3,4,6-tetra-*O*-methyl-1,5-di-thio-*α*-D-glucopyranoside (**47**):

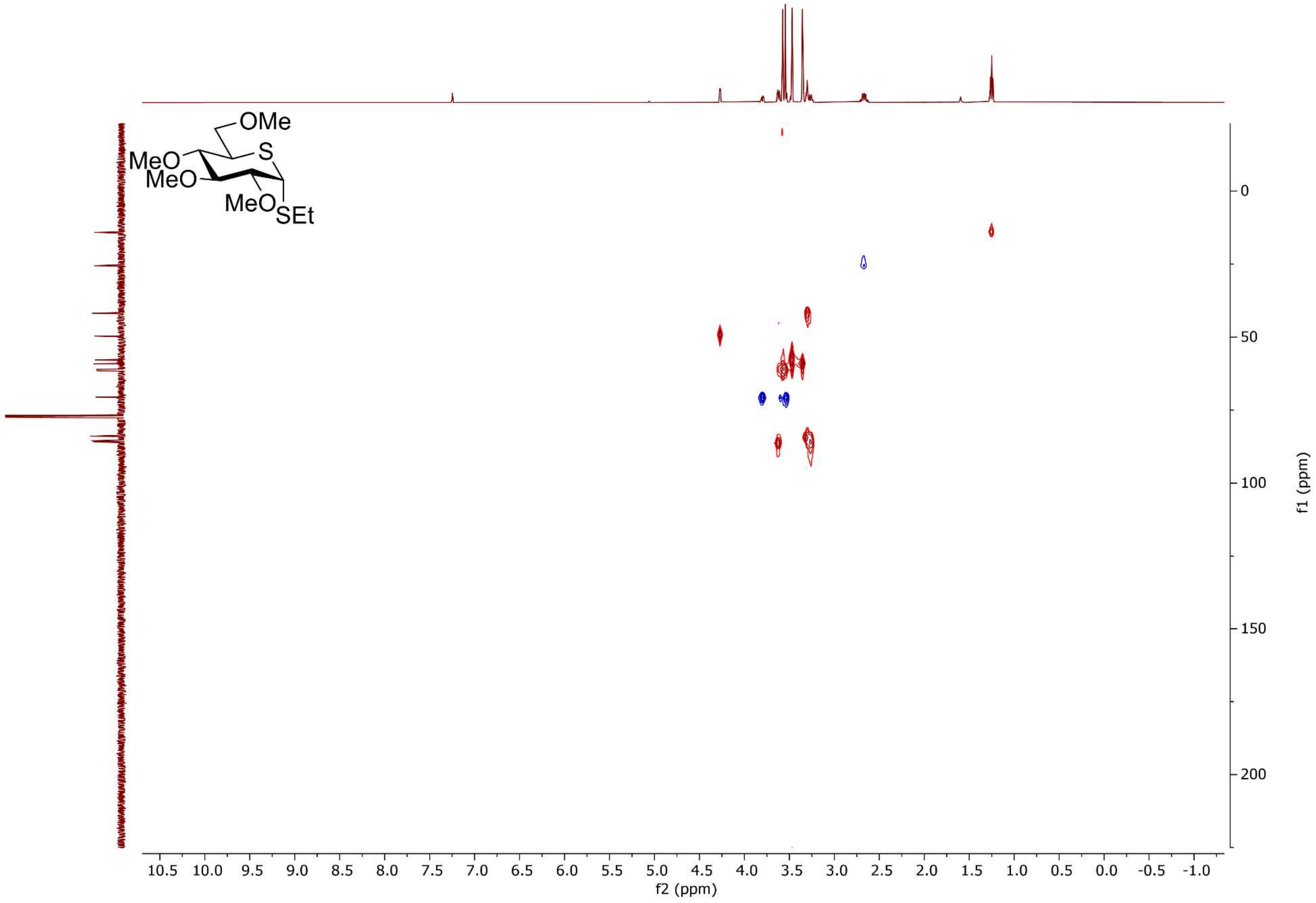

^{1}H NMR (500 MHz, CD_2Cl_2) spectrum of (R_S),(S_S)-ethyl 2,3,4,6-tetra-*O*-methyl-1,5-di-thio-*α*-D-glucopyranosyl-1-*S*-Oxides (**48**):

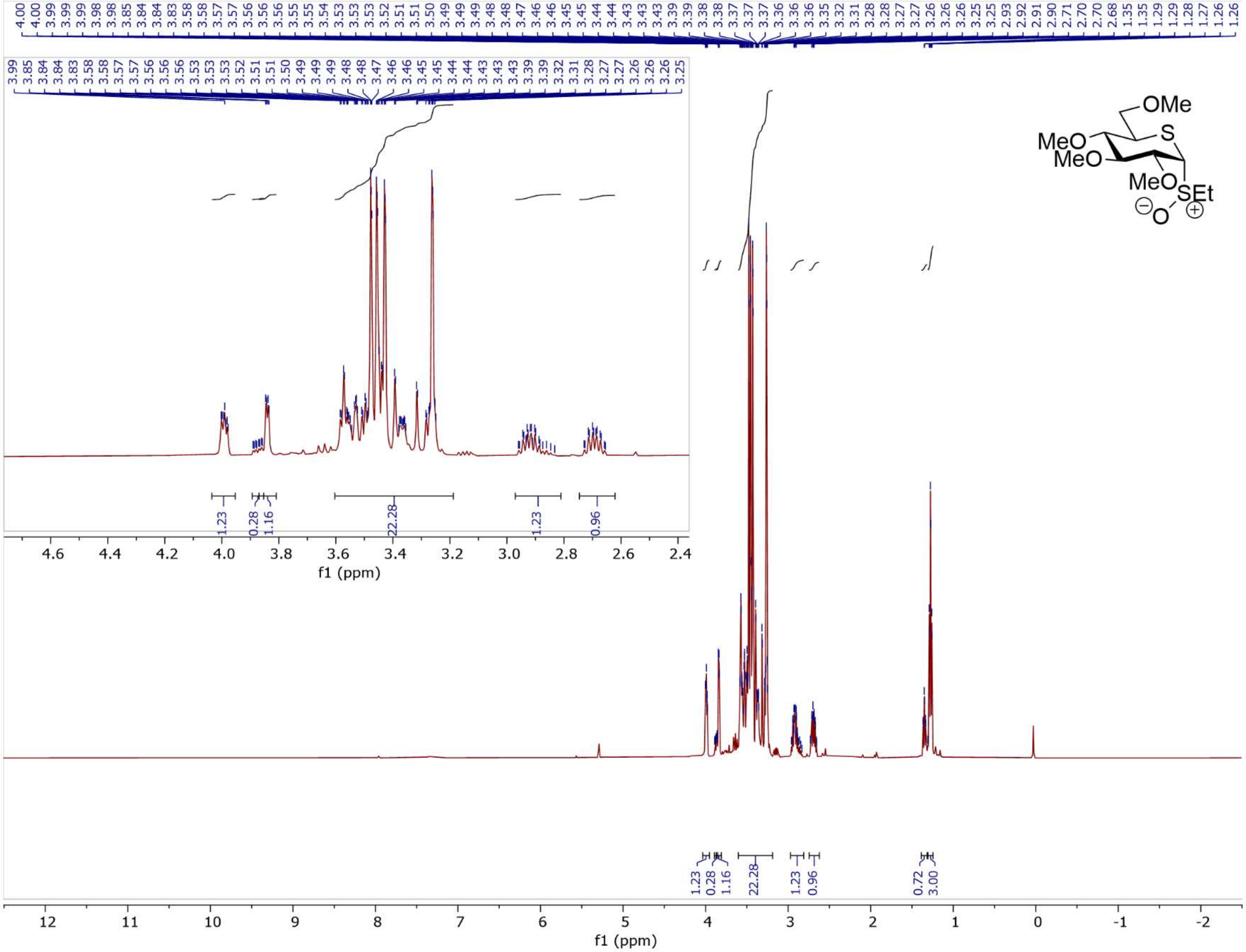

^{13}C NMR (125.67 MHz, CD_2Cl_2) spectrum of (R_S),(S_S)-ethyl 2,3,4,6-tetra-*O*-methyl-1,5-di-thio-*α*-D-glucopyranosyl-1-*S*-Oxides (**48**):

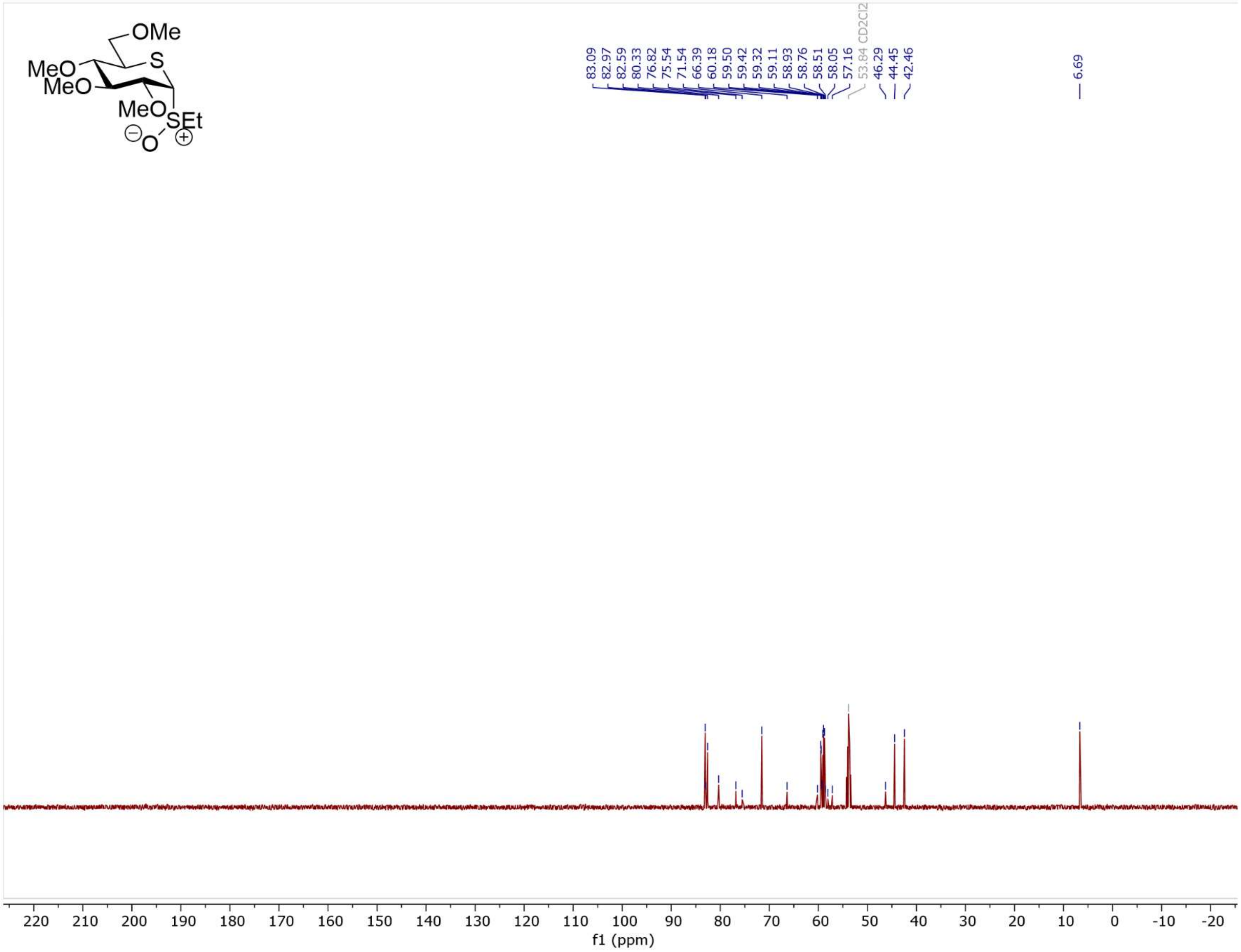

DEPT-135 (CD_2Cl_2) spectrum of (R_S),(S_S)-ethyl 2,3,4,6-tetra-*O*-methyl-1,5-di-thio-*α*-D-glucopyranosyl-1-*S*-Oxides (**48**):

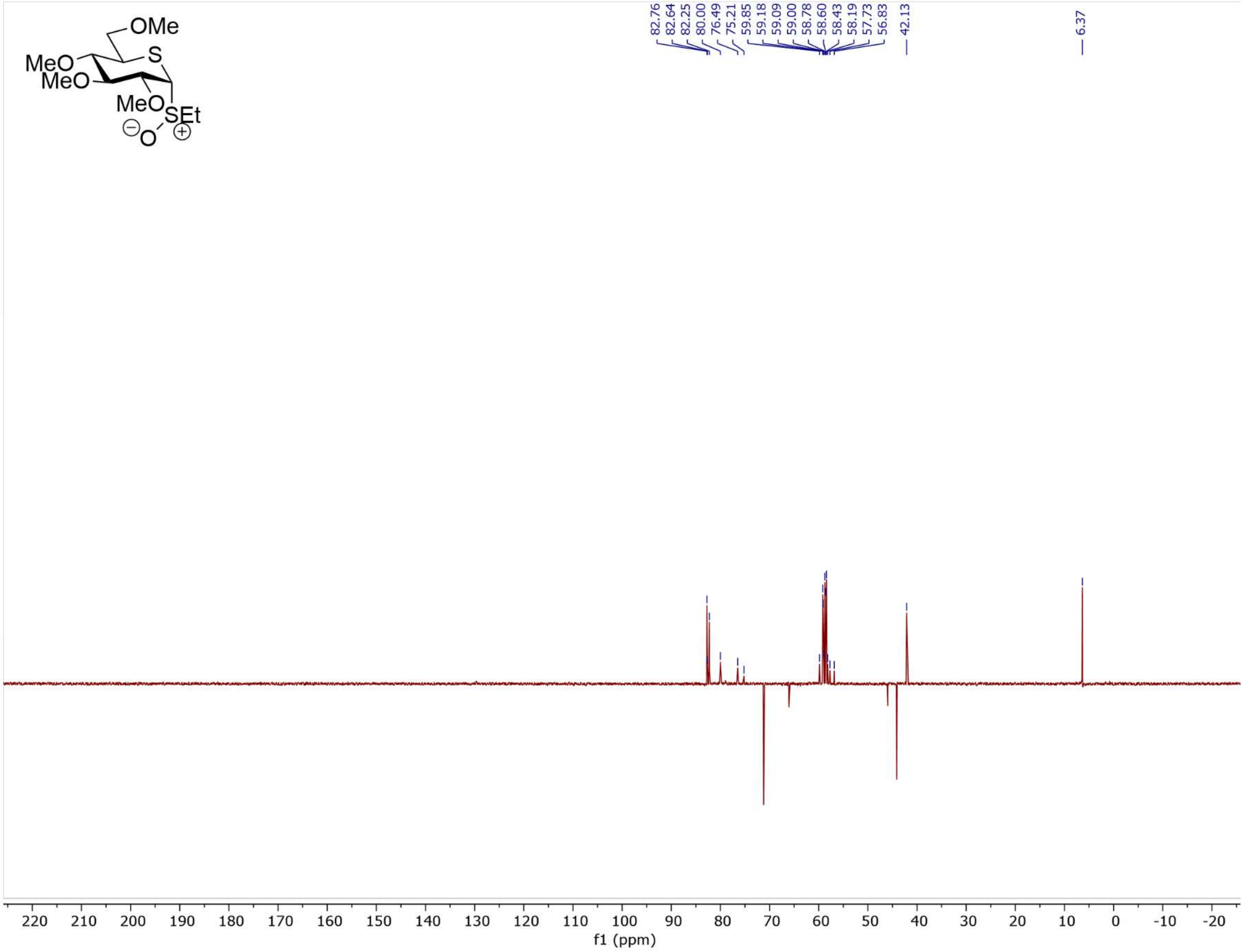

COSY (CD_2Cl_2) spectrum of (*R*$_S$),(*S*$_S$)-ethyl 2,3,4,6-tetra-*O*-methyl-1,5-di-thio-*α*-D-glucopyranosyl-1-*S*-Oxides (**48**):

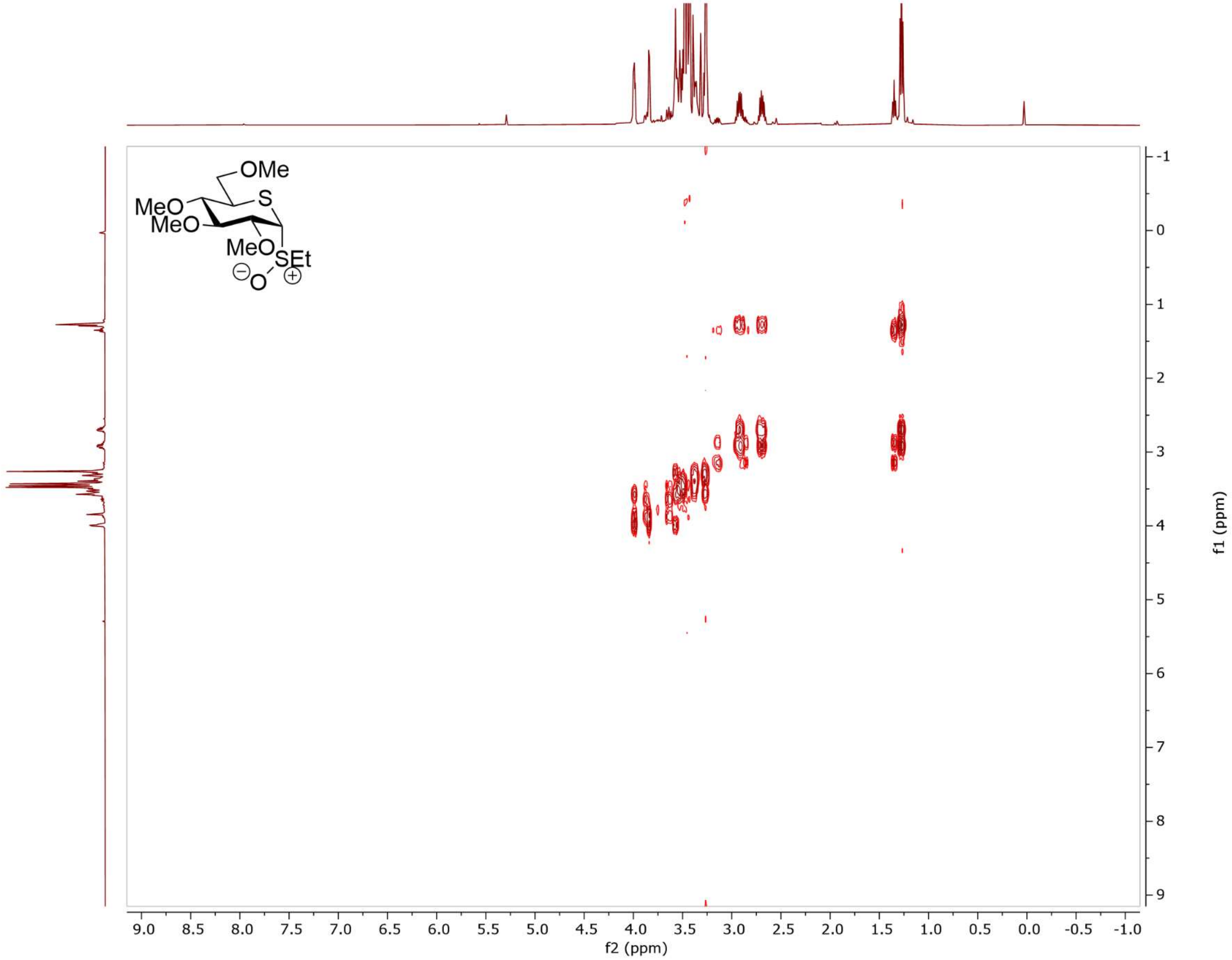

HSQC (CD_2Cl_2) spectrum of (R_S),(S_S)-ethyl 2,3,4,6-tetra-*O*-methyl-1,5-di-thio-*α*-D-glucopyranosyl-1-*S*-Oxides (**48**):

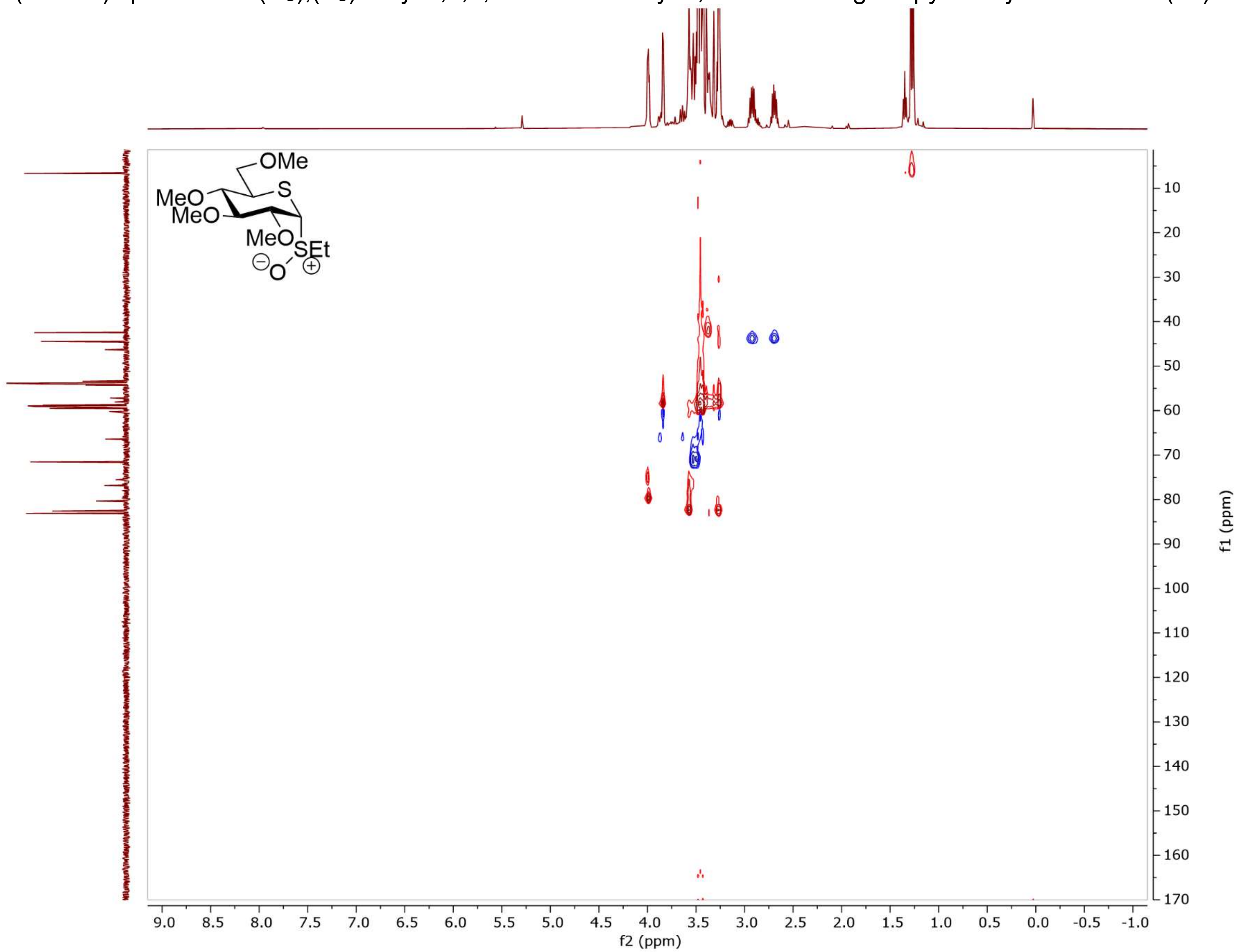

^{1}H NMR (500 MHz, $CDCl_3$) spectrum of (R_S),(S_S)-ethyl 2,3,4,6-tetra-*O*-acetyl-1-thio-*β*-D-glucopyranosyl-1-*S*-oxides (**50**):

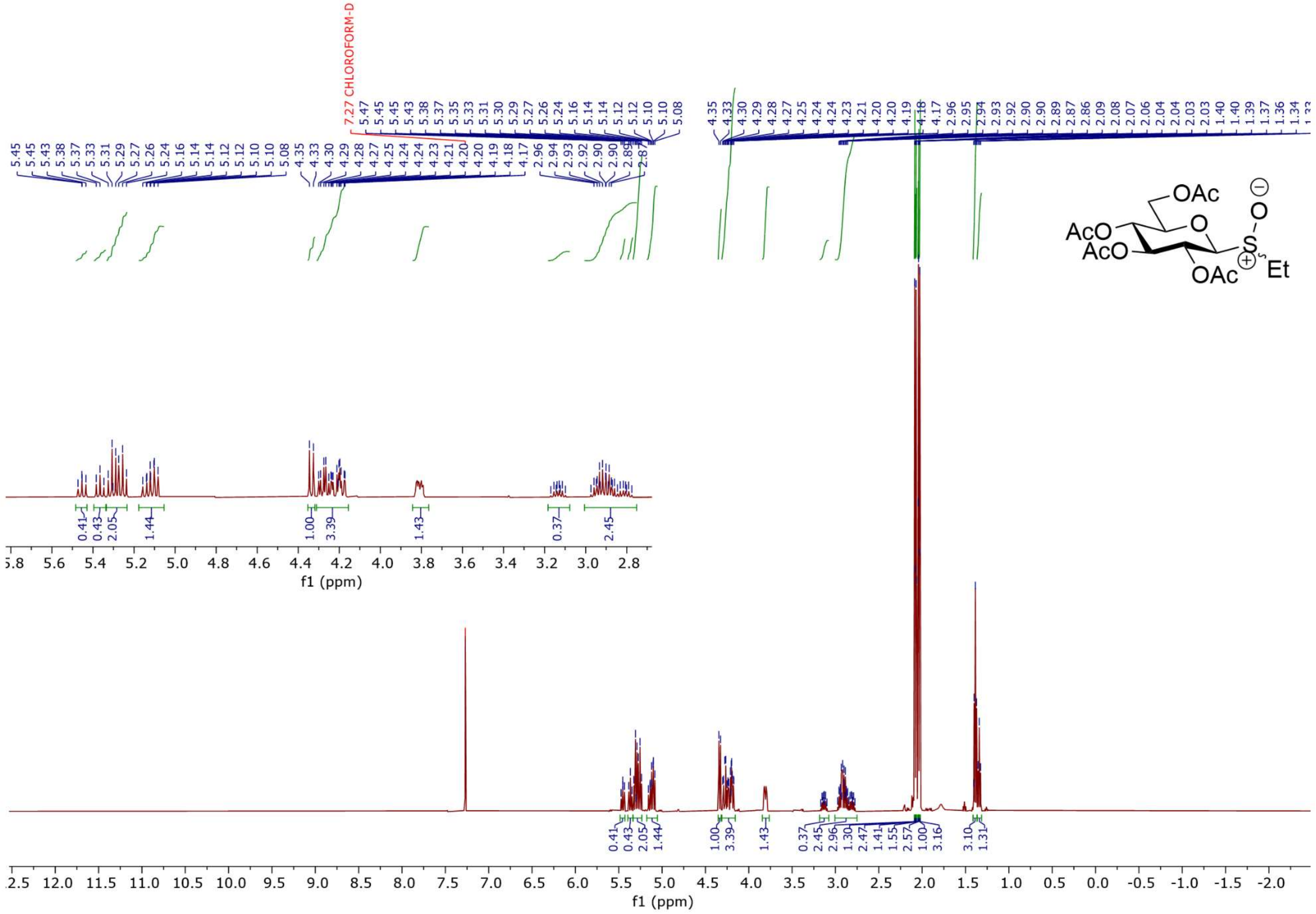

^{13}C NMR (125.67 MHz, $CDCl_3$) spectrum of (R_S),(S_S)-ethyl 2,3,4,6-tetra-*O*-acetyl-1-thio-*β*-D-glucopyranosyl-1-*S*-oxides (**50**):

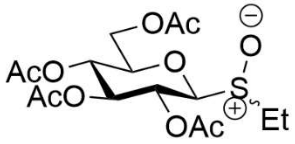

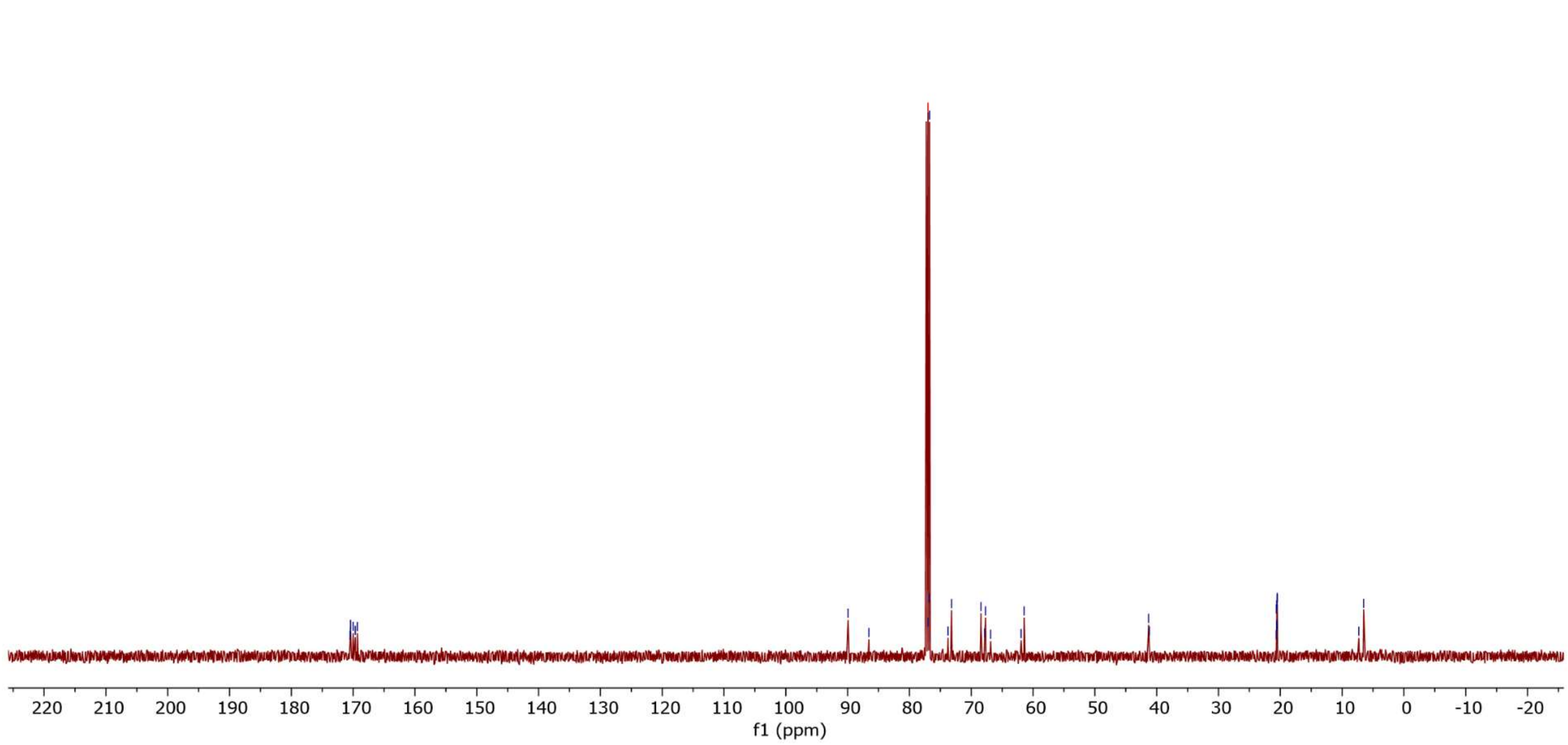

^{1}H NMR (500 MHz, $CDCl_3$) spectrum of (R_S)-ethyl 2,3,4,6-tetra-*O*-acetyl-1-thio-*α*-D-glucopyranosyl-1-*S*-oxide (**51**):

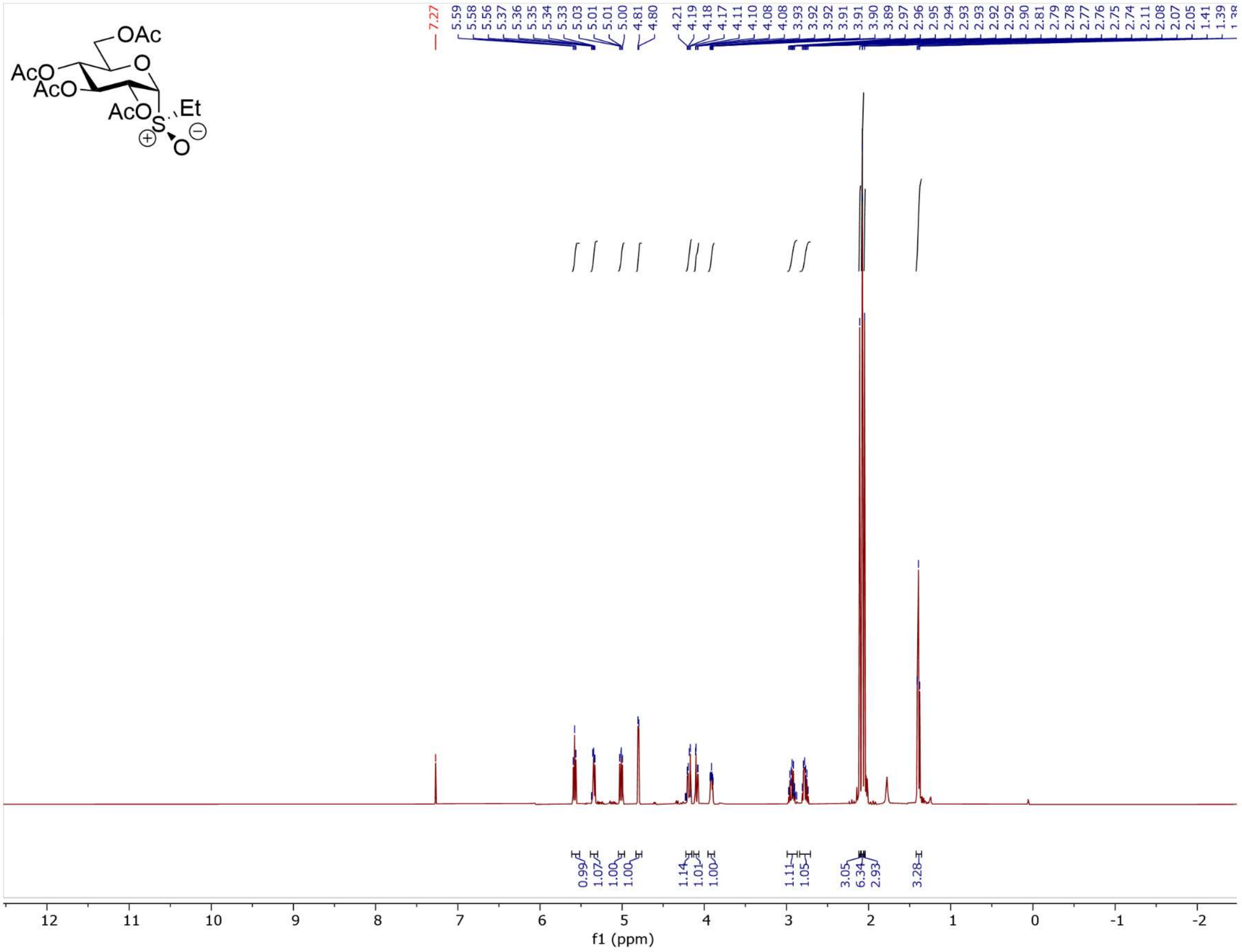

^{13}C NMR (125.67 MHz, $CDCl_3$) spectrum of (R_S)-ethyl 2,3,4,6-tetra-*O*-acetyl-1-thio-*α*-D-glucopyranosyl-1-*S*-oxide (**51**):

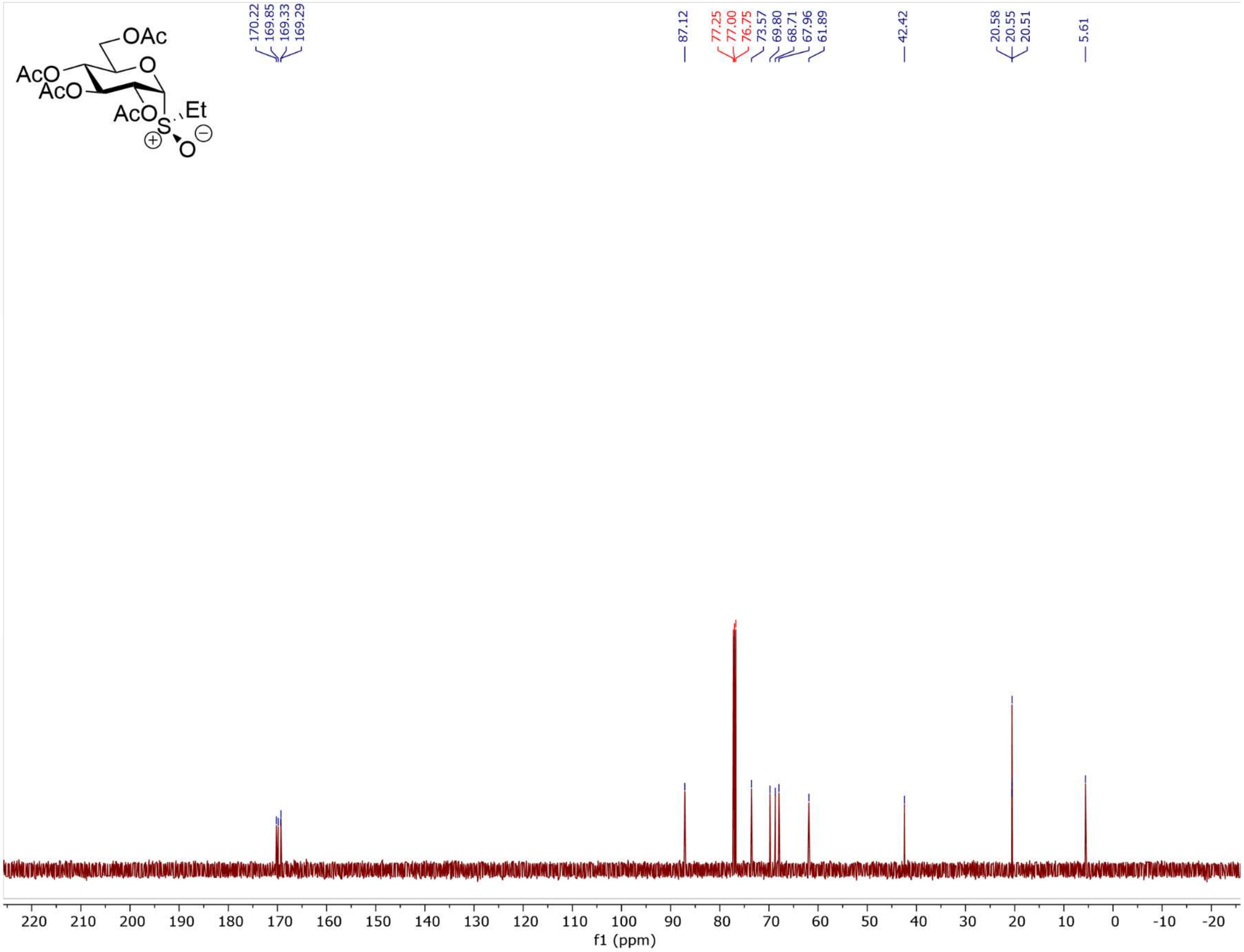

¹H NMR (500 MHz, CD_2Cl_2) spectrum of (R_S),(S_S)-Ethyl 2,3,4,6-tetra-*O*-methyl-1-thio-*β*-D-glucopyranoside-1-*S*-Oxides (**53**):

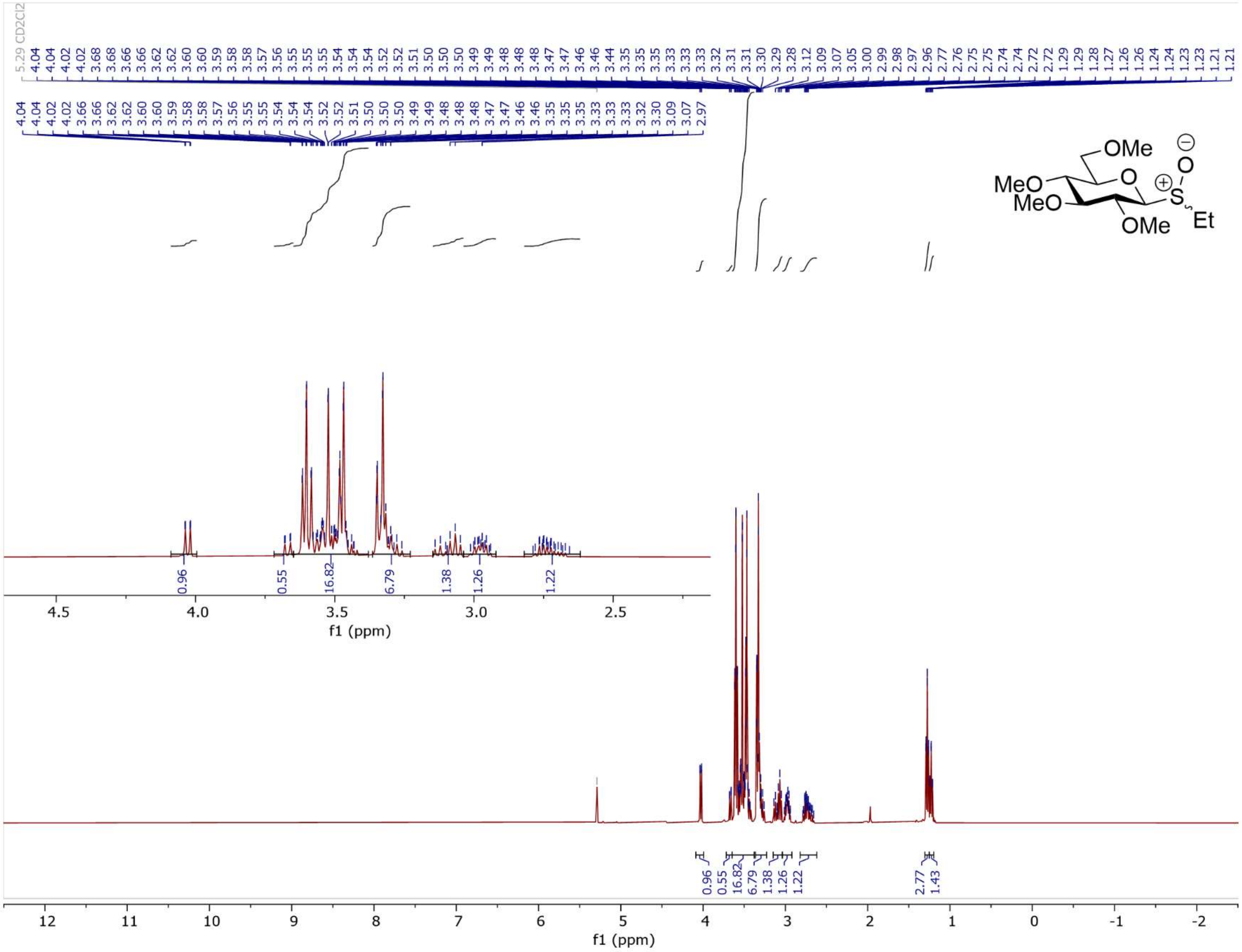

^{13}C NMR (125.67 MHz, CD_2Cl_2) spectrum of (R_S),(S_S)-Ethyl 2,3,4,6-tetra-*O*-methyl-1-thio-*β*-D-glucopyranoside-1-*S*-Oxides (**53**):

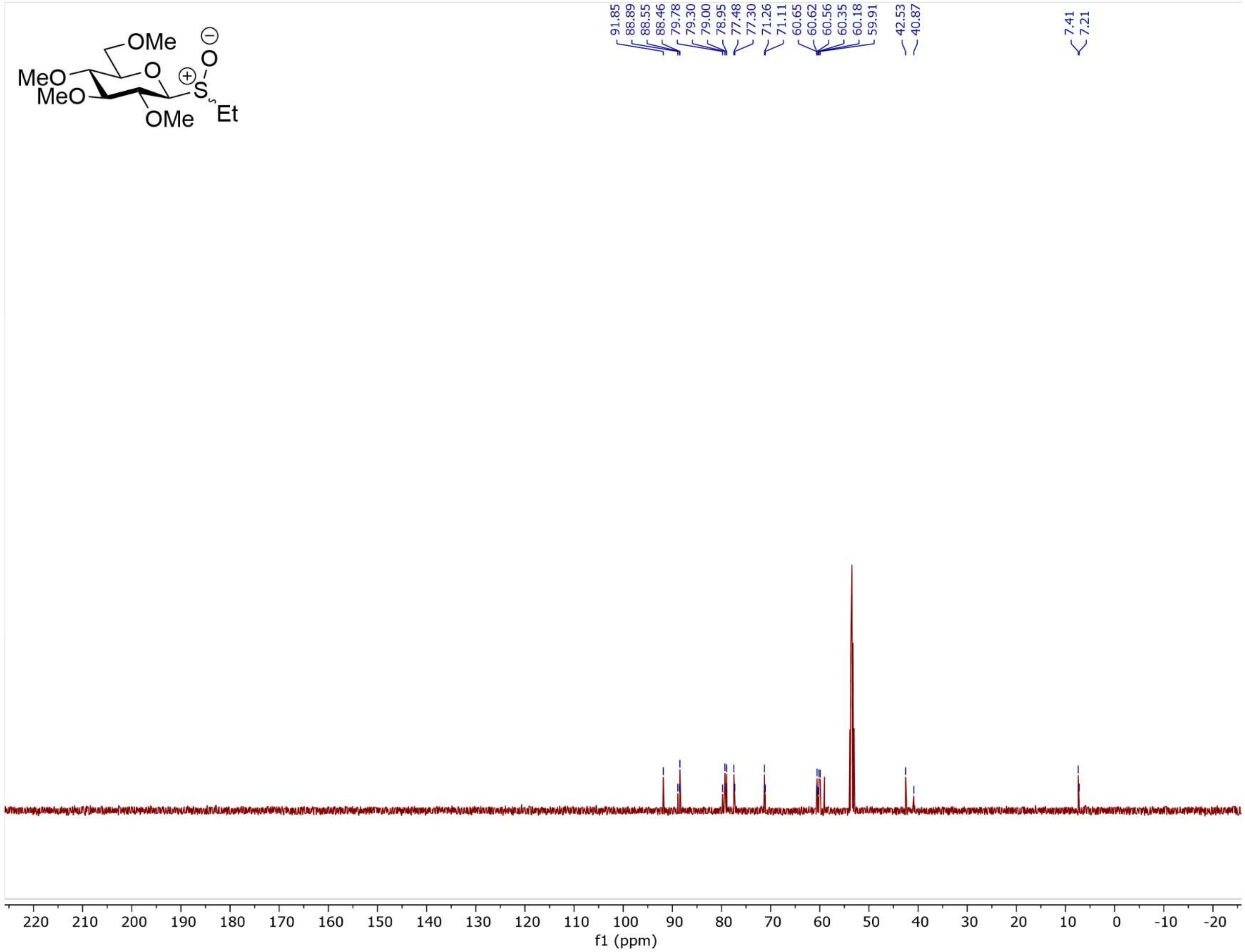

DEPT-90 (CD_2Cl_2) spectrum of (R_S),(S_S)-Ethyl 2,3,4,6-tetra-*O*-methyl-1-thio-*β*-D-glucopyranoside-1-*S*-Oxides (**53**):

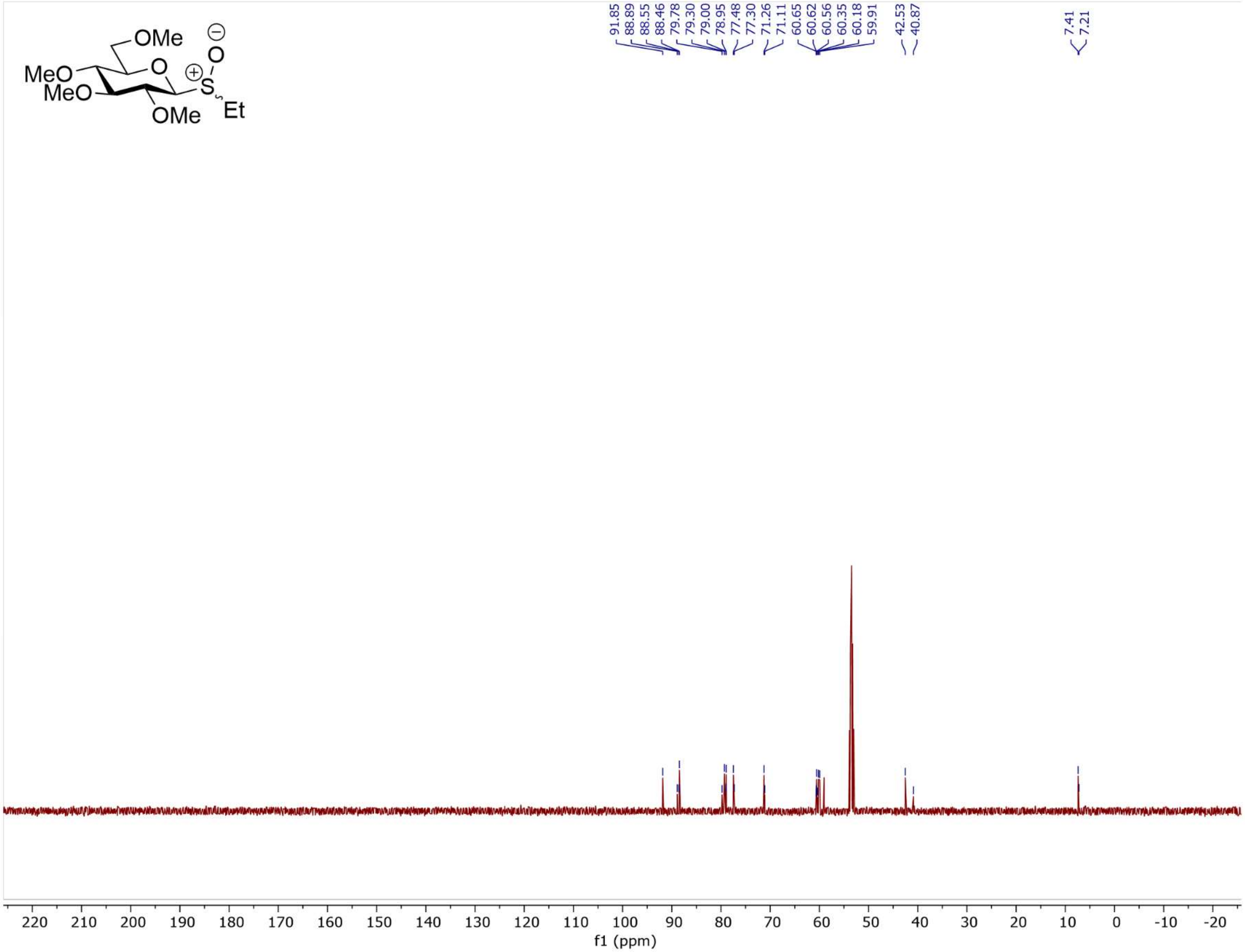

COSY (CD_2Cl_2) spectrum of (*R*$_S$),(*S*$_S$)-Ethyl 2,3,4,6-tetra-*O*-methyl-1-thio-*β*-D-glucopyranoside-1-*S*-Oxides (**53**):

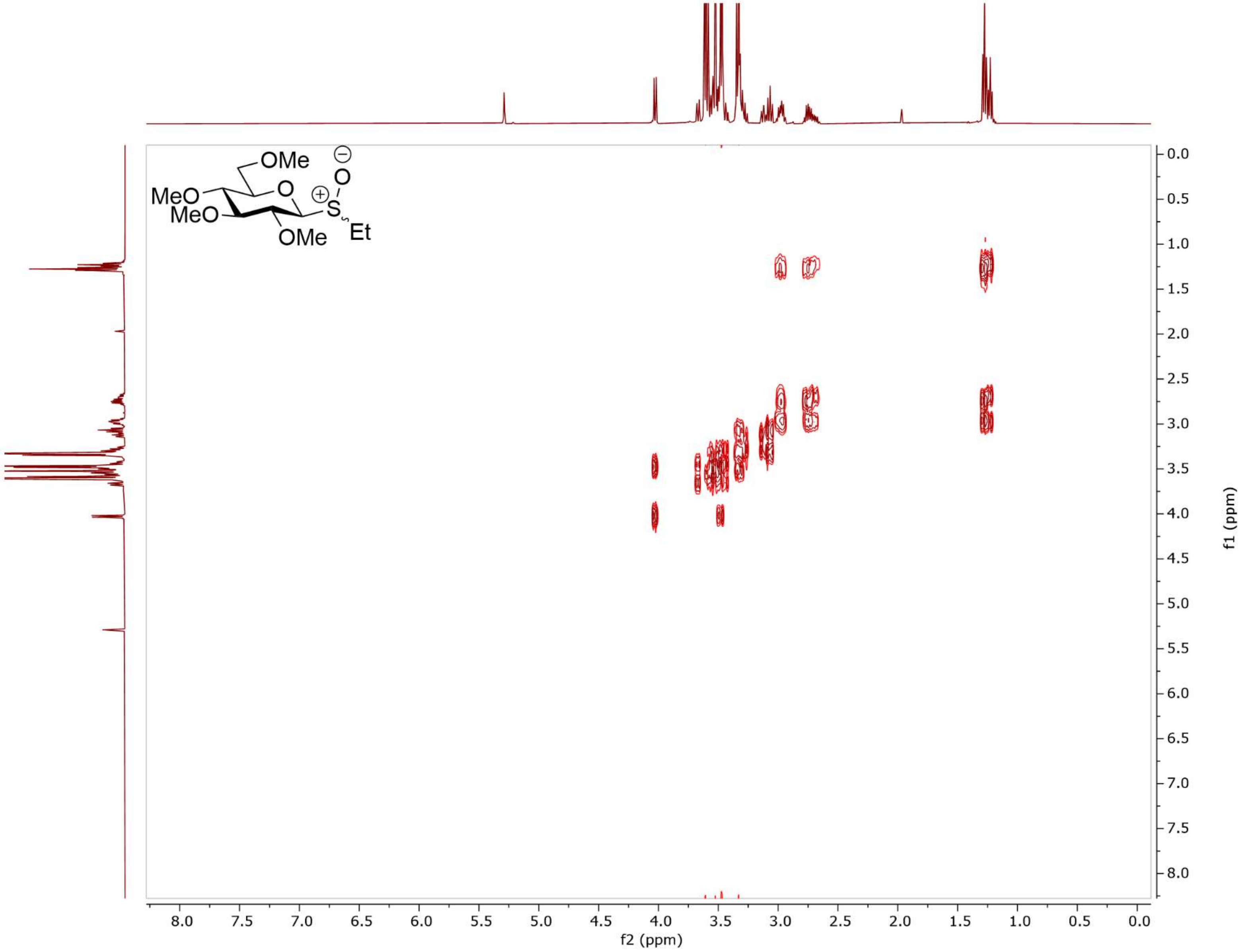

HSQC (CD_2Cl_2) spectrum of (R_S),(S_S)-Ethyl 2,3,4,6-tetra-*O*-methyl-1-thio-*β*-D-glucopyranoside-1-*S*-Oxides (**53**):

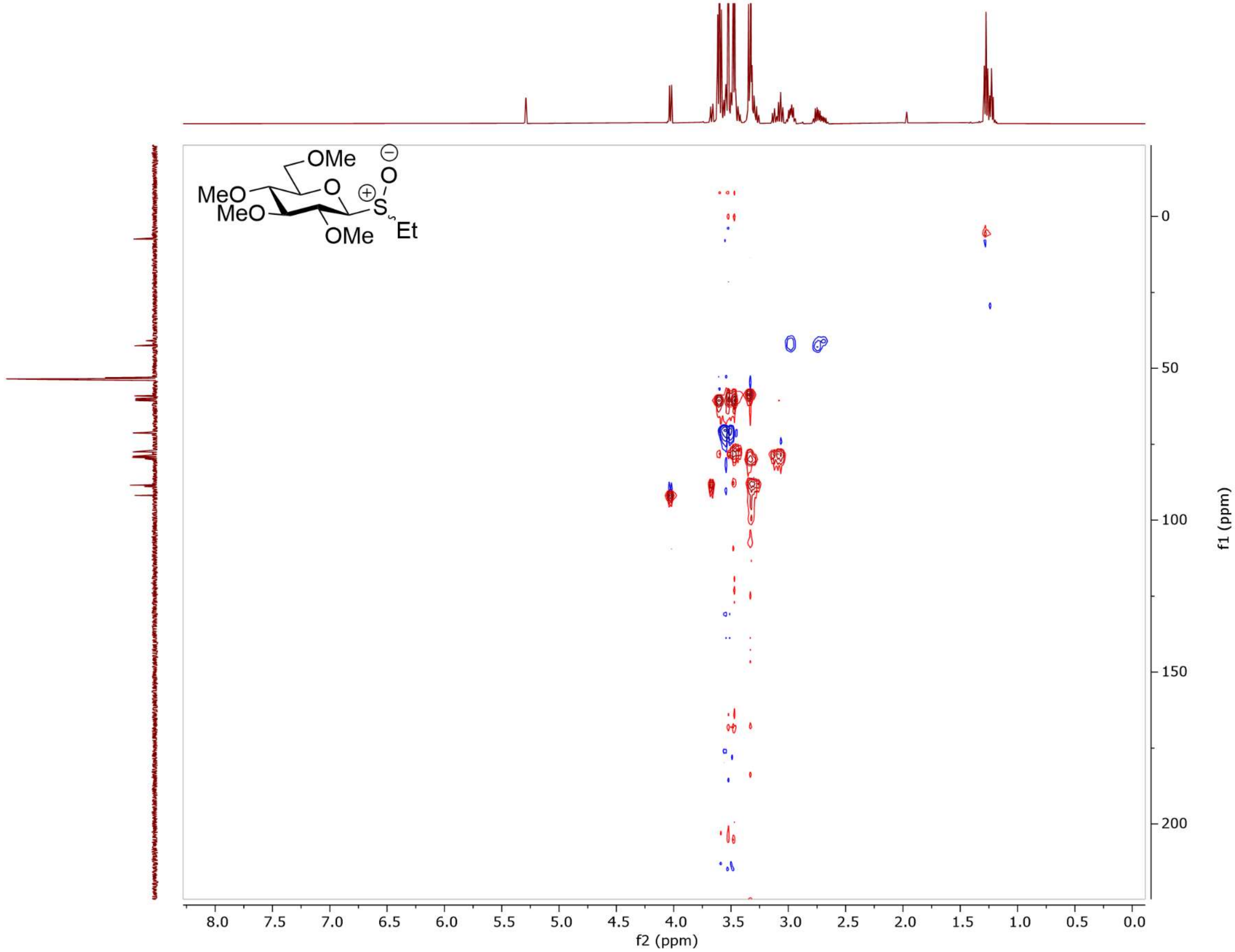

^{1}H NMR (500 MHz, CD_2Cl_2) spectrum of ethyl 2,3,4,6-tetra-*O*-methyl-1-thio-*α*-D-glucopyranoside-1-*S*-oxide (55):

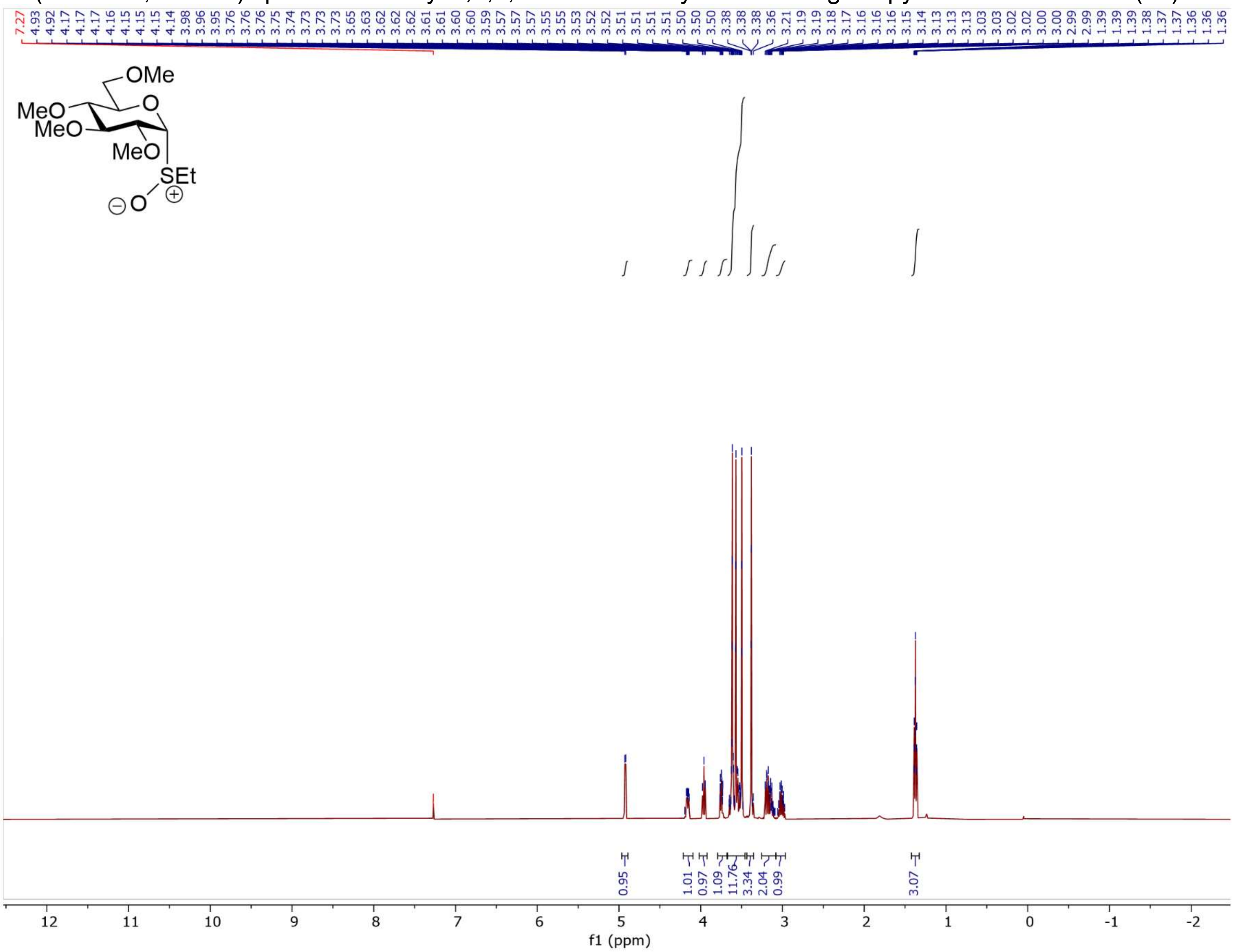

^{13}**C NMR** (125.67 MHz, CD_2Cl_2) spectrum of ethyl 2,3,4,6-tetra-*O*-methyl-1-thio-*α*-D-glucopyranoside-1-*S*-oxide (55):

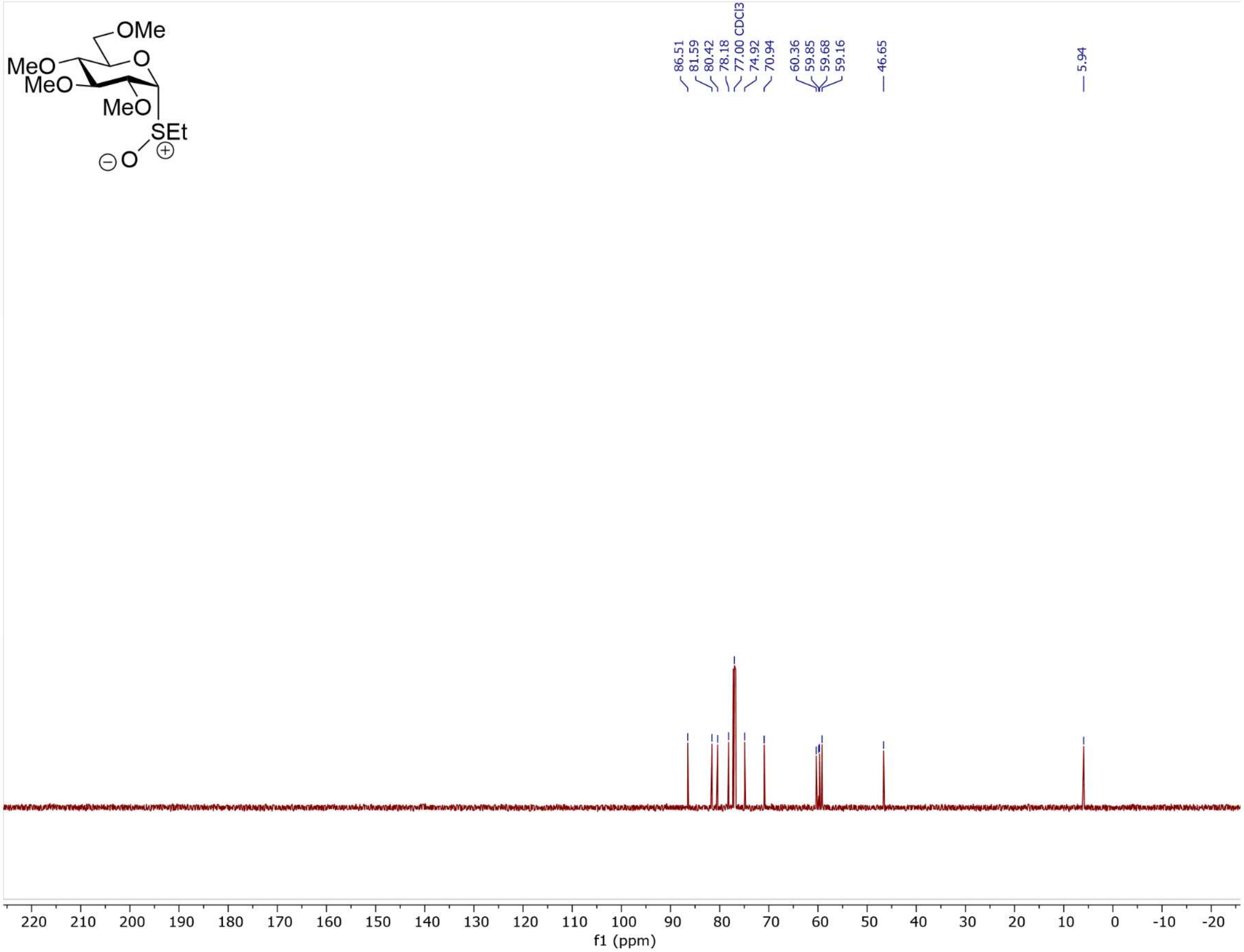

COSY (CD_2Cl_2) spectrum of ethyl 2,3,4,6-tetra-*O*-methyl-1-thio-*α*-D-glucopyranoside-1-*S*-oxide (55):

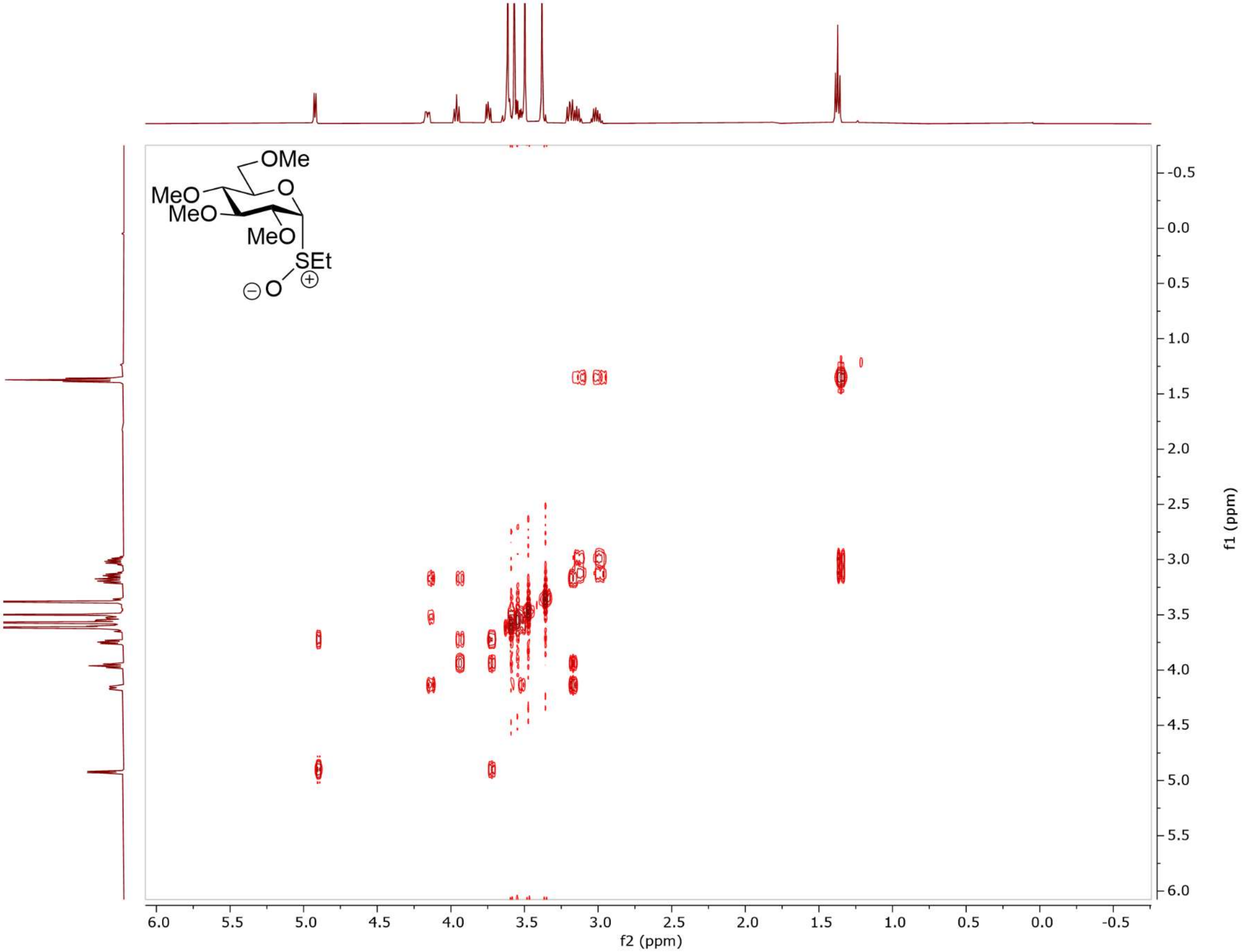

HSQC (CD_2Cl_2) spectrum of ethyl 2,3,4,6-tetra-*O*-methyl-1-thio-*α*-D-glucopyranoside-1-*S*-oxide (55):

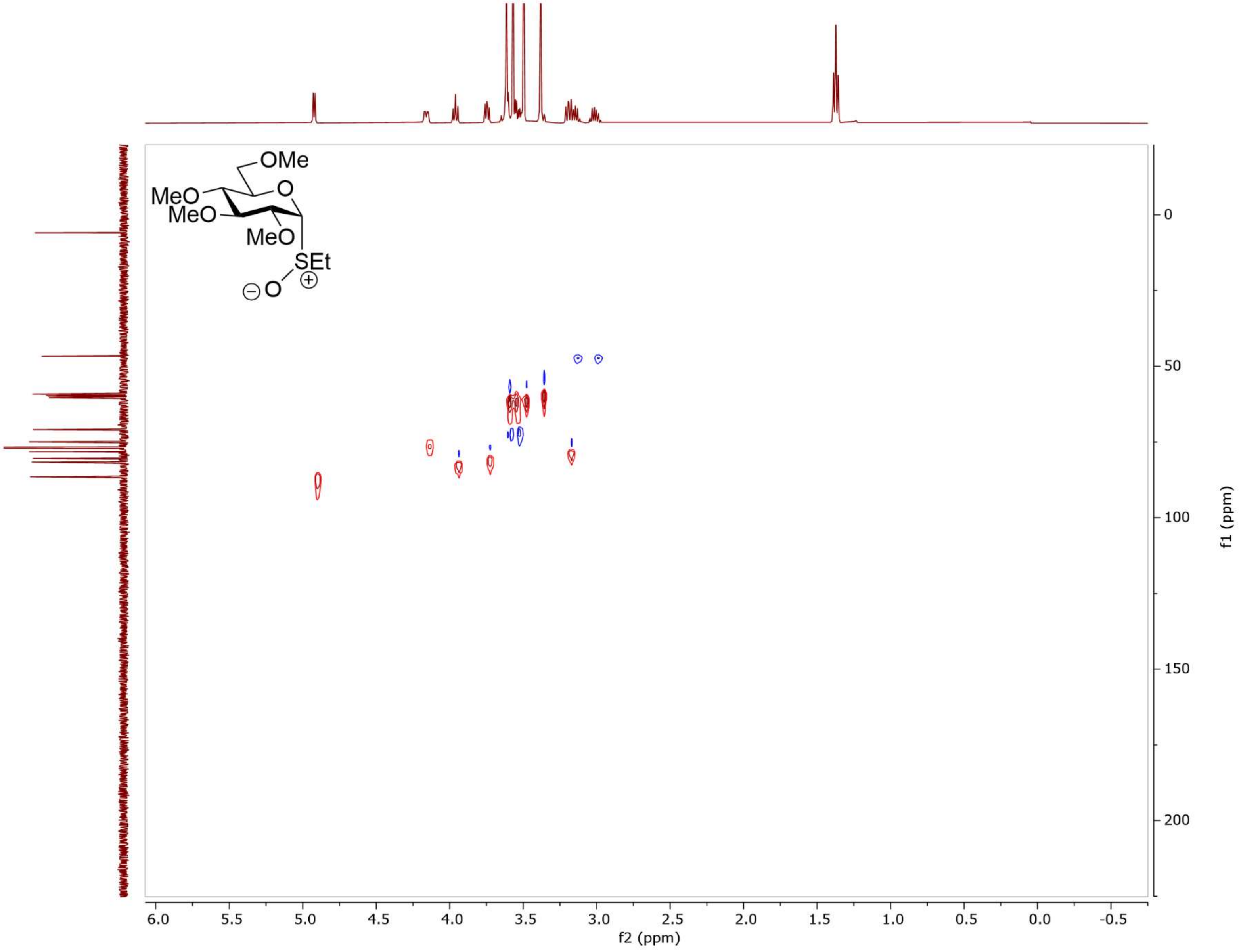

¹H NMR (500 MHz, $CDCl_3$) spectrum of 2,3,4,6-tetra-*O*-acetyl-*α*-D-glucopyranosyl trichloroacetoimidate (**57**):

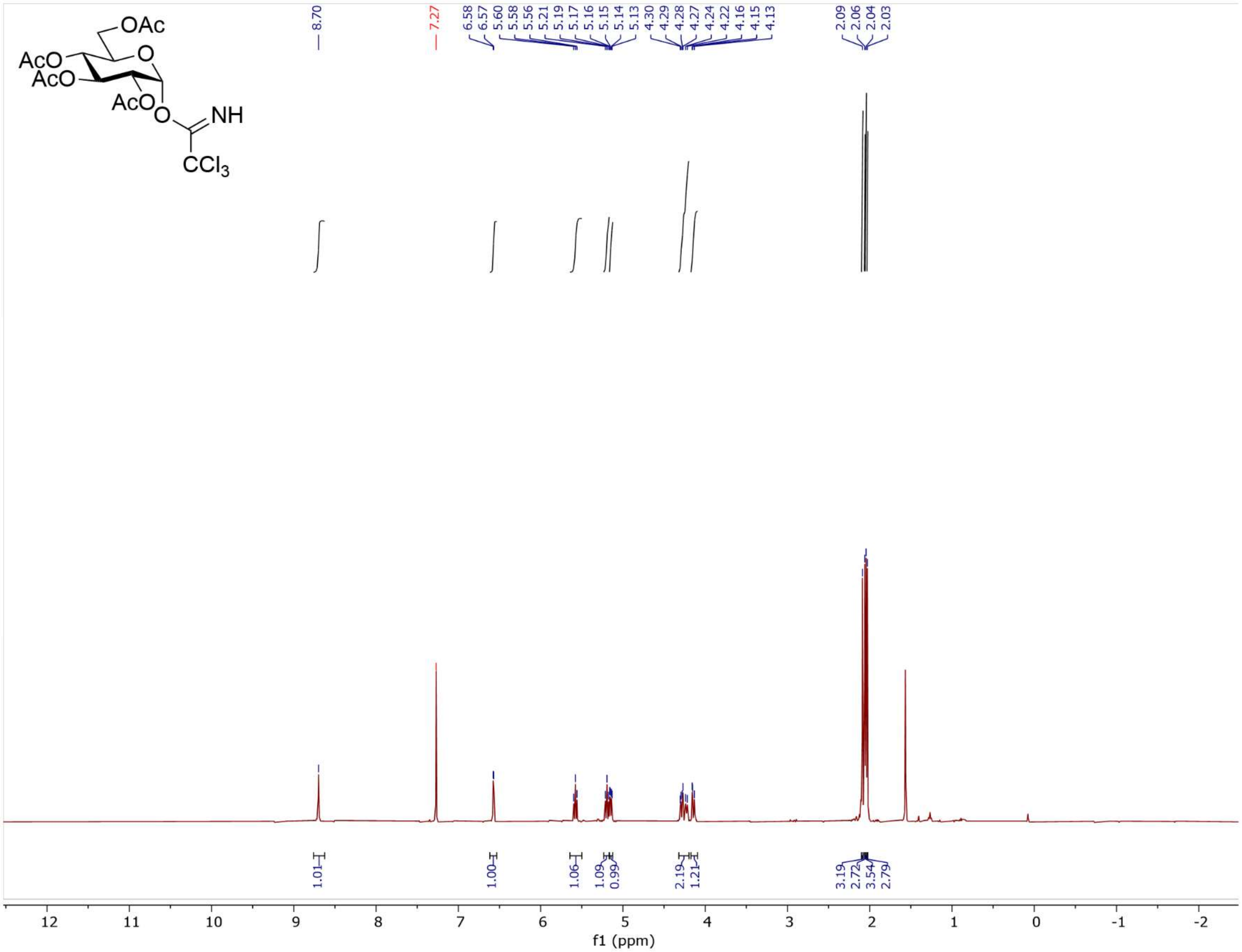

^{13}C NMR (125.67 MHz, $CDCl_3$) spectrum of 2,3,4,6-tetra-*O*-acetyl-*α*-D-glucopyranosyl trichloroacetoimidate (**57**):

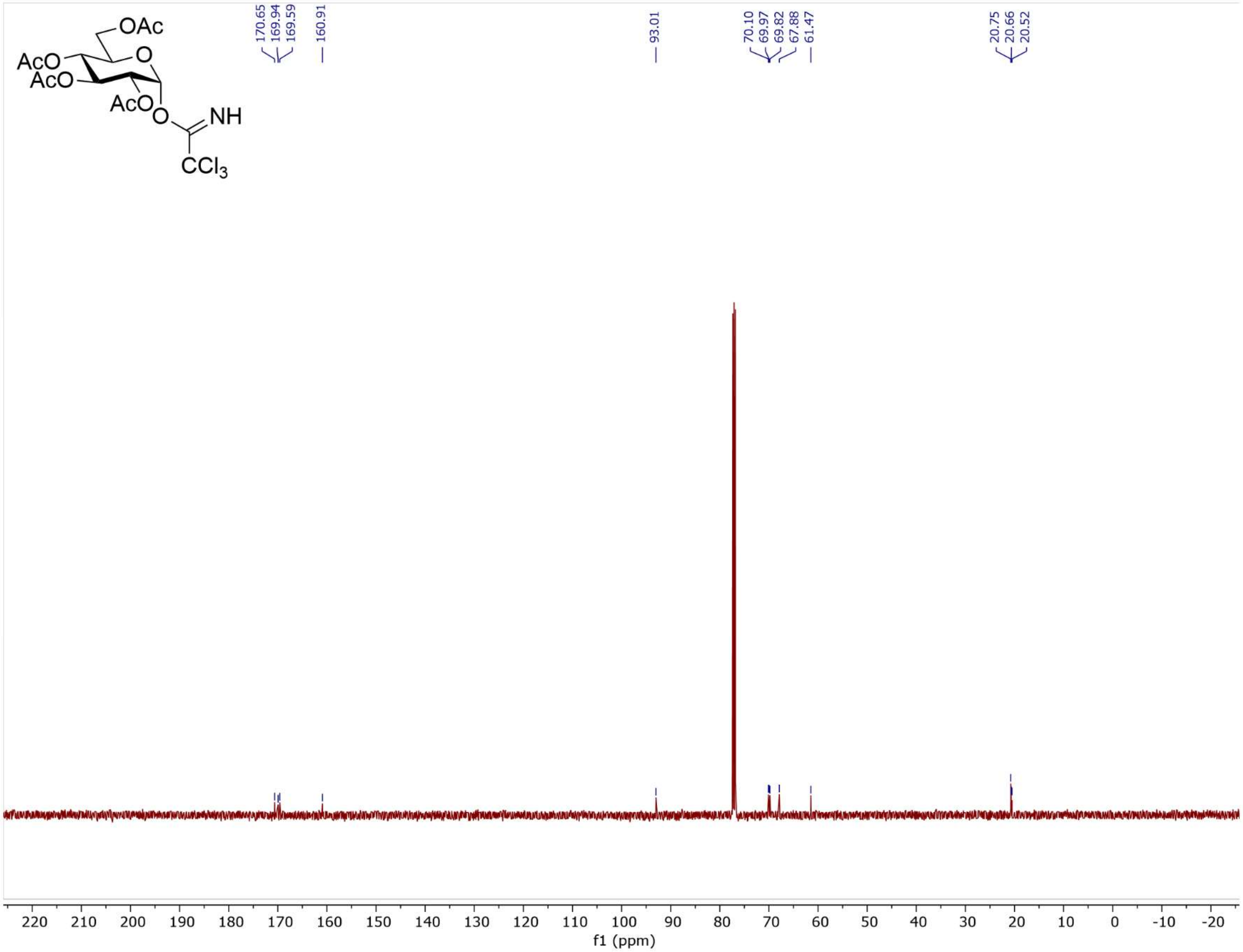

^{1}H NMR (500 MHz, $CDCl_3$) spectrum of 2,3,4,6-tetra-*O*-methyl-*α,β*-D-glucopyranosyl trichloroacetimidate (**60**):

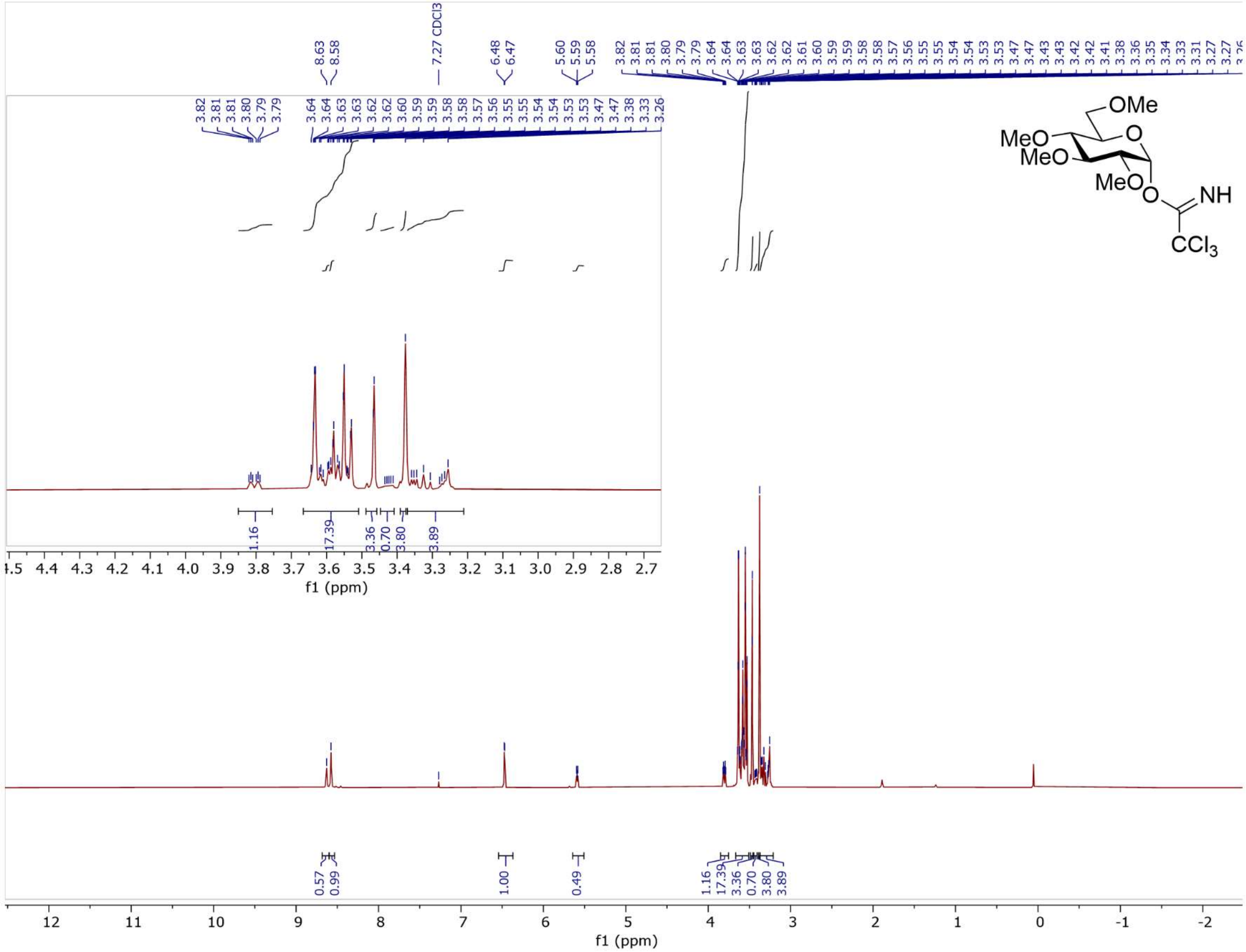

^{13}C NMR (125.67 MHz, $CDCl_3$) spectrum of 2,3,4,6-tetra-*O*-methyl-*α,β*-D-glucopyranosyl trichloroacetimidate (**60**):

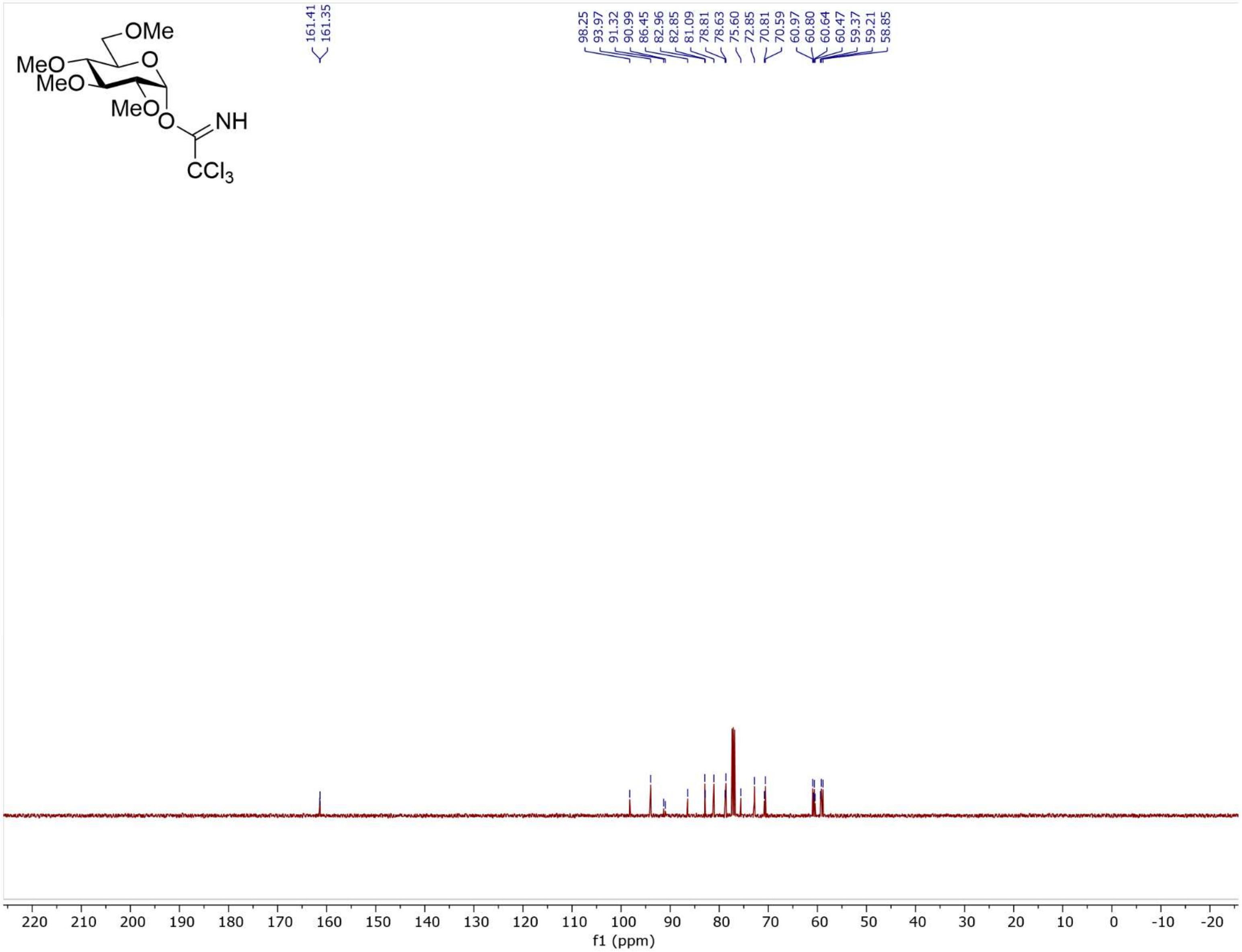

DEPT-90 ($CDCl_3$) spectrum of 2,3,4,6-tetra-*O*-methyl-*α,β*-D-glucopyranosyl trichloroacetimidate (**60**):

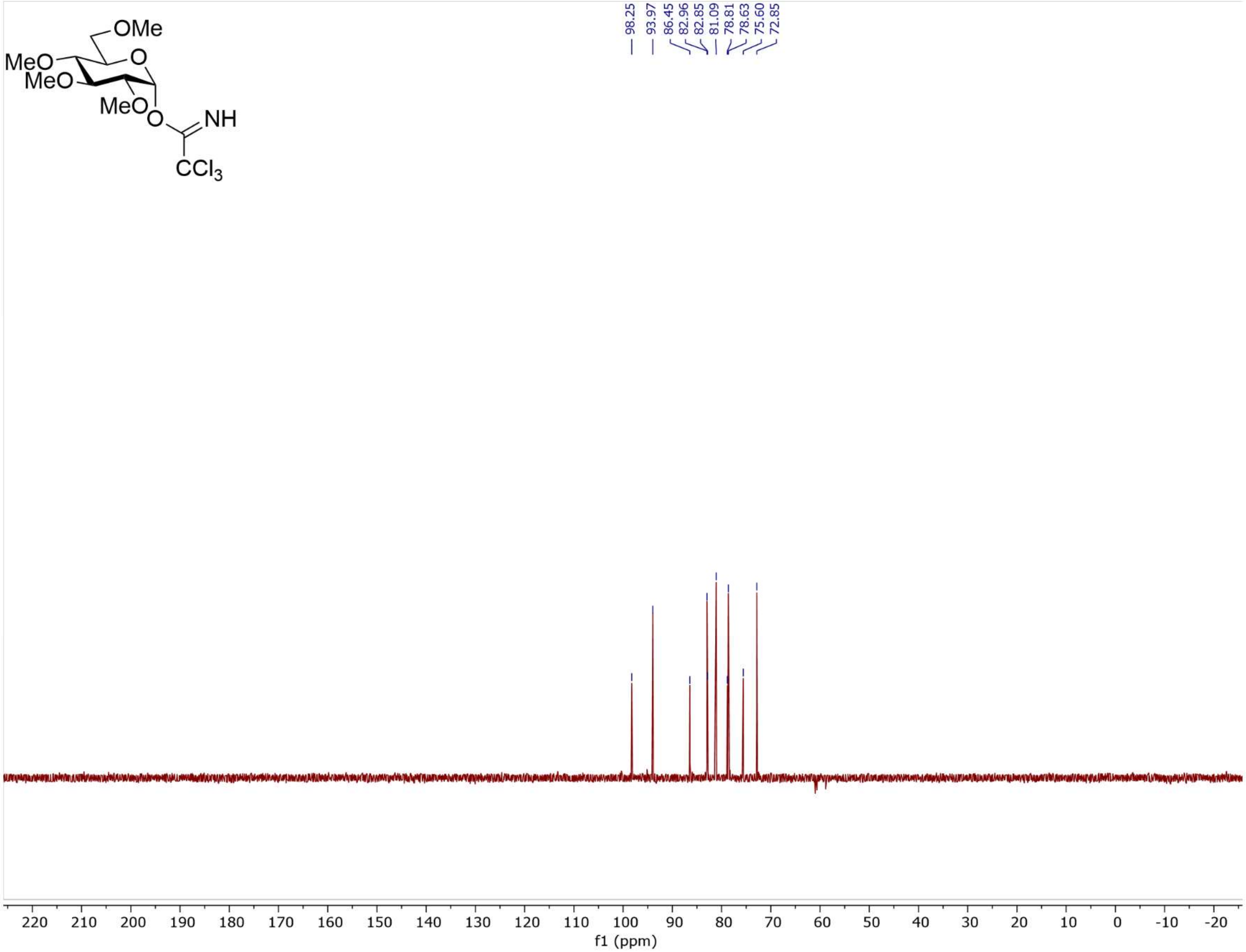

COSY ($CDCl_3$) spectrum of 2,3,4,6-tetra-*O*-methyl-*α,β*-D-glucopyranosyl trichloroacetimidate (**60**):

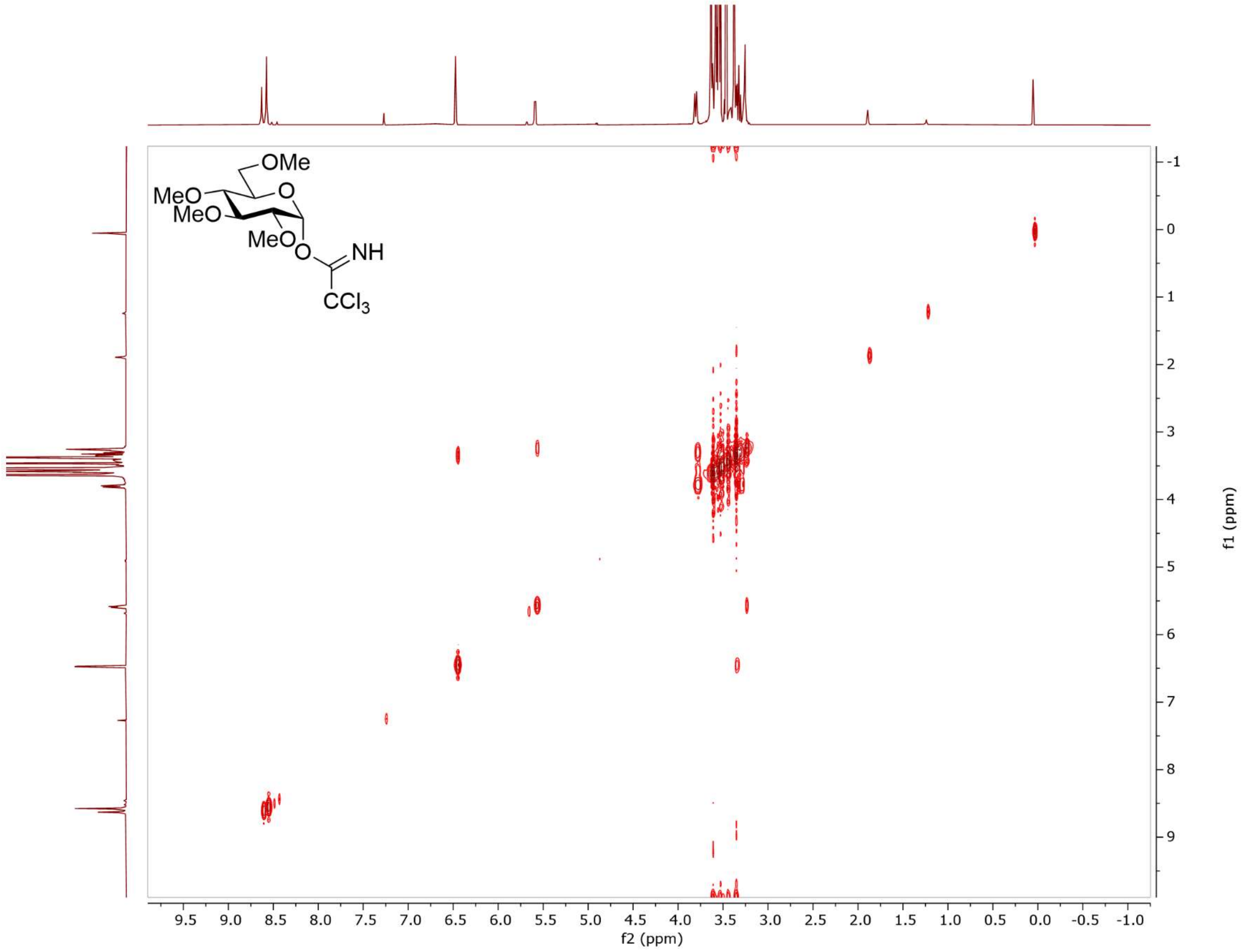

HSQC ($CDCl_3$) spectrum of 2,3,4,6-tetra-*O*-methyl-*α,β*-D-glucopyranosyl trichloroacetimidate (**60**):

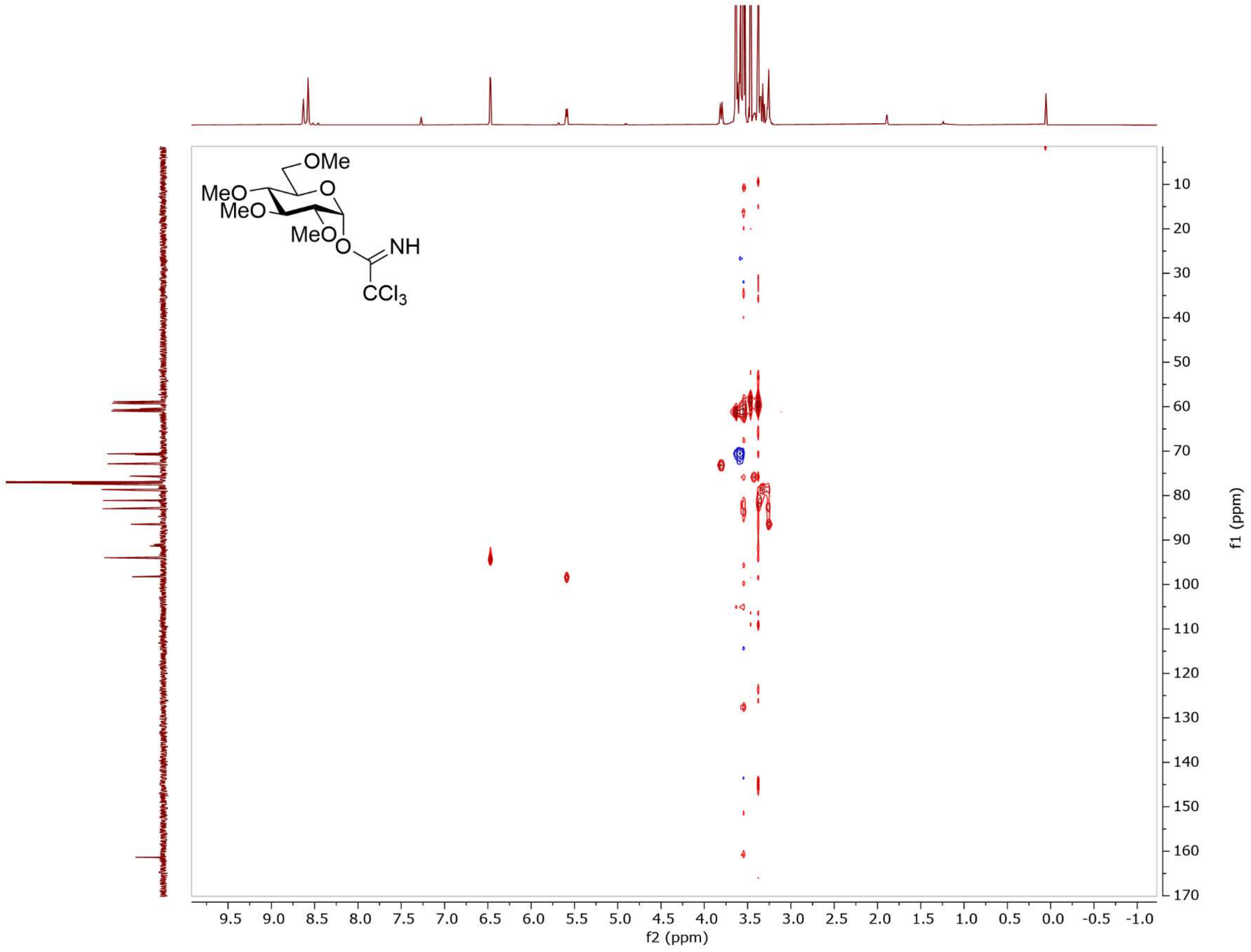

^{1}H NMR (500 MHz, $CDCl_3$) spectrum of 2,3,4,6-tetra-*O*-acetyl-5-thio-*α*,*β*-D-glucopyranosyl trichloroacetoimidate (**1**):

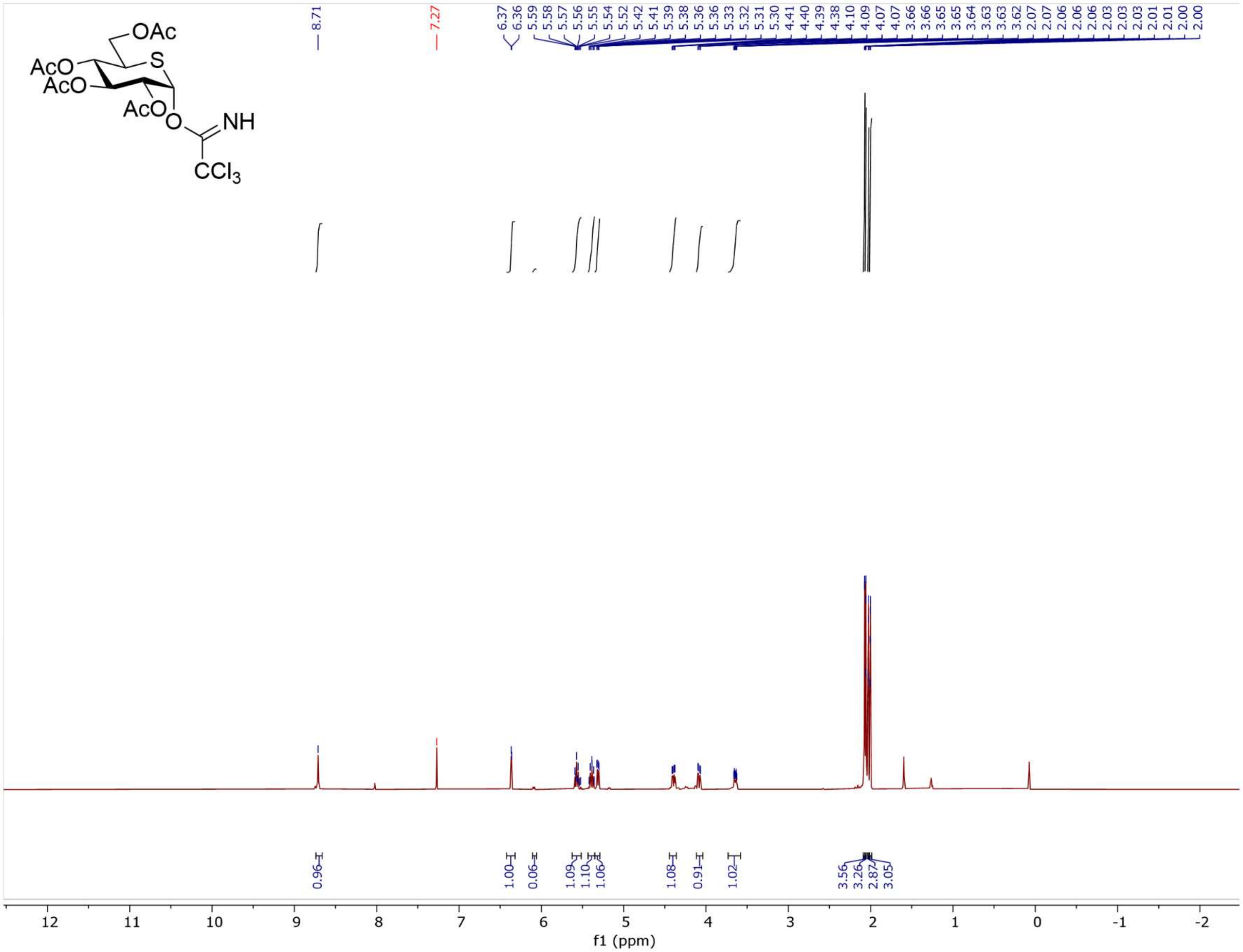

^{13}C NMR (125.67 MHz, $CDCl_3$) spectrum of 2,3,4,6-tetra-*O*-acetyl-5-thio-*α*,*β*-D-glucopyranosyl trichloroacetoimidate (**1**):

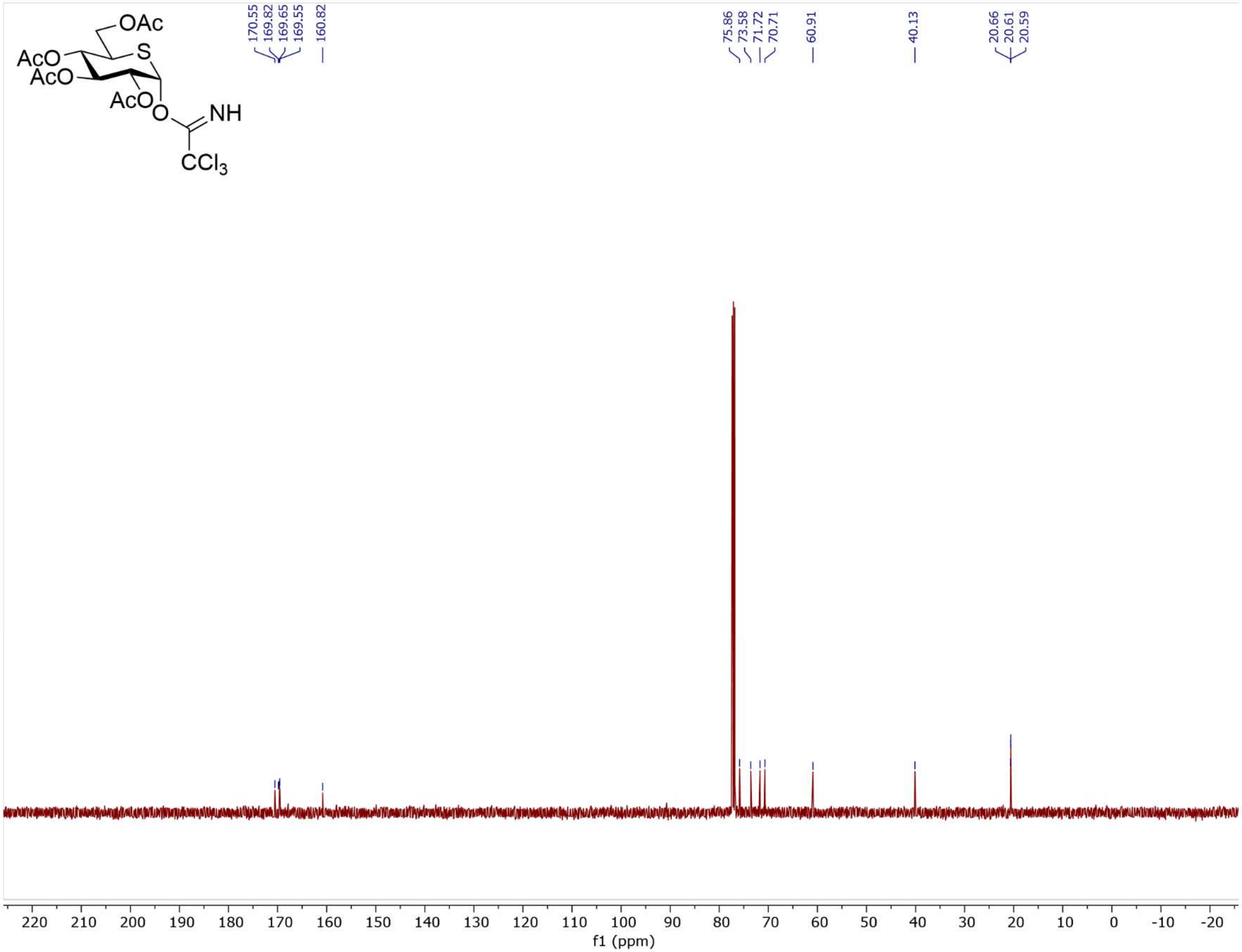

^{1}H NMR (500 MHz, CD_2Cl_2) spectrum of 2,3,4,6-tetra-*O*-methyl-5-thio-*α*,*β*-D-glucopyranoside (**63**):

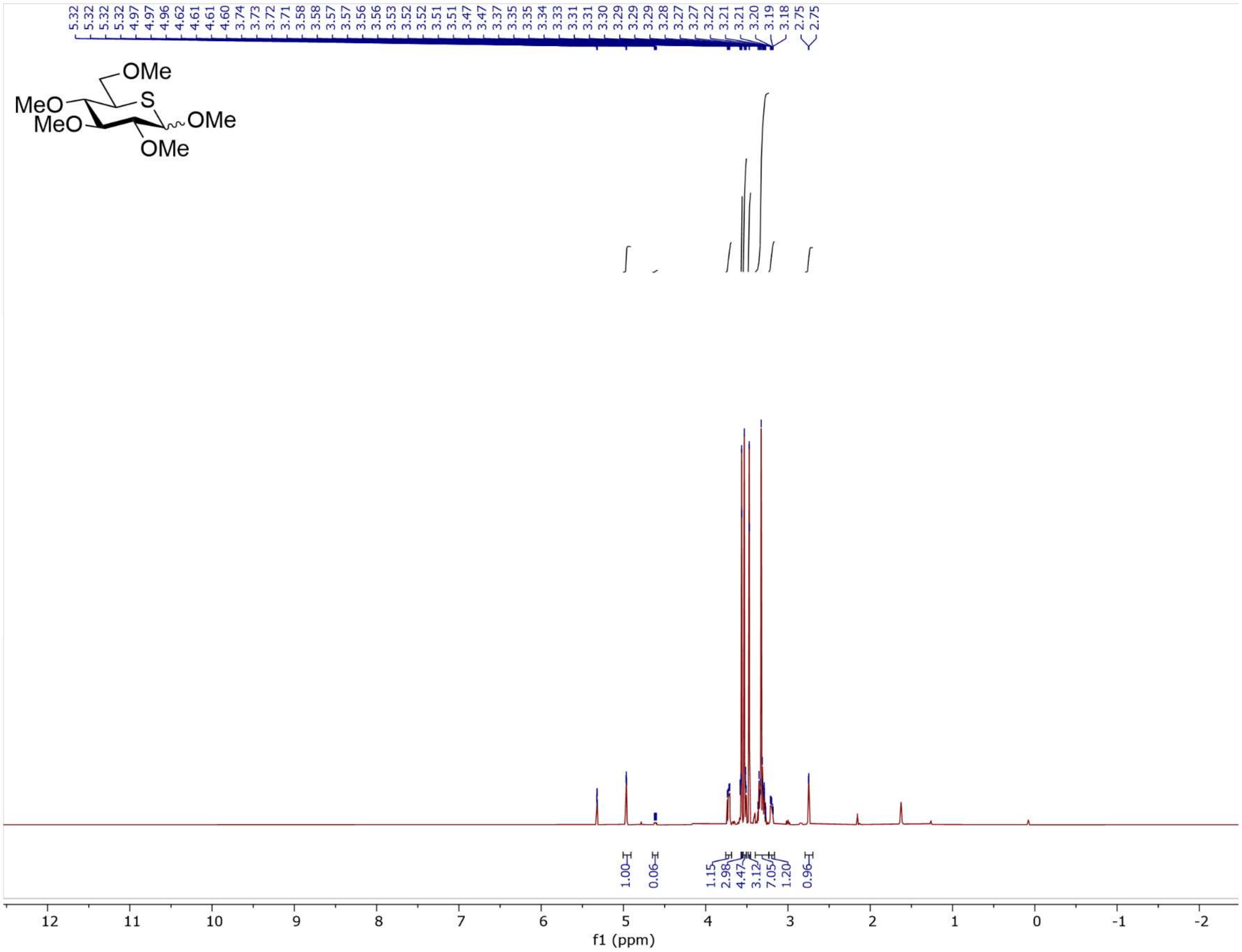

^{13}C NMR (125.67 MHz, CD_2Cl_2) spectrum of 2,3,4,6-tetra-*O*-methyl-5-thio-*α*,*β*-D-glucopyranoside (**63**):

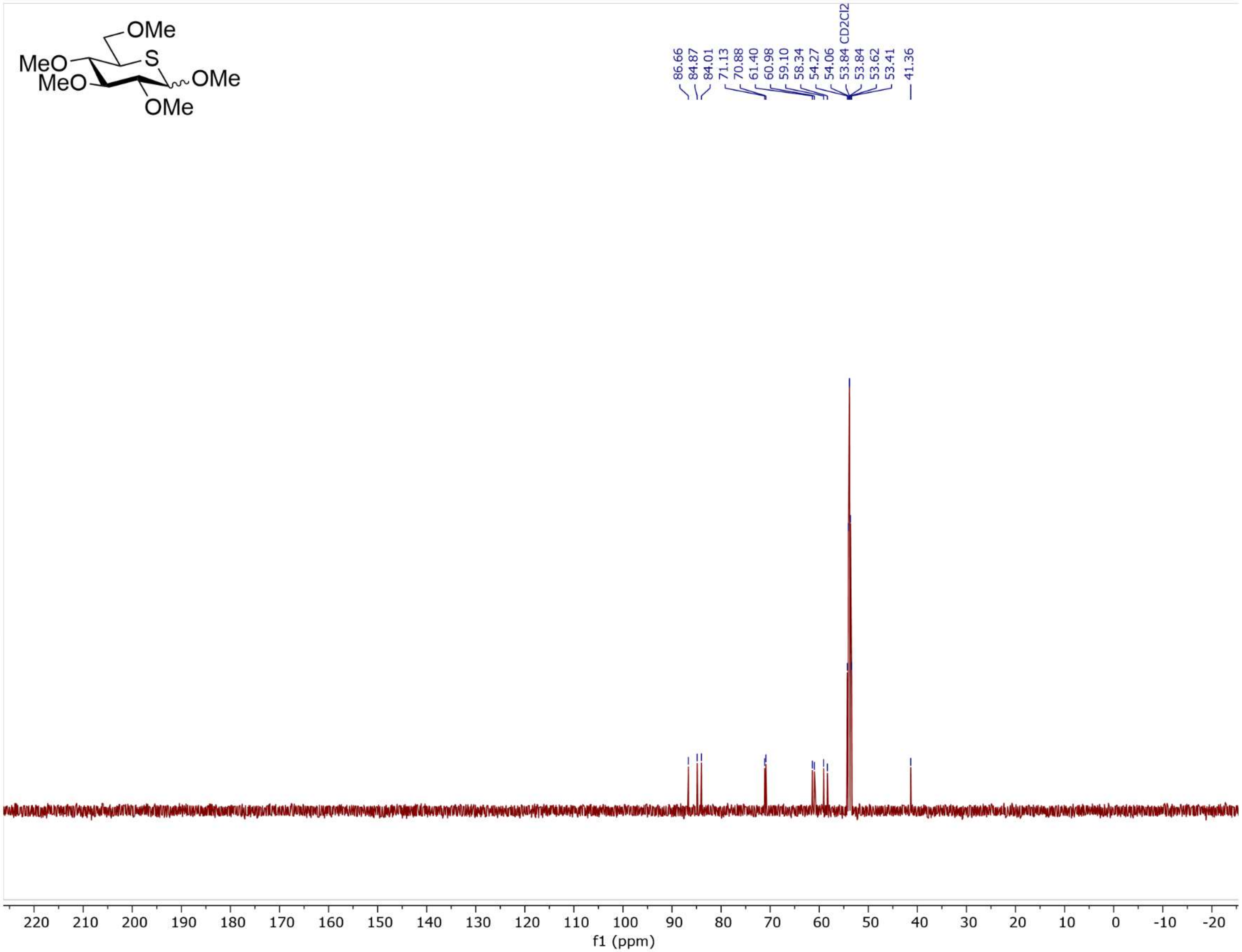

DEPT-90 (CD_2Cl_2) spectrum of 2,3,4,6-tetra-*O*-methyl-5-thio-*α*,*β*-D-glucopyranoside (**63**):

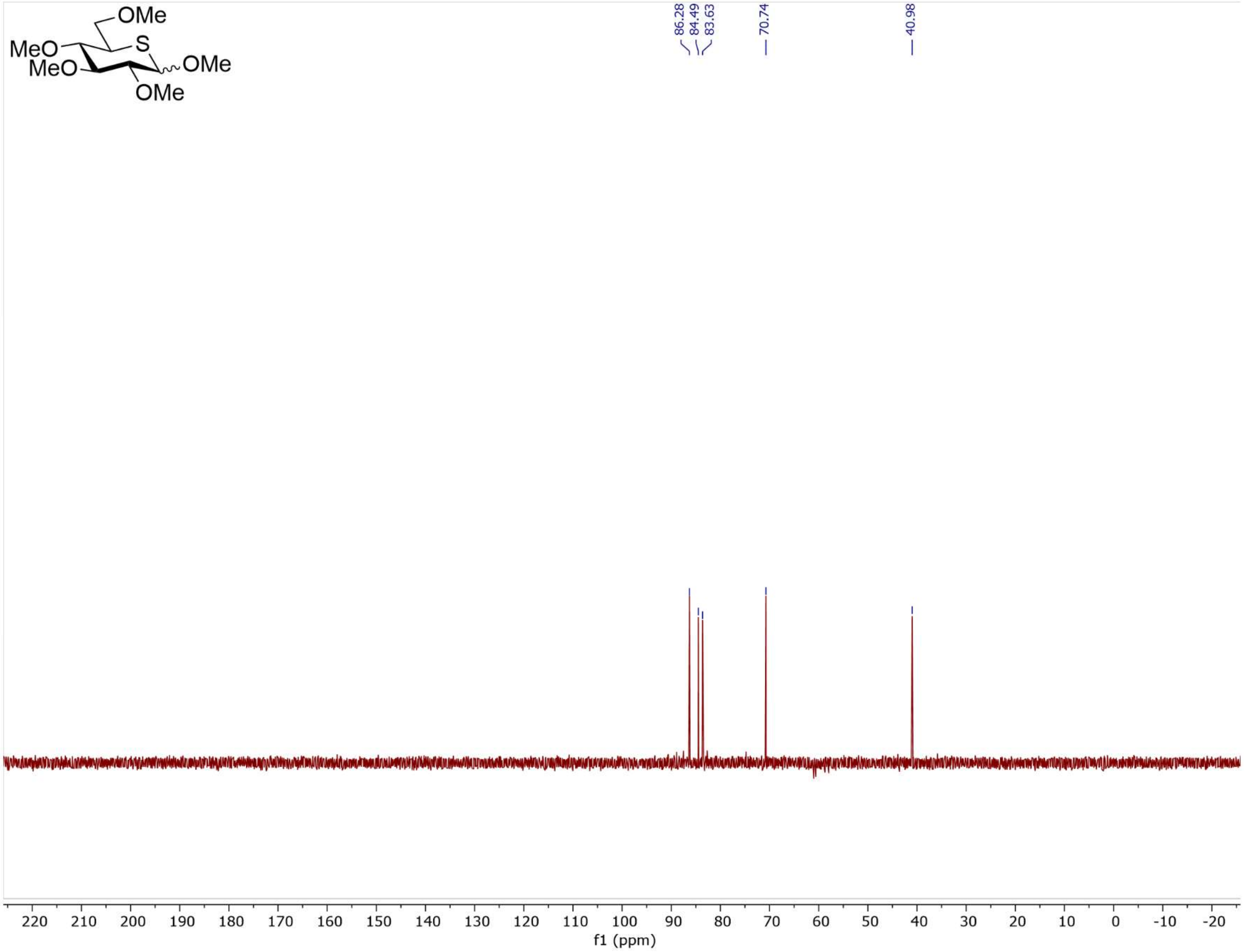

COSY (CD_2Cl_2) spectrum of 2,3,4,6-tetra-*O*-methyl-5-thio-*α*,*β*-D-glucopyranoside (**63**):

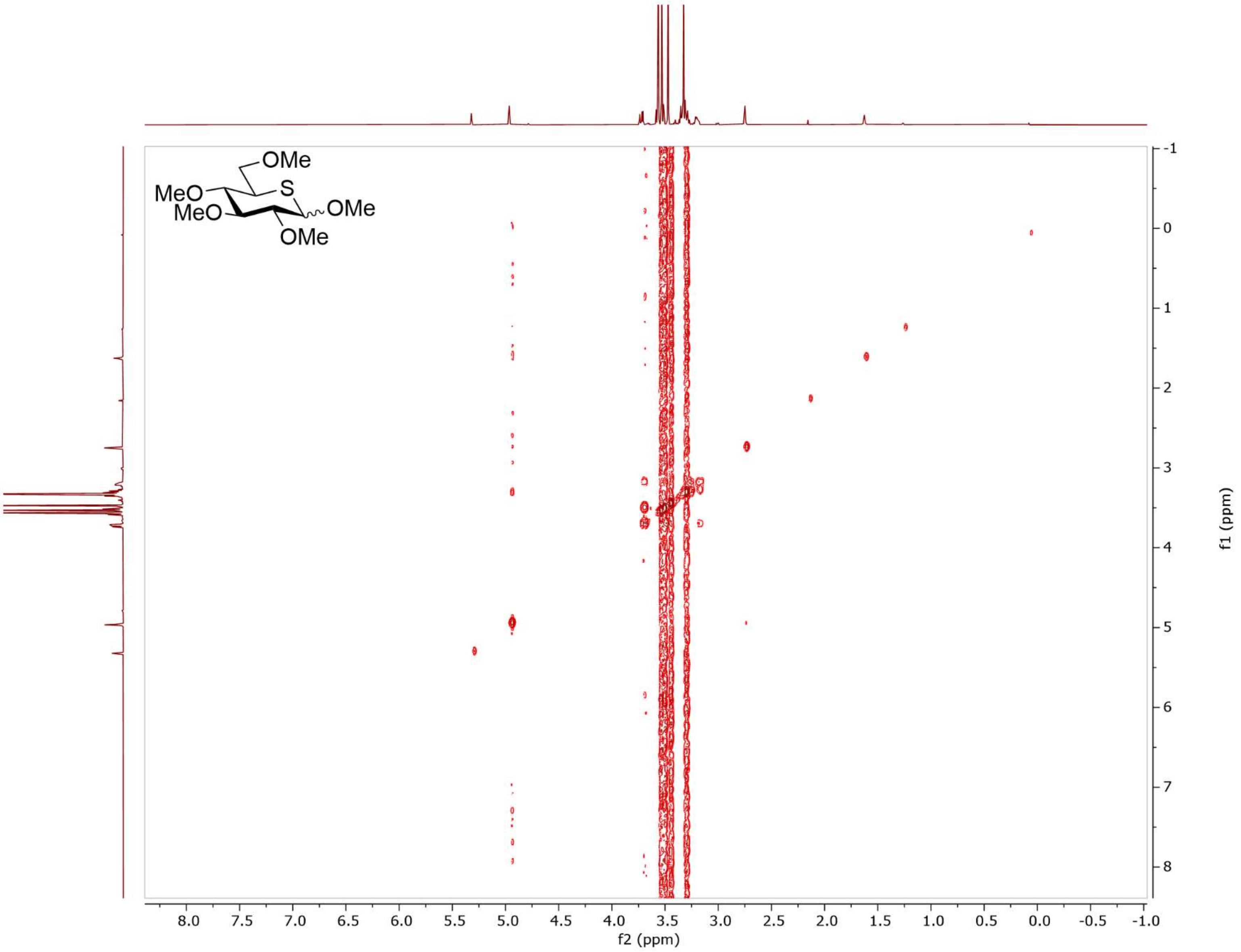

HSQC (CD_2Cl_2) spectrum of 2,3,4,6-tetra-*O*-methyl-5-thio-*α*,*β*-D-glucopyranoside (**63**):

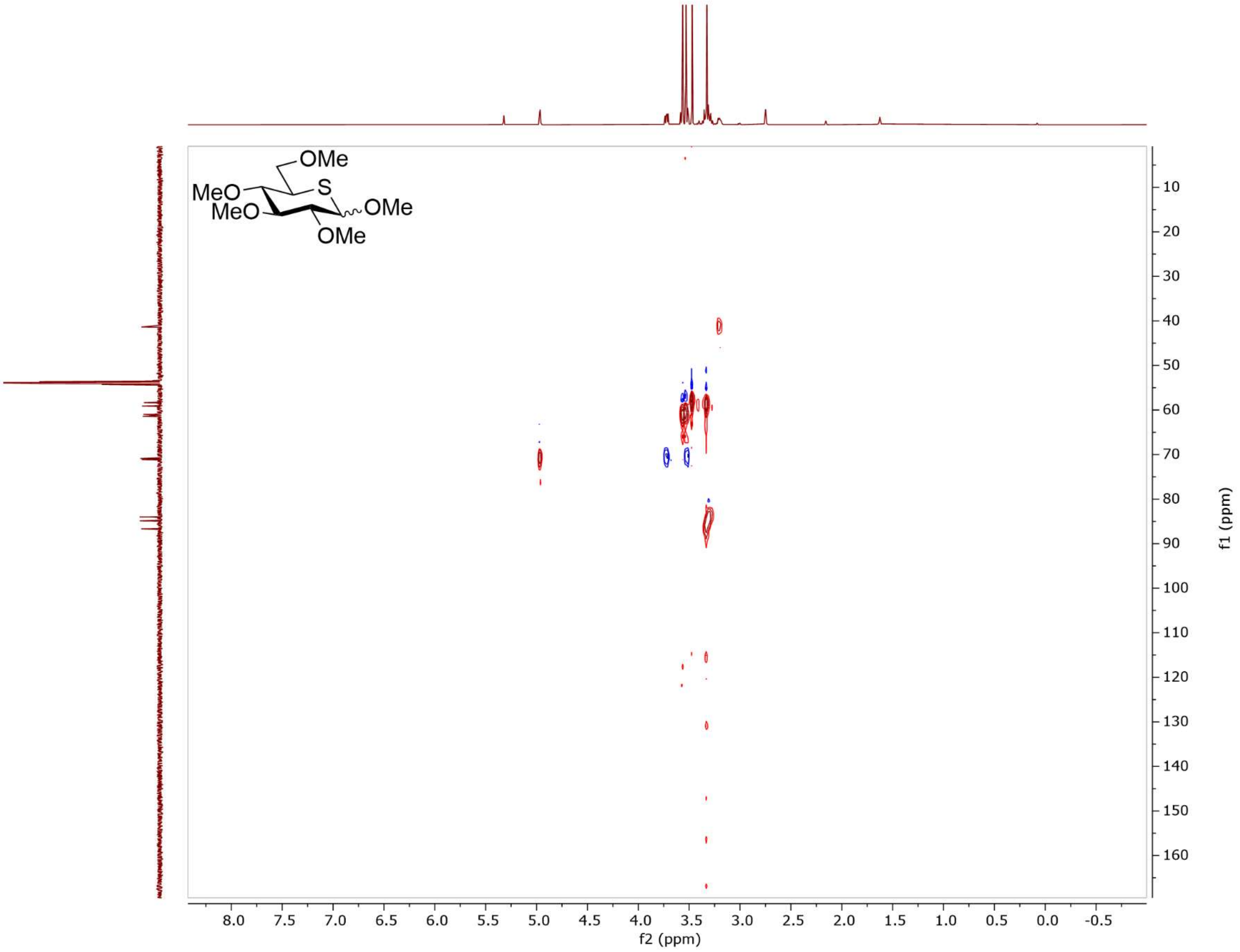

^{1}H NMR (500 MHz, $CDCl_3$) spectrum of 2,3,4,6-tetra-*O*-methyl-5-thio-*α*-D-glucopyranosyl trichloroacetoimidate (**64**):

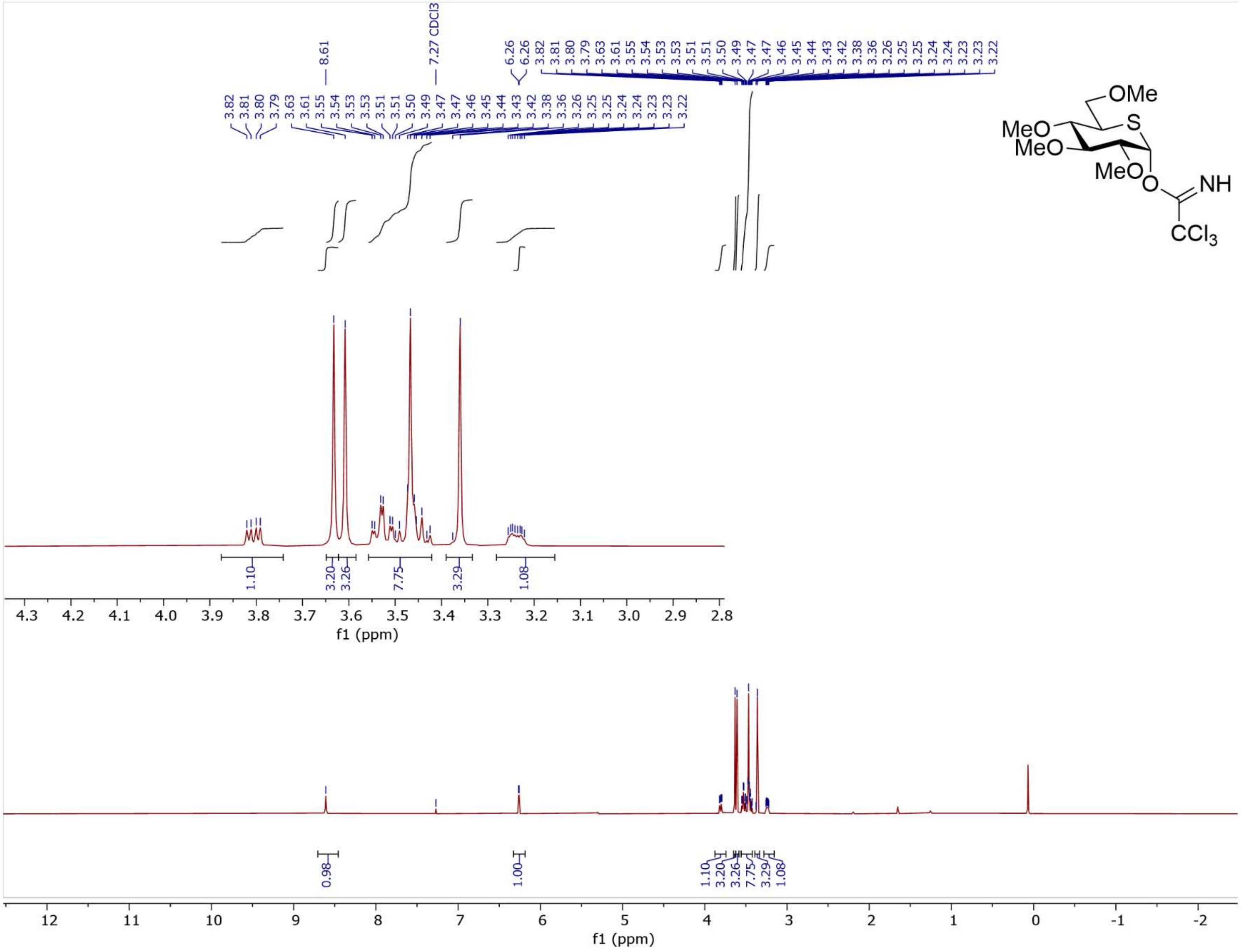

^{13}**C NMR** (125.67 MHz, $CDCl_3$) spectrum of 2,3,4,6-tetra-*O*-methyl-5-thio-*α*-D-glucopyranosyl trichloroacetoimidate (**64**):

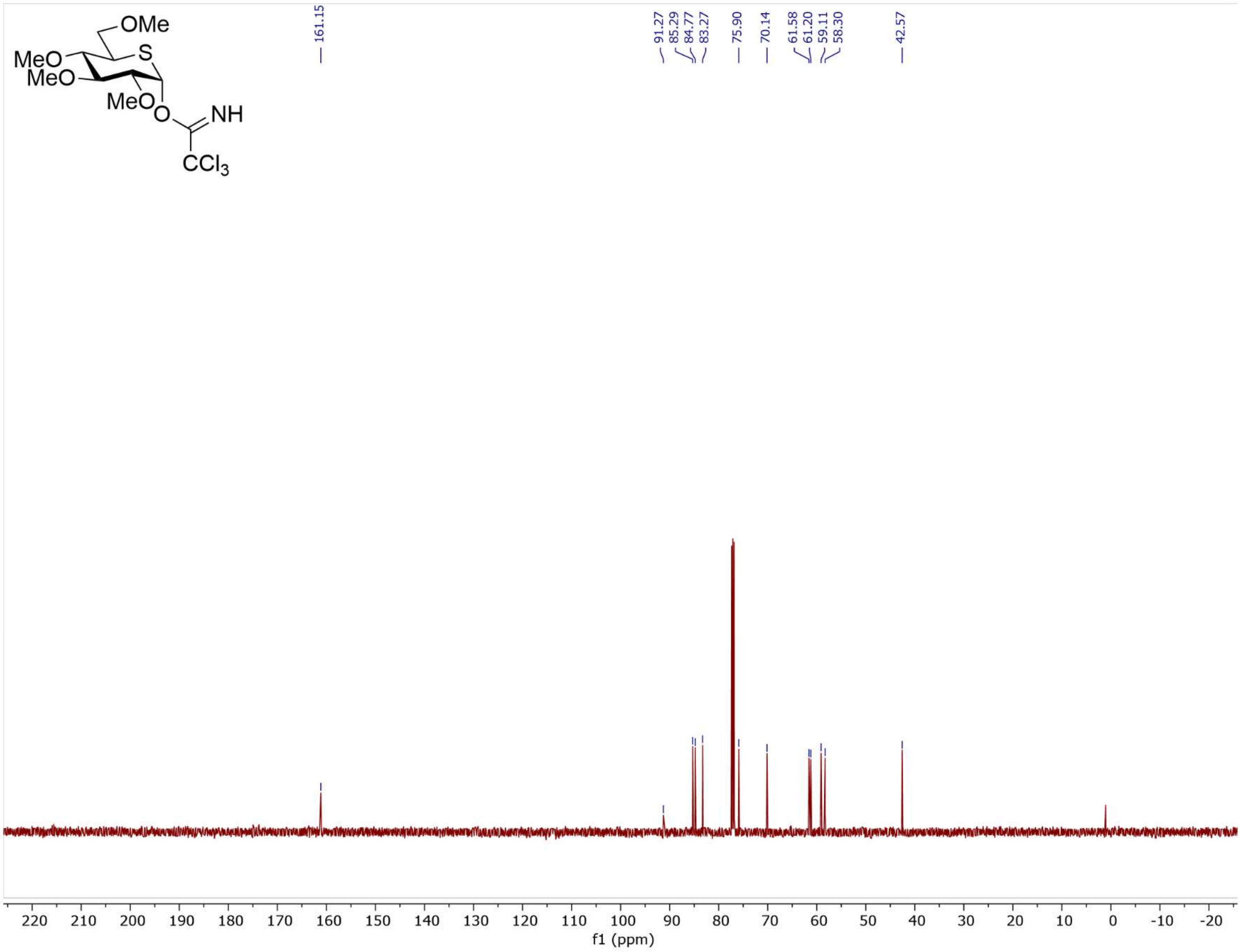

DEPT-90 ($CDCl_3$) spectrum of 2,3,4,6-tetra-*O*-methyl-5-thio-*α*-D-glucopyranosyl trichloroacetoimidate (**64**):

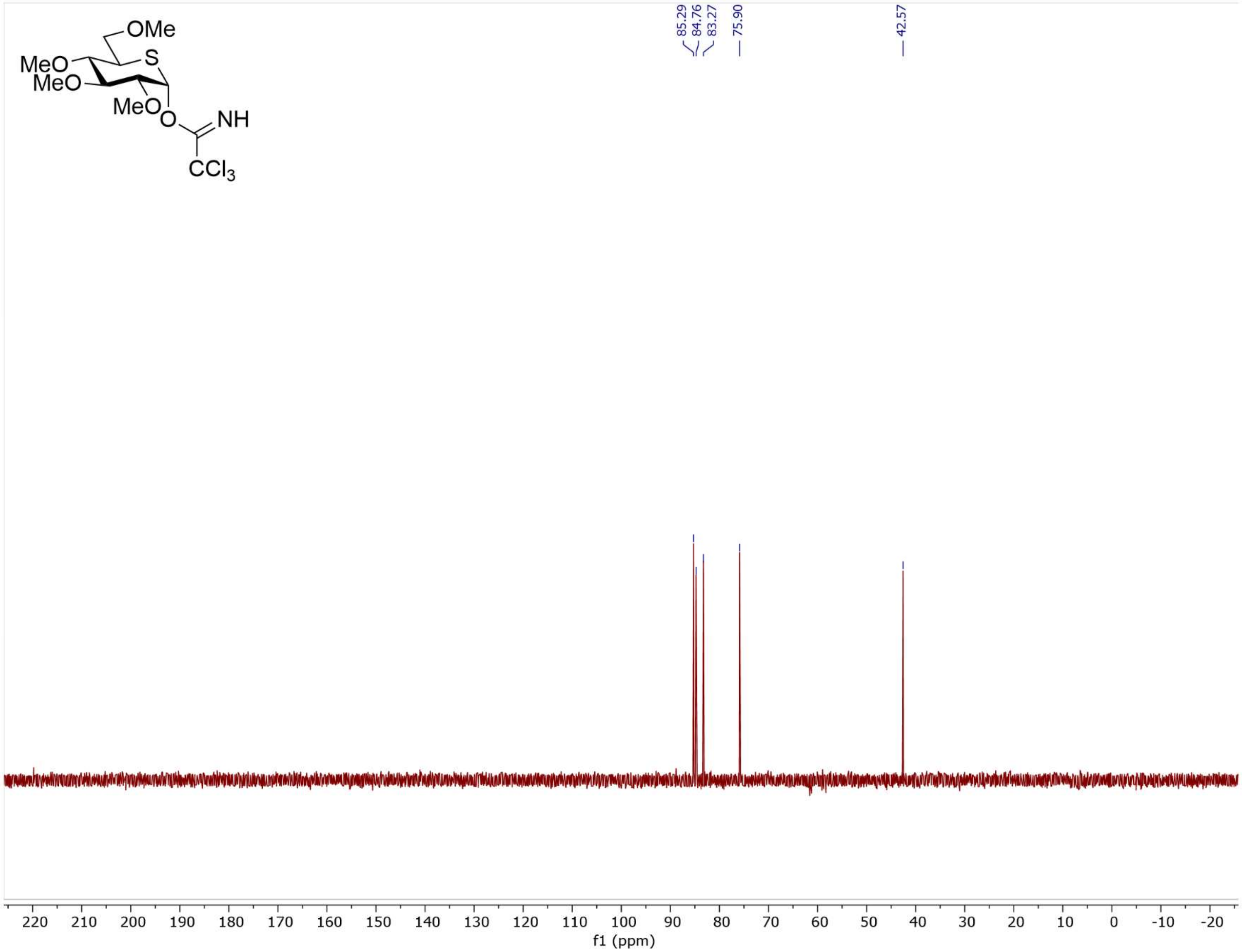

COSY ($CDCl_3$) spectrum of 2,3,4,6-tetra-*O*-methyl-5-thio-*α*-D-glucopyranosyl trichloroacetoimidate (**64**):

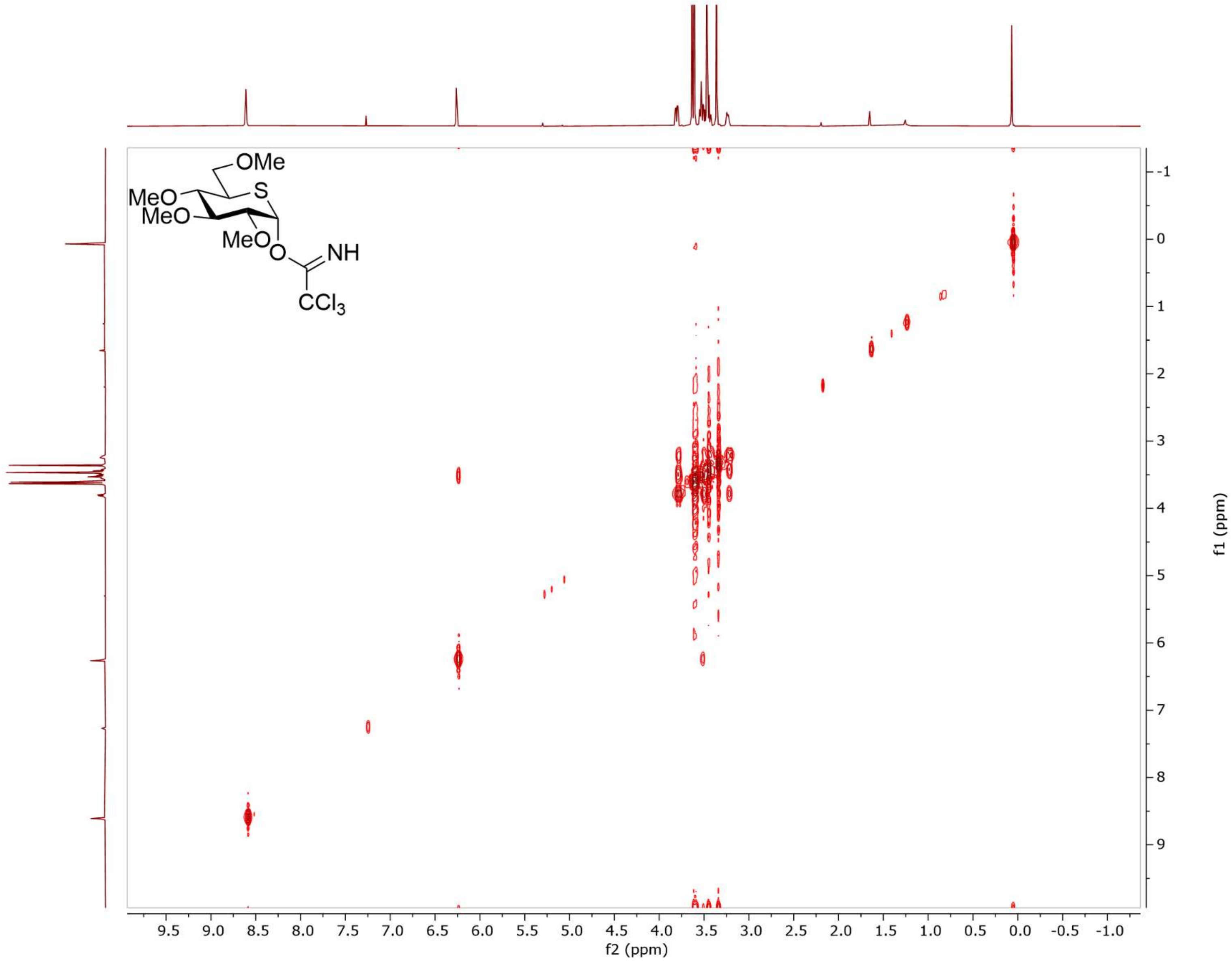

HSQC ($CDCl_3$) spectrum of 2,3,4,6-tetra-*O*-methyl-5-thio-*α*-D-glucopyranosyl trichloroacetoimidate (**64**):

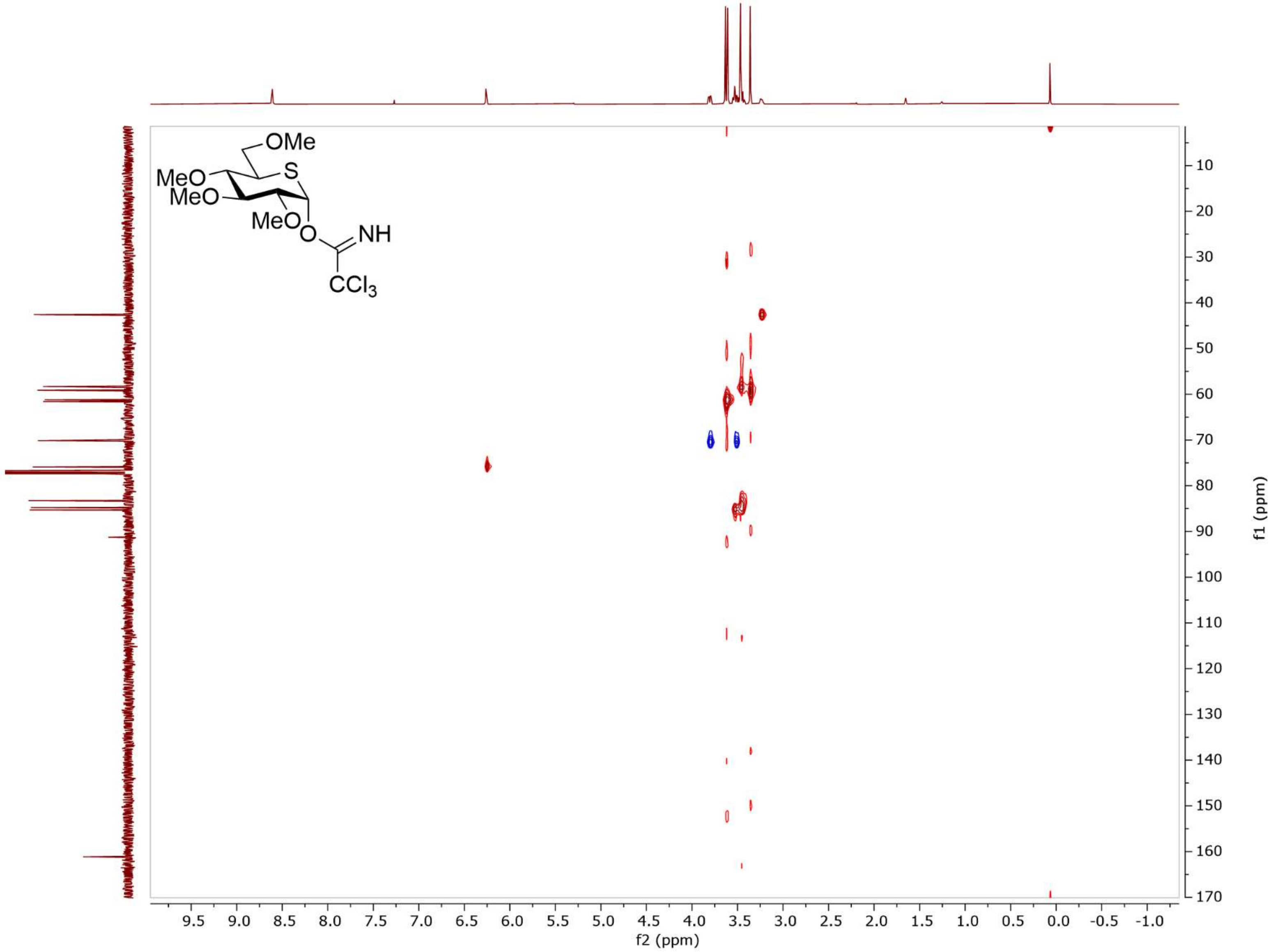

^{1}H NMR (500 MHz, CDCl$_3$) spectrum of mixture of decomposition products 11 and 69:

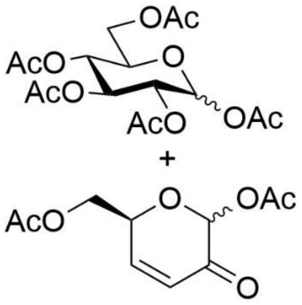

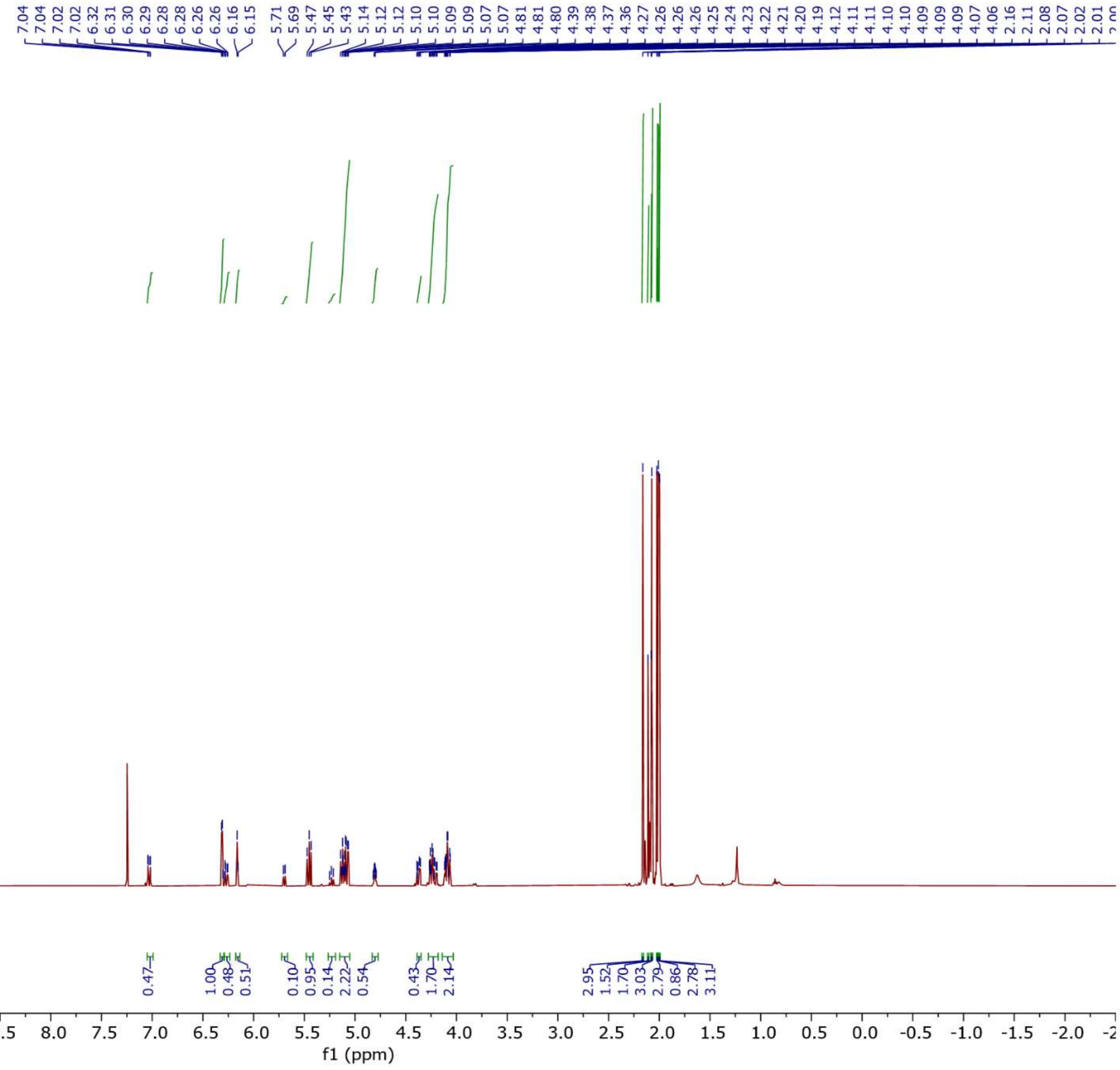

^{13}C NMR (125.67 MHz, $CDCl_3$) spectrum of decomposition products 11 and 69:

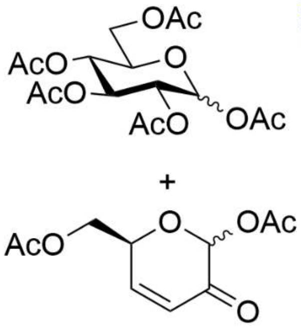

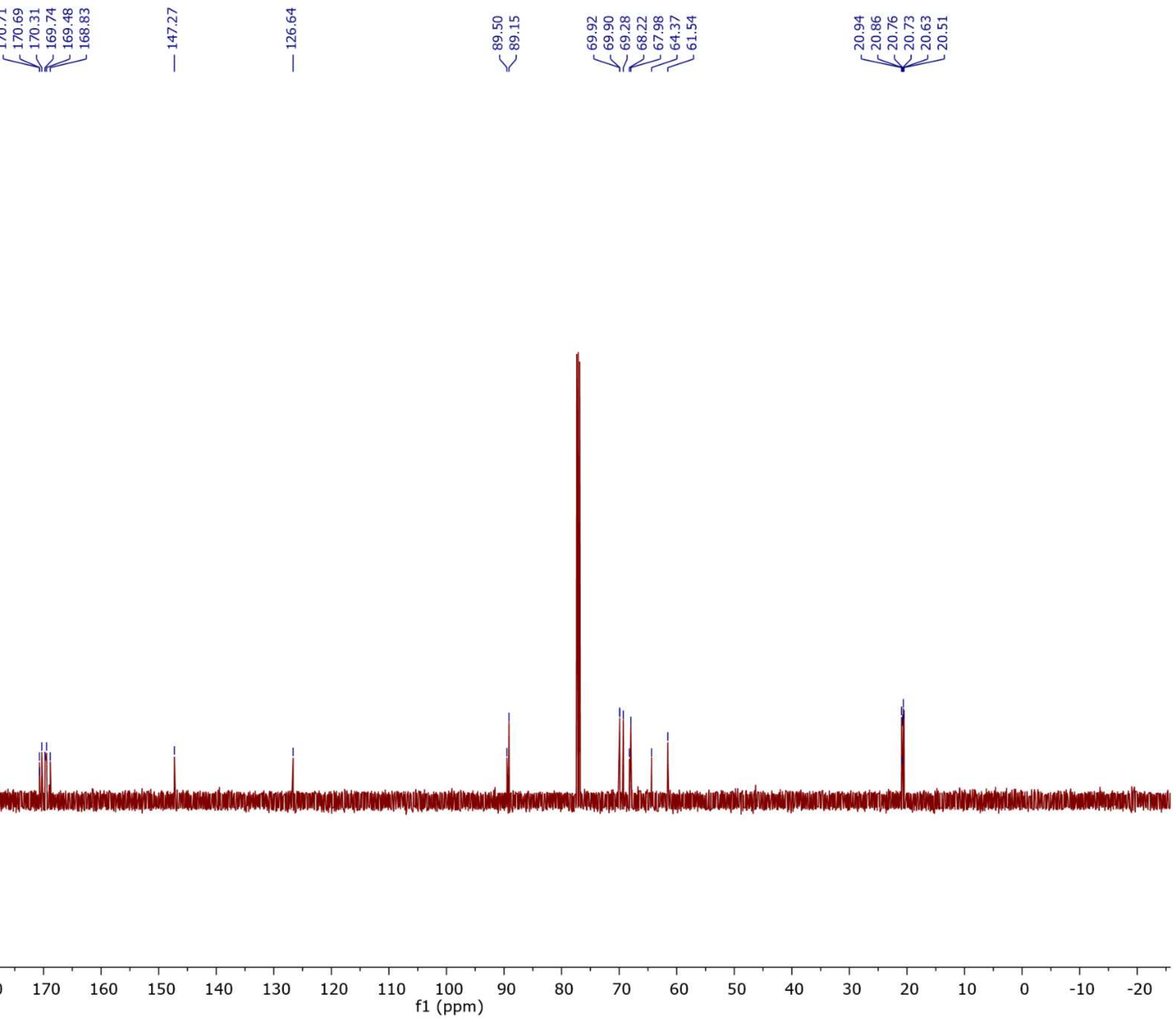

COSY ($CDCl_3$) spectrum of decomposition products 11 and 69:

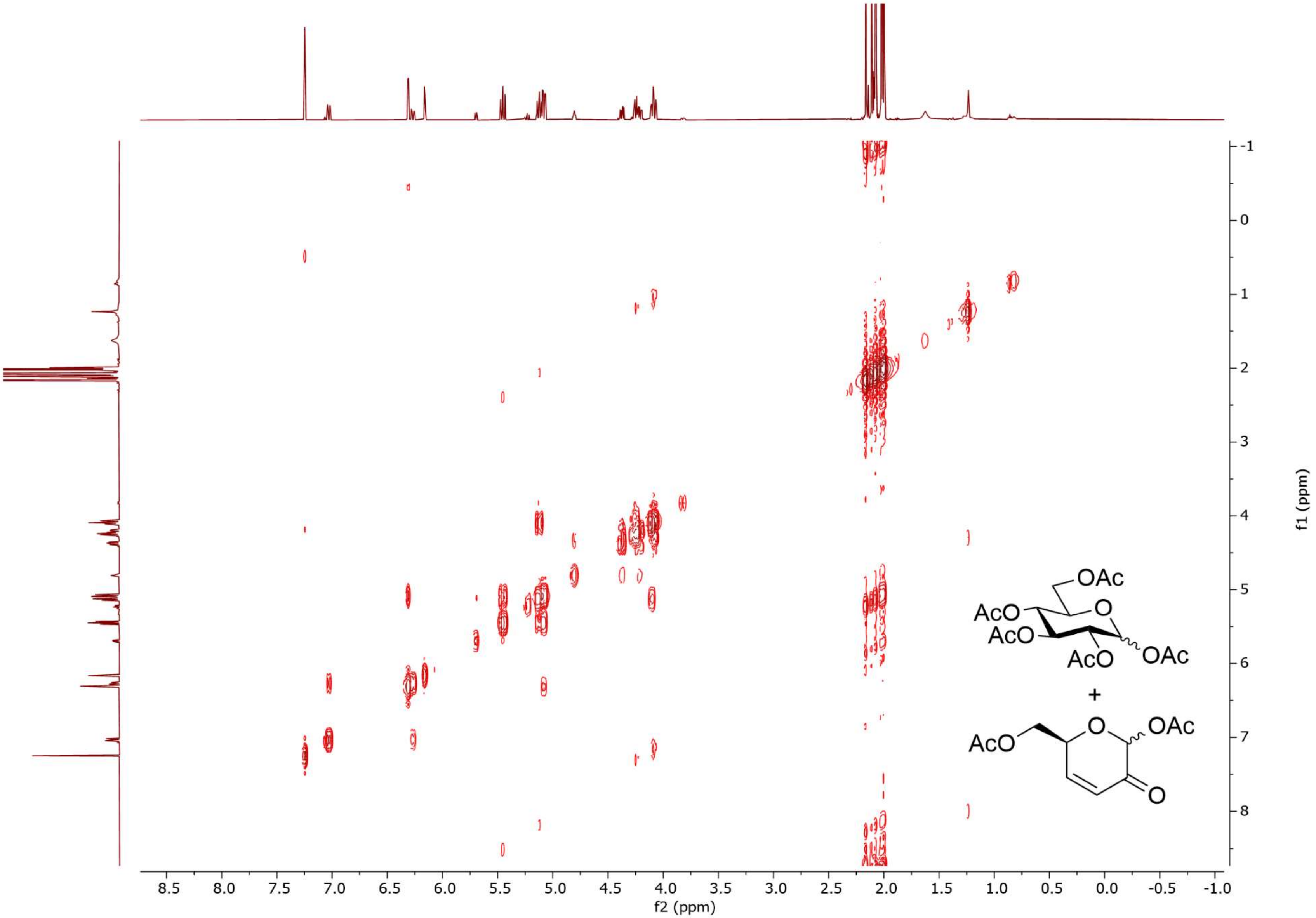

HMQC ($CDCl_3$) spectrum of decomposition products 11 and 69:

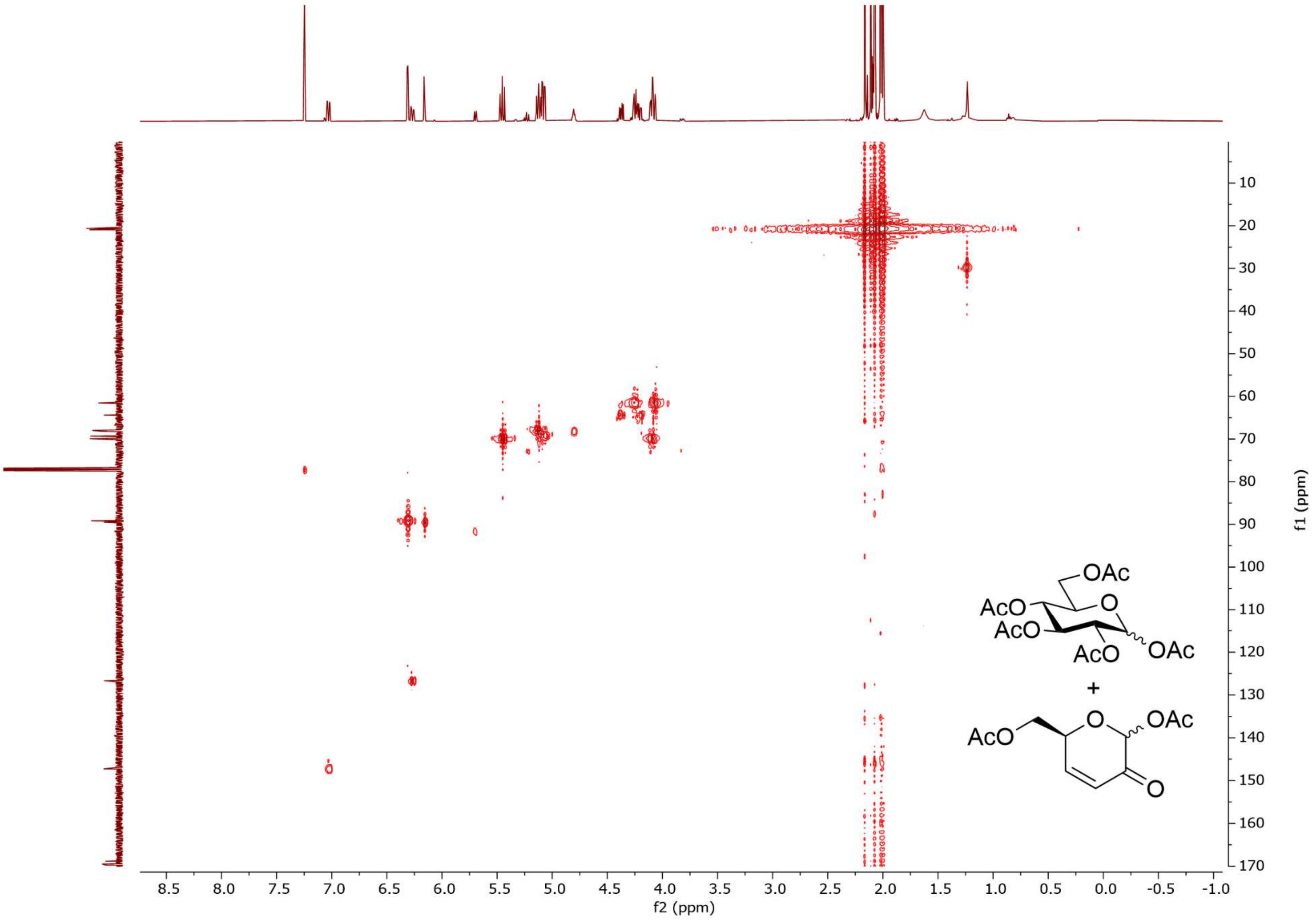

^{1}H NMR (500 MHz, $CDCl_3$) spectrum of decomposition product 9:

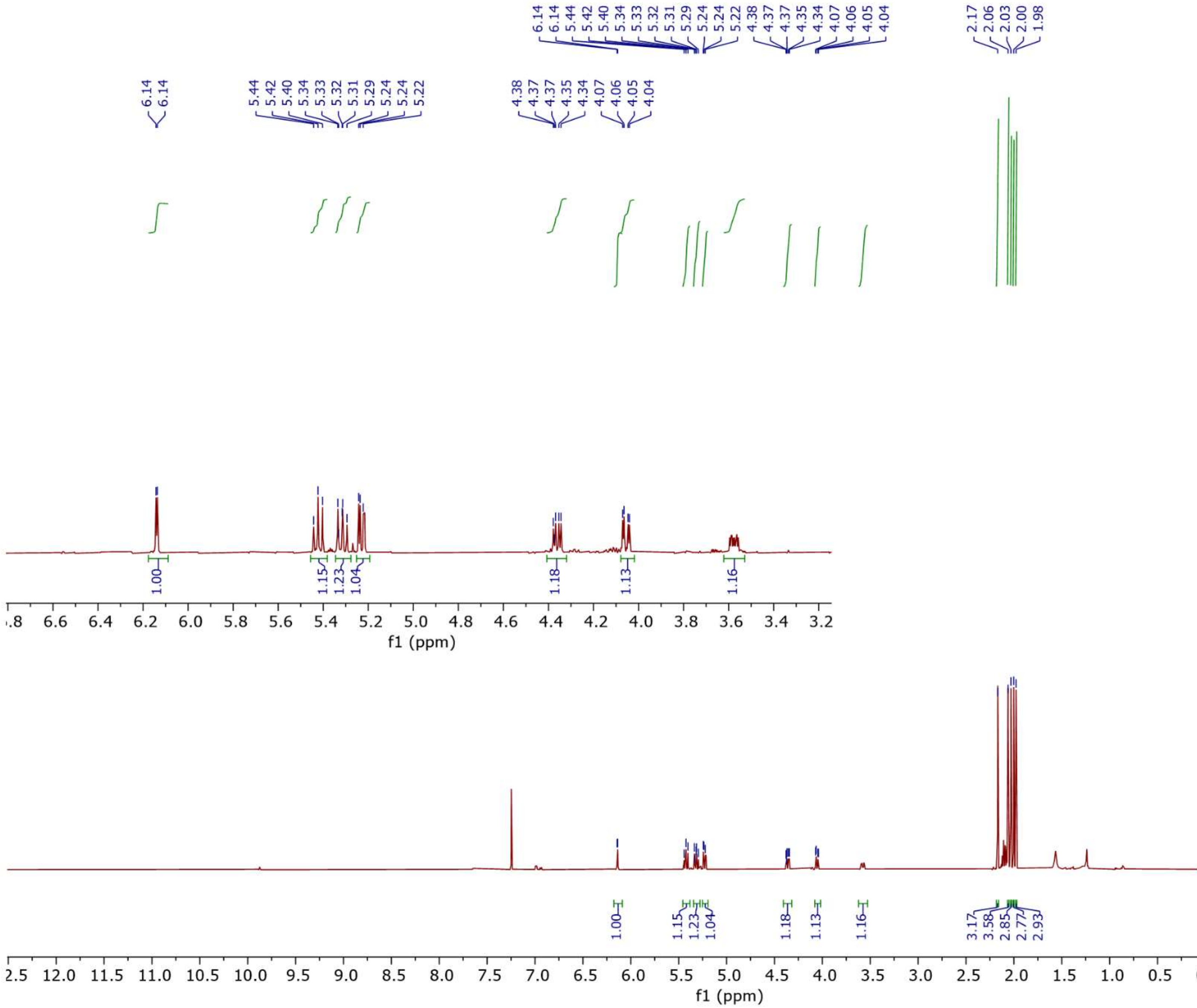

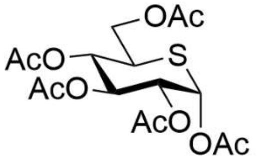

^{13}C NMR (125.67 MHz, $CDCl_3$) spectrum of decomposition product 9:

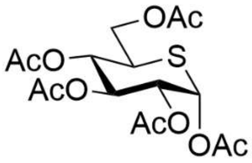

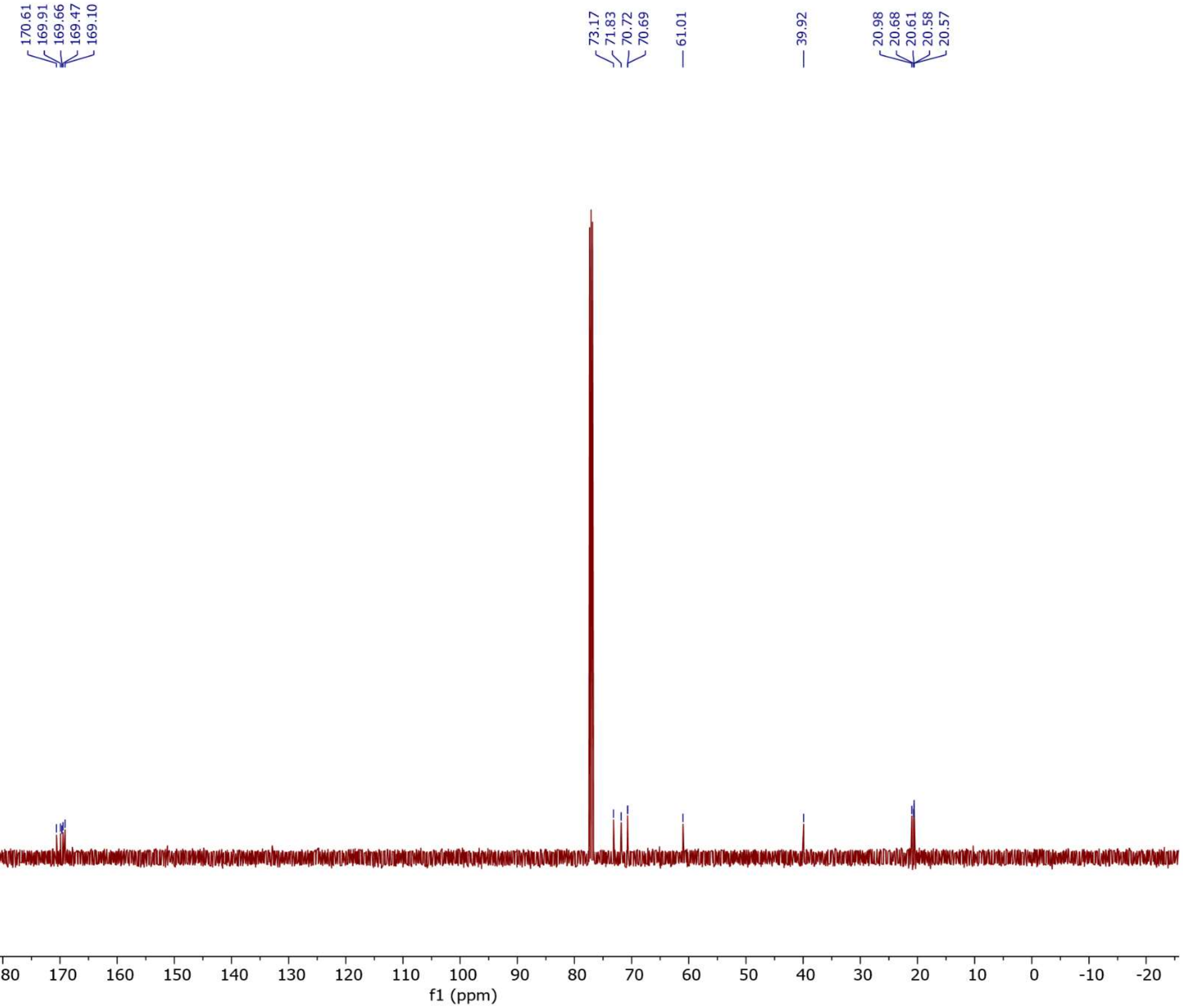

^{1}H NMR (500 MHz, $CDCl_3$) spectrum of mixture of decomposition products 11, 78:

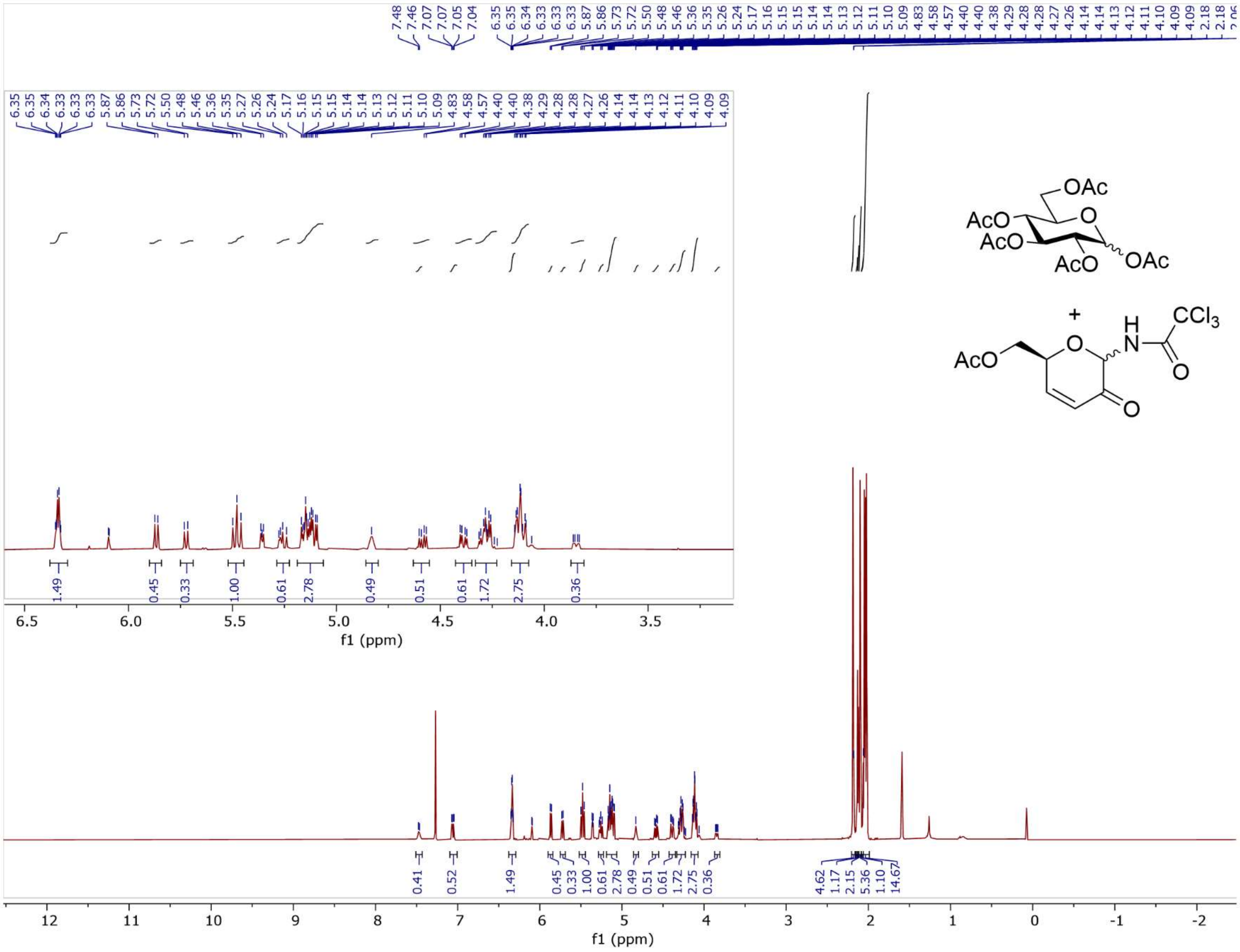

^{13}C NMR (125.67 MHz, $CDCl_3$) spectrum of mixture of decomposition products 11, 78:

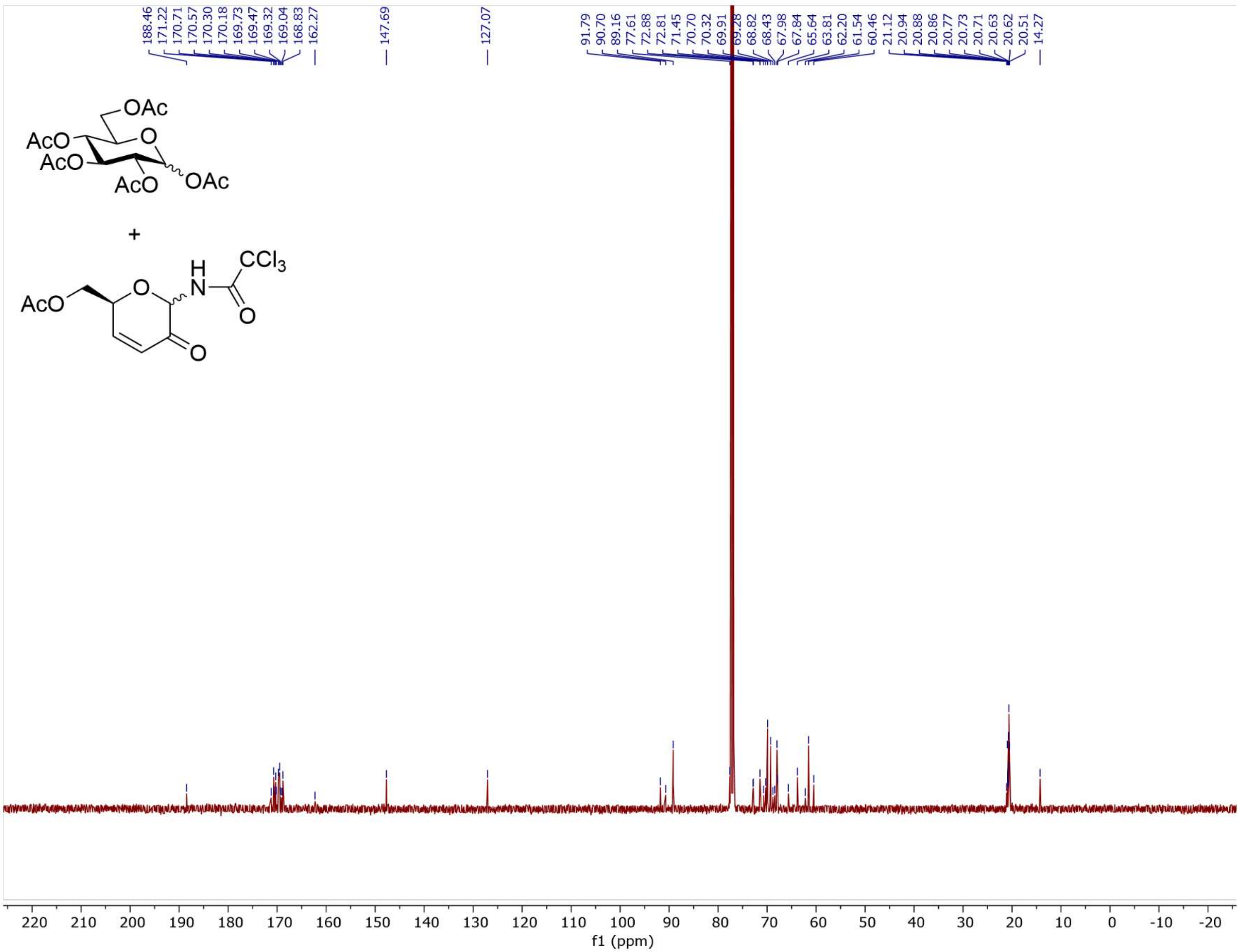

COSY ($CDCl_3$) spectrum of mixture of decomposition products 11, 78:

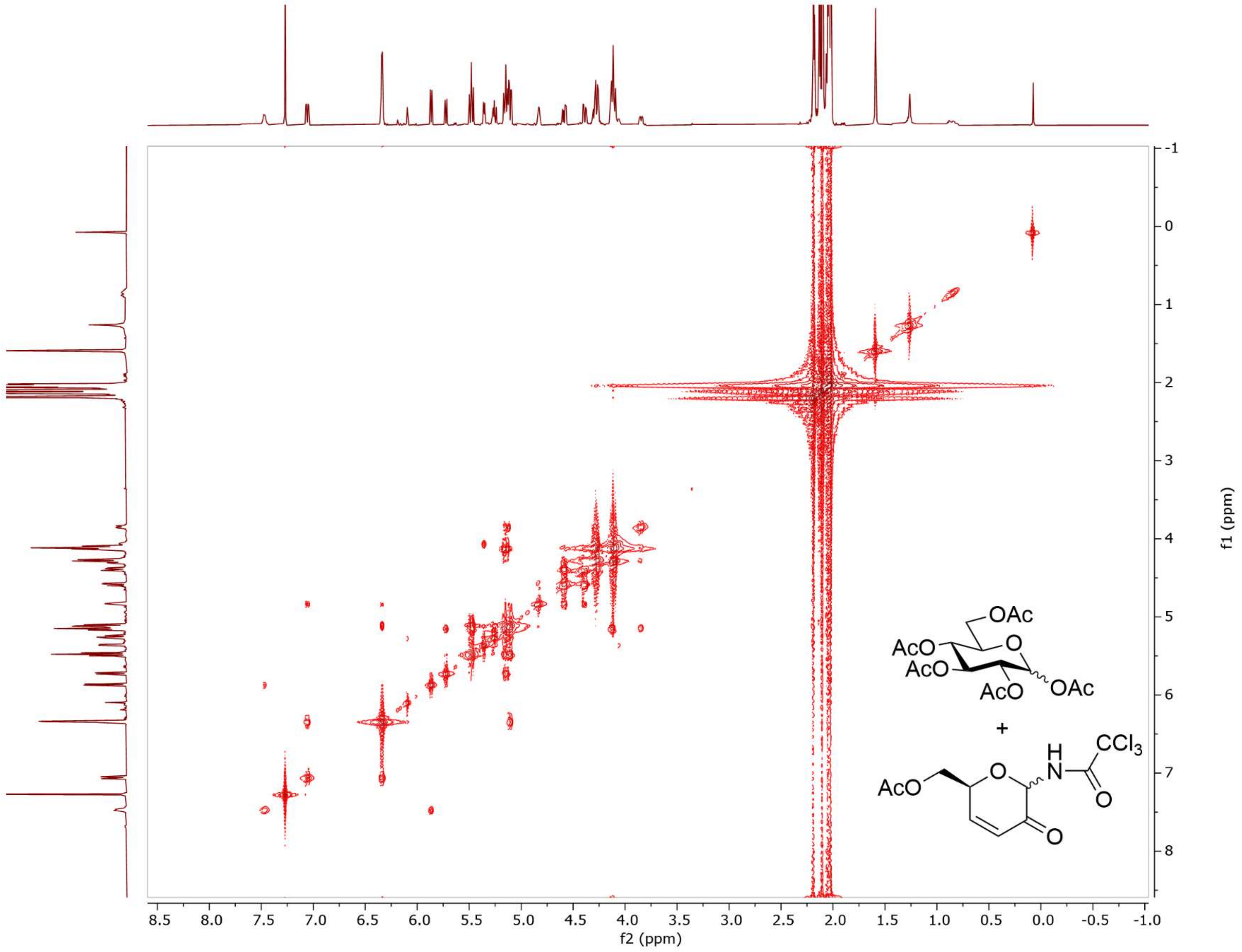

HSQC ($CDCl_3$) spectrum of mixture of decomposition products 11, 78:

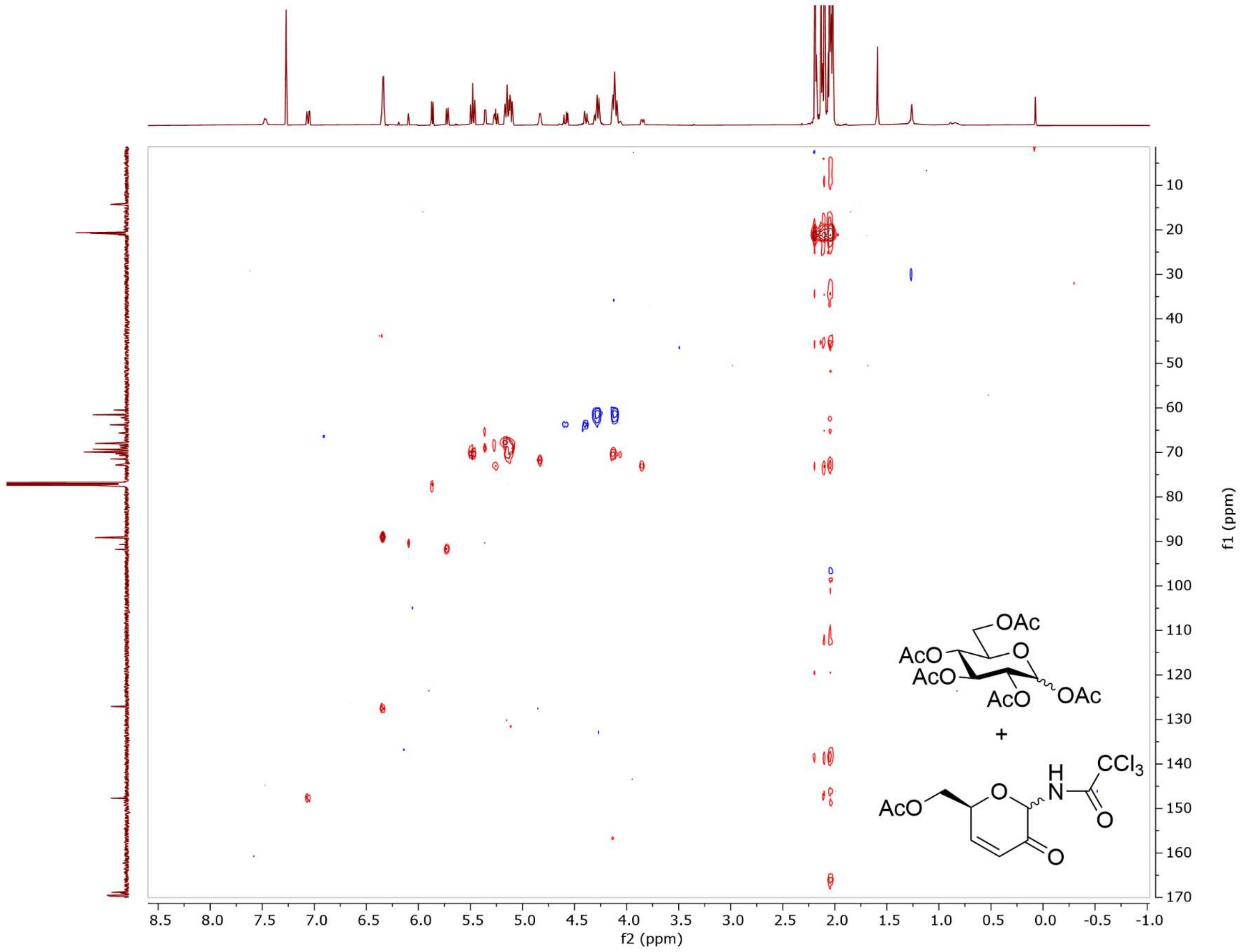

HMBC ($CDCl_3$) spectrum of mixture of decomposition products 11, 78:

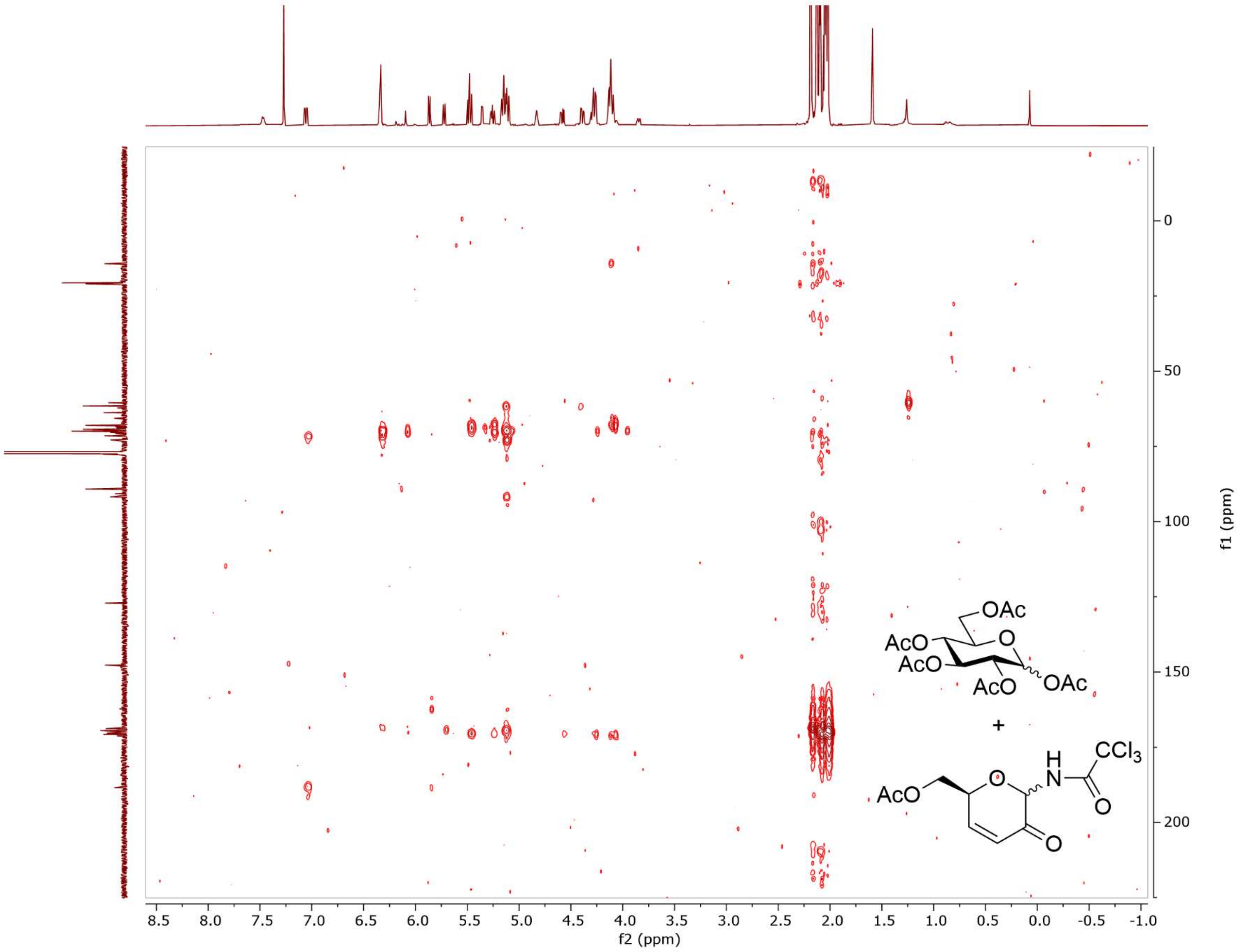

1H NMR (500 MHz, CDCl3) spectrum of the decomposition product 84:

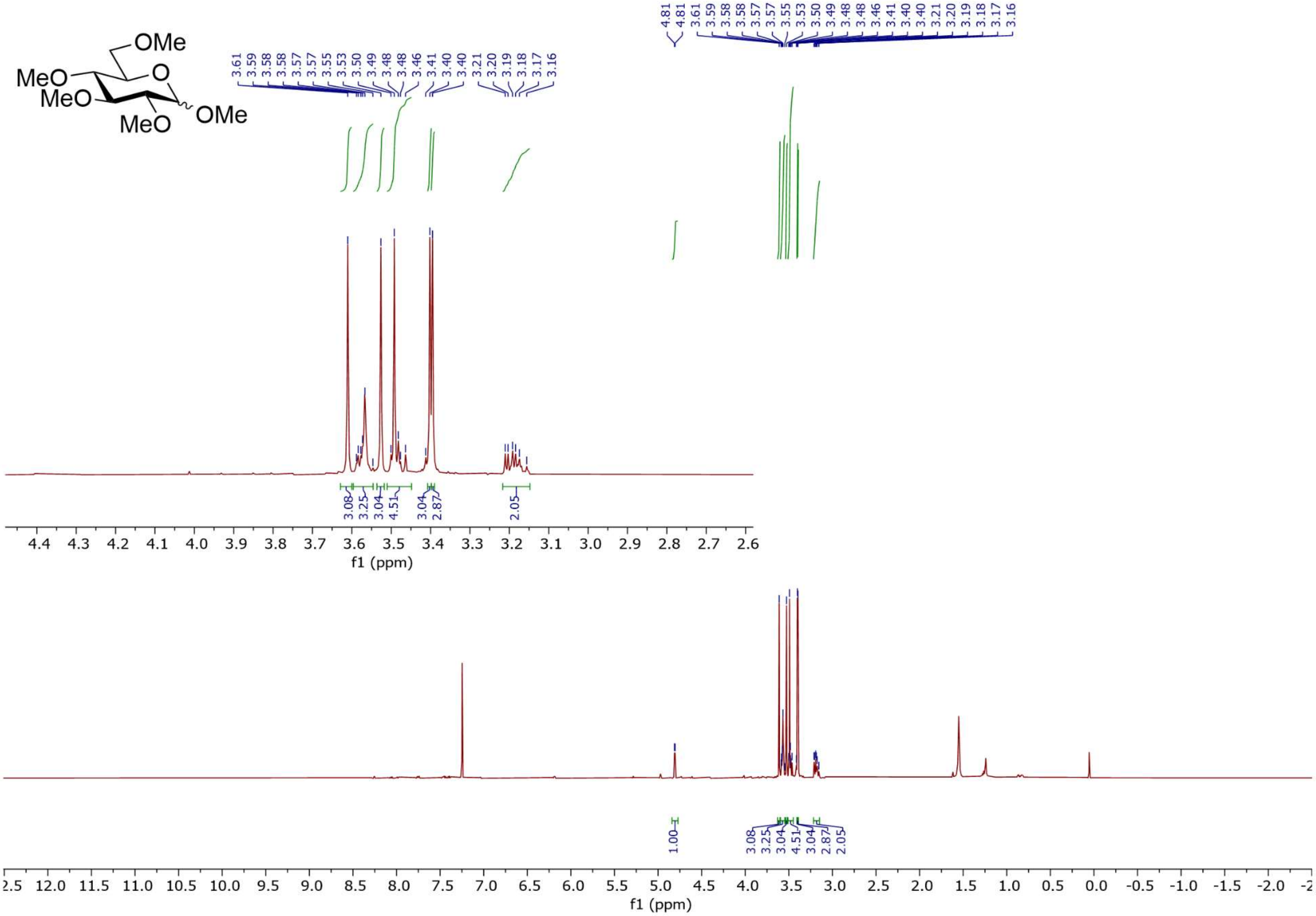

13C NMR (125.67 MHz, CDCl3) spectrum of mixture of the decomposition product 84:

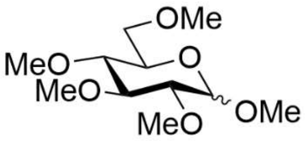

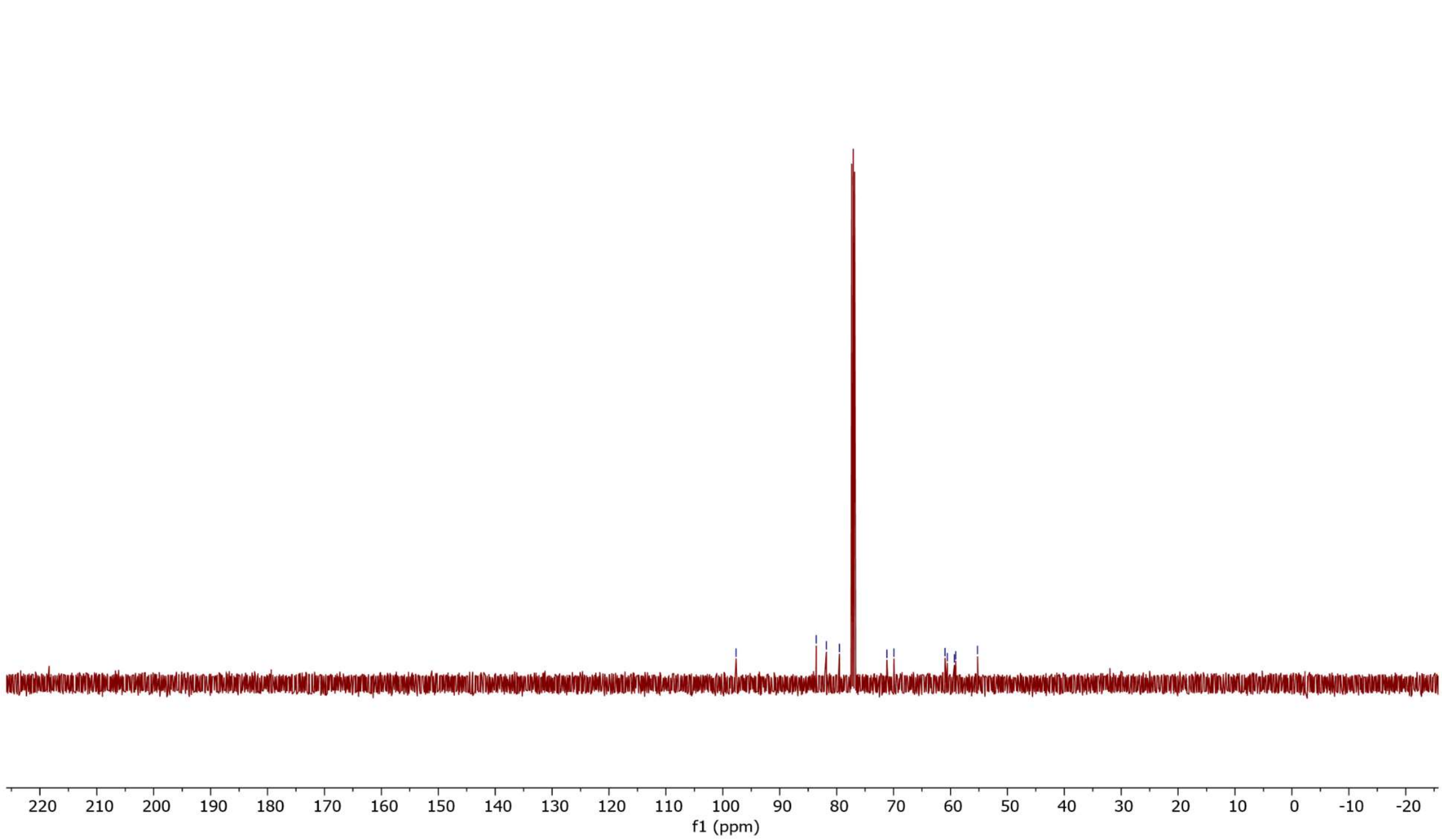

^{1}H NMR (500 MHz, CDCl$_3$) spectrum of decomposition product 90:

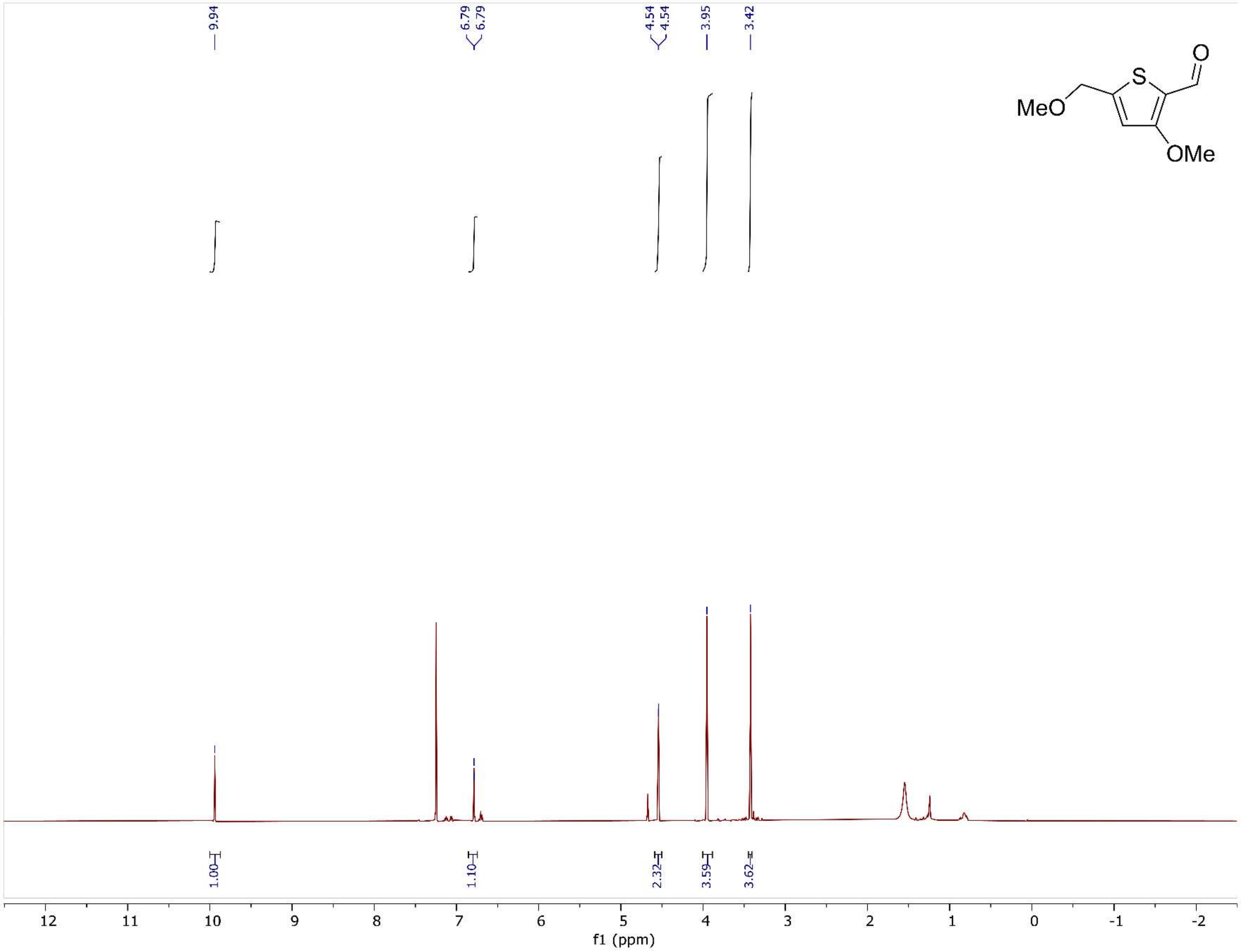

^{13}C NMR (125.67 MHz, $CDCl_3$) spectrum of decomposition product 90:

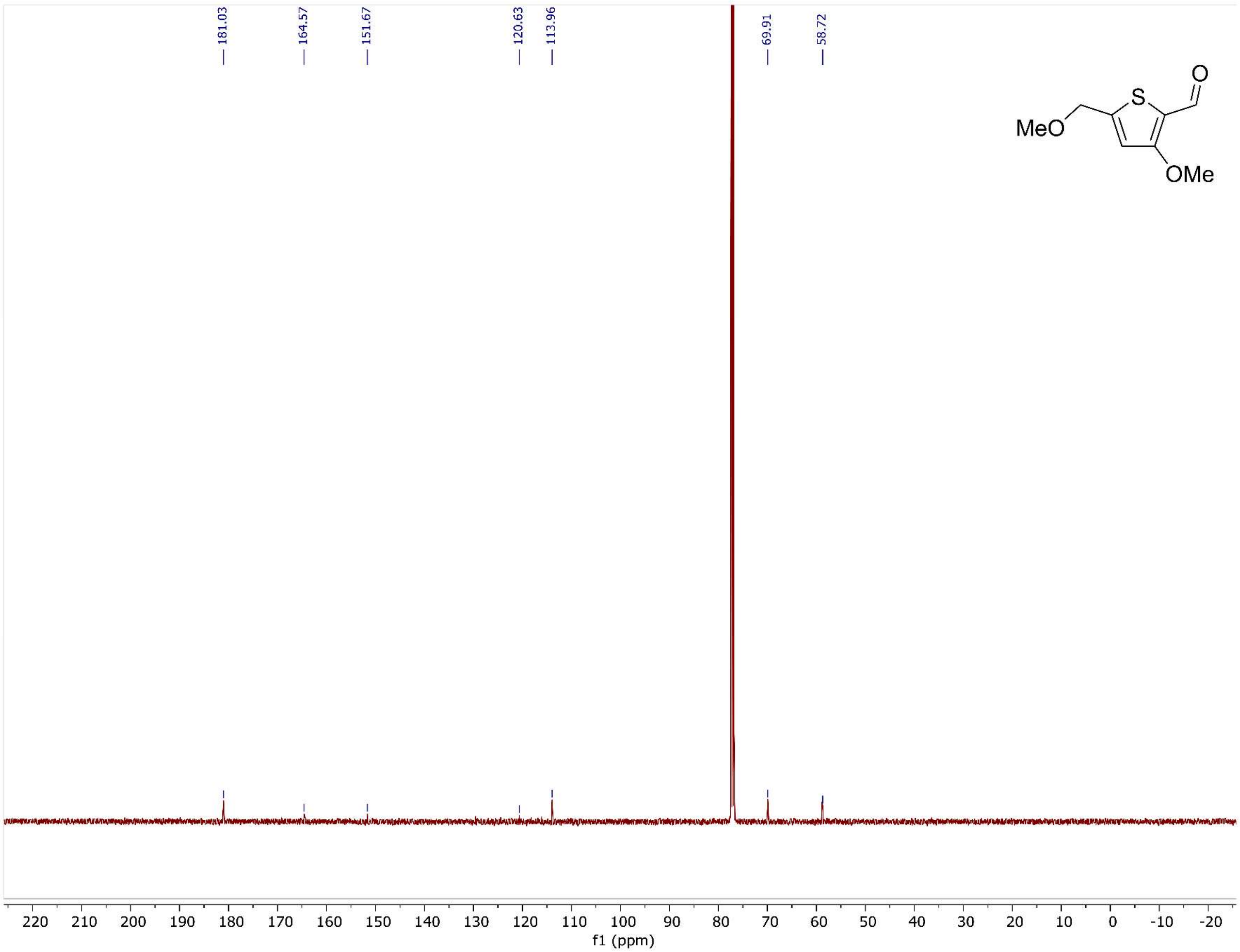

HSQC ($CDCl_3$) spectrum of decomposition product 90:

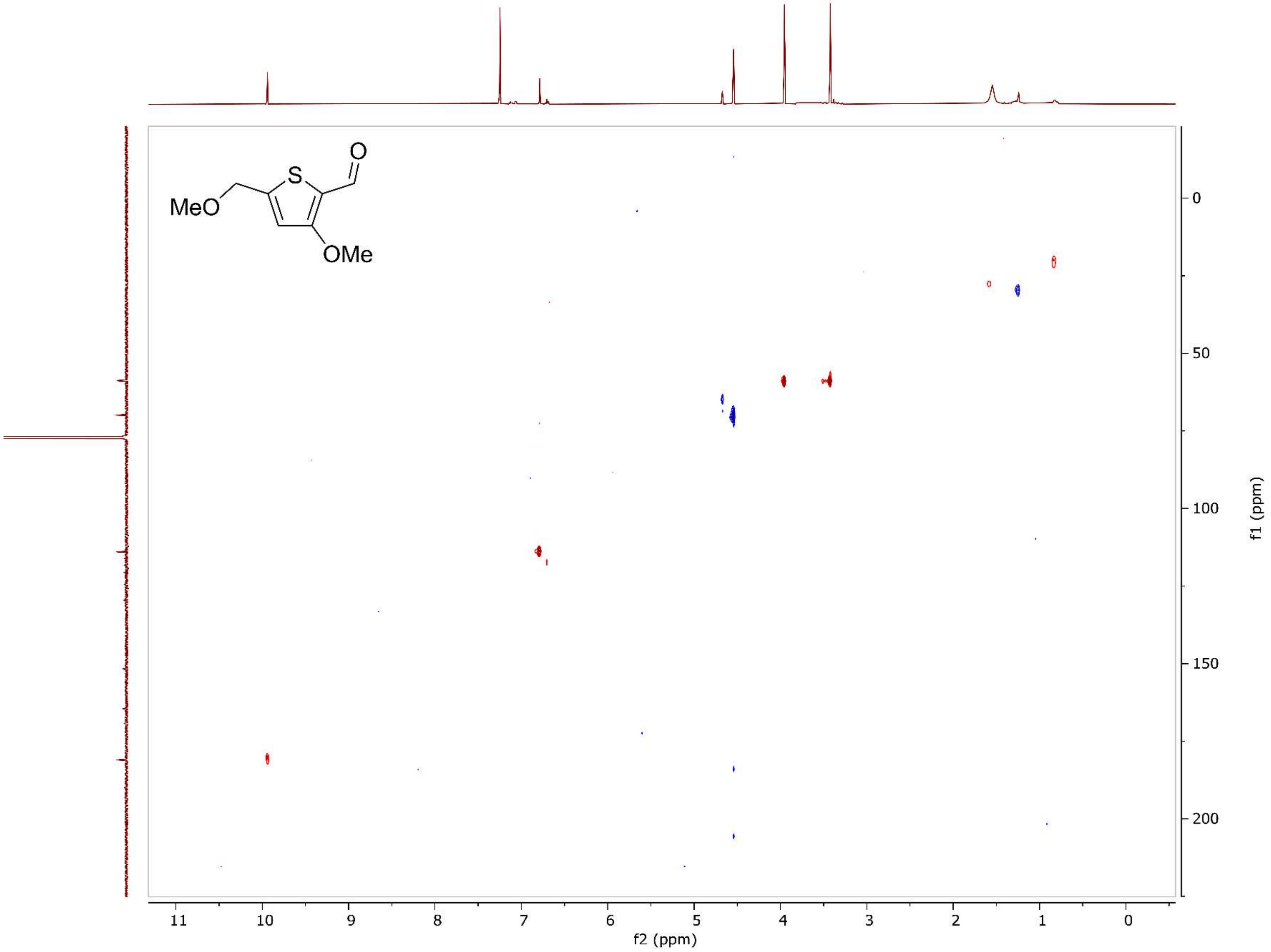

HMBC ($CDCl_3$) spectrum of decomposition product 90:

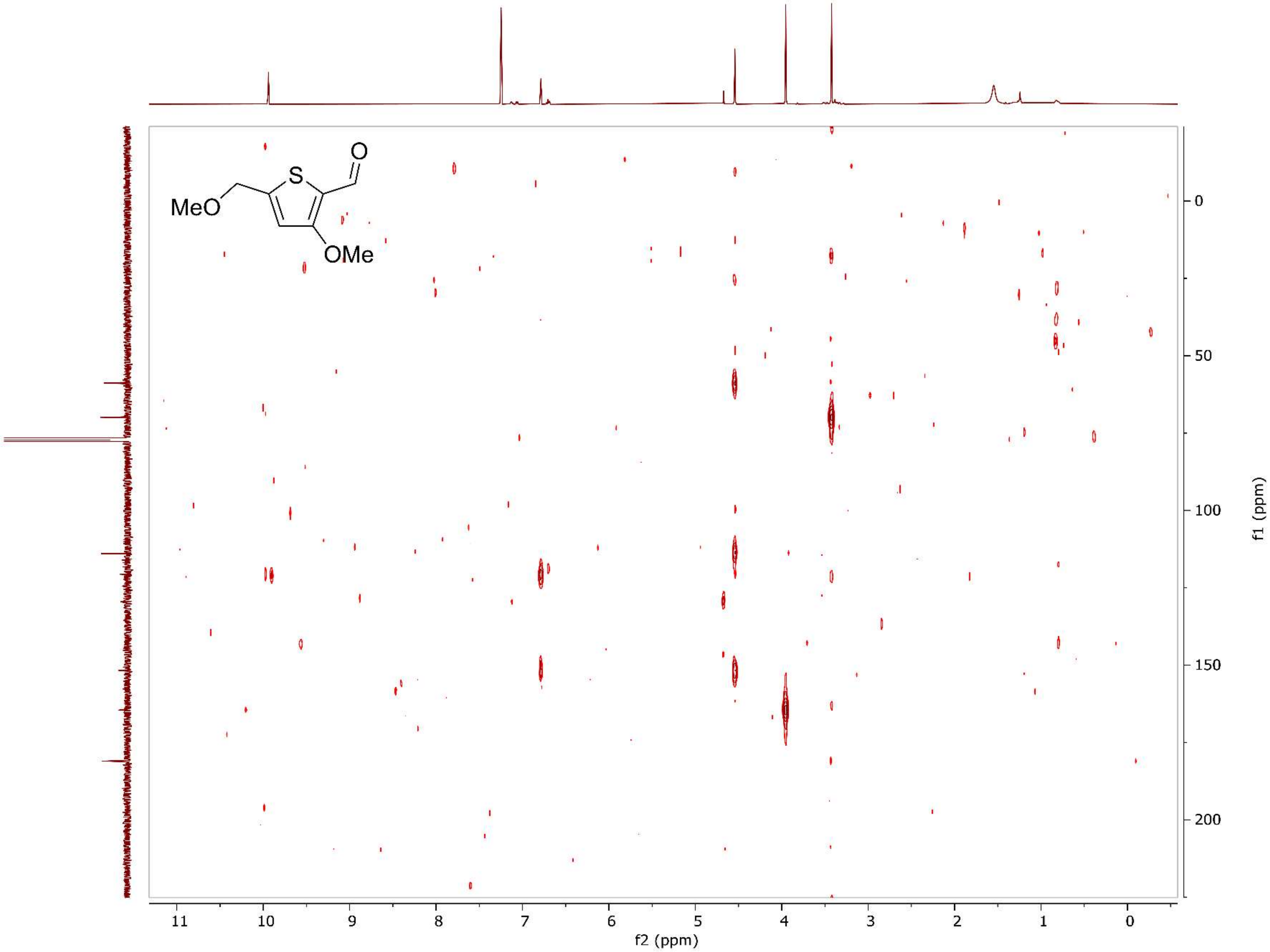

FTIR ($CHCl_3$) spectrum of decomposition product 90:

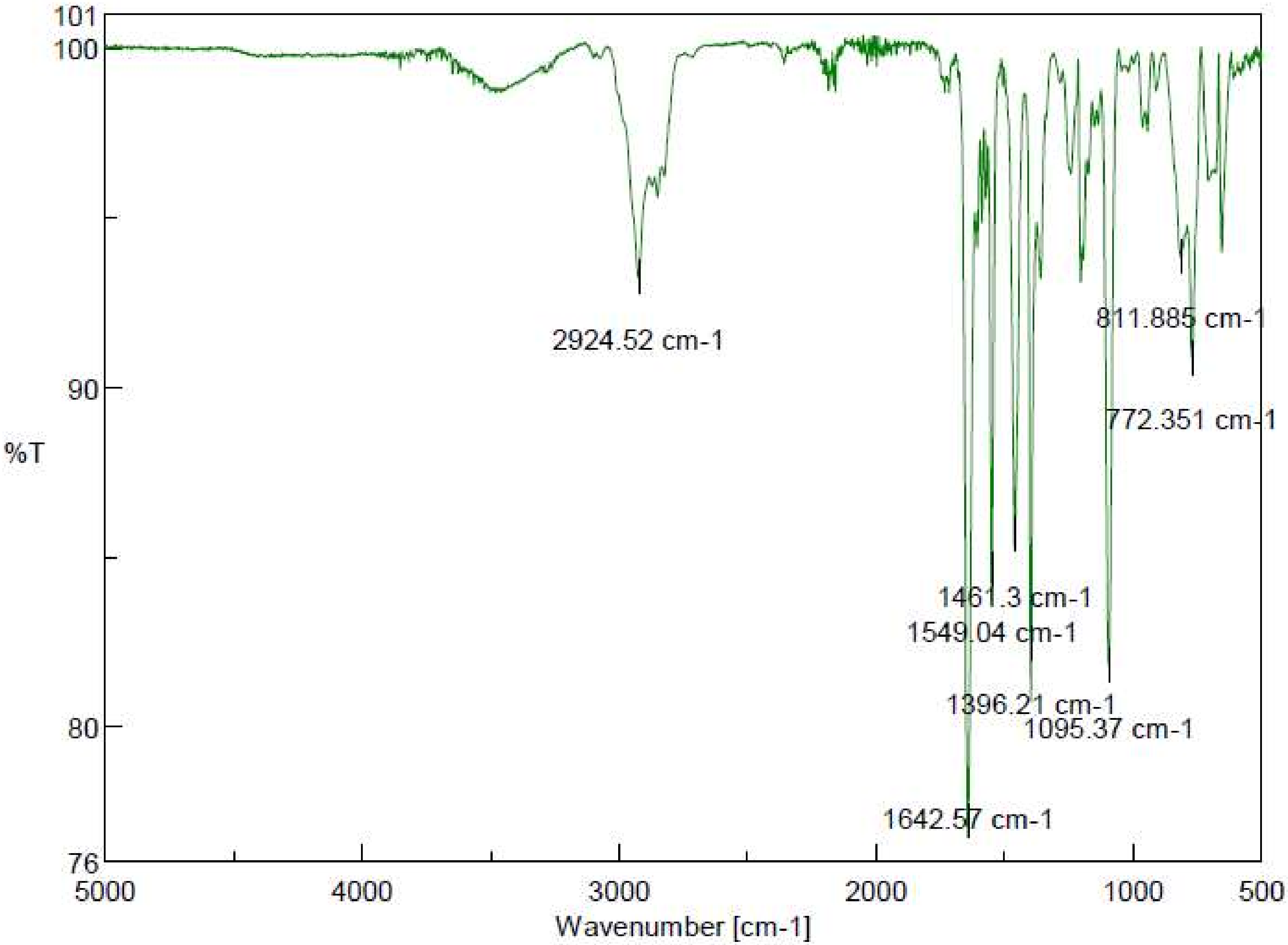

^{1}H NMR (500 MHz, $CDCl_3$) spectrum of decomposition product 92:

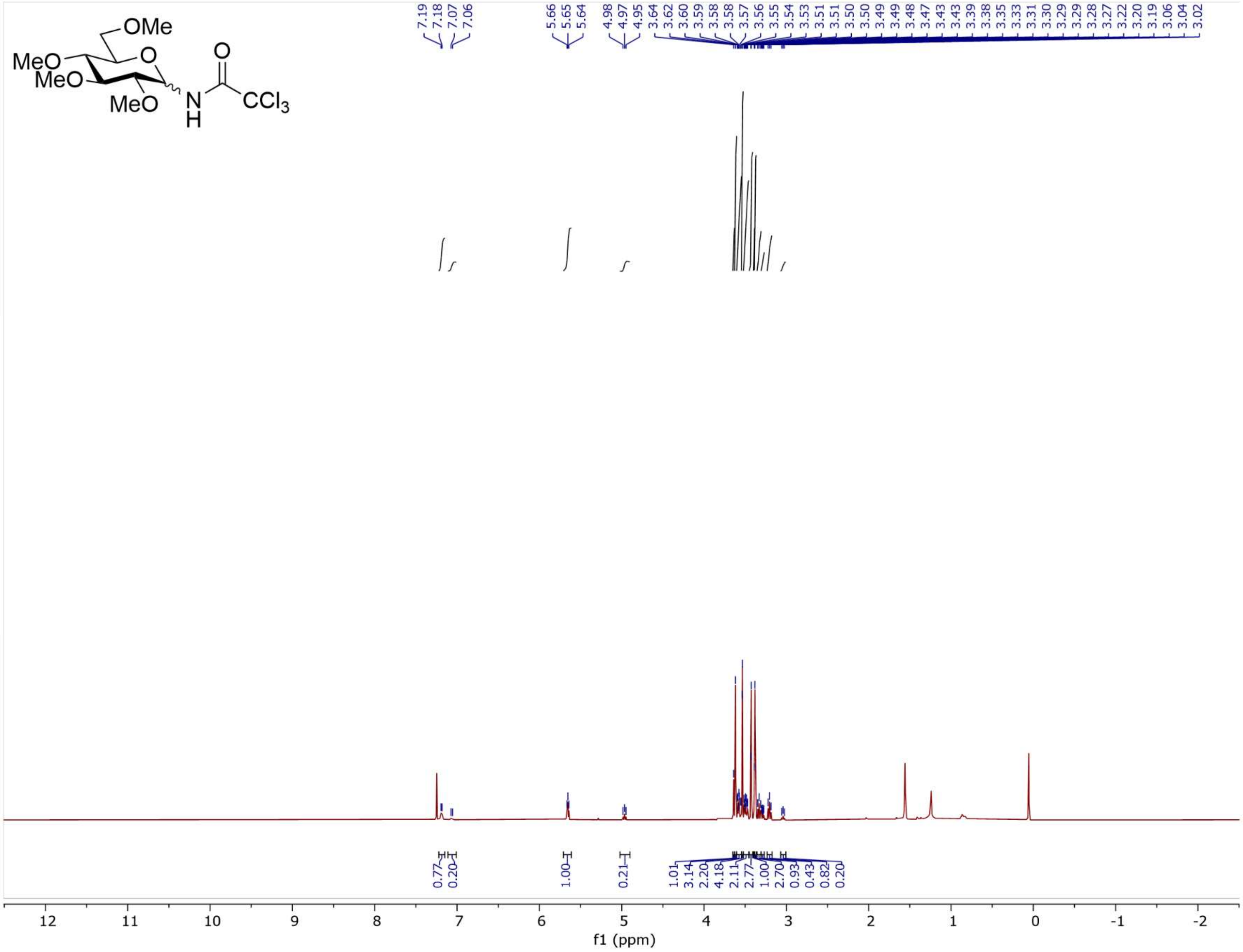

^{13}C NMR (125.67 MHz, $CDCl_3$) spectrum of decomposition product 92:

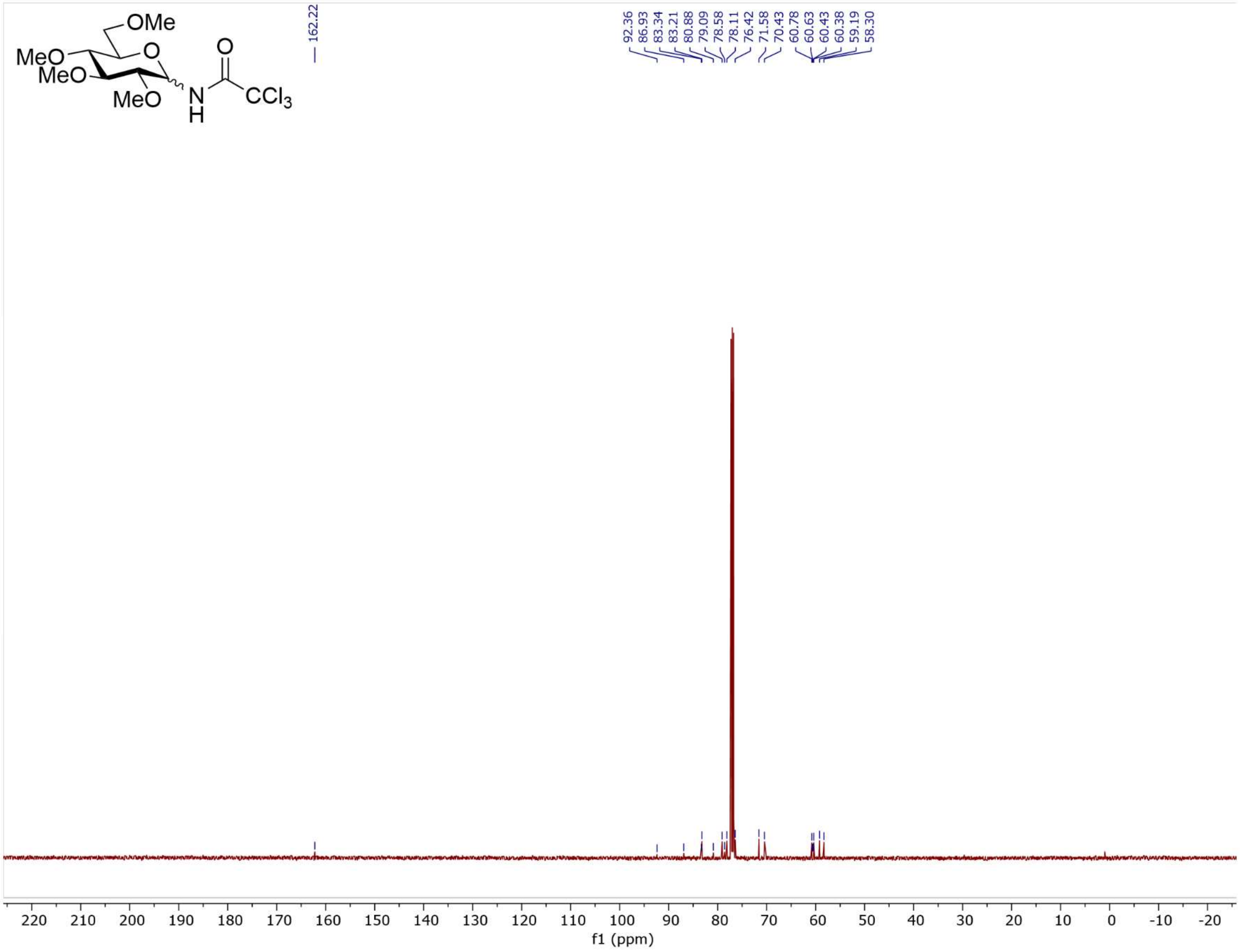

DEPT-90 ($CDCl_3$) spectrum of decomposition product 92:

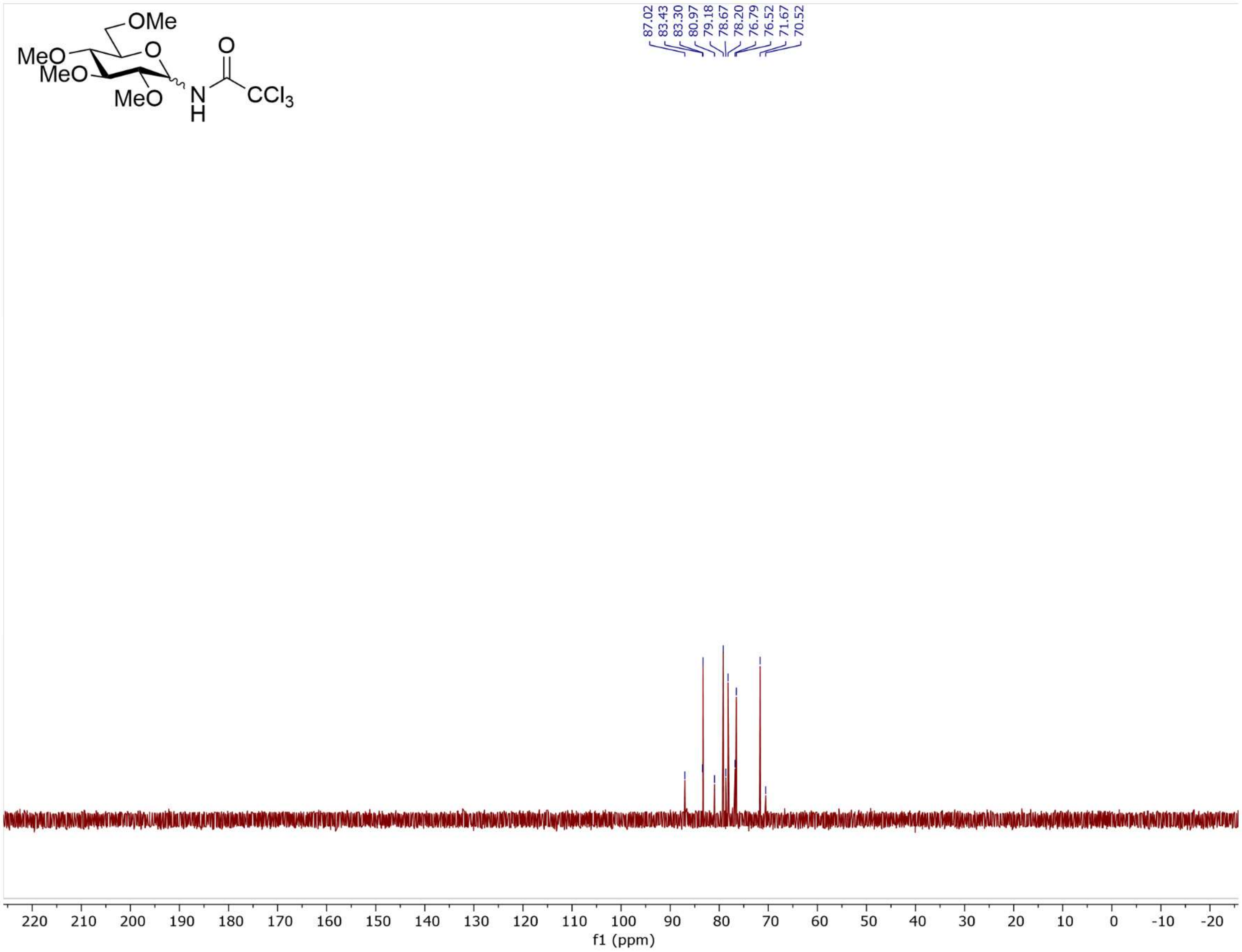

COSY ($CDCl_3$) spectrum of decomposition product 92:

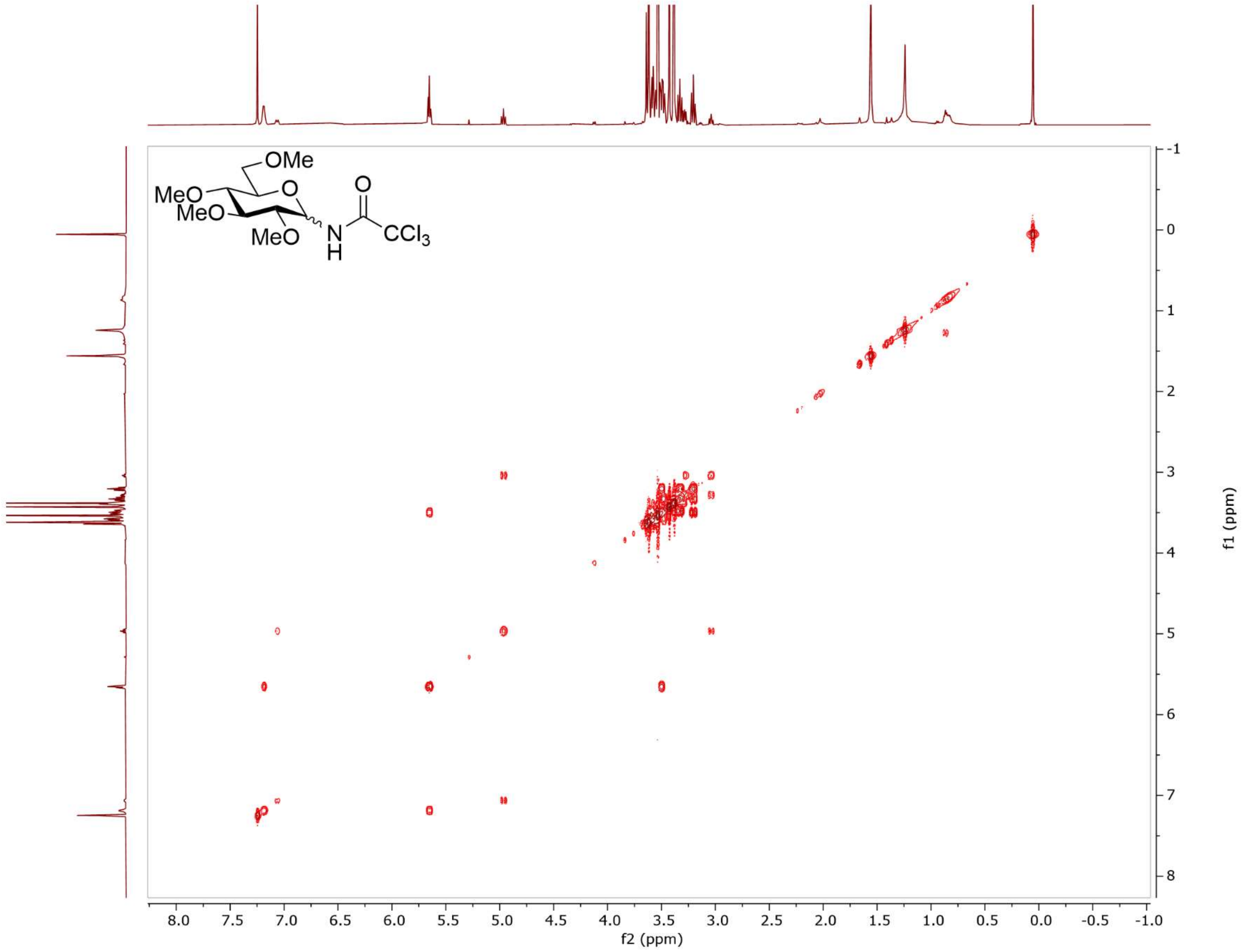

HSQC ($CDCl_3$) spectrum of decomposition product 92:

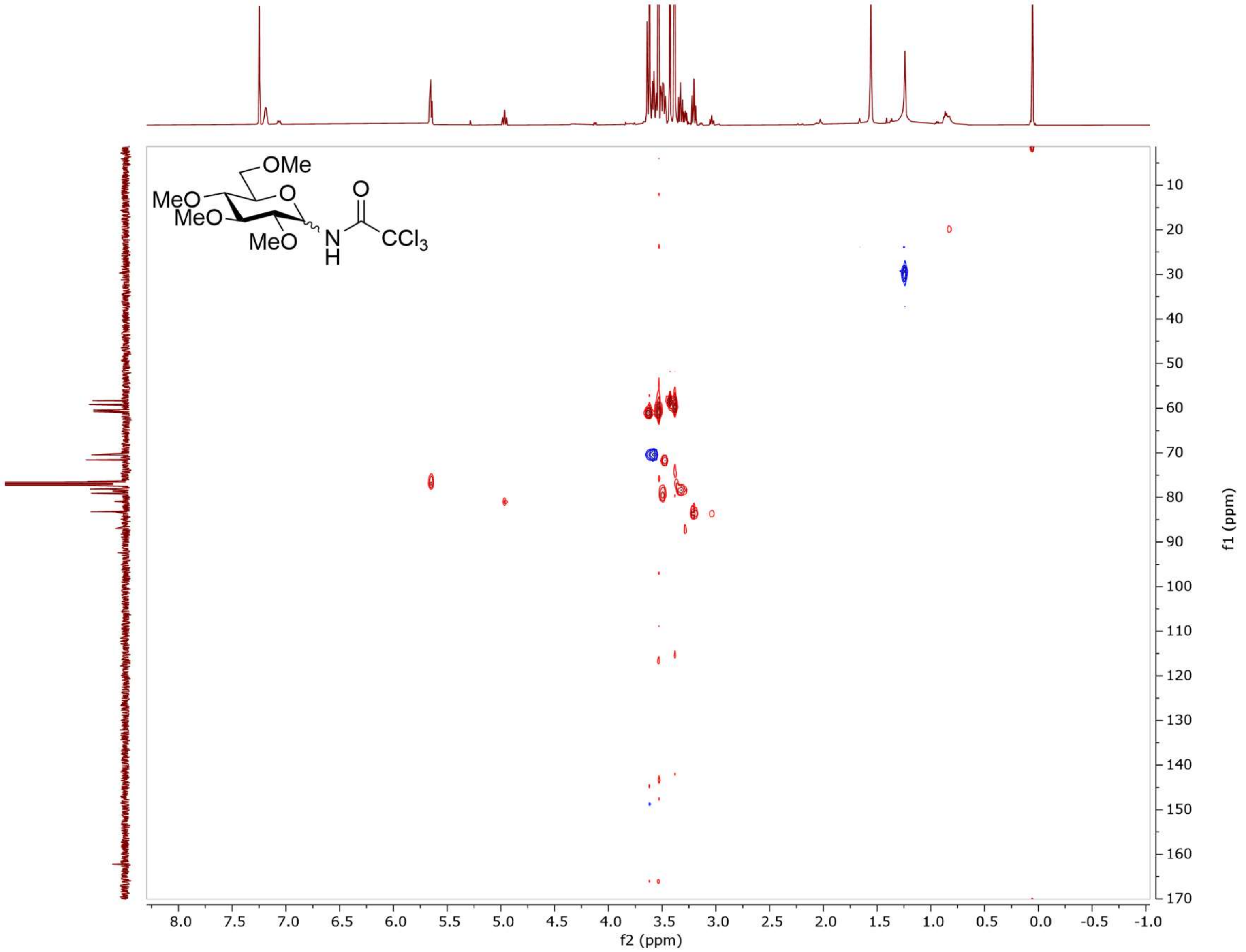

^{1}H NMR (500 MHz, $CDCl_3$) spectrum of decomposition product 94:

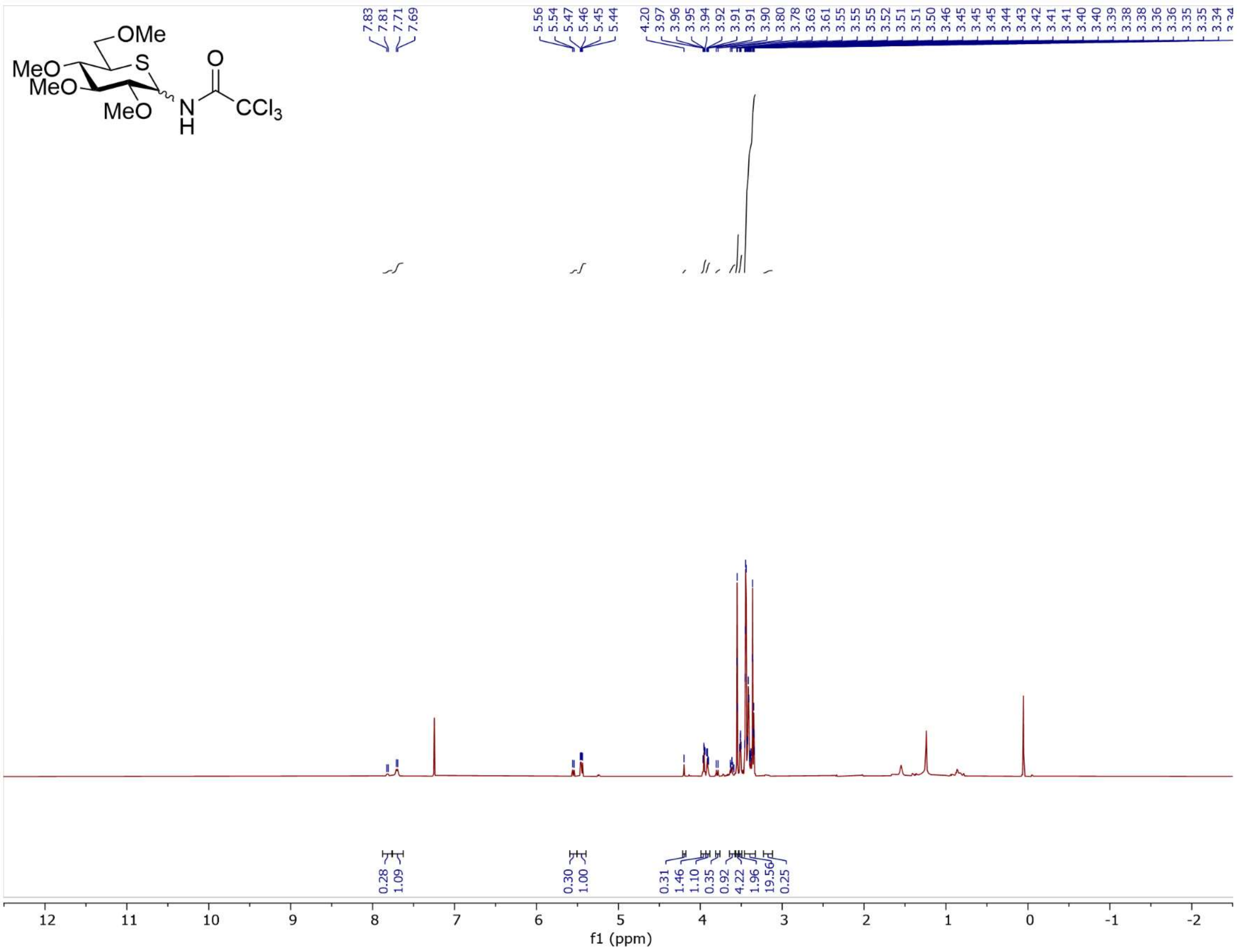

^{13}C NMR (125.67 MHz, CDCl$_3$) spectrum of decomposition product 94:

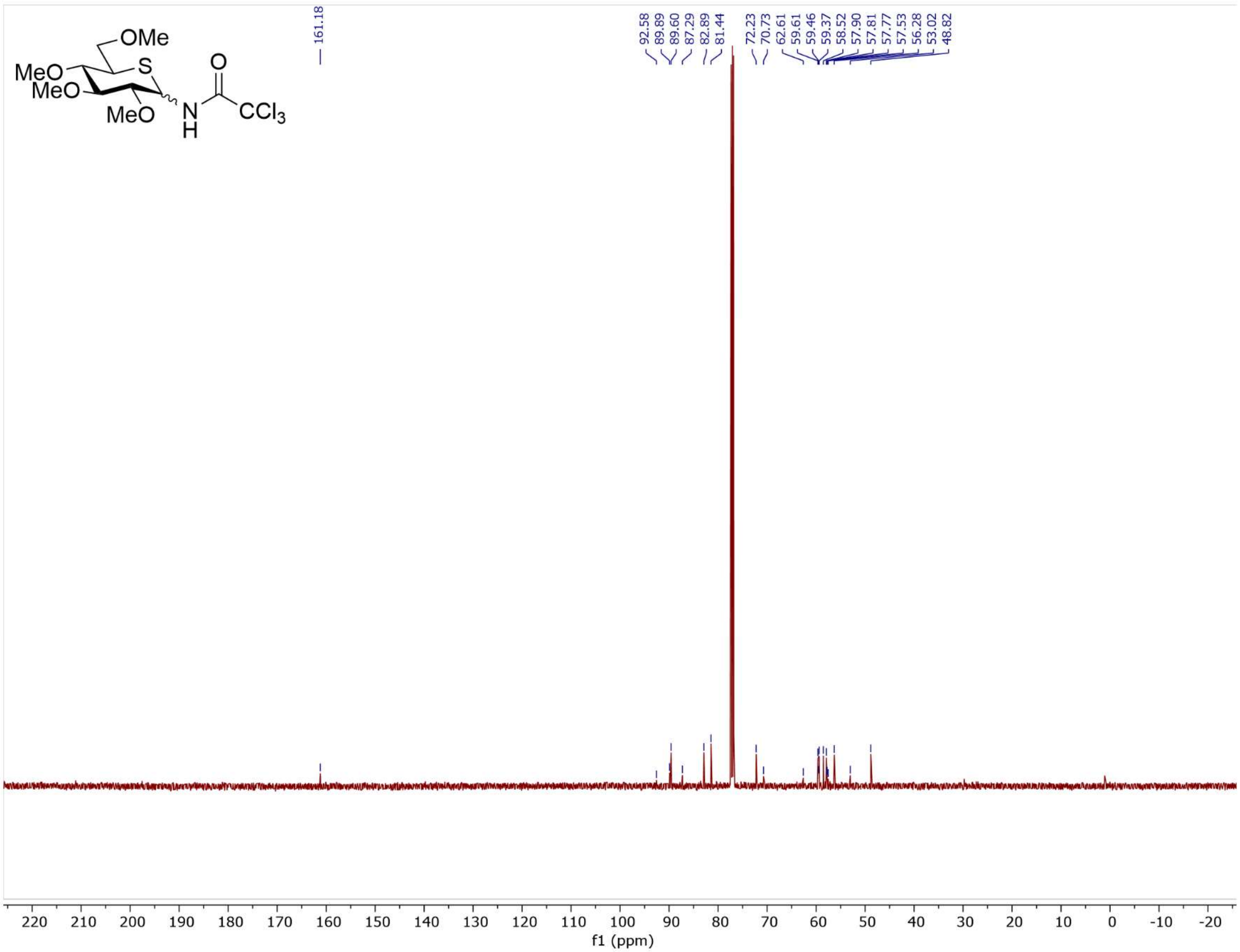

COSY ($CDCl_3$) spectrum of decomposition product 94:

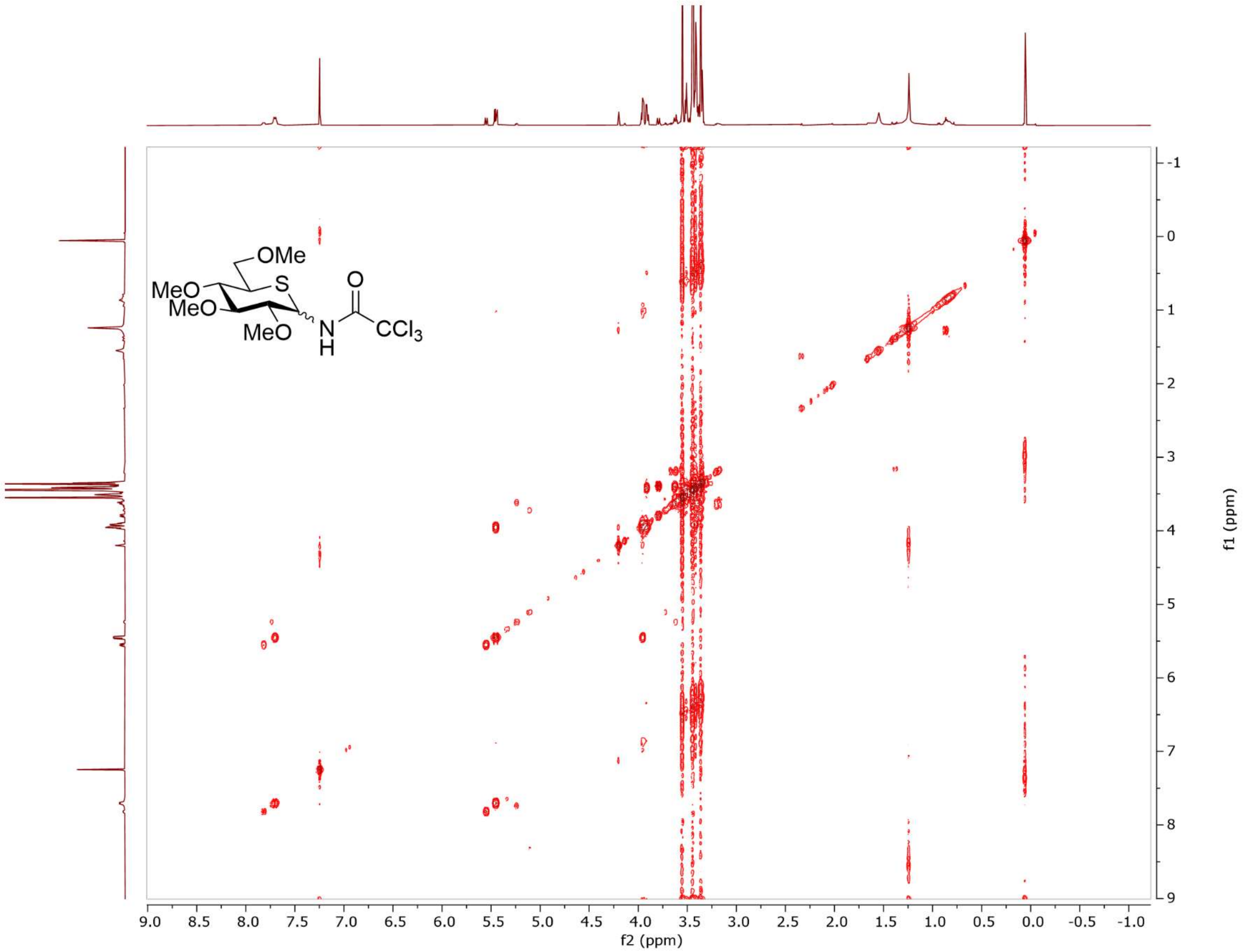

HSQC ($CDCl_3$) spectrum of decomposition product 94:

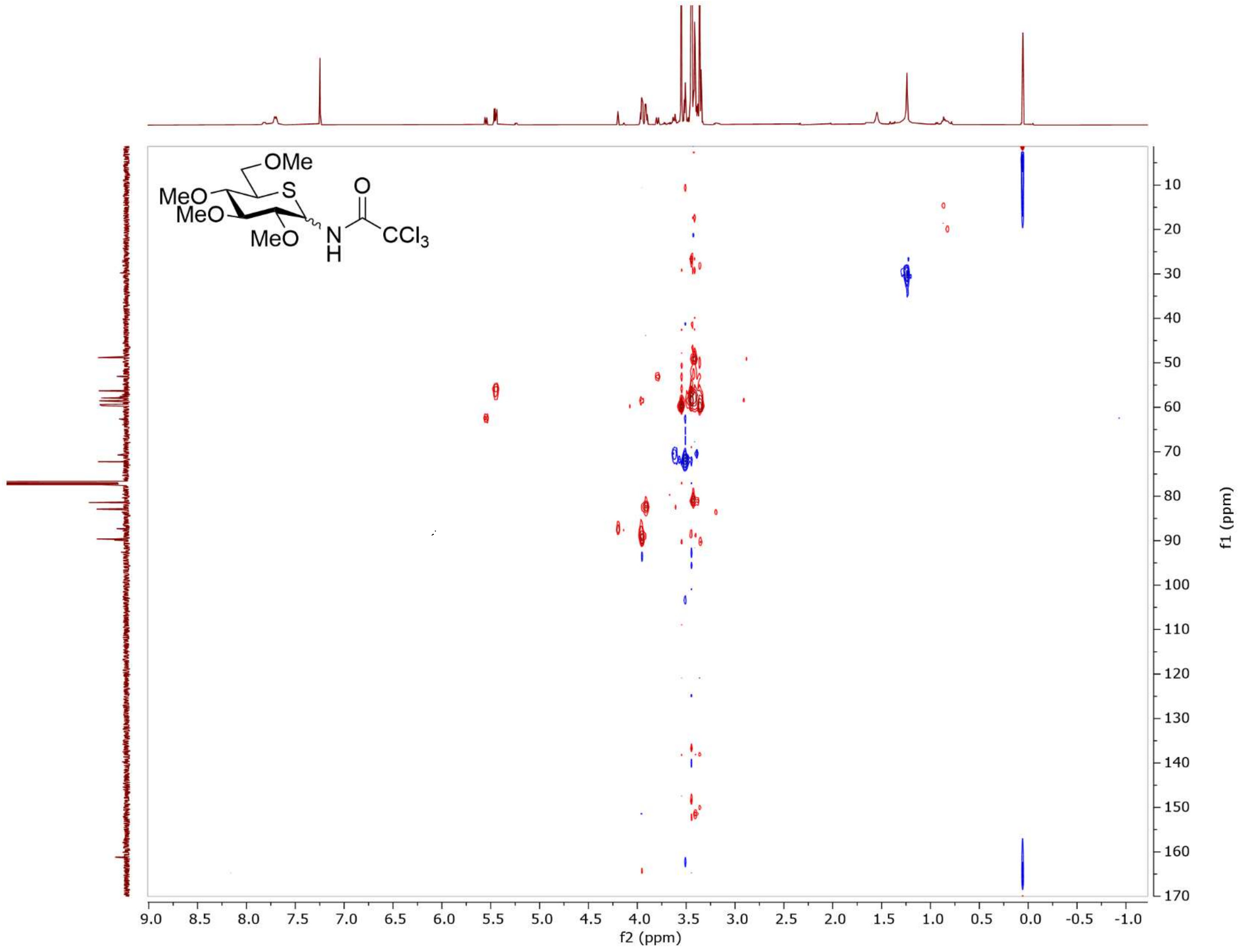

References

(1) Gu, X.; Li, X.; Chai, Y.; Yang, Q.; Li, P.; Yao, Y. A simple metal-free catalytic sulfoxidation under visible light and air. *Green Chem.* **2013**, *15* (2), 357-361. DOI: 10.1039/C2GC36683E.

(2) Dupuy, C.; Crozet, M.-P.; Surzur, J.-M. Heterocyclisation radicalaire de thiols acetyleniques. *Bull. Soc. Chim. Fr.* **1980**, *2* (7), 361-373.

(3) Svansson, L.; Johnston, B. D.; Gu, J.-H.; Patrick, B.; Pinto, B. M. Synthesis and Conformational Analysis of a Sulfonium-Ion Analogue of the Glycosidase Inhibitor Castanospermine. *J. Am. Chem. Soc.* **2000**, *122* (44), 10769-10775. DOI: 10.1021/ja002038h.

(4) Sanhueza, C. A.; Dorta, R. L.; Vázquez, J. T. Stereochemical Properties of Glucosyl Sulfoxides in Solution. *J. Org. Chem.* **2011**, *76* (19), 7769-7780. DOI: 10.1021/jo201130x.

(5) Sanhueza, C. A.; Arias, A. C.; Dorta, R. L.; Vázquez, J. T. Absolute configuration of glycosyl sulfoxides. *Tetrahedron: Asymmetry* **2010**, *21* (15), 1830-1832. DOI: 10.1016/j.tetasy.2010.06.019.

(6) Deore, B.; Ocando, J. E.; Pham, L. D.; Sanhueza, C. A. Anodic Reactivity of Alkyl S-Glucosides. *J. Org. Chem.* **2022**, *87* (9), 5952-5960. DOI: 10.1021/acs.joc.2c00222.

(7) Bowden, T.; Garegg, P. J.; Maloisel, J.-L.; Konradsson, P. A mechanistic study: Nucleophile dependence in glucosylations with glucosyl bromides. *Isr. J. Chem.* **2000**, *40* (3-4), 271-277. DOI: 10.1560/P2J6-2MN2-0WHQ-R3BF.

(8) Neumaier, J. M.; Madani, A.; Klein, T.; Ziegler, T. Low-budget 3D-printed equipment for continuous flow reactions. *Beilstein J. Org. Chem.* **2019**, *15*, 558-566. DOI: 10.3762/bjoc.15.50.

(9) Xu, G.; Moeller, K. D. Anodic Coupling Reactions and the Synthesis of C-Glycosides. *Org. Lett.* **2010**, *12* (11), 2590-2593. DOI: 10.1021/ol100800u.

(10) Addanki, R. B.; Moktan, S.; Halder, S.; Sharma, M.; Sarmah, B. K.; Bhattacharyya, K.; Kancharla, P. K. Exploiting the Strained Ion-Pair Interactions of Sterically Hindered Pyridinium Salts Toward SN2 Glycosylation of Glycosyl Trichloroacetimidates. *J. Org. Chem.* **2024**, *89* (6), 3713-3725. DOI: 10.1021/acs.joc.3c02207.

(11) Morii, Y.; Matsuda, H.; Ohara, K.; Hashimoto, M.; Miyairi, K.; Okuno, T. Synthetic studies on oligosaccharides composed of 5-thioglucopyranose units. *Biorg. Med. Chem.* **2005**, *13* (17), 5113-5144. DOI: 10.1016/j.bmc.2005.05.028.

(12) Becke, A. D. Density-functional exchange-energy approximation with correct asymptotic behavior. *Phys. Rev. A* **1988**, *38* (6), 3098-3100. DOI: 10.1103/PhysRevA.38.3098.

(13) Clark, T.; Chandrasekhar, J.; Spitznagel, G. W.; Schleyer, P. V. R. Efficient diffuse function-augmented basis sets for anion calculations. III. The 3-21+G basis set for first-row elements, Li–F. *J. Comput. Chem.* **1983**, *4* (3), 294-301. DOI: 10.1002/jcc.540040303.

(14) Francl, M. M.; Pietro, W. J.; Hehre, W. J.; Binkley, J. S.; Gordon, M. S.; DeFrees, D. J.; Pople, J. A. Self-consistent molecular orbital methods. XXIII. A polarization-type basis set for second-row elements. *J. Chem. Phys.* **1982**, *77* (7), 3654-3665. DOI: 10.1063/1.444267 (acccessed 12/16/2024).

(15) Krishnan, R.; Binkley, J. S.; Seeger, R.; Pople, J. A. Self-consistent molecular orbital methods. XX. A basis set for correlated wave functions. *J. Chem. Phys.* **1980**, *72* (1), 650-654. DOI: 10.1063/1.438955.

(16) McLean, A. D.; Chandler, G. S. Contracted Gaussian basis sets for molecular calculations. I. Second row atoms, Z=11–18. *J. Chem. Phys.* **1980**, *72* (10), 5639-5648. DOI: 10.1063/1.438980.

(17) Spitznagel, G. W.; Clark, T.; von Ragué Schleyer, P.; Hehre, W. J. An evaluation of the performance of diffuse function-augmented basis sets for second row elements, Na-Cl. *J. Comput. Chem.* **1987**, *8* (8), 1109-1116. DOI: 10.1002/jcc.540080807.

(18) Marenich, A. V.; Cramer, C. J.; Truhlar, D. G. Universal Solvation Model Based on Solute Electron Density and on a Continuum Model of the Solvent Defined by the Bulk Dielectric Constant and Atomic Surface Tensions. *The Journal of Physical Chemistry B* **2009**, *113* (18), 6378-6396. DOI: 10.1021/jp810292n.

(19) Shao, Y.; Gan, Z.; Epifanovsky, E.; Gilbert, A. T. B.; Wormit, M.; Kussmann, J.; Lange, A. W.; Behn, A.; Deng, J.; Feng, X.; et al. Advances in molecular quantum chemistry contained in the Q-Chem 4 program package. *Mol. Phys.* **2015**, *113* (2), 184-215. DOI: 10.1080/00268976.2014.952696.

(20) Safi, Z. S.; Wazzan, N. DFT calculations of 1H- and 13C-NMR chemical shifts of 3-methyl-1-phenyl-4-(phenyldiazenyl)-1H-pyrazol-5-amine in solution. *Scientific Reports* **2022**, *12* (1), 17798. DOI: 10.1038/s41598-022-22900-y.

(21) Iron, M. A. Evaluation of the Factors Impacting the Accuracy of 13C NMR Chemical Shift Predictions using Density Functional Theory—The Advantage of Long-Range Corrected Functionals. *J. Chem. Theory Comput.* **2017**, *13* (11), 5798-5819. DOI: 10.1021/acs.jctc.7b00772.

(22) Neese, F. Software update: the ORCA program system, version 4.0. *WIREs Computational Molecular Science* **2018**, *8* (1), e1327. DOI: 10.1002/wcms.1327.

(23) Neese, F. The ORCA program system. *WIREs Computational Molecular Science* **2012**, *2* (1), 73-78. DOI: 10.1002/wcms.81.

(24) Zhao, Y.; Truhlar, D. G. The M06 suite of density functionals for main group thermochemistry, thermochemical kinetics, noncovalent interactions, excited states, and transition elements: two new functionals and systematic testing of four M06-class functionals and 12 other functionals. *Theor. Chem. Acc.* **2008**, *120* (1), 215-241. DOI: 10.1007/s00214-007-0310-x.

(25) Woon, D. E.; Dunning, T. H., Jr. Gaussian basis sets for use in correlated molecular calculations. V. Core-valence basis sets for boron through neon. *J. Chem. Phys.* **1995**, *103* (11), 4572-4585. DOI: 10.1063/1.470645.

(26) Kendall, R. A.; Dunning, T. H., Jr.; Harrison, R. J. Electron affinities of the first-row atoms revisited. Systematic basis sets and wave functions. *J. Chem. Phys.* **1992**, *96* (9), 6796-6806. DOI: 10.1063/1.462569.

(27) Dunning, T. H., Jr. Gaussian basis sets for use in correlated molecular calculations. I. The atoms boron through neon and hydrogen. *J. Chem. Phys.* **1989**, *90* (2), 1007-1023. DOI: 10.1063/1.456153.

(28) Grimme, S. Supramolecular Binding Thermodynamics by Dispersion-Corrected Density Functional Theory. *Chem. Eur. J.* **2012**, *18* (32), 9955-9964. DOI: 10.1002/chem.201200497.

(29) Ditchfield, R.; Hehre, W. J.; Pople, J. A. Self-Consistent Molecular-Orbital Methods. IX. An Extended Gaussian-Type Basis for Molecular-Orbital Studies of Organic Molecules. *J. Chem. Phys.* **1971**, *54* (2), 724-728. DOI: 10.1063/1.1674902.

(30) Gordon, M. S.; Binkley, J. S.; Pople, J. A.; Pietro, W. J.; Hehre, W. J. Self-consistent molecular-orbital methods. 22. Small split-valence basis sets for second-row elements. *J. Am. Chem. Soc.* **1982**, *104* (10), 2797-2803. DOI: 10.1021/ja00374a017.

(31) Hariharan, P. C.; Pople, J. A. The influence of polarization functions on molecular orbital hydrogenation energies. *Theoretica chimica acta* **1973**, *28* (3), 213-222. DOI: 10.1007/BF00533485.

(32) Hehre, W. J.; Ditchfield, R.; Pople, J. A. Self—Consistent Molecular Orbital Methods. XII. Further Extensions of Gaussian—Type Basis Sets for Use in Molecular Orbital Studies of Organic Molecules. *J. Chem. Phys.* **1972**, *56* (5), 2257-2261. DOI: 10.1063/1.1677527.

(33) Tokmakov, G. P.; Grandberg, I. I. Reaction of 3,4-dihydro-2H-thiopyran with phenylhydrazines. Synthesis of homothiotryptophols. *Chem. Heterocycl. Compd.* **1989**, *25* (10), 1134-1139. DOI: 10.1007/BF00470691.

(34) Shafizadeh, F.; Furneaux, R. H.; Stevenson, T. T. Some reactions of levoglucosenone. *Carbohydr. Res.* **1979**, *71* (1), 169-191. DOI: 10.1016/S0008-6215(00)86069-3.

(35) Köll, P.; Steinweg, E.; Meyer, B.; Metzger, J. Darstellung ungesättigter Kohlenhydrate durch Esterpyrolyse, II. Thermische cis-Eliminierungen aus vollständig acetylierten Aldopyranosen. *Liebigs Ann. Chem.* **1982**, *1982* (6), 1039-1051. DOI: 10.1002/jlac.198219820605.

(36) Nayak, U. G.; Whistler, R. L. Synthesis of 5-thio-D-glucose. *J. Org. Chem.* **1969**, *34* (1), 97-100. DOI: 10.1021/jo00838a023.

(37) Liao, X. X.; Vetvicka, V.; Crich, D. Synthesis and Evaluation of 1,5-Dithia-D-laminaribiose, Triose, and Tetraose as Truncated beta-(1 -> 3)-Glucan Mimetics. *J. Org. Chem.* **2018**, *83* (24), 14894-14904, Article. DOI: 10.1021/acs.joc.8b01645.
